# Development and validation of a photographic food atlas for portion size estimation in Singapore

**DOI:** 10.3389/fnut.2026.1930409

**Published:** 2026-09-18

**Authors:** Shu Jin Ong, Geraldine Songlen Lim, Jia En Valerie Sin, Joanna Khoo, Joanne Sheot Harn Chan, Kyaw Thu Aung

**Affiliations:** 1 National Centre for Food Science, Singapore Food Agency, Singapore, Singapore; 2 Department of Food Science & Technology, Faculty of Science, National University of Singapore, Singapore, Singapore; 3 School of Biological Sciences, Nanyang Technological University, Singapore, Singapore

**Keywords:** dietary assessment, food consumption, multi-ethnic population, photographic food atlas, portion size estimation, Singapore, validation study

## Abstract

**Background:**

Accurate portion size estimation is essential for dietary assessment, yet existing food atlases have limited applicability in Singapore due to its multi-ethnic culinary heritage and unique dietary patterns. This study describes the development and validation of the first comprehensive photographic food atlas designed specifically for Singapore.

**Method:**

The atlas was developed using two primary data sources, the 2021 Health Promotion Board Energy and Nutrient Composition of Food database and 24-h dietary recall survey from Singapore’s 1st Total Diet Study, to ensure representativeness of commonly consumed foods. Following systematic exclusion criteria, 298 atlas entries were included, representing foods in specific forms or presentations. Of these, 67 entries were series photographs and 231 entries were guide photographs. Validation involved 41 adult participants, recruited through convenience sampling from the national reference laboratory for food science. Participants evaluated 14 entries presented as photo series by selecting the images that best matched corresponding pre-weighed food portions, with two different portions tested per entry. Accuracy was assessed using mean differences, standard deviations, quadratic weighted kappa coefficients, and Spearman coefficients between selected and actual photograph numbers. Additional usability evaluation and participant feedback were also collected.

**Results:**

Of the 14 entries tested, 11 (78.6%) met the predefined validation criteria (mean difference < |0.3| and standard deviation < 1). Three entries (beef, peanut, and fish) showed underestimation. Quadratic weighted kappa coefficients ranged from 0.23 to 0.96, while Spearman coefficients ranged from 0.44 to 0.96. Most entries demonstrated good agreement and strong rank-order association. The proportion of correct responses decreased as portion size increased (96.7% for small portions vs. 42.4% for extra-large portions). Most participants rated the atlas favourably for representativeness (76%), portion size appropriateness (73%), and confidence in using the atlas for portion size estimation (71%).

**Conclusion:**

This first comprehensive photographic food atlas for Singapore comprises 298 entries of commonly consumed foods. Preliminary validation of 14 selected series entries demonstrated satisfactory accuracy, identifying areas for future refinement, particularly regarding presentation methods for certain entries. Overall, the study demonstrates that the atlas can be useful as a practical, localised tool to support dietary assessment of relevant foods in research and policy.

## 1 Introduction

Dietary assessment is the systematic measurement of individual food consumption, including type, quantity, and occasion, which is fundamental to understanding the relationship between diet and health outcomes (1). This information can subsequently be used to assess dietary exposure to both nutrients and chemical contaminants, informing food safety and risk management strategies.

Estimation of consumption quantity is key for dietary assessment yet obtaining accurate dietary intake data is inherently challenging, with portion size estimation being one of the key factors affecting accuracy (2). The gold standard for portion size estimation involves weighing food before and after consumption. However, this method is not feasible for large-scale surveys due to its time-consuming nature and high level of participant commitment required (3). To address this limitation, various portion size estimation tools have been developed, including the use of household measures, food models, and food photographs (4).

Food photographs have been widely adopted in dietary surveys due to their practical advantages: easy to use, inexpensive, and allow the coverage of a broad range of foods (4). Food atlases are structured photograph collections showing different foods in various portion sizes (5). These visual aids help respondents recall and quantify the amount of food consumed by linking visual appearance to corresponding weights. This reduces reliance on subjective interpretations, preconceived notions of portion sizes, and potential miscommunication between interviewer and participant.

Food atlases have been developed internationally across Europe, North and South America, the Middle East, Africa, and Asia (4, 5). They are usually country-specific, developed based on local food availability and dietary patterns. Consumption habits vary across geographical locations, where cultural influences impact dietary practices, food preparation methods, and serving presentations. Thus, this reinforces the importance of country-specific atlases to ensure accuracy and relevance (5). Recent developments have seen some countries transitioning towards digital formats to enhance functionality and accessibility (5–7).

Singapore presents a unique dietary landscape characterised by its multi-ethnic population and diverse culinary heritage (8). Several tools for dietary assessment are currently available in Singapore, including the My Healthy Plate visual guide (9) and a compendium of food pictures with a set of household measures used in the National Nutrition Survey (10). Additionally, mobile applications such as MyFitnessPal and Healthy 365’s Log My Meal are available, and (11) demonstrated the feasibility of software platforms for portion size selection based on caloric increments.

While these tools are useful for dietary assessment, a comprehensive food atlas offers distinct advantages through its coverage of a wider range of commonly consumed foods with typical weight-based portion sizes, and its practical utility as an accessible, standardised tool for accurate dietary assessment across varied research and clinical settings.

To the best of our knowledge, Singapore lacks a comprehensive, validated food atlas developed based on local dietary consumption patterns to date. To address this gap, this study aimed to develop and validate the first comprehensive food atlas specific to Singapore, incorporating commonly consumed local foods based on national nutrition databases and food consumption surveys. The atlas was designed as a reference tool for dietary assessment that could be adapted for future digital applications.

## 2 Materials and methods

### 2.1 Atlas development

#### 2.1.1 Selection of foods

The initial food list was consolidated from two primary comprehensive data sources: the 2021 Health Promotion Board Energy and Nutrient Composition of Food database, which contains a list of basic ingredients and foods consumed in Singapore (*n* = 3,719), and foods identified from a nationally representative 24-h dietary recall survey conducted in 2021 in support of Singapore’s 1st Total Diet Study (*n* = 91). The 24-h dietary recall survey employed stratified random sampling to obtain dietary data from 2,000 respondents aged 15 and above as described elsewhere (8). Together, these complementary data sources captured foods that are both commonly available and commonly consumed in Singapore.

Foods for inclusion in the food atlas were selected using a systematic methodological approach to ensure relevance and practicality (Figure 1). Foods that were raw or required further preparation before consumption were excluded because dietary assessment captures foods in a ready-to-consume form. Composite dishes whose principal components were already represented individually within the atlas were generally excluded to minimise redundancy and allow flexible estimation of individual food portions; for example, braised chicken rice could be represented through its chicken and rice components. As the atlas is intended to be used in conjunction with dietary assessment systems that apply standard recipes for composite dishes, this approach does not preclude estimation of mixed-dish consumption.

**Figure 1:**
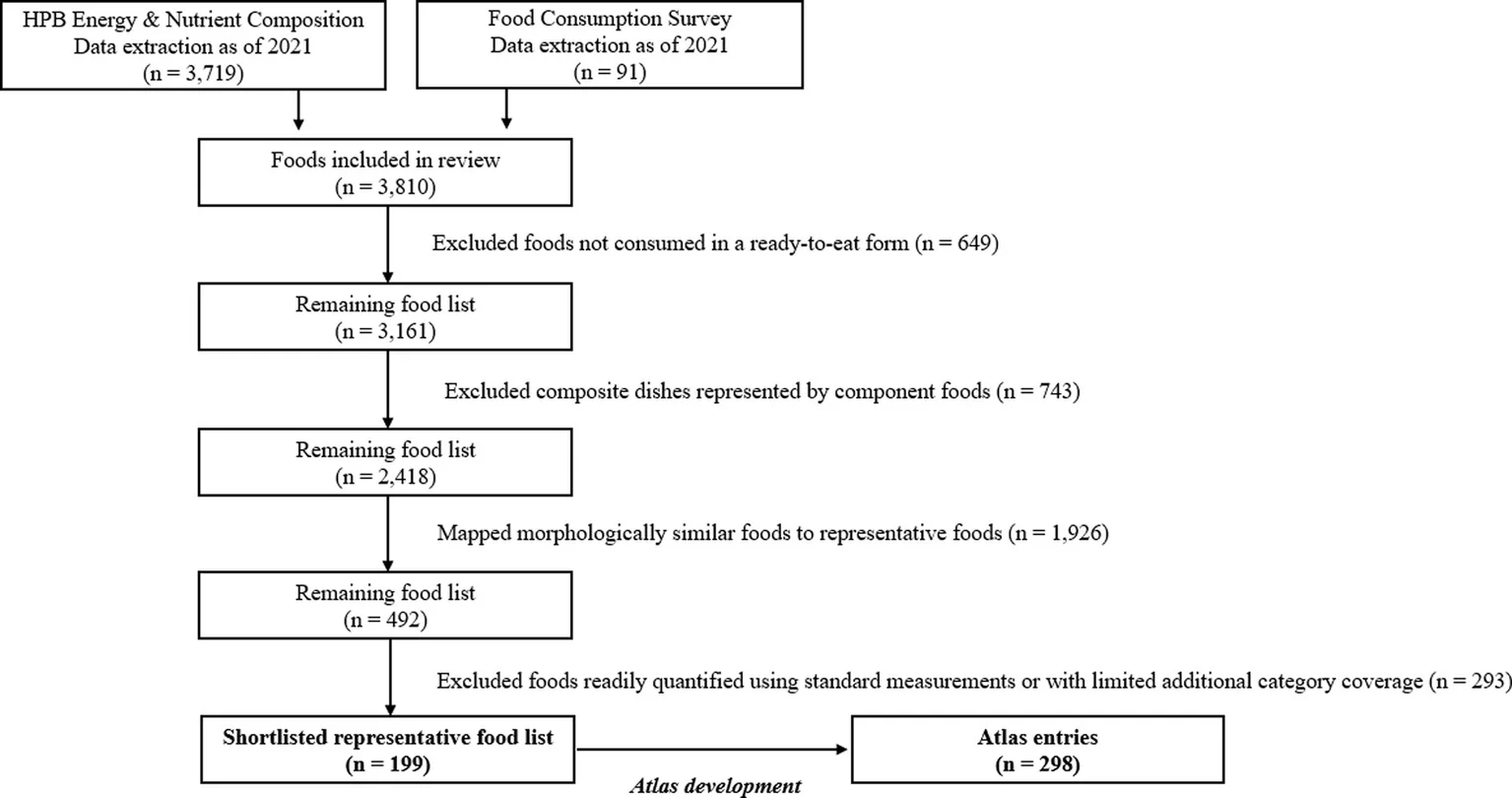
Flow diagram for selection of foods.

Foods with similar visual characteristics were mapped to representative foods according to morphology and presentation, where such differences were not expected to materially influence portion size estimation. Foods that could be readily quantified using standard measurements (e.g., beverages commonly reported by volume) were selectively excluded, as photographic representation was not considered necessary for portion size estimation. Finally, foods that did not improve coverage within a food category were excluded to maintain a manageable atlas scope while preserving representation across major food groups.

Following systematic exclusion, 199 foods were shortlisted for inclusion in the atlas. The selected foods were designed to provide broad coverage of commonly consumed food groups and food morphologies from the source data.

The foods were represented as atlas entries, with each entry referring to a single representation in a specific form, size, or presentation, which may include one or more portion images. Some foods were presented as multiple entries to capture variation, resulting in a total of 298 atlas entries.

#### 2.1.2 Food purchase and preparation

The foods were sourced from local restaurants, eateries, bakeries, supermarkets, and convenience stores in Singapore to represent typical serving sizes that were accessible and commonly consumed by the population. As the foods were cooked and ready to consume, minimal preparation was required to represent the state in which they are usually eaten. Where necessary, this mainly involved the removal of inedible parts (such as skin, shell, seeds, and bones).

#### 2.1.3 Type of photographs

Based on previous studies, two types of photographs were commonly used for atlas development: series photographs and guide photographs (4, 12–14). A series of photographs shows incremental portion sizes for variable-portion foods such as rice, noodles, and vegetables (Figure 2). A guide photograph is used to represent foods typically consumed as standard units such as fruit and bread, and foods consumed in relatively small quantities such as sauces and condiments where series photography would be impractical (Figures 3, 4). This approach is consistent with other studies conducted in Ecuador, Japan, and the Middle East.

**Figure 2:**
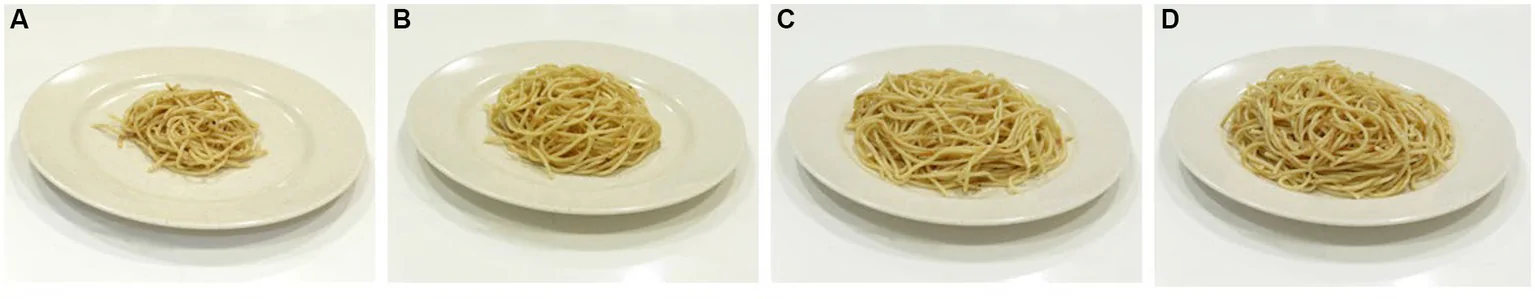
Example of series photographs for Pasta, spaghetti, with increasing portion size from **(A–D)**.

**Figure 3:**
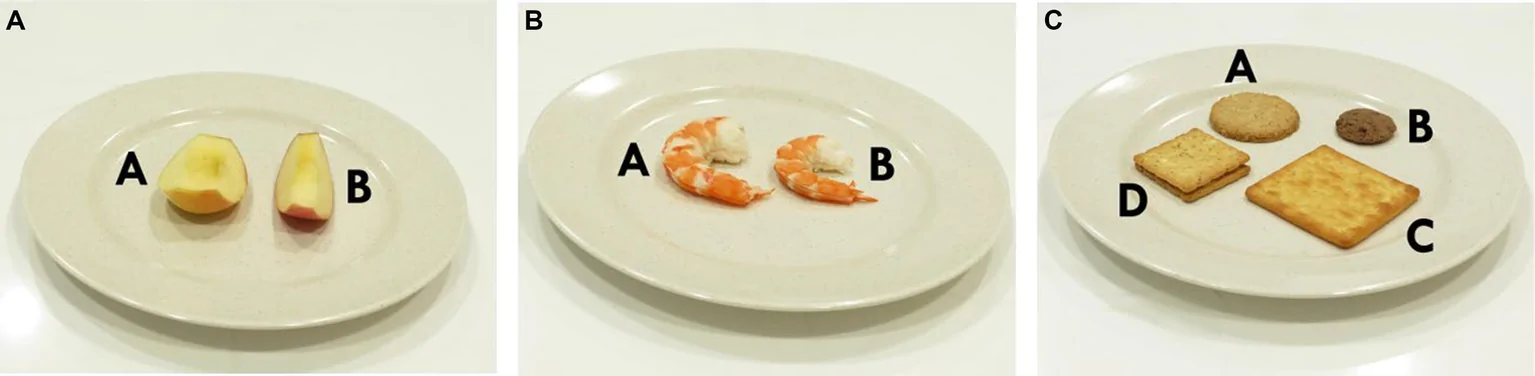
Examples of guide photographs with discrete units. **(A)** Apple, sliced, **(B)** Prawn, king, deshelled, **(C)** Biscuit, assorted.

**Figure 4:**
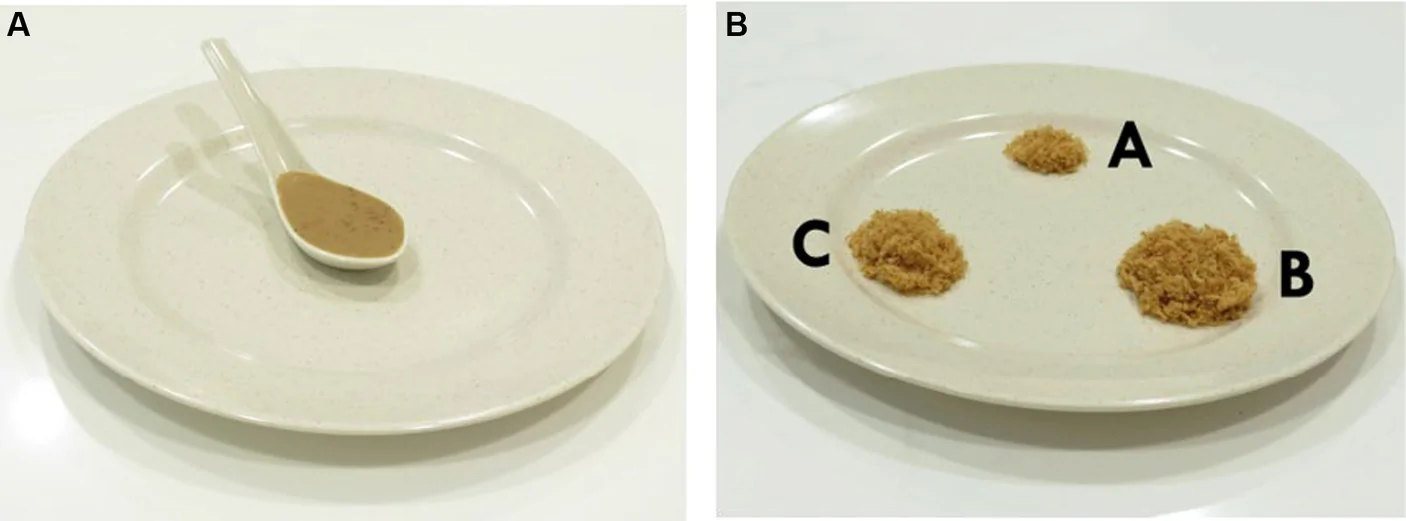
Examples of guide photographs of foods commonly consumed in small quantities. **(A)** Sauce, cream-based, **(B)** Meat floss.

Each entry was classified into one of these photograph types. In cases the entry could be represented in both types of photographs, the decision was based on the approach that best reflected typical consumption patterns as well as practical considerations such as visual clarity and usability. This resulted in 67 entries presented as series photographs and 231 entries as guide photographs.

#### 2.1.4 Portion size representation

The number of photographs and corresponding edible weights for each atlas entry are provided in Supplementary Table S1.

For guide photographs, foods were purchased and presented as they are commonly prepared and served. Selected foods within the same category were captured in the same photograph to facilitate relative comparison of size and type.

For series photographs, portion size ranges represented typical portions likely to be consumed. These were primarily anchored to the serving size of the purchased food, as sold or indicated on product packaging where available. This was supplemented by observations of locally available portions and, for selected entries where relevant data were available, reference checks against the national nutrition database and national food consumption survey. Minimum and maximum portions were selected as practical bounds rather than absolute limits of consumption.

Within each entry, the defined range was represented using food-specific increments that provided observable differences between adjacent portions, thereby supporting effective use of the photographic scale. The number of photographs and corresponding weights for each entry are provided in Supplementary Table S1.

#### 2.1.5 Preparation of tableware

A standard tableware set was used in the development of the food atlas to ensure consistency across photographs and enable relative comparison. Plain coloured tableware was selected to provide good contrast against the food for optimal visualisation. The tableware included various plates, bowls, cups, and measuring spoons, with commonly used tableware used to aid recognition when estimating portion size.

Photographs of the tableware were also included in the food atlas as a reference tool for estimation of portion size (Figure 5).

**Figure 5:**
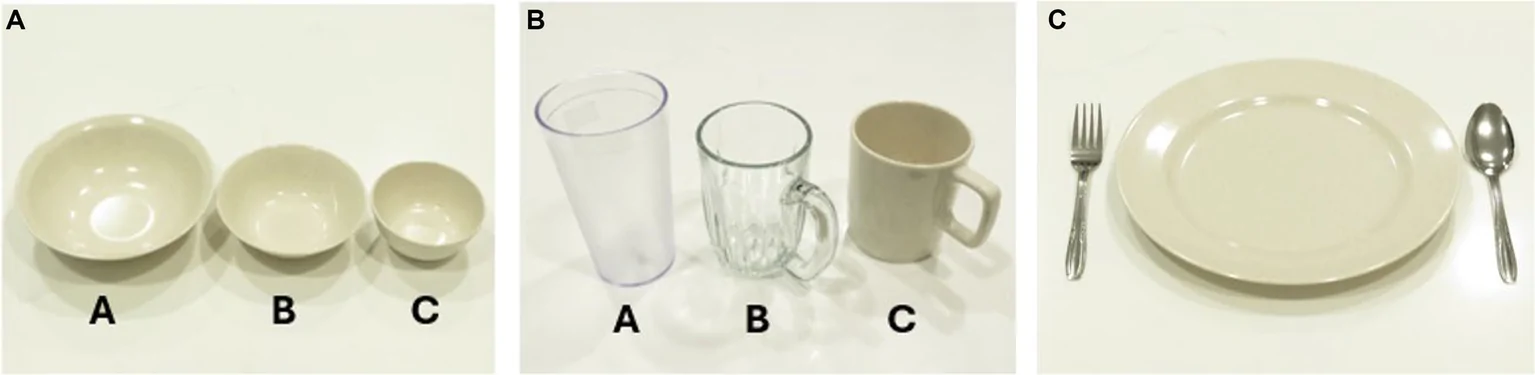
Examples of household measurement items. **(A)** Bowls (diameter = 20 cm, 15 cm, 11.5 cm), **(B)** Cups (volume = 325 mL, 220 mL, 270 mL), **(C)** Plate with fork and spoon.

#### 2.1.6 Weighing

Foods were weighed using an electronic kitchen scale (Weiheng, WH-B05). The scale has a maximum weighing capacity of 5 kg, with a precision of 1 g. For foods weighing less than 5 g, an electronic scientific scale (Sartorius, CPA2202S) was used, with a precision of 0.1 g.

#### 2.1.7 Photography

Photos were taken using a Canon PowerShot SX40 HS, at an angle of 45° to represent the typical viewing angle of a person sitting at a table. This angle provides an optimal balance between capturing the height and depth of the object, while maintaining a natural perspective that is relevant to everyday dining experience. A shutter speed of 1/25 s, with aperture of f/4, and ISO 100 were used with consistent artificial lighting to ensure uniform exposure and white balance across all photographs. The camera was mounted on a tripod to maintain consistent angles, and all photos were taken in the same room to ensure consistent lighting throughout. Photos were reviewed and edited using Microsoft Photos software to standardise image presentation, including minor adjustments and formatting for the atlas. Each photo was assigned a unique photo identification code.

#### 2.1.8 Atlas database

The entries were organised into 16 categories (Table 1). Information on the number of photographs and corresponding edible weights for each atlas entry was compiled with the photographs to develop the food atlas (Supplementary Table S1, Supplementary Figure S1).

**Table 1:** List of broad categories.

Category
Bakery products
Beverages
Composite foods
Confectionery
Egg and egg products
Fish and seafood
Fruit and fruit products
Fungi and seaweed
Grain and grain-based products
Meat and meat products
Milk and dairy products
Nuts and seeds
Ready-to-eat savouries
Sauces and condiments
Vegetables
Vegetable protein

### 2.2 Atlas validation

#### 2.2.1 Selection of entries for validation

A validation study was conducted to assess the accuracy of portion size estimation using the developed atlas. Validation focused on series photographs with a minimum of four images. Out of the 67 entries with series photographs, 38 entries had a minimum of four images, with 14 being selected for validation (Table 2). Selection aimed to provide representation across food categories, morphologies, and textures, while also considering the practical feasibility of conducting the study. The number of entries selected was consistent with other validation studies, which ranged from 9 to 23 (2, 4, 15–17).

**Table 2:** List of entries for validation.

Category	Selected entry for validation
Composite foods	Soup, cream
Egg and egg products	Egg, scrambled
Fish and seafood	Fish, white, boneless, sliced
Fruit and fruit products	Blueberry
Fruit and fruit products	Strawberry
Fungi and seaweed	Mushroom, sliced
Grains and grain-based products	Pasta, spaghetti
Grains and grain-based products	Rice, white, regular
Meat and meat products	Beef, lean, sliced
Meat and meat products	Chicken, boneless, pieces
Nuts and seeds	Peanut
Vegetables (Brassica)	Broccoli, florets
Vegetables (leafy vegetables, herbs)	Beansprout
Vegetables (leafy vegetables, herbs)	Kailan

#### 2.2.2 Recruitment of study participants

Recruitment of study participants was through convenience sampling where an email invitation was sent to approximately 130 staff from the national reference laboratory for food science, of whom 42 accepted the invite. Eligible participants were aged 18 and above, able to commit to the study duration, and had no conditions affecting visual perception. Validation guidelines required a minimum sample size of 32 participants (18), and 41 were eventually recruited in this study to account for potential attrition. Written consent was obtained, and participants were assigned unique identifiers for anonymity and data management throughout the study.

#### 2.2.3 Study approach and design

The perception approach was used to validate the atlas entries (18). Participants were presented with pre-weighed foods plated on tableware identical to that used in the food atlas and tasked to select the photograph within each entry that best represented the portion size. Two different portion sizes were randomly selected from each of the 14 entries, resulting in a total of 28 portions evaluated (Supplementary Table S1).

The evaluation was conducted over two sessions, with all participants completing both sessions. Each participant evaluated the same 14 entries in both sessions, with a different portion size presented for each entry in each session. This approach was used to prevent respondent fatigue and to ensure that the same entry was not evaluated more than once within a single session. The presentation order of the entries was randomly assigned using Microsoft Excel’s RAND function and remained the same across both evaluation sessions. For each entry, two portion sizes were randomly selected from the available photographs, with one presented in the first session and the other in the second session.

The first and second sessions were conducted 1 week apart in November 2024. Foods were purchased in accordance with Section 2.1.2 before each session and stored appropriately before use. To maintain consistency between sessions, foods were sourced from the same establishments where possible. On evaluation days, foods were weighed and plated using standard tableware, then arranged at the study site with their corresponding photographs presented in printed format. Validation was conducted in the same room under consistent indoor lighting conditions for both sessions, with standardised tableware, food placement, and labelling maintained across sessions. The site accommodated up to four participants at a time, who completed the evaluations individually, with staff present to oversee the evaluation process.

Participants were informed of the study objectives and procedures at the beginning of the evaluation, before receiving a paper-based questionnaire containing their assigned unique identifier. The questionnaire comprised demographic questions (age, sex, and self-reported familiarity with accurate measurements, such as weighing or volumetric measurements) and a validation section with multiple choice questions requiring participants to select photographs corresponding to the presented foods.

Additional usability evaluation and participant feedback were collected via the questionnaire. Participants were asked to rate the representativeness of the atlas entries, appropriateness of the portion size ranges, and confidence in using the atlas for portion size estimation using specified response categories. For reporting purposes, adjacent positive response categories were combined for the questions on representativeness and confidence. Participants were also invited to provide suggestions for improving the usability of the food atlas through specified response options and free-text comments. Open-ended responses were reviewed descriptively, and overlapping suggestions were grouped to identify common areas for improvement.

A template of the validation questionnaire is provided in Data Sheet 1.

#### 2.2.4 Statistical analysis

All statistical analyses were conducted using Microsoft Excel and R version 4.6.1.

The results from both evaluation sessions were pooled for analysis. Photographs were numbered sequentially from 1 to 4, representing smallest to largest portions. The proportion of participants selecting the correct photograph, adjacent photographs (within 1 position), and distant photographs (2 or more positions away) was calculated.

Mean differences with 95% confidence intervals and standard deviations were calculated between selected and actual photograph numbers. The acceptable limit of bias for the mean difference of |0.3| was derived using a 99.999% confidence interval based on the distribution of normalised mean differences across evaluated entries, following established validation studies. The use of a highly conservative confidence interval was intended to account for inherent variability in portion size estimation and to avoid over-interpretation of small deviations unlikely to be of practical relevance (2, 19). The acceptable standard deviation was set at < 1, since selecting adjacent photographs (i.e., one portion size above or below the actual size) was considered satisfactory (2, 17).

Quadratic weighted kappa coefficients with 95% confidence intervals were calculated to assess agreement between selected and actual photograph numbers while accounting for the ordinal scale. Spearman coefficients were calculated to assess rank-order association between selected and actual photograph numbers. To account for repeated observations contributed by each participant, a linear mixed-effects model with atlas entry as a fixed effect and participant as a random effect was applied.

Details of the statistical models are provided in Data Sheet 2.

## 3 Results

The food atlas comprised 298 entries, with 67 represented as series photographs and 231 represented as guide photographs. Validation findings reported in this study relate to the 14 selected series entries assessed. Guide entries were not prioritised for validation, as they primarily serve as visual references for foods consumed as discrete units, standard servings, or small measured quantities.

### 3.1 Series photographs

Series photographs were developed for entries representing foods commonly consumed in variable portion sizes, with each entry shown in a sequence of 2 to 4 portions with progressive increments. The 67 entries (22.5%) covered 13 categories, with key representation for grain and grain-based products, vegetables, meat and meat products, and fish and seafood. The list of entries represented as series photographs can be found in Supplementary Table S1.

### 3.2 Guide photographs

Guide photographs were developed for entries representing foods typically served in discrete countable units or condiments consumed in small, measured quantities. The 231 entries (77.5%) covered 14 categories, including single servings of bakery products and measured quantities for sauces and condiments. The list of entries represented as guide photographs can be found in Supplementary Table S1.

### 3.3 Household measurement

Commonly used household measurement items were photographed to provide reference points for portion size estimation, including bowls, cups, plates, and spoons of various sizes. Table 3 shows the specifications of the household measurement items.

**Table 3:** List of household measurement items.

Household items	Specification	Photo ID
Bowl	Diameter = 20 cm	R01A
Bowl	Diameter = 15 cm	R01B
Bowl	Diameter = 11.5 cm	R01C
Cup	Volume = 325 mL	R02A
Cup	Volume = 220 mL	R02B
Cup	Volume = 270 mL	R02C
Plate	Diameter = 25 cm	R03A
Plate	Diameter = 17.5 cm	R03B
Plate	Diameter = 12.5 cm	R03C
Saucer	Diameter = 9.5 cm	R03D
Spoon	Teaspoon	R04A
Spoon	Tablespoon	R04B
Spoon	Soup spoon	R04C
Standard cutlery set	—	R05

### 3.4 Demographics of participants

A total of 41 participants were recruited for the validation study. The participants were predominantly female (75.6%), with most participants aged between 30 to 40 years (48.8%). Table 4 shows the age group, sex, and self-reported familiarity with accurate measurement among the study participants.

**Table 4:** Age group, sex, and self-reported familiarity with accurate measurement among study participants (n = 41).

Age	Male	Female	Total
18 to 30 years	1 (2.4%)	9 (22.0%)	10 (24.4%)
30 to 40 years	8 (19.5%)	12 (29.3%)	20 (48.8%)
Above 40 years	1 (2.4%)	10 (24.4%)	11 (26.8%)
Total	10 (24.4%)	31 (75.6%)	41 (100.0%)

Self-reported familiarity with accurate measurement	*n*
Not familiar	14 (34.1%)
Somewhat familiar	23 (56.1%)
Very familiar	4 (9.8%)
Total	41 (100.0%)

### 3.5 Proportion of correct, adjacent, and distant responses

Each entry was shown in two different portion sizes across both sessions, with 28 total evaluations per participant. The proportion of correct responses varied across entries, with beansprout having the highest proportion of correct responses (97.6%) and peanut having the lowest (39.0%). Table 5 shows the proportion of correct, adjacent, and distant responses for each evaluated entry.

**Table 5:** Proportion of correct, adjacent, and distant responses for evaluated entries.

Entry name	Correct (%)	Adjacent (%) (± 1 position)	Distant (%) (≥2 positions)
Soup, cream	76.8	23.2	0.0
Egg, scrambled	90.2	9.8	0.0
Fish, white, boneless, sliced	51.2	47.6	1.2
Blueberry	93.9	6.1	0.0
Strawberry	89.0	11.0	0.0
Mushroom, sliced	69.5	30.5	0.0
Pasta, spaghetti	79.3	20.7	0.0
Rice, white, regular	90.2	9.8	0.0
Beef, lean, sliced	56.1	41.5	2.4
Chicken, boneless, pieces	75.6	24.4	0.0
Peanut	39.0	61.0	0.0
Broccoli, florets	81.7	18.3	0.0
Beansprout	97.6	2.4	0.0
Kailan	91.5	8.5	0.0

The photograph series for each entry was numbered sequentially from 1 to 4 based on increasing portion sizes, assigned as small, medium, large, and extra-large. The proportion of correct responses decreased as evaluated portion size increased, with “small” showing 96.7% correct responses as compared to “extra-large” with 42.4% correct responses. A trend towards lower accuracy and greater underestimation was observed with increasing portion size. However, these findings should be interpreted cautiously because different entries contributed to each portion size category. In addition, the bounded nature of the photograph scale constrains the direction of error for the smallest and largest portions. Table 6 shows the proportion of correct responses and direction of errors.

**Table 6:** Proportion of correct responses and direction of errors for the evaluated portion sizes.

Portion size	No. of entries	% Correct	% Underestimation	% Overestimation
Small	9	96.7	—	3.3
Medium	8	85.7	7.3	7.0
Large	6	65.9	27.2	6.9
Extra-large	5	42.4	56.1	—

### 3.6 Mean difference, standard deviation, weighted kappa coefficient, Spearman coefficient

The acceptable range was defined as mean difference between selected and actual photograph numbers < |0.3| and standard deviation < 1, based on established validation criteria (2, 17). Of the 14 entries evaluated, 11 met the criteria. Three entries did not meet these criteria: fish (mean difference = −0.43, SD = 0.59), beef (mean difference = −0.46, SD = 0.55), and peanut (mean difference = −0.61, SD = 0.49), all showing underestimation.

Quadratic weighted kappa coefficients ranged from 0.23 to 0.96, with 12 entries demonstrating good agreement (κ > 0.60). Spearman coefficients ranged from 0.44 to 0.96, with 12 entries demonstrating strong monotonic association (ρ > 0.70). Peanut and beef had the weakest agreement and rank-order association among the evaluated entries. The mean differences, standard deviations, weighted kappa coefficients, and Spearman coefficients for each evaluated entry are presented in Table 7.

**Table 7:** Mean difference, standard deviation, weighted kappa coefficient, and Spearman coefficient for evaluated entries.

Entry name	Mean difference (95% CI)	S.D.	Weighted kappa coefficient (95% CI)	Spearman coefficient
Soup, cream	−0.21 (−0.30 to −0.11)	0.44	0.64 (0.53–0.76)	0.71
Egg, scrambled	−0.07 (−0.14 to −0.01)	0.31	0.80 (0.68–0.93)	0.81
Fish, white, boneless, sliced	−0.43 (−0.56 to −0.30)	0.59	0.85 (0.81–0.89)	0.96
Blueberry	−0.01 (−0.07 to 0.04)	0.25	0.88 (0.79–0.98)	0.90
Strawberry	−0.09 (−0.16 to −0.01)	0.32	0.82 (0.72–0.91)	0.87
Mushroom, sliced	−0.23 (−0.34 to −0.12)	0.50	0.83 (0.76–0.89)	0.91
Pasta, spaghetti	0.21 (0.12 to 0.30)	0.41	0.92 (0.89–0.95)	0.94
Rice, white, regular	0.05 (−0.02 to 0.12)	0.31	0.84 (0.76–0.93)	0.91
Beef, lean, sliced	−0.46 (−0.58 to −0.34)	0.55	0.45 (0.34–0.57)	0.62
Chicken, boneless, pieces	−0.20 (−0.30 to −0.09)	0.46	0.94 (0.91–0.96)	0.93
Peanut	−0.61 (−0.72 to −0.50)	0.49	0.23 (0.16–0.30)	0.44
Broccoli, florets	0.18 (0.10 to 0.27)	0.39	0.79 (0.73–0.85)	0.95
Beansprout	−0.02 (−0.06 to 0.01)	0.16	0.95 (0.88–1.02)	0.95
Kailan	−0.06 (−0.12 to 0.00)	0.29	0.96 (0.93–0.99)	0.96

In the linear mixed-effects model, atlas entry was included as a fixed effect to account for systematic differences across food types, with participant as a random effect. Fixed effect estimates showed larger negative estimates for fish, beef, and peanut (Supplementary Figure S3), consistent with the results reported in Table 7. The participant-level variance was small relative to the residual variance, indicating minimal clustering by participant and suggesting that repeated observations within individuals did not materially influence the study findings (Table 8).

**Table 8:** Linear mixed effects model.

Random effect	Variance	Std. Dev.
Participant (intercept)	0.01	0.07
Residual	0.16	0.40

### 3.7 Usability evaluation

The usability evaluation covered three aspects: representativeness of the selected entries, appropriateness of the portion size ranges, and confidence in using the atlas for portion size estimation. Overall, participants provided favourable ratings across all three aspects. Most participants (76%) considered the selected entries to be mostly or highly representative of a typical diet. Similarly, 73% reported that the portion size ranges in the atlas were appropriate for the majority of evaluated entries (10–14 entries), while 71% were mostly or highly confident in using the atlas to estimate portion sizes.

## 4 Discussion

Singapore’s multi-ethnic culinary heritage and hawker culture, coupled with easy access to diverse foods, create unique dietary patterns that limit the applicability of existing food atlases developed for other populations. The developed atlas represents the first comprehensive food atlas designed for Singapore, based on the national nutrition database and dietary survey data to ensure representativeness and relevance to the local population. The portion size ranges were based on market availability to reflect the local culture of eating out, given that 46% of the Singapore population consumes food prepared outside the home (8).

A systematic approach was adopted, prioritising key criteria to ensure that commonly consumed foods across the population were adequately represented. This selection process maximises the utility of the atlas while maintaining a practical scope. Beyond its defined food list, the atlas may provide a useful reference for foods with similar physical attributes (4) when exact matches are unavailable. Formal evaluation should be conducted to support its applicability beyond the foods represented.

To address technical challenges during development including photographic presentation and selection of suitable portion increments, this study prioritised consistency and visual clarity across all entries. This was considered an appropriate mitigating approach given that real-world presentations will inevitably vary from these standardised presentations.

A validation study was conducted on selected series photograph entries to evaluate the atlas’s accuracy as a dietary assessment tool. As guide photograph entries were not evaluated, their accuracy in supporting portion size estimation remains to be established and represents an important direction for future work. The preliminary validation outcomes align well with a Balkan study using a similar methodology. The proportion of respondents making correct selections (77.3%) compares favourably with their reported rate of 60.3%, while the combined proportion of correct and adjacent selections (99.7%) is consistent with their reported rate of >98% (2).

Of the 14 entries evaluated, 11 (78.6%) met the predefined criteria for mean difference < |0.3| and standard deviation < 1, indicating that the atlas provides acceptable accuracy for portion size estimation for most evaluated entries. The proportion of entries meeting the criteria is comparable with other validation studies using the same basis for establishing acceptance criteria (99.999% CI), with 80% meeting the predefined criteria among lay individuals (2).

Three entries failed to meet the predefined criteria: beef (mean difference = −0.46), peanut (mean difference = −0.61), and fish (mean difference = −0.43), all indicating underestimation. For sliced meat products, a validation study in China similarly reported underestimation of pork (15). Notably, it showed that the underestimation was larger with atlas use compared to without, highlighting the limitations of photographic tools in capturing the morphology of such food types.

Similarly, an Argentine validation study also showed underestimation of roasted peanut (20), consistent with the observed underestimation for the peanut entry. This demonstrates that difficulties with portion estimation exist even for uniformly shaped, discrete foods in contrast to the blueberry entry (mean difference = −0.01), which met the validation criteria. It is possible that the smaller size of peanuts and their variable form (half or whole pieces) enable a denser packing when placed on a plate, where subtle differences in plating and arrangement may have reduced the visual distinctness between portion size increments.

Interestingly, a European validation study found fish slices performed well using a much larger portion range of 87 g – 298 g (17), compared to our portion range of 24 g–96 g for the fish entry. One possible explanation is that the wider range and larger portion increments used in the European study provided greater visual differentiation between adjacent photographs, which may have improved participants’ ability to distinguish between portion sizes. Plate size may also influence portion size estimation, as larger plates are associated with a decrease in perceived portion size (21). However, the plate size used in this study (25 cm) was comparable to that used in the European validation study (26 cm), suggesting that plate size was unlikely to be a significant contributing factor.

Quadratic weighted kappa and Spearman coefficients were used to evaluate performance in terms of agreement and rank-order association, respectively. Based on the results in Table 7, most entries showed strong agreement and rank order association, indicating that participants were generally able to identify the correct photograph and distinguish relative portion sizes within the same entry.

Peanut and beef demonstrated the weakest agreement (*κ* = 0.23 and 0.45, respectively) and rank-order association (*ρ* = 0.44 and 0.62, respectively) among the evaluated entries. This is consistent with their larger negative mean differences and lower proportions of correct responses. These findings suggest that the visual characteristics of certain food types may make accurate portion size discrimination more challenging.

The portions selected for evaluation of these entries were adjacent “large” and “extra-large” portions, which may have contributed to the poorer performance. In contrast, fish showed better agreement (*κ* = 0.85) and rank-order association (*ρ* = 0.96), despite not meeting the predefined criteria for mean difference. This may be attributed to the greater visual distinction between “small” and “extra-large” portions tested for fish.

A decrease in correct responses was observed with increasing portion size (96.7% for “small” vs. 42.4% for “extra-largee”), which may indicate a tendency to underestimate large and extra-large portion sizes. However, it should be noted that different entries contributed to each portion size category. In addition, the bounded nature of the photograph scale constrains the direction of error for the smallest and largest portions. Despite these limitations, the observation is consistent with the flat-slope phenomenon (22) and has also been reported in other validation studies (2). This may suggest that larger increments between portion sizes could improve estimation accuracy for some entries.

For the usability evaluation, participants provided favourable ratings for representativeness (76% mostly or highly representative), portion size appropriateness (73%), and confidence in using the atlas for portion size estimation (71% mostly or highly confident). These ratings support the overall approach and design adopted in the atlas development. Participant feedback also identified areas for improvement, including the incorporation of reference objects for scale comparison and better clarity of the photos. These suggested improvements align with practices adopted in other food atlases, such as the use of standardised containers with volume markers for liquids (4, 14, 23) and adoption of photography techniques to enable visualisation of both depth and width and improve background contrast (14, 15).

The scope and design of this validation study present some limitations. Firstly, only 14 of the 67 series entries were validated, representing approximately 21% of all series entries, with only a subset of portion sizes tested within each entry. Guide entries were not prioritised for validation as they primarily serve as visual references for foods consumed as discrete units, standard servings, or small measured quantities. Further validation involving a broader range of series entries, portion sizes, and a representative subset of guide entries would be valuable to confirm the initial validation outcomes and establish the suitability of the atlas for dietary assessment.

Secondly, the participants were recruited through convenience sampling from the national reference laboratory for food science, with under-representation of males. The study population may therefore not be representative of the general population, and participant information was limited to age, sex, and self-reported familiarity with accurate measurement. Future studies involving a larger and more representative sample across age, sex, ethnicity, education, and occupation would increase the generalisability of the findings.

Additionally, the atlas may require age-appropriate photographs for children, given differences in dietary consumption patterns (4, 24, 25). Future digitisation of the food atlas as a mobile application or web-based platform could further enhance accessibility, usability, and facilitate seamless updates to reflect Singapore’s evolving food landscape.

Despite these limitations, the favourable performance observed for most evaluated entries provides preliminary support for the broader applicability of the atlas design. Additional validation may be warranted for entries with morphological characteristics similar to those that demonstrated poorer performance, particularly where visual discrimination between adjacent portion sizes is more challenging.

From an application perspective, while validation was conducted under controlled conditions involving visual matching of pre-weighed foods, the atlas addresses an important gap in dietary assessment by providing a locally developed reference tool for portion size estimation. This has the potential to support dietary surveys by helping respondents recall and report their food intake more accurately. Fieldwork implementation of the atlas in real-life dietary assessment settings would be required to evaluate its impact on downstream estimation of nutrient intake and exposure to chemical contaminants. The atlas may also serve as a practical reference for healthcare professionals during clinical consultations and its design allows for potential adaptation into digital formats, which could further broaden its accessibility across multiple settings.

## 5 Conclusion

This study presents the first comprehensive food atlas developed specifically for Singapore’s diverse food landscape, with good representation of commonly consumed foods and portion sizes based on the national nutrition database and food consumption survey data. Preliminary validation of selected series entries demonstrated satisfactory accuracy, with good agreement and strong rank-order association for most tested entries. Future work could focus on expanding validation to include a wider range of entries and a broader, more representative participant sample, while addressing morphological challenges identified for specific entries through improved photography techniques and reference objects. Overall, the atlas provides a practical reference tool for portion size estimation, with potential to support dietary assessment applications in food safety and nutrition.

## Data Availability

The original contributions presented in the study are included in the article/Supplementary material, further inquiries can be directed to the corresponding author.

## Supplementary material

SUPPLEMENTARY FIGURE S1 Food atlas photo booklet.

SUPPLEMENTARY FIGURE S2 Validation questionnaire.

SUPPLEMENTARY FIGURE S3 Statistical models.

SUPPLEMENTARY TABLE S1 Food atlas database.
