# Supplementary material for: Development and validation of a photographic food atlas for portion size estimation in Singapore
Source: Front Nutr. 2026 Sep 18;13:1930409. doi: 10.3389/fnut.2026.1930409 (PMC13630621; doi:10.3389/fnut.2026.1930409)
Supplement: Supplementary file 1 [file Data_Sheet_1.PDF]

Supplementary Information
S1. Food Atlas Database
Table 1. List of entries in series

Note: Entries with portions included in the validation study are shaded in purple.

S/N	Category	Entry name	Photo ID	No. of portions	Primary tableware (pictured)	Edible portion weight 1 (g)	Edible portion weight 2 (g)	Edible portion weight 3 (g)	Edible portion weight 4 (g)
1	Bakery products	Biscuit, stick, plain	FA0187	4	25cm plate	8	17	25	35
2	Bakery products	Biscuit, stick, with coating	FA0001	4	25cm plate	24	49	72	96
3	Composite foods	Soup, clear	FA0067	2	11.5cm bowl	127	254		
4	Composite foods	Soup, cream	FA0094	4	15cm bowl	59	**118***	**177***	236
5	Confectionery	Chocolate candy	FA0198	2	17.5cm plate	13	27		
6	Confectionery	Tapioca pearl	FA0173	2	12.5cm plate	45	90		
7	Egg and egg products	Egg, scrambled	FA0190	4	25cm plate	**39***	**78***	117	156
8	Fish and seafood	Clam / Lala	FA0120	4	25cm plate	12	21	32	41
9	Fish and seafood	Fish, saba, boneless	FA0056	2	25cm plate	41	82		
10	Fish and seafood	Fish, sardine, mashed	FA0112	2	12.5cm plate	70	140		
11	Fish and seafood	Fish, tuna, canned	FA0110	2	12.5cm plate	37	74		
12	Fish and seafood	Fish, white, boneless, sliced	FA0101	4	25cm plate	**24***	48	72	**96***
13	Fish and seafood	Squid, pieces	FA0155	2	25cm plate	8	16		
14	Fruit and fruit products	Blueberry	FA0033	4	15cm bowl	**66***	**132***	198	264
15	Fruit and fruit products	Papaya, pieces	FA0197	4	25cm plate	65	130	195	261
16	Fruit and fruit products	Raisin, seedless	FA0160	4	12.5cm plate	15	30	45	60
17	Fruit and fruit products	Strawberry	FA0004	4	15cm bowl	68	**136***	**204***	272
18	Fungi and seaweed	Mushroom, sliced	FA0091	4	25cm plate	**22***	44	**66***	89
19	Fungi and seaweed	Seaweed snack, dried	FA0007	4	25cm plate	2	4	6	8
20	Grain and grain-based products	Chee cheong fun (猪肠粉)	FA0065	2	20.5cm plate	153	306		
21	Grain and grain-based products	Congee	FA0095	2	11.5cm bowl	165	330		

22	Grain and grain-based products	Corn, kernel	FA0074	4	25cm plate	23	46	69	92
23	Grain and grain-based products	Noodle, egg	FA0092	2	11.5cm bowl	100	177		
24	Grain and grain-based products	Noodle, thick	FA0071	2	11.5cm bowl	100	200		
25	Grain and grain-based products	Pasta, fusilli	FA0181	4	25cm plate	55	111	166	222
26	Grain and grain-based products	Pasta, macaroni	FA0066	2	11.5cm bowl	80	160		
27	Grain and grain-based products	Pasta, penne	FA0180	4	25cm plate	49	98	147	196
28	Grain and grain-based products	Pasta, spaghetti	FA0169	4	25cm plate	**67***	135	**203***	270
29	Grain and grain-based products	Porridge	FA0194	4	15cm bowl	144	288	432	575
30	Grain and grain-based products	Rice noodle, bee hoon, thin, fried	FA0059	2	11.5cm bowl	55	110		
31	Grain and grain-based products	Rice noodle, bee hoon, thick, fried	FA0195	4	20cm bowl	113	226	339	452
32	Grain and grain-based products	Rice, white, japonica	FA0081	2	25cm plate	118	236		
33	Grain and grain-based products	Rice, white, regular	FA0193	4	25cm plate	**100***	**200***	300	400
34	Meat and meat products	Beef, lean, sliced	FA0191	4	25cm plate	28	56	**84***	**112***
35	Meat and meat products	Beef, with fat, sliced	FA0079	4	25cm plate	49	98	147	196
36	Meat and meat products	Chicken, boneless, pieces	FA0192	4	25cm plate	**45***	90	135	**180***
37	Meat and meat products	Chicken, leg, boneless	FA0140	2	25cm plate	67	133		
38	Meat and meat products	Pork, chop, boneless	FA0124	4	25cm plate	56	112	168	222
39	Meat and meat products	Pork, sliced	FA0123	4	25cm plate	27	54	81	108
40	Nuts and seeds	Cashew	FA0084	2	11.5cm bowl	75	150		
41	Nuts and seeds	Peanut	FA0105	4	17.5cm plate	15	30	**45***	**60***
42	Ready-to-eat savouries	Cuttlefish snack, stick	FA0010	2	25cm plate	8	16		
43	Ready-to-eat savouries	Popcorn	FA0002	2	11.5cm bowl	7	14		
44	Ready-to-eat savouries	Potato chip	FA0189	4	25cm plate	15	30	45	60
45	Vegetable protein	Mock meat, canned	FA0113	4	25cm plate	38	76	114	152
46	Vegetables (Brassica)	Broccoli, florets	FA0075	4	25cm plate	**41***	**83***	124	166
47	Vegetables (Brassica)	Cabbage, sliced	FA0130	2	25cm plate	38	77		
48	Vegetables (Fruiting vegetables)	Bittergourd, sliced	FA0129	2	25cm plate	28	55		

49	Vegetables (Fruiting vegetables)	Brinjal / Eggplant, sliced	FA0077	4	25cm plate	53	106	159	212
50	Vegetables (Fruiting vegetables)	Chilli / Capsicum, sliced	FA0076	4	25cm plate	11	23	34	46
51	Vegetables (Leafy vegetables, herbs)	Beansprout	FA0098	4	25cm plate	**44***	**88***	132	176
52	Vegetables (Leafy vegetables, herbs)	Chye sim	FA0128	4	25cm plate	46	92	138	184
53	Vegetables (Leafy vegetables, herbs)	Kailan	FA0139	4	25cm plate	53	**106***	159	**212***
54	Vegetables (Leafy vegetables, herbs)	Preserved vegetable, salted	FA0078	4	12.5cm plate	34	68	102	136
55	Vegetables (Leafy vegetables, herbs)	Spinach	FA0057	2	25cm plate	53	106		
56	Vegetables (Legumes)	Beancurd, dessert, traditional	FA0026	2	15cm bowl	205	410		
57	Vegetables (Legumes)	Egg tofu	FA0131	2	25cm plate	34	68		
58	Vegetables (Legumes)	Long bean	FA0127	2	25cm plate	29	58		
59	Vegetables (Legumes)	Tofu, firm (tau kwa)	FA0080	4	25cm plate	78	155	233	310
60	Vegetables (Legumes)	Tofu, silken / soft	FA0150	2	25cm plate	50	100		
61	Vegetables (Root and tubers)	Carrot, shredded	FA0132	2	25cm plate	7	13		
62	Vegetables (Root and tubers)	Fries, shoestring	FA0164	4	25cm plate	28	55	83	110
63	Vegetables (Root and tubers)	Fries, crinkle cut	FA0201	4	25cm plate	41	82	123	164
64	Vegetables (Root and tubers)	Fries, thick cut	FA0058	2	25cm plate	45	91		
65	Vegetables (Root and tubers)	Potato, mashed	FA0170	2	25cm plate	71	142		
66	Vegetables (Root and tubers)	Potato, sliced	FA0126	4	25cm plate	30	60	90	120
67	Vegetables (Stalk, stem and bulb)	Onion, sliced	FA0138	2	25cm plate	14	28		

Table 2. List of entries in guide

S/N	Category	Entry name	Photo ID	Primary tableware (pictured)	Edible portion weight (g)
1	Bakery products	Baguette, sliced, small piece	FA0038A	25cm plate	16
2	Bakery products	Baguette, sliced, big piece	FA0038B	25cm plate	22
3	Bakery products	Baguette, sliced, end piece	FA0038C	25cm plate	17

4	Bakery products	Bao, char siew, mini (叉烧包)	FA0039D	25cm plate	62
5	Bakery products	Bao, lotus, mini (莲蓉包)	FA0039C	25cm plate	76
6	Bakery products	Bao, meat, big (肉包)	FA0039A	25cm plate	166
7	Bakery products	Bao, red bean, mini (豆沙包)	FA0039B	25cm plate	68
8	Bakery products	Biscuit, cream cracker	FA0200C	25cm plate	8
9	Bakery products	Biscuit, round, chocolate chip, mini	FA0200B	25cm plate	4
10	Bakery products	Biscuit, round, fruit / nut	FA0200A	25cm plate	9
11	Bakery products	Biscuit, sandwiched with filling	FA0200D	25cm plate	10
12	Bakery products	Biscuit, filled, small	FA0145	25cm plate	13
13	Bakery products	Biscuit, iced gem	FA0144	25cm plate	7
14	Bakery products	Biscuit, square	FA0172	25cm plate	4
15	Bakery products	Biscuit, wafer	FA0143	25cm plate	14
16	Bakery products	Bread, brioche	FA0085	25cm plate	40
17	Bakery products	Bread, pita	FA0006	25cm plate	174
18	Bakery products	Bread, roll, long	FA0086	25cm plate	39
19	Bakery products	Bread, roll, long, multigrain	FA0136	25cm plate	67
20	Bakery products	Bread, roll, round	FA0087	25cm plate	33
21	Bakery products	Cake, banana	FA0035A	25cm plate	65
22	Bakery products	Cake, steamed	FA0035B	25cm plate	73
23	Bakery products	Cake, swiss roll, mini	FA0035D	25cm plate	29
24	Bakery products	Cake, swiss roll, regular	FA0035C	25cm plate	70
25	Bakery products	Cake, chiffon	FA0003	25cm plate	39
26	Bakery products	Cake, chocolate and fruit filling	FA0037A	25cm plate	125
27	Bakery products	Cake, tiramisu	FA0037B	25cm plate	66
28	Bakery products	Cake, cream	FA0036B	25cm plate	50
29	Bakery products	Cake, cream, fruit	FA0036A	25cm plate	113

30	Bakery products	Chwee kueh (水粿)	FA0032	25cm plate	42
31	Bakery products	Egg tart, mini	FA0027A	25cm plate	36
32	Bakery products	Egg tart, portuguese	FA0027C	25cm plate	50
33	Bakery products	Egg tart, regular	FA0027B	25cm plate	70
34	Bakery products	Kueh, ang ku	FA0023A	25cm plate	55
35	Bakery products	Kueh, rainbow (九层糕)	FA0023D	25cm plate	75
36	Bakery products	Kueh, salat	FA0023B	25cm plate	67
37	Bakery products	Kueh, tapioca	FA0023C	25cm plate	60
38	Bakery products	Kueh, kosui	FA0025	Commercial packaging	173
39	Bakery products	Kueh, onde onde	FA0024	Commercial packaging	117
40	Bakery products	Mooncake, mini, no egg, ¼ slice	FA0159B	25cm plate	8
41	Bakery products	Mooncake, mini, no egg, ½ slice	FA0159A	25cm plate	16
42	Bakery products	Mooncake, regular, no egg, ⅛ slice	FA0146C	25cm plate	25
43	Bakery products	Mooncake, regular, no egg, ¼ slice	FA0146B	25cm plate	50
44	Bakery products	Mooncake, regular, no egg, ½ slice	FA0146A	25cm plate	98
45	Bakery products	Mooncake, snow skin, no egg, ⅛ slice	FA0122A	25cm plate	24
46	Bakery products	Mooncake, snow skin, no egg, ¼ slice	FA0122C	25cm plate	48
47	Bakery products	Mooncake, snow skin, no egg, ½ slice	FA0122B	25cm plate	96
48	Bakery products	Pancake, regular	FA0043	25cm plate	47
49	Bakery products	Pretzel, regular	FA0034	25cm plate	100
50	Bakery products	Pretzel, stick	FA0008	25cm plate	36
51	Bakery products	Sesame ball, bean	FA0015B	25cm plate	61
52	Bakery products	Sesame ball, peanut	FA0015A	25cm plate	61
53	Bakery products	You tiao (油条)	FA0014	25cm plate	78

54	Beverages	Barley drink, glass cup	FA0099B	220ml cup	220
55	Beverages	Barley drink, melamine cup	FA0099A	270ml cup	220
56	Beverages	Blended shake, regular	FA0097A	Commercial packaging	431
57	Beverages	Blended shake, large	FA0097B	Commercial packaging	587
58	Beverages	Coffee / Kopi, regular	FA0040B	Commercial packaging	219
59	Beverages	Coffee / Kopi, large	FA0040A	Commercial packaging	321
60	Composite foods	Dim sum, beancurd roll (腐皮卷)	FA0028D	25cm plate	50
61	Composite foods	Dim sum, char siew pastry (叉烧酥)	FA0028C	25cm plate	44
62	Composite foods	Dim sum, fried prawn dumpling (明虾角)	FA0028A	25cm plate	47
63	Composite foods	Dim sum, yam puff (芋角)	FA0028B	25cm plate	53
64	Composite foods	Dim sum, carrot cake (萝卜糕)	FA0030	25cm plate	124
65	Composite foods	Dim sum, lor mai gai (糯米鸡)	FA0064	12.5cm plate	213
66	Composite foods	Dim sum, meat roll, ngoh hiang (五香卷)	FA0031	25cm plate	69
67	Composite foods	Dim sum, siew mai (烧卖)	FA0072	25cm plate	44
68	Composite foods	Gravy, braised, thick, bowl	FA0090B	11.5cm bowl	135
69	Composite foods	Gravy, braised, thick, soup spoon	FA0090A	Soup spoon	18
70	Composite foods	Gravy, curry, thick	FA0082	11.5cm bowl	112
71	Composite foods	Kimchi, plate	FA0013A	12.5cm plate	62
72	Composite foods	Kimchi, soup spoon	FA0013B	Soup spoon	22
73	Composite foods	Meat gyoza	FA0161	25cm plate	22
74	Composite foods	Pie, chicken	FA0029	25cm plate	110
75	Composite foods	Sesame paste, bowl	FA0021A	11.5cm bowl	142
76	Composite foods	Sesame paste, soup spoon	FA0021B	Soup spoon	15

77	Composite foods	Spring roll, fried	FA0016	25cm plate	64
78	Composite foods	Sushi, salmon	FA0178	25cm plate	26
79	Composite foods	Yam paste (orh nee), soup spoon	FA0020B	Soup spoon	24
80	Composite foods	Yam paste (orh nee), tub	FA0020A	Commercial packaging	191
81	Confectionery	Chocolate, block, 1 piece	FA0060D	25cm plate	6
82	Confectionery	Chocolate, block, 2 piece	FA0060C	25cm plate	11
83	Confectionery	Chocolate, block, 4 piece	FA0060B	25cm plate	22
84	Confectionery	Chocolate, block, 16 piece	FA0060A	25cm plate	90
85	Confectionery	Palm sugar (gula melaka), block	FA0188A	25cm plate	65
86	Confectionery	Palm sugar (gula melaka), pieces	FA0188B	12.5cm plate	17
87	Confectionery	Pudding, mango	FA0019	12.5cm plate	143
88	Egg and egg products	Egg, hard boiled	FA0069	25cm plate	54
89	Fish and seafood	Abalone, baby	FA0088	25cm plate	11
90	Fish and seafood	Abalone, sliced	FA0162	25cm plate	4
91	Fish and seafood	Crabstick, whole	FA0148	25cm plate	19
92	Fish and seafood	Fish cake, sliced	FA0068	25cm plate	20
93	Fish and seafood	Fish, battered, boneless, small piece	FA0102A	25cm plate	13
94	Fish and seafood	Fish, battered, boneless, medium piece	FA0102B	25cm plate	16
95	Fish and seafood	Fish, battered, boneless, large piece	FA0102C	25cm plate	25
96	Fish and seafood	Fish, grilled, boneless	FA0168	25cm plate	138
97	Fish and seafood	Fish, ikan selar	FA0152	25cm plate	28
98	Fish and seafood	Fish, pomfret, fried	FA0154	25cm plate	103
99	Fish and seafood	Fish, raw (sashimi)	FA0177	25cm plate	12
100	Fish and seafood	Fish, saba	FA0153	25cm plate	82
101	Fish and seafood	Fish, salmon, fillet	FA0151	25cm plate	87

102	Fish and seafood	Fish, sardine, small	FA0111B	25cm plate	49
103	Fish and seafood	Fish, sardine, big	FA0111A	25cm plate	76
104	Fish and seafood	Fishball	FA0119	25cm plate	16
105	Fish and seafood	Mussel	FA0179	25cm plate	4
106	Fish and seafood	Otah, banana leaf	FA0063A	25cm plate	50
107	Fish and seafood	Otah, regular, non-spicy	FA0063C	25cm plate	20
108	Fish and seafood	Otah, regular, spicy	FA0063B	25cm plate	23
109	Fish and seafood	Prawn, regular, deshelled	FA0135	25cm plate	7
110	Fish and seafood	Prawn, king, deshelled	FA0182B	25cm plate	13
111	Fish and seafood	Prawn, jumbo king, deshelled	FA0182A	25cm plate	25
112	Fish and seafood	Scallop	FA0163	25cm plate	6
113	Fruit and fruit products	Apple, sliced, thin piece	FA0046B	25cm plate	26
114	Fruit and fruit products	Apple, sliced, thick piece	FA0046A	25cm plate	34
115	Fruit and fruit products	Banana, ball, fried	FA0017B	25cm plate	31
116	Fruit and fruit products	Banana, fried (goreng pisang)	FA0017A	25cm plate	92
117	Fruit and fruit products	Banana, regular	FA0174	25cm plate	106
118	Fruit and fruit products	Banana, small	FA0204	25cm plate	37
119	Fruit and fruit products	Dragon fruit, white flesh, ½ wedge	FA0050B	25cm plate	30
120	Fruit and fruit products	Dragon fruit, white flesh, wedge	FA0050A	25cm plate	58
121	Fruit and fruit products	Grape, bunch	FA0005B	25cm plate	25
122	Fruit and fruit products	Grape, loose	FA0005A	15cm bowl	194
123	Fruit and fruit products	Kiwi, sliced, thin piece	FA0049A	25cm plate	17
124	Fruit and fruit products	Kiwi, sliced, middle piece	FA0049C	25cm plate	42
125	Fruit and fruit products	Kiwi, sliced, end piece	FA0049B	25cm plate	34
126	Fruit and fruit products	Mango, dried	FA0009	12.5cm plate	34
127	Fruit and fruit products	Mango, sliced, thin piece	FA0052B	25cm plate	20

128	Fruit and fruit products	Mango, sliced, middle piece	FA0052C	25cm plate	39
129	Fruit and fruit products	Mango, sliced, end piece	FA0052A	25cm plate	20
130	Fruit and fruit products	Orange, regular, sliced, middle piece	FA0048A	25cm plate	43
131	Fruit and fruit products	Orange, regular, sliced, end piece	FA0048C	25cm plate	36
132	Fruit and fruit products	Orange, regular, wedge	FA0048B	25cm plate	17
133	Fruit and fruit products	Orange, small (tangerine)	FA0176	25cm plate	54
134	Fruit and fruit products	Pear, golden, thin wedge	FA0051B	25cm plate	25
135	Fruit and fruit products	Pear, golden, thick wedge	FA0051A	25cm plate	52
136	Fruit and fruit products	Pear, green, wedge	FA0175	25cm plate	32
137	Fruit and fruit products	Pineapple, block	FA0047A	25cm plate	70
138	Fruit and fruit products	Pineapple, sliced, small piece	FA0047B	25cm plate	10
139	Fruit and fruit products	Pineapple, sliced, big piece	FA0047C	25cm plate	36
140	Fruit and fruit products	Pomelo, shredded	FA0053A	Soup spoon	17
141	Fruit and fruit products	Pomelo, ½ wedge	FA0053C	25cm plate	36
142	Fruit and fruit products	Pomelo, wedge	FA0053B	25cm plate	82
143	Fruit and fruit products	Watermelon, wedge	FA0186	25cm plate	65
144	Fungi and seaweed	Fungus, black	FA0196	25cm plate	27
145	Fungi and seaweed	Mushroom, button, canned	FA0109	25cm plate	51
146	Fungi and seaweed	Mushroom, shiitake	FA0089	25cm plate	9
147	Grain and grain-based products	Rice dumpling, mini, nonya	FA0202	25cm plate	124
148	Grain and grain-based products	Rice dumpling, regular, nonya	FA0100	25cm plate	232
149	Grain and grain-based products	Rice dumpling, regular, hokkien	FA0203	25cm plate	246
150	Grain and grain products	Rice noodle, bee hoon, thin, cooked	FA0185	11.5cm bowl	160
151	Grain and grain products	Rice noodle, kway teow, cooked	FA0117	15cm bowl	120
152	Meat and meat products	Bacon	FA0137	25cm plate	7

153	Meat and meat products	Chicken, breast, fillet, boneless	FA0134	25cm plate	70
154	Meat and meat products	Chicken, breast, whole	FA0183	25cm plate	220
155	Meat and meat products	Chicken, chop, boneless	FA0165	25cm plate	150
156	Meat and meat products	Chicken, drumstick, regular	FA0166	25cm plate	57
157	Meat and meat products	Chicken, drumstick, small	FA0158	25cm plate	24
158	Meat and meat products	Chicken, leg, whole	FA0157	25cm plate	155
159	Meat and meat products	Chicken, thigh, fillet, boneless	FA0045	25cm plate	98
160	Meat and meat products	Chicken, mid-joint wing	FA0070	25cm plate	10
161	Meat and meat products	Chicken, wing	FA0167	25cm plate	55
162	Meat and meat products	Chicken, spring	FA0156	25cm plate	384
163	Meat and meat products	Luncheon meat	FA0055	25cm plate	36
164	Meat and meat products	Meat floss, teaspoon	FA0018A	25cm plate	1
165	Meat and meat products	Meat floss, tablespoon	FA0018C	25cm plate	2.3
166	Meat and meat products	Meat floss, soup spoon	FA0018B	25cm plate	4.5
167	Meat and meat products	Meatball, handmade	FA0096	25cm plate	54
168	Meat and meat products	Meatball, processed	FA0118	25cm plate	10
169	Meat and meat products	Pork belly, small piece	FA0125B	25cm plate	15
170	Meat and meat products	Pork belly, big piece	FA0125A	25cm plate	35
171	Meat and meat products	Pork rib, with bone	FA0141	25cm plate	50
172	Meat and meat products	Pork rib, with bone, short	FA0184	25cm plate	19
173	Meat and meat products	Sausage, bratwurst	FA0054C	25cm plate	43
174	Meat and meat products	Sausage, cocktail	FA0054D	25cm plate	15
175	Meat and meat products	Sausage, franks	FA0054B	25cm plate	28
176	Meat and meat products	Sausage, taiwan	FA0054A	25cm plate	51
177	Milk and dairy products	Butter, sliced, thin	FA0149C	25cm plate	3
178	Milk and dairy products	Butter, sliced, medium	FA0149A	25cm plate	6
179	Milk and dairy products	Butter, sliced, thick	FA0149B	25cm plate	12

180	Milk and dairy products	Cheese, cream, soft, teaspoon	FA0062A	Teaspoon	6
181	Milk and dairy products	Cheese, cream, soft, tablespoon	FA0062B	Tablespoon	20
182	Milk and dairy products	Cheese, cream, soft, soup spoon	FA0062C	Soup spoon	26
183	Milk and dairy products	Cheese, mozzarella, shredded, teaspoon	FA0061A	Teaspoon	3
184	Milk and dairy products	Cheese, mozzarella, shredded, tablespoon	FA0061B	Tablespoon	6
185	Milk and dairy products	Cheese, mozzarella, shredded, soup spoon	FA0061C	Soup spoon	10
186	Milk and dairy products	Milk, condensed	FA0108	Soup spoon	20
187	Milk and dairy products	Yoghurt	FA0133	Commercial packaging	130
188	Ready-to-eat savouries	Cuttlefish snack, red, sugary	FA0011	12.5cm plate	24
189	Ready-to-eat savouries	Cuttlefish snack, shredded	FA0012	12.5cm plate	9
190	Sauces and condiments	Black pepper, seed, teaspoon	FA0041A	25cm plate	2
191	Sauces and condiments	Black pepper, seed, tablespoon	FA0041B	25cm plate	7
192	Sauces and condiments	Black pepper, seed, soup spoon	FA0041C	25cm plate	11
193	Sauces and condiments	Chilli, powder, teaspoon	FA0083A	25cm plate	2
194	Sauces and condiments	Chilli, powder, tablespoon	FA0083B	25cm plate	7
195	Sauces and condiments	Chilli, sambal, teaspoon	FA0199A	25cm plate	6
196	Sauces and condiments	Chilli, sambal, tablespoon	FA0199B	25cm plate	17
197	Sauces and condiments	Chilli, sambal, soup spoon	FA0199C	25cm plate	24
198	Sauces and condiments	Salt, teaspoon	FA0106A	25cm plate	5
199	Sauces and condiments	Salt, tablespoon	FA0106B	25cm plate	13
200	Sauces and condiments	Salt, soup spoon	FA0106C	25cm plate	24
201	Sauces and condiments	Sauce, black pepper	FA0171	Commercial packaging	63
202	Sauces and condiments	Sauce, chilli, small sachet	FA0142B	25cm plate	7
203	Sauces and condiments	Sauce, chilli, big sachet	FA0142A	25cm plate	13

204	Sauces and condiments	Sauce, cream-based	FA0107	Soup spoon	18
205	Sauces and condiments	Soy sauce, dark, soup spoon	FA0104A	Soup spoon	24
206	Sauces and condiments	Soy sauce, dark, saucer	FA0104B	9.5cm saucer	20
207	Sauces and condiments	Soy sauce, light, soup spoon	FA0103A	Soup spoon	21
208	Sauces and condiments	Soy sauce, light, saucer	FA0103B	9.5cm saucer	25
209	Vegetables (Fruiting vegetables)	Cucumber, sliced, ½	FA0116B	25cm plate	3
210	Vegetables (Fruiting vegetables)	Cucumber, sliced	FA0116A	25cm plate	5
211	Vegetables (Fruiting vegetables)	Cucumber, strip	FA0116C	25cm plate	4
212	Vegetables (Fruiting vegetables)	Tomato, sliced	FA0115A	25cm plate	13
213	Vegetables (Fruiting vegetables)	Tomato, ⅛ wedge	FA0115C	25cm plate	12
214	Vegetables (Fruiting vegetables)	Tomato, ½ wedge	FA0115B	25cm plate	47
215	Vegetables (Fruiting vegetables)	Tomato, cherry, small	FA0114C	25cm plate	5
216	Vegetables (Fruiting vegetables)	Tomato, cherry, medium	FA0114B	25cm plate	9
217	Vegetables (Fruiting vegetables)	Tomato, cherry, large	FA0114A	25cm plate	13
218	Vegetables (Leafy vegetables, herbs)	Basil, dried, teaspoon	FA0042A	25cm plate	0.6
219	Vegetables (Leafy vegetables, herbs)	Basil, dried, tablespoon	FA0042B	25cm plate	1.3
220	Vegetables (Leafy vegetables, herbs)	Basil, dried, soup spoon	FA0042C	25cm plate	2.9
221	Vegetables (Leafy vegetables, herbs)	Lettuce, leaf	FA0147	25cm plate	30
222	Vegetables (Legumes)	Beancurd puff (tau pok)	FA0073	25cm plate	25
223	Vegetables (Legumes)	Beancurd, dessert	FA0022	Commercial packaging	256
224	Vegetables (Root and tubers)	Hashbrown, mini (tater tot)	FA0044B	25cm plate	16
225	Vegetables (Root and tubers)	Hashbrown, oblong	FA0044A	25cm plate	52
226	Vegetables (Root and tubers)	Hashbrown, triangle	FA0044C	25cm plate	41
227	Vegetables (Root and tubers)	Potato, peeled, small piece	FA0093A	25cm plate	13
228	Vegetables (Root and tubers)	Potato, peeled, medium piece	FA0093B	25cm plate	19
229	Vegetables (Root and tubers)	Potato, peeled, large piece	FA0093C	25cm plate	26

230	Vegetables (Stalk, stem and bulb)	Ginger, sliced, small piece	FA0121A	25cm plate	0.5
231	Vegetables (Stalk, stem and bulb)	Ginger, sliced, large piece	FA0121B	25cm plate	1
