# Supplementary material for: Development and validation of a photographic food atlas for portion size estimation in Singapore
Source: Front Nutr. 2026 Sep 18;13:1930409. doi: 10.3389/fnut.2026.1930409 (PMC13630621; doi:10.3389/fnut.2026.1930409)

Biscuit, stick, plain (FA0187)

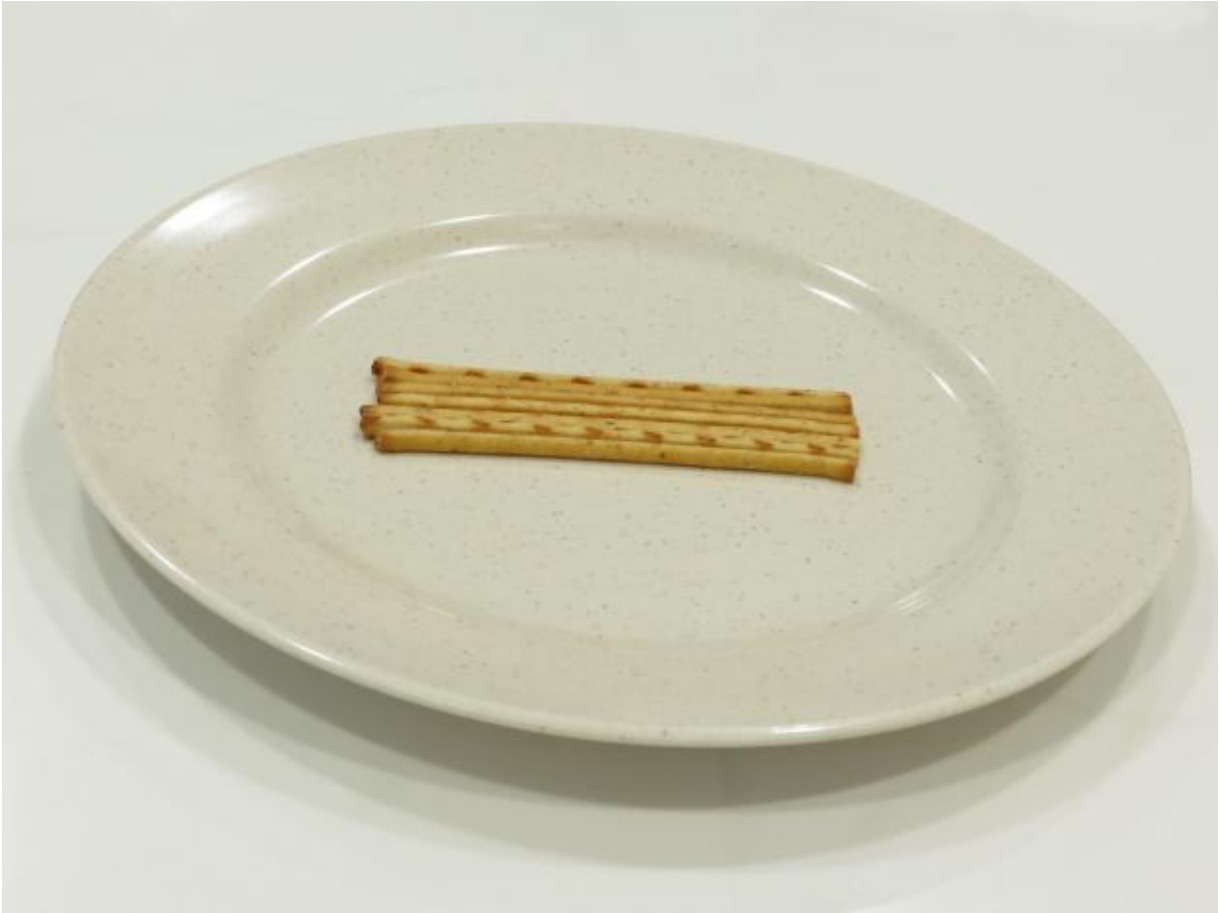
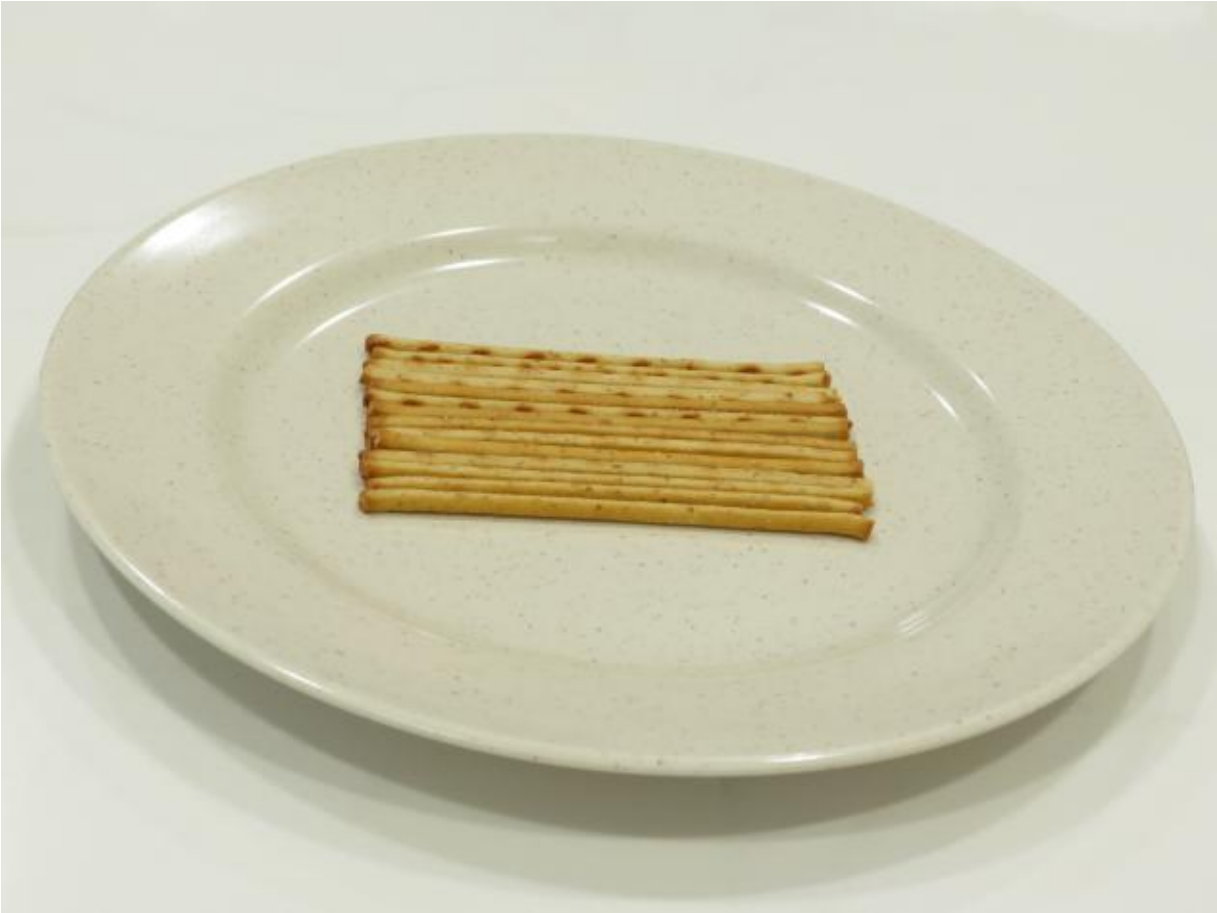
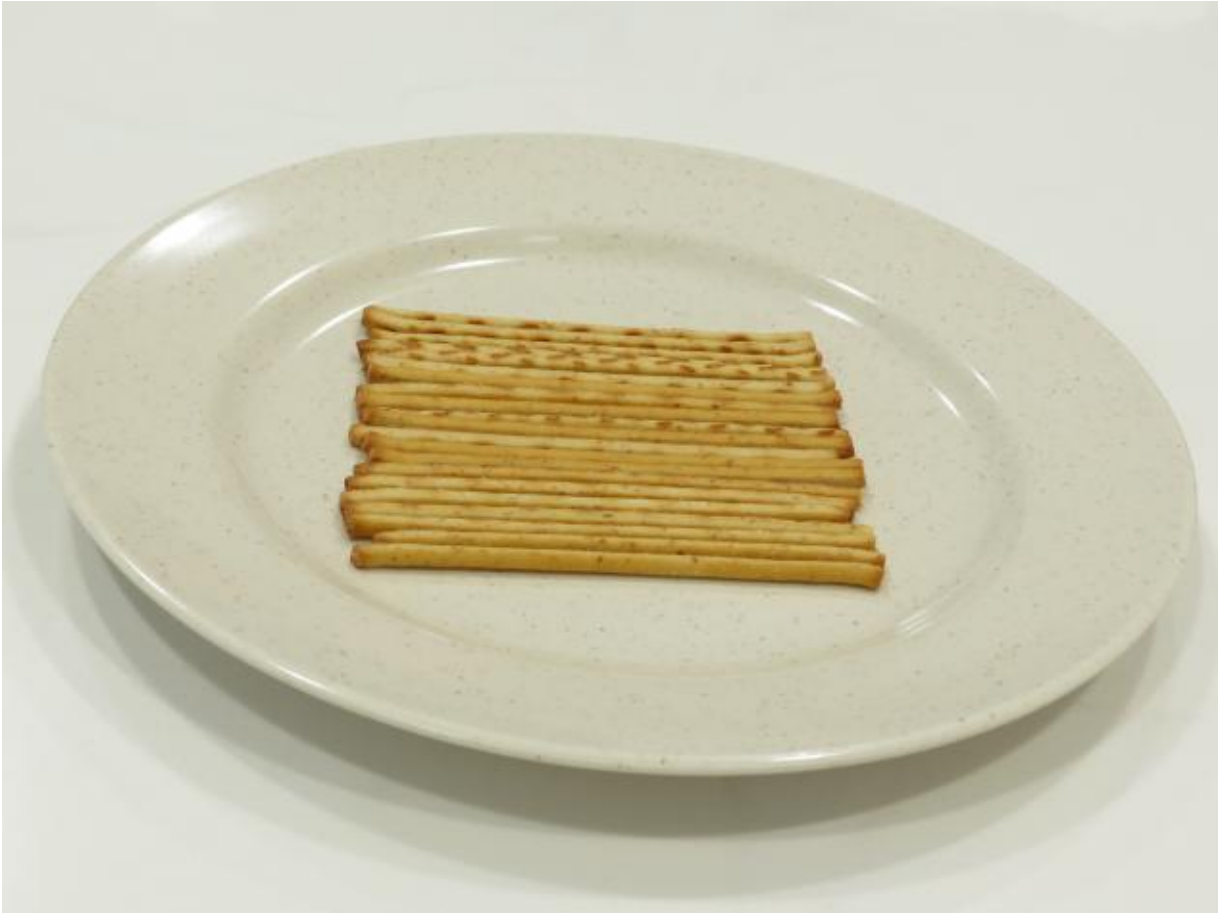
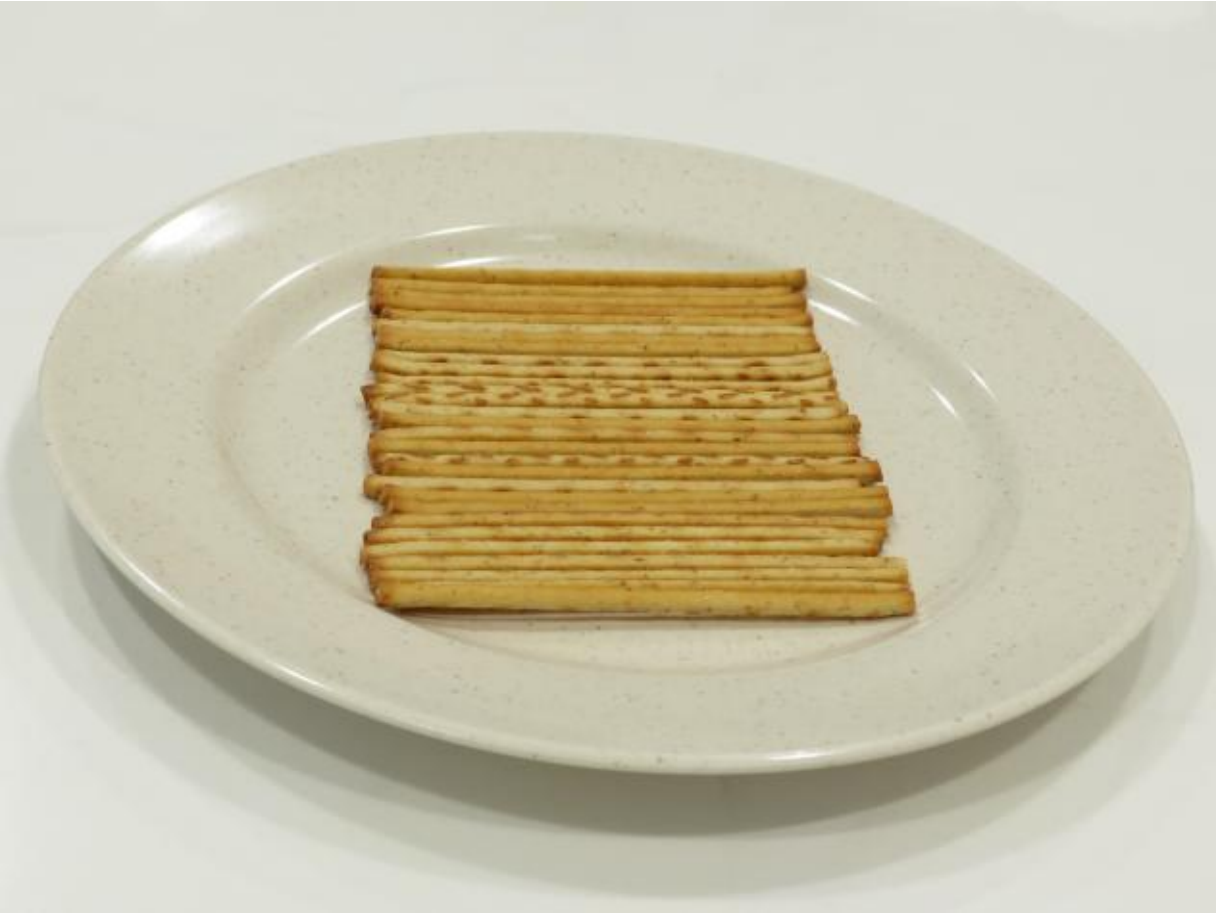

Biscuit, stick, with coating (FA0001)

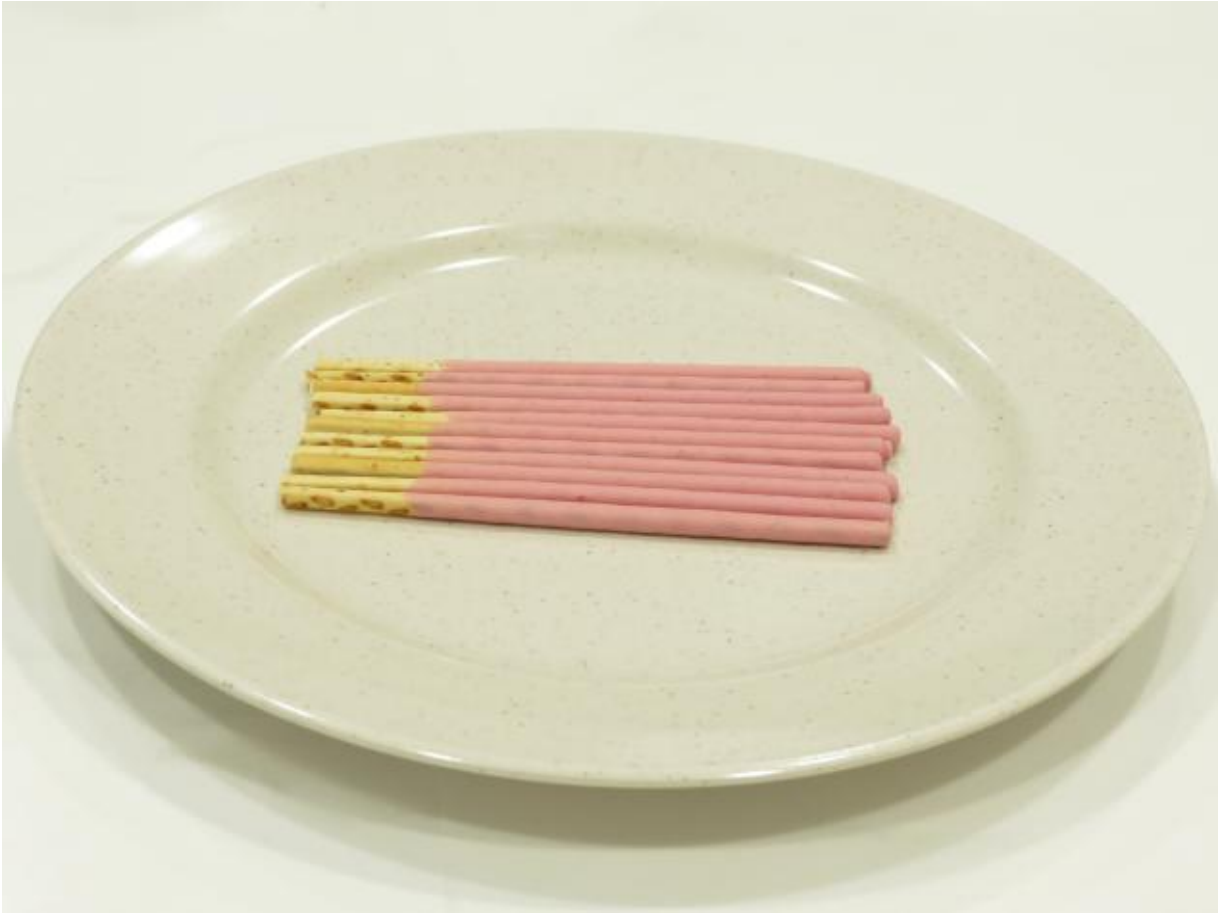
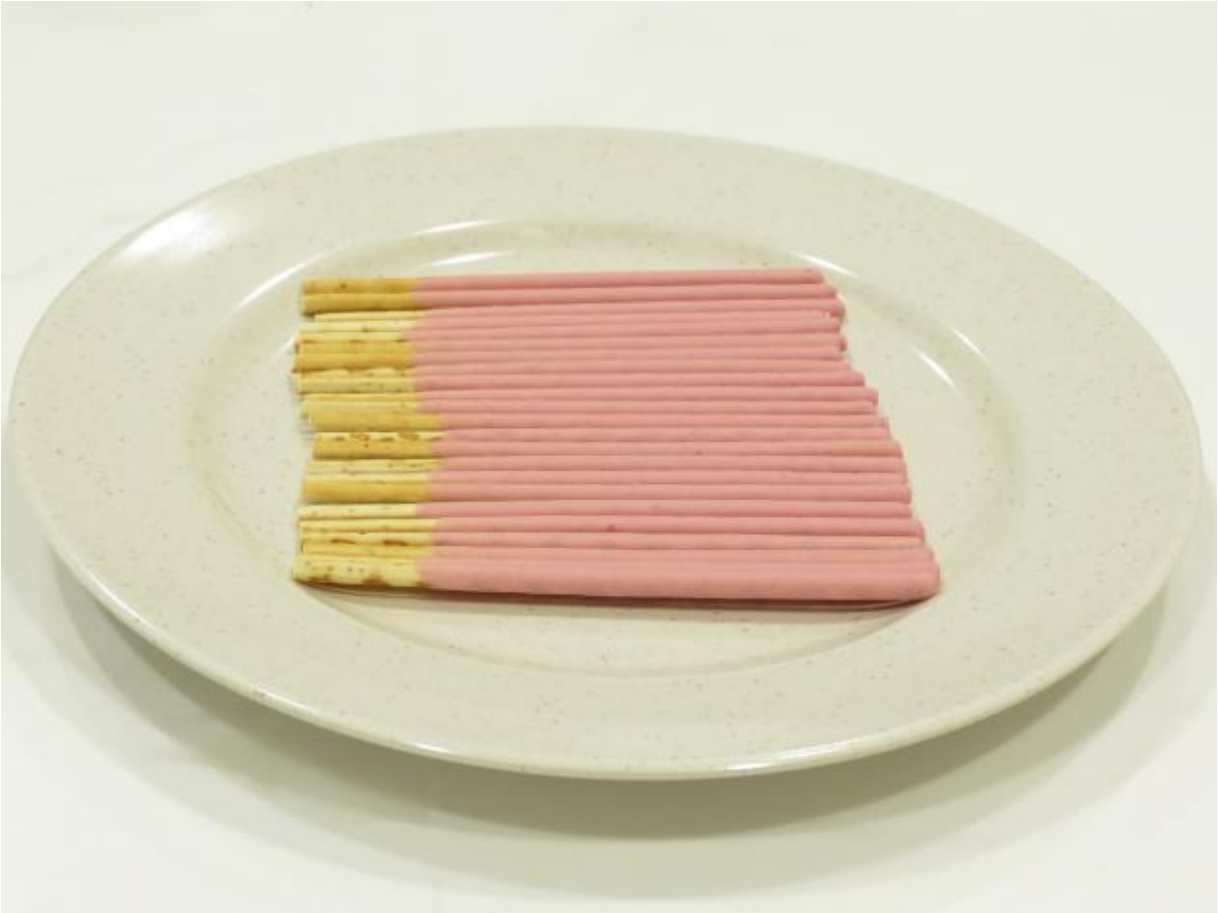
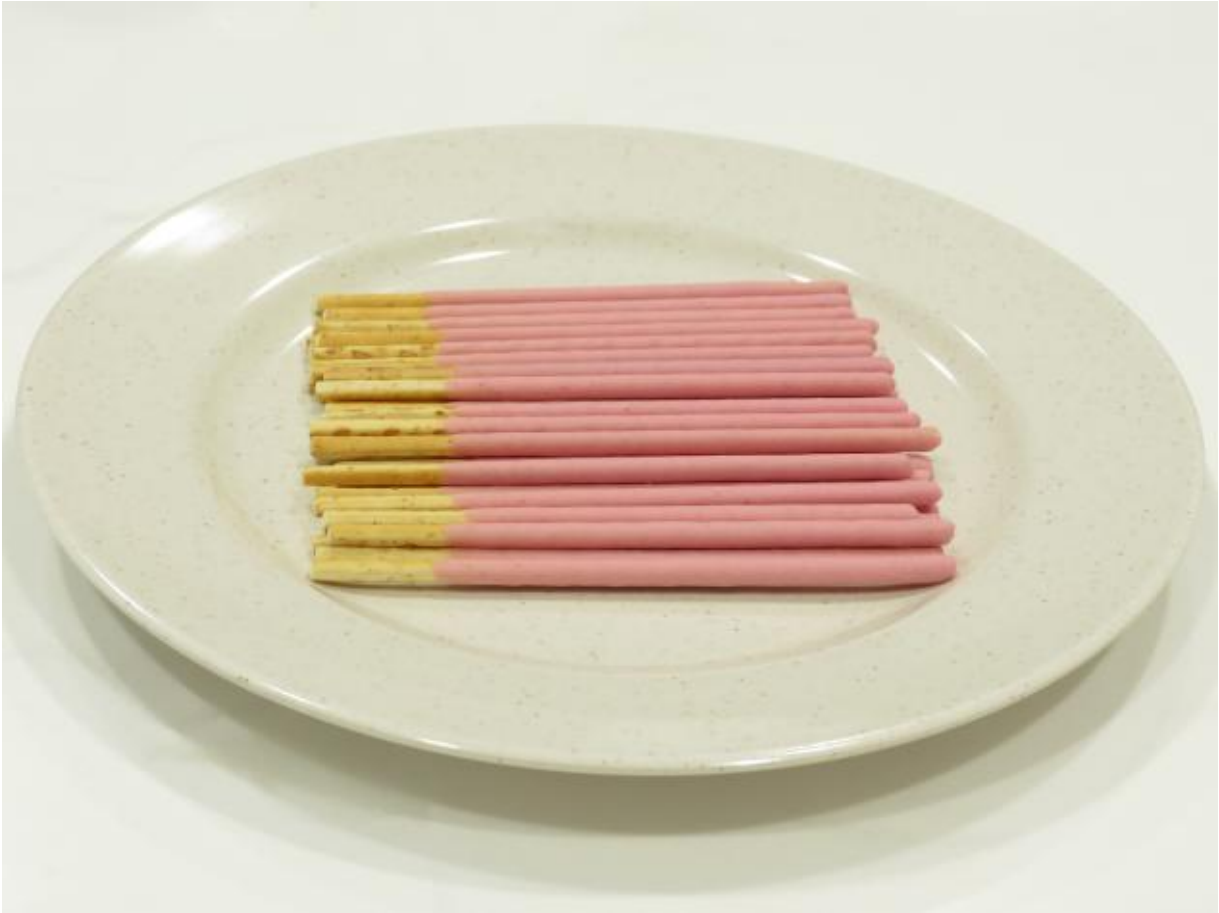
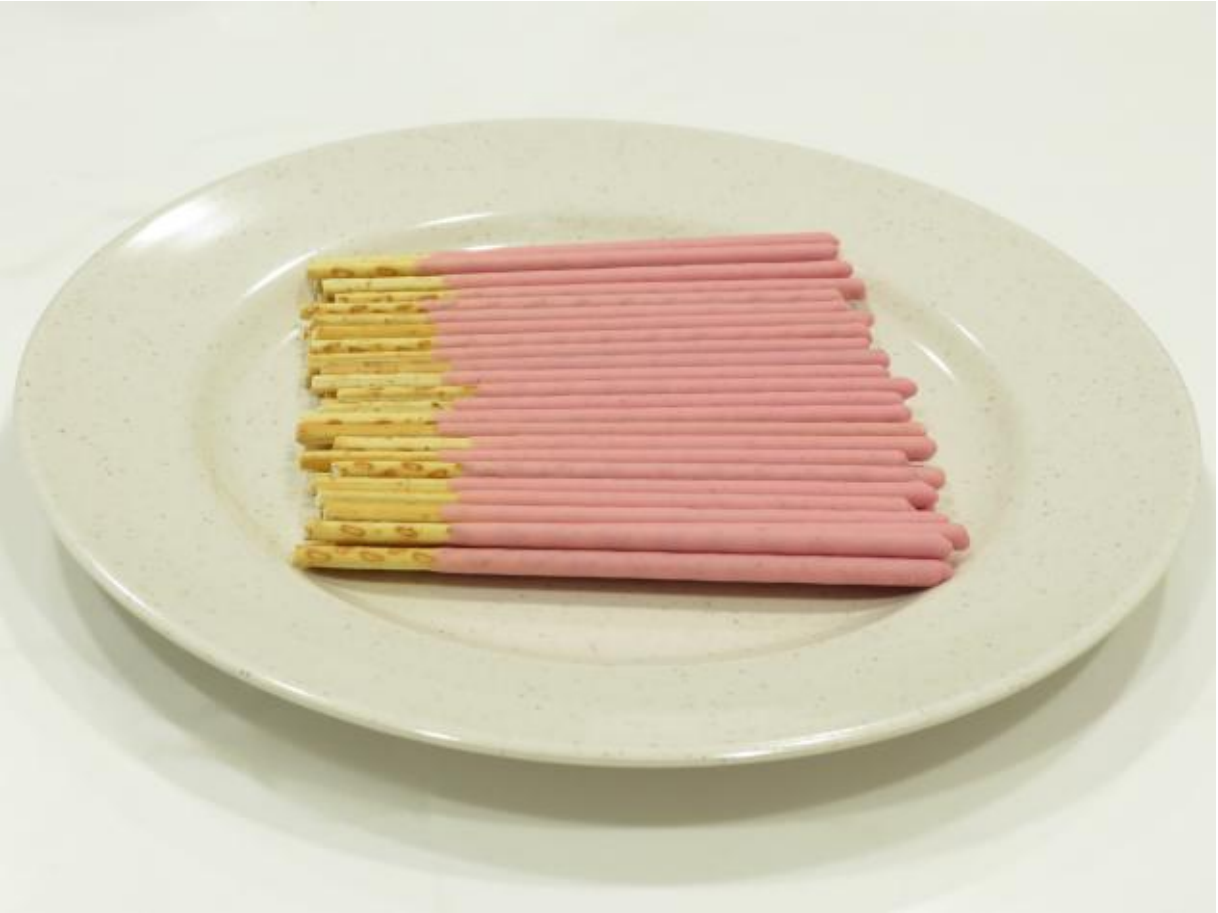

Soup, clear (FA0067)

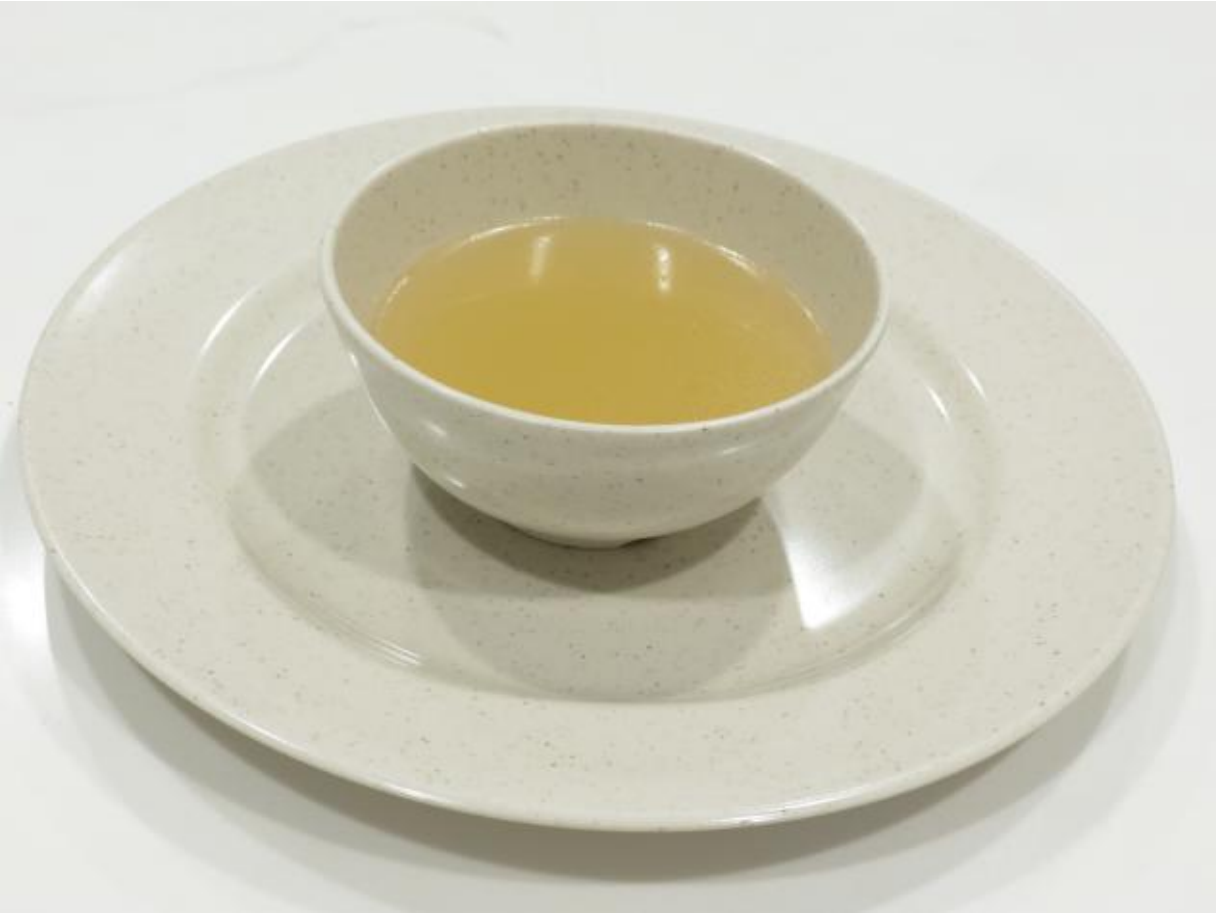

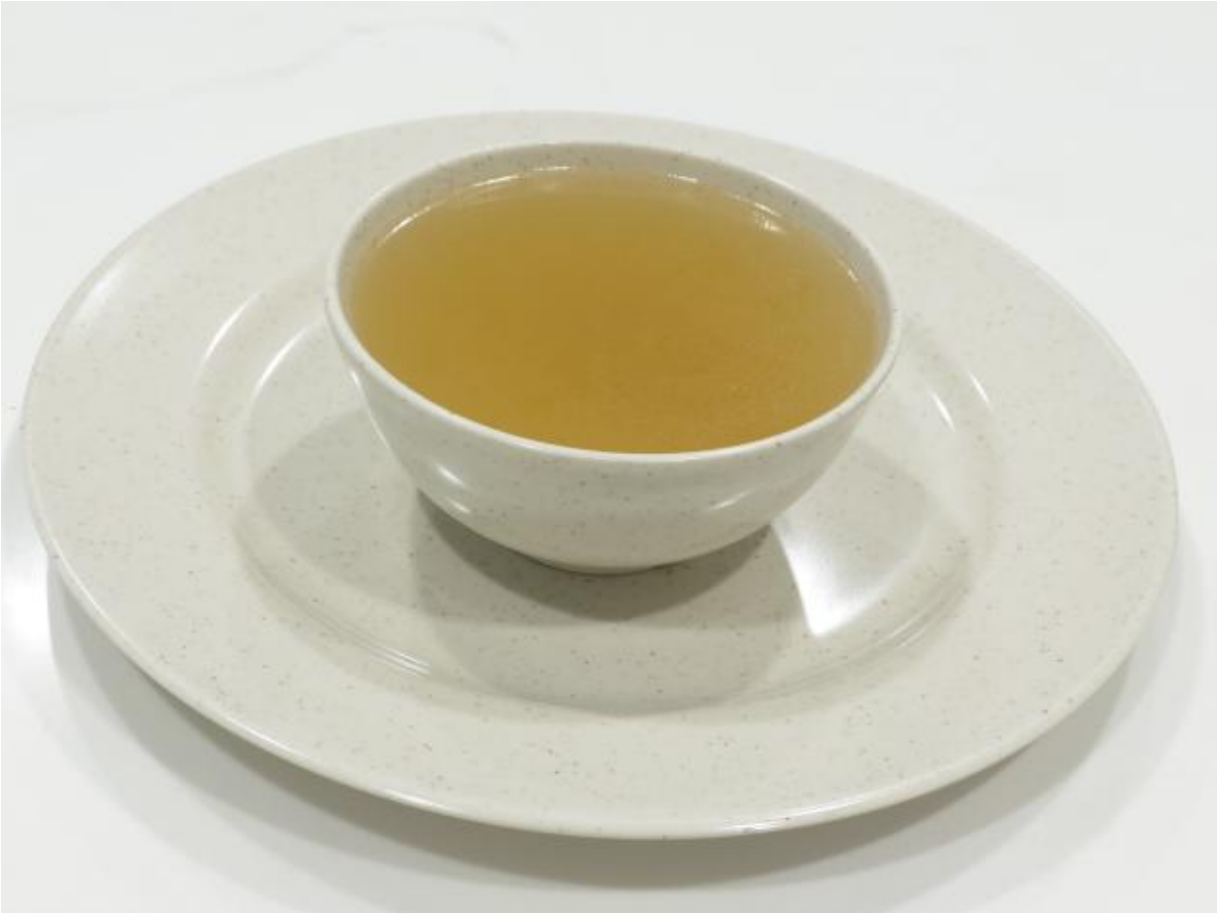

Soup, cream (FA0094)

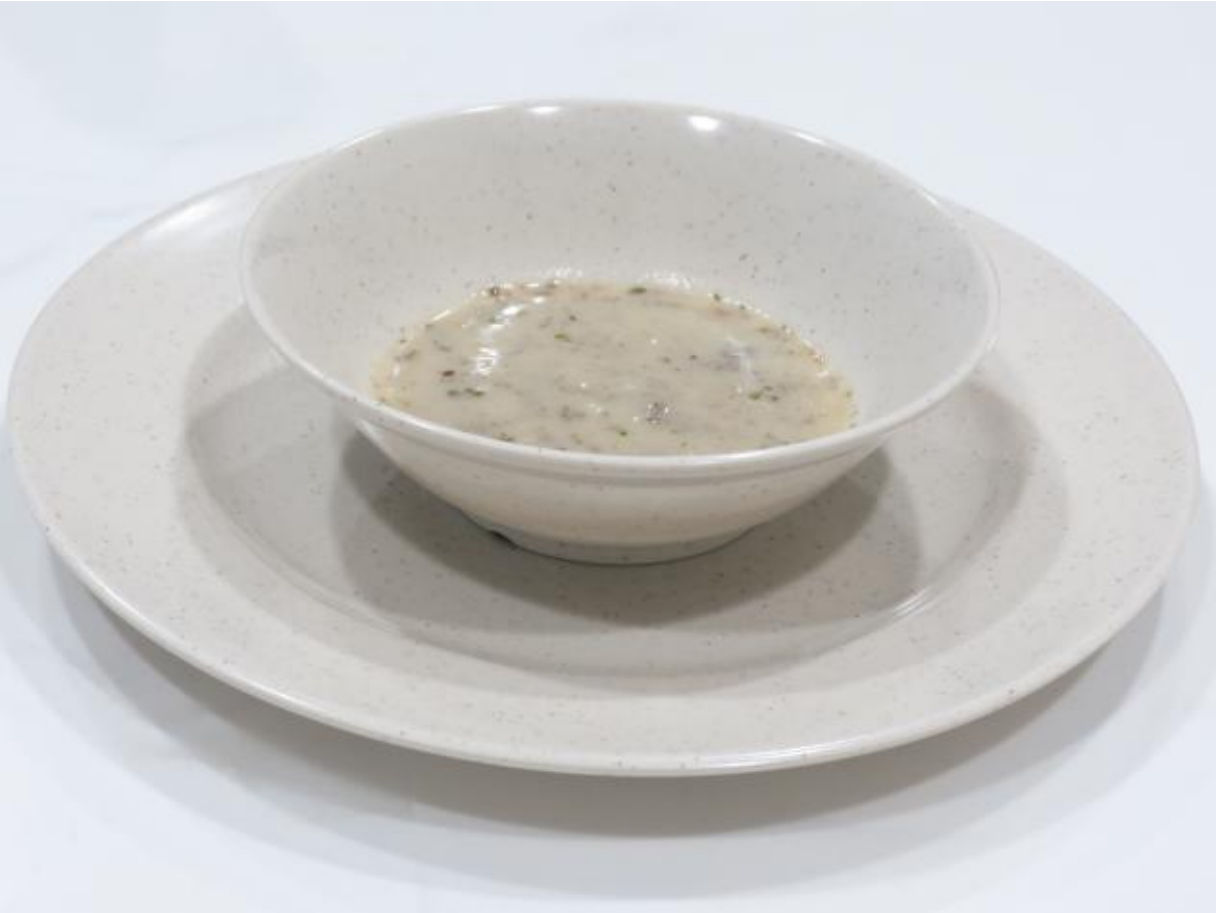
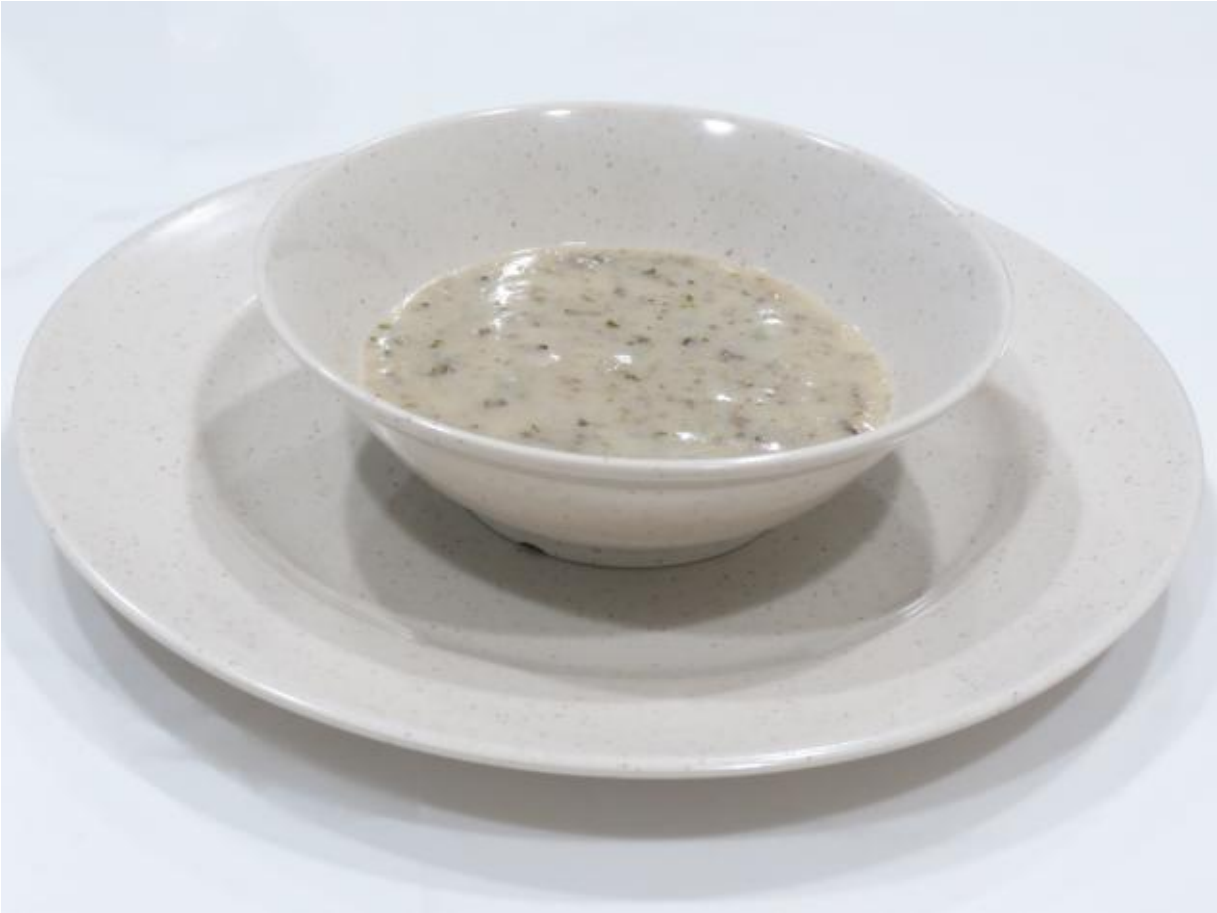
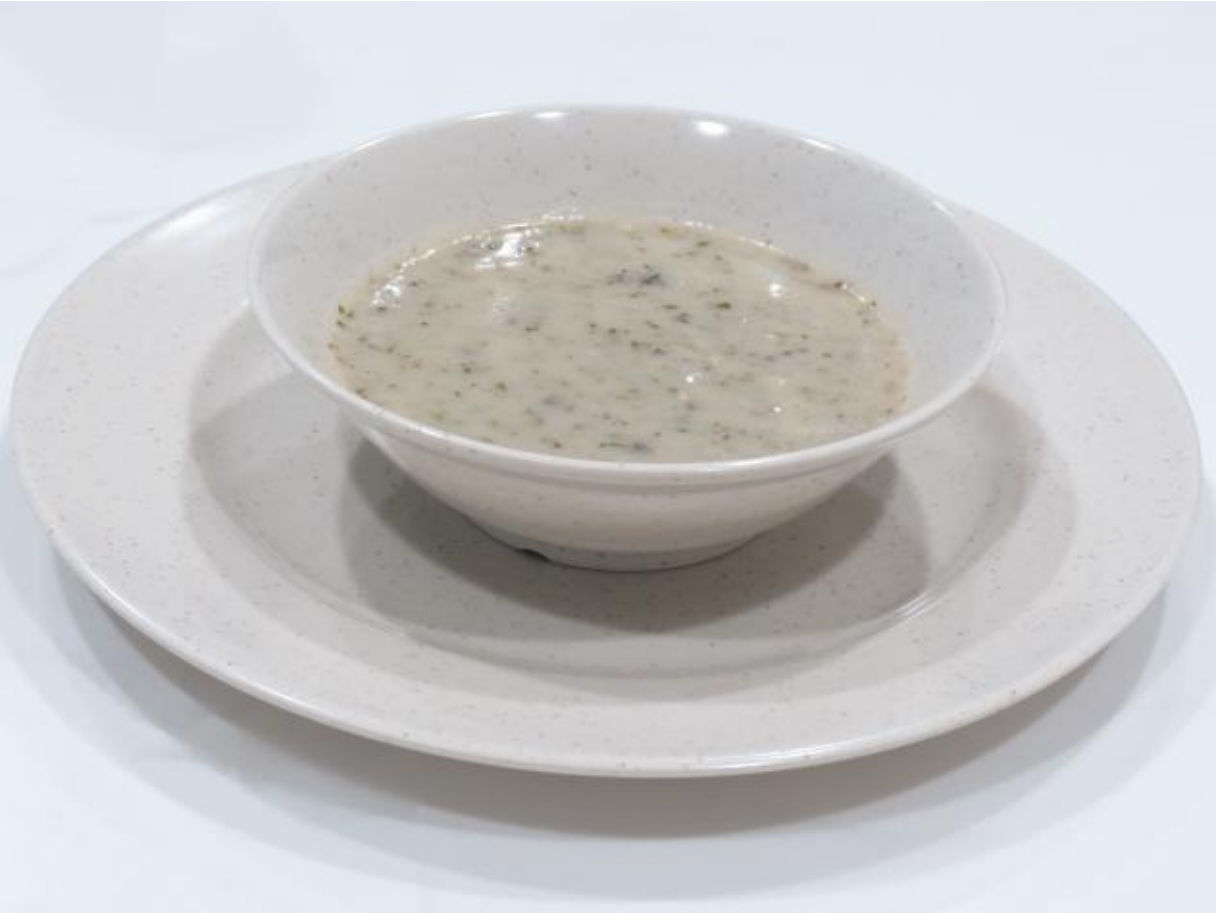
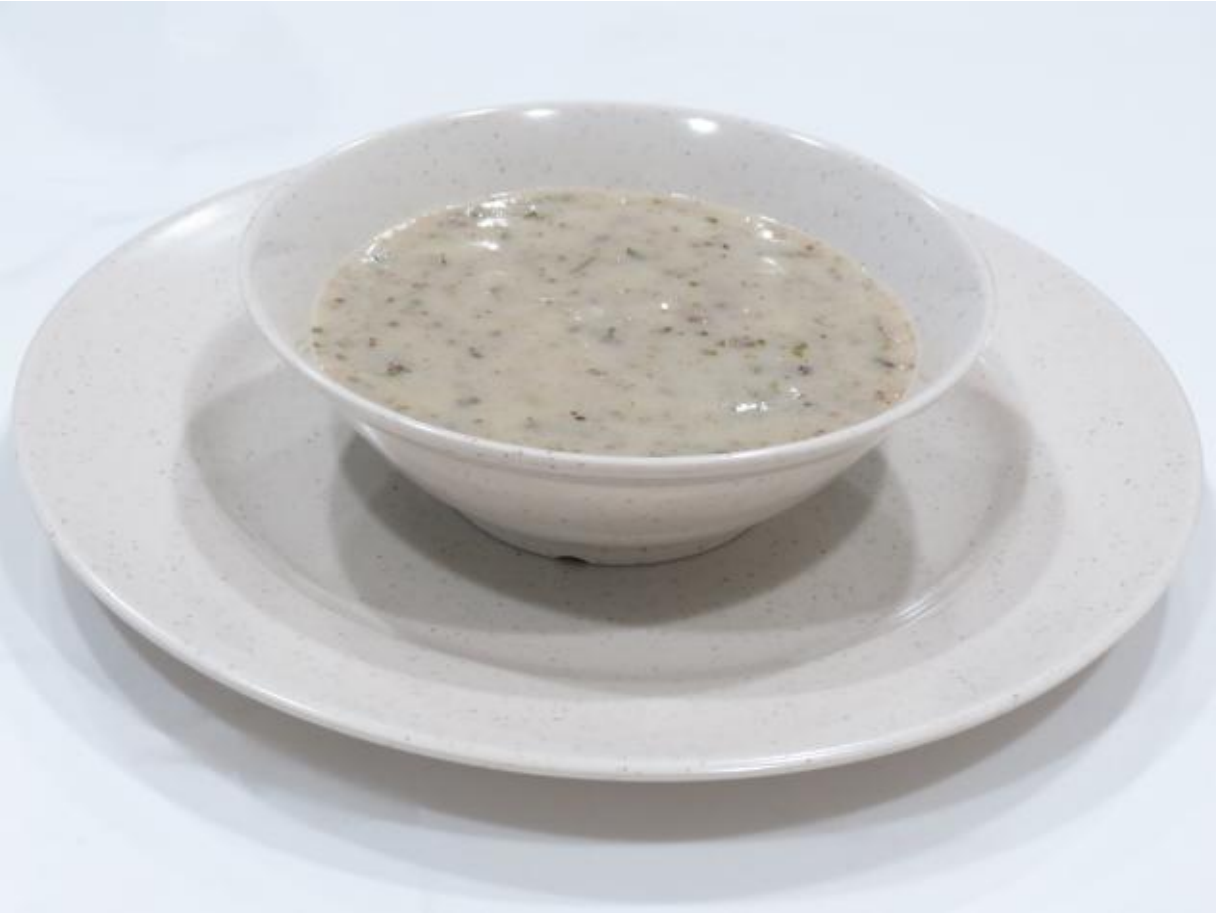

Chocolate candy (FA0198)

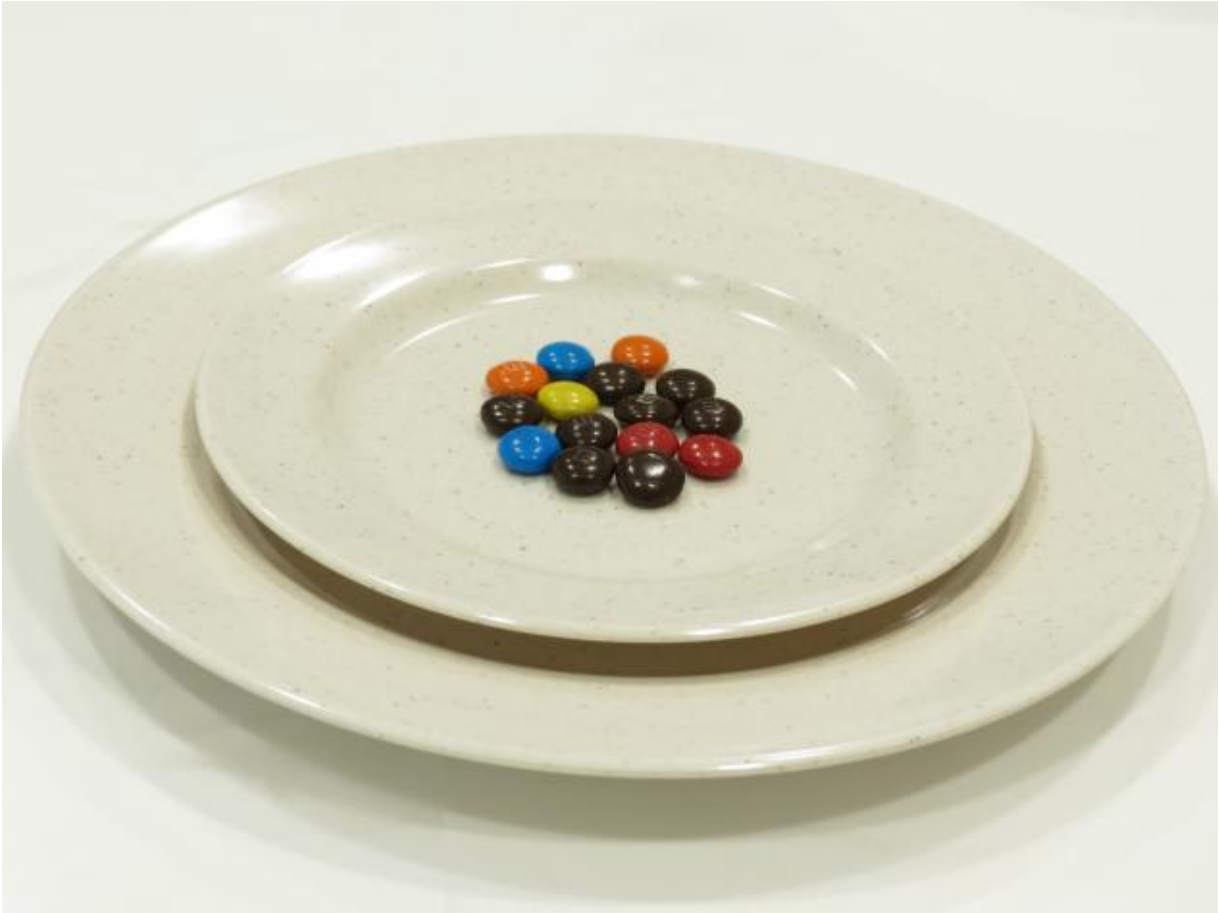

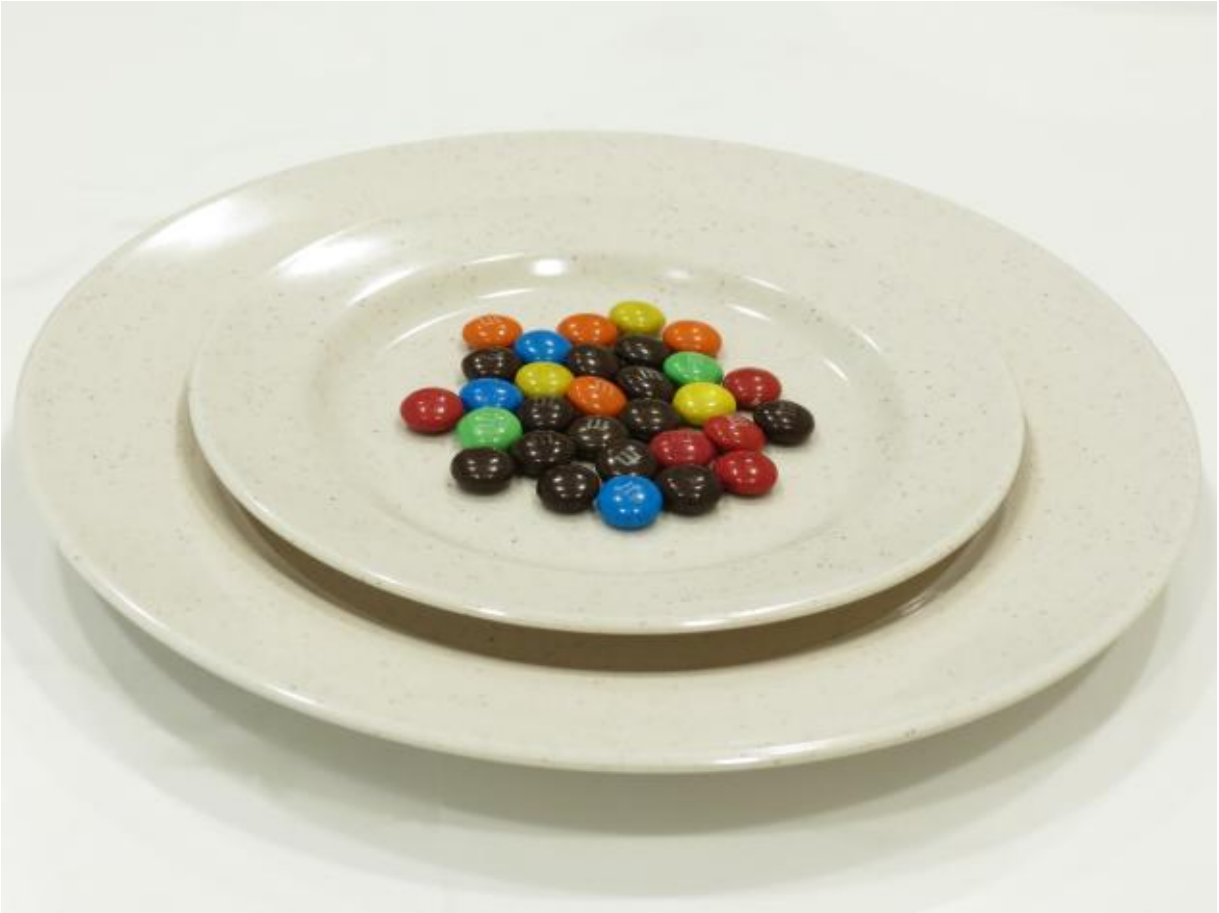

Tapioca pearl (FA0173)

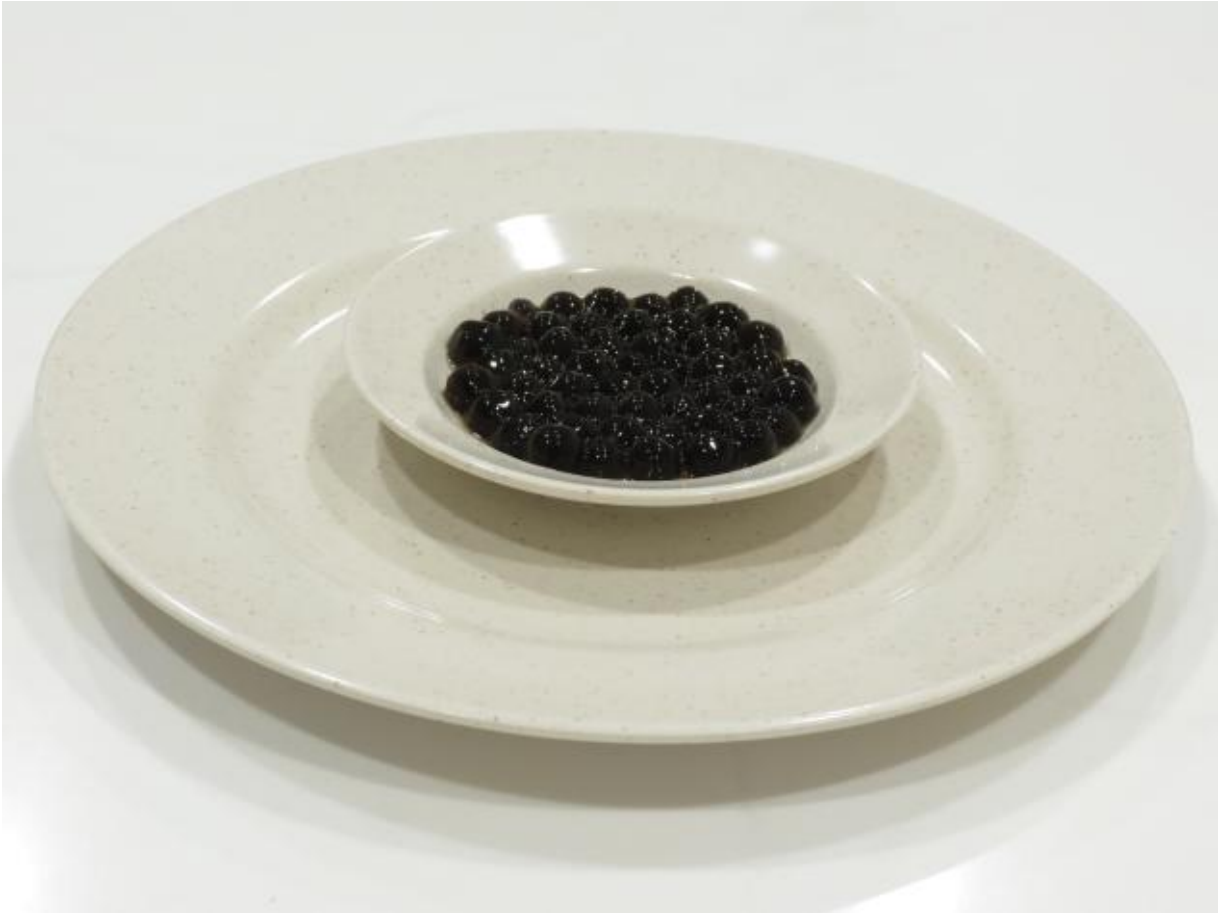

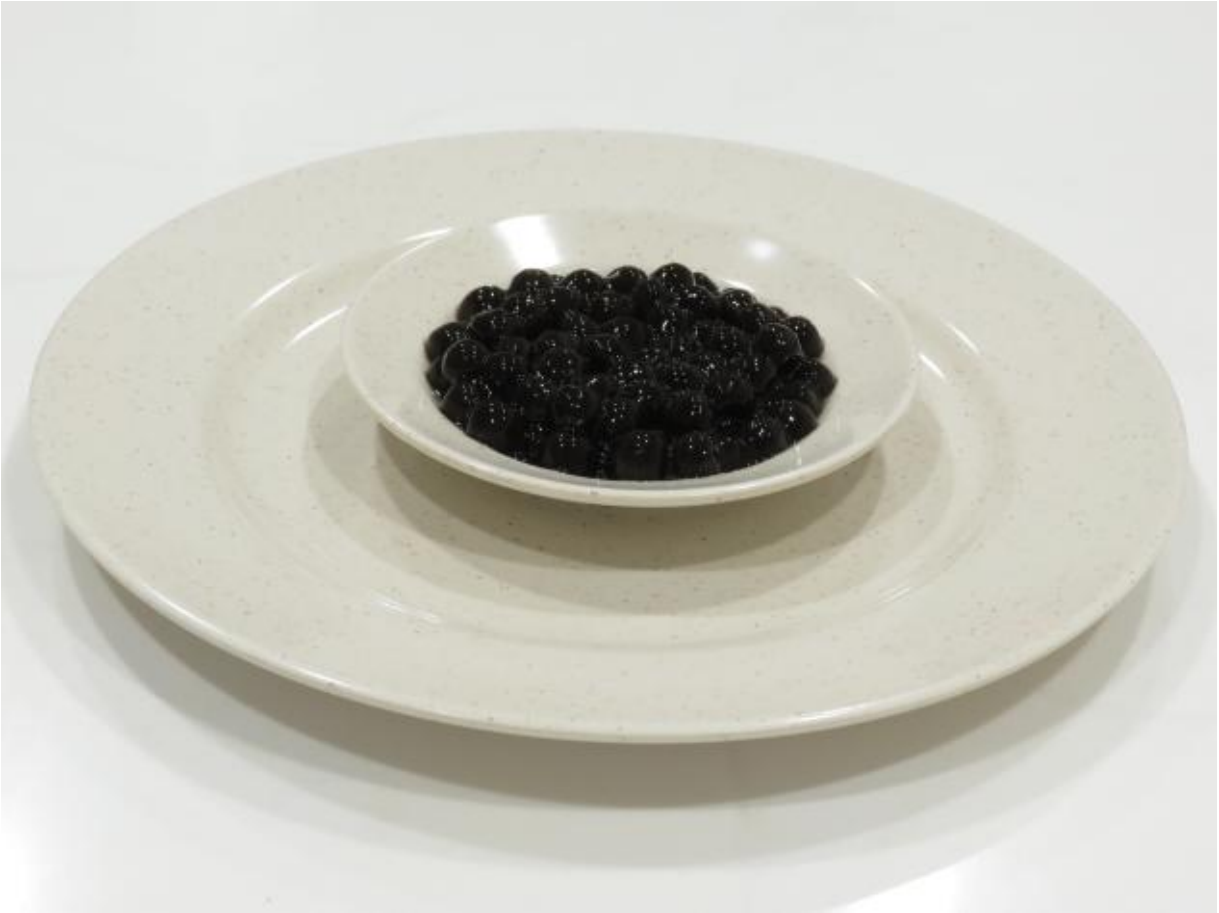

Egg, scrambled (FA0190)

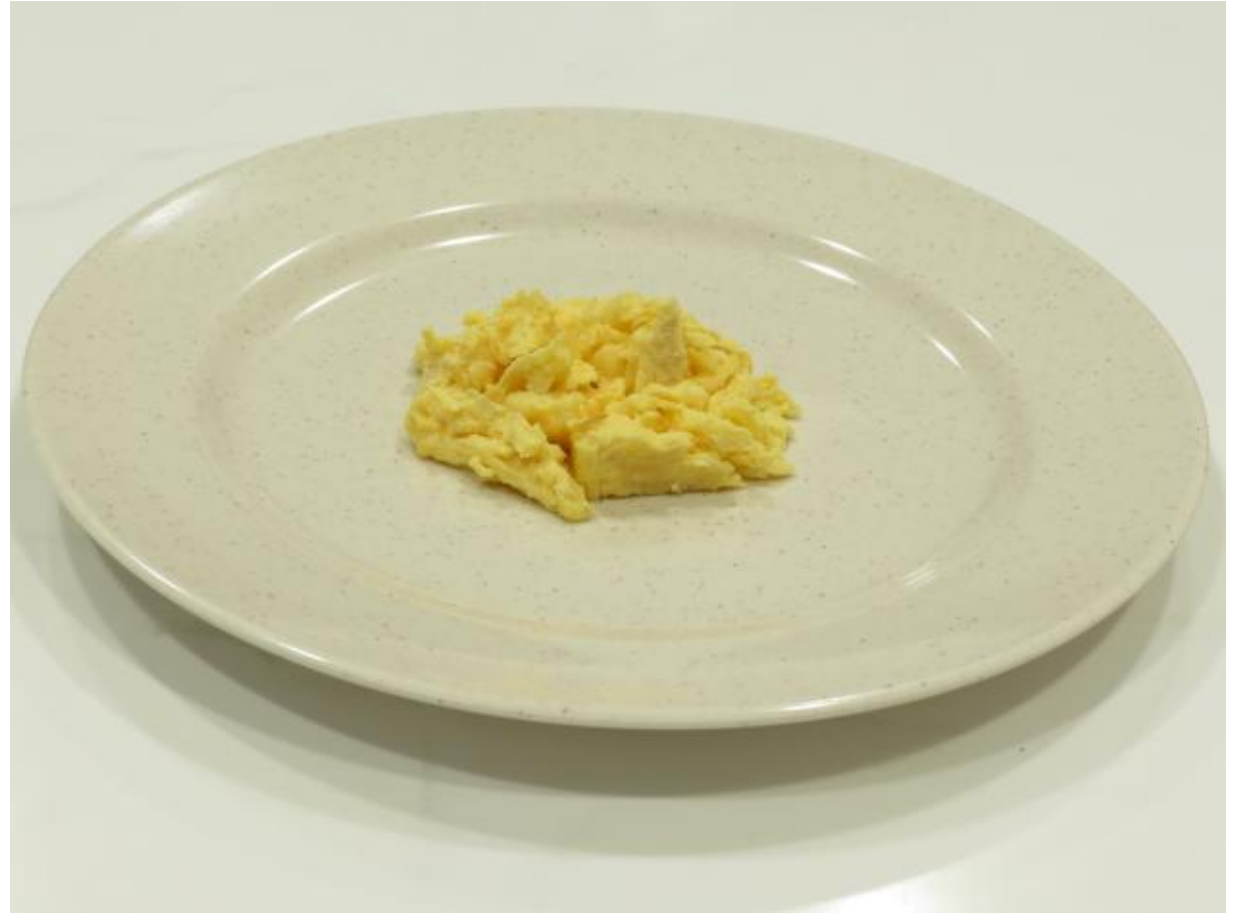

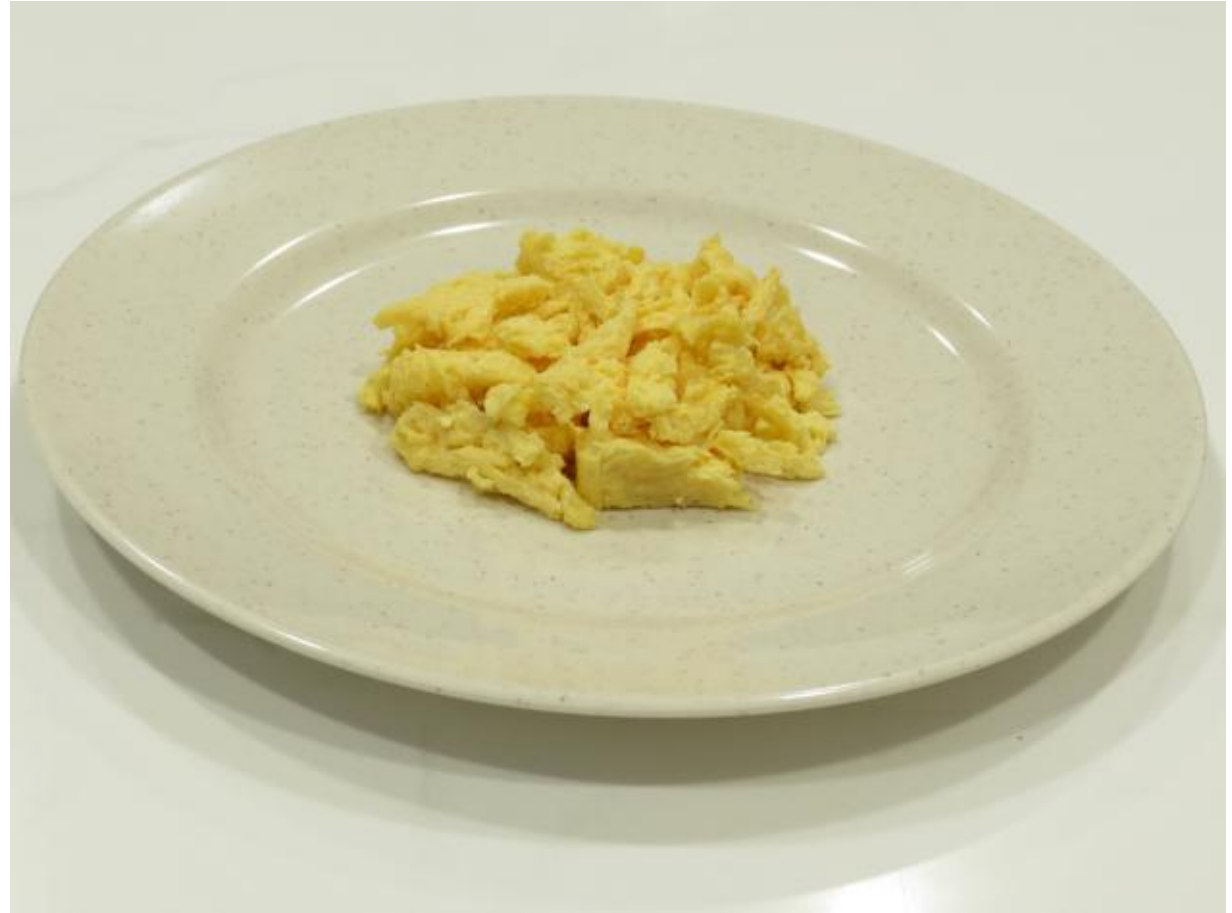

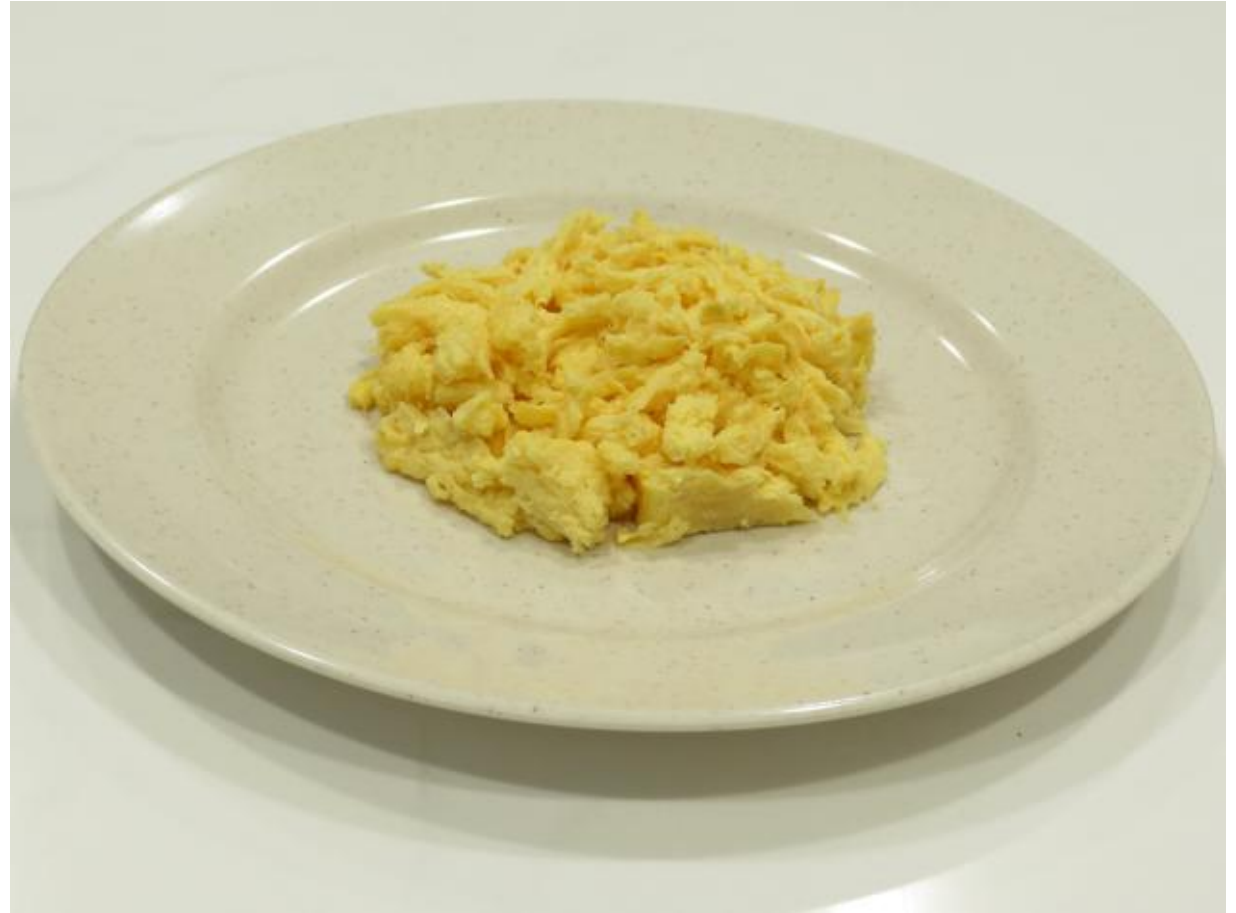

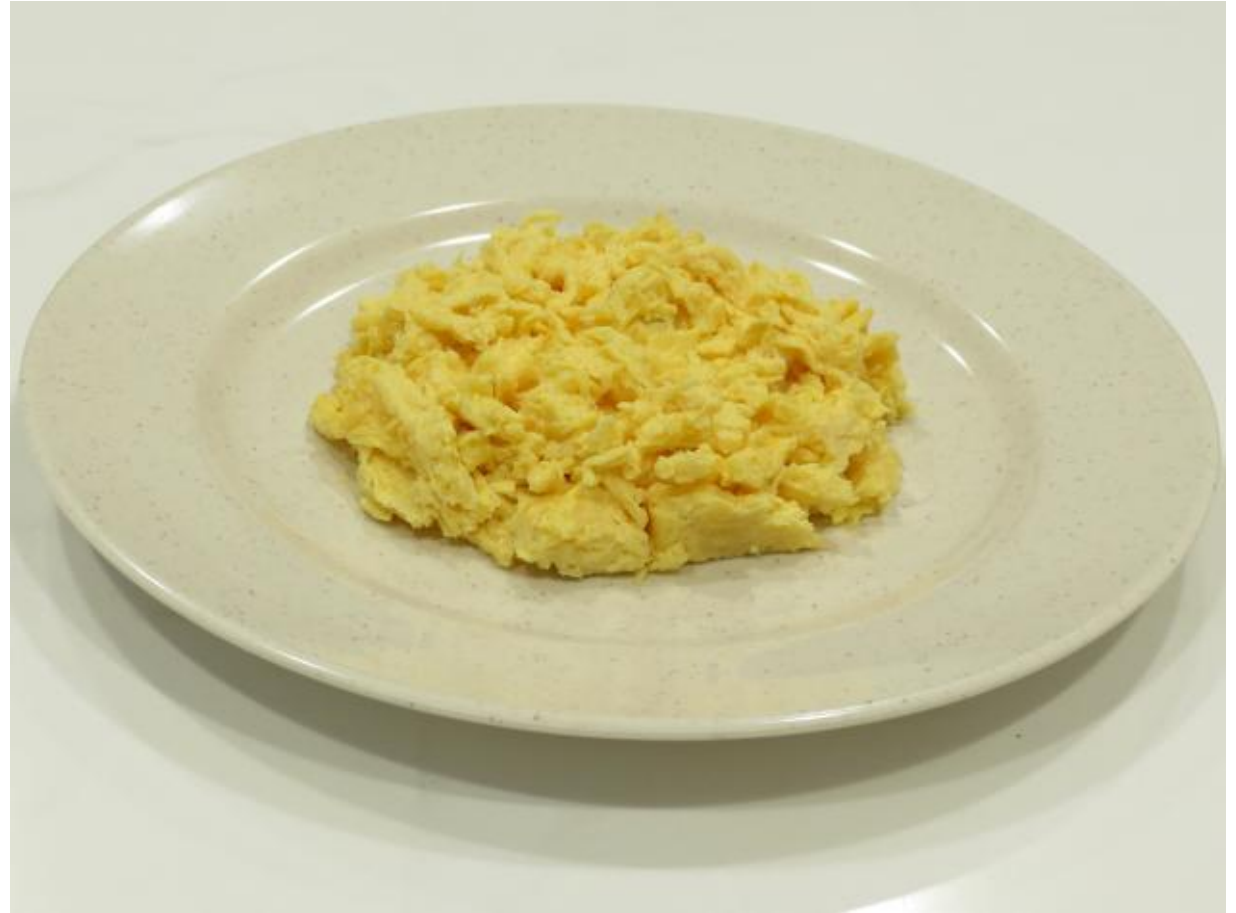

Clam / Lala (FA0120)

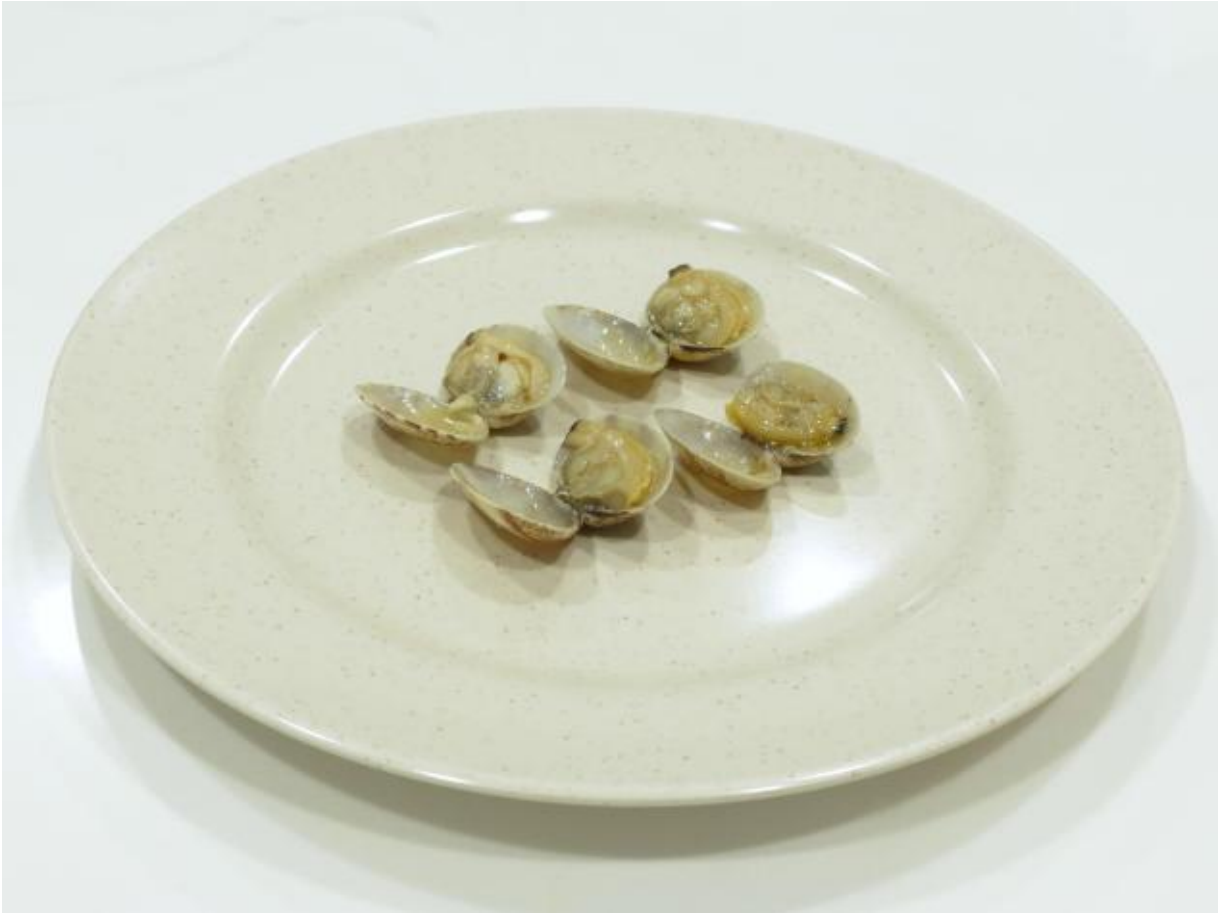
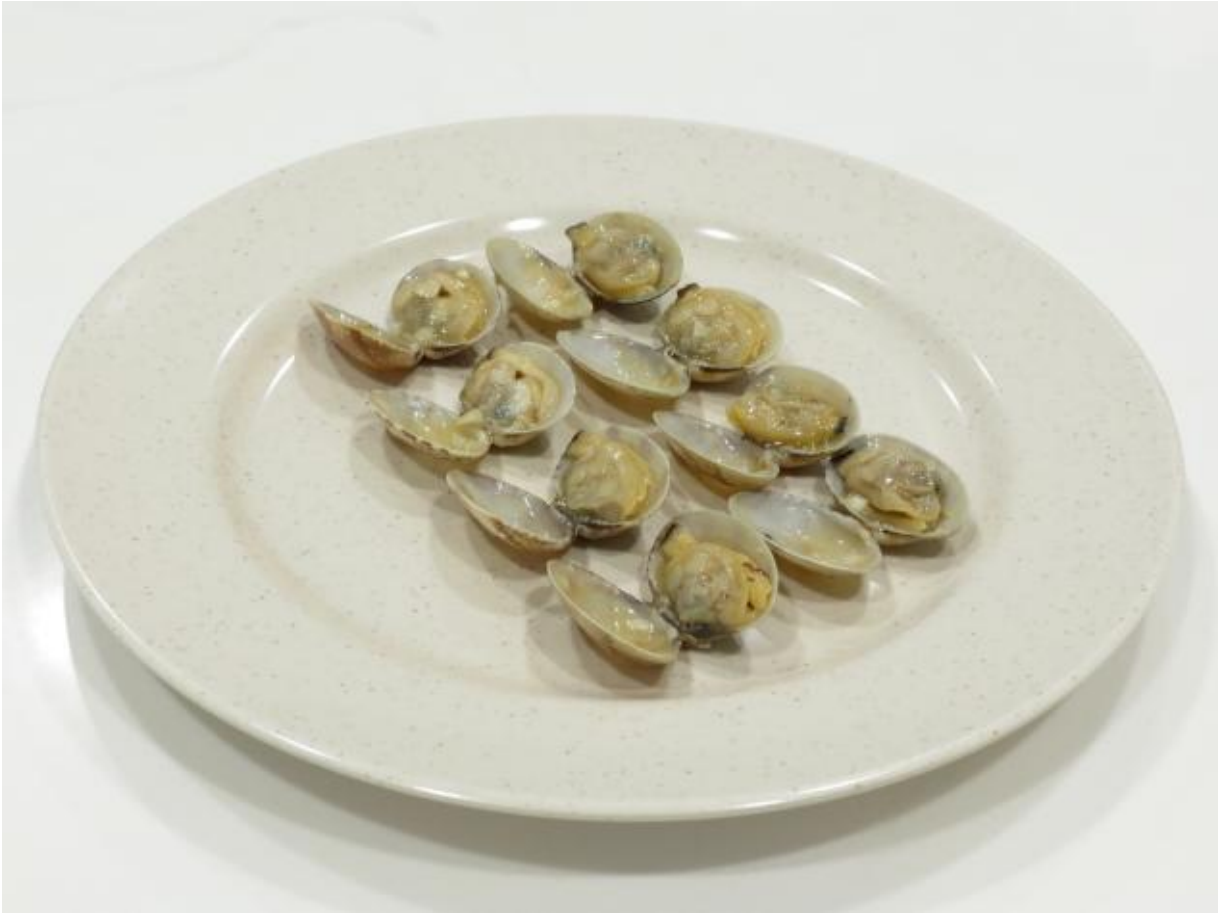
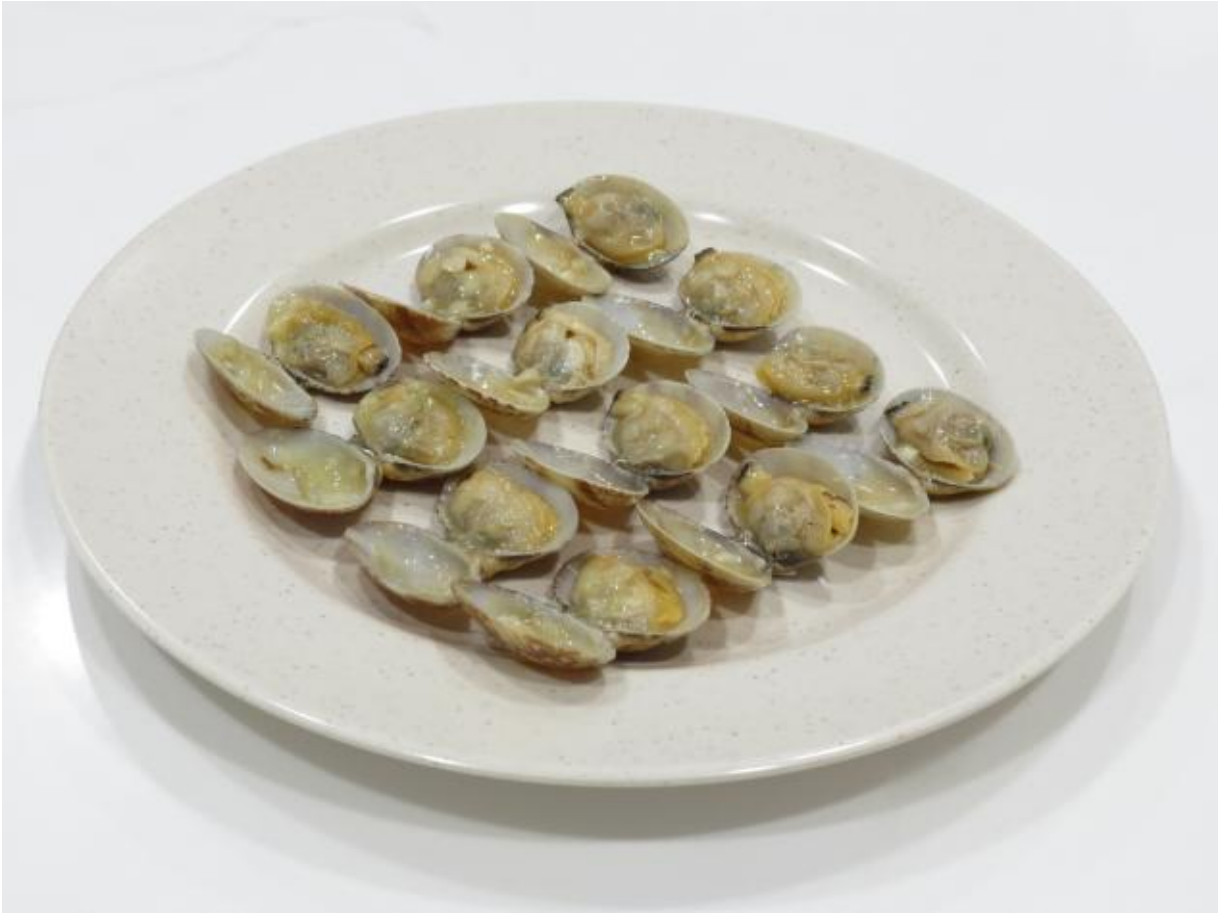
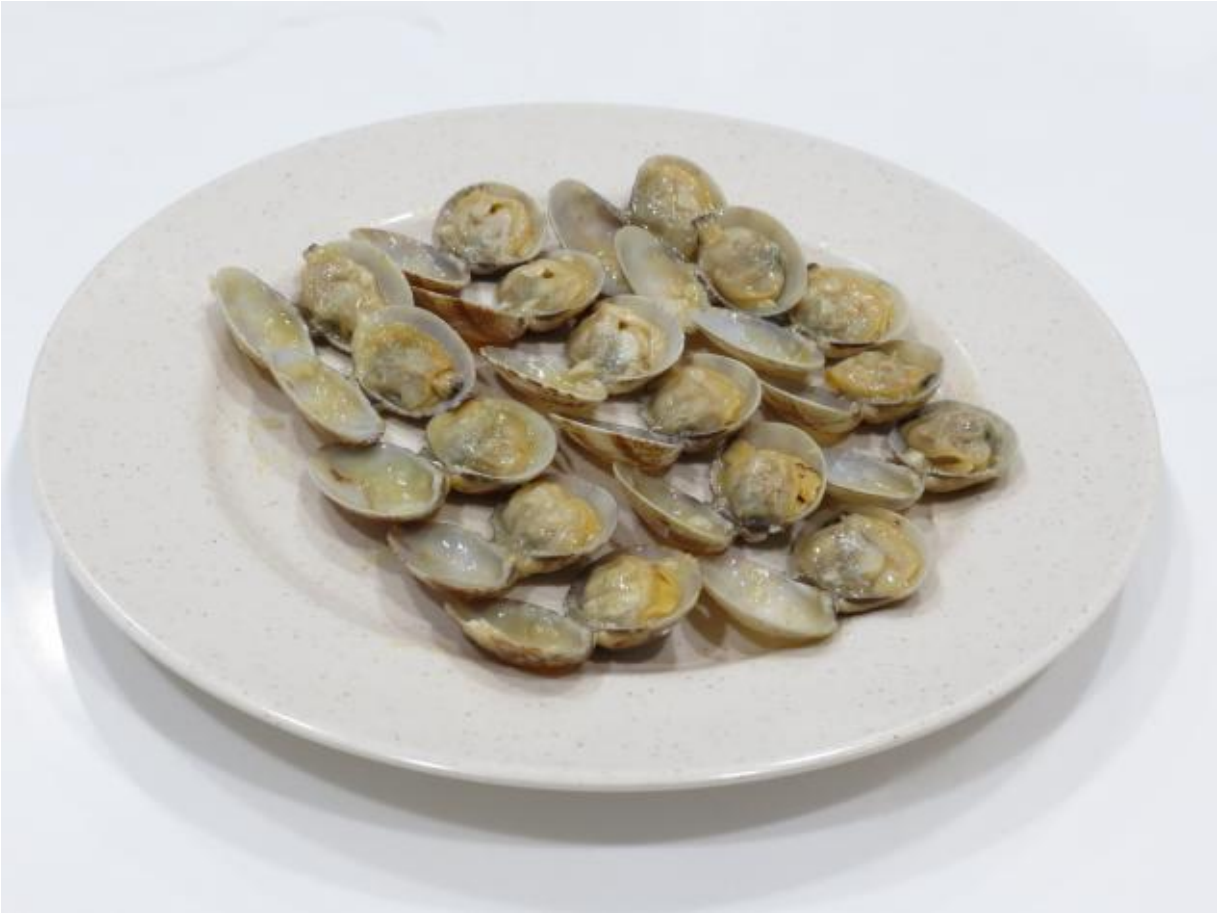

Fish, saba, boneless (FA0056)

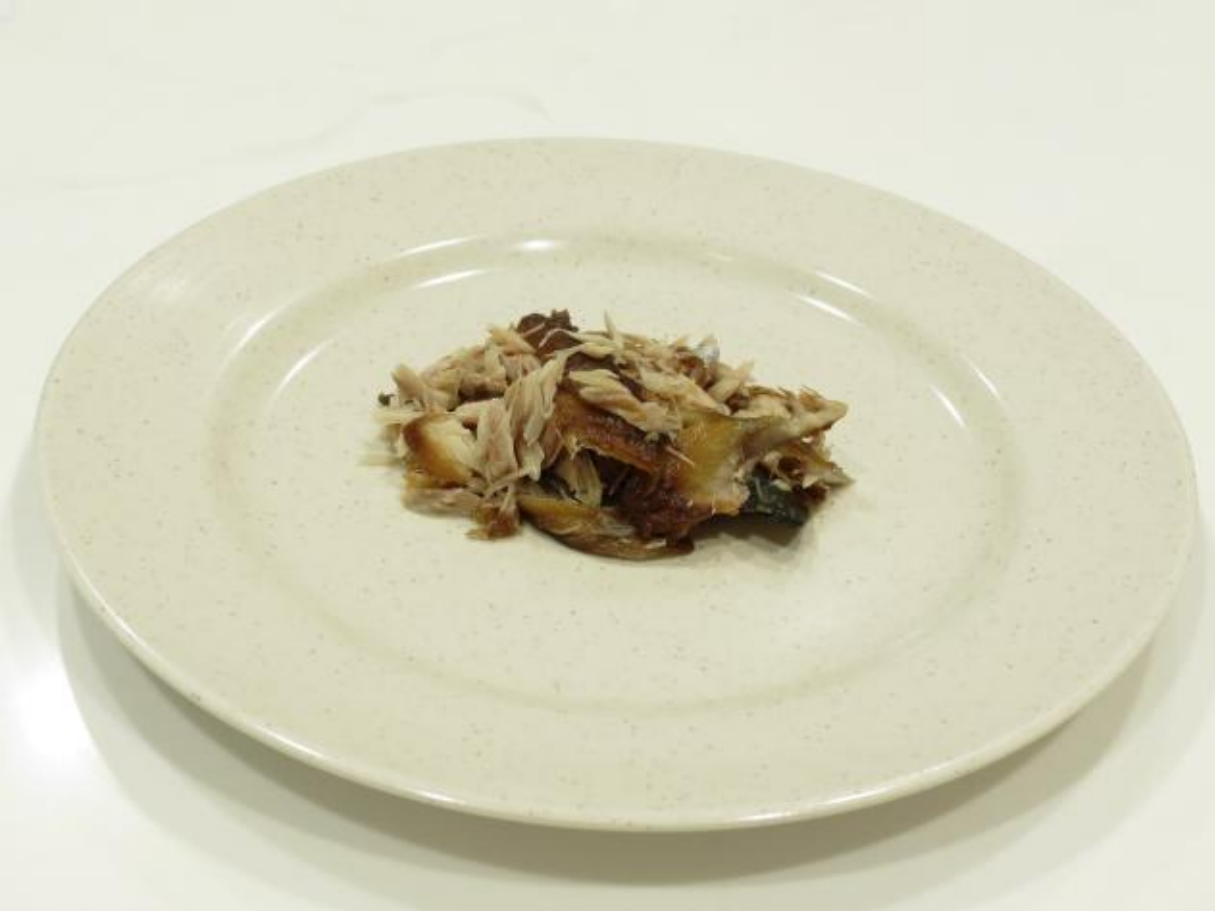

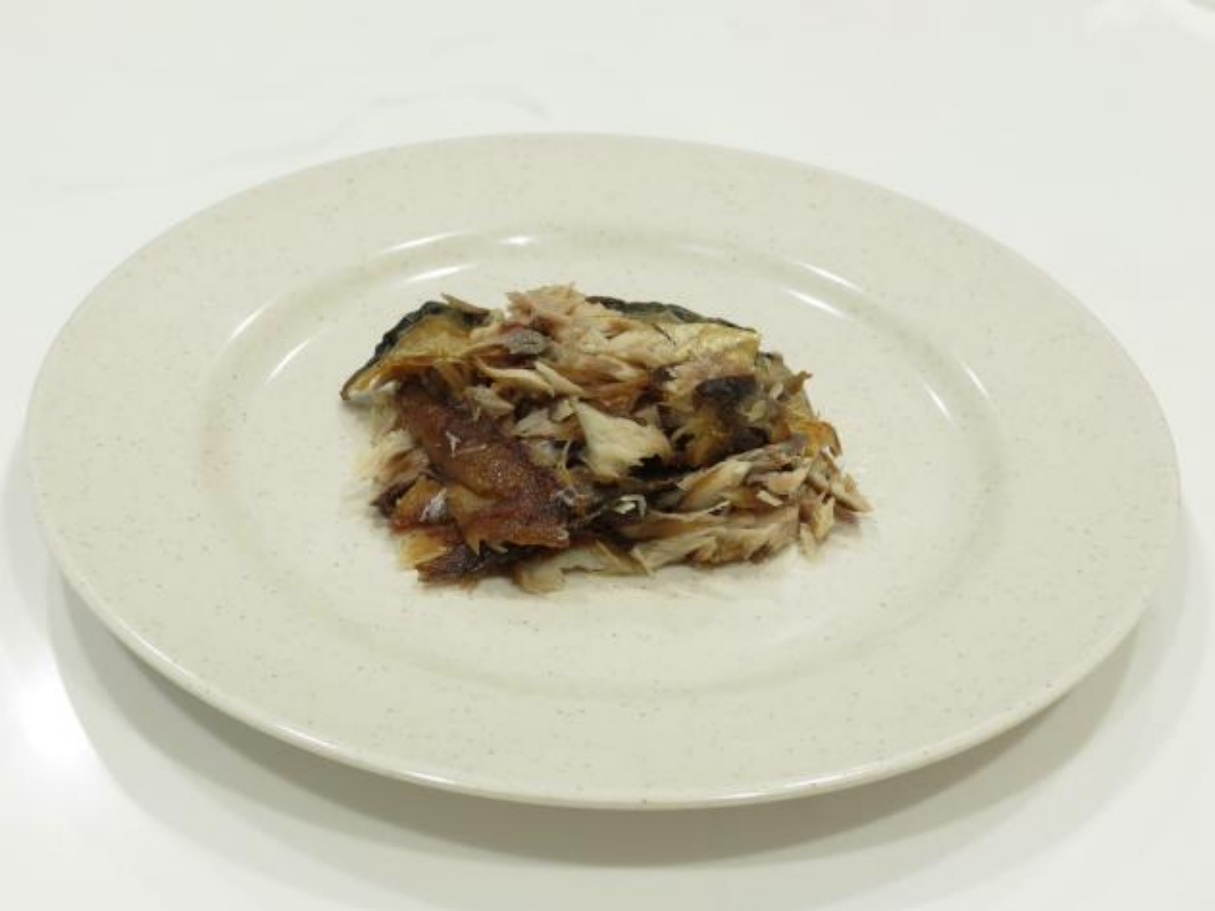

Fish, sardine, mashed (FA0112)

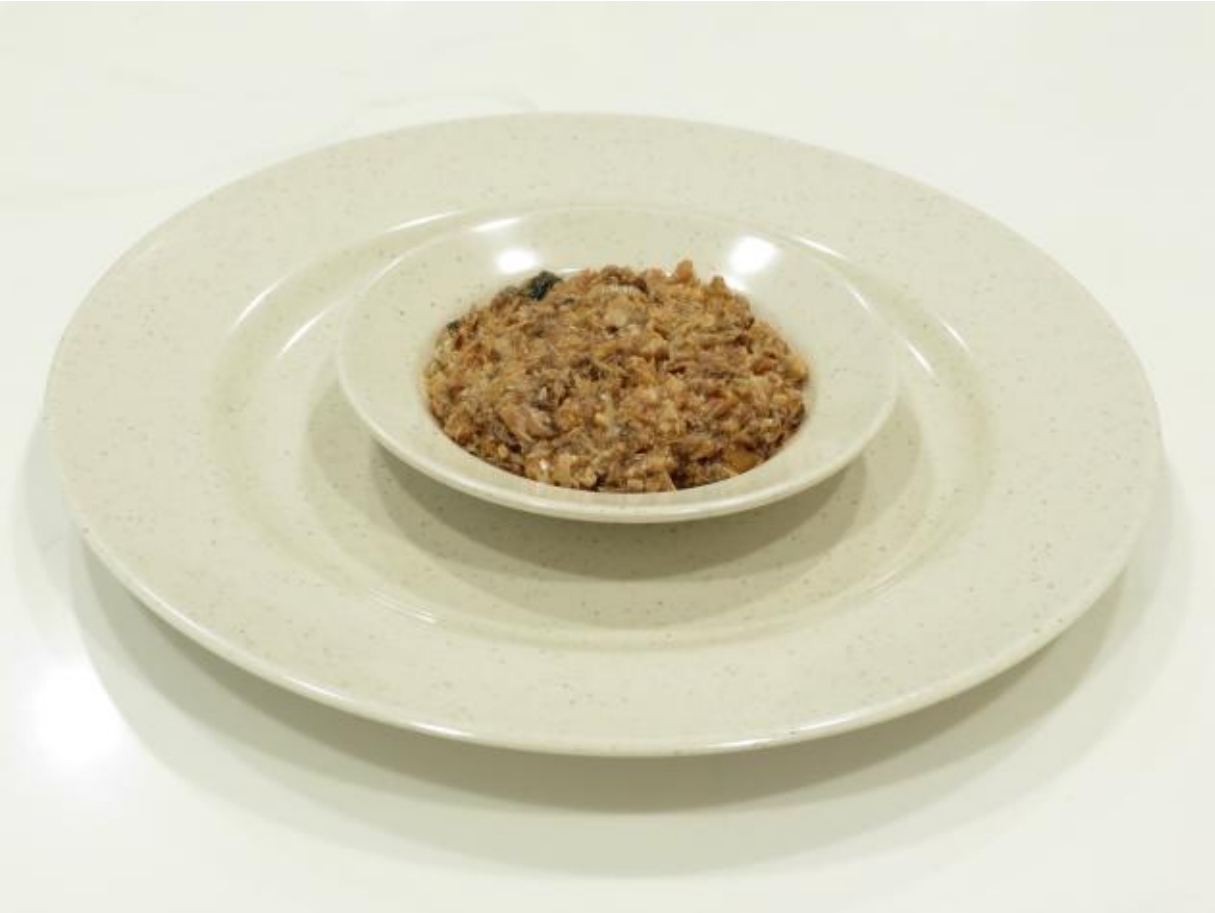

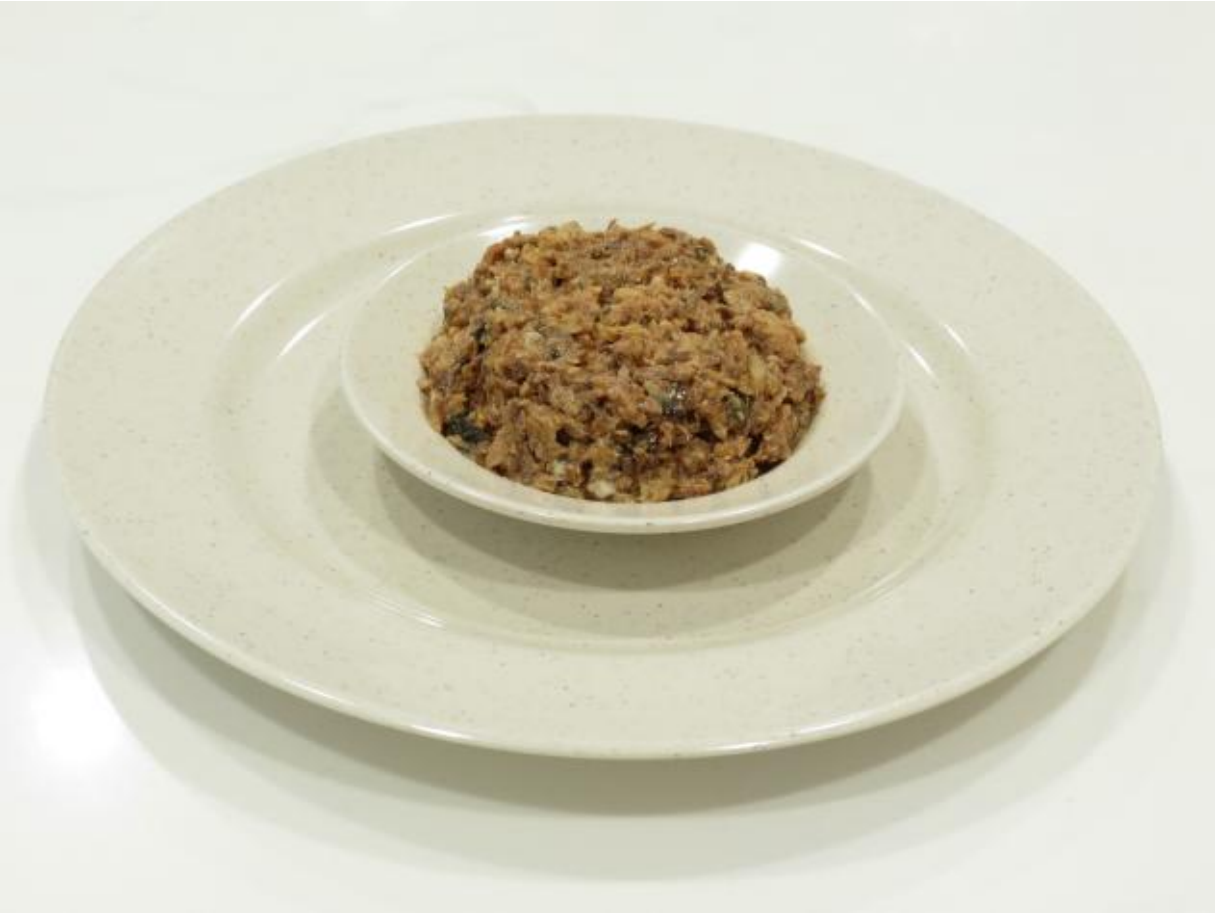

Fish, tuna, canned (FA0110)

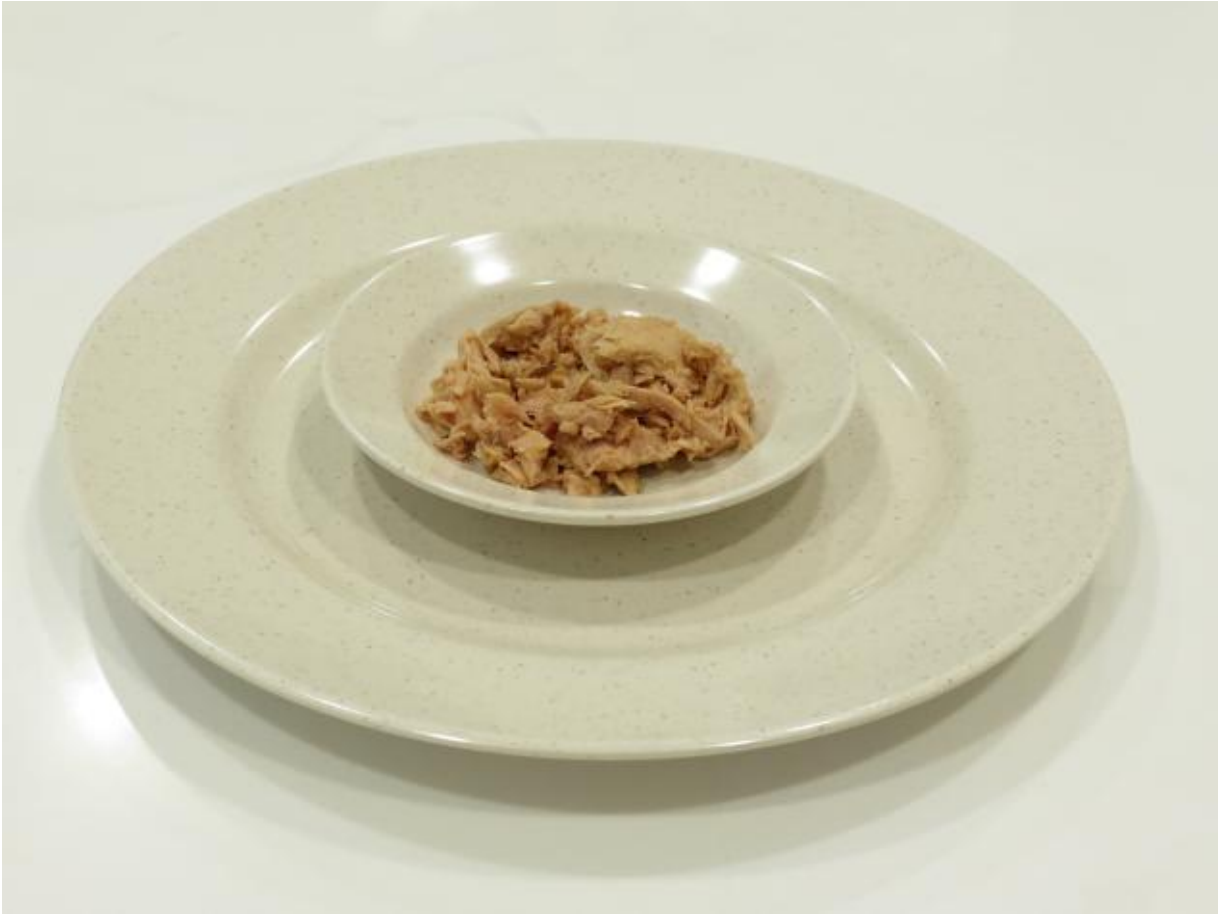

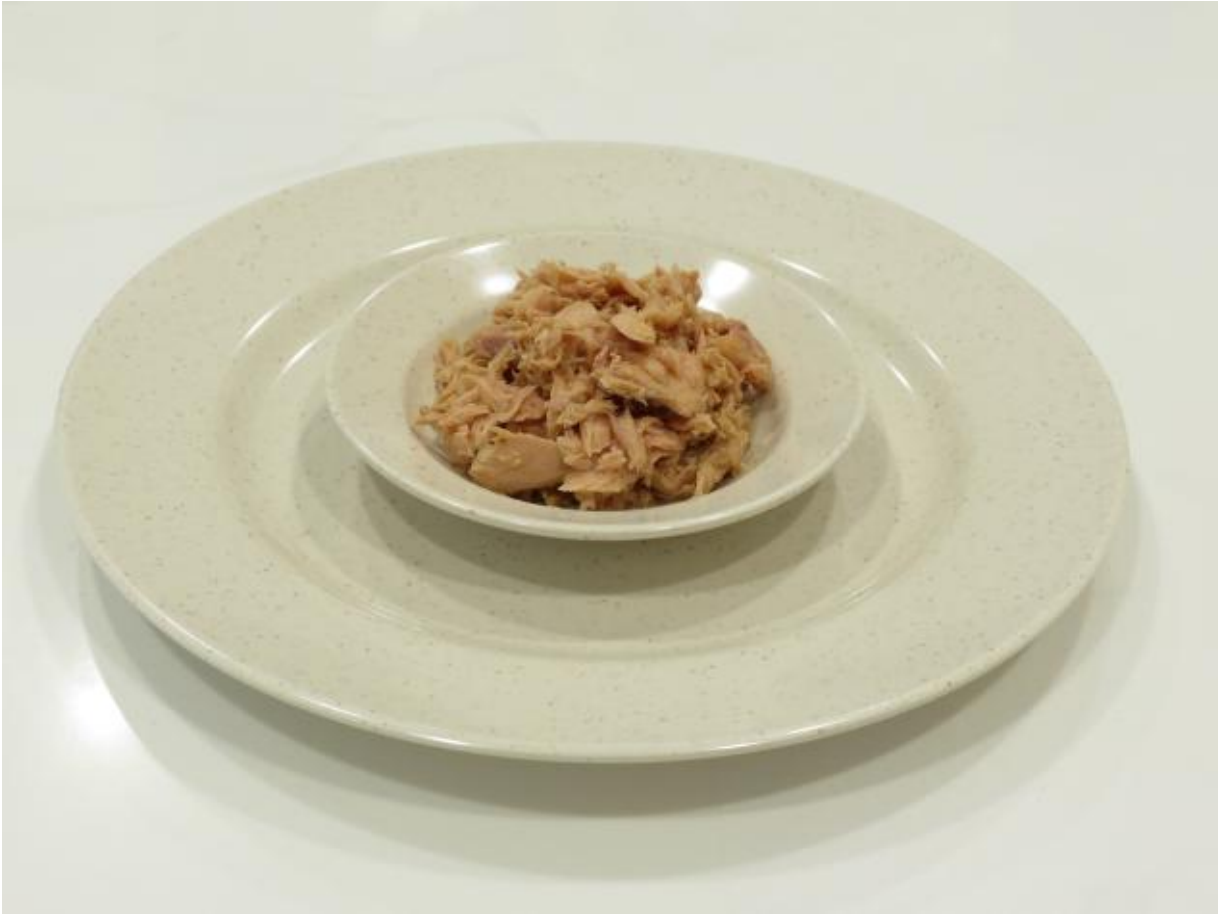

Fish, white, boneless, sliced (FA0101)

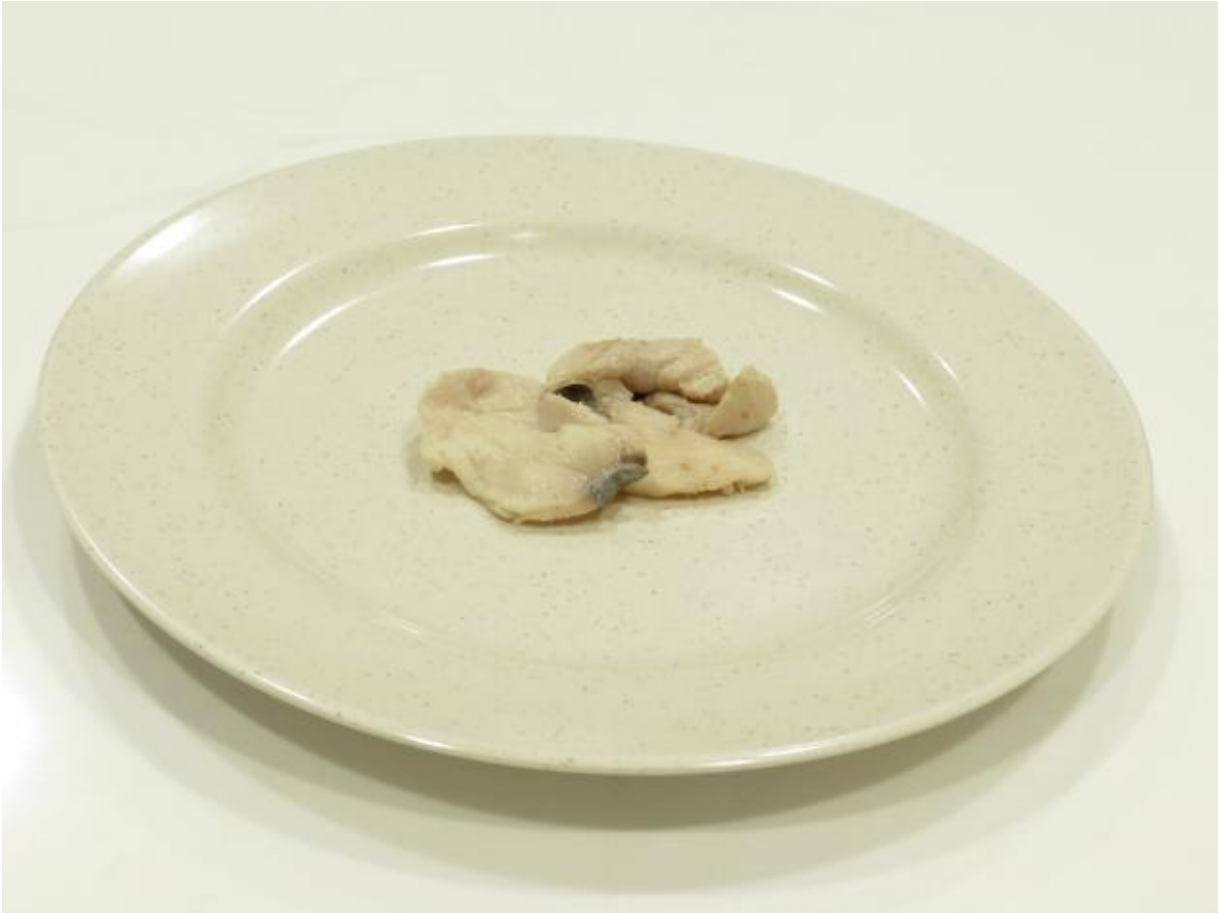
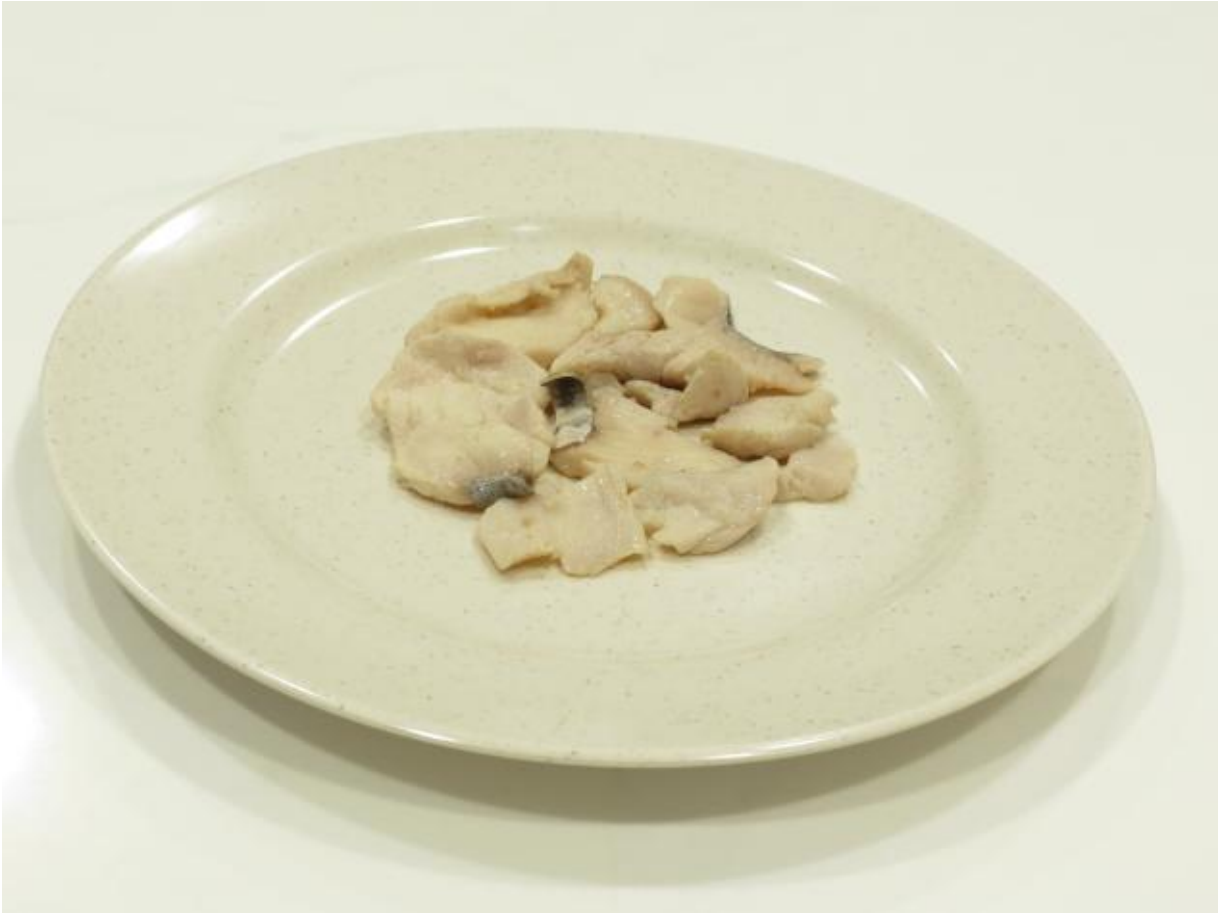
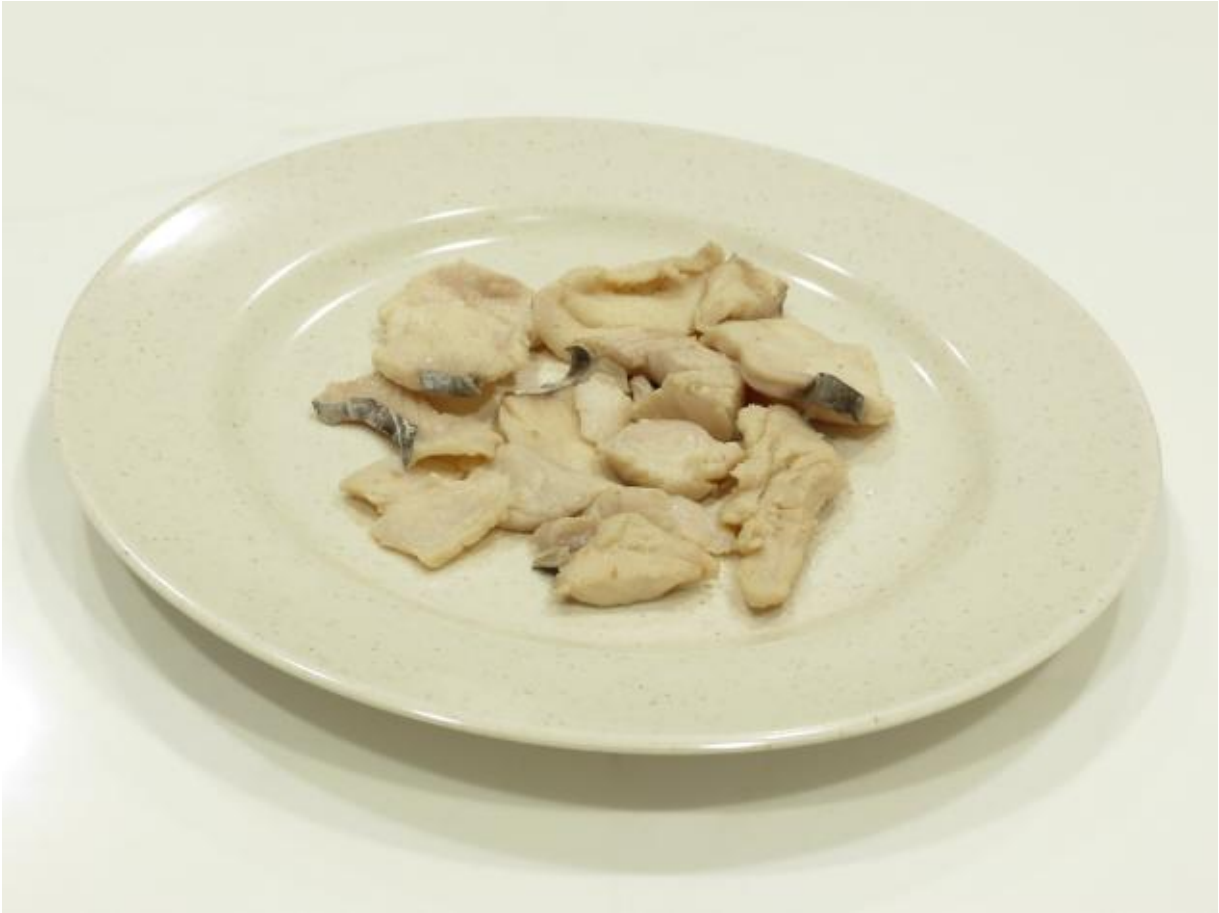
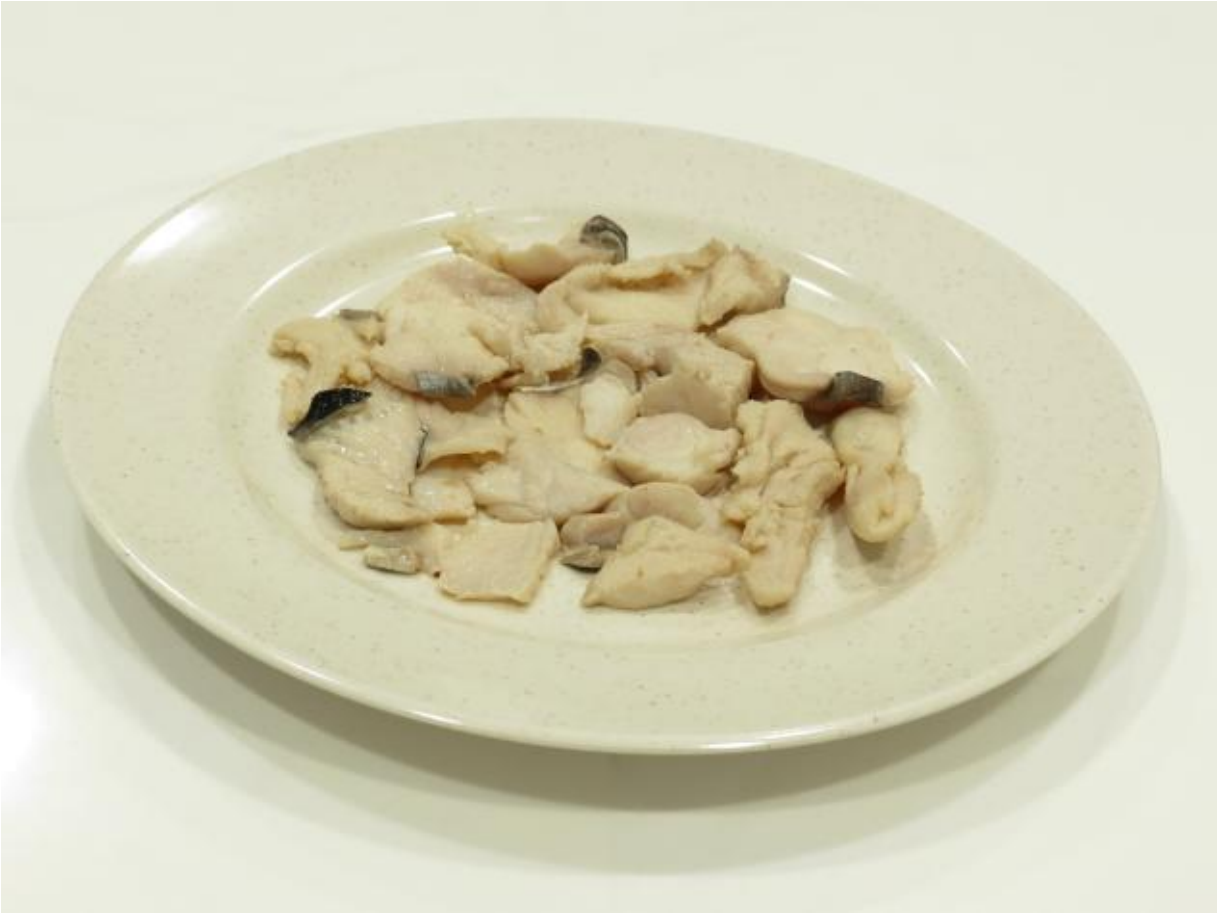

Squid, pieces (FA0155)

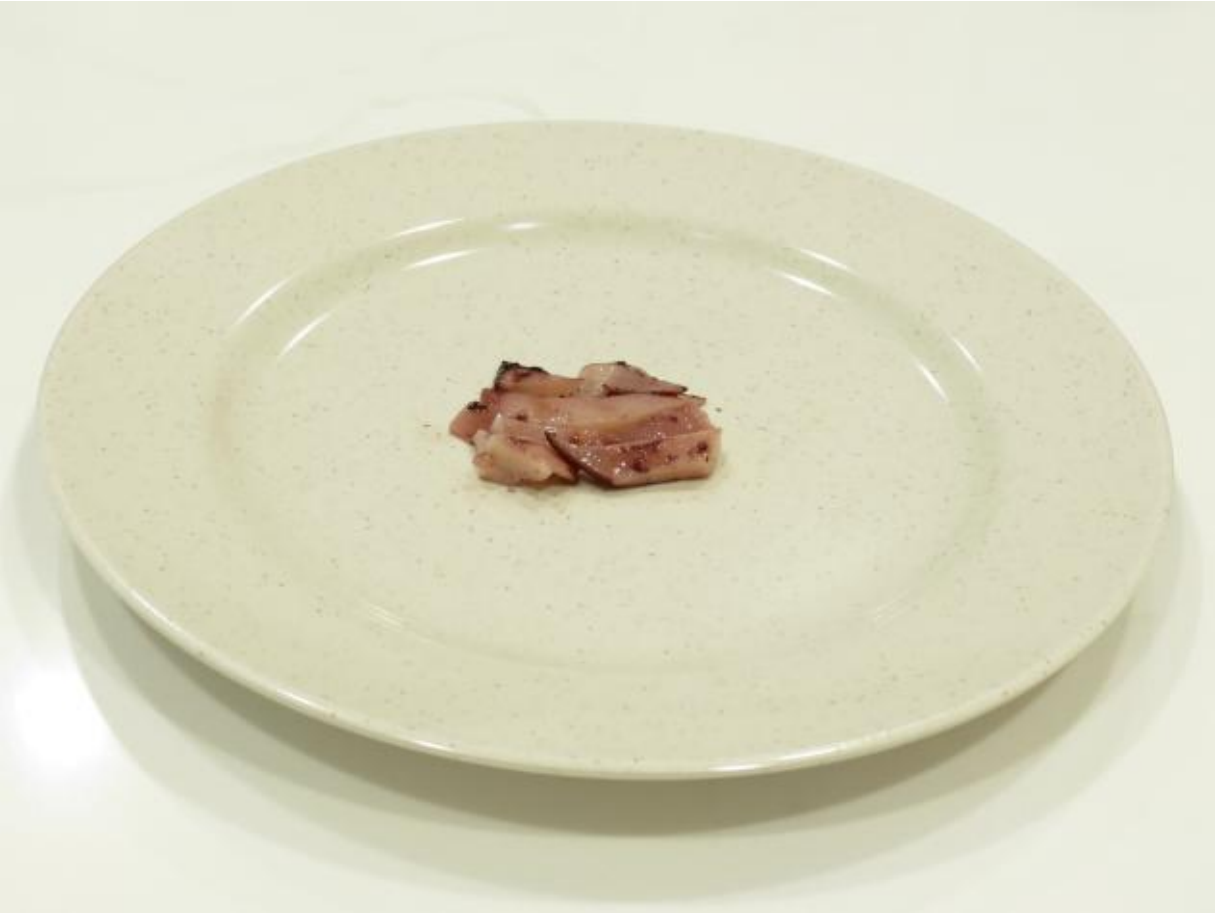

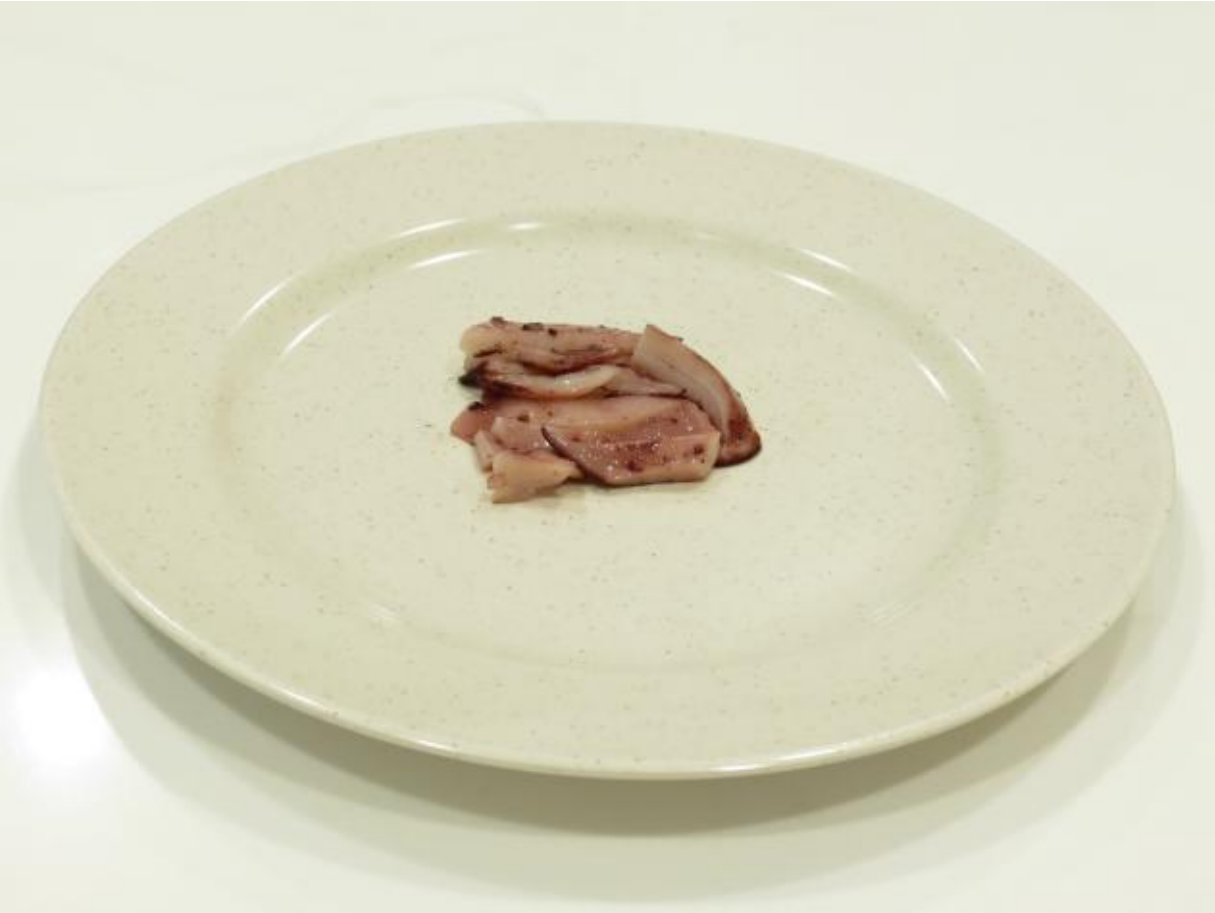

Blueberry (FA0033)

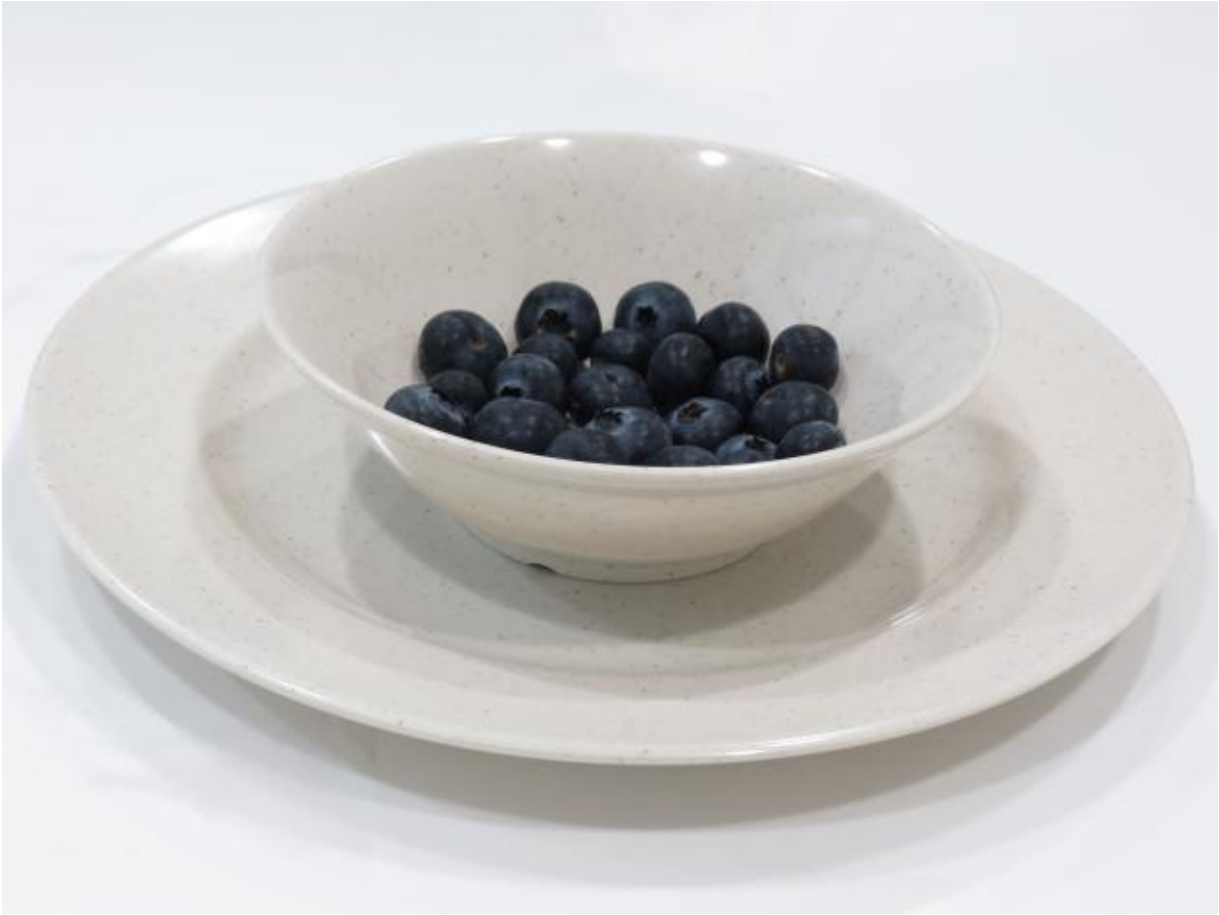

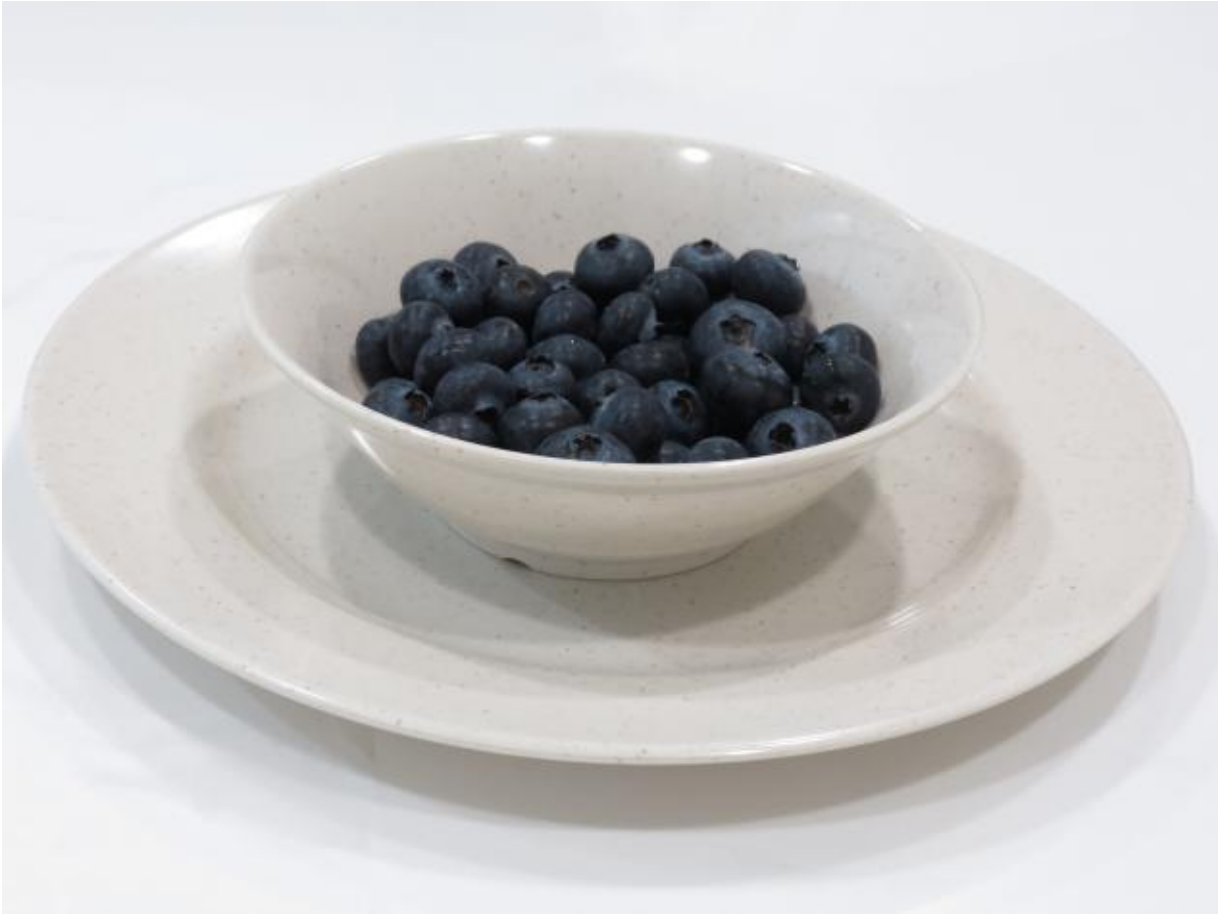

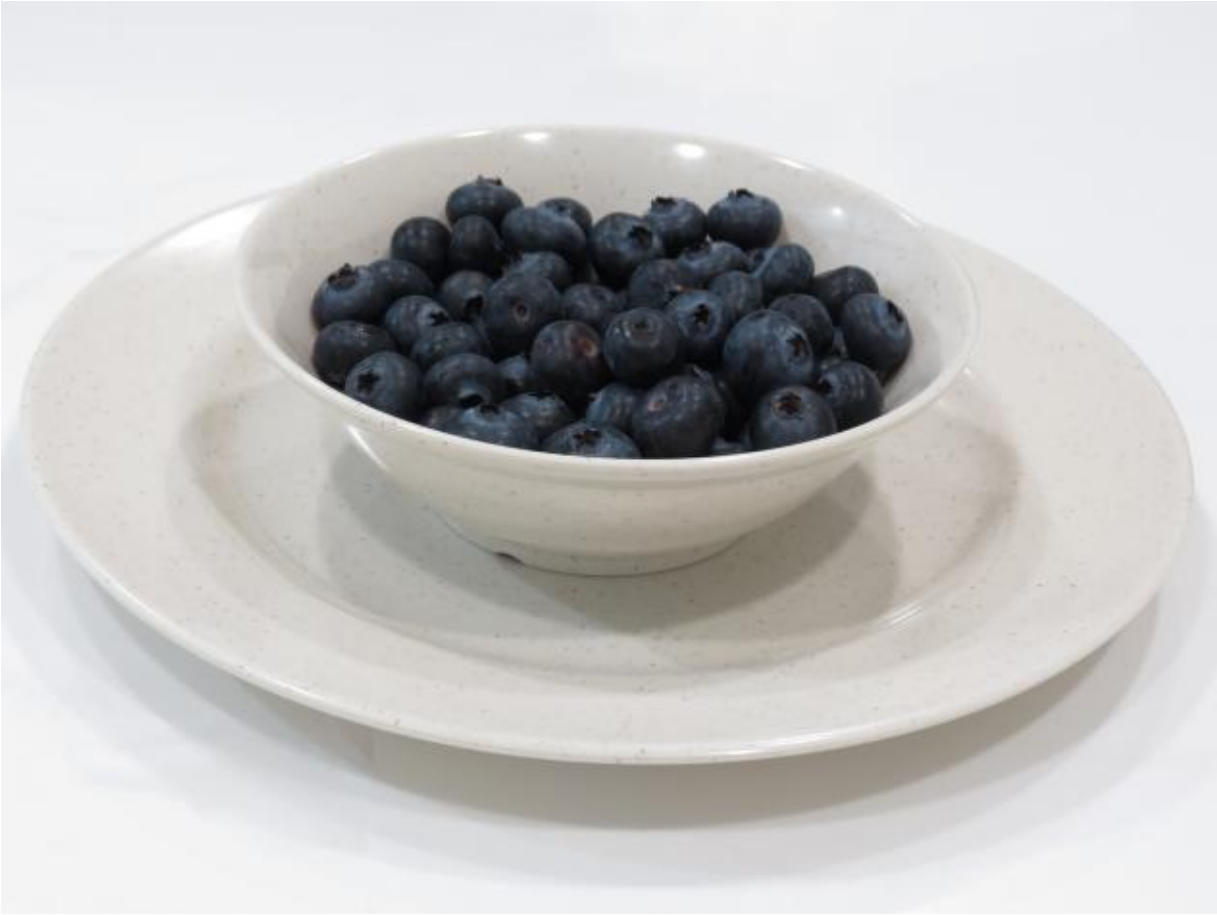

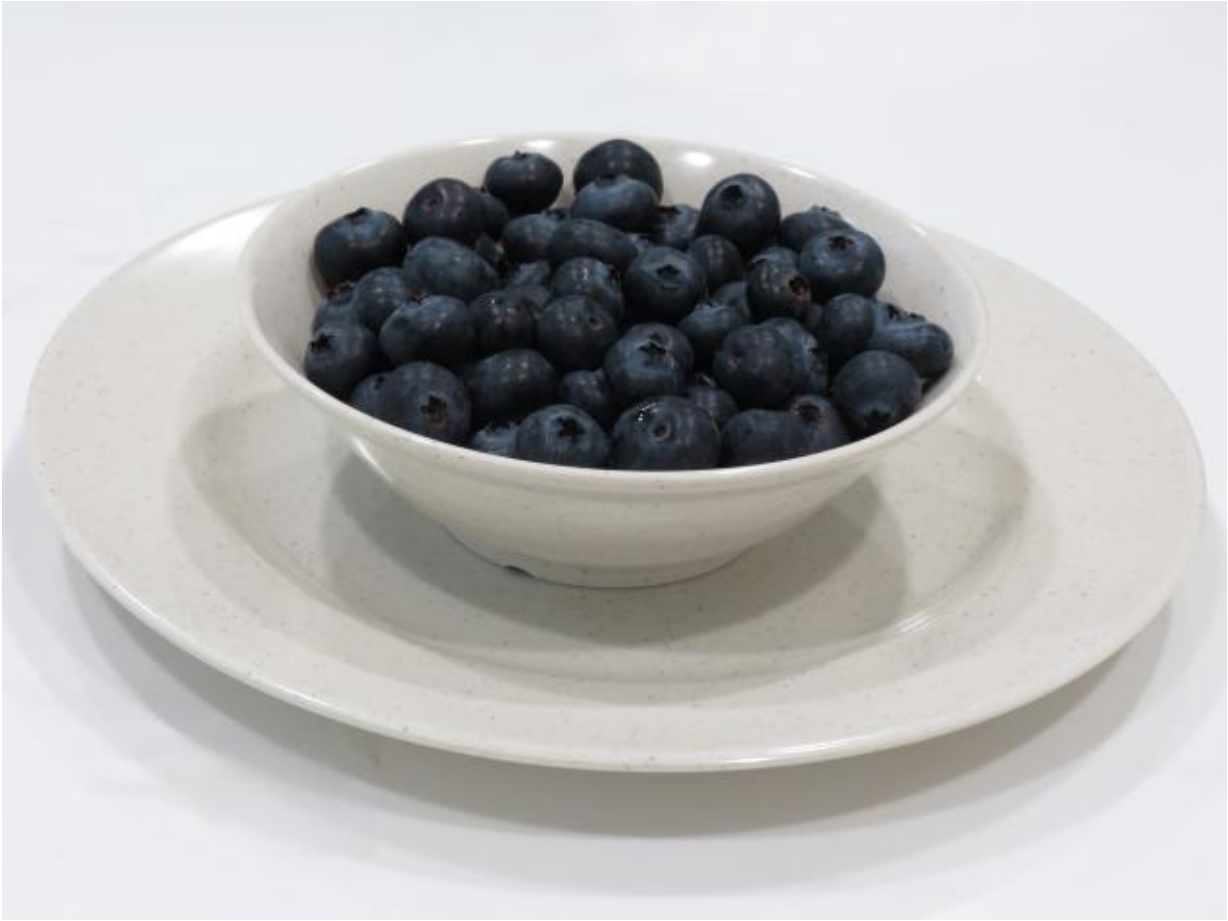

Papaya, pieces (FA0197)

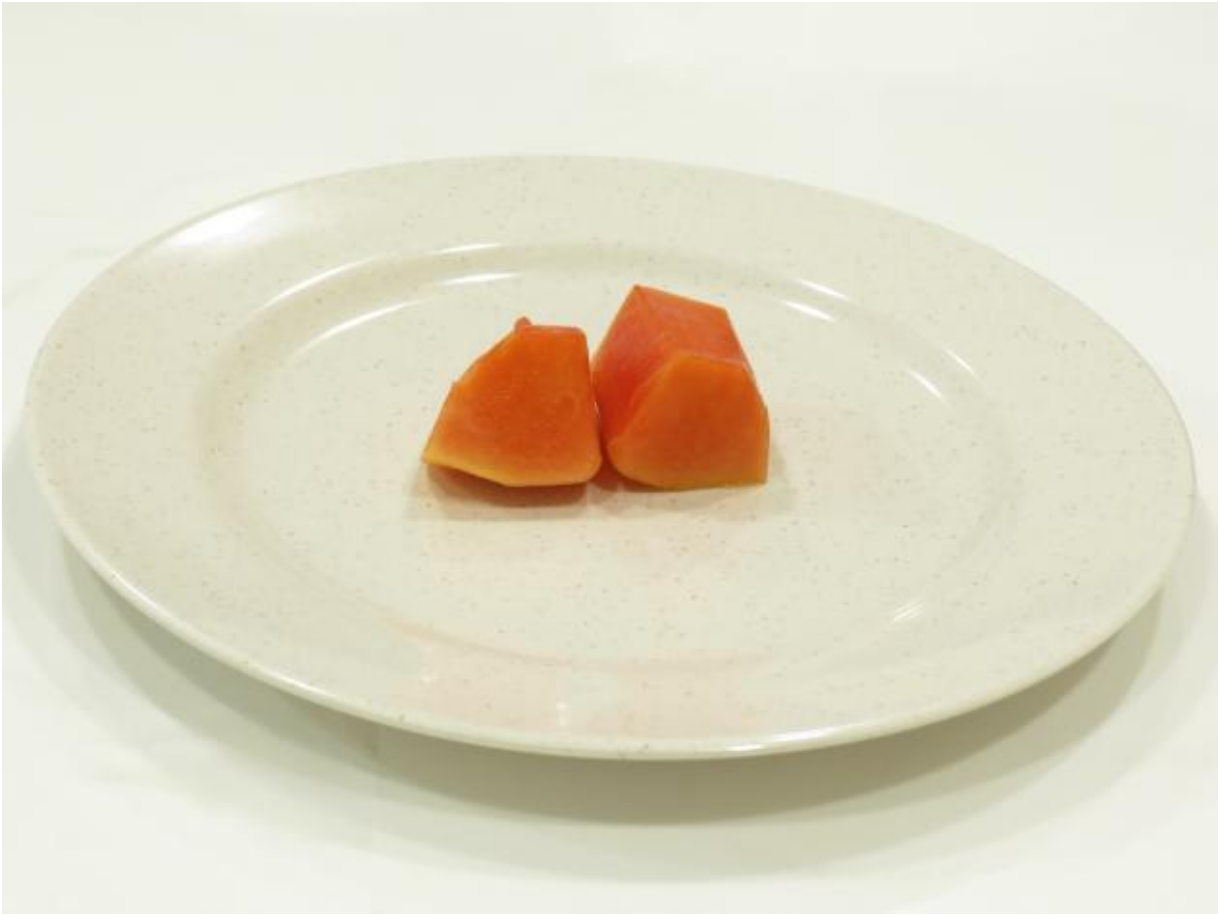
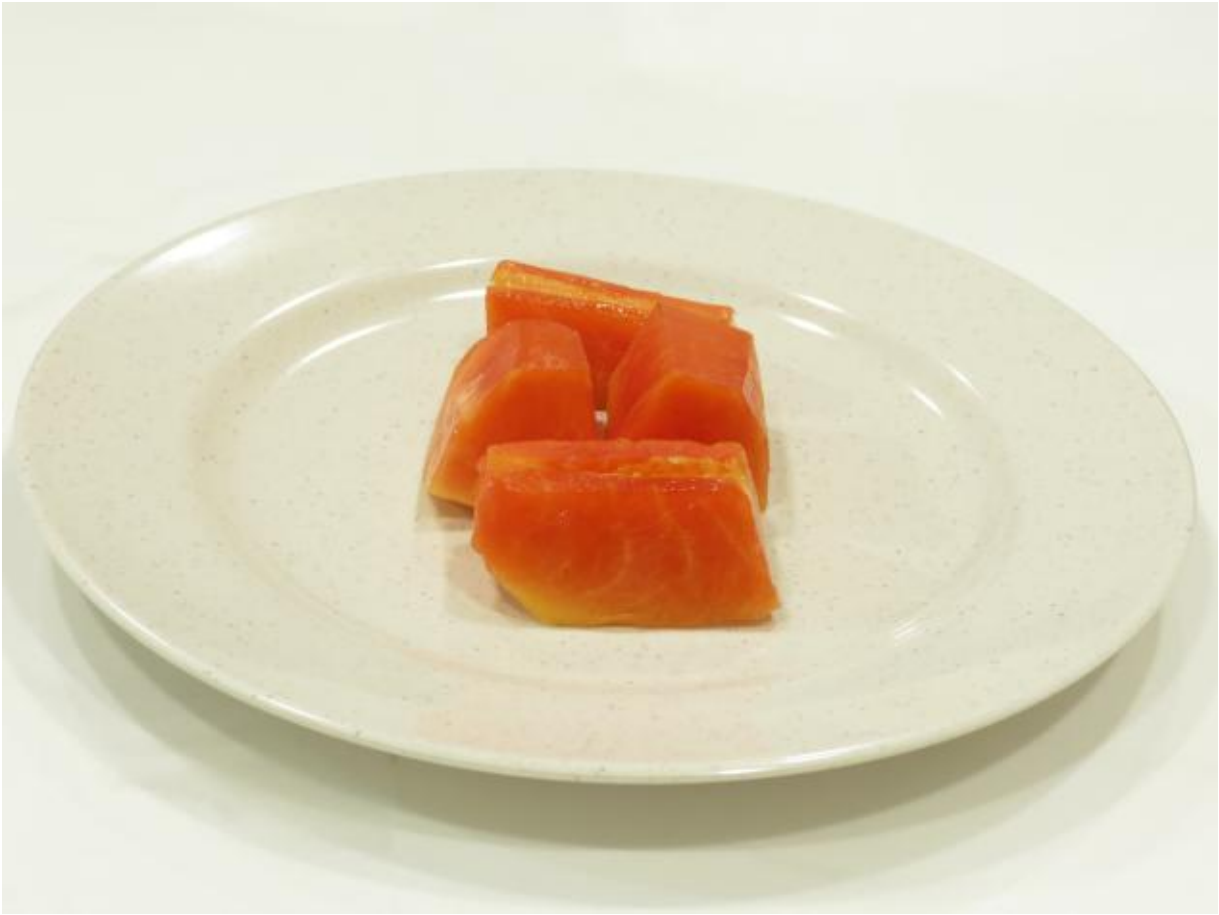
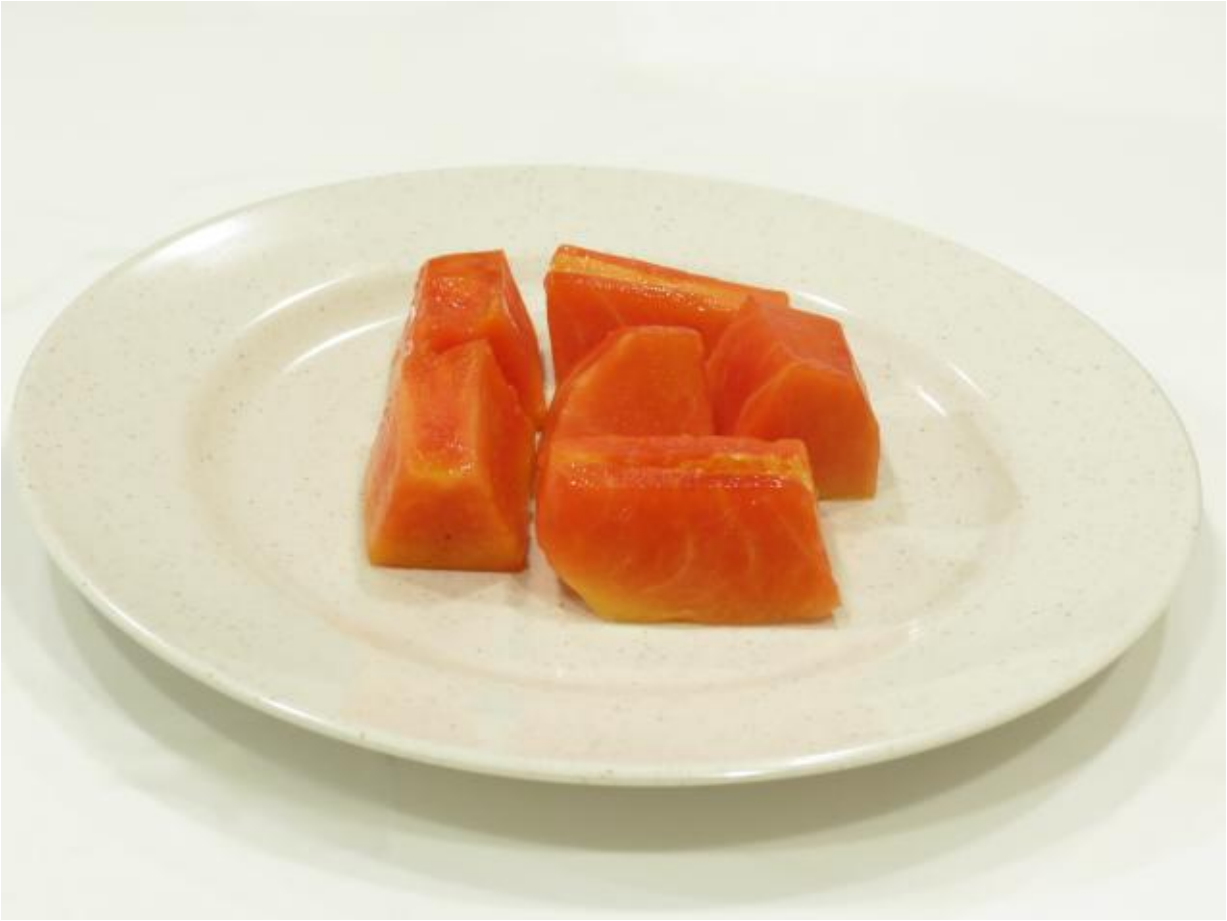
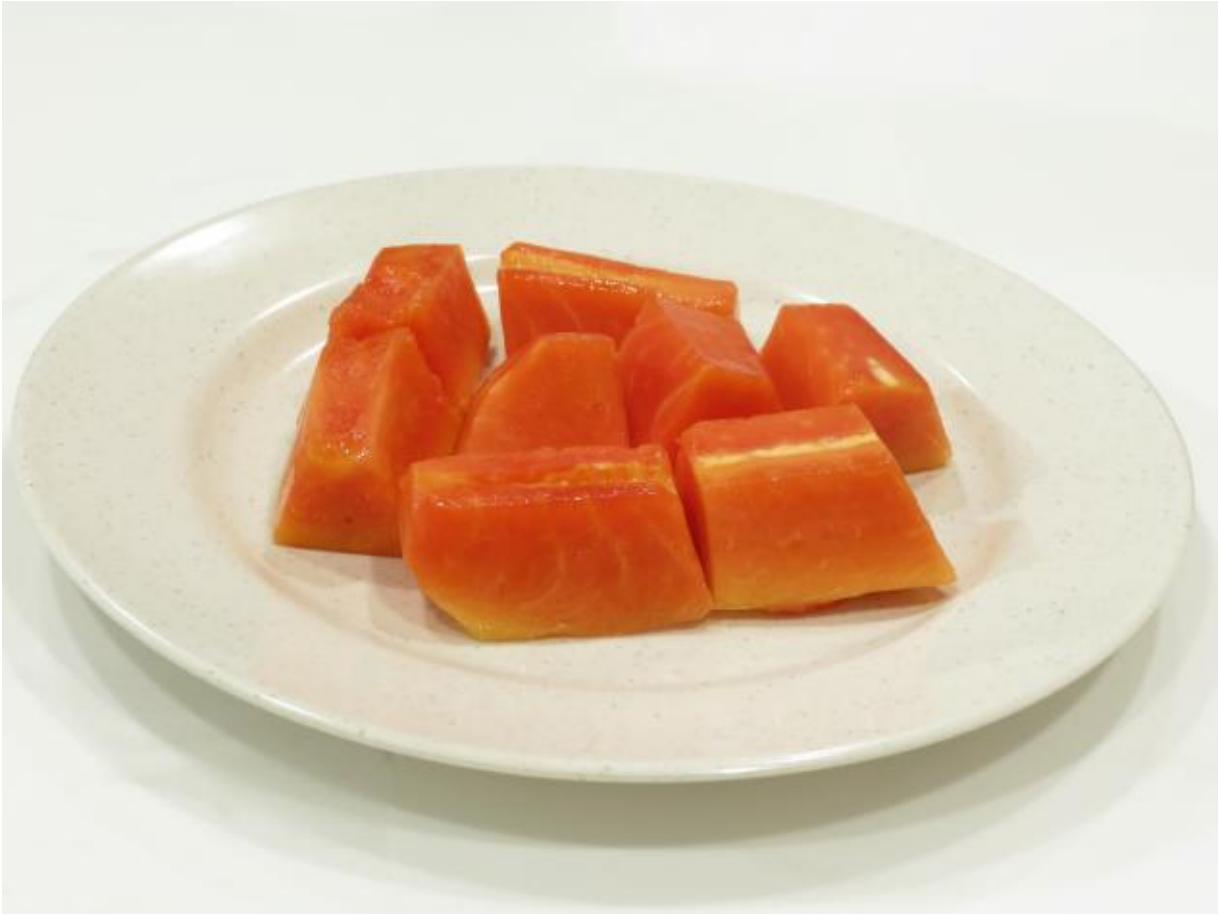

Raisin, seedless (FA0160)

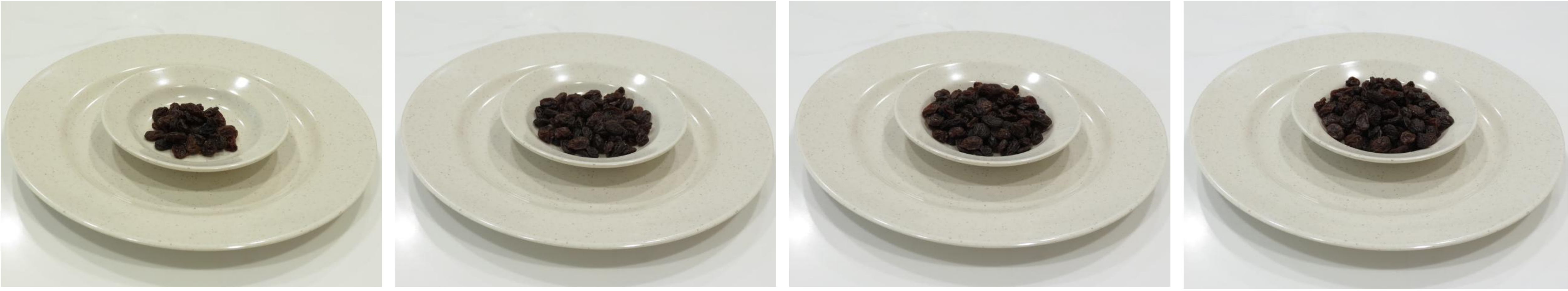

Strawberry (FA0004)

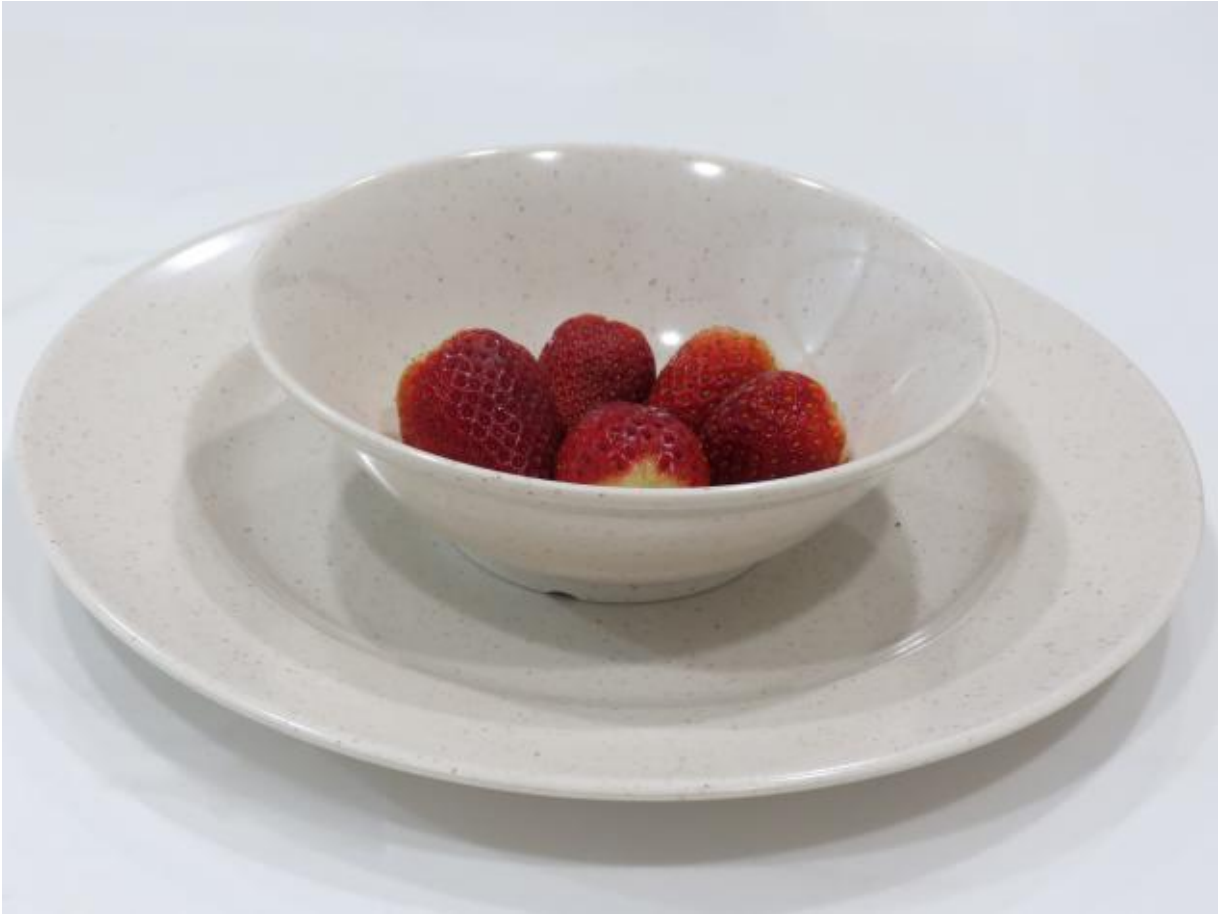
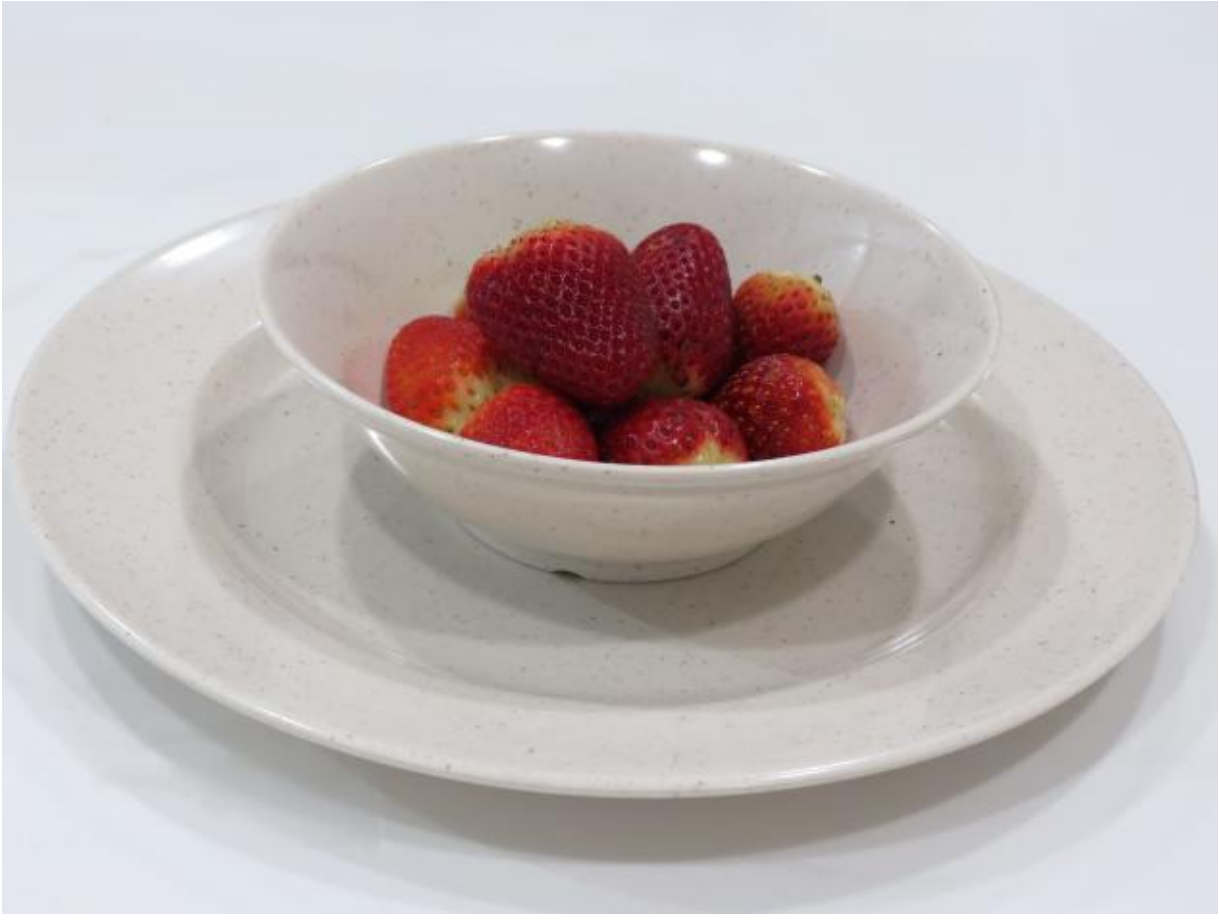
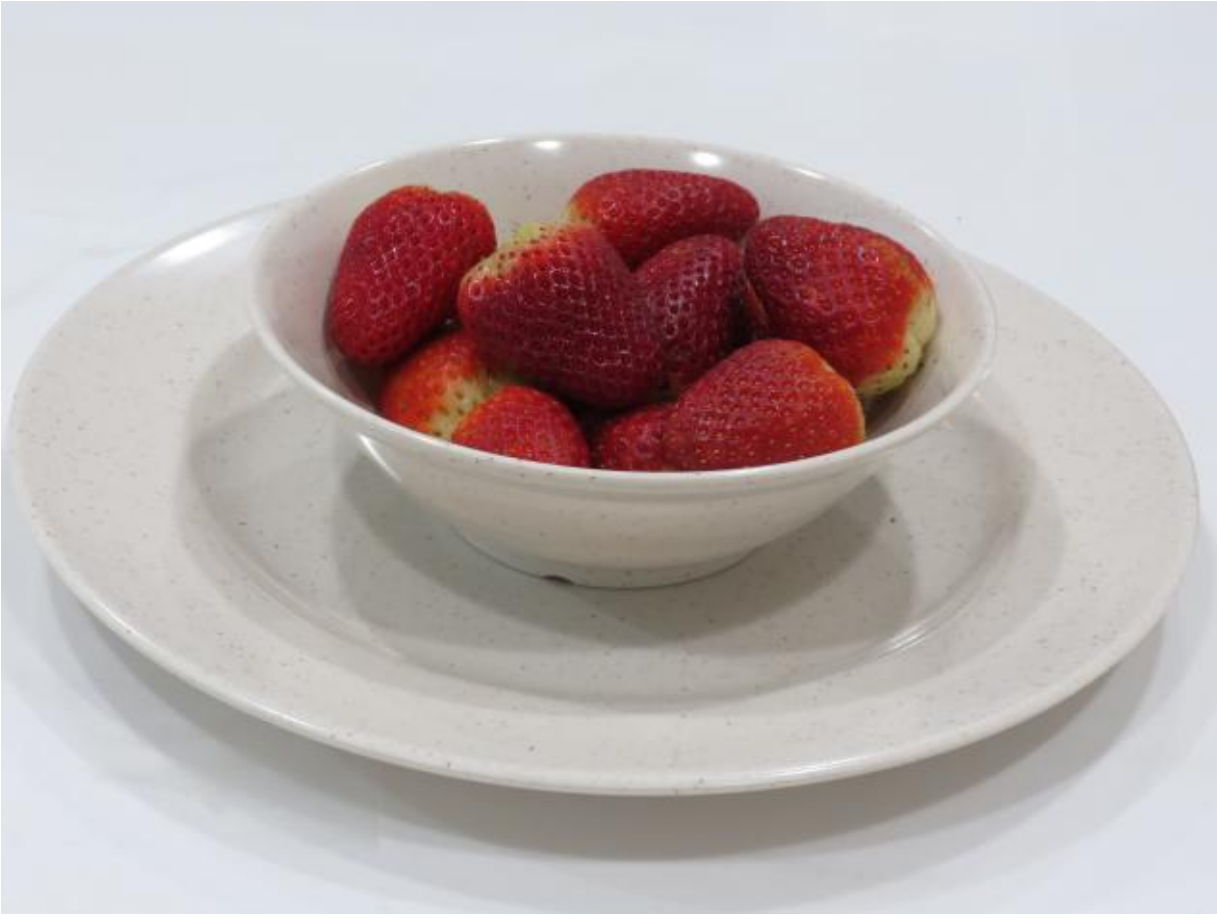
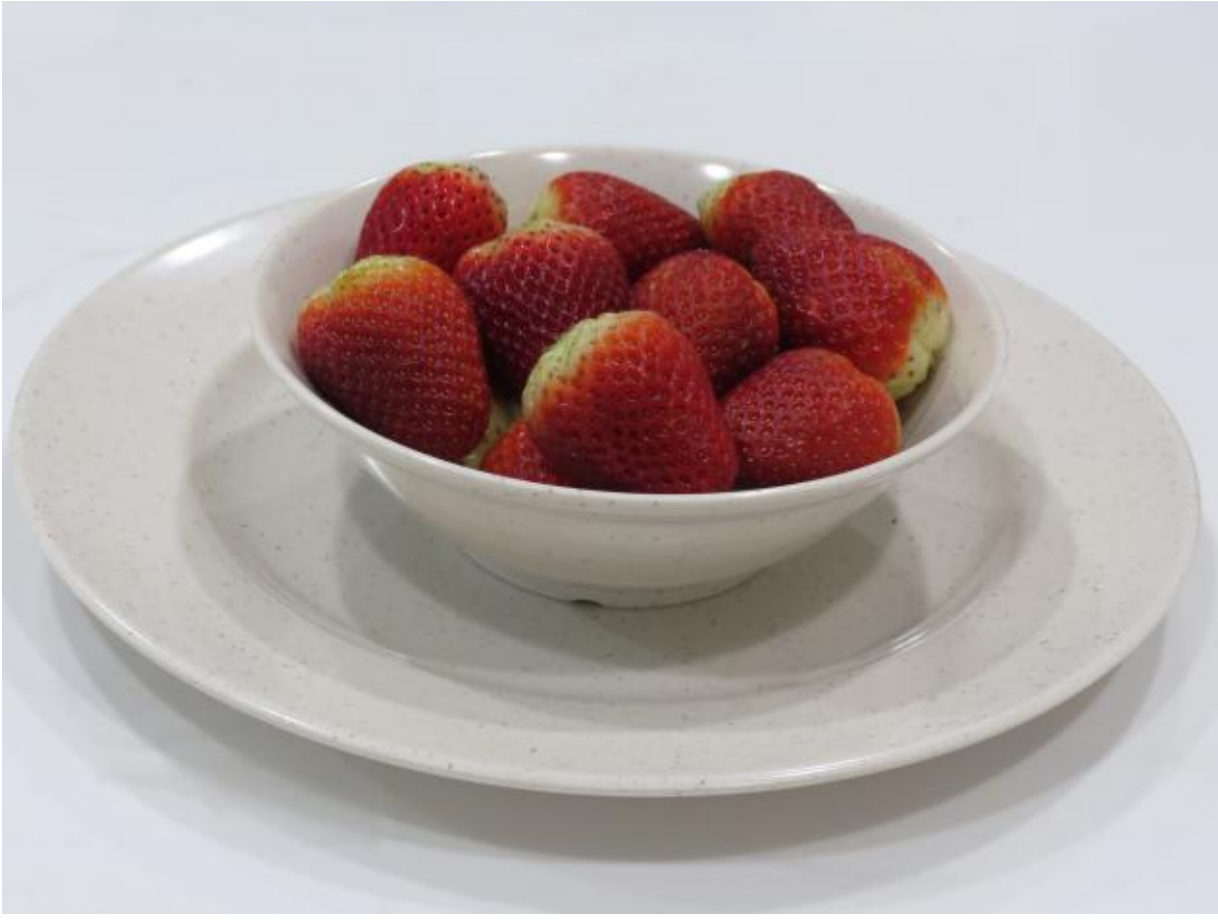

Mushroom, sliced (FA0091)

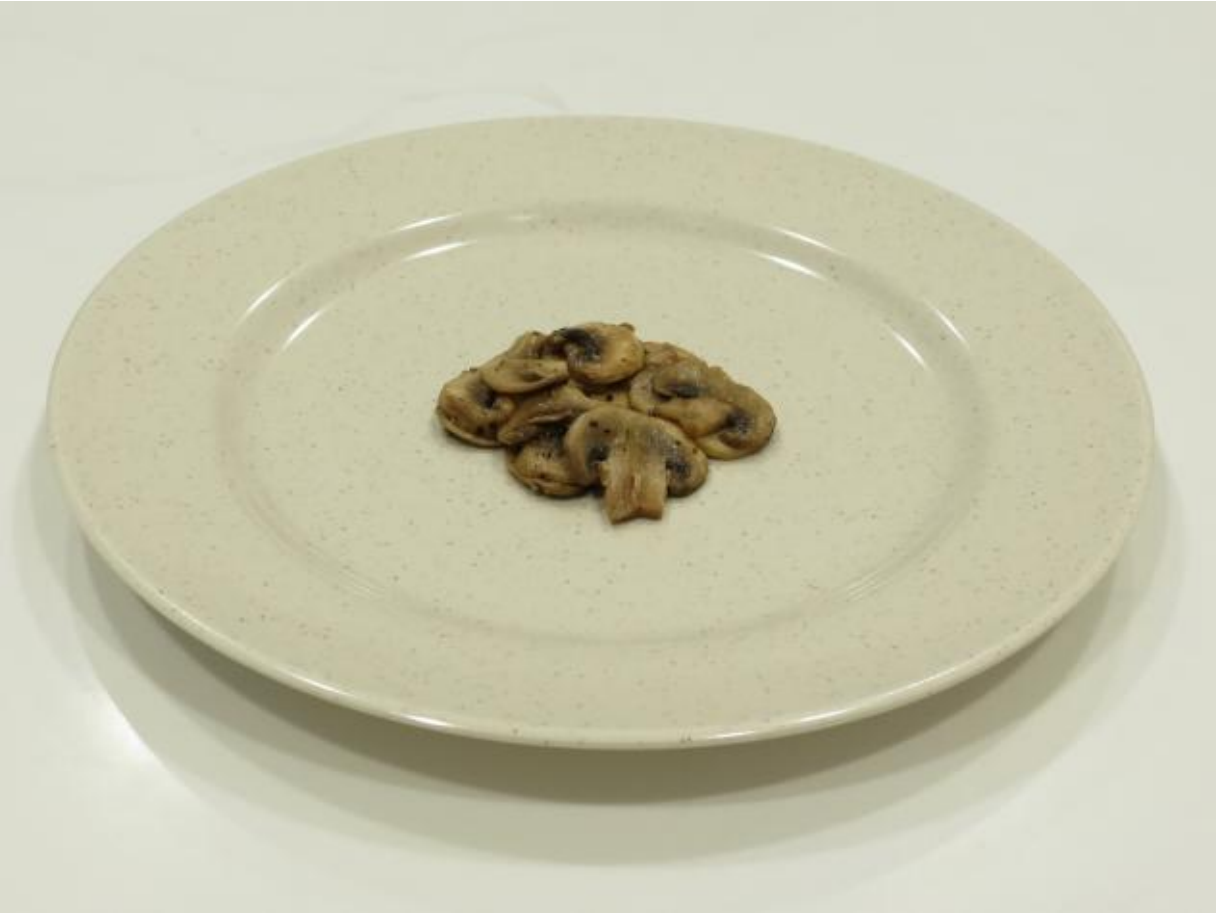

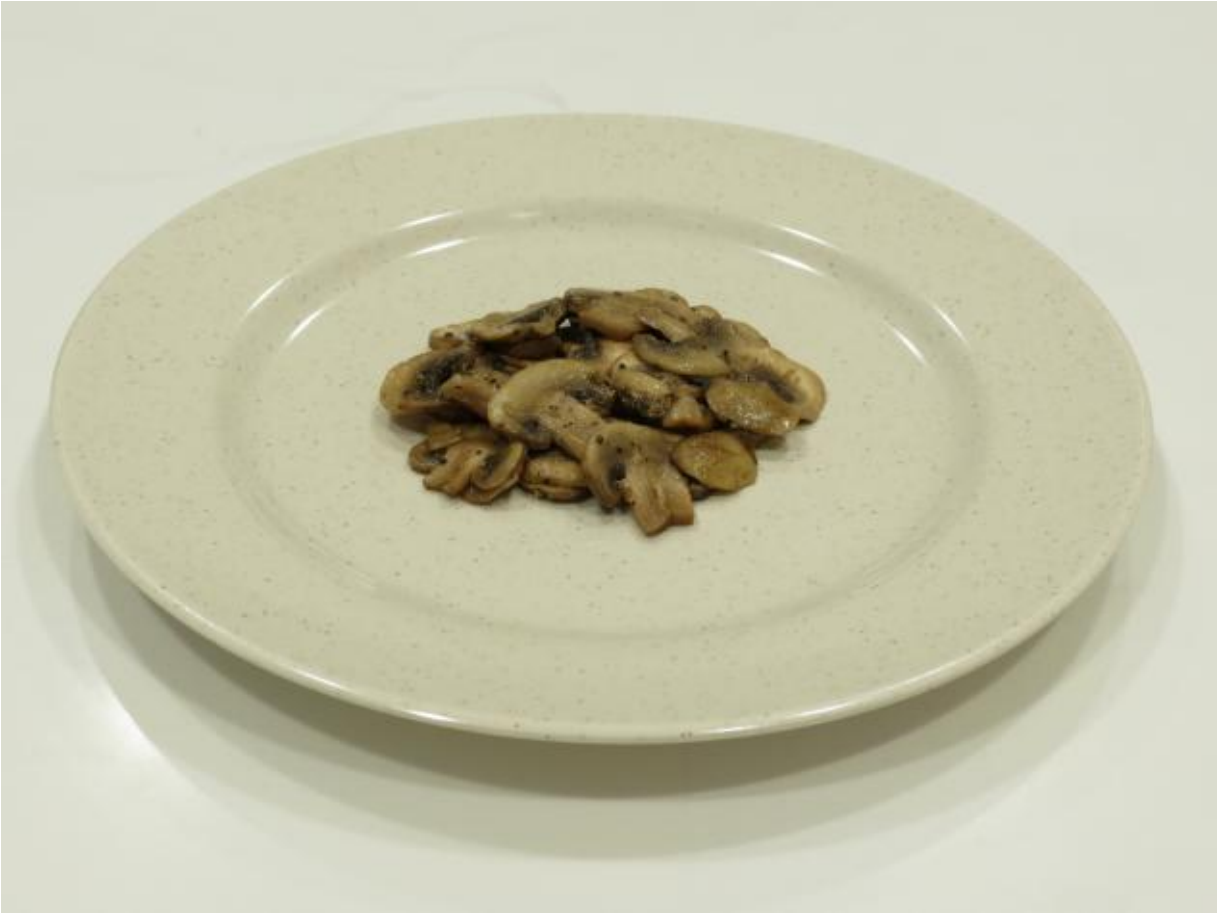

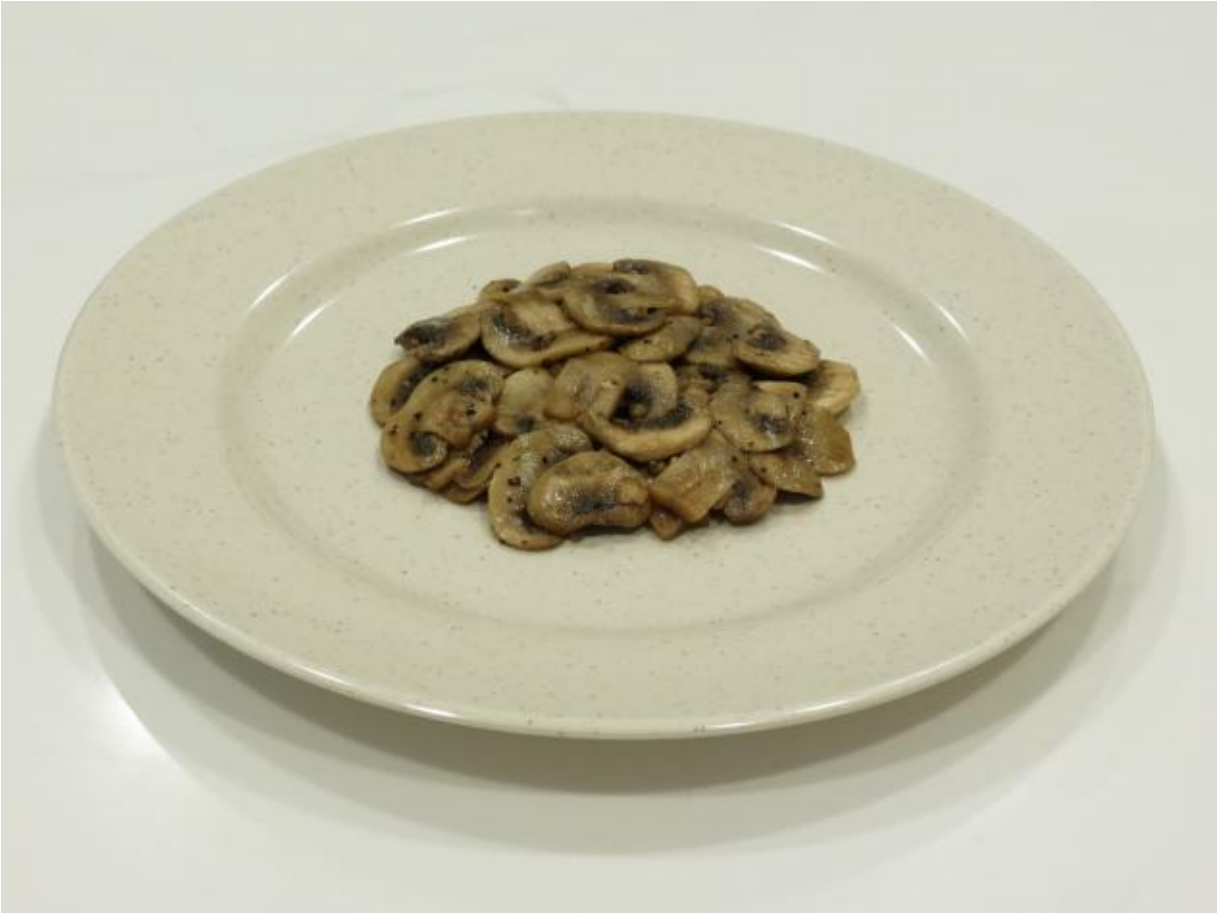

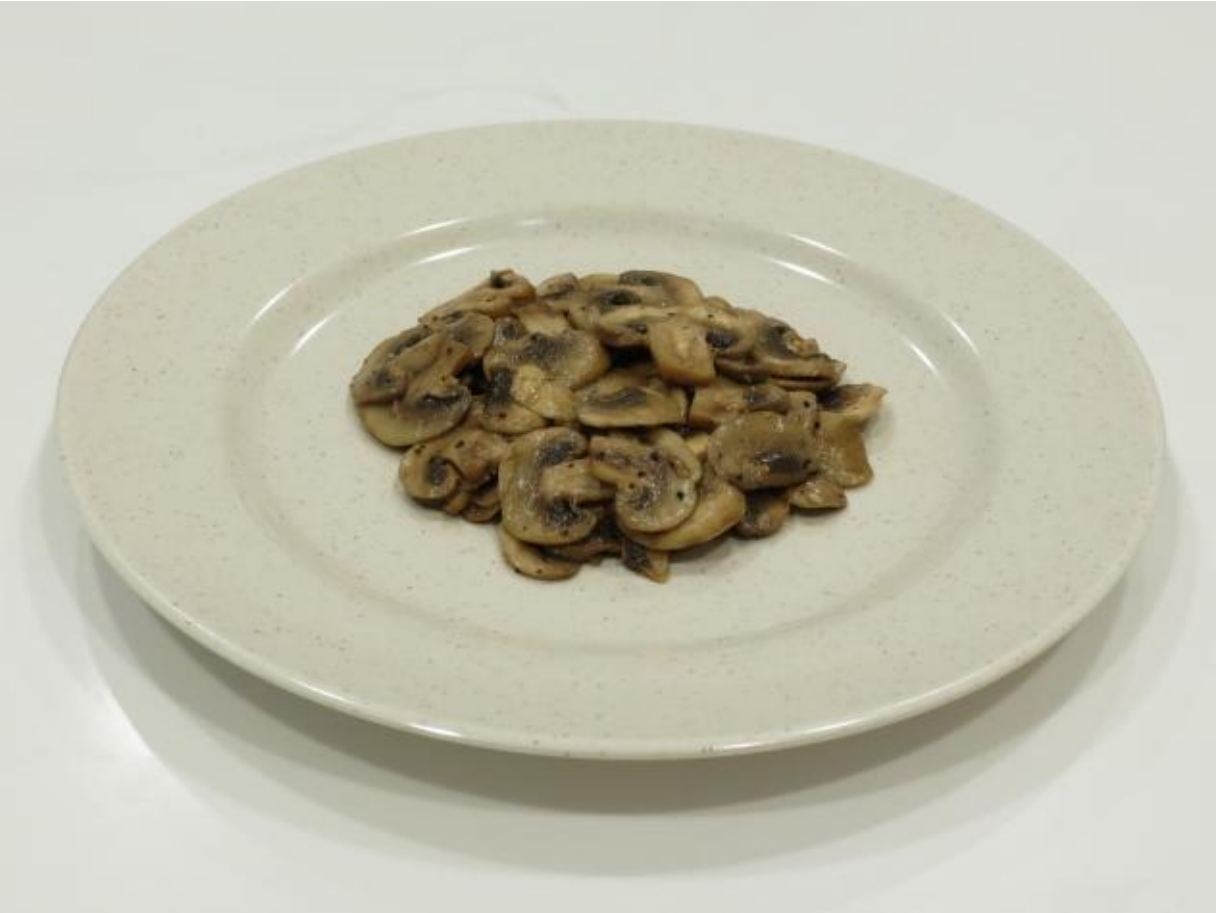

Seaweed snack, dried (FA0007)

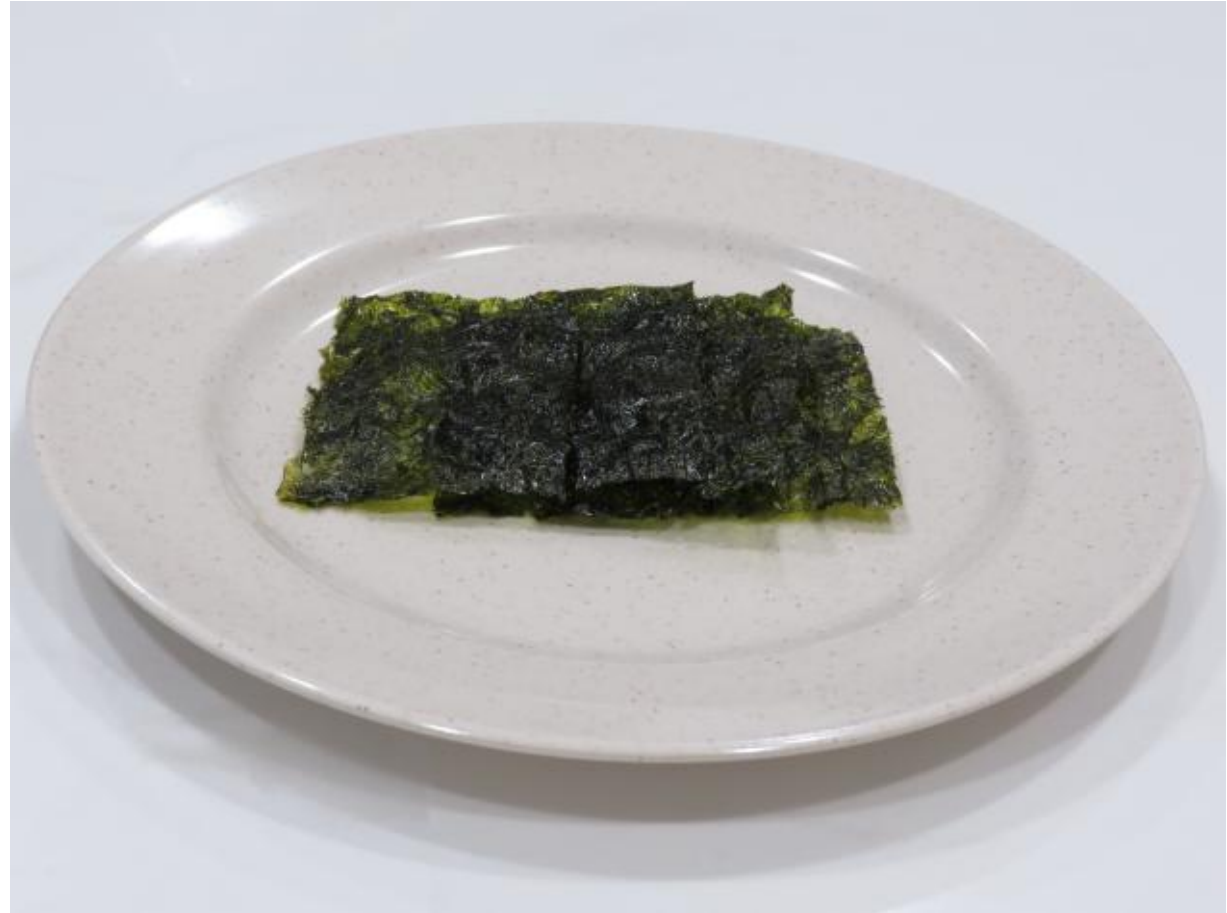
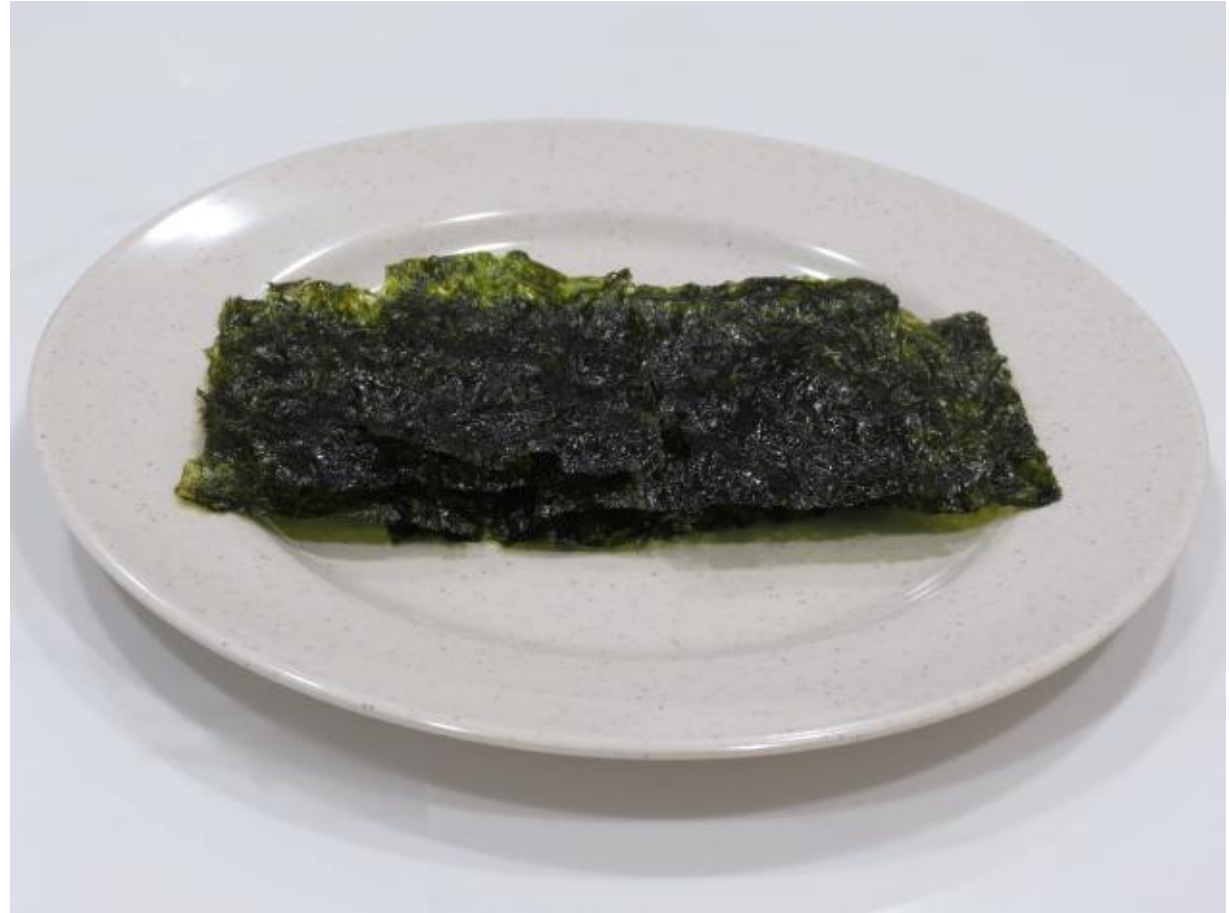
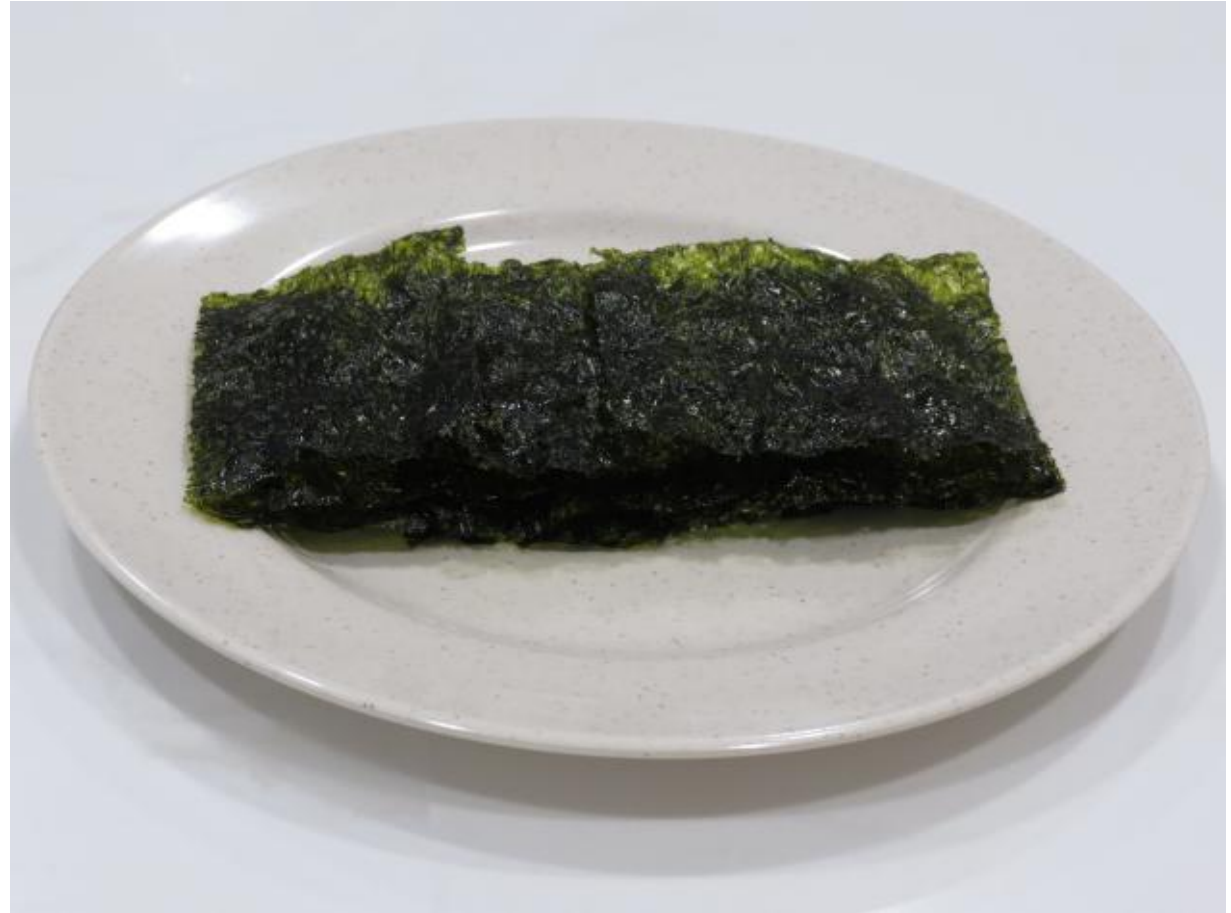
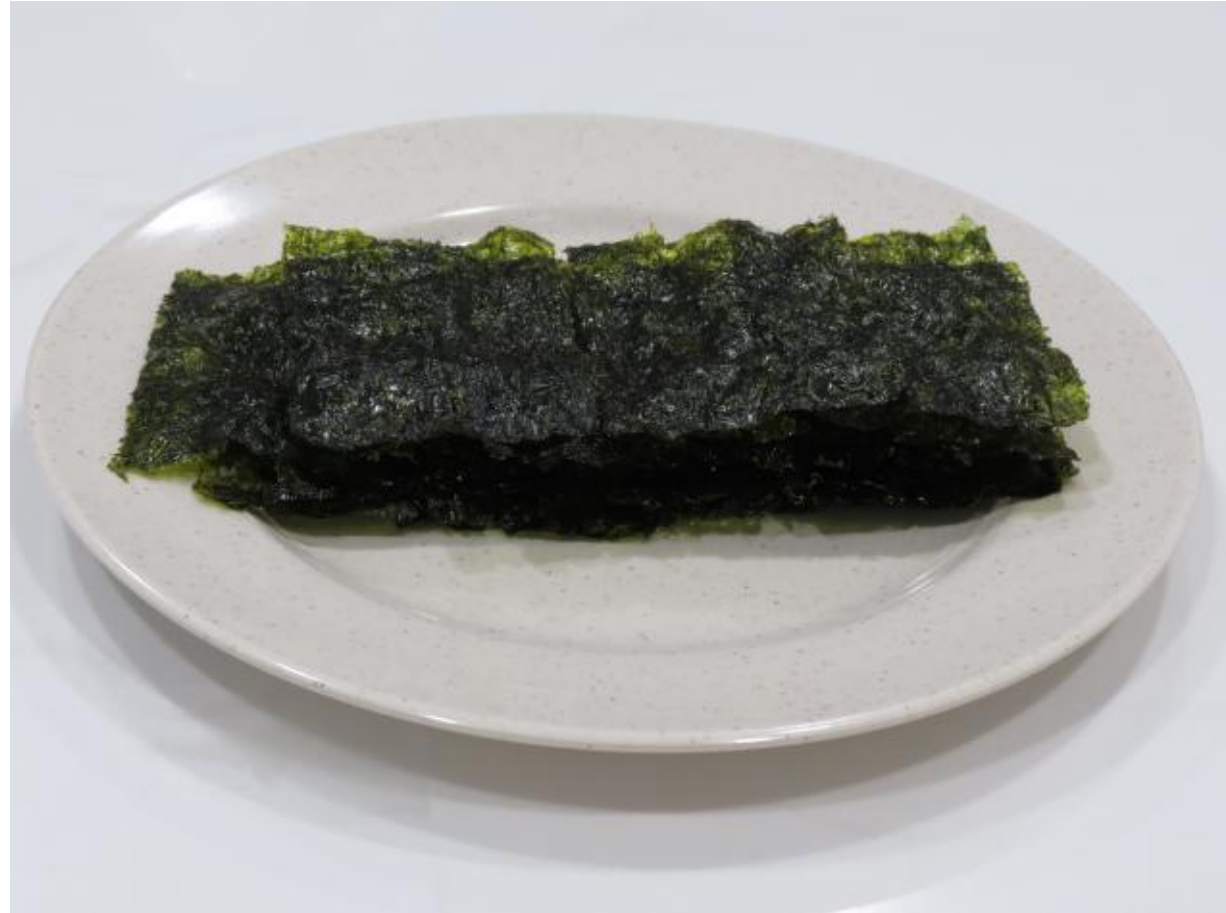

Chee cheong fun (猪肠粉) (FA0065)

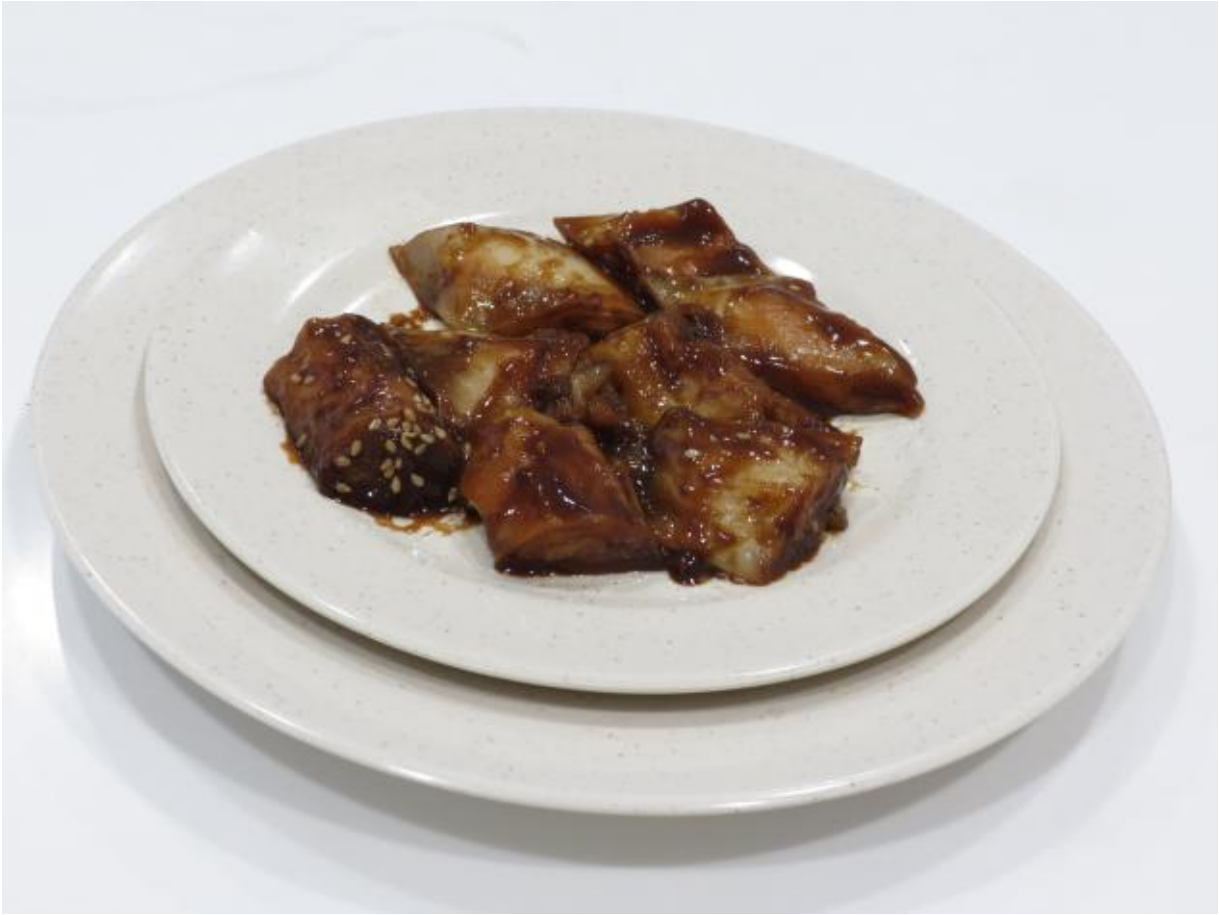 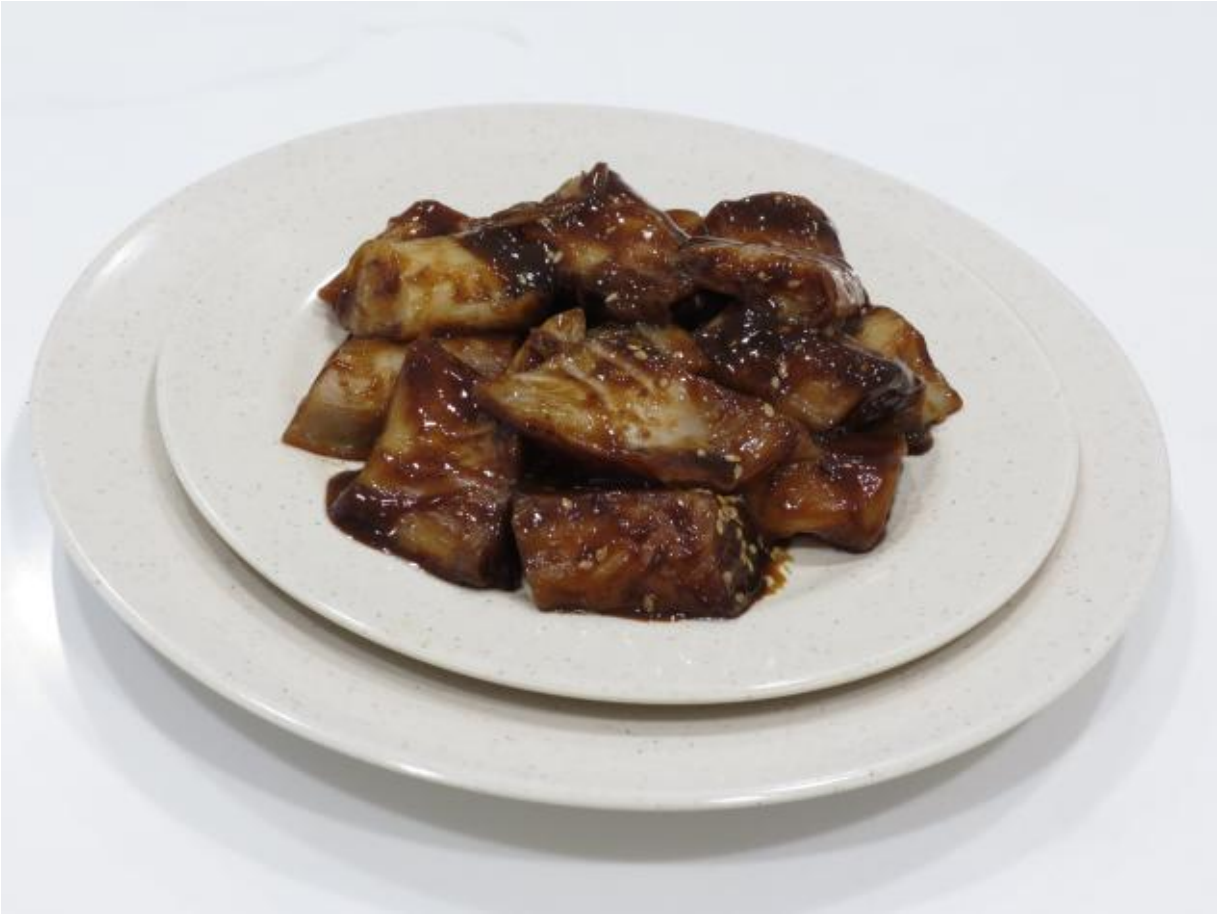

Congee (FA0095)

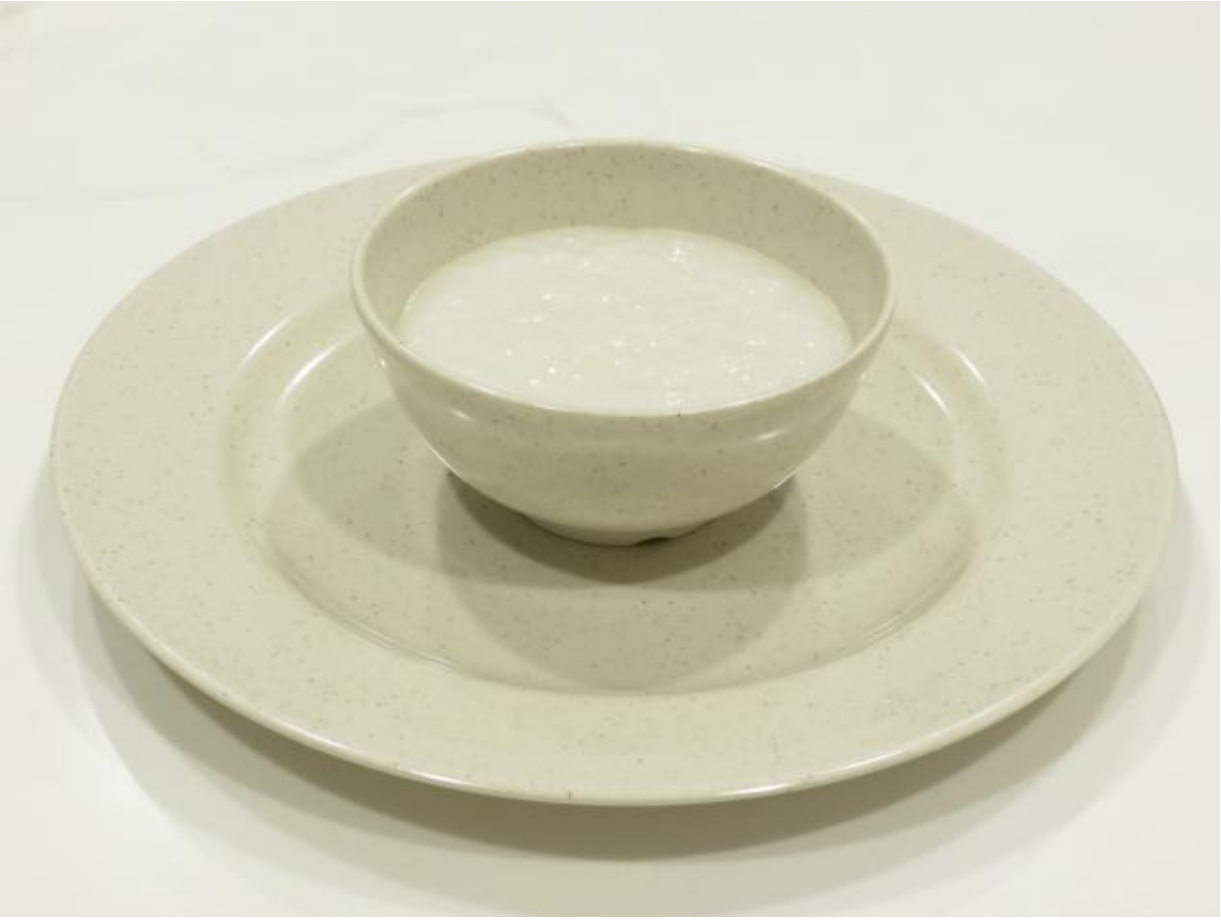

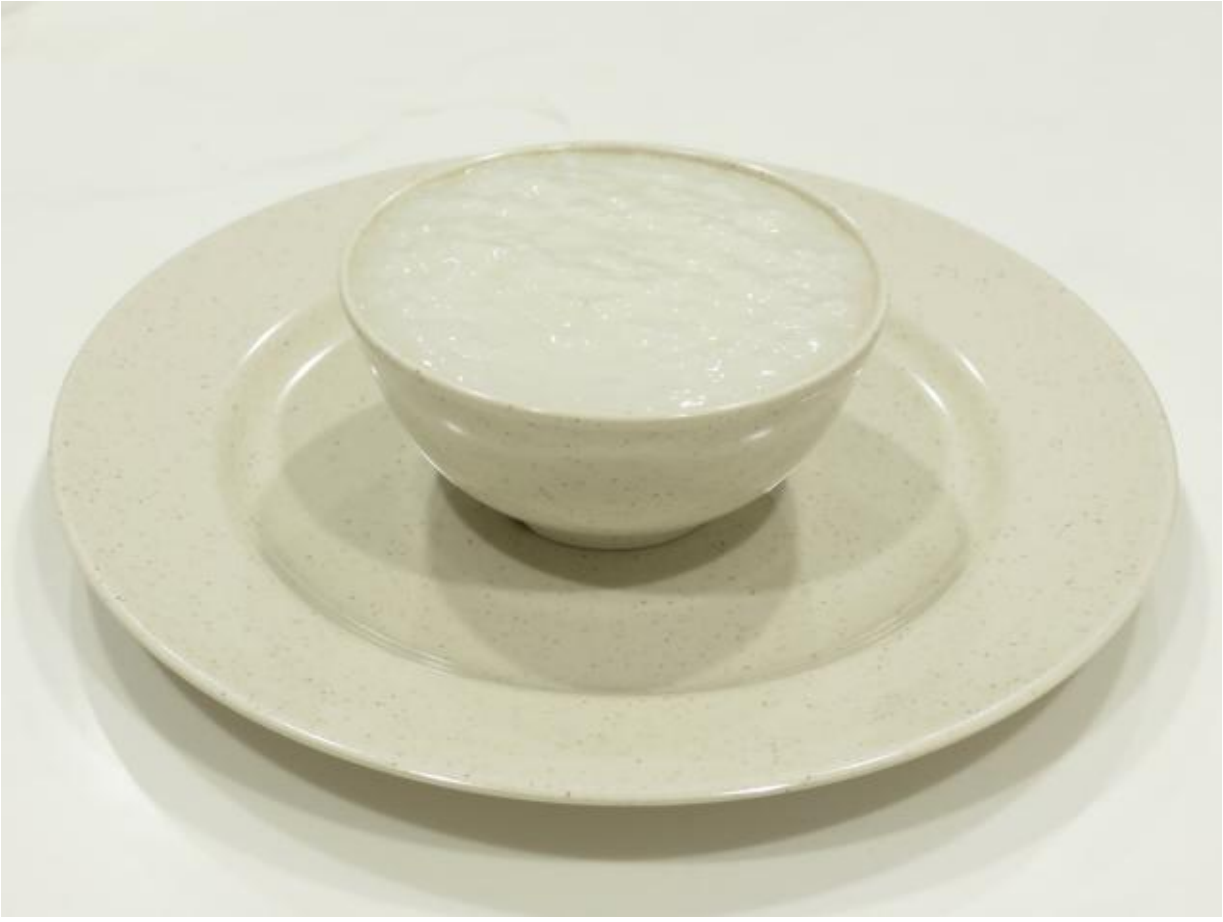

Corn, kernel (FA0074)

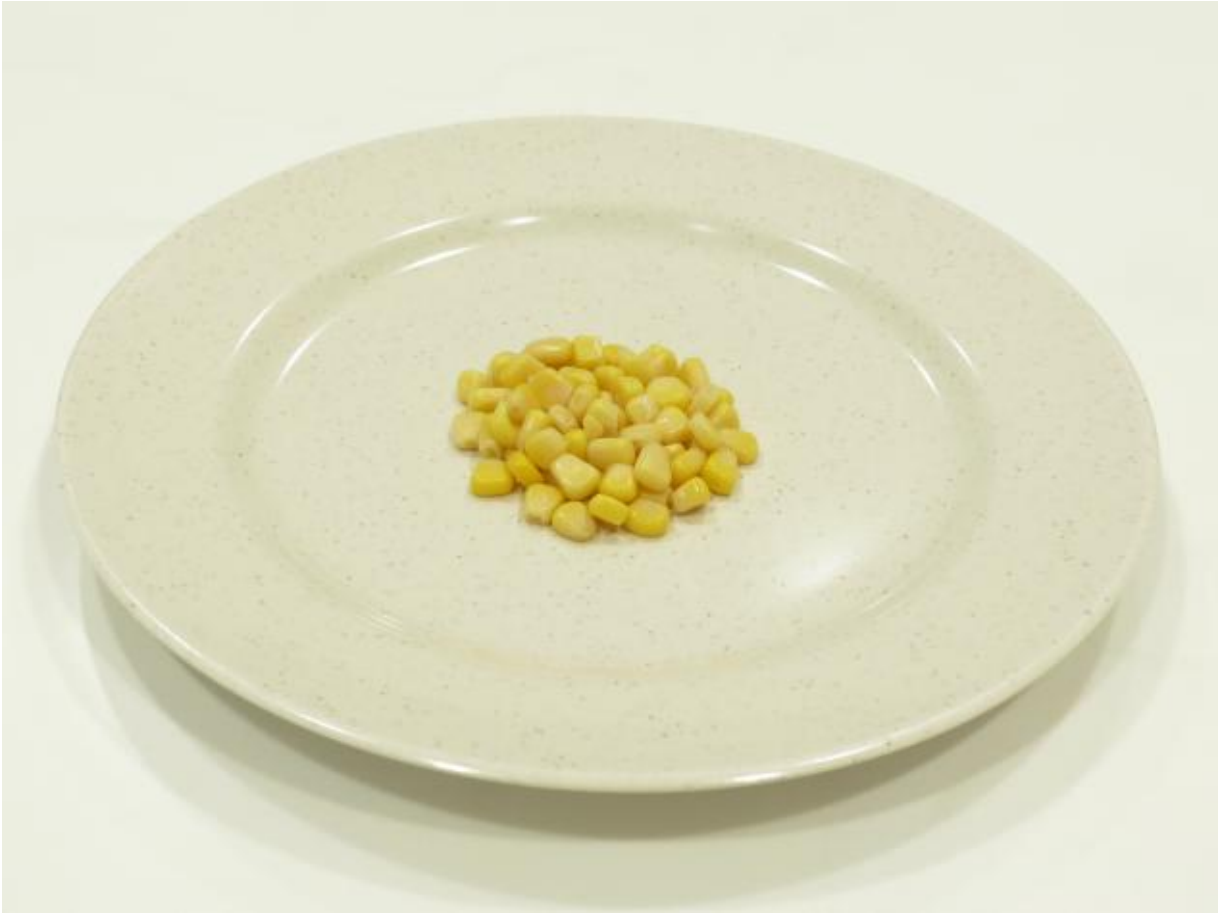
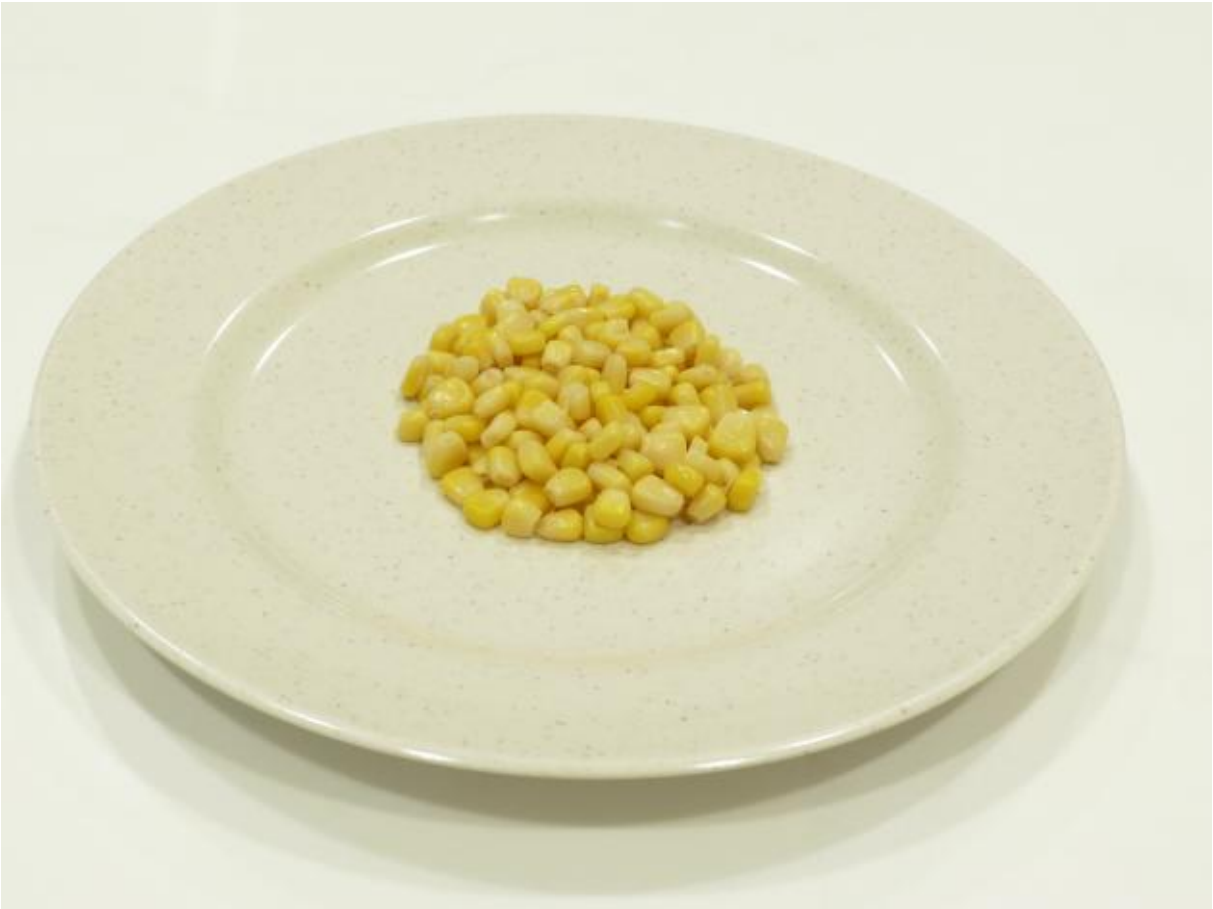
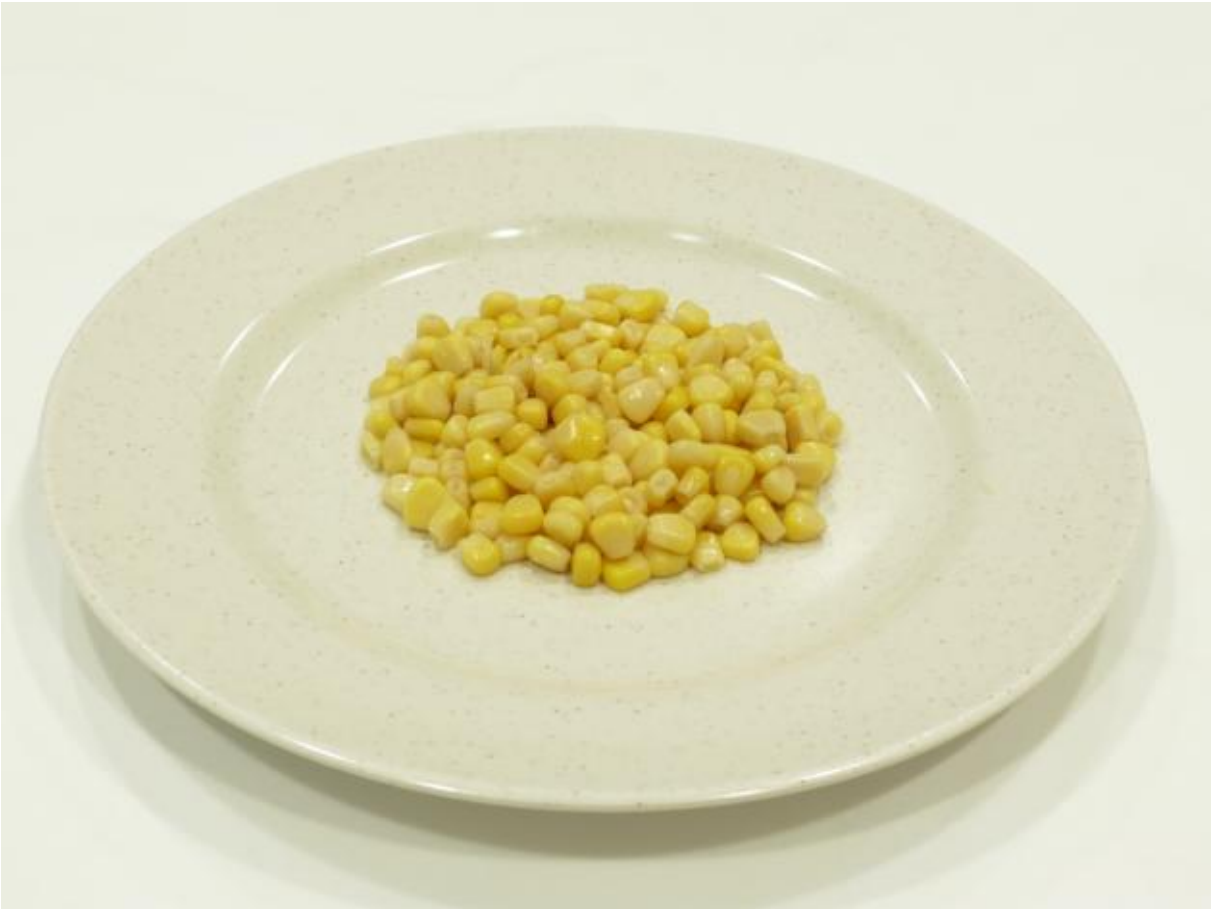
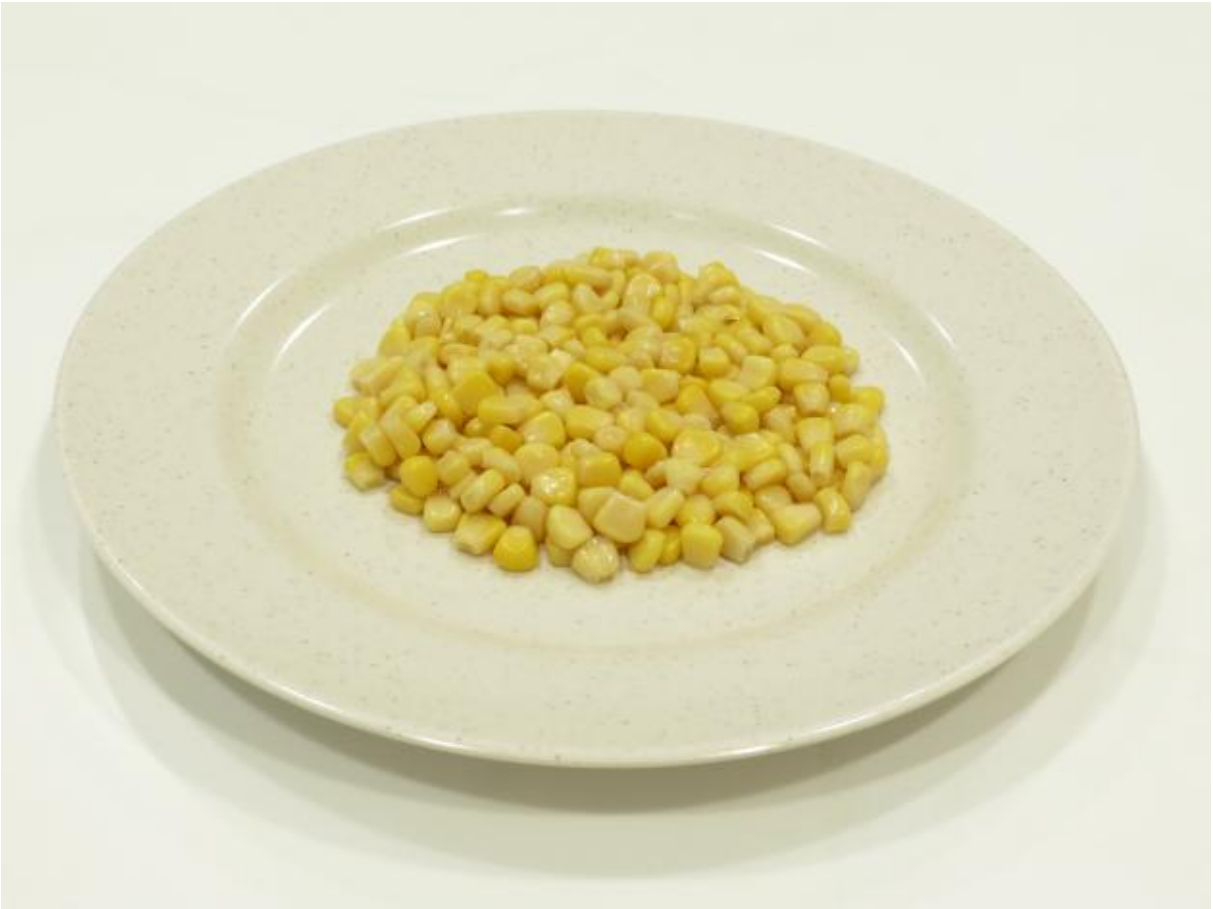

Noodle, egg (FA0092)

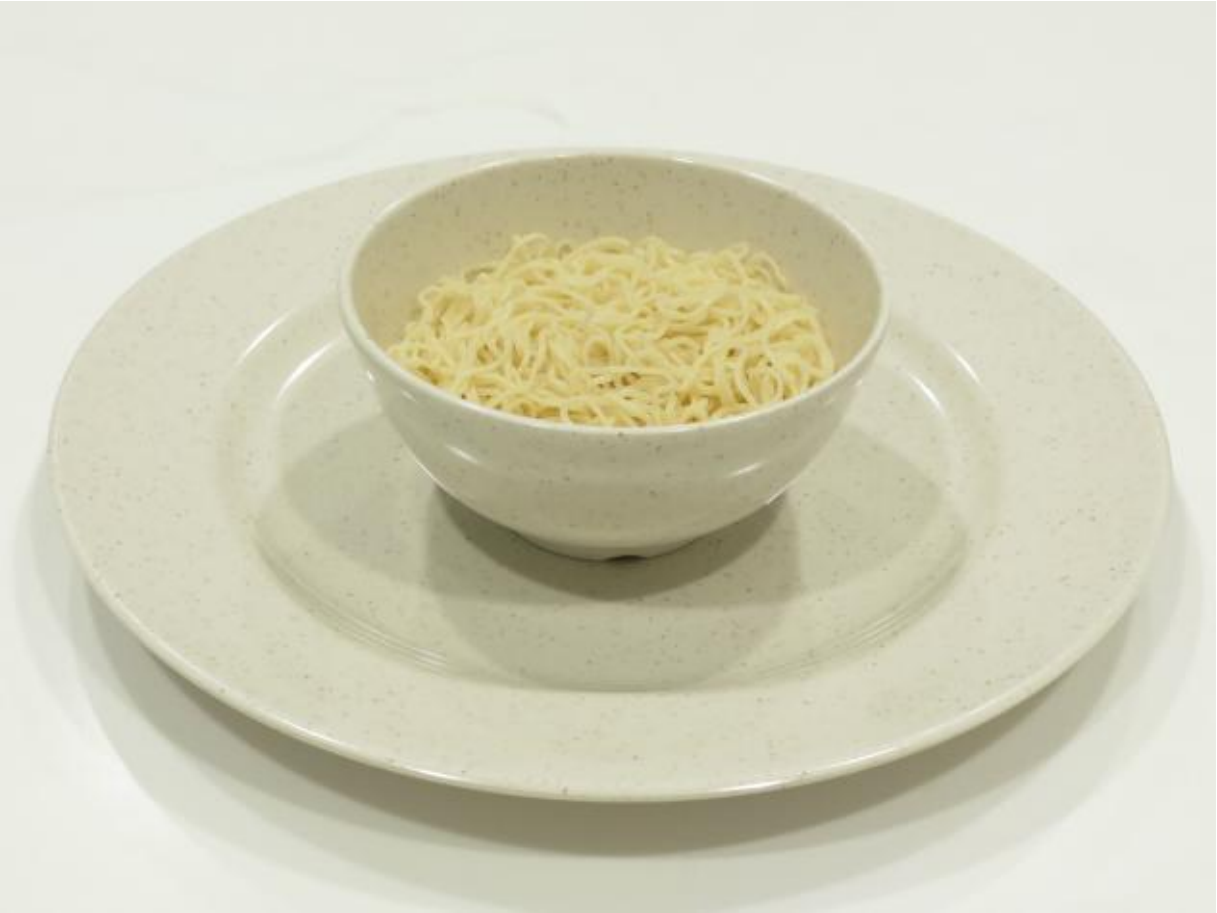

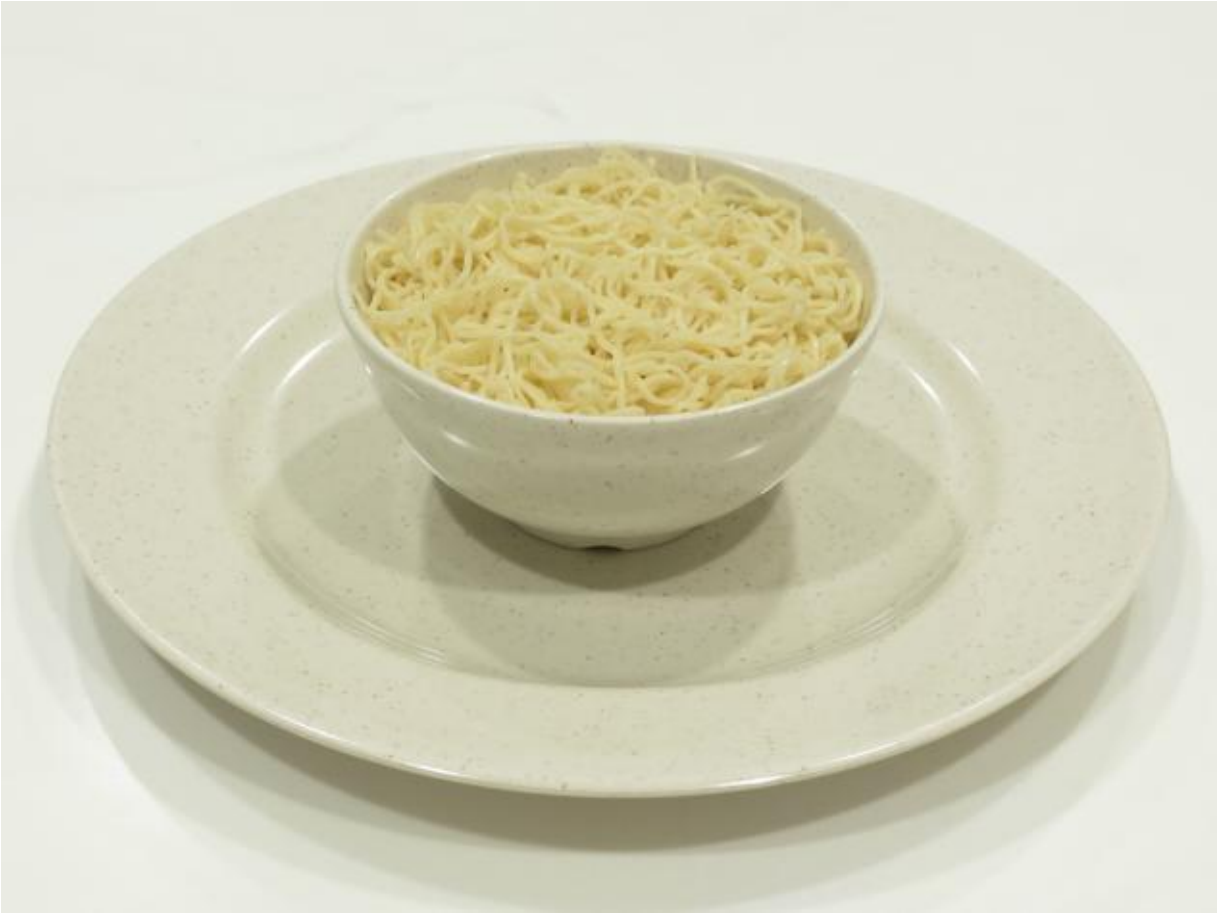

Noodle, thick (FA0071)

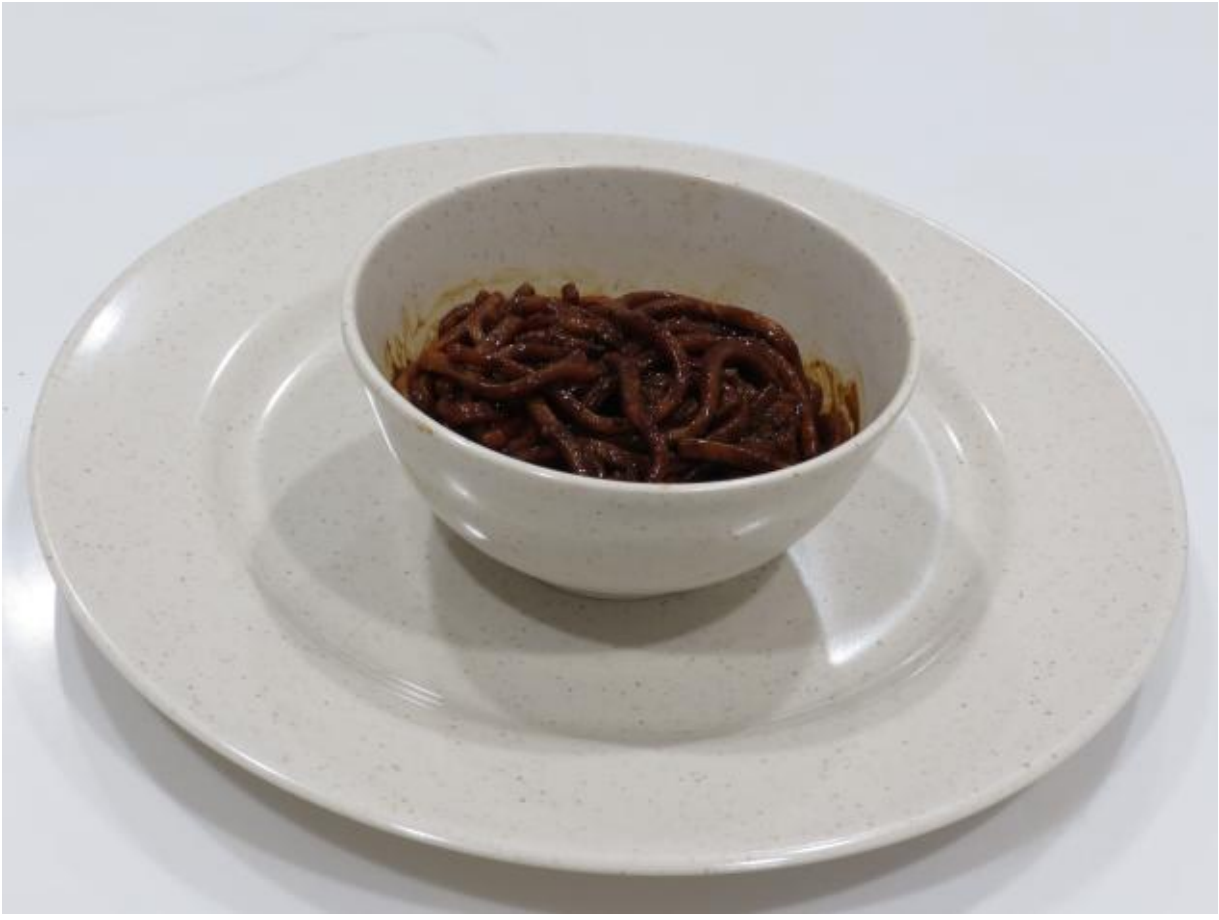

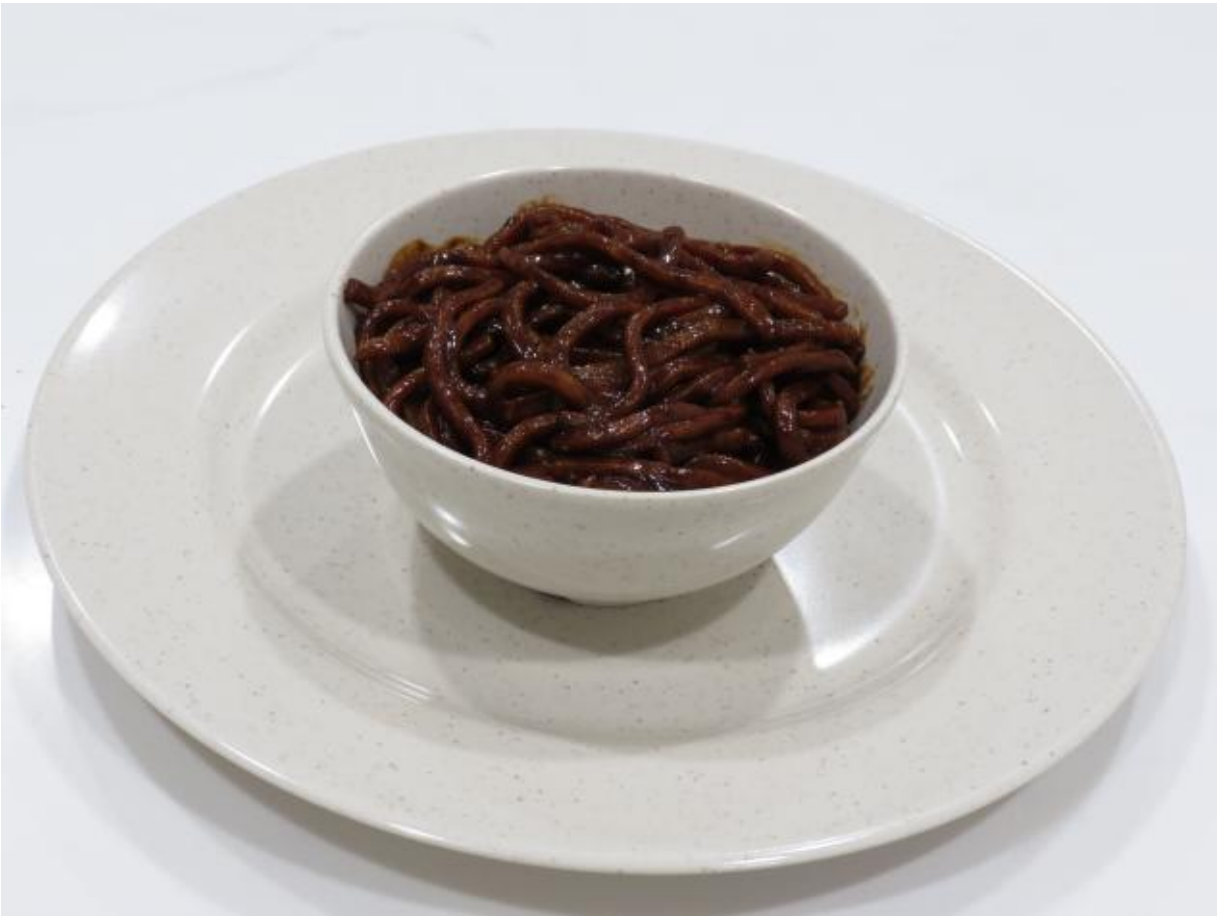

Pasta, fusilli (FA0181)

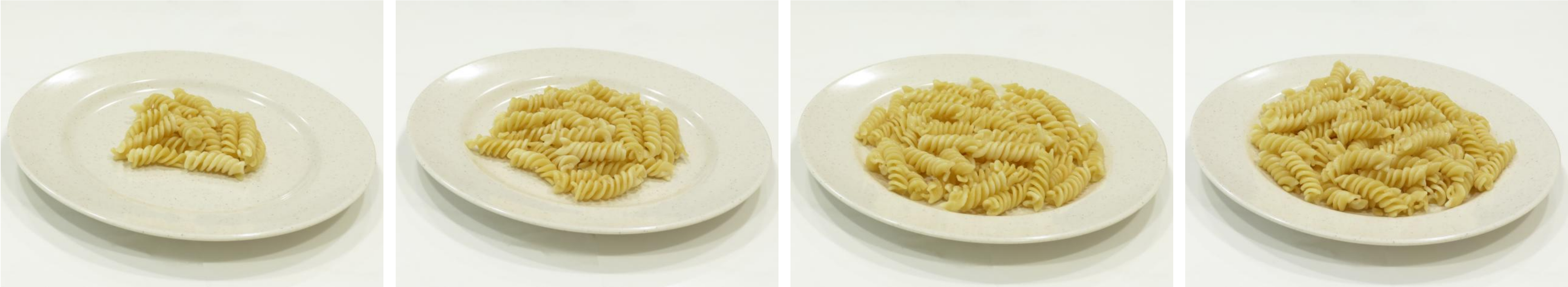

Pasta, macaroni (FA0066)

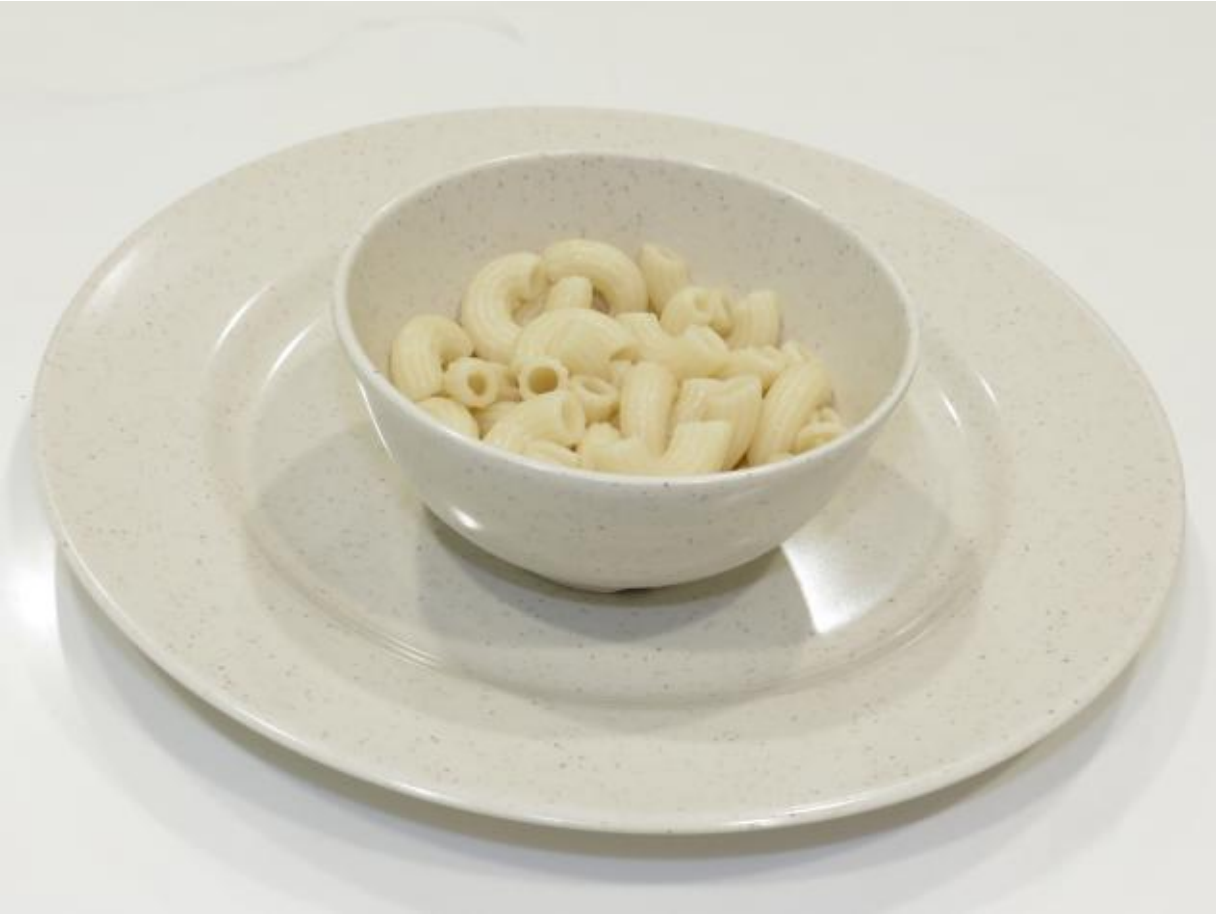

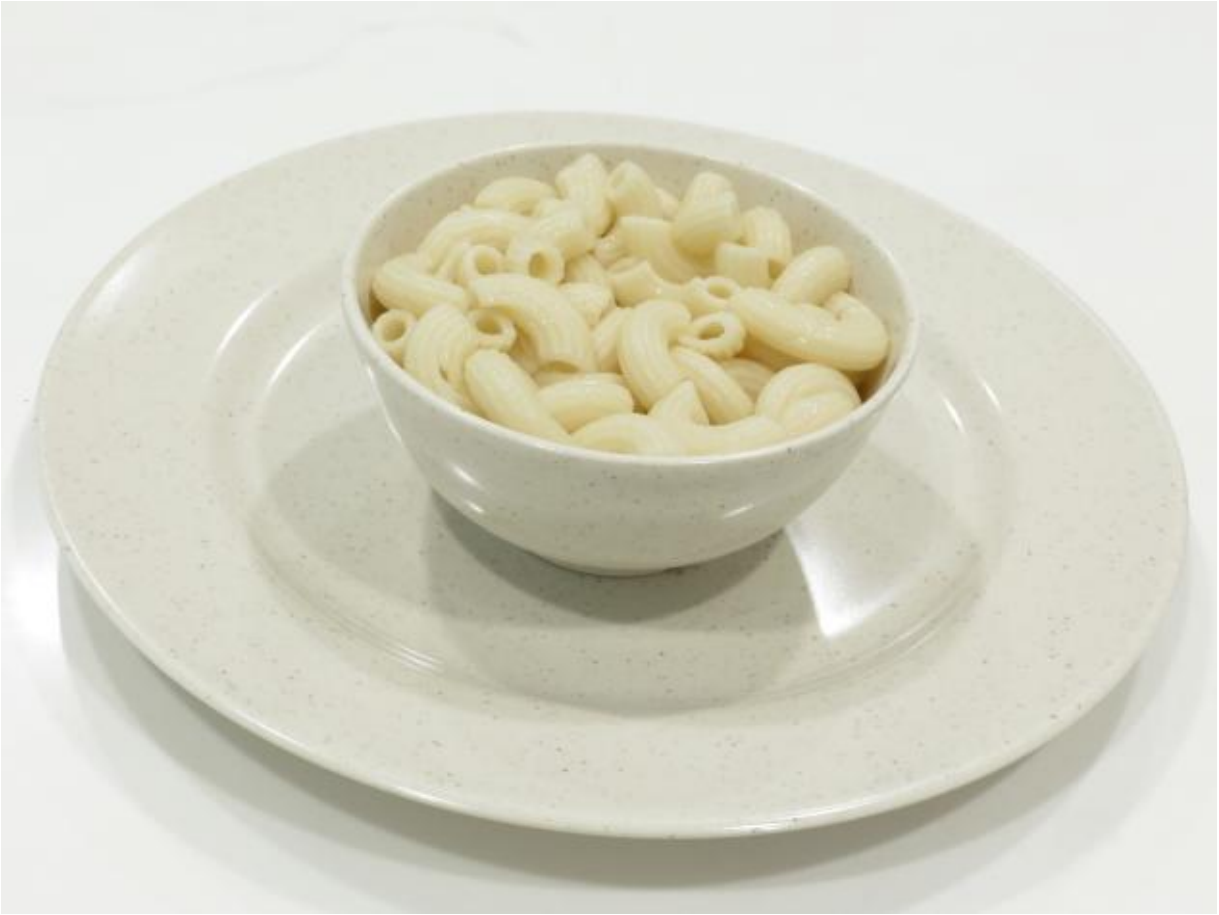

Pasta, penne (FA0180)

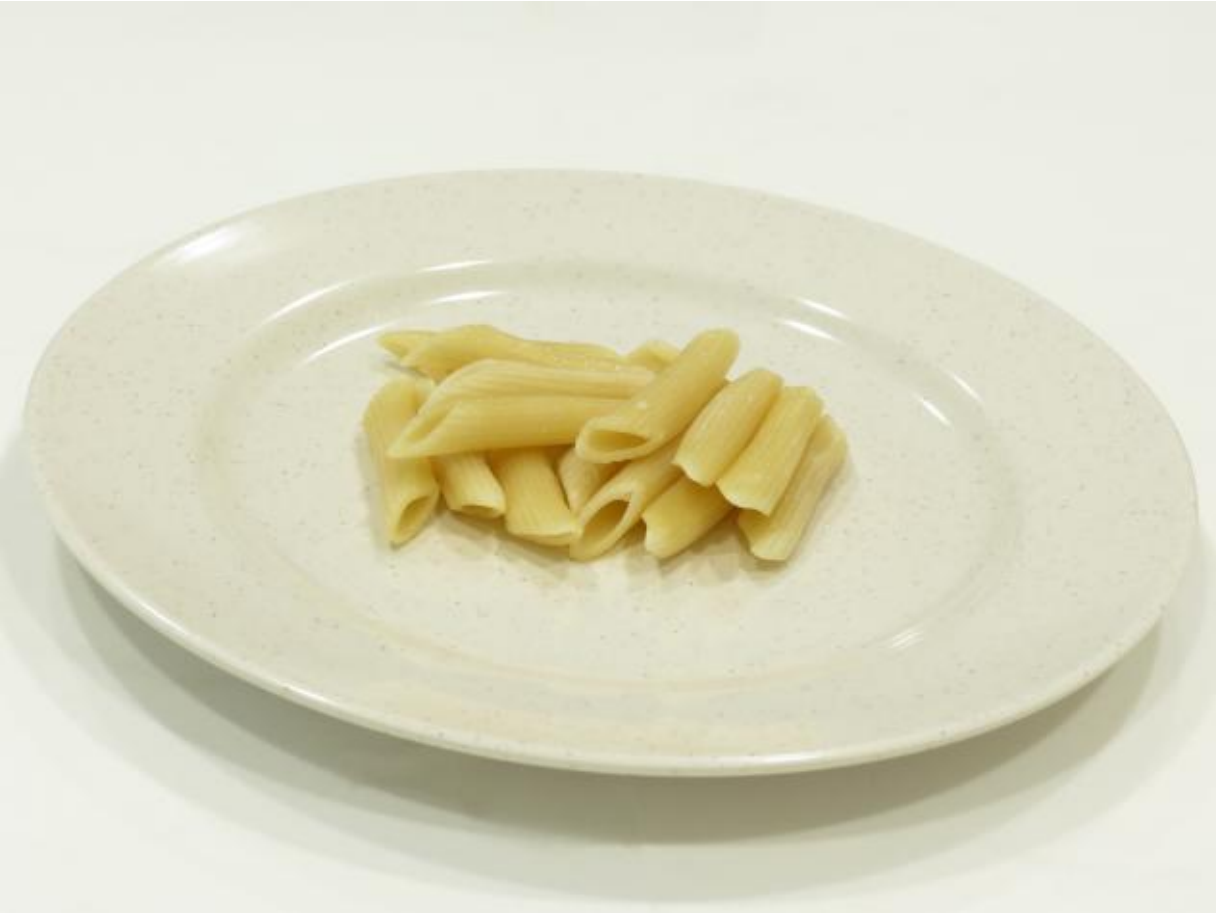

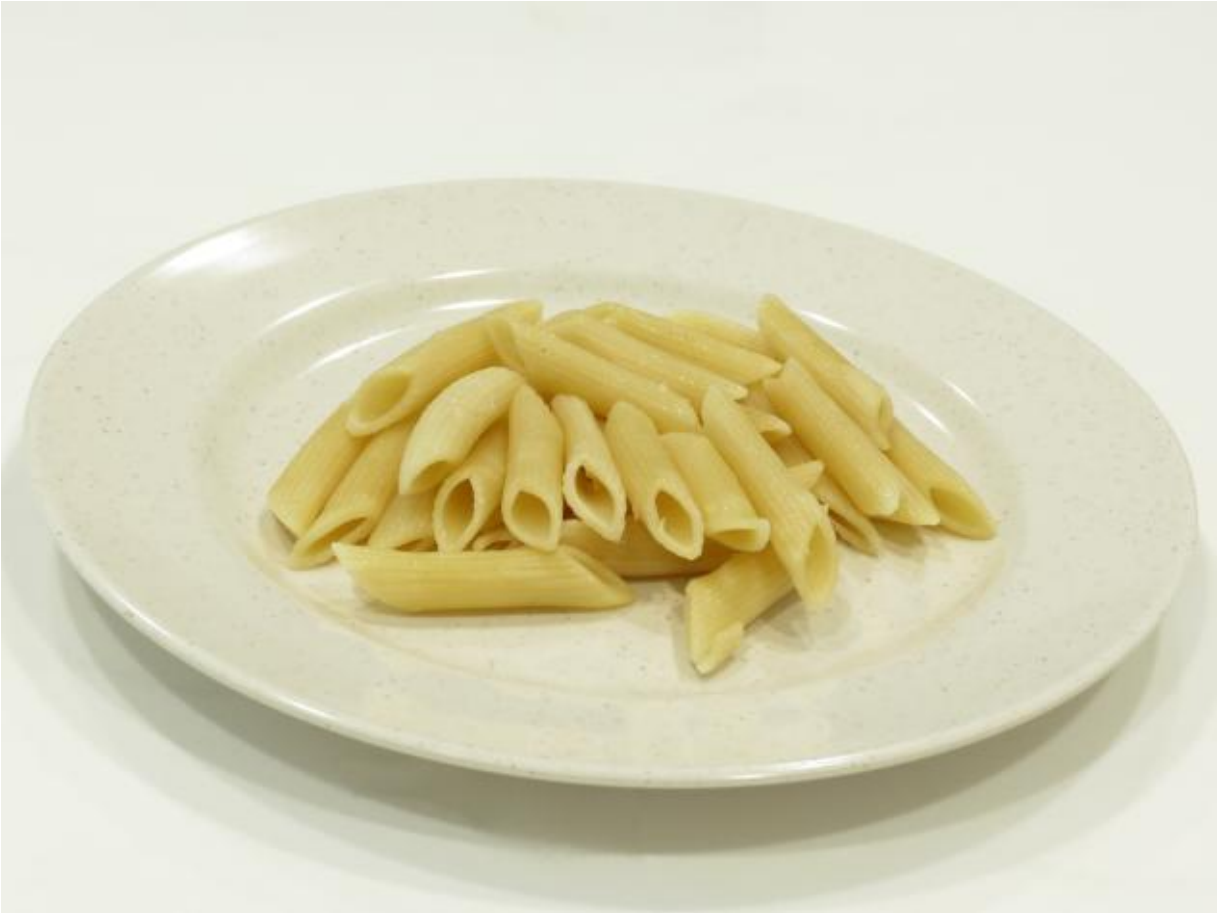

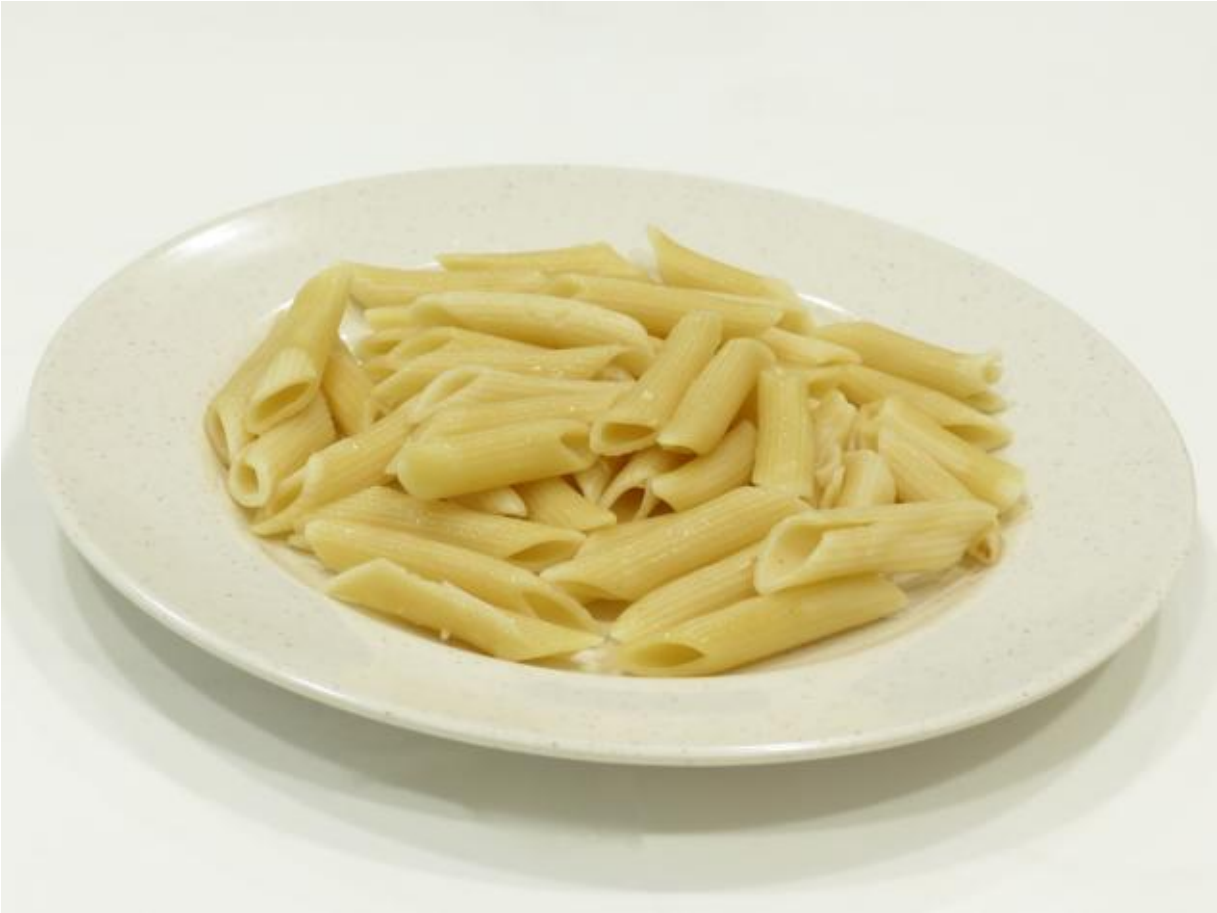

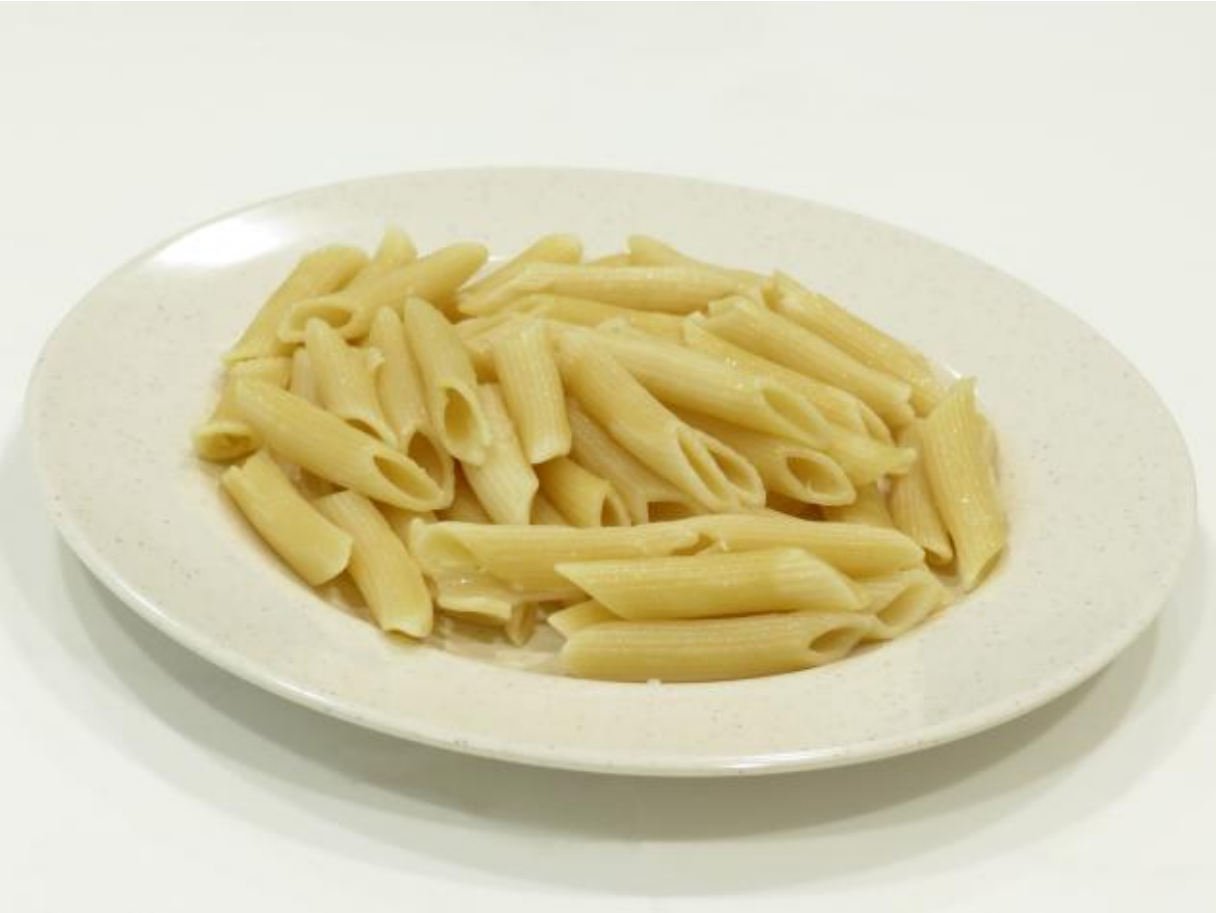

Pasta, spaghetti (FA0169)

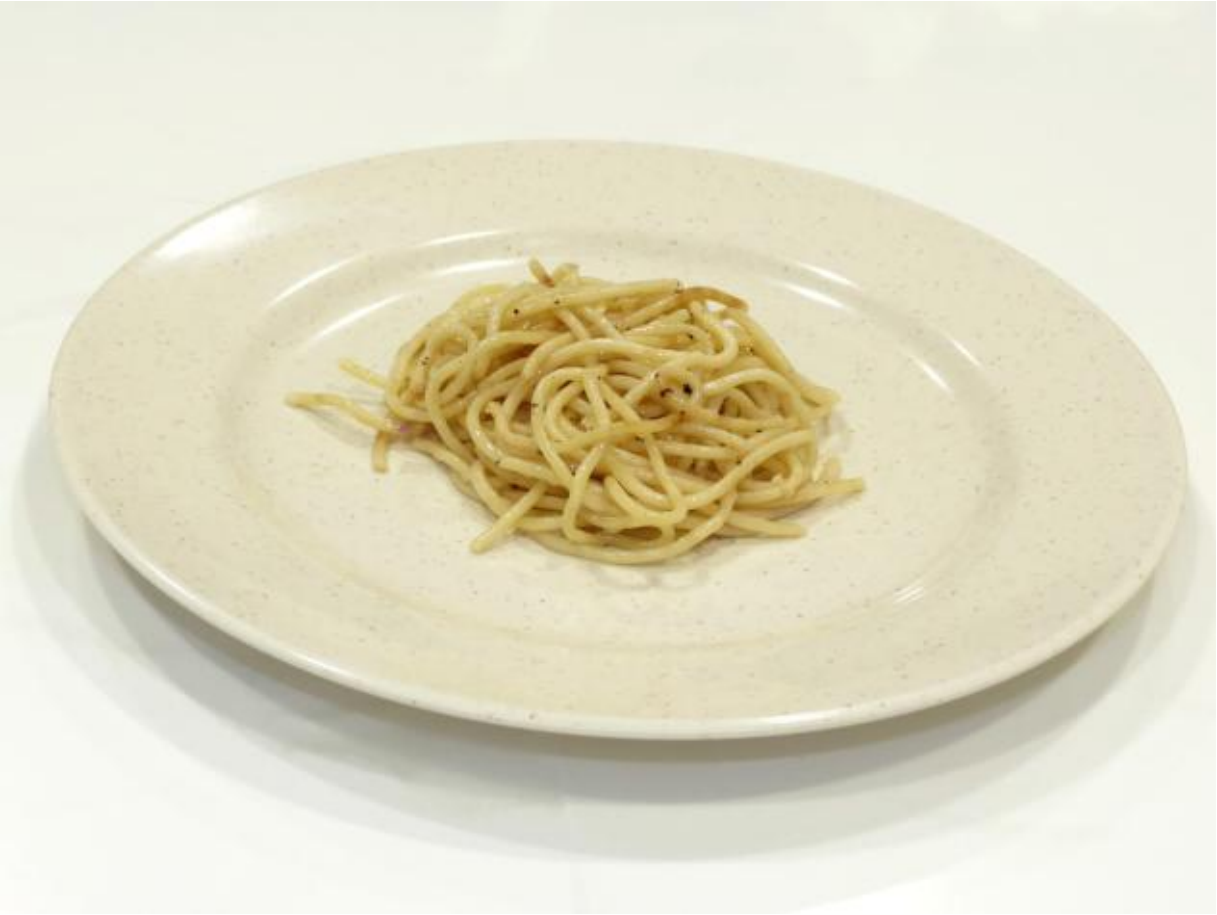

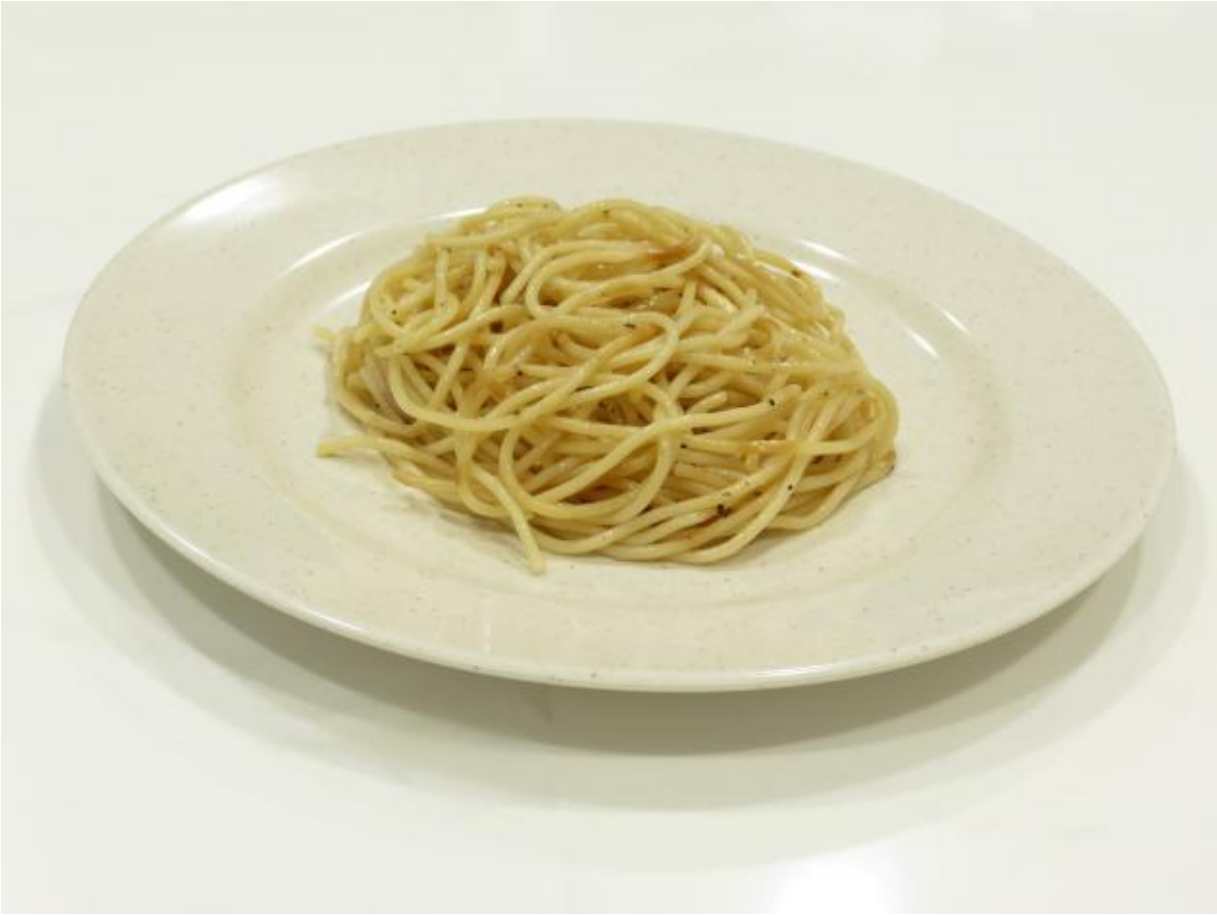

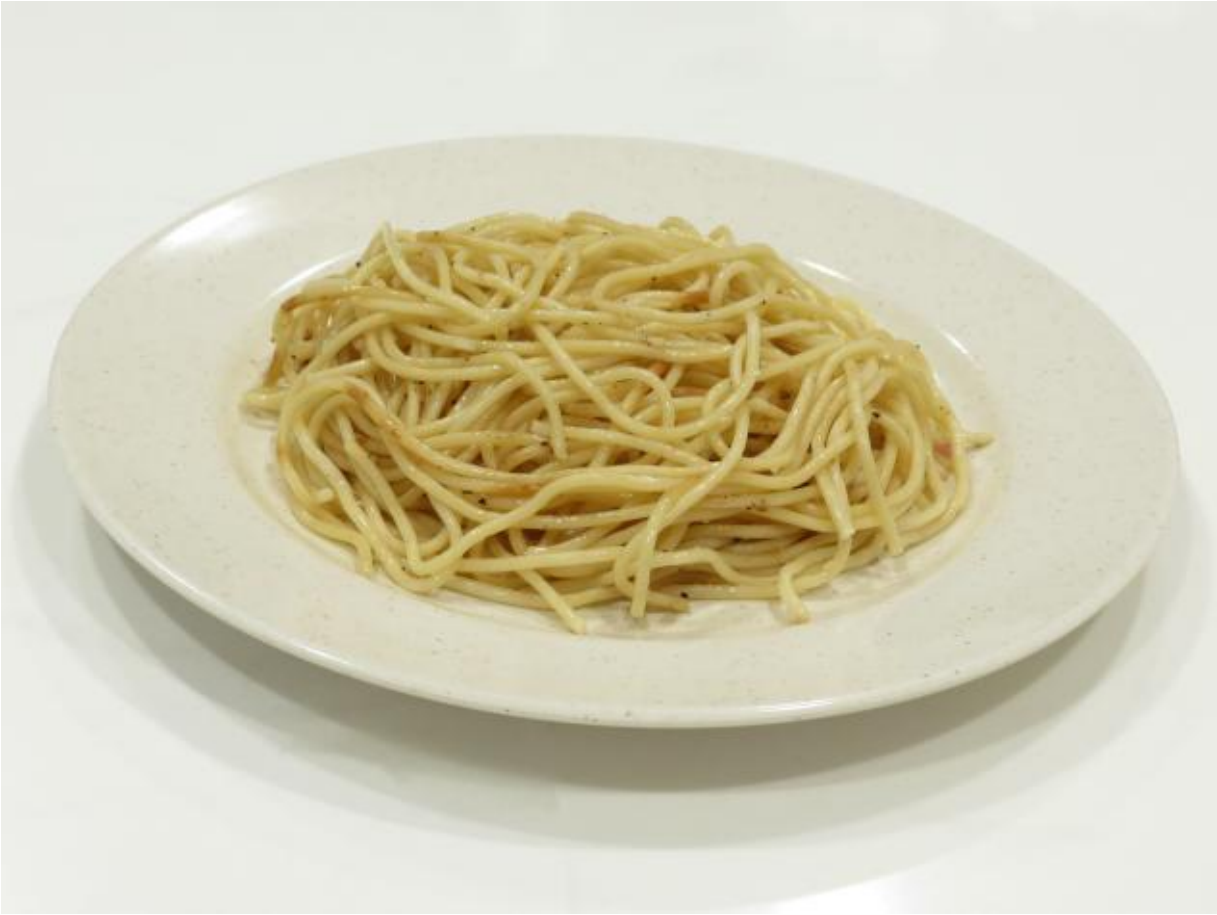

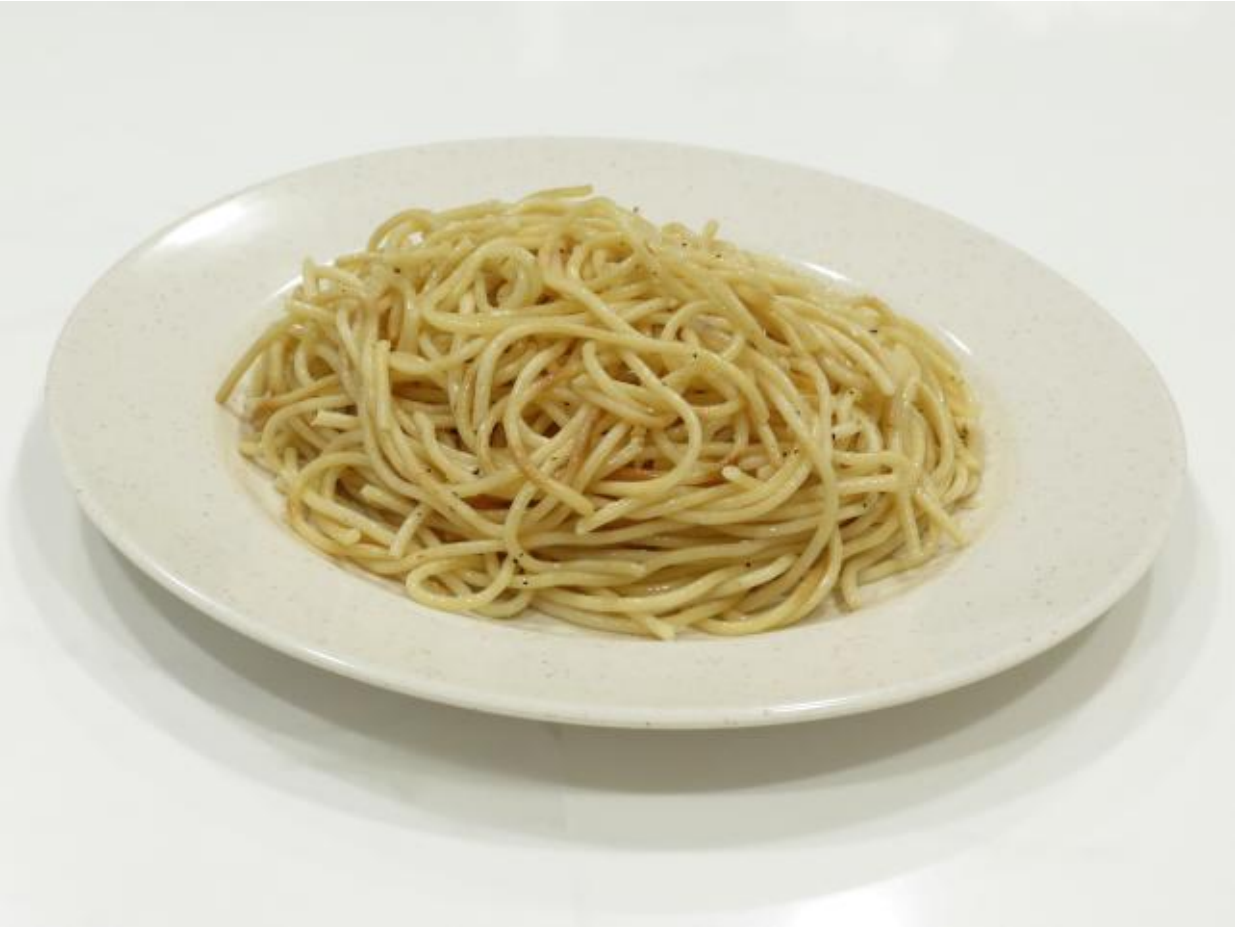

Porridge (FA0194)

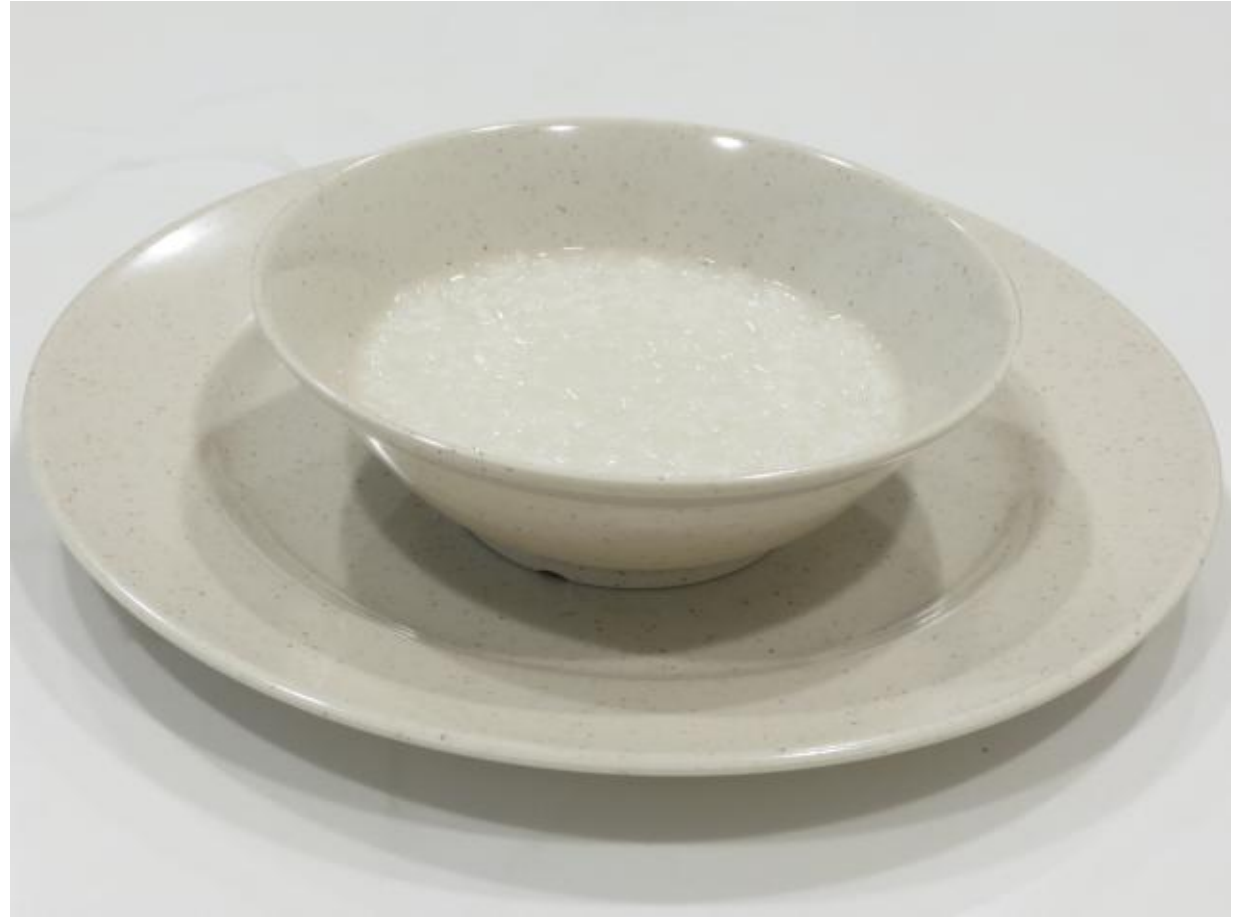
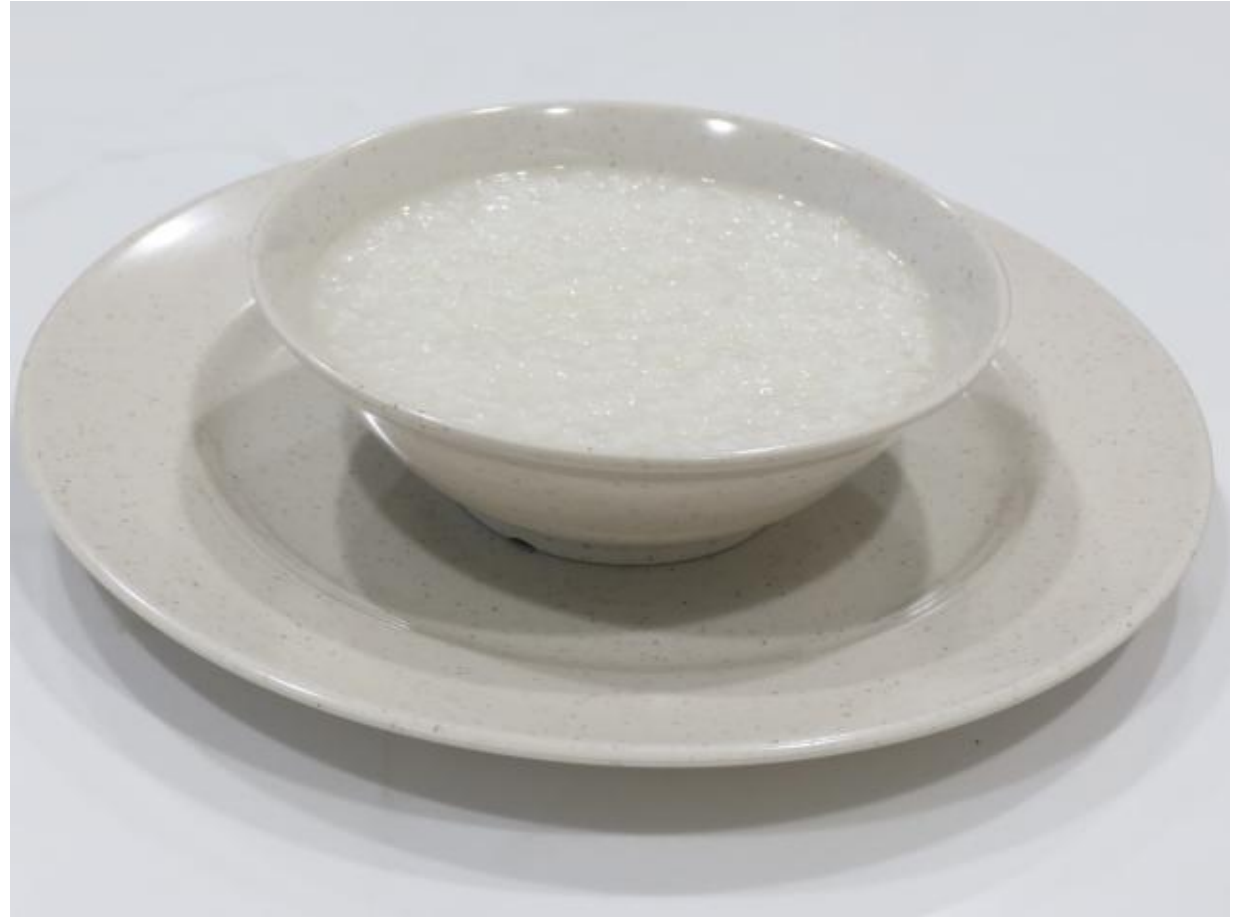
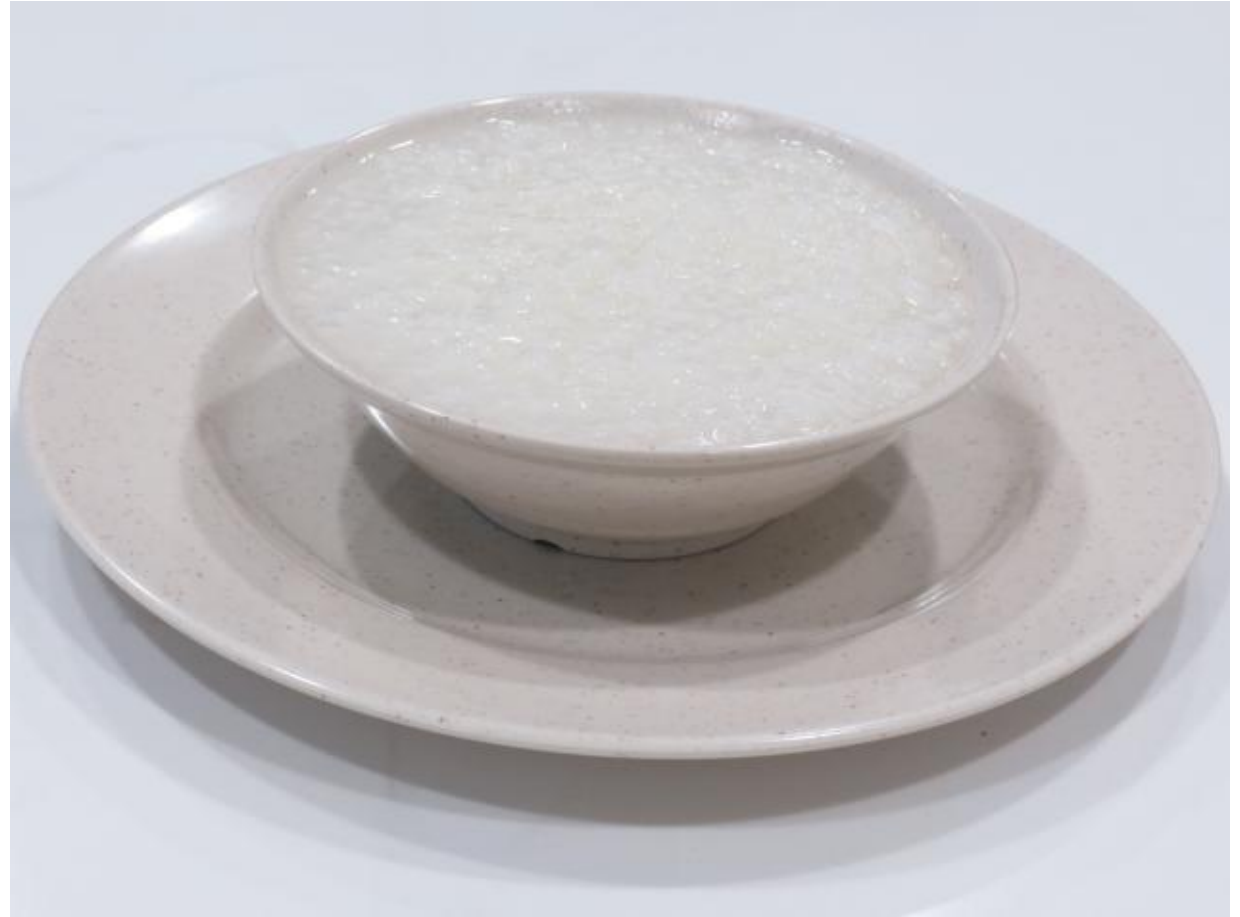
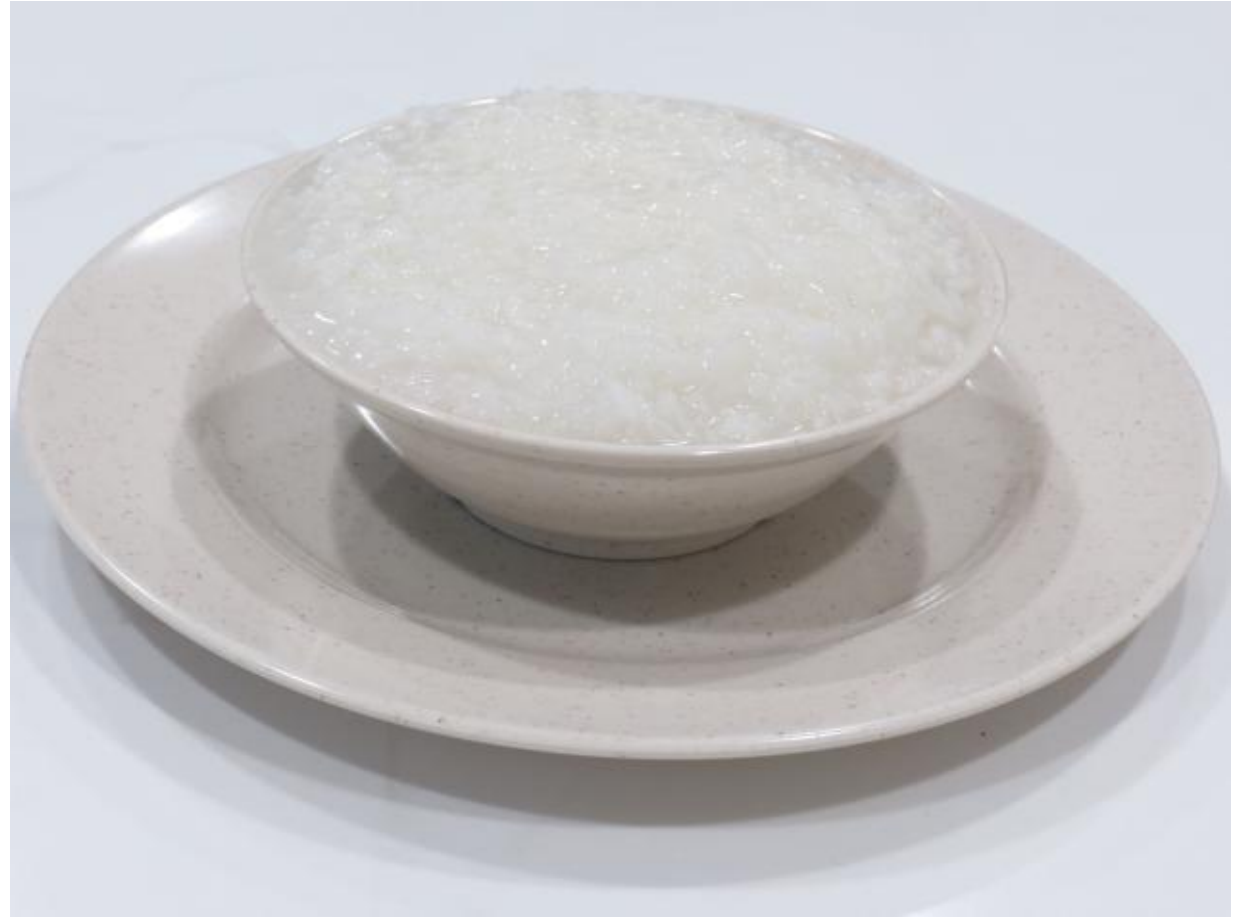

Rice noodle, bee hoon, thin, fried (FA0059)

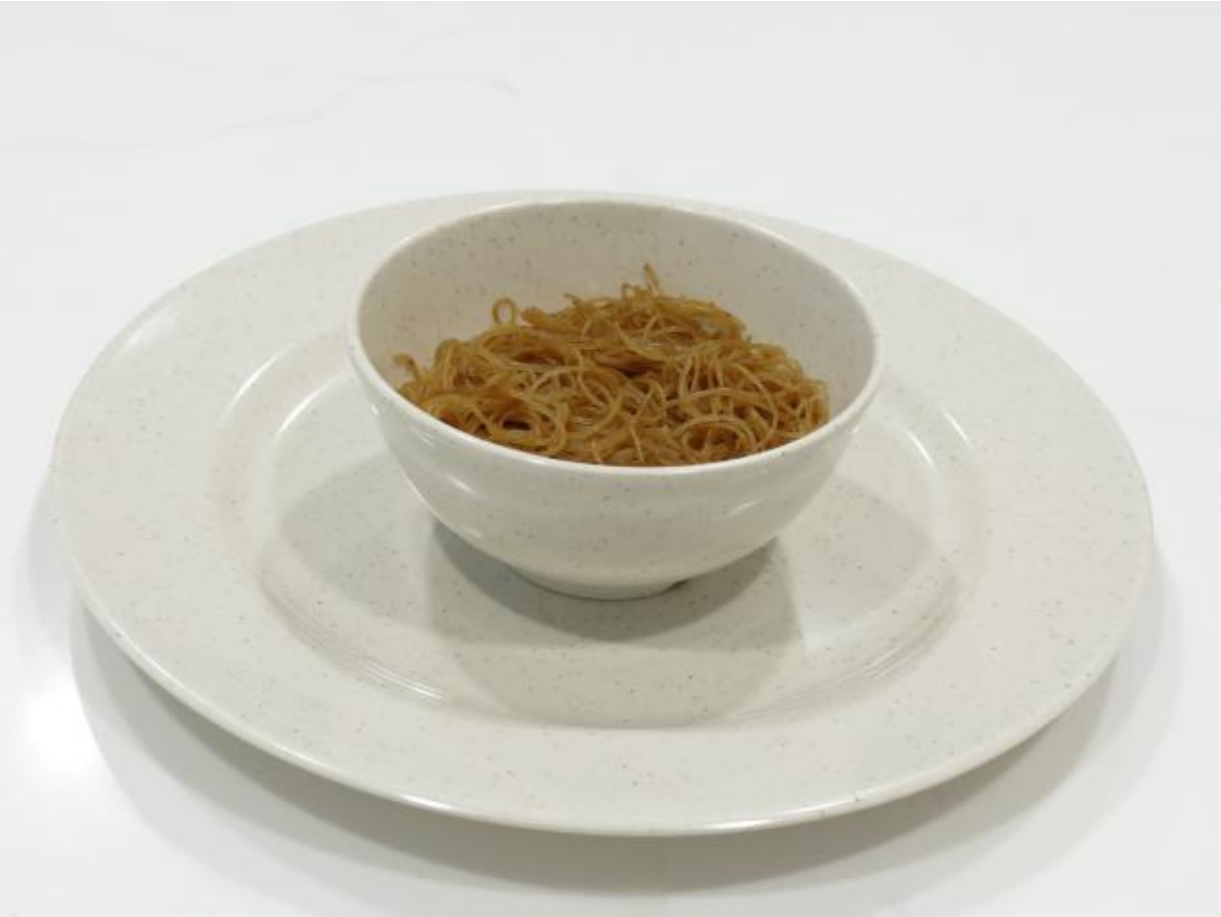

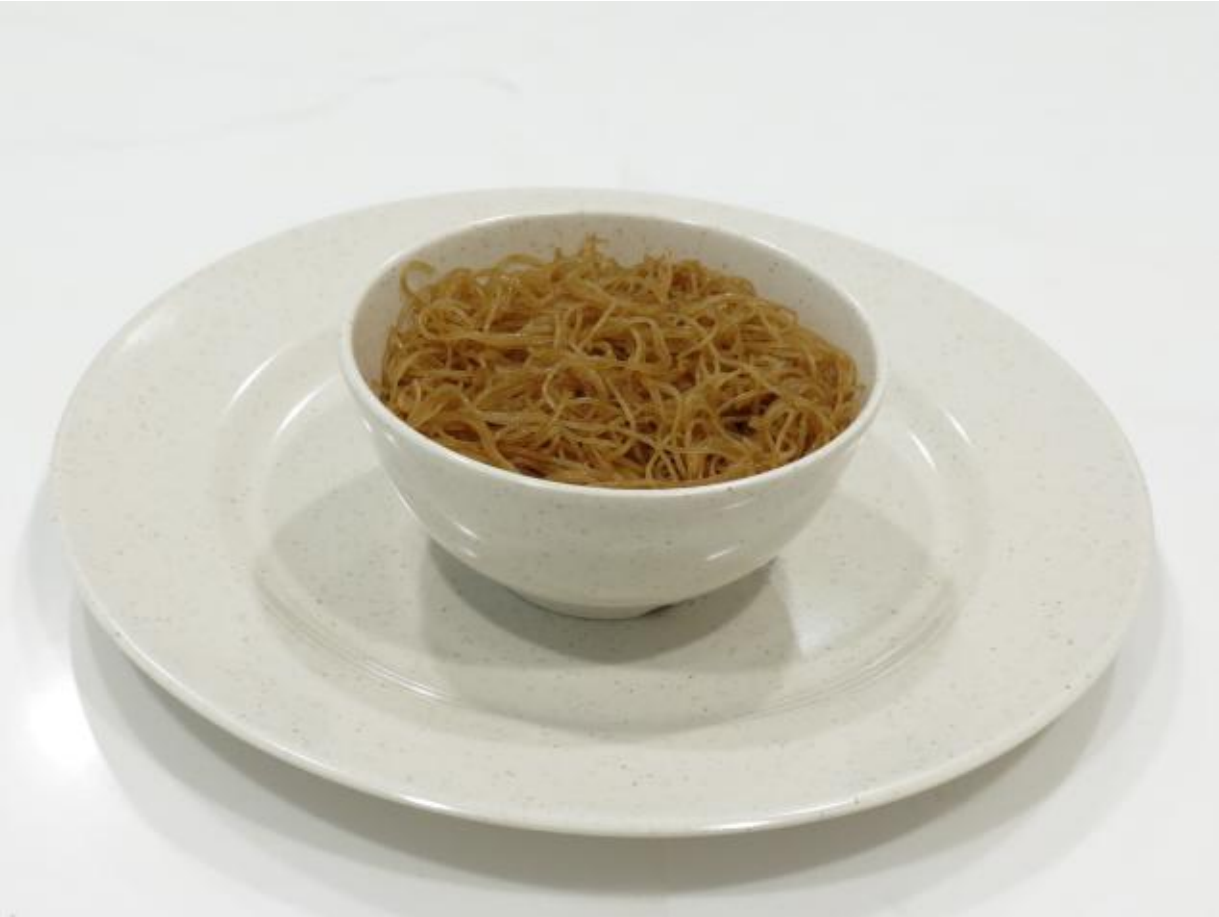

Rice noodle, bee hoon, thick, fried (FA0195)

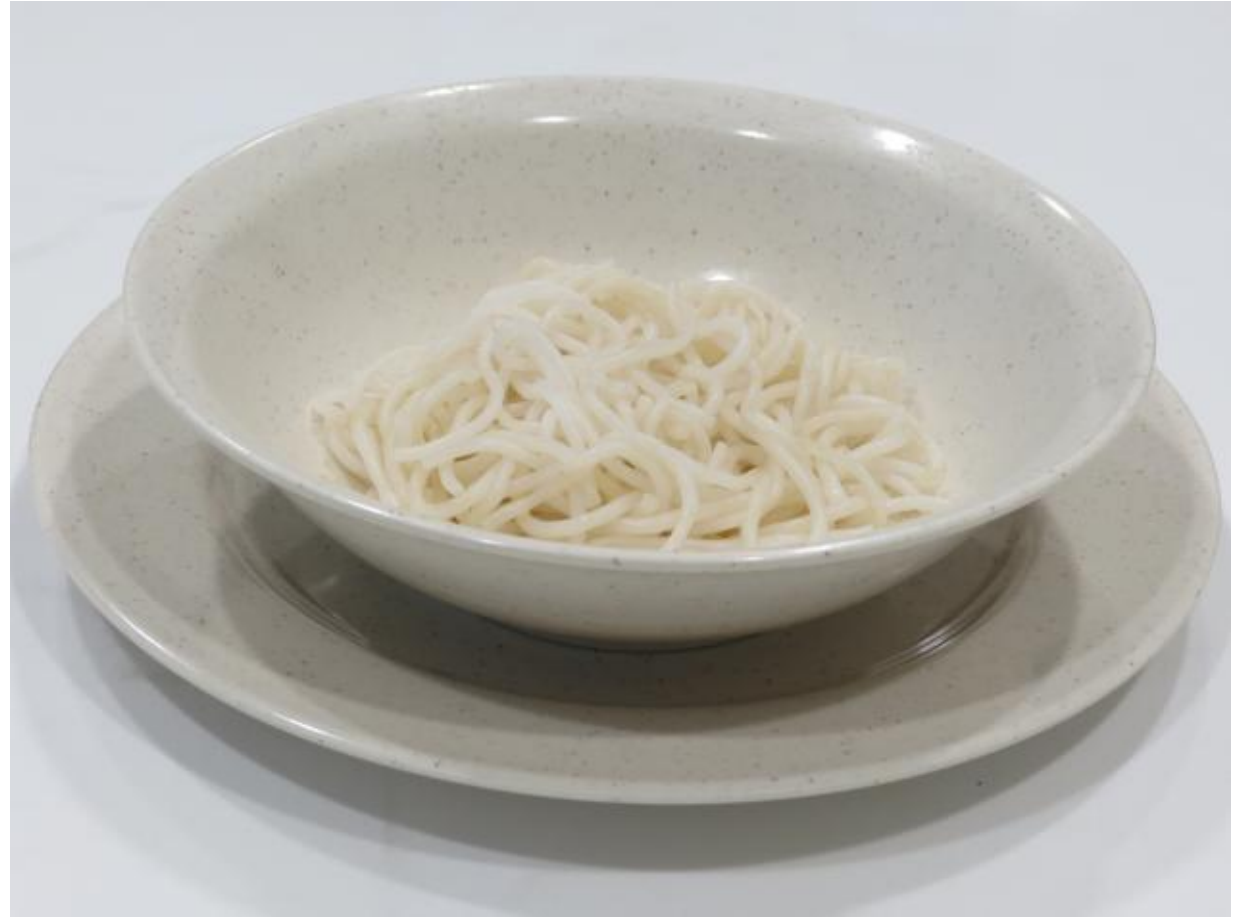
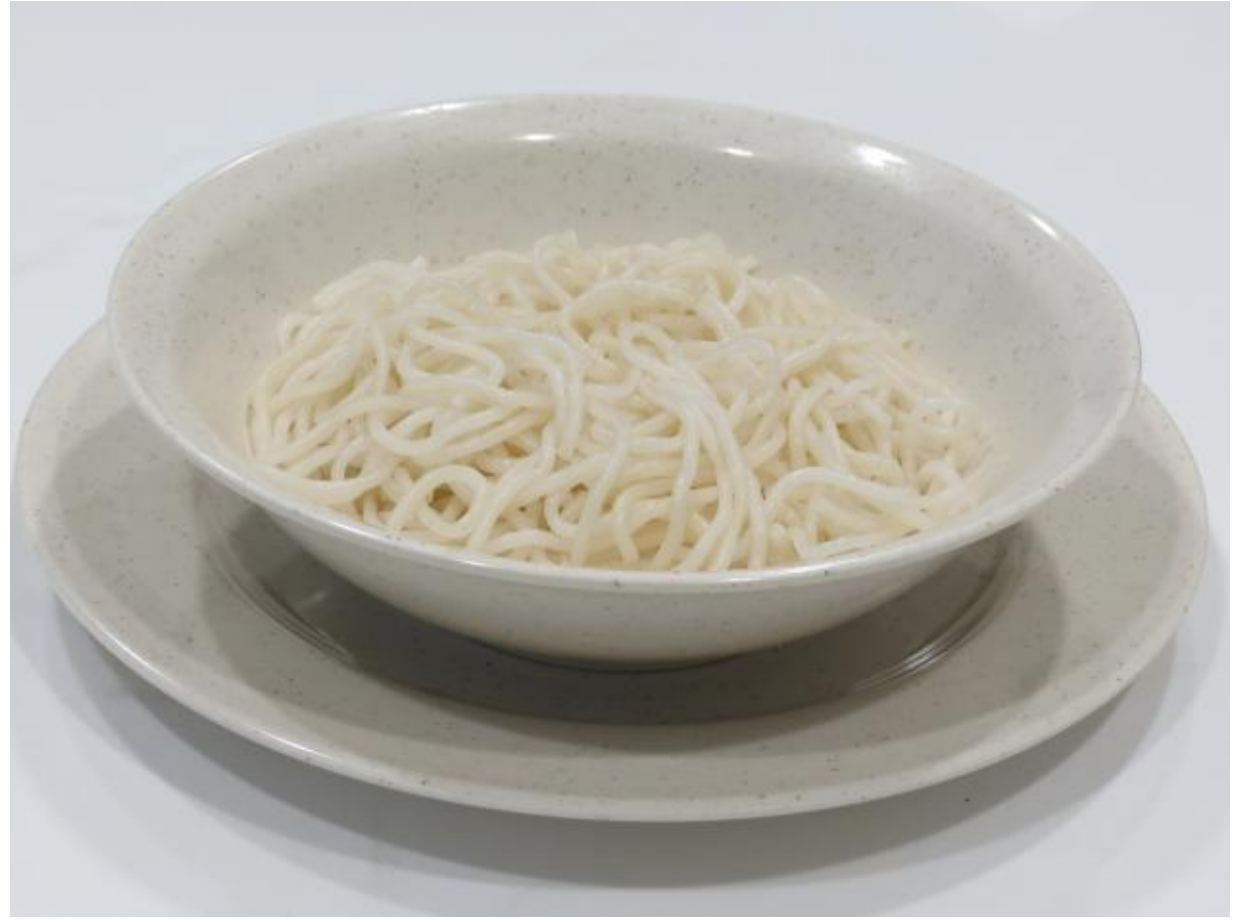
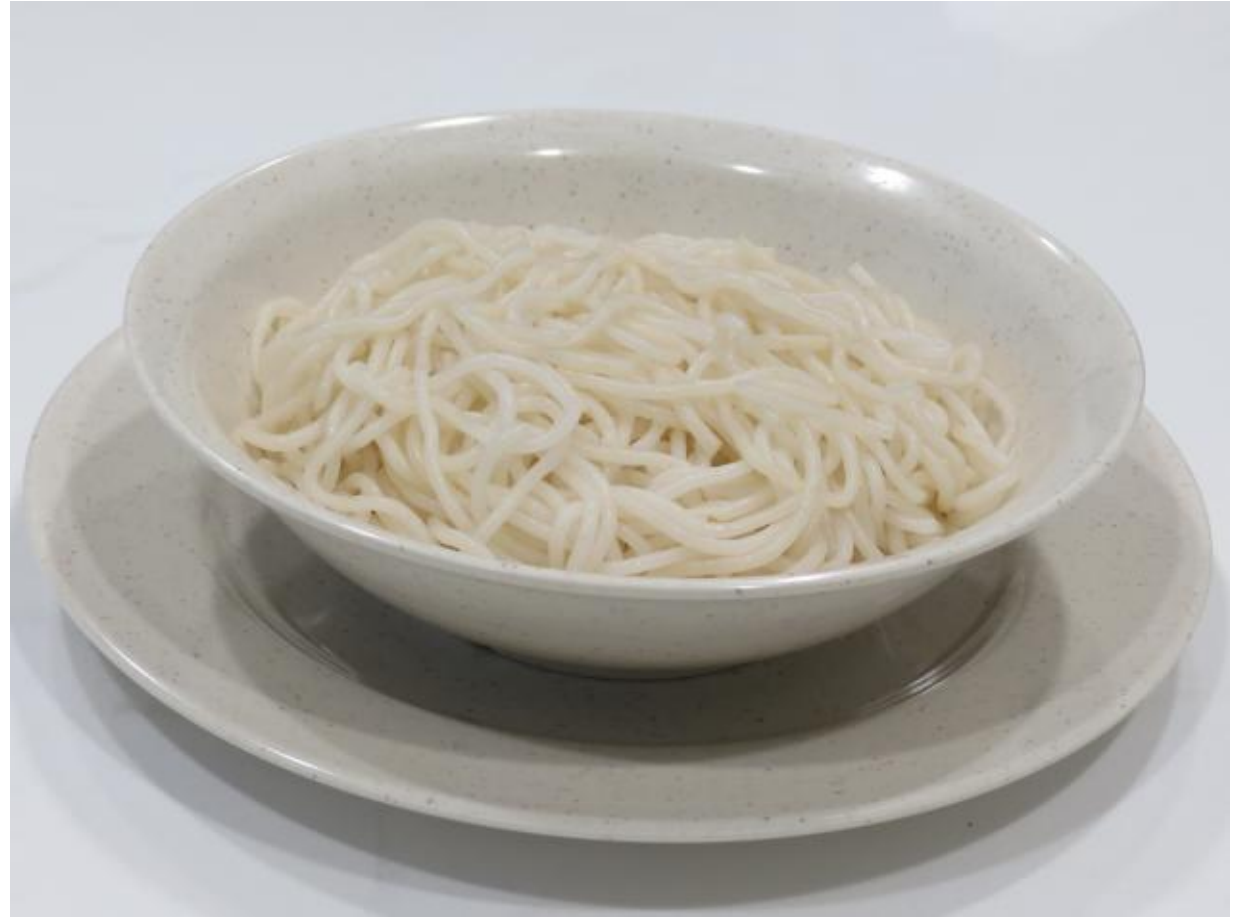
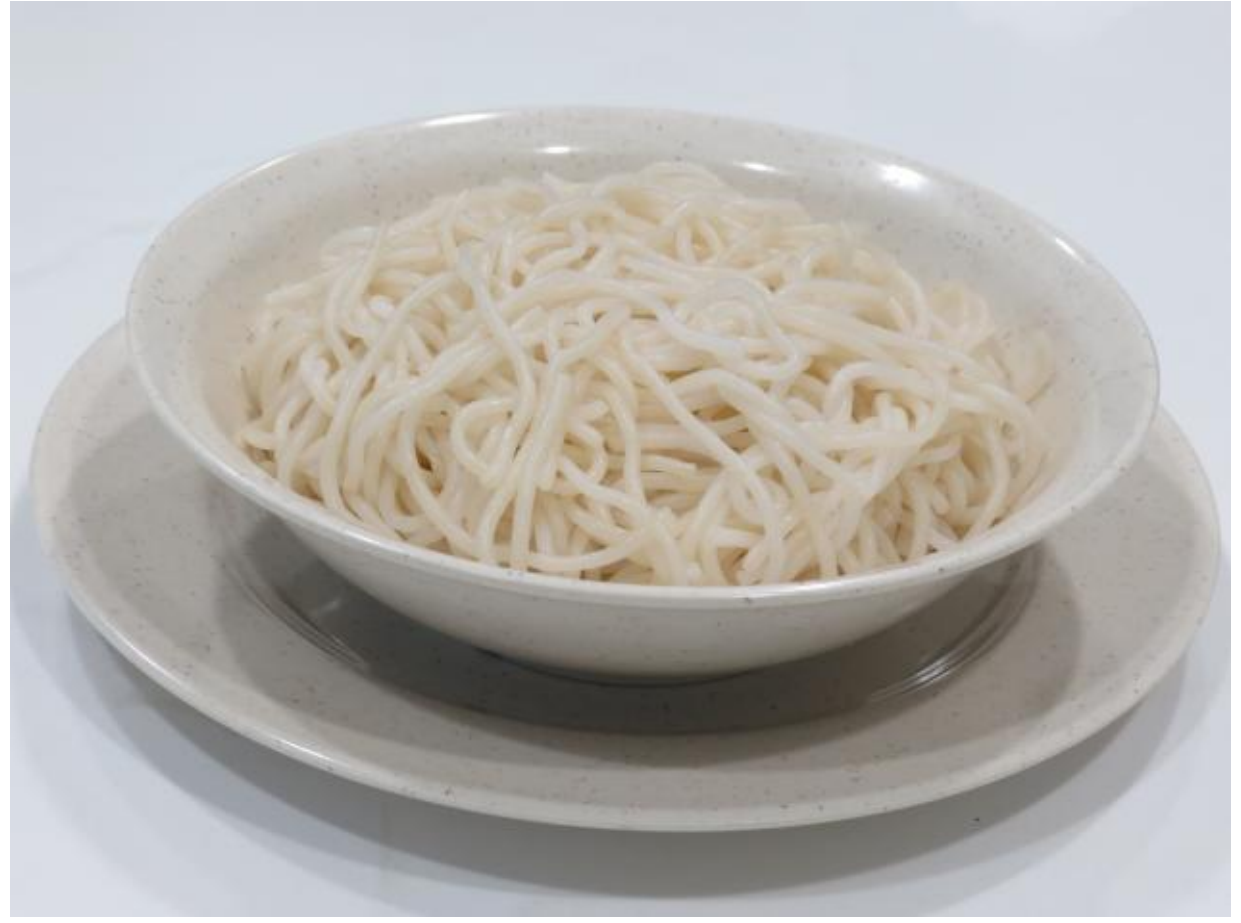

Rice, white, japonica (FA0081)

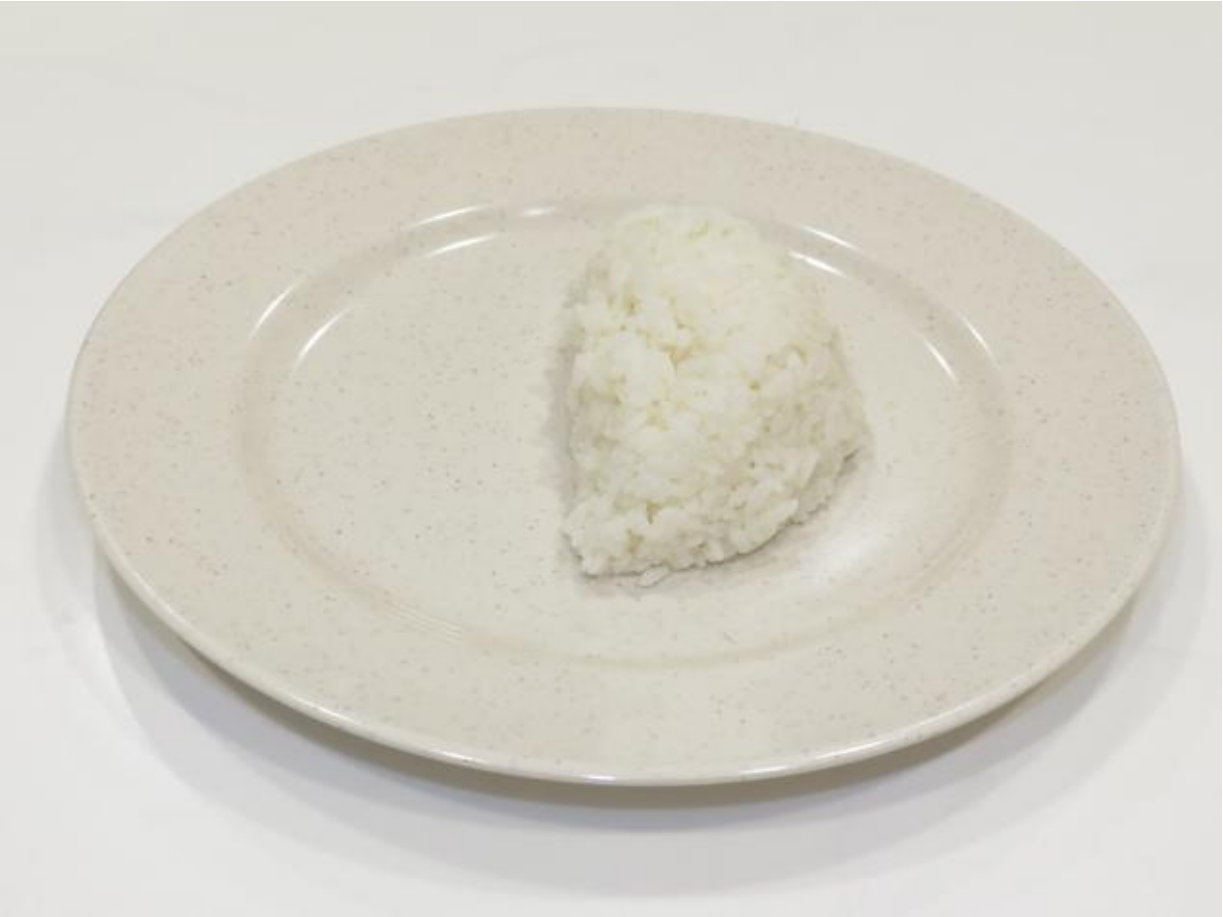

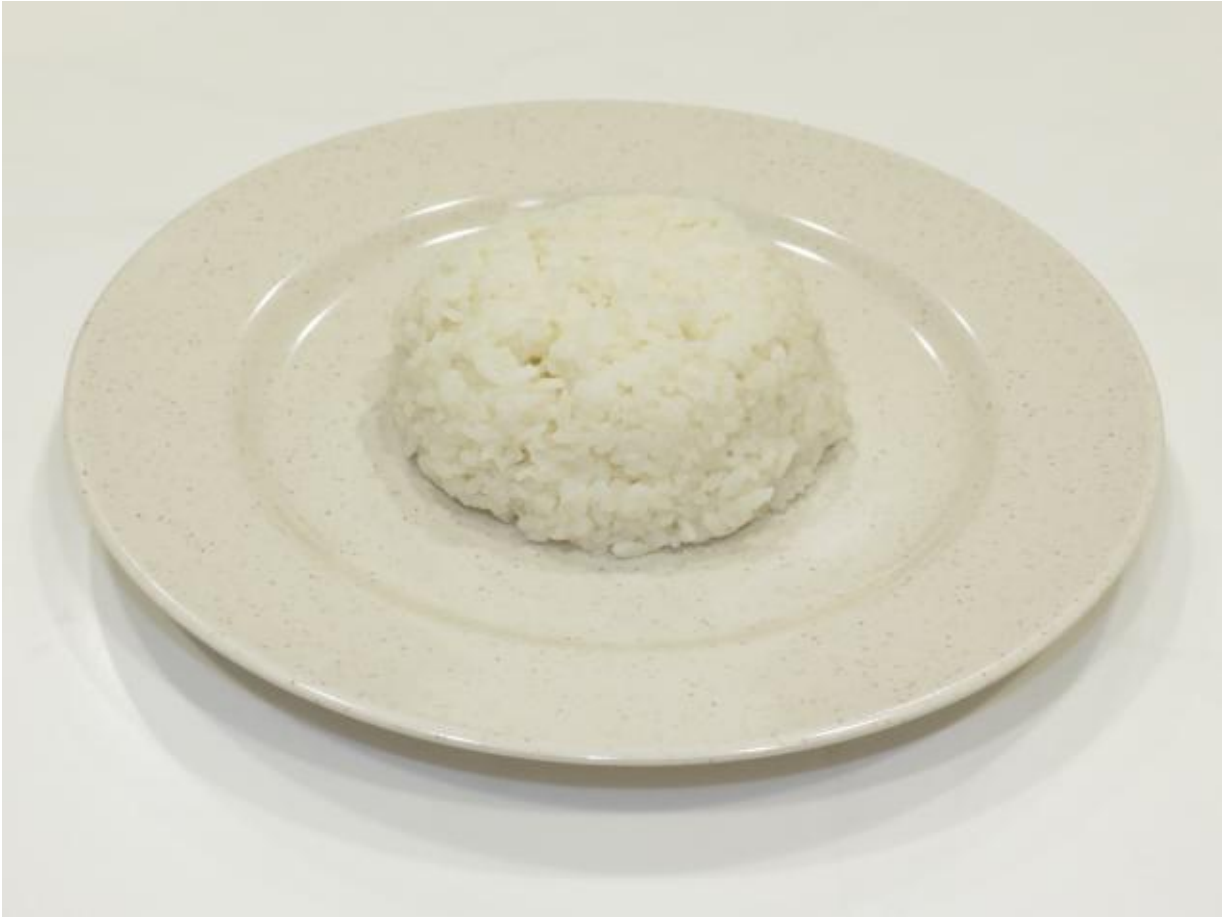

Rice, white, regular (FA0193)

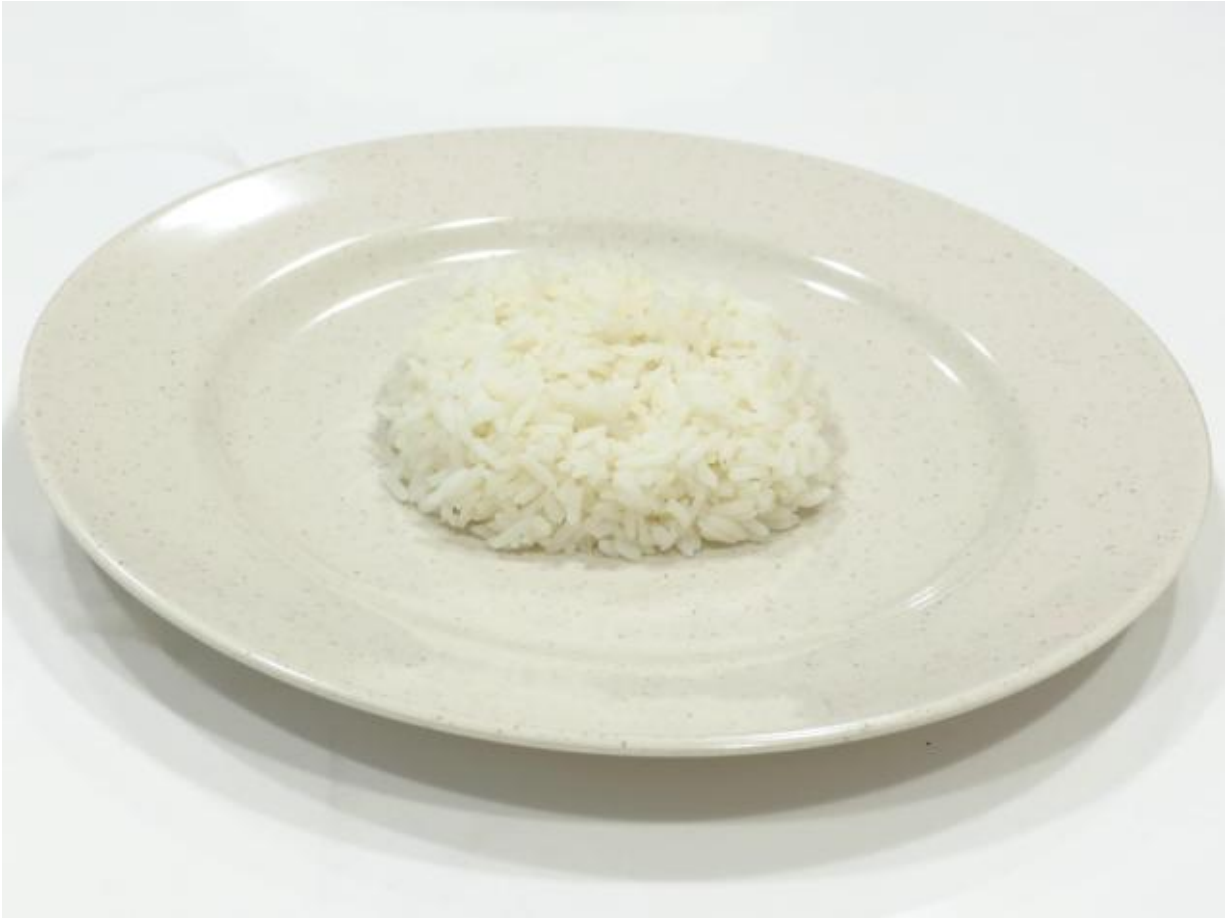
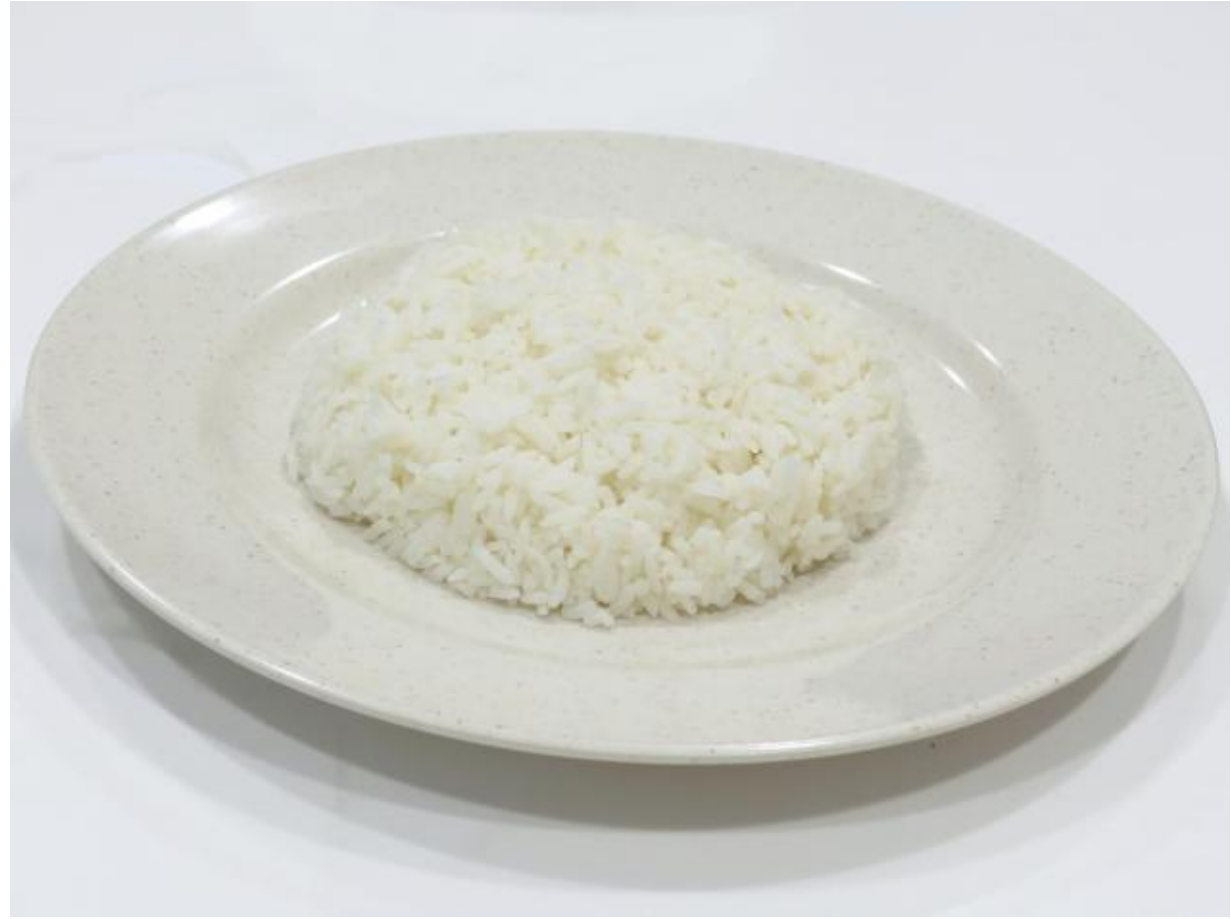
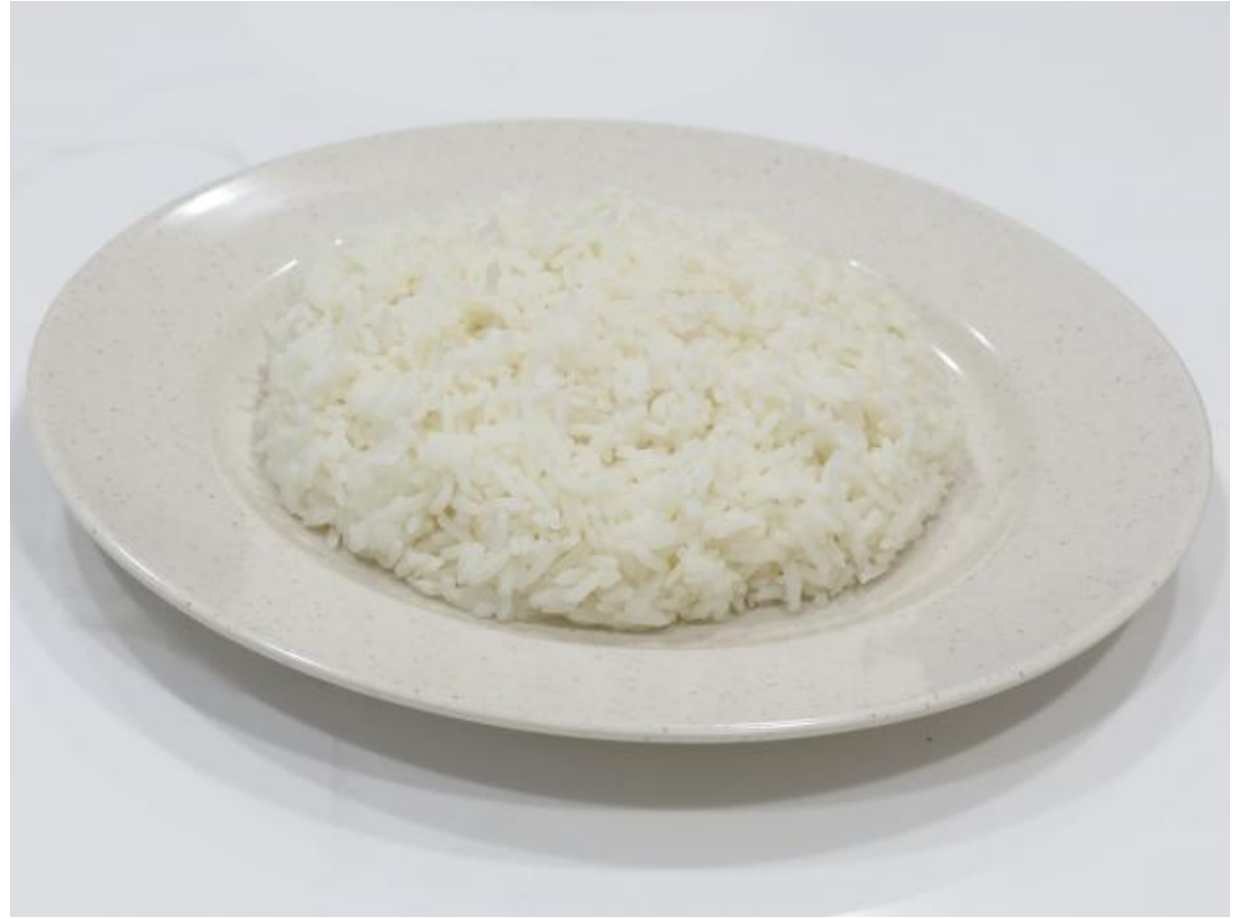
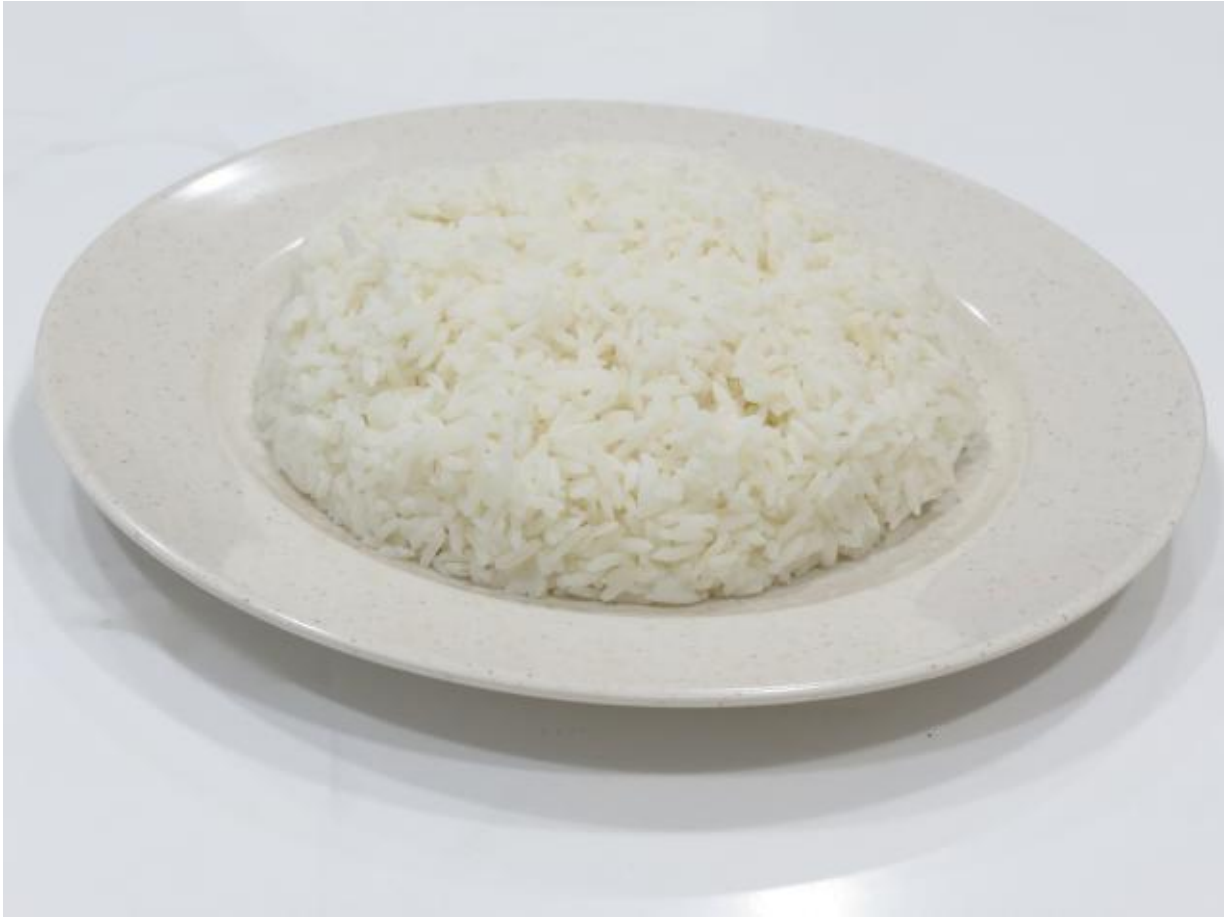

Beef, lean, sliced (FA0191)

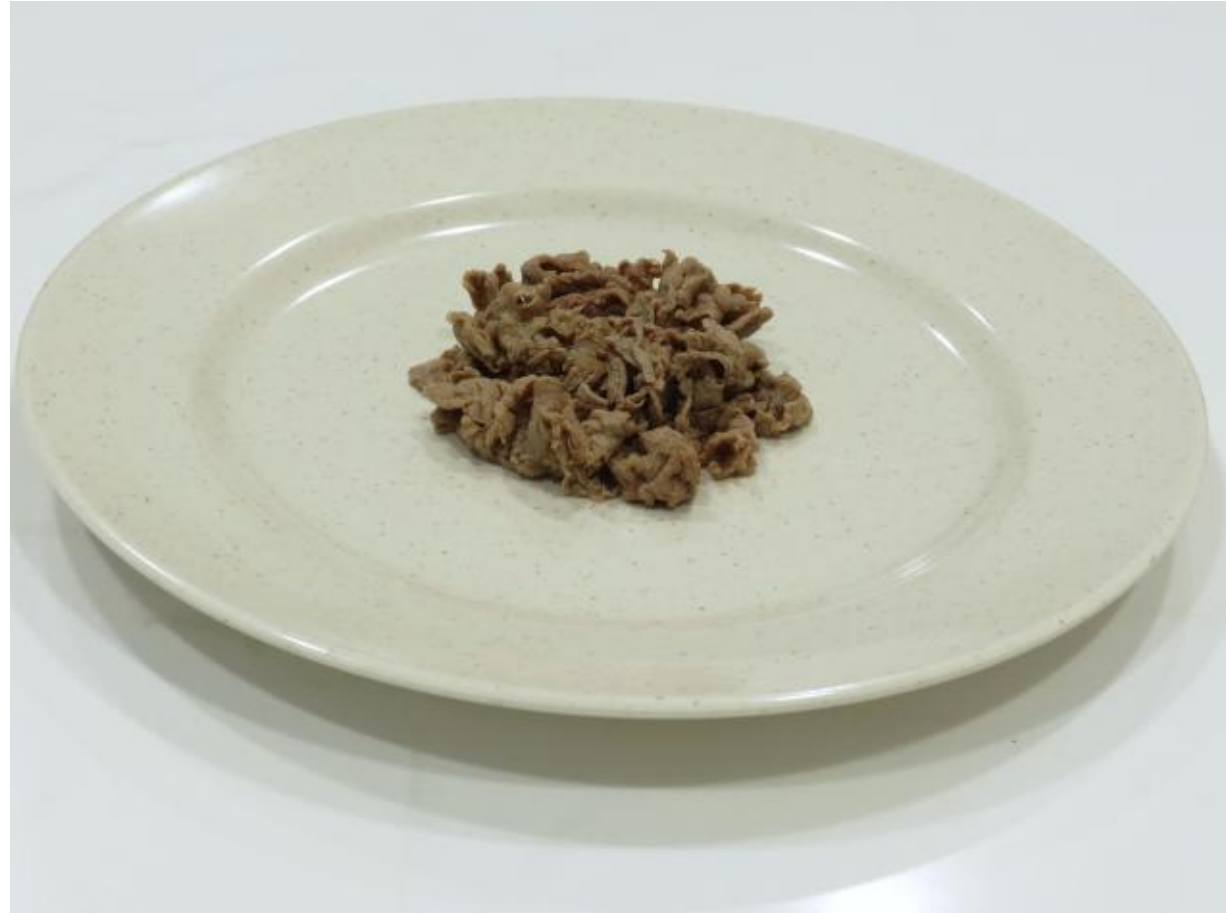 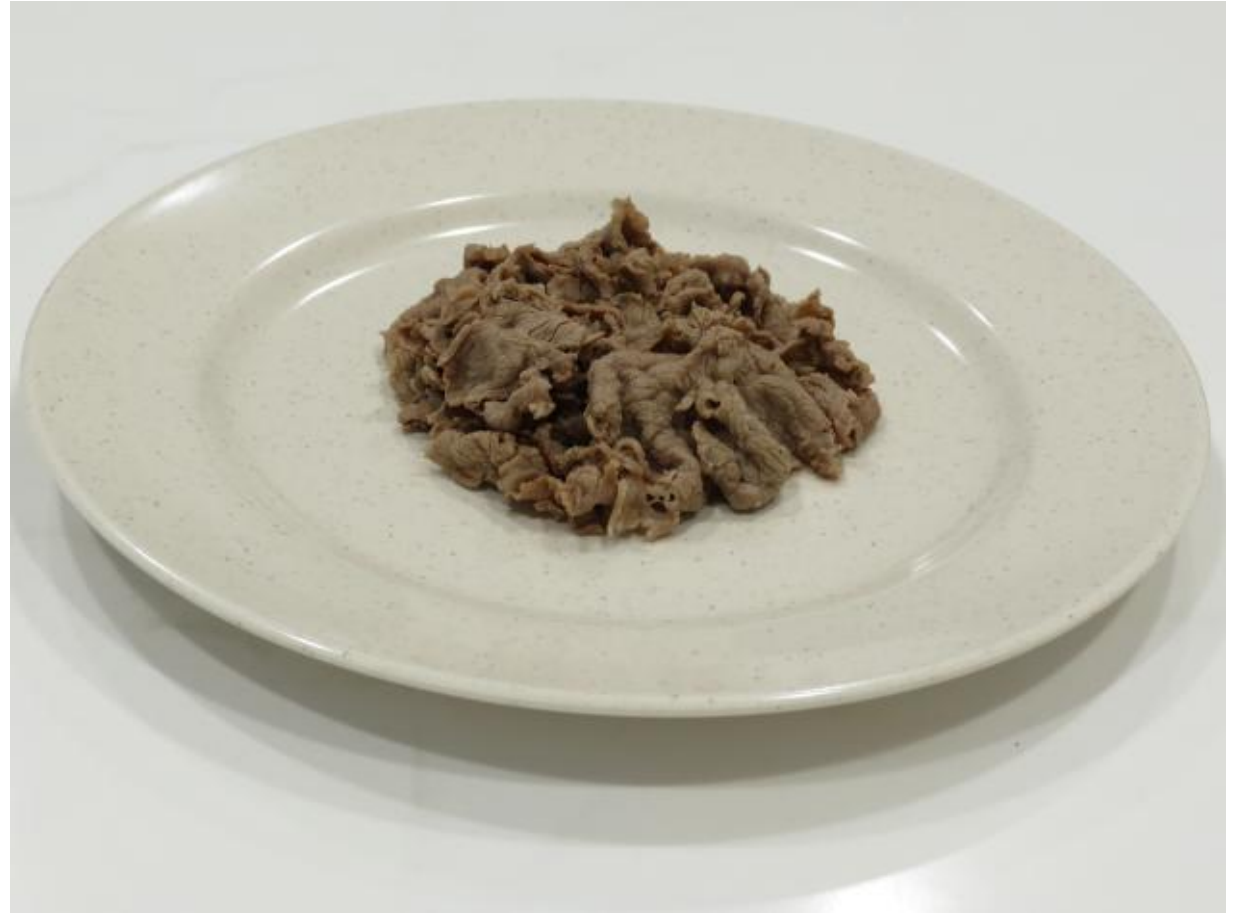 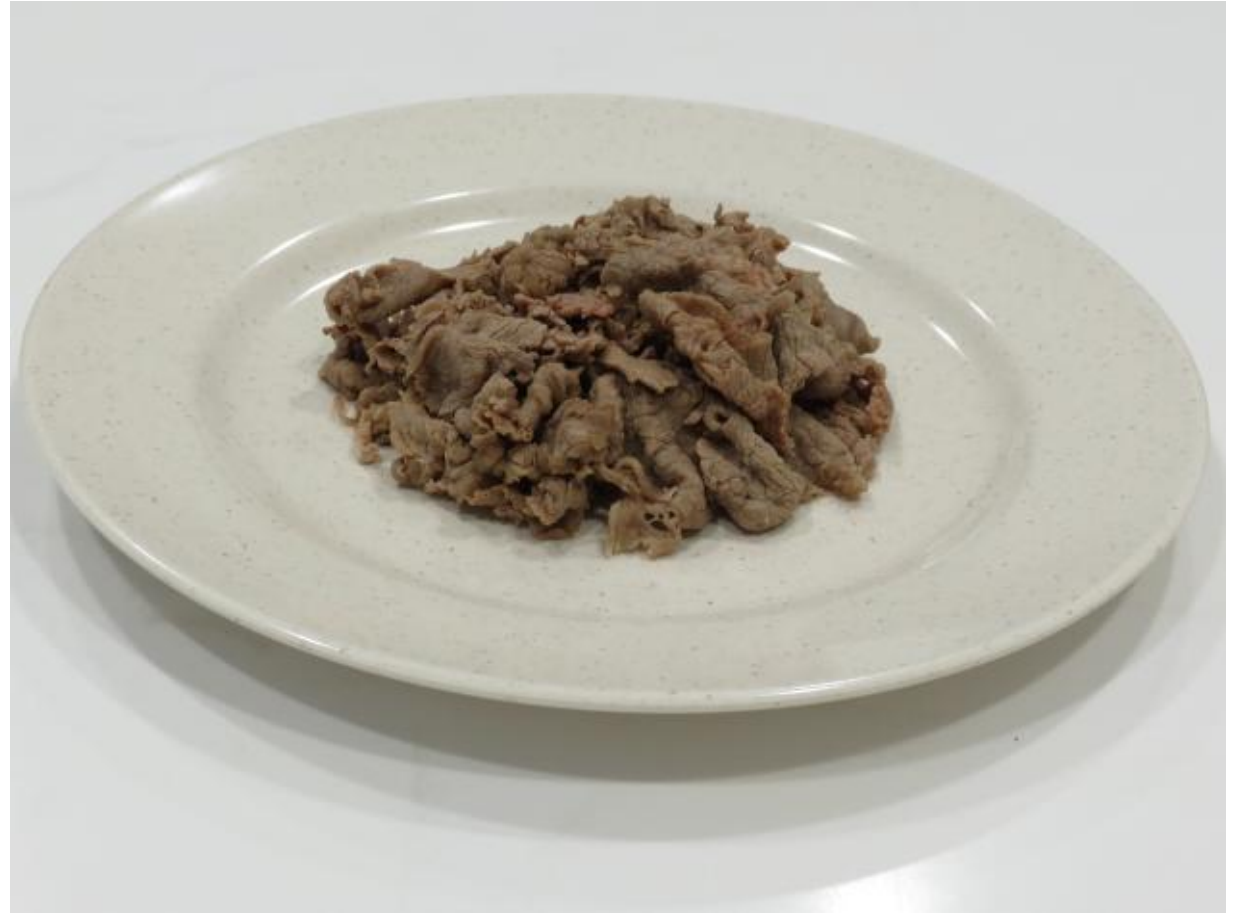 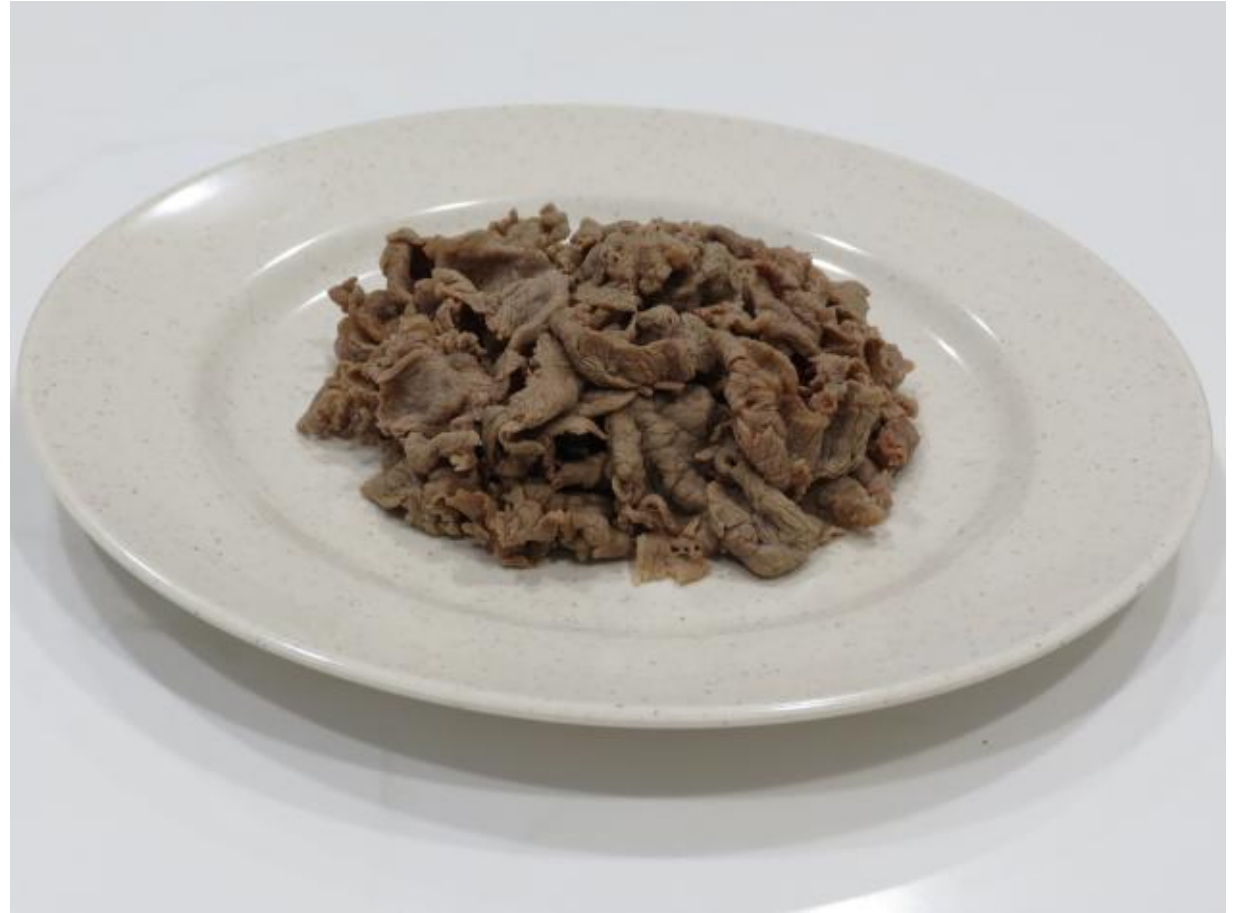

Beef, with fat, sliced (FA0079)

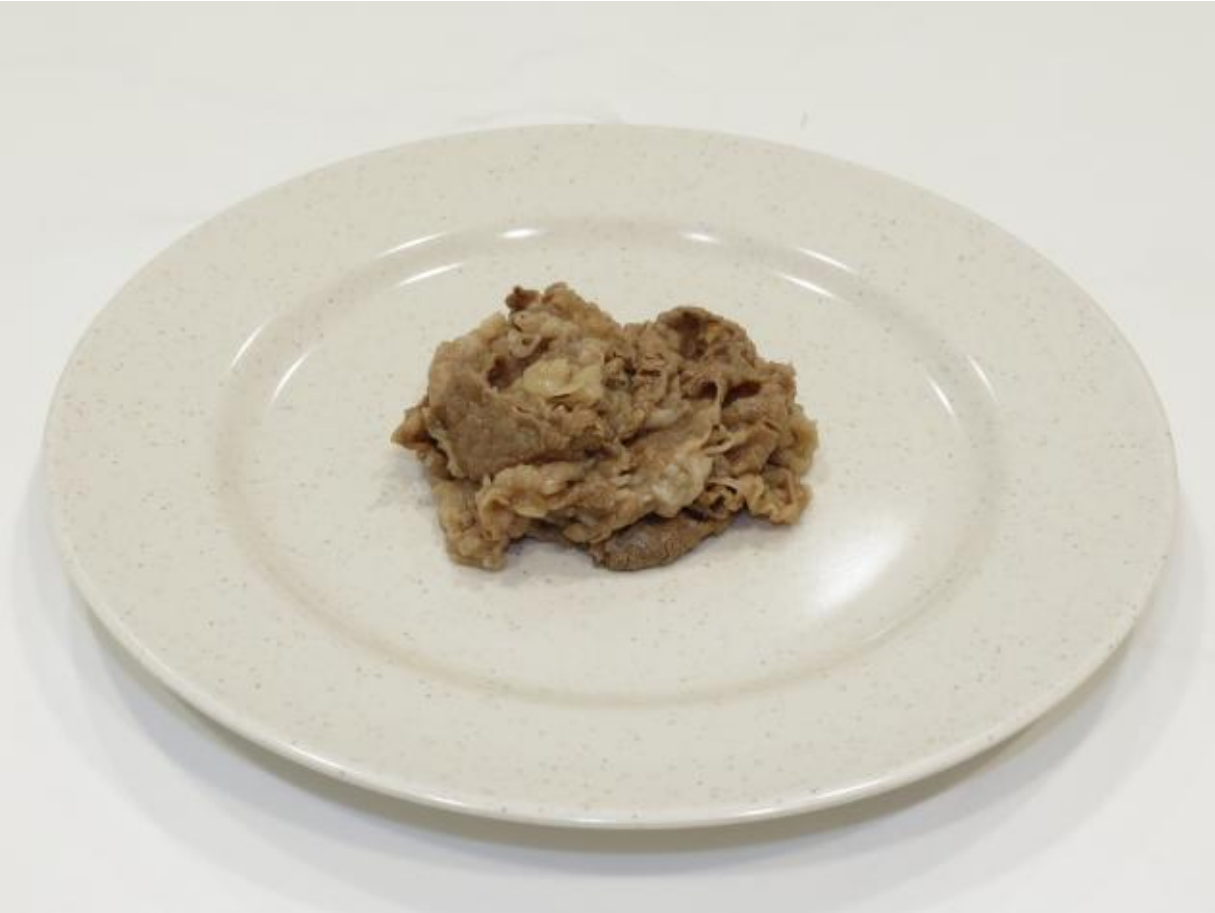
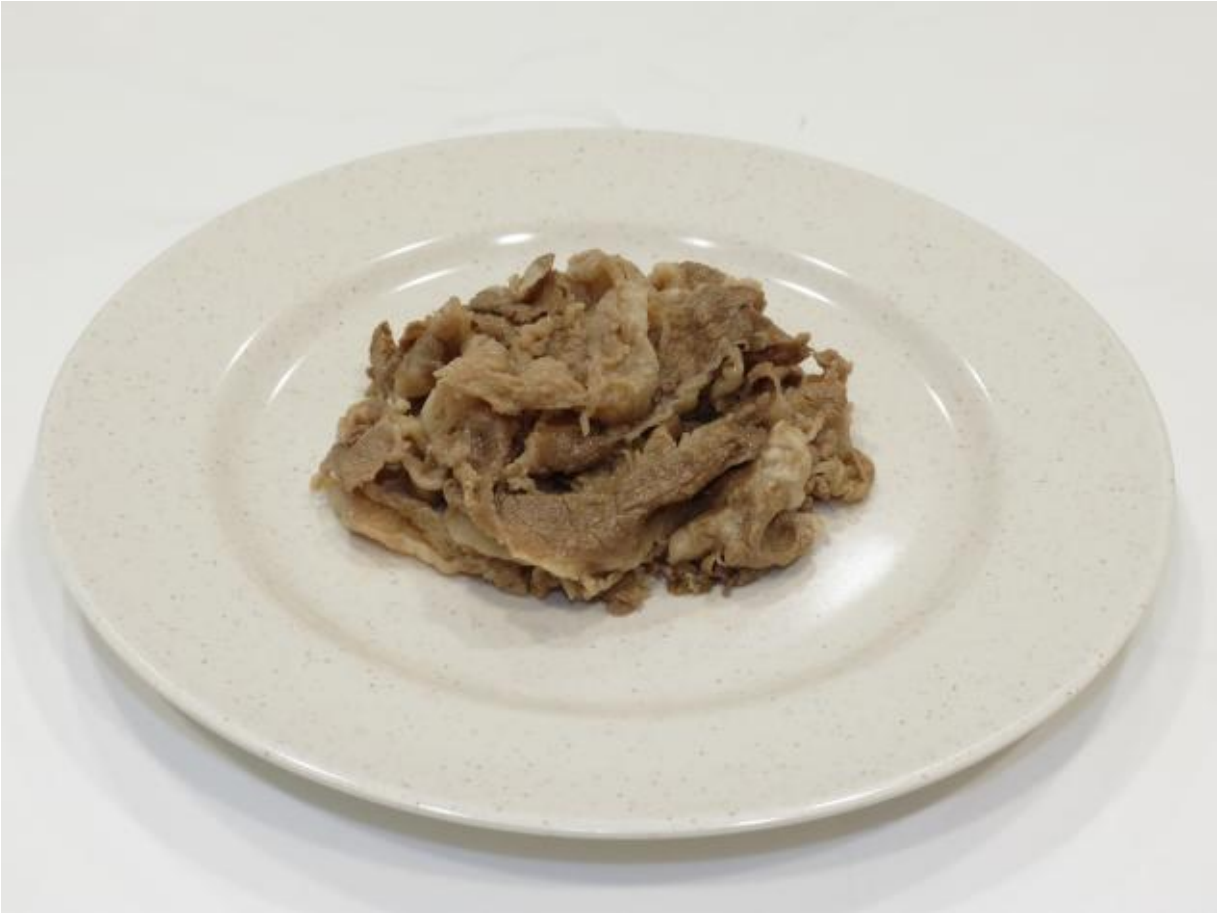
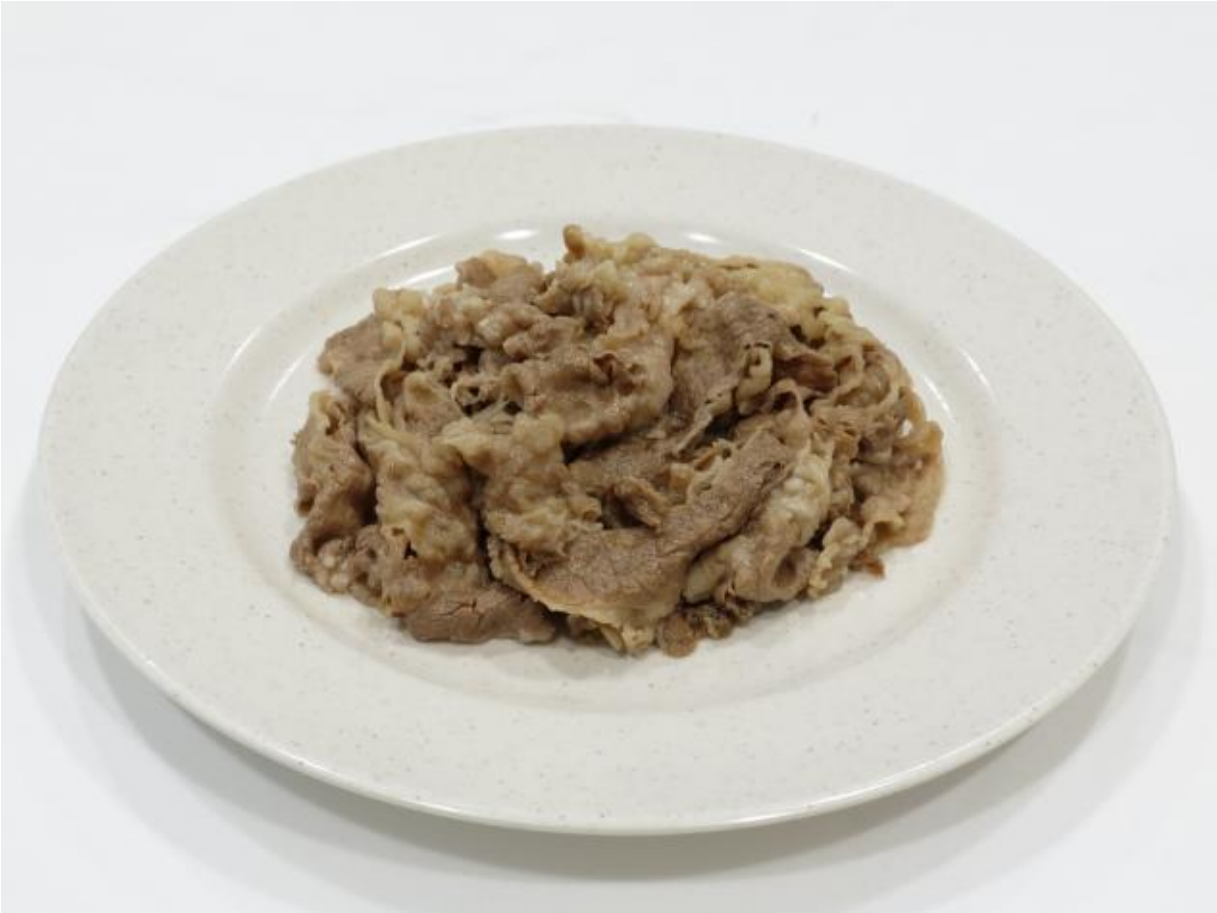
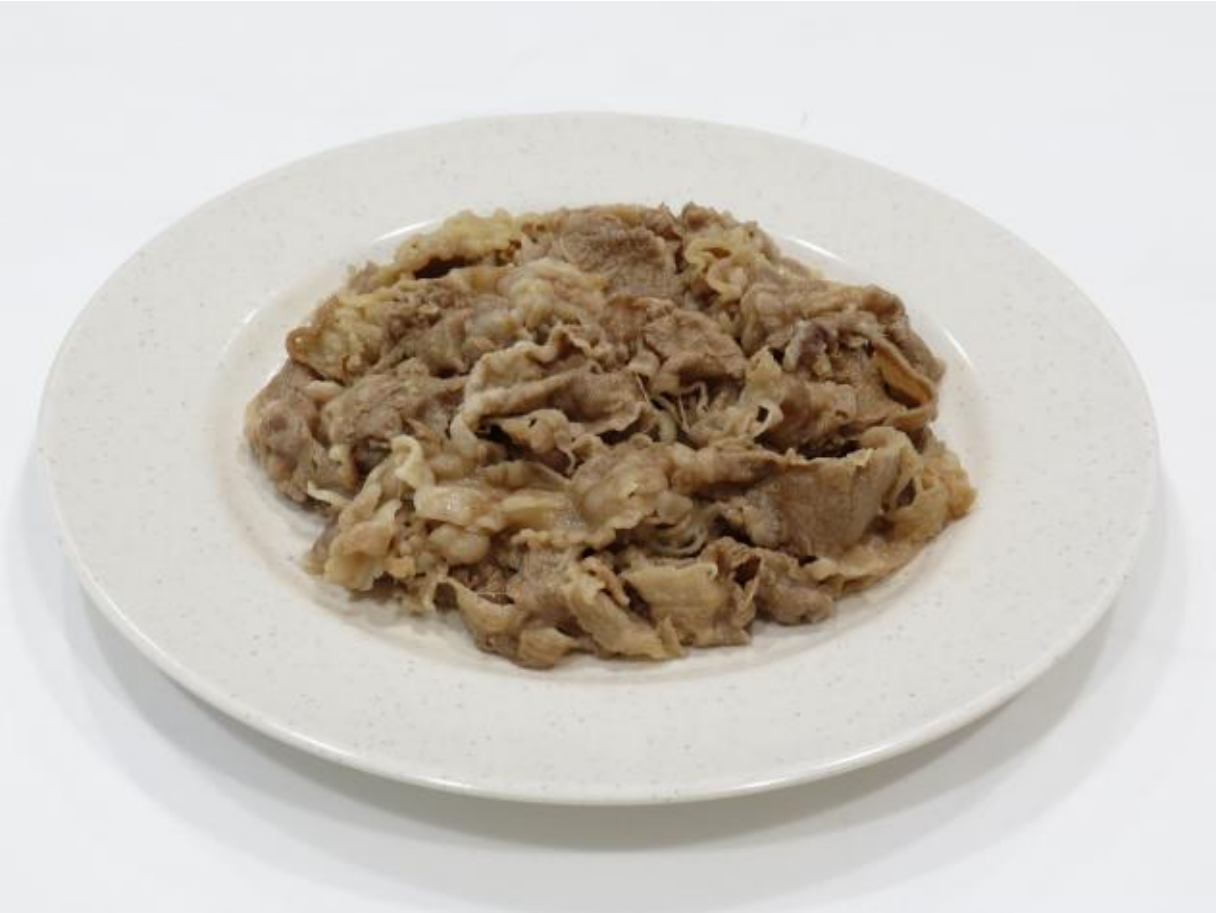

Chicken, boneless, pieces (FA0192)

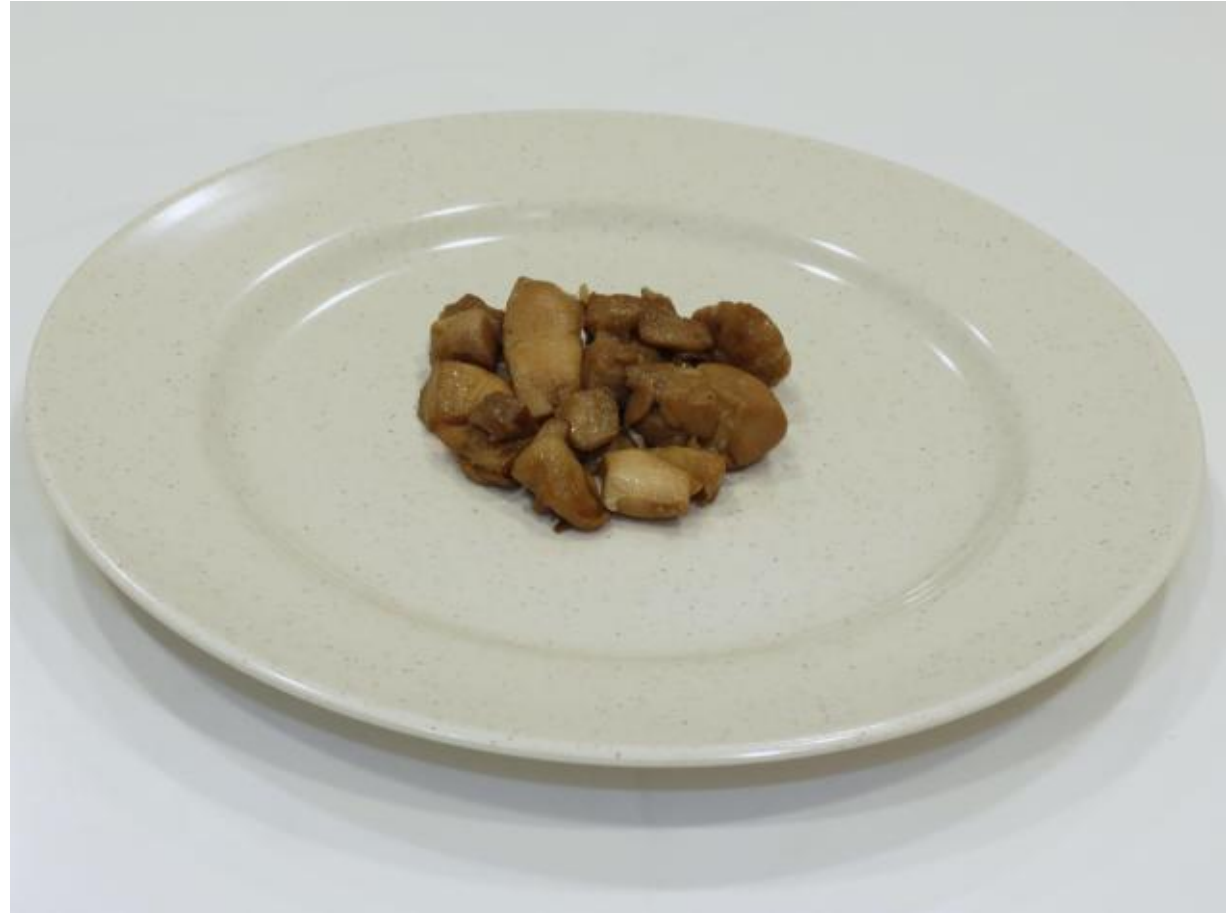

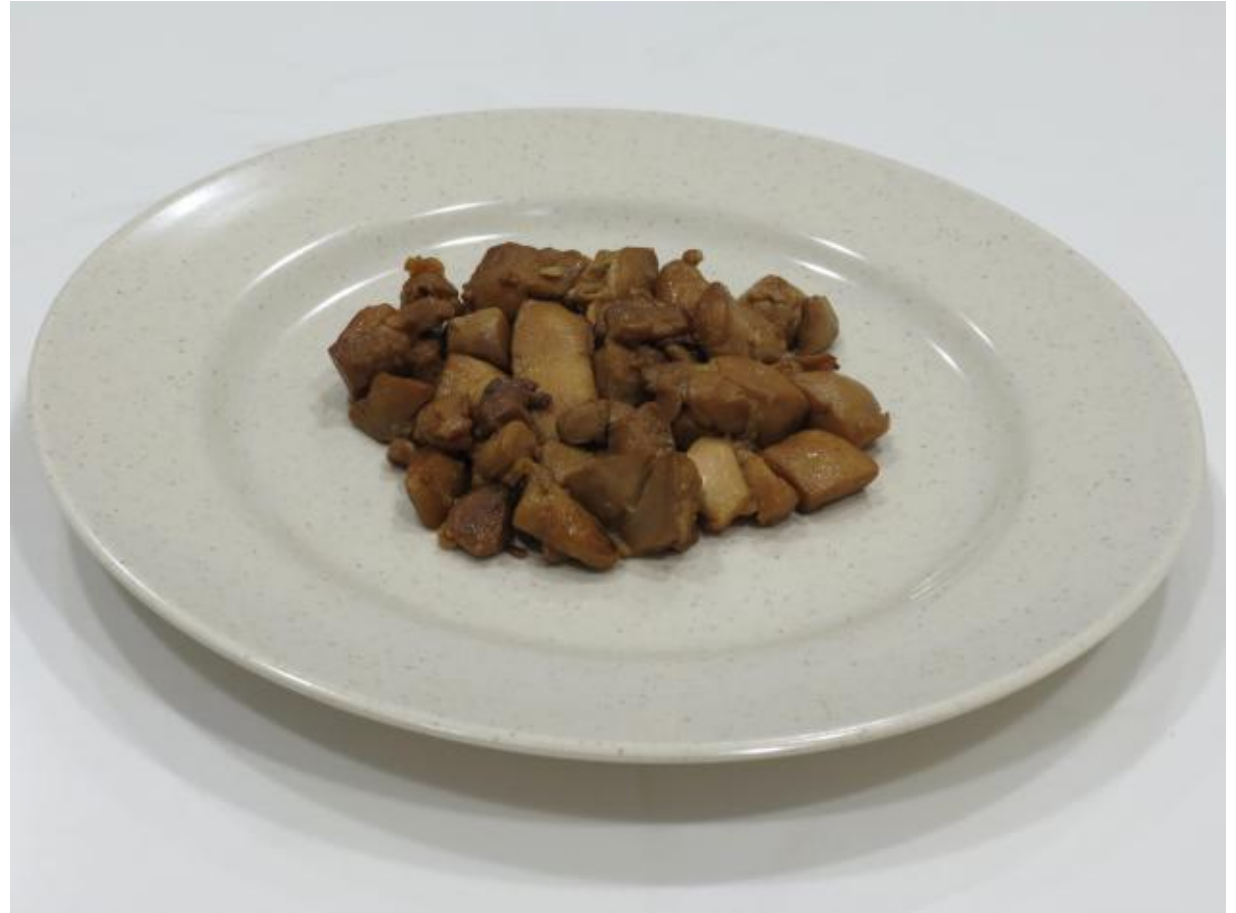

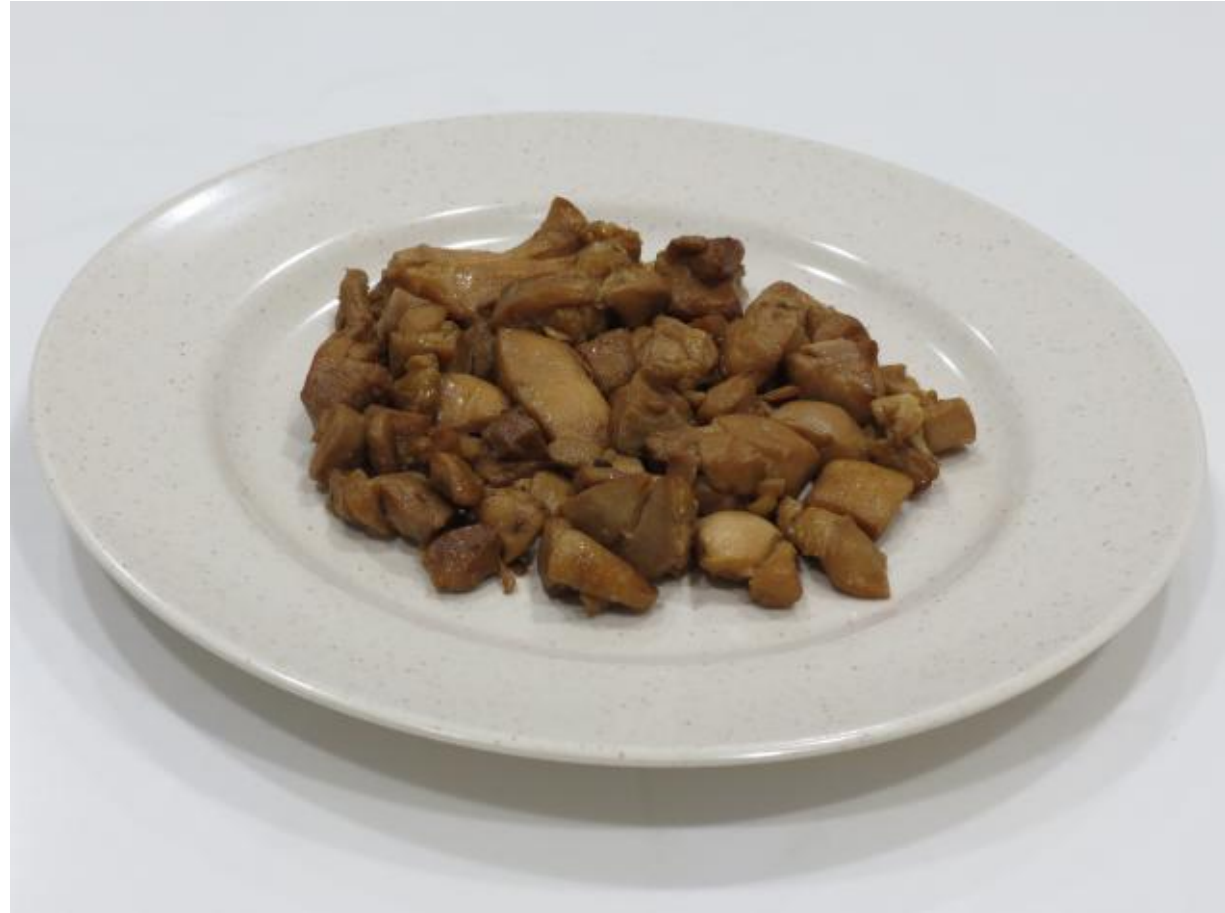

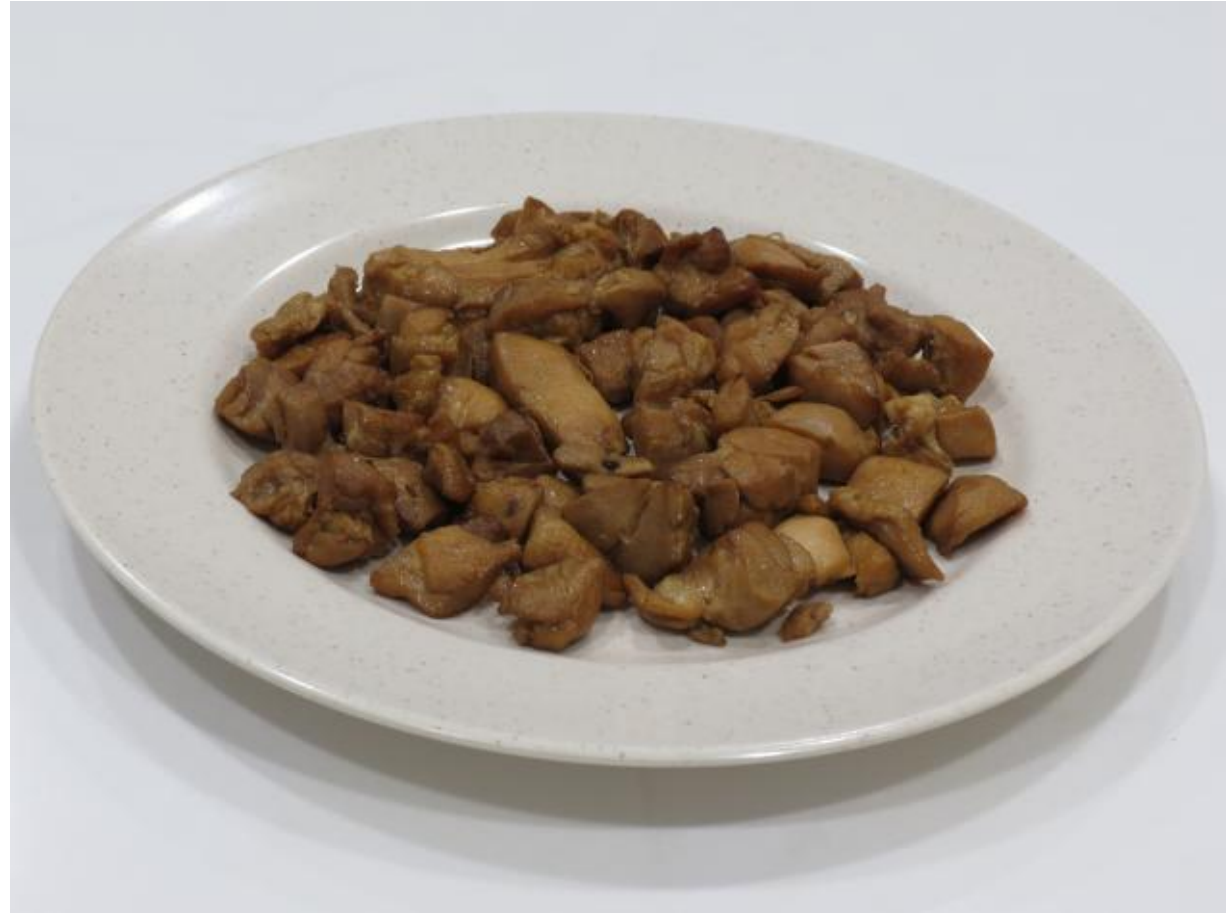

Chicken, leg, boneless (FA0140)

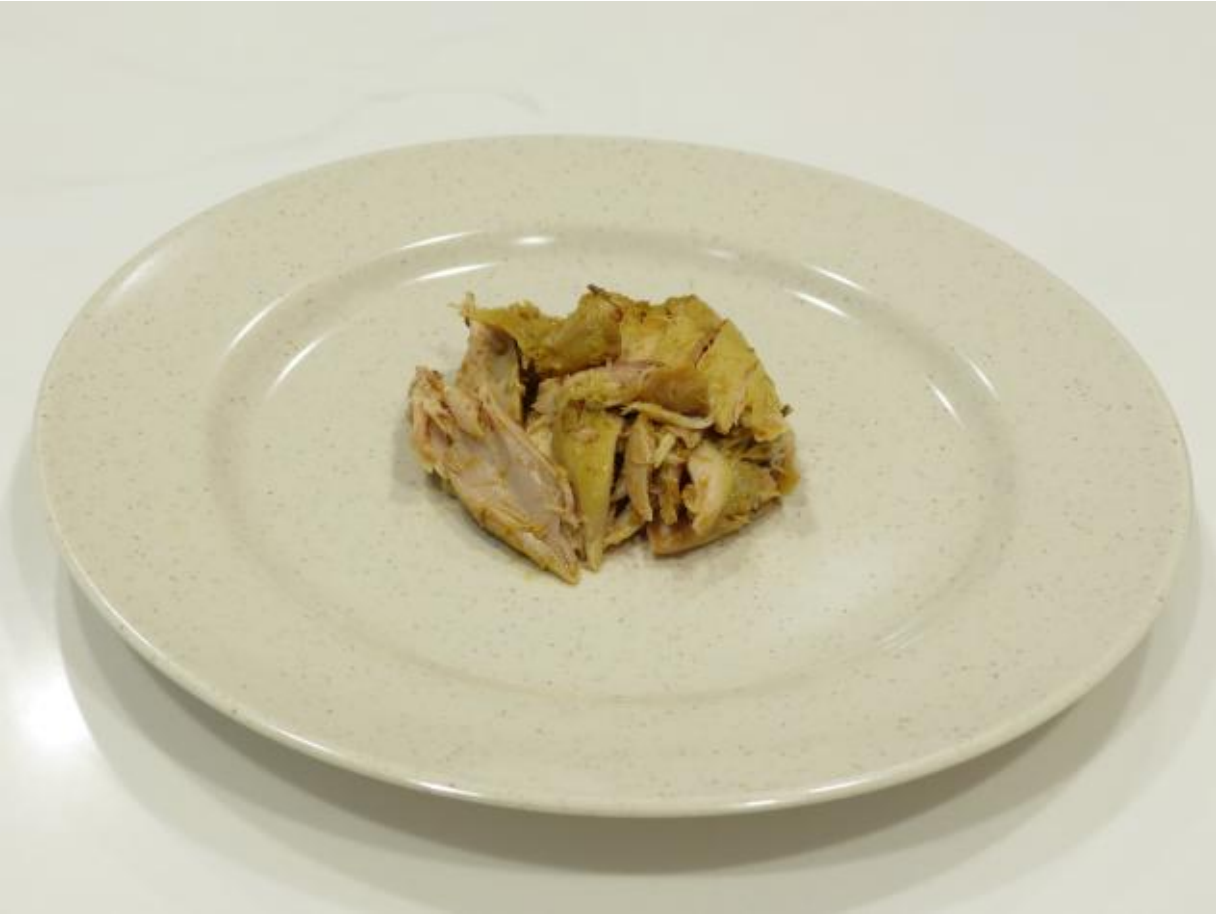 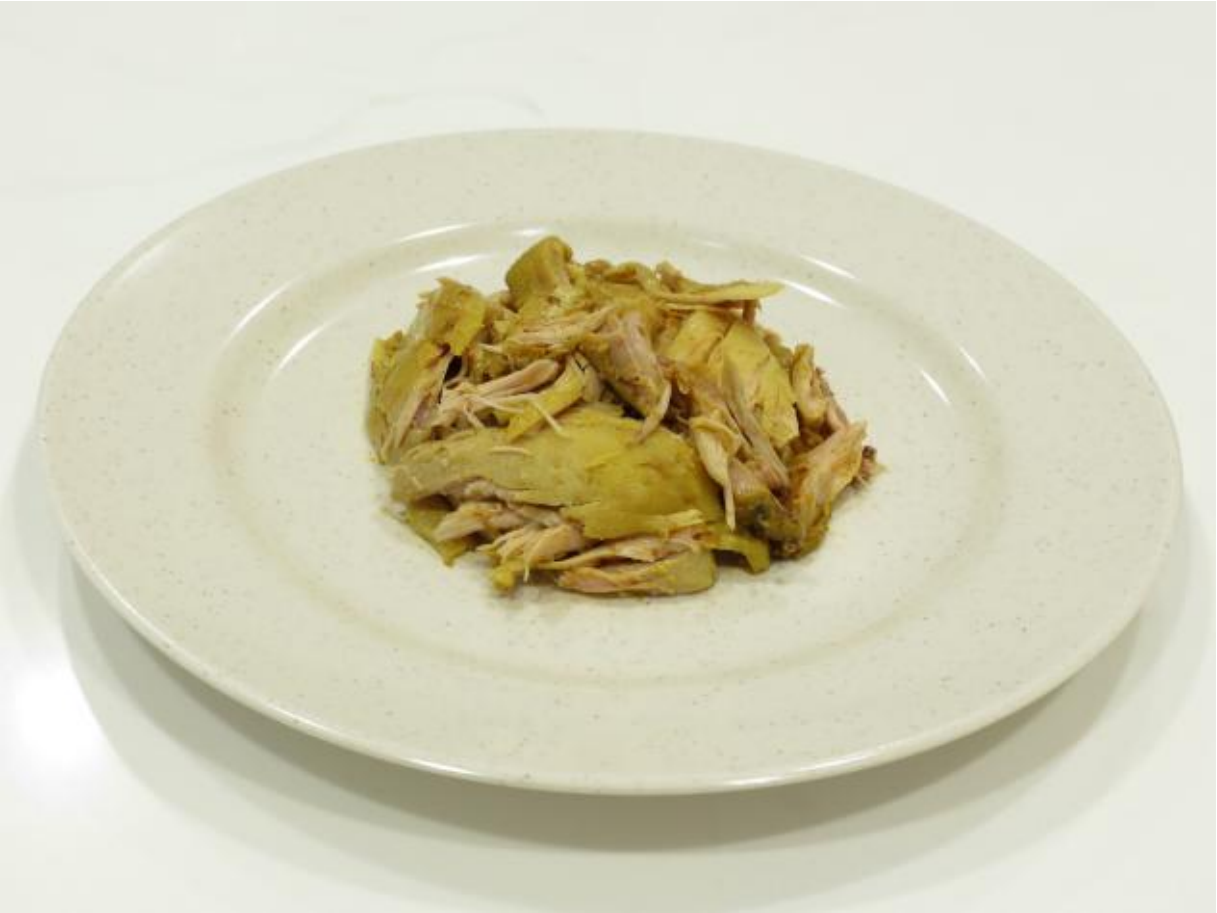

Pork, chop, boneless (FA0124)

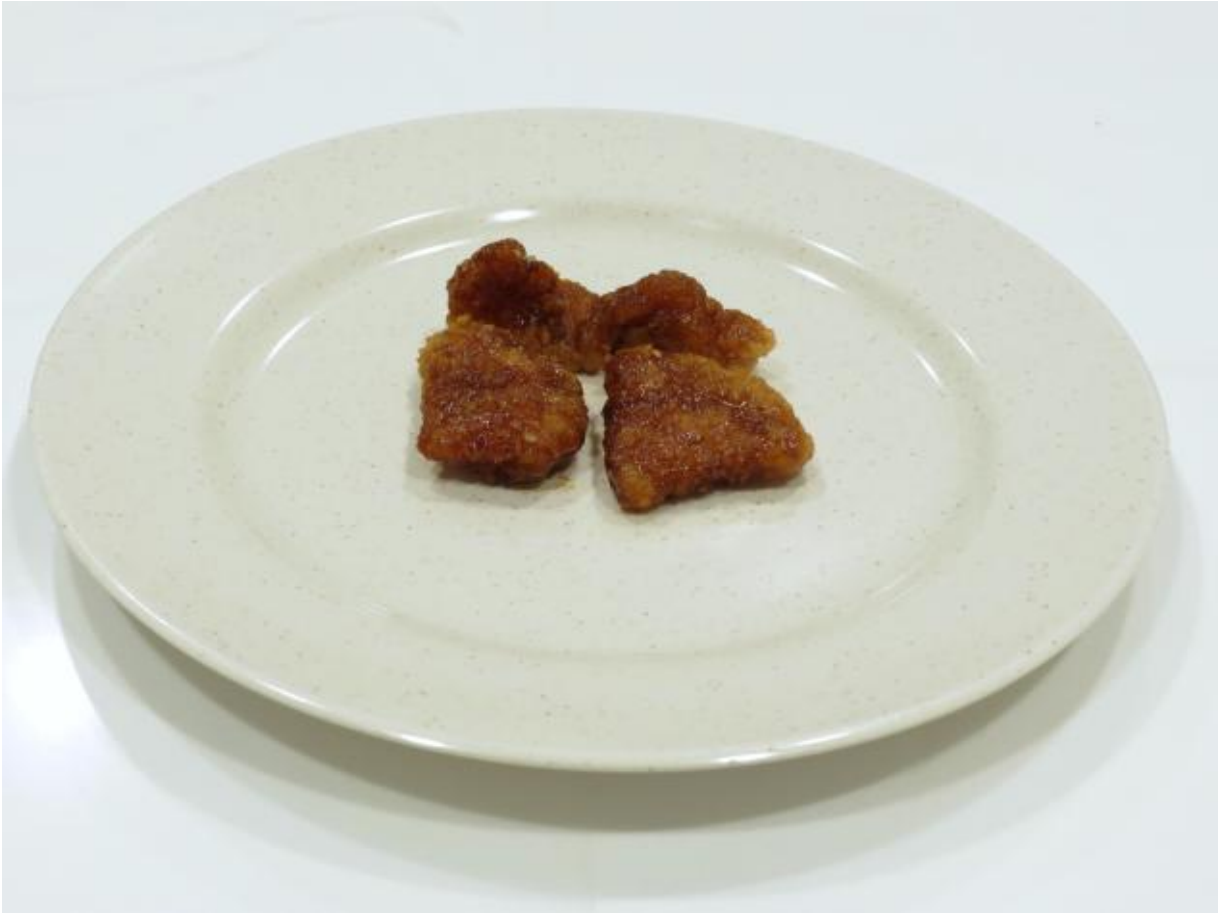
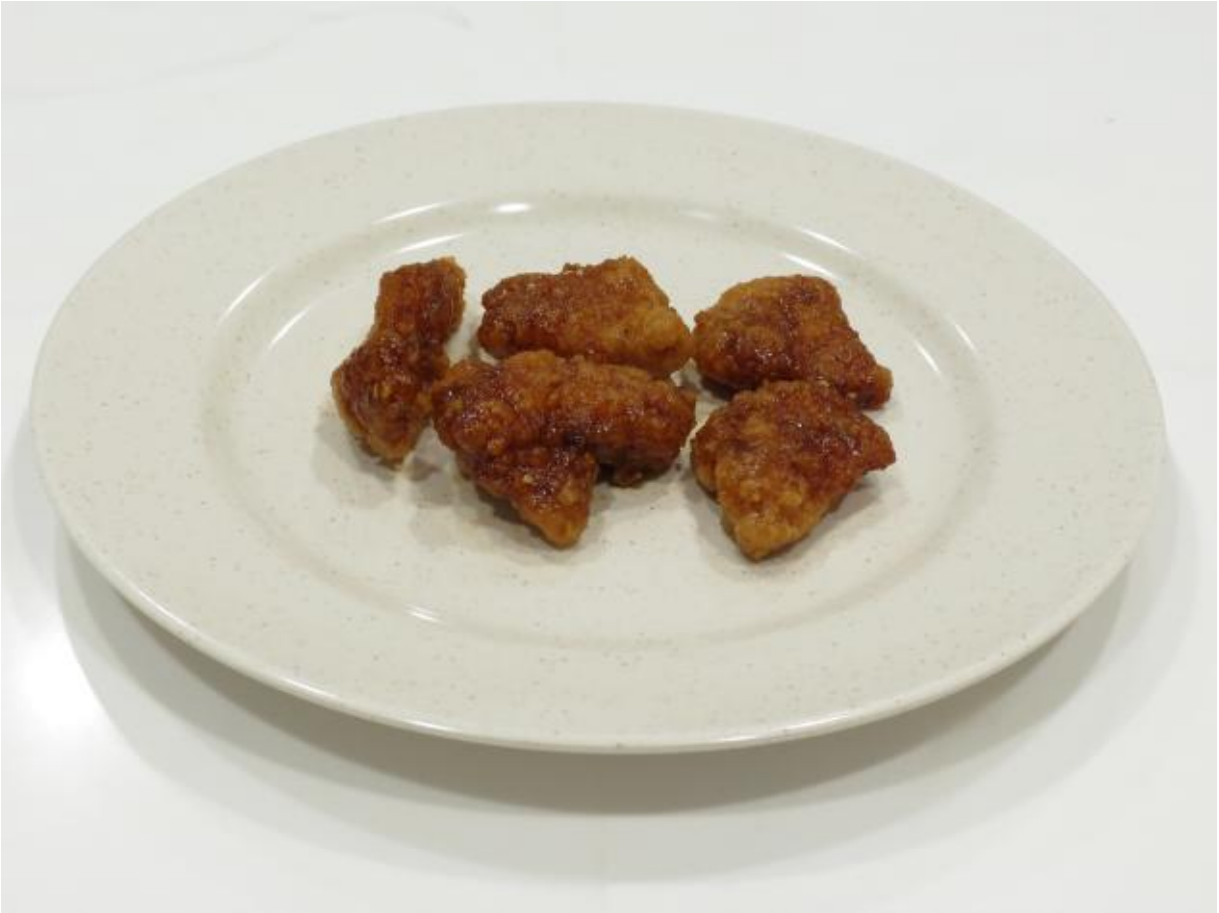
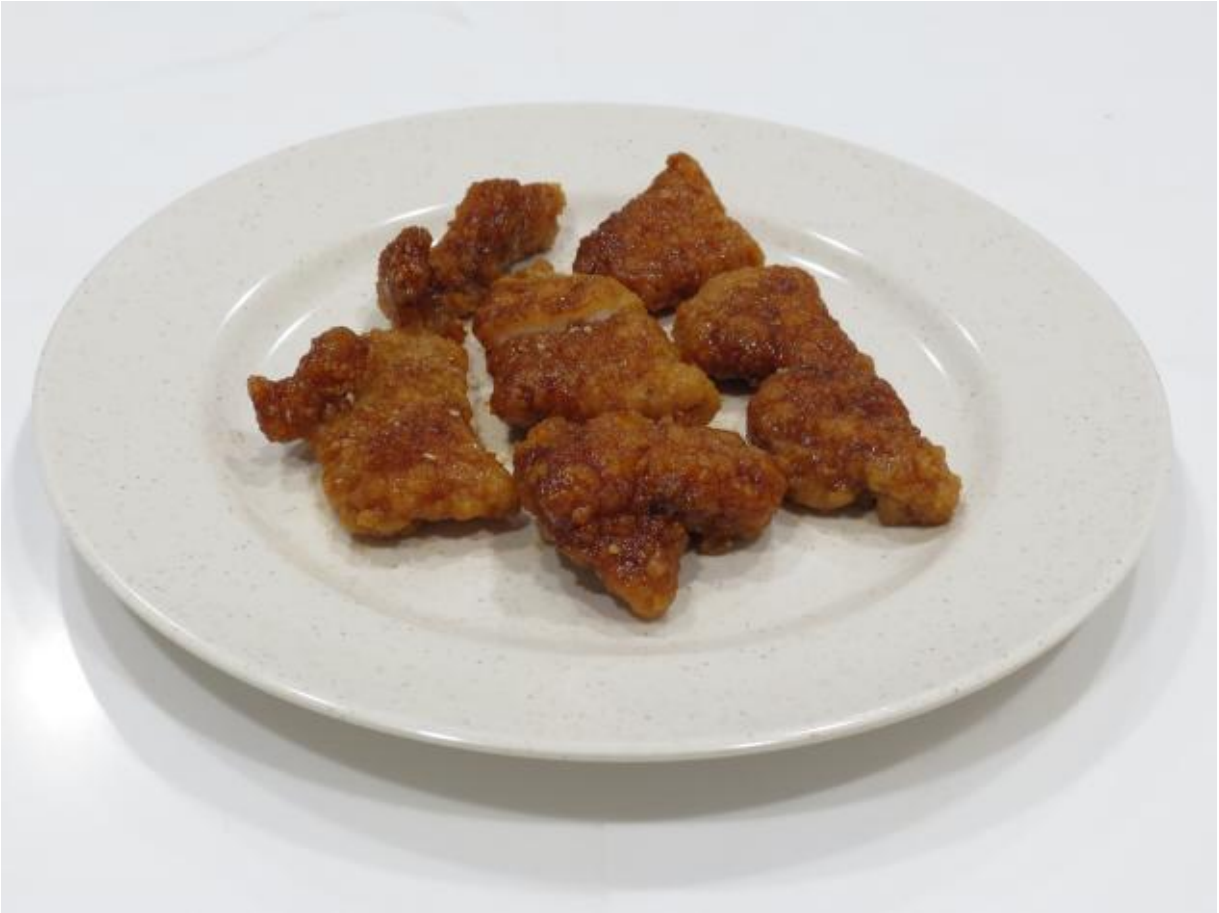
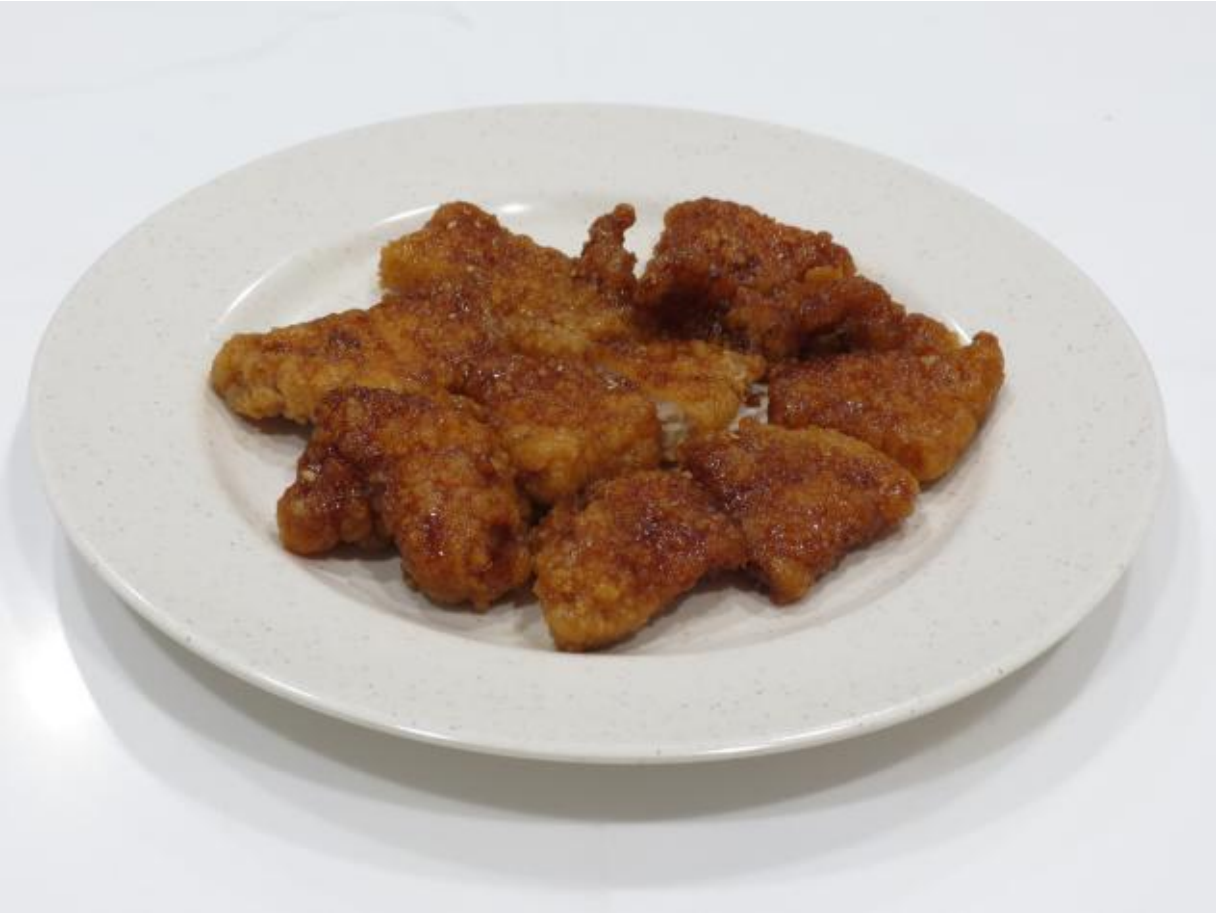

Pork, sliced (FA0123)

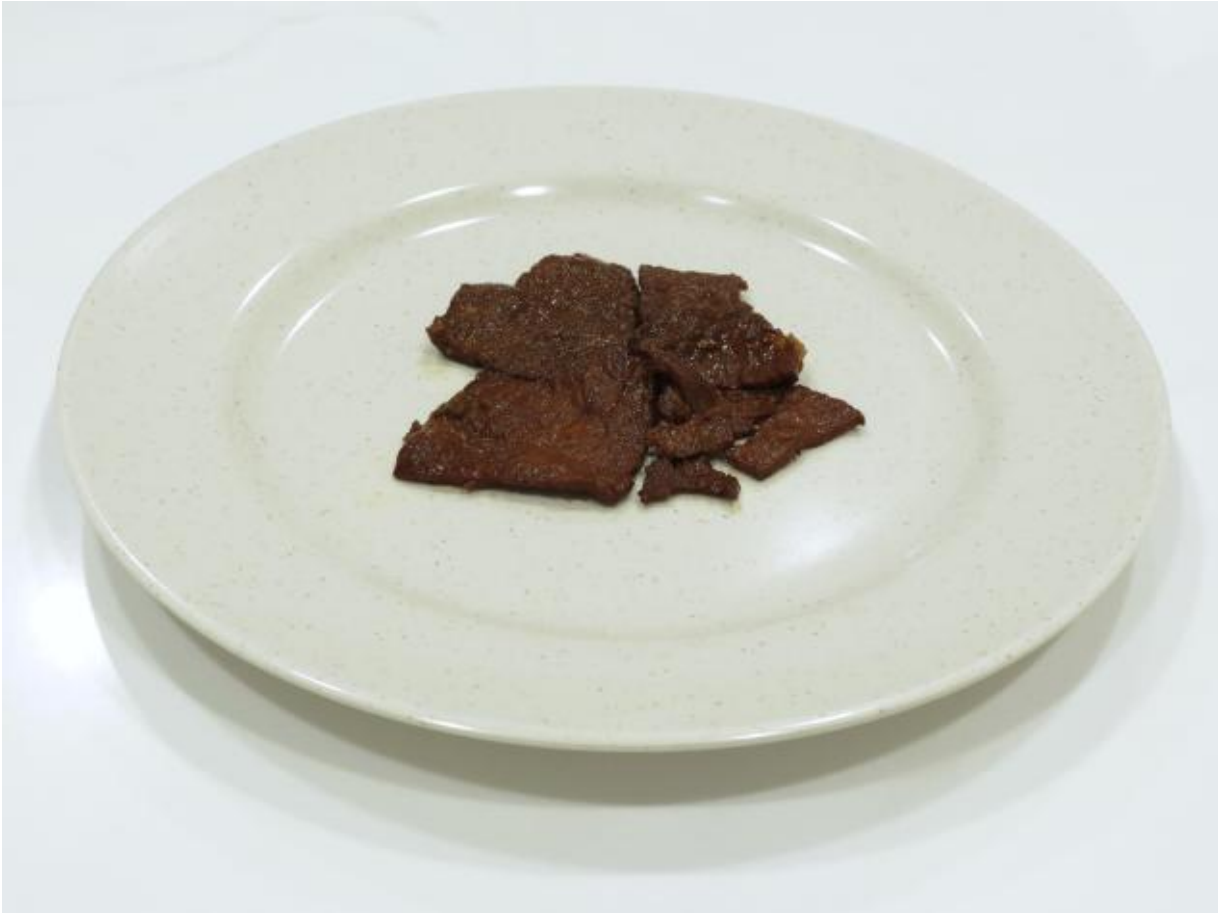
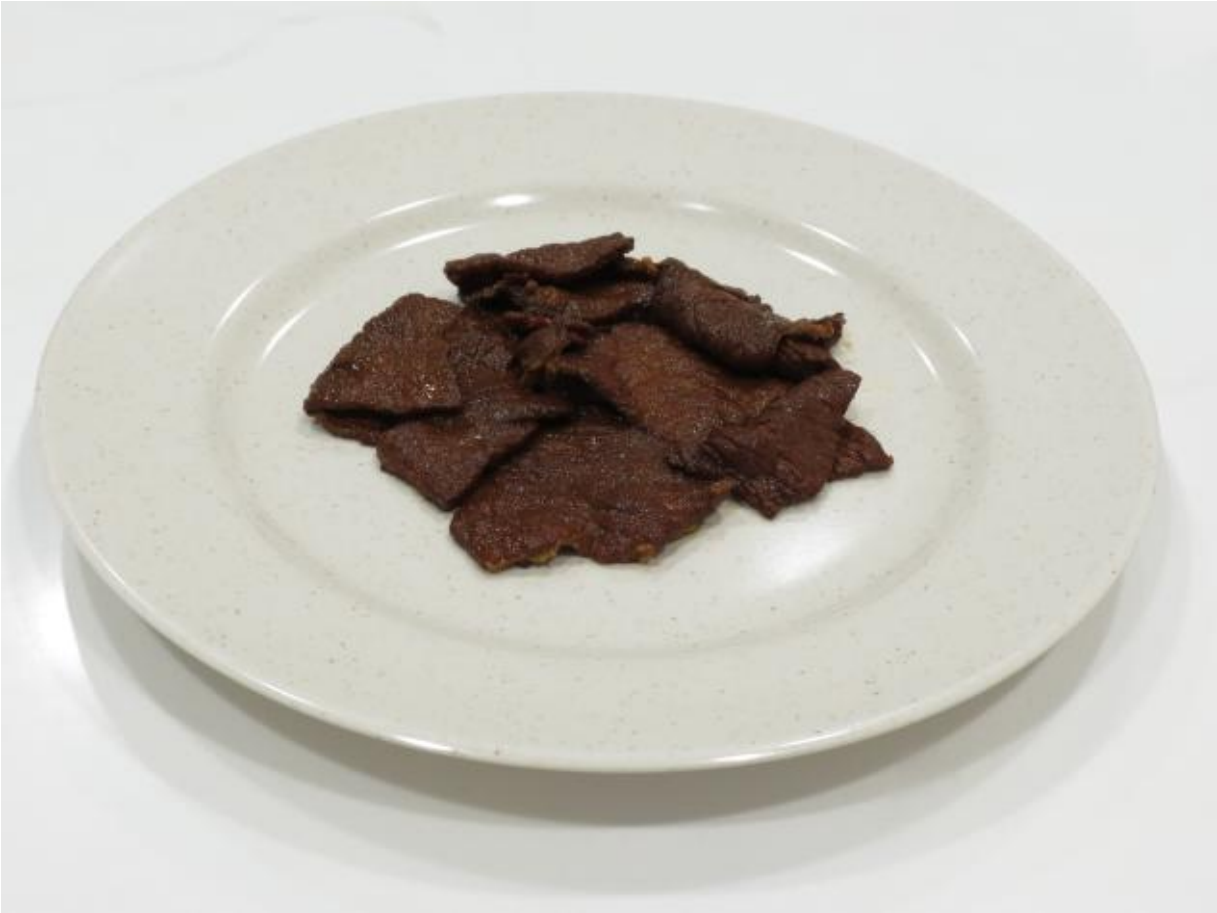
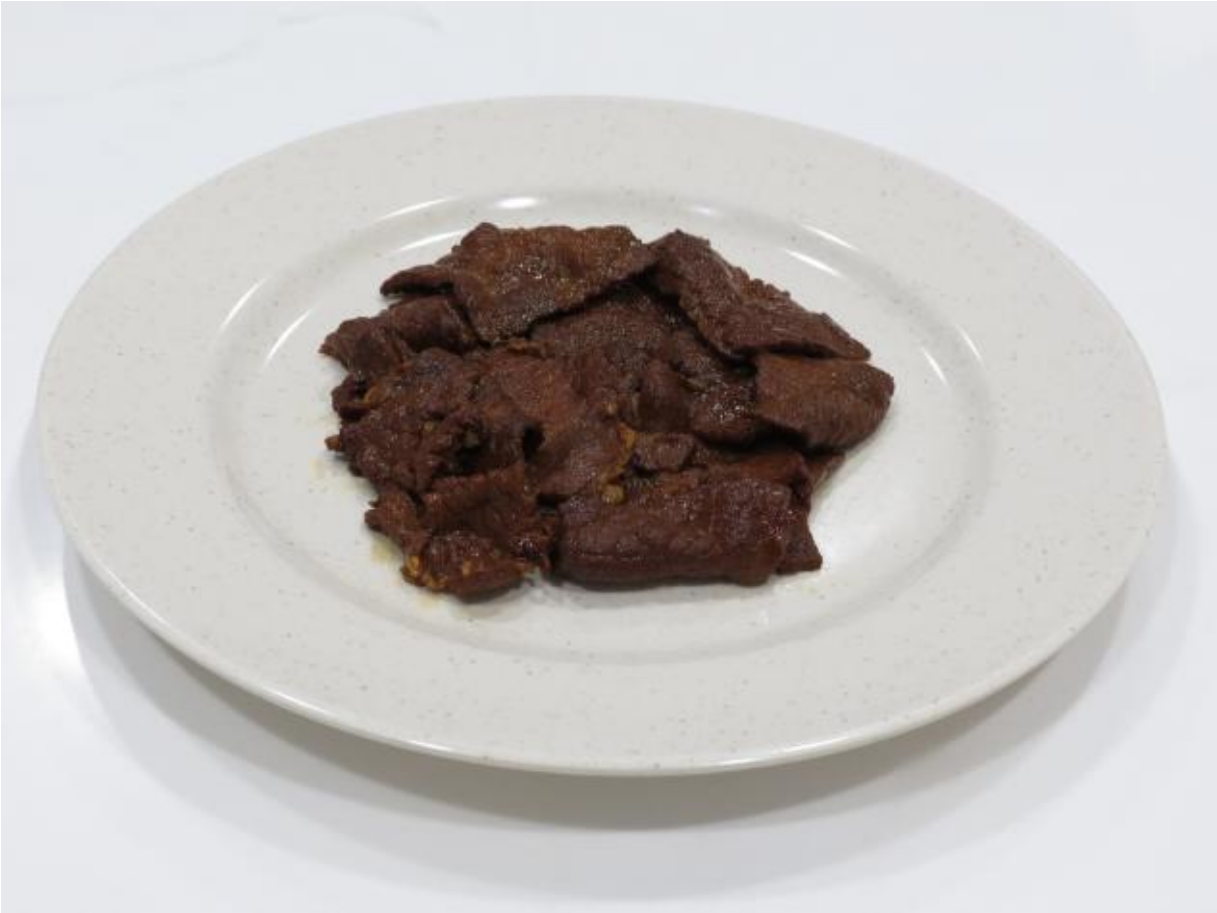
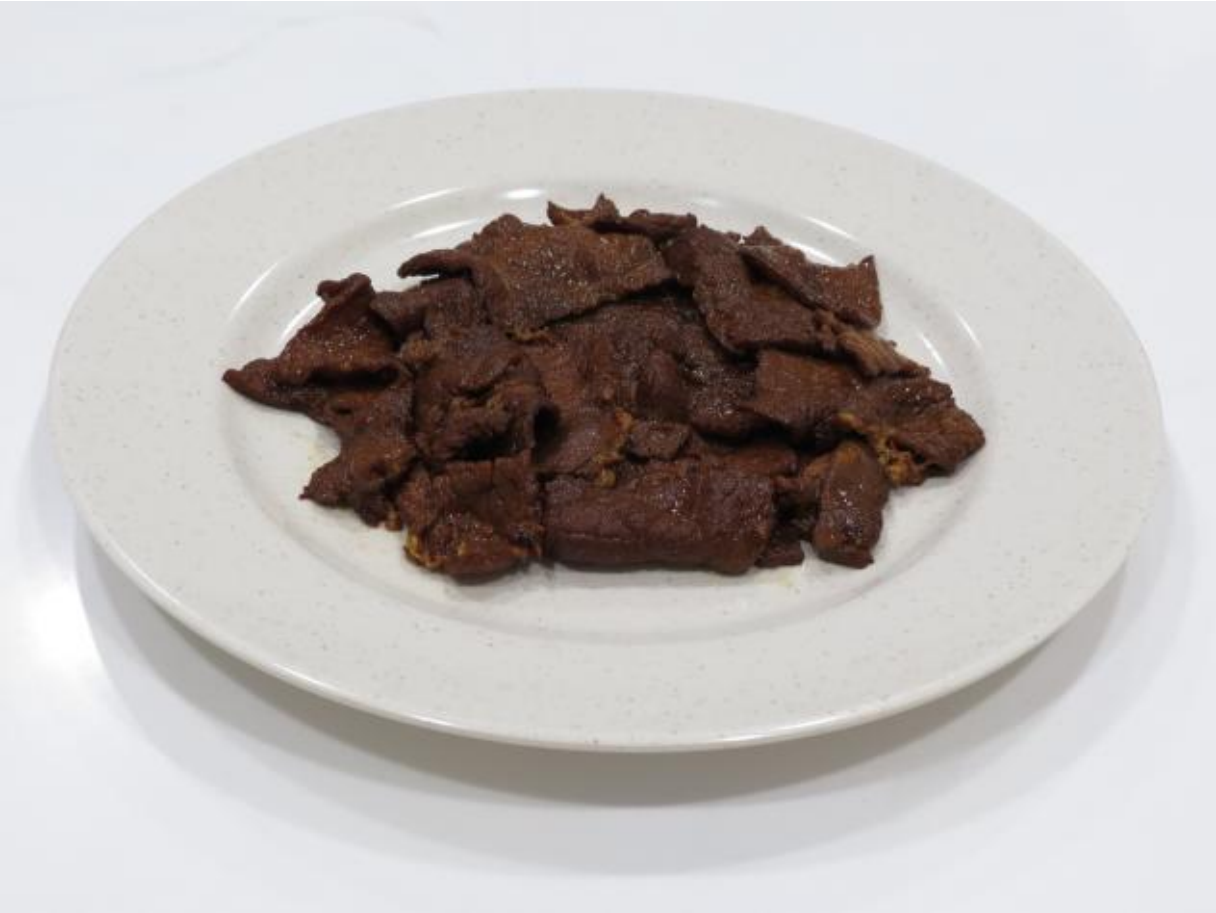

Cashew (FA0084)

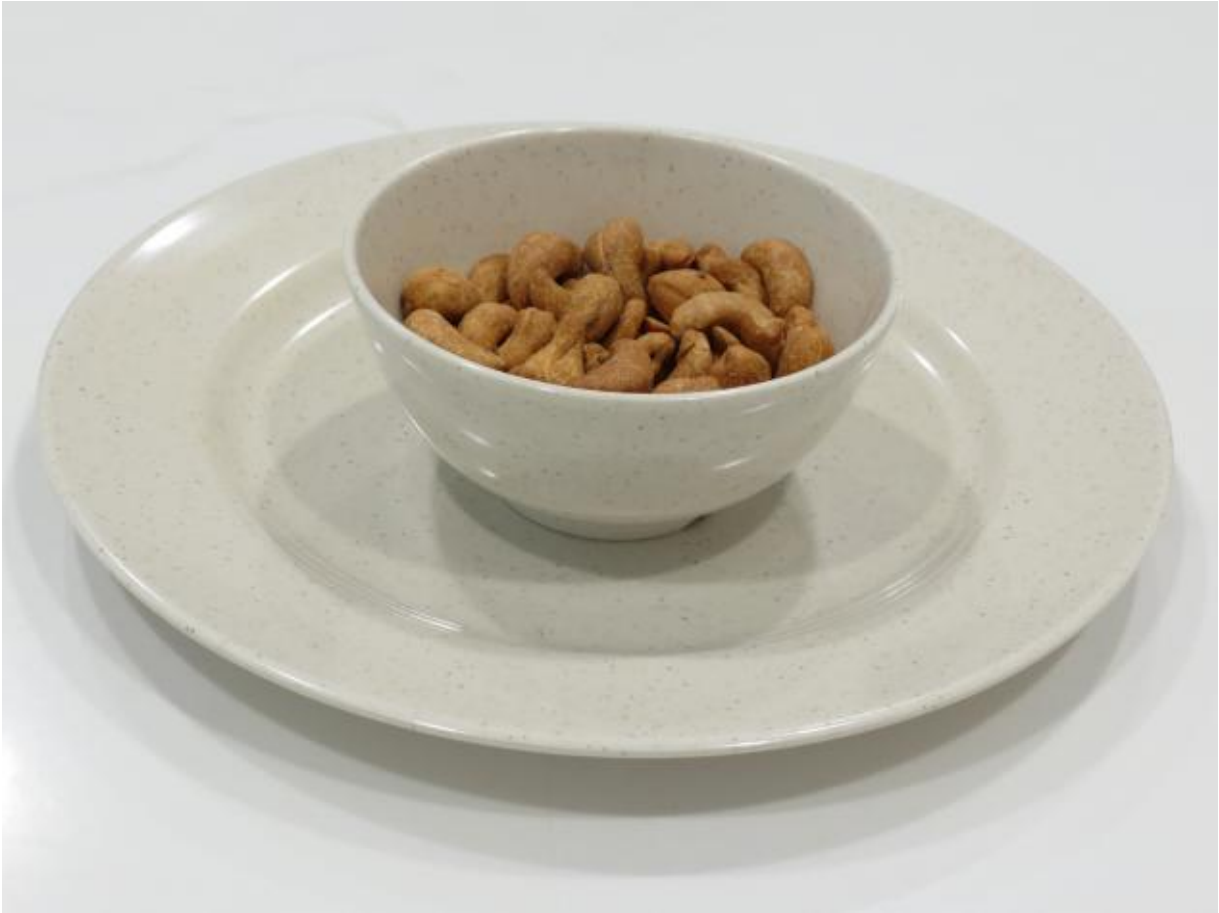

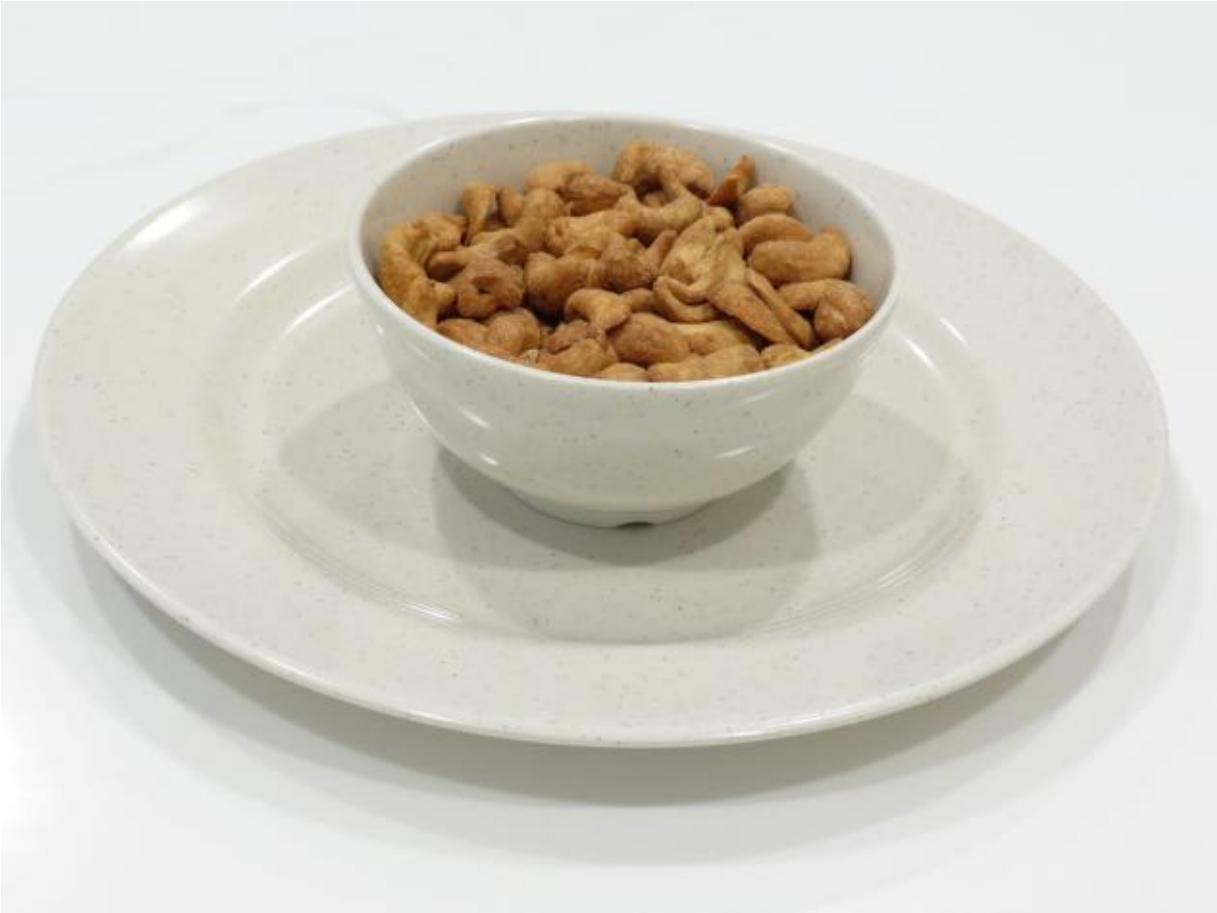

Peanut (FA0105)

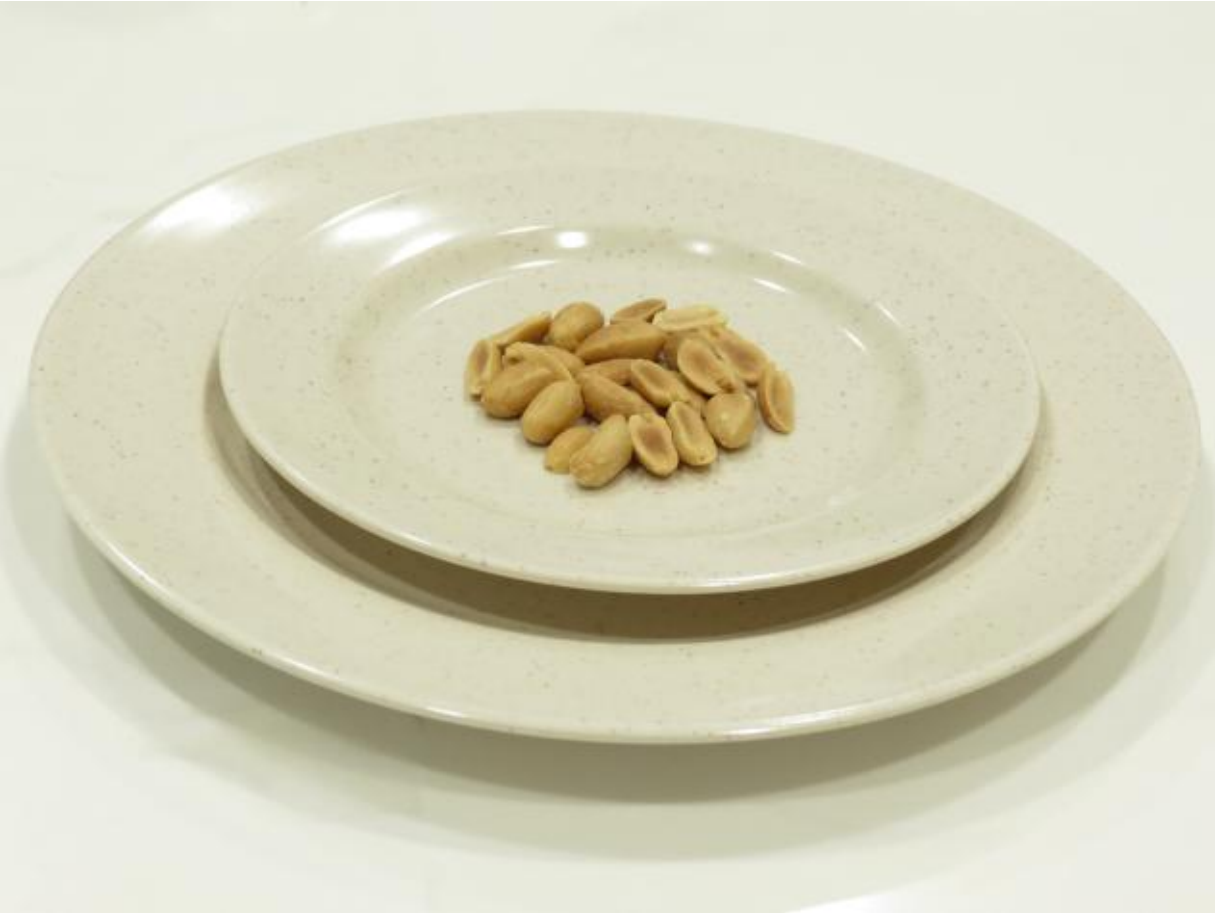

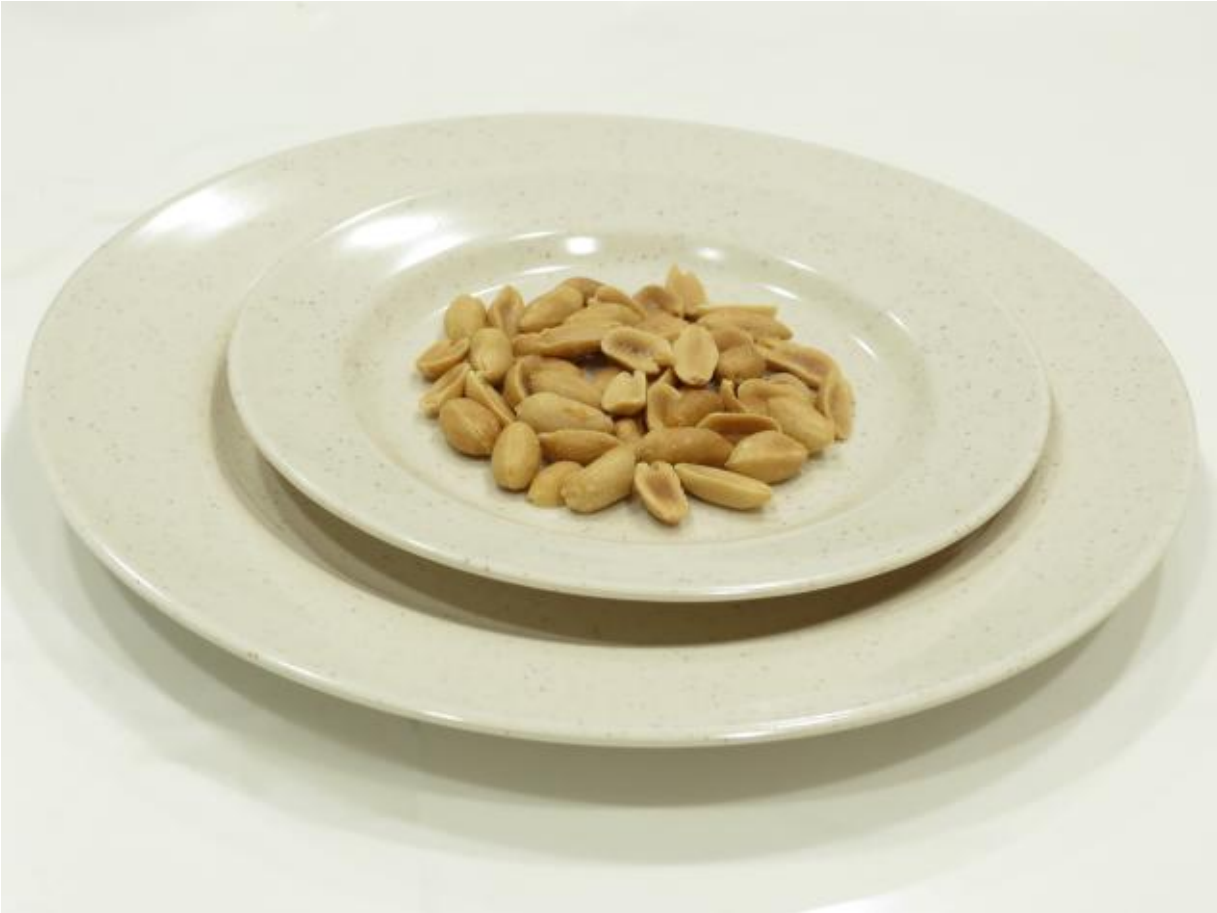

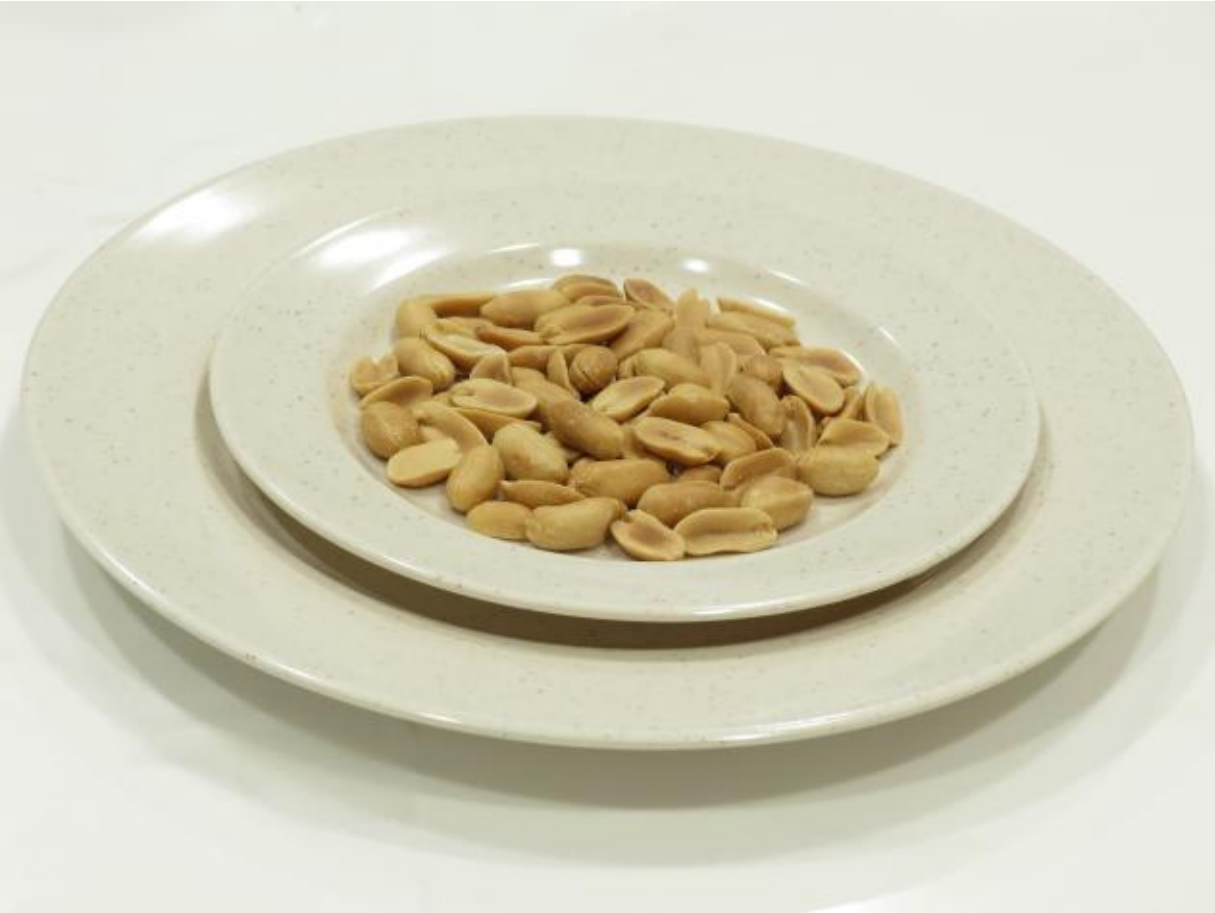

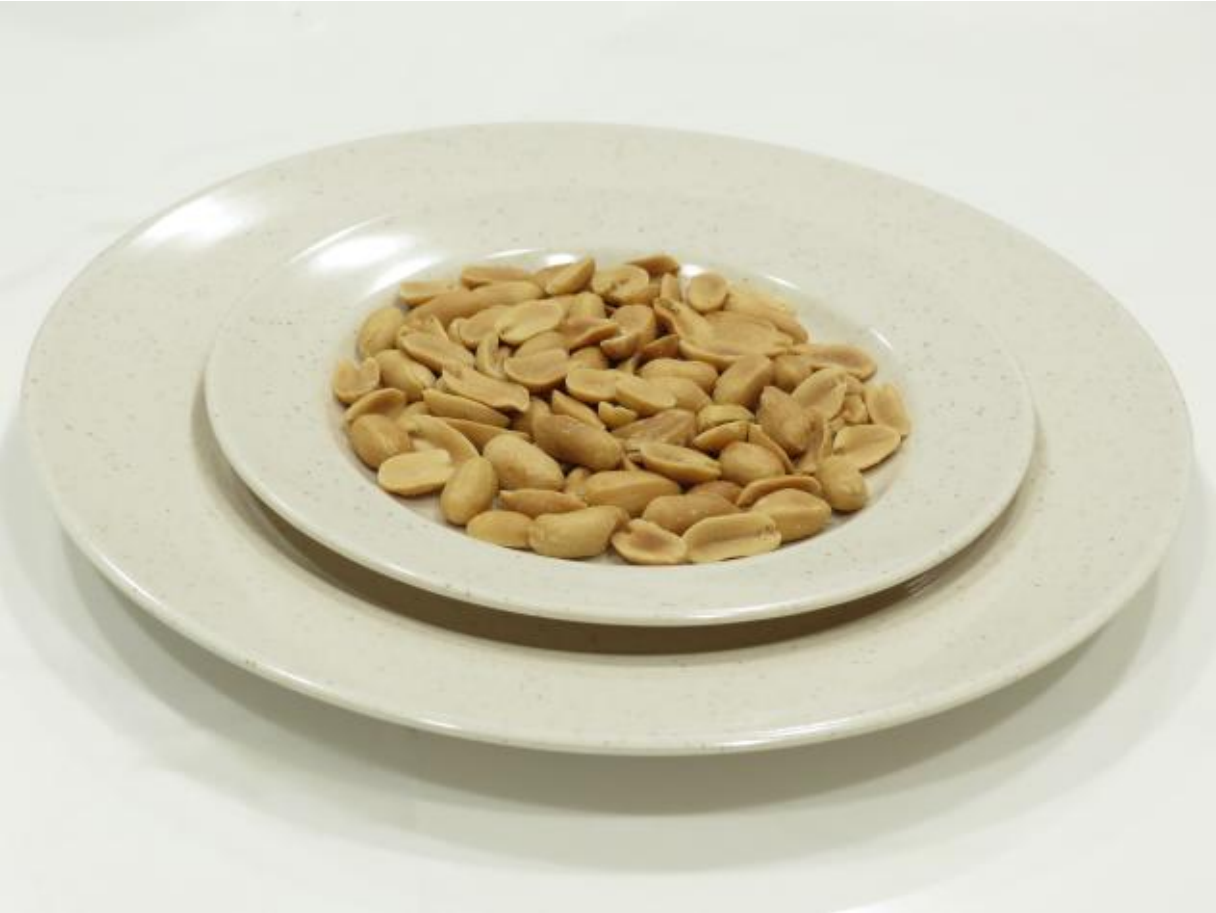

Cuttlefish snack, stick (FA0010)

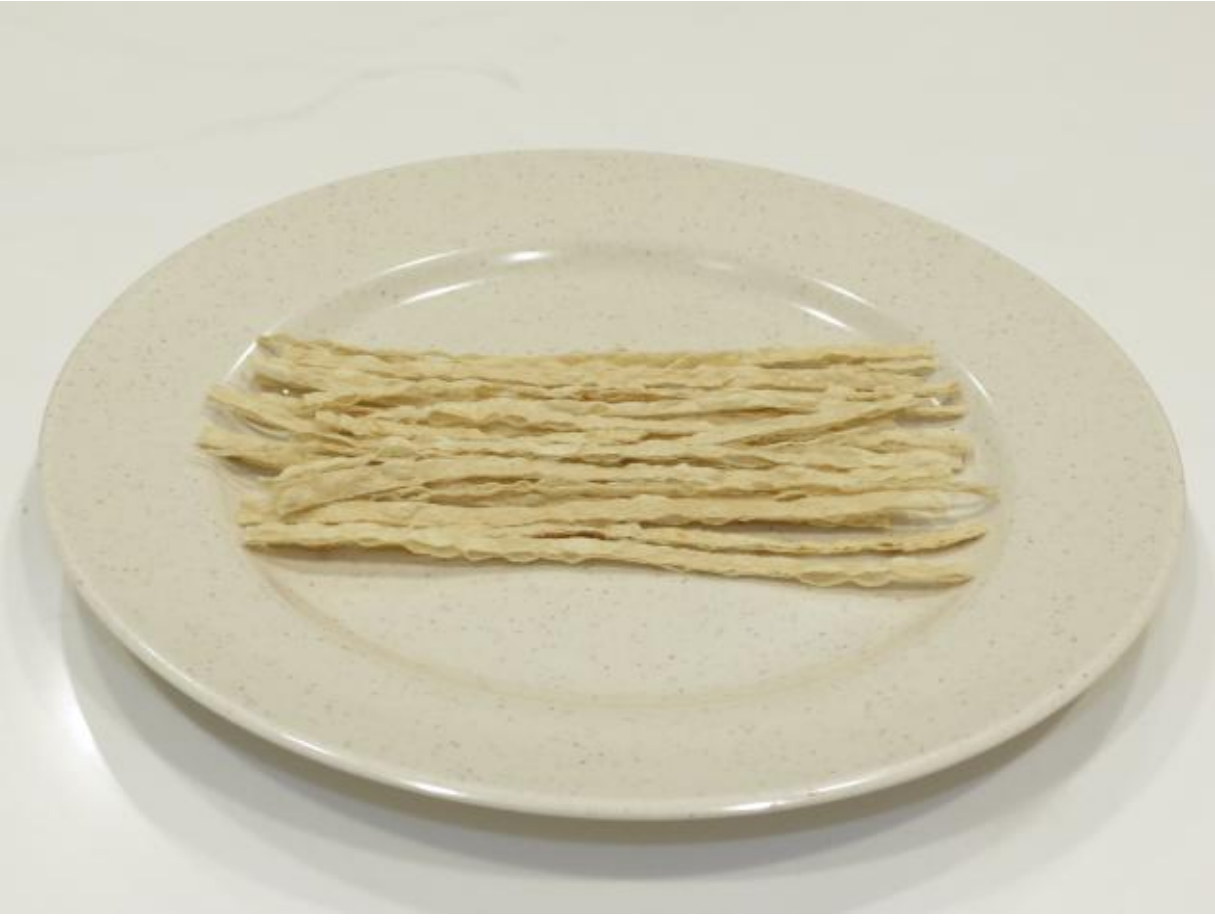

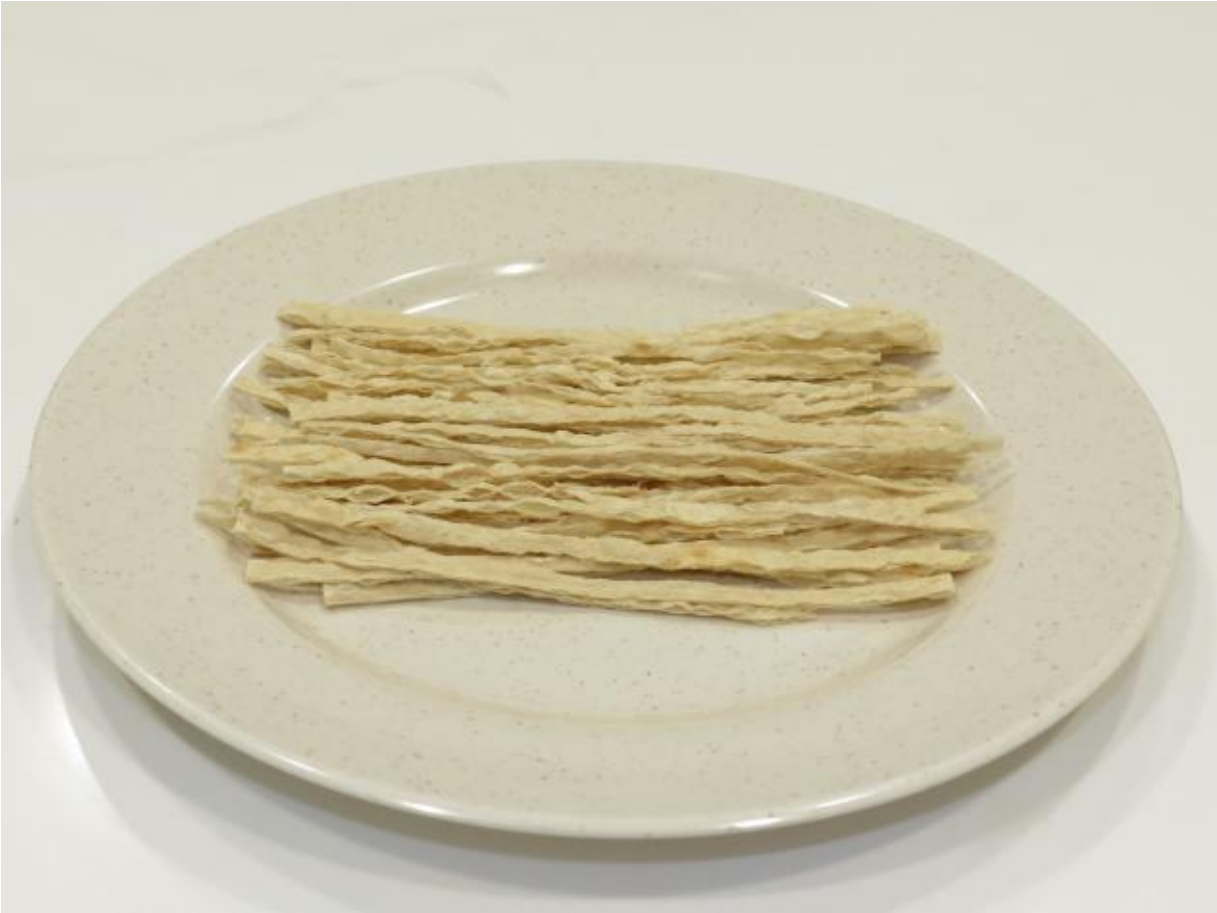

Popcorn (FA0002)

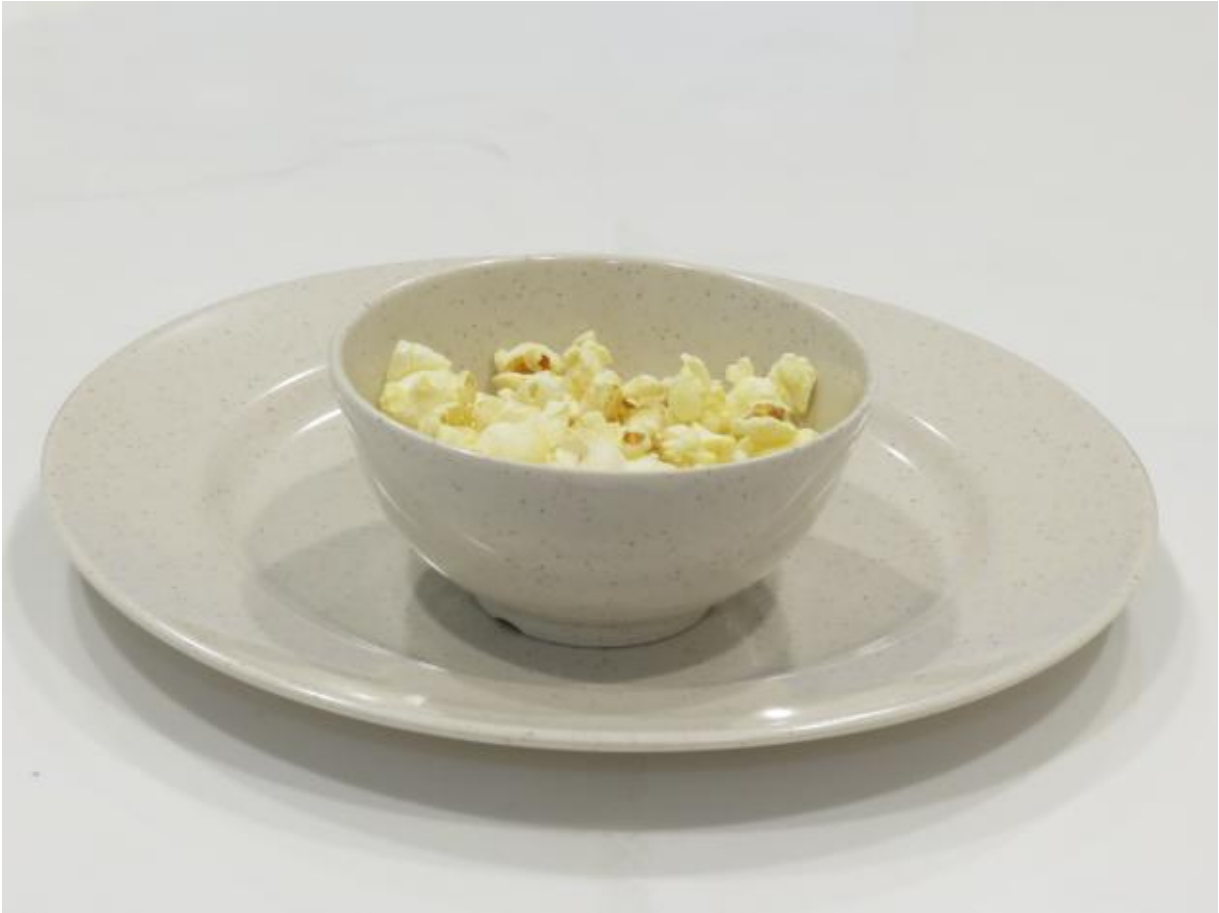

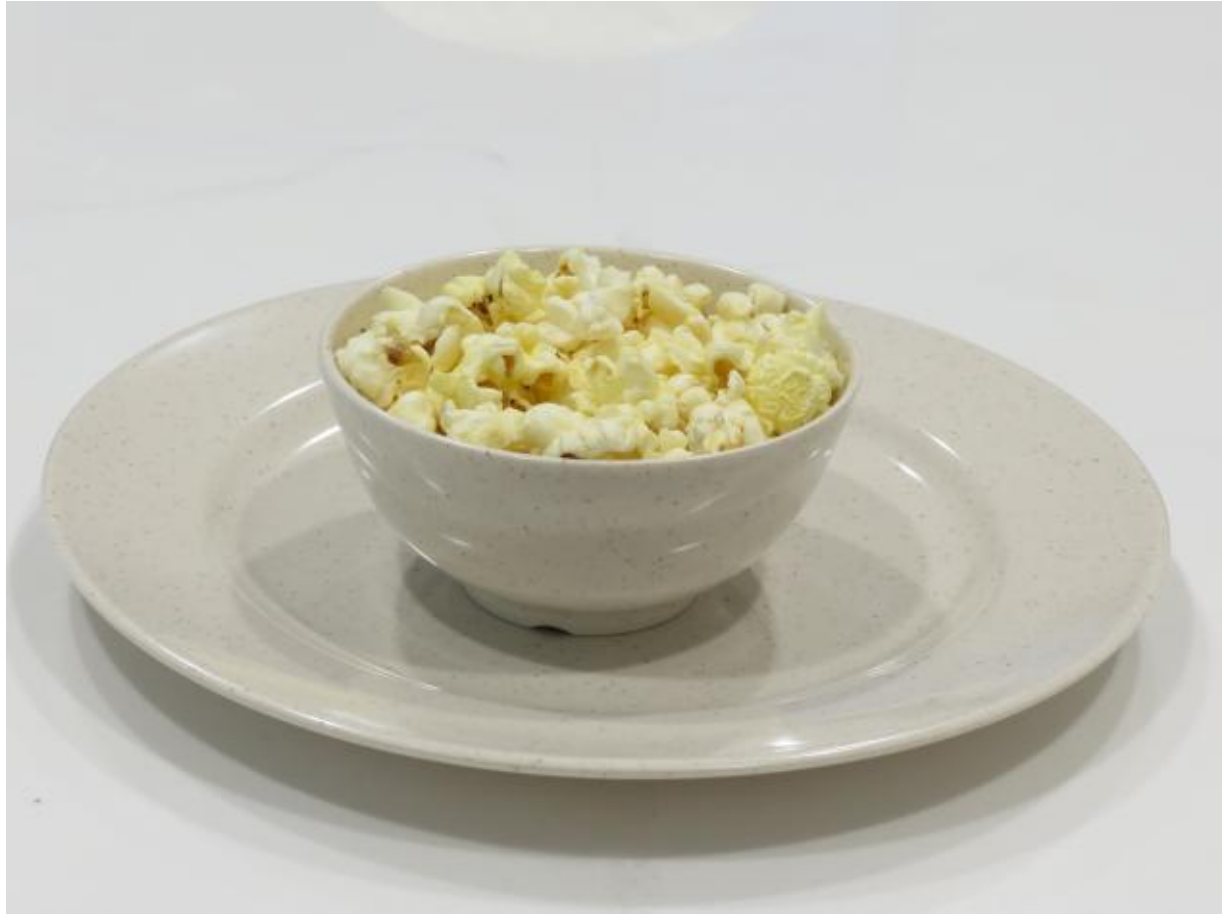

Potato chip (FA0189)

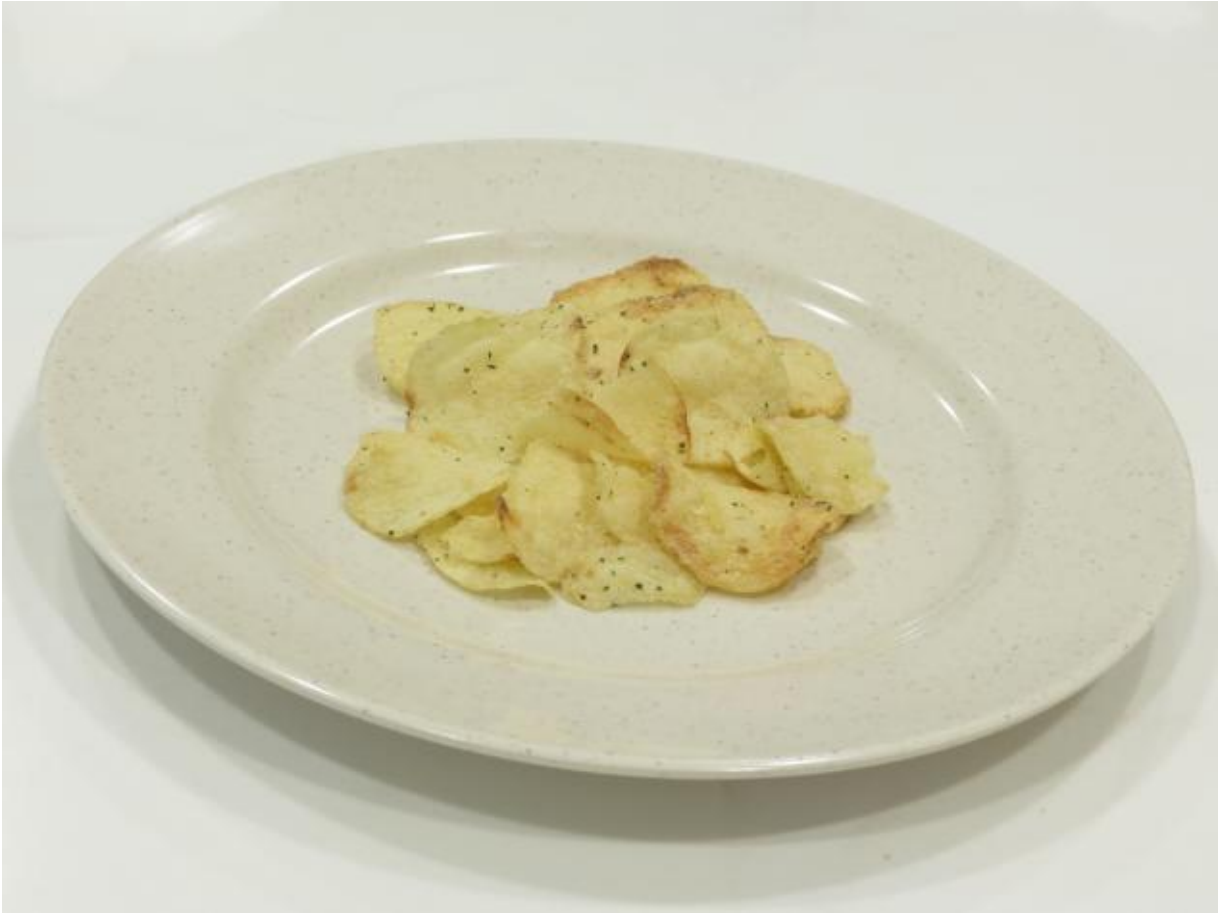

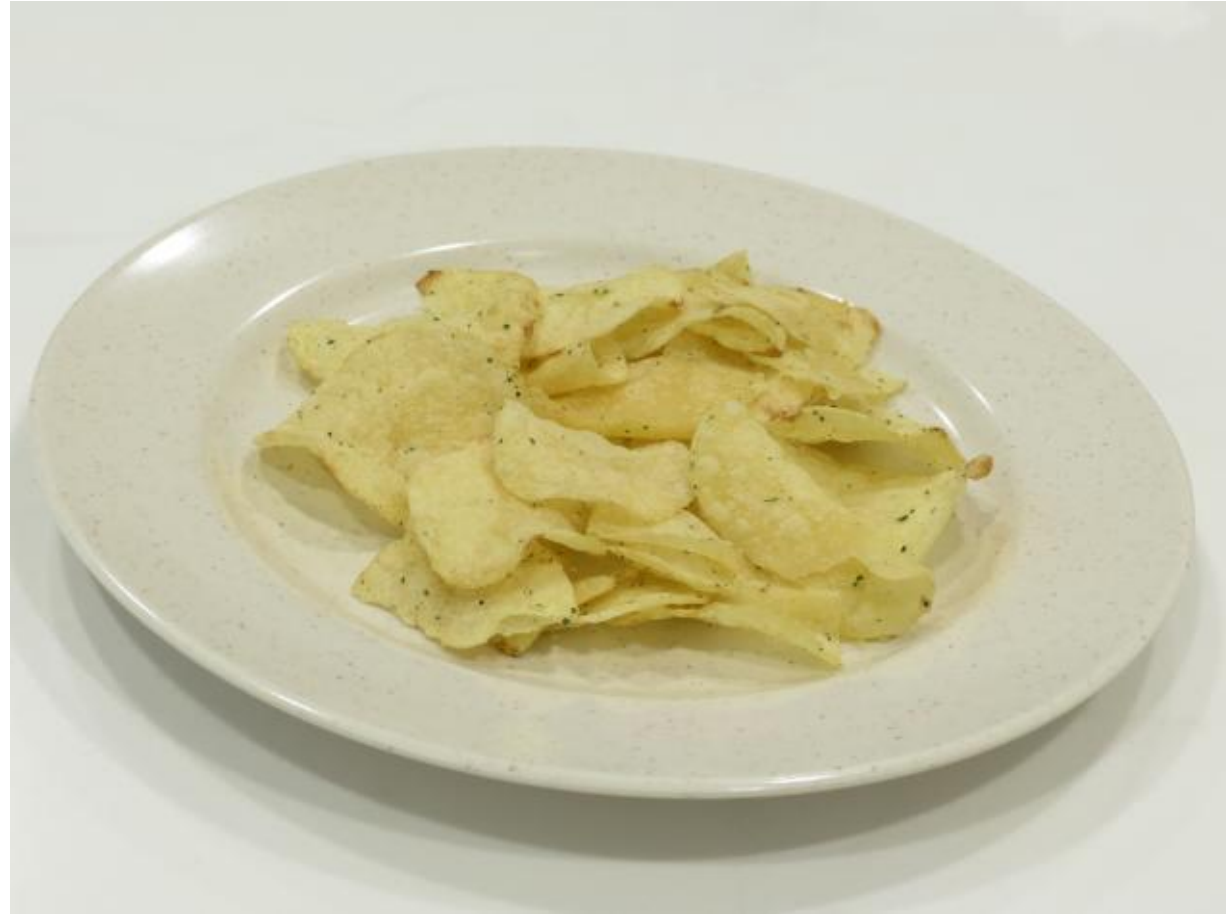

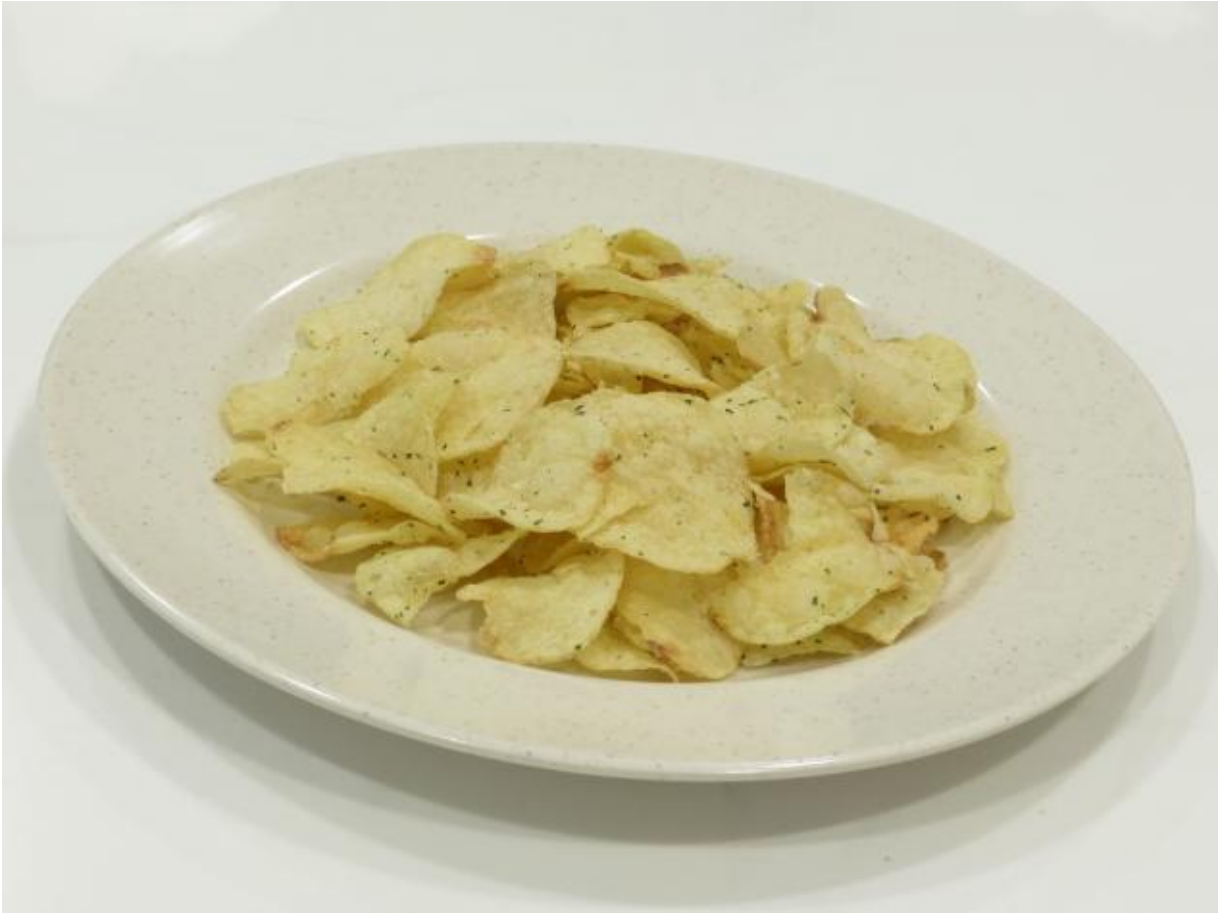

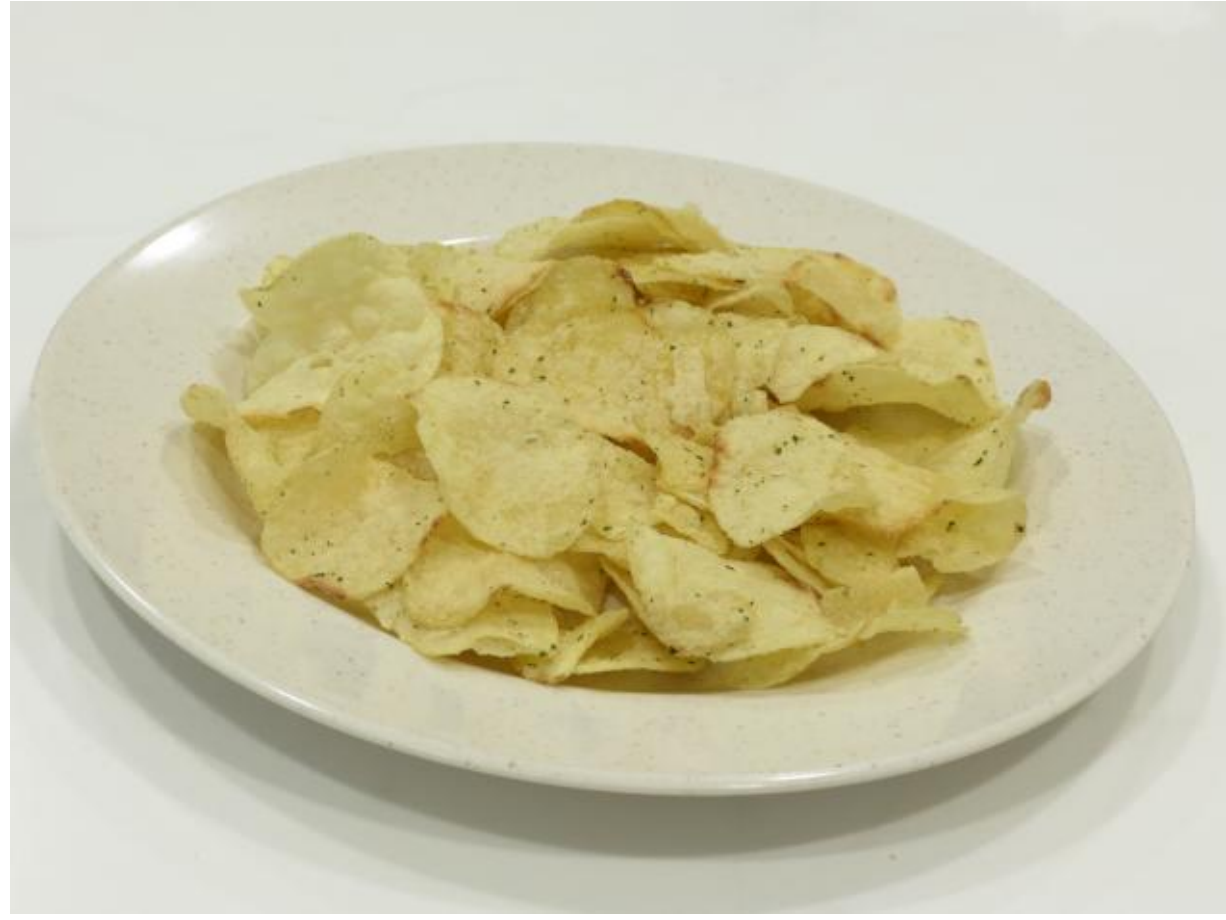

Mock meat, canned (FA0113)

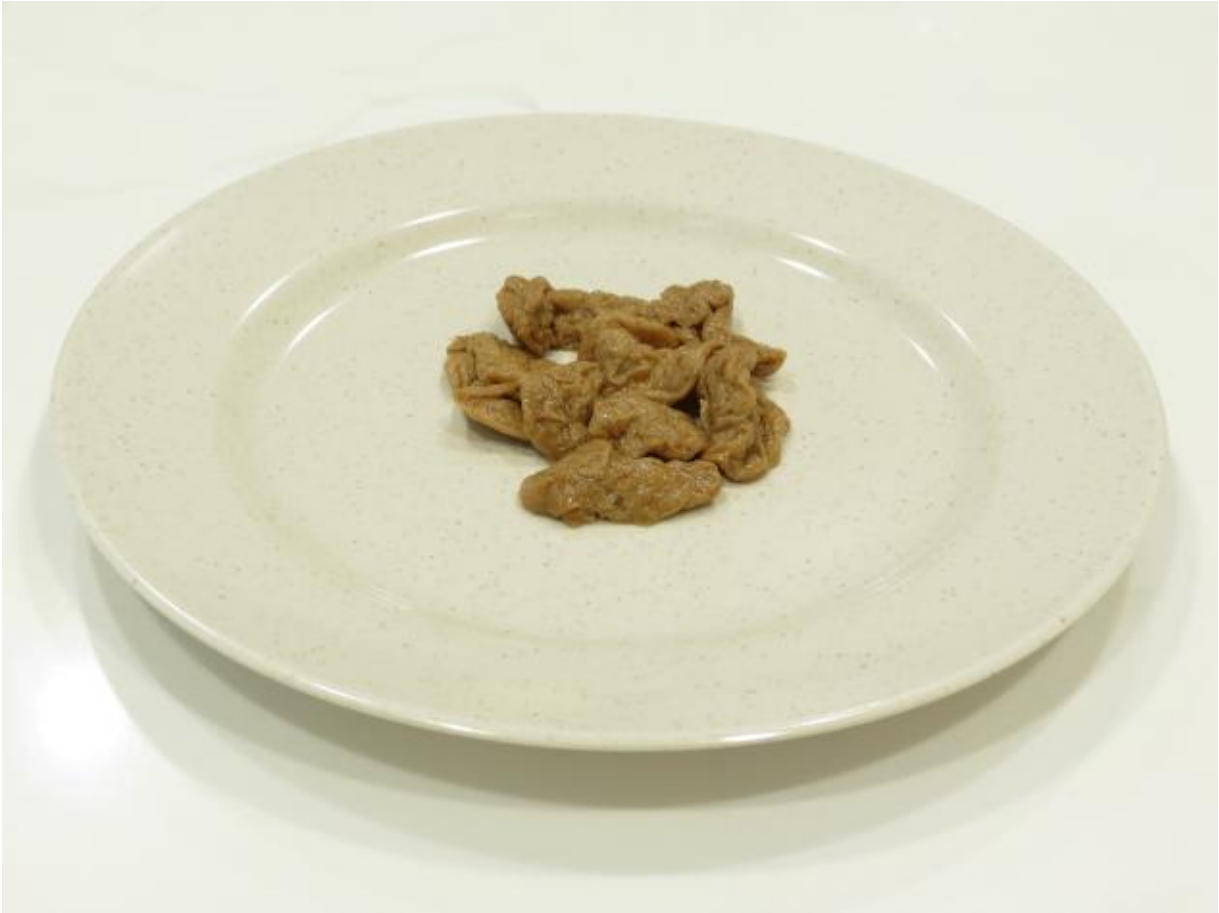
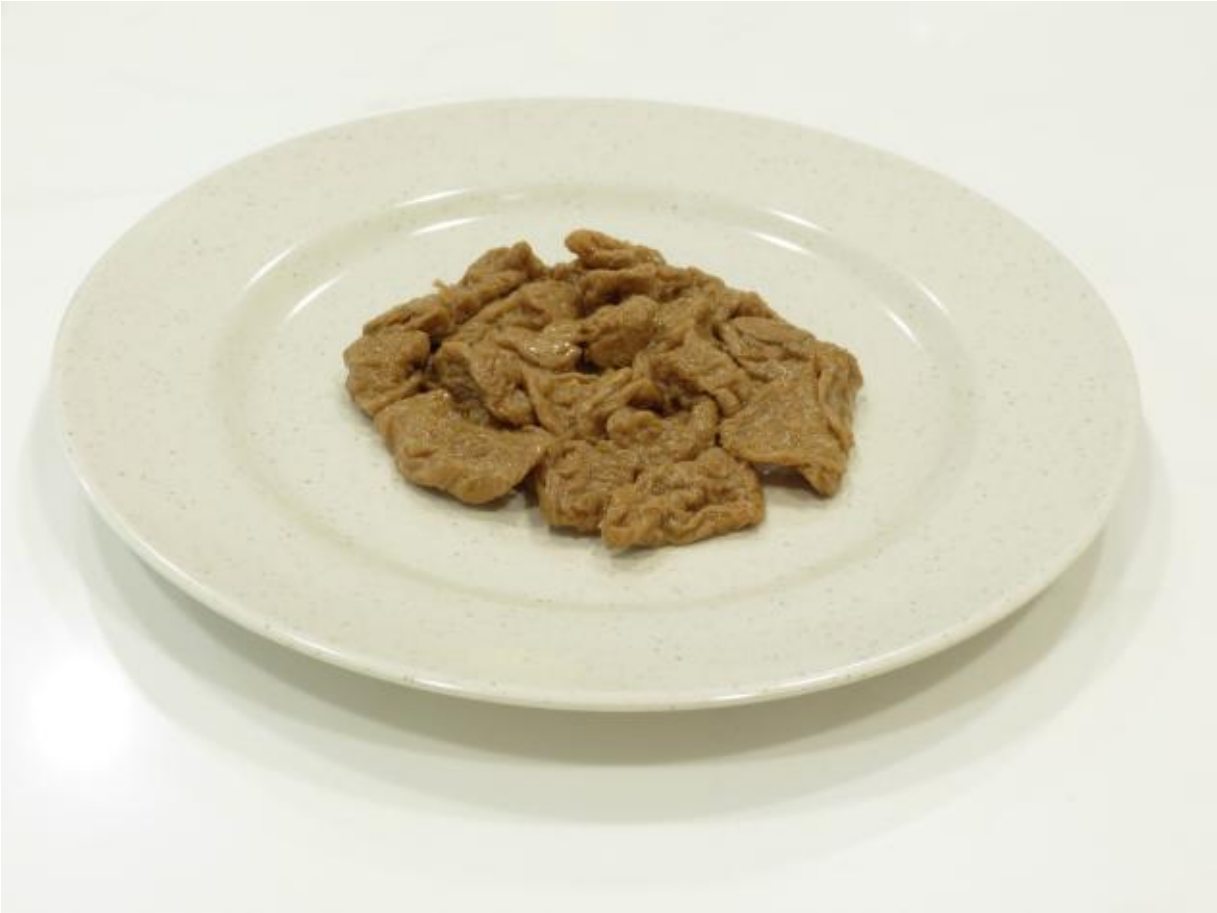
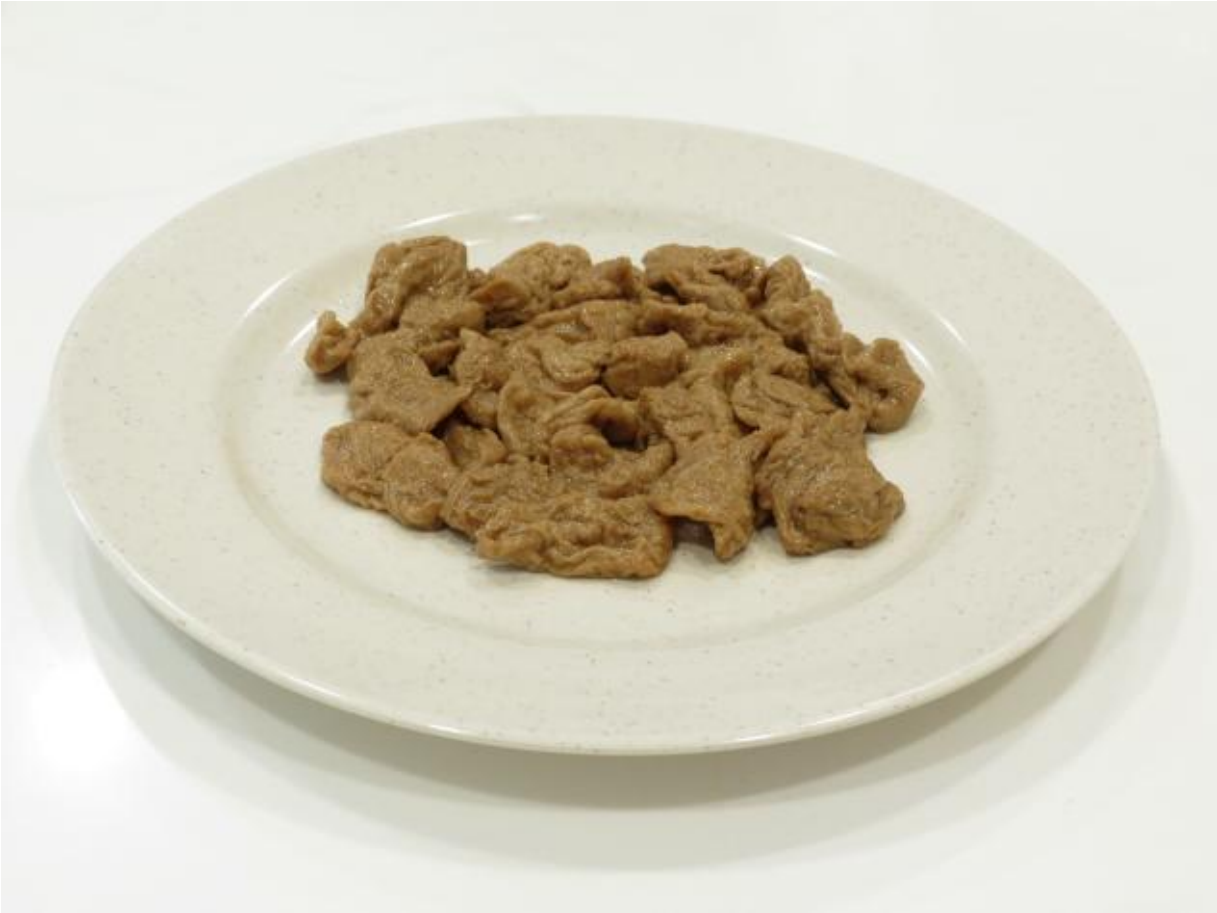
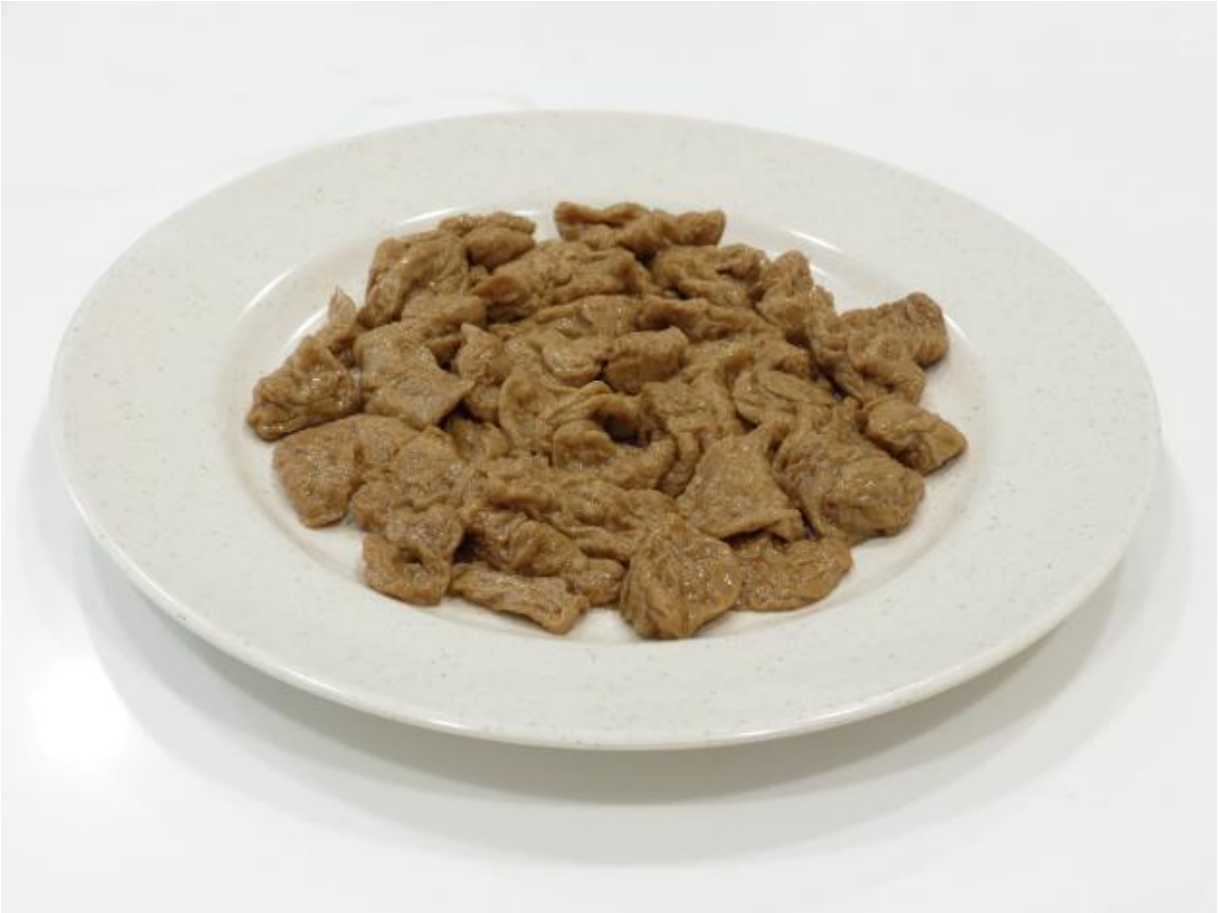

Broccoli, florets (FA0075)

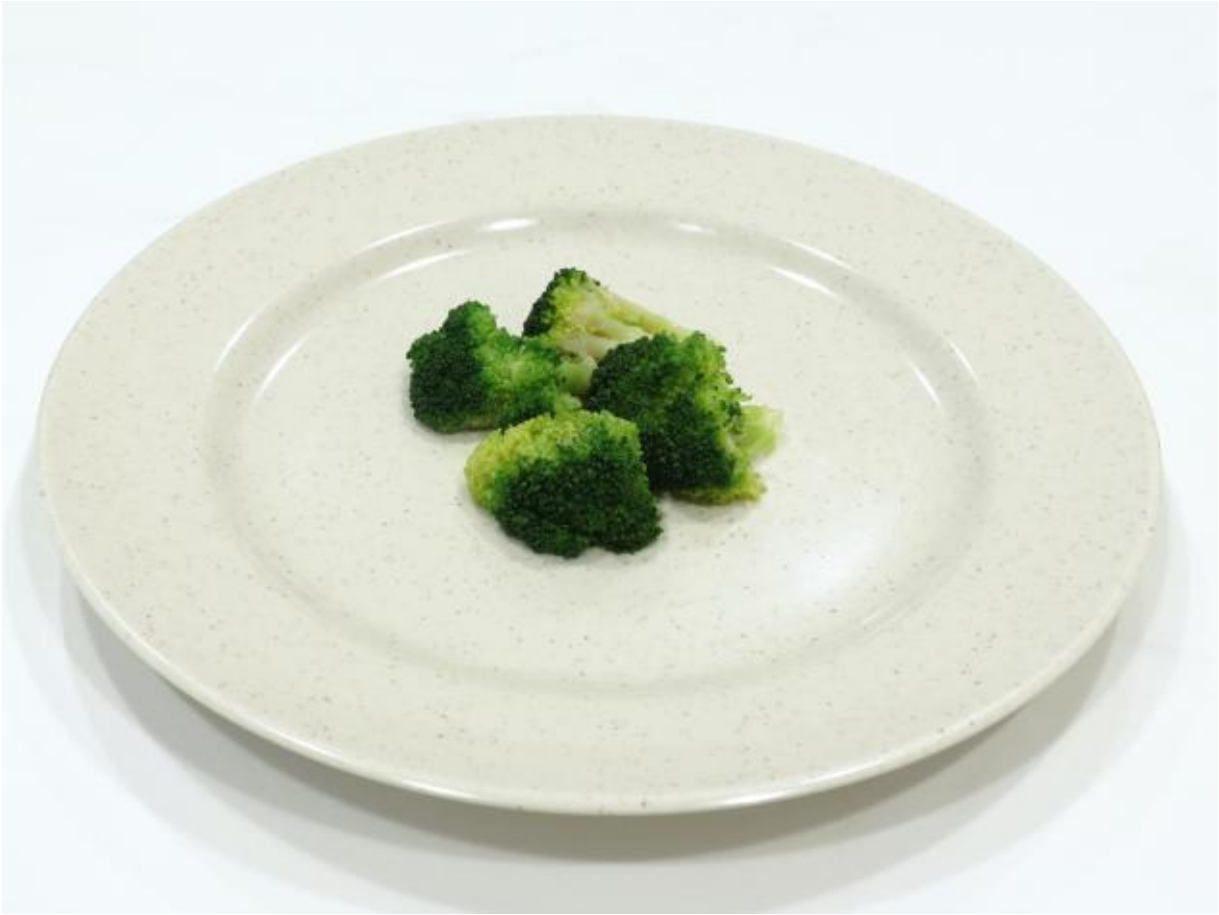
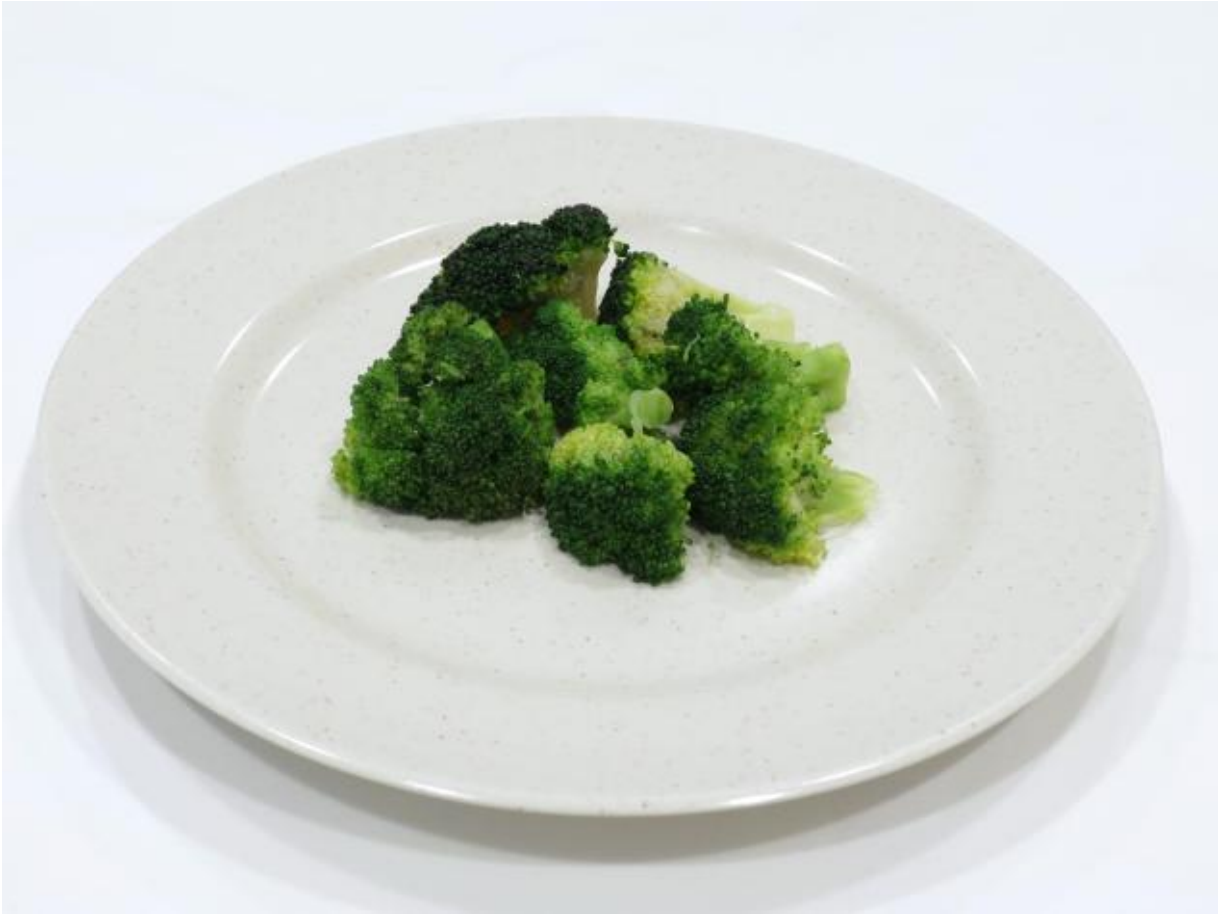
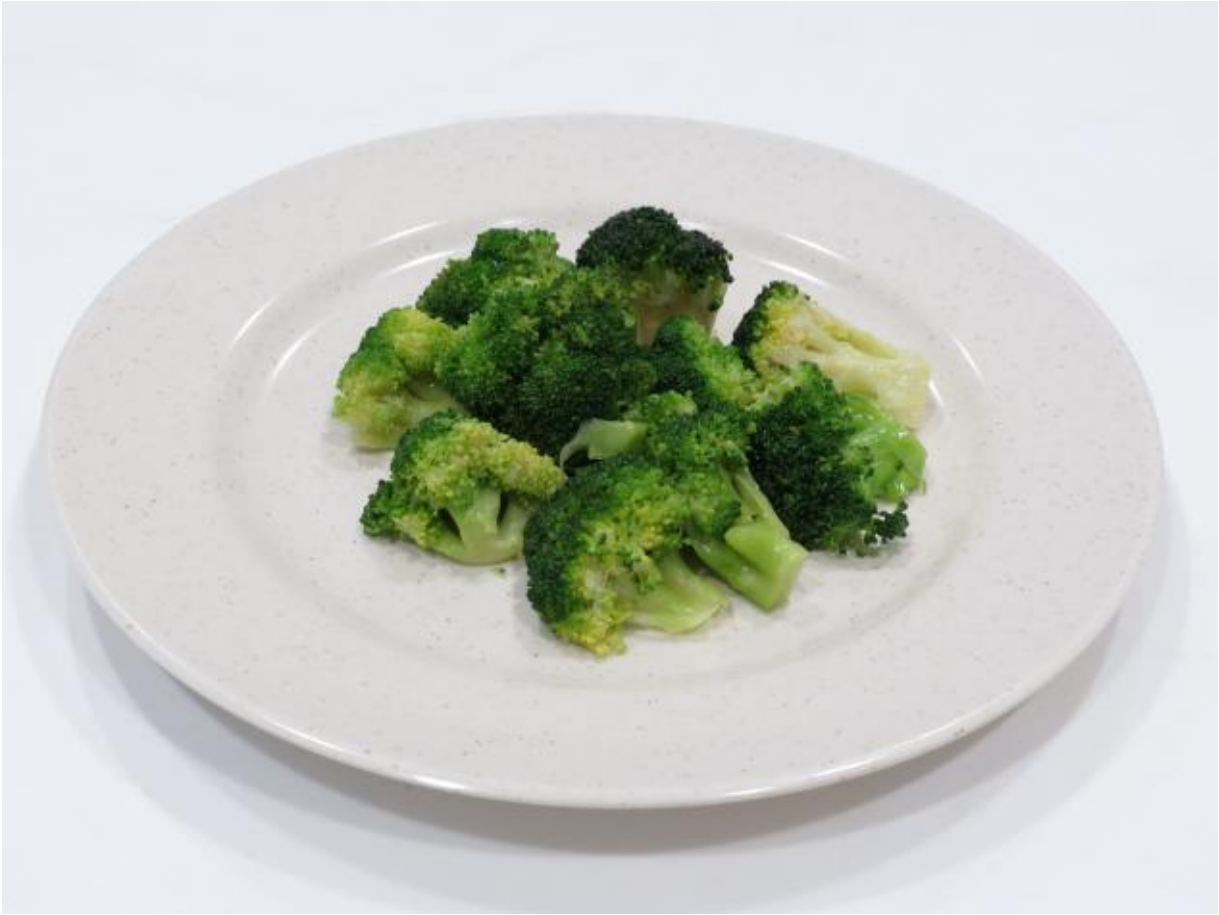
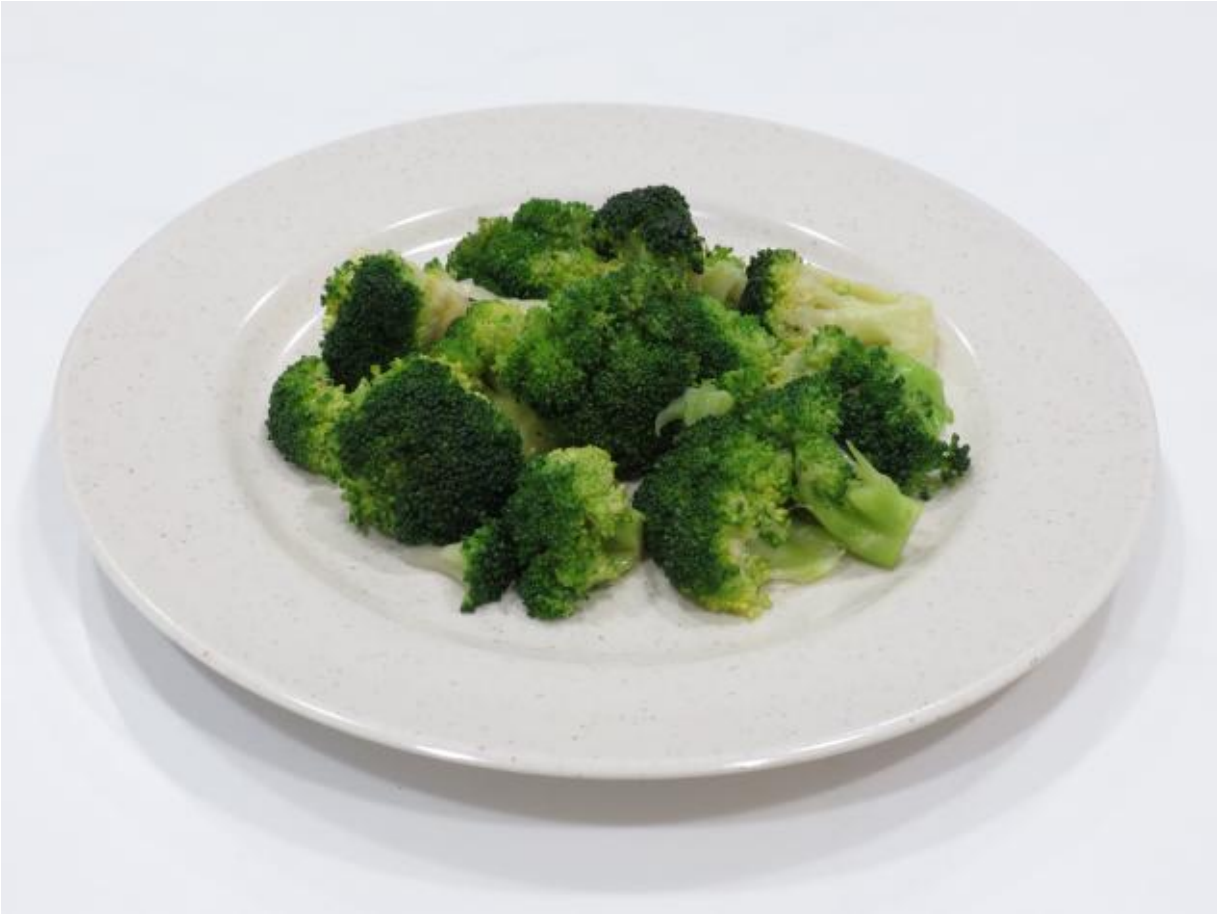

Cabbage, sliced (FA0130)

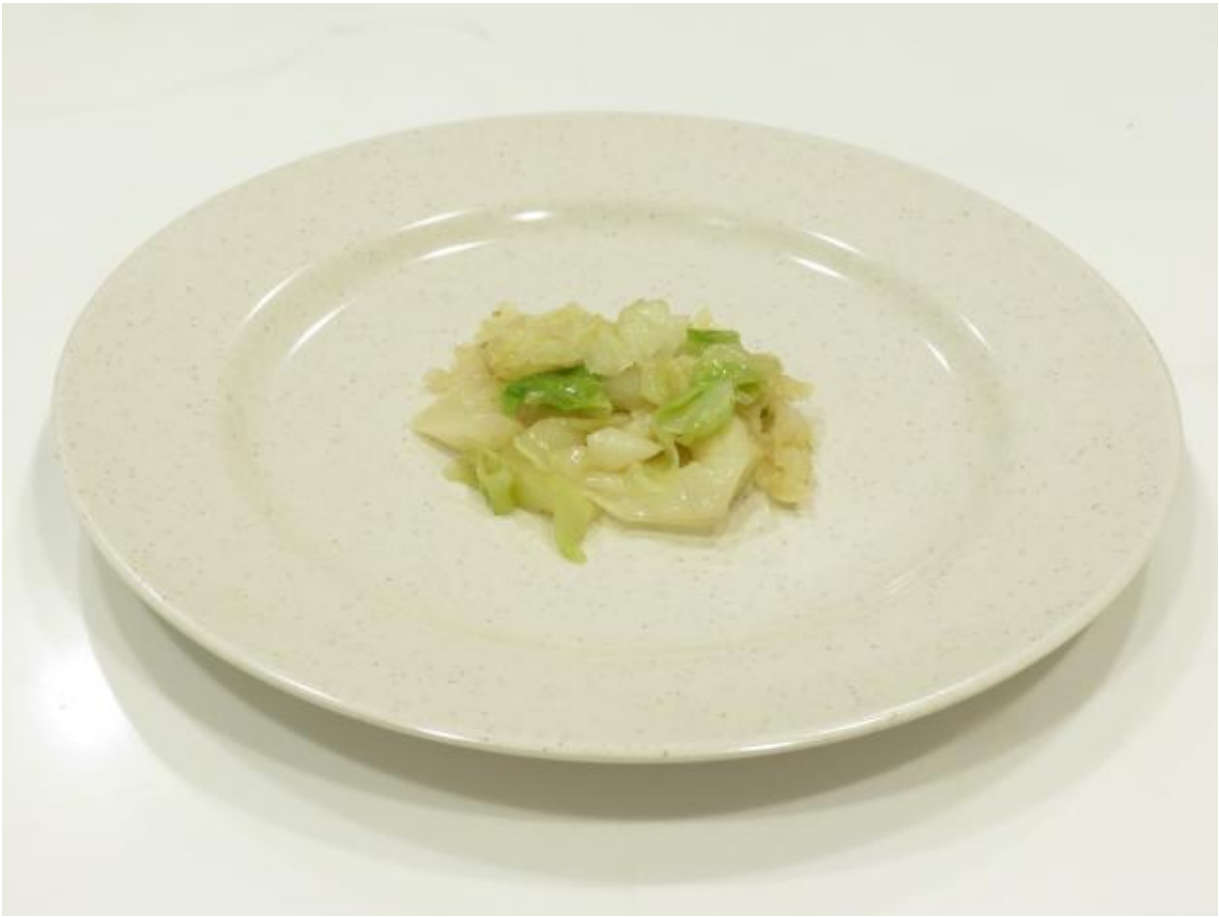

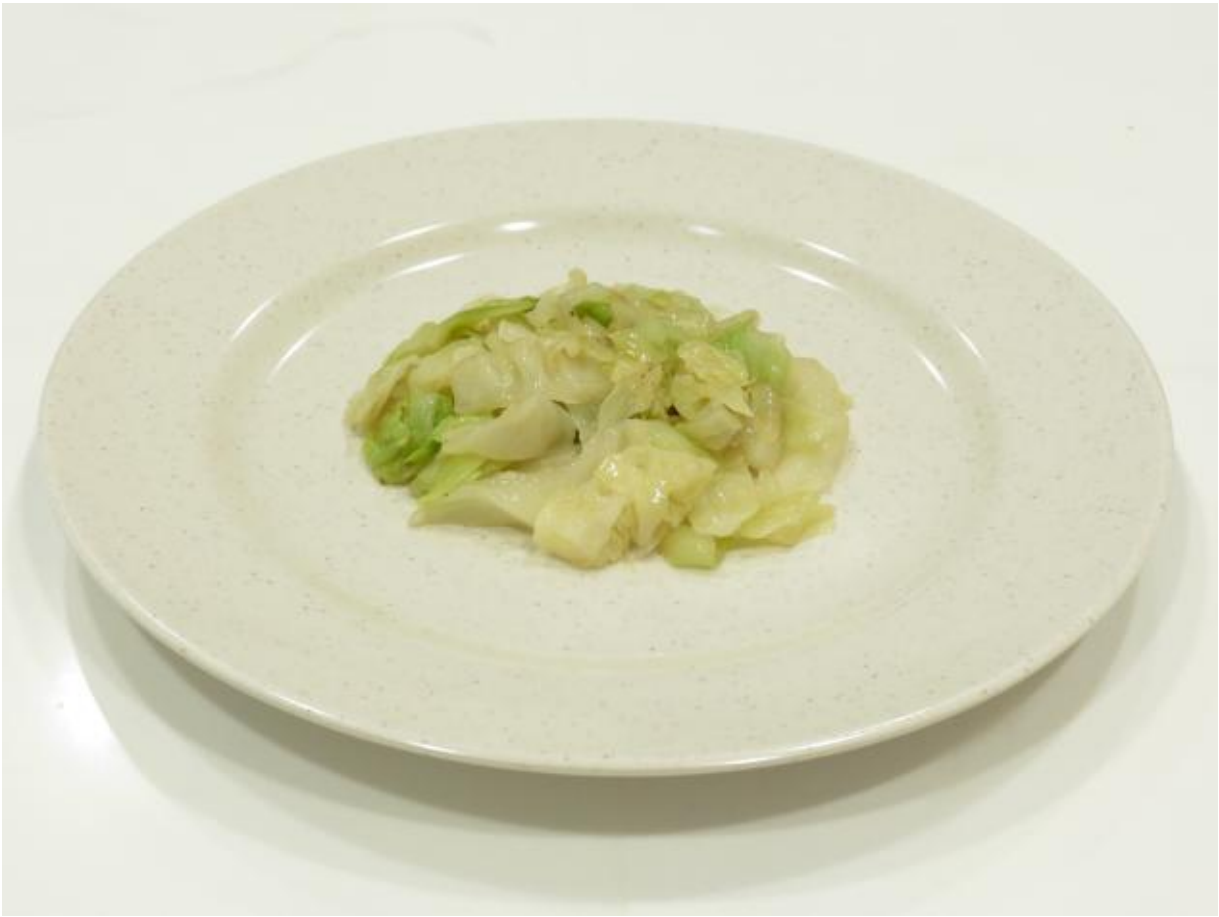

Bittergourd, sliced (FA0129)

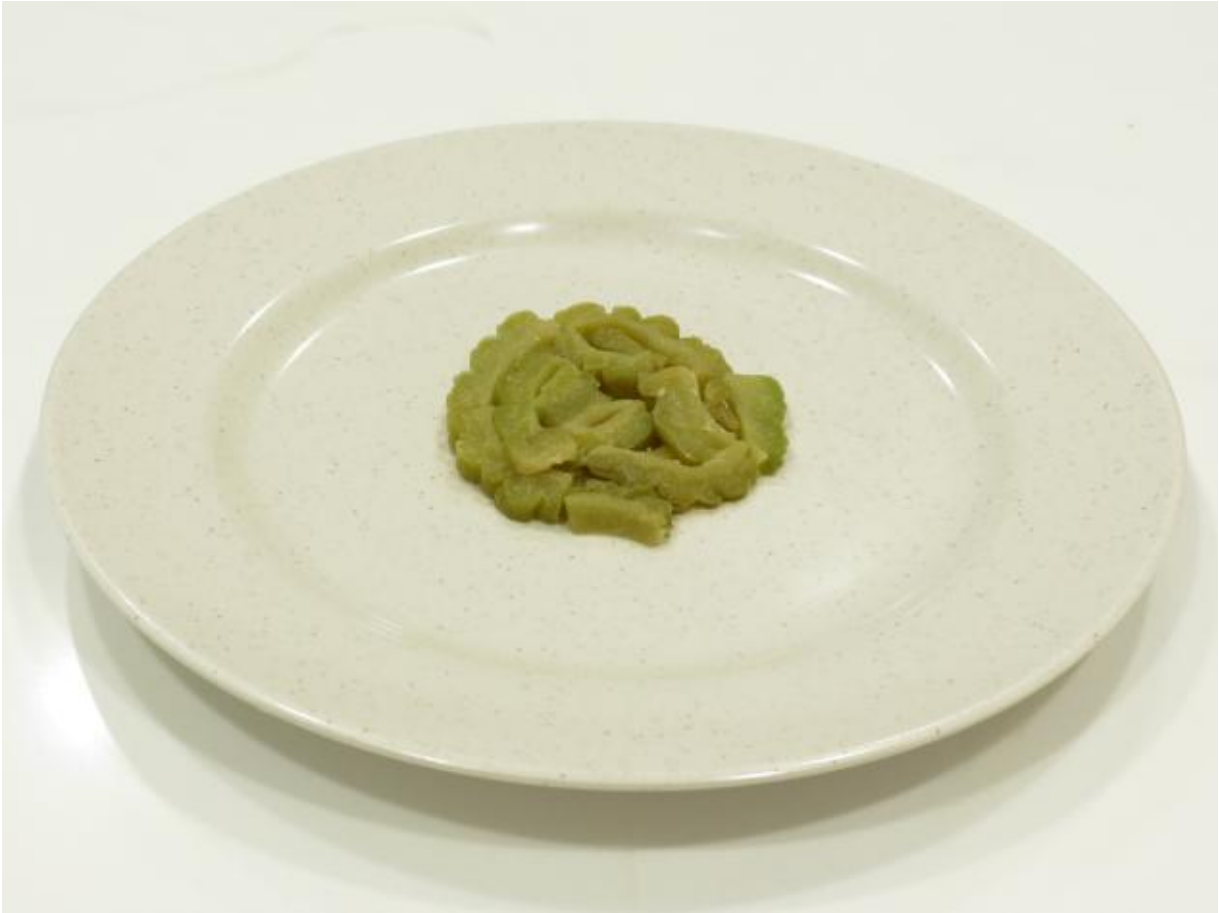

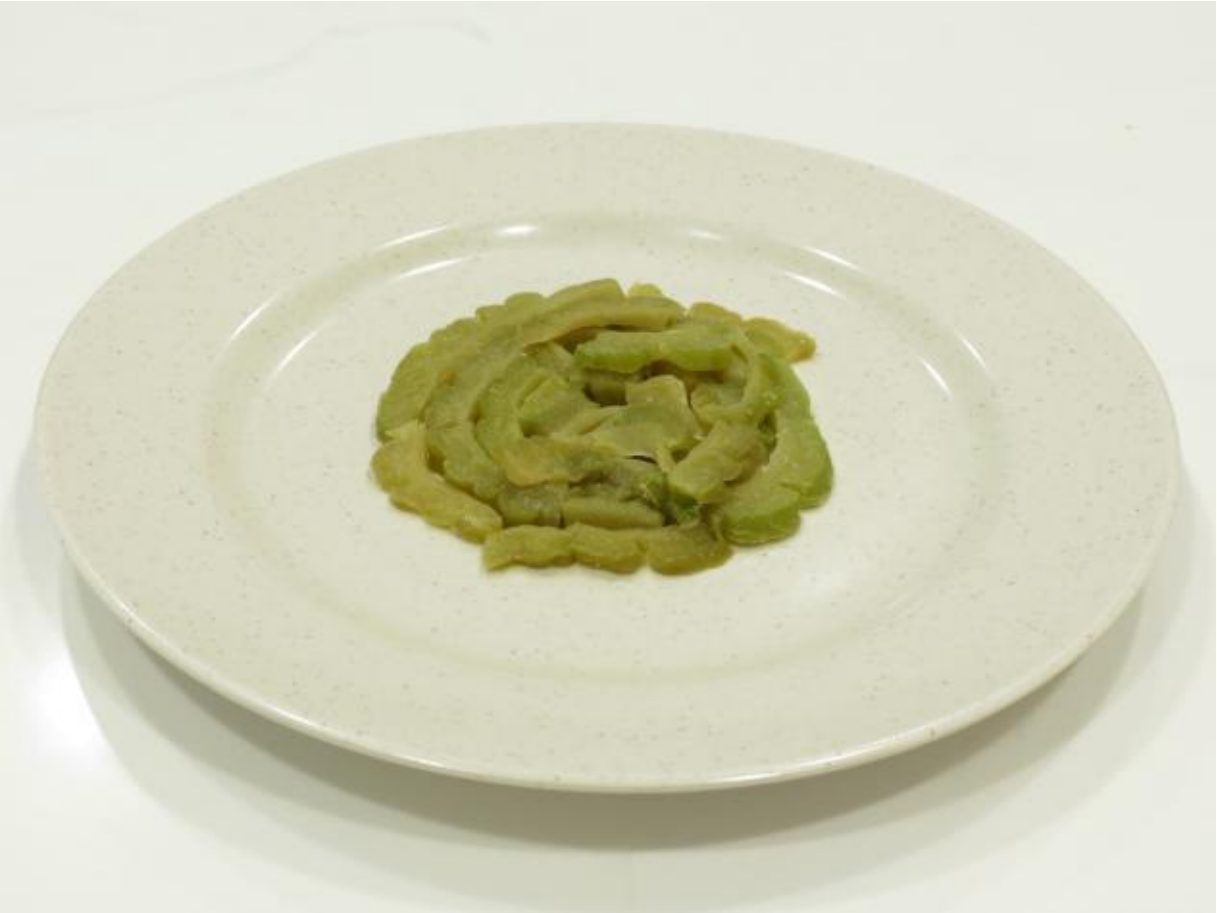

Brinjal / Eggplant, sliced (FA0077)

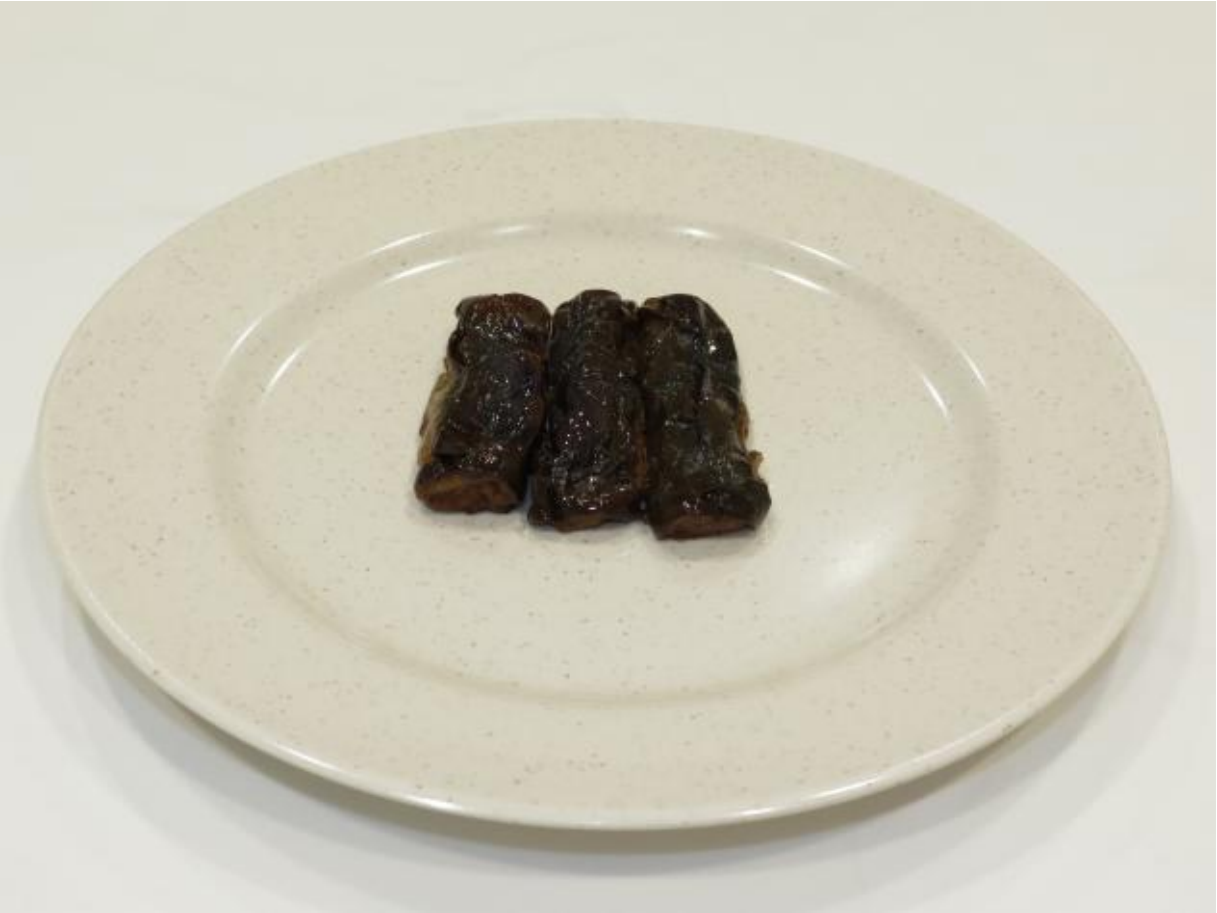
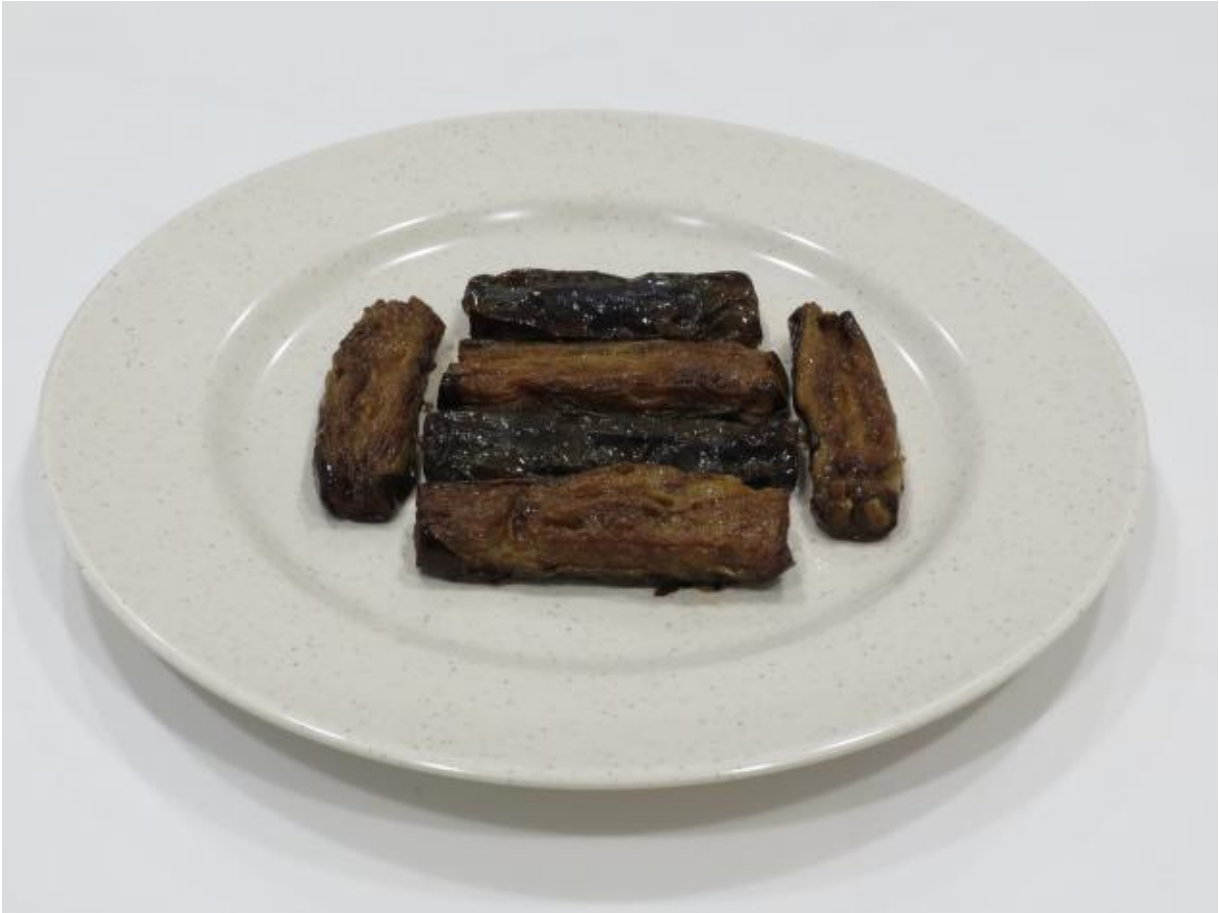
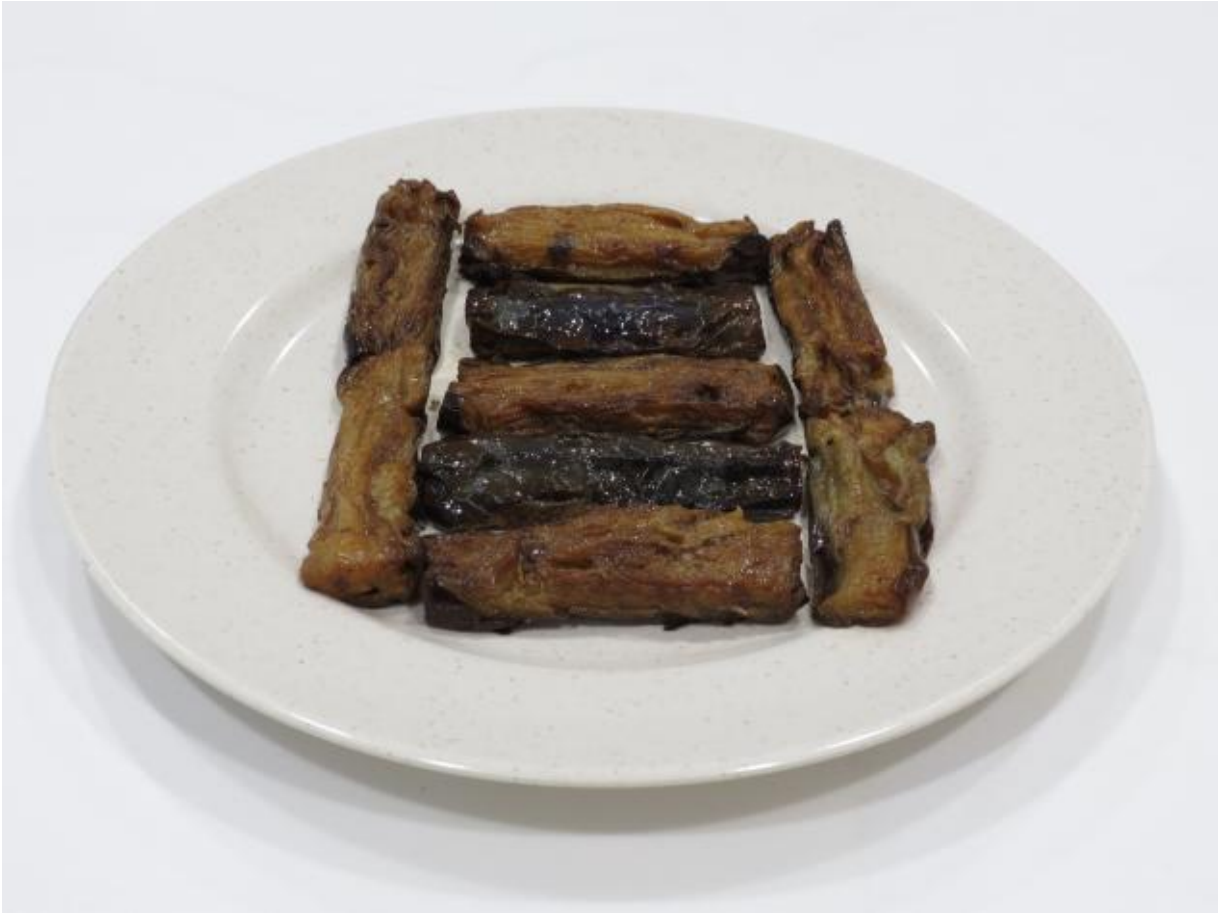
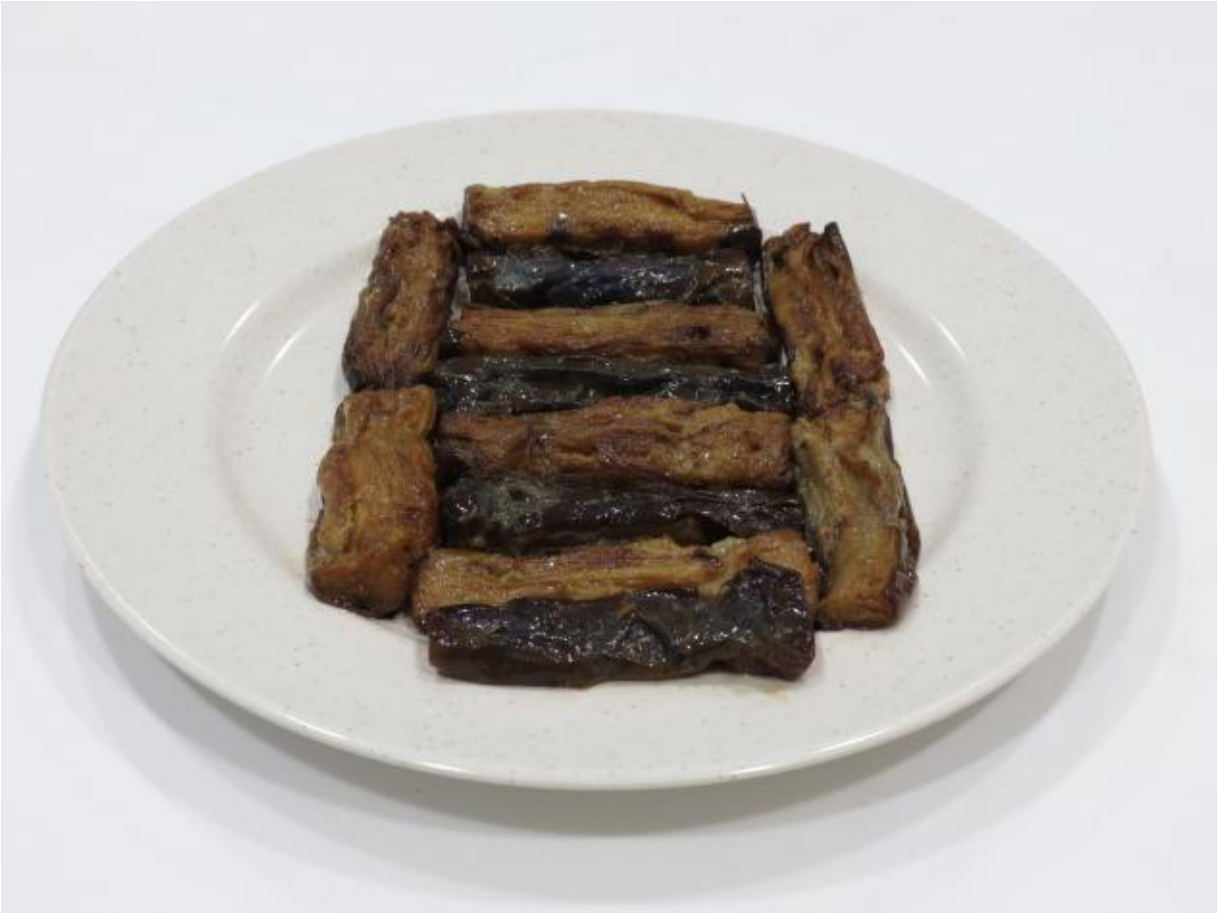

Chilli / Capsicum, sliced (FA0076)

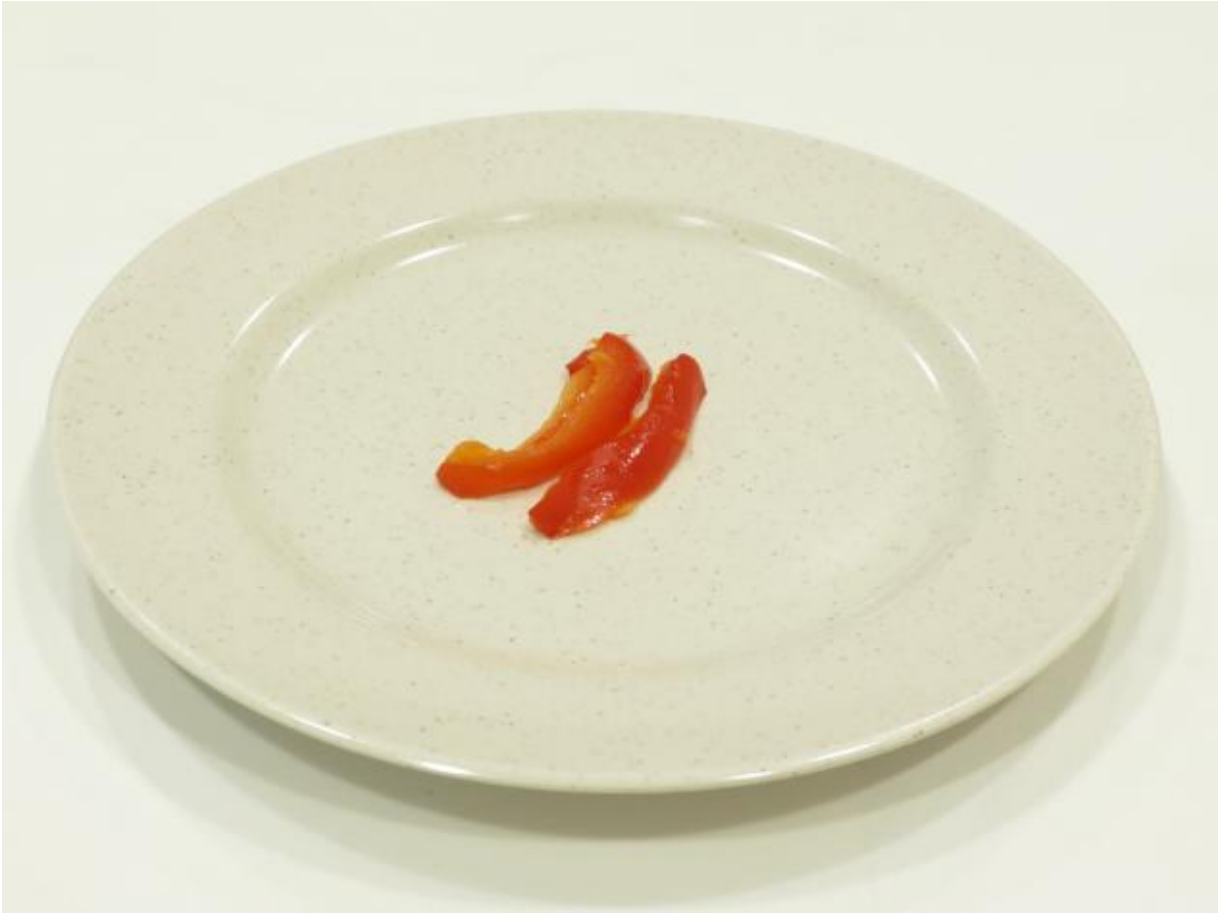
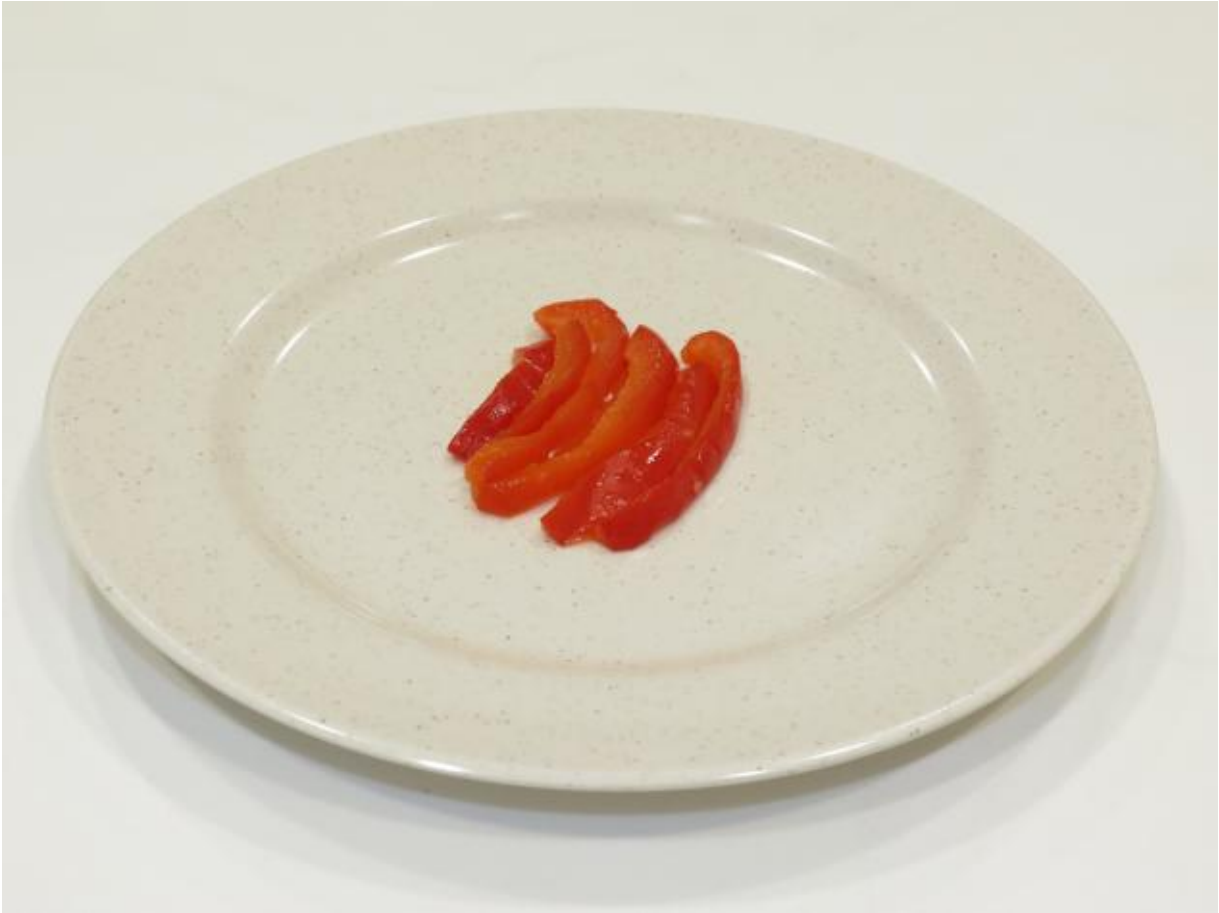
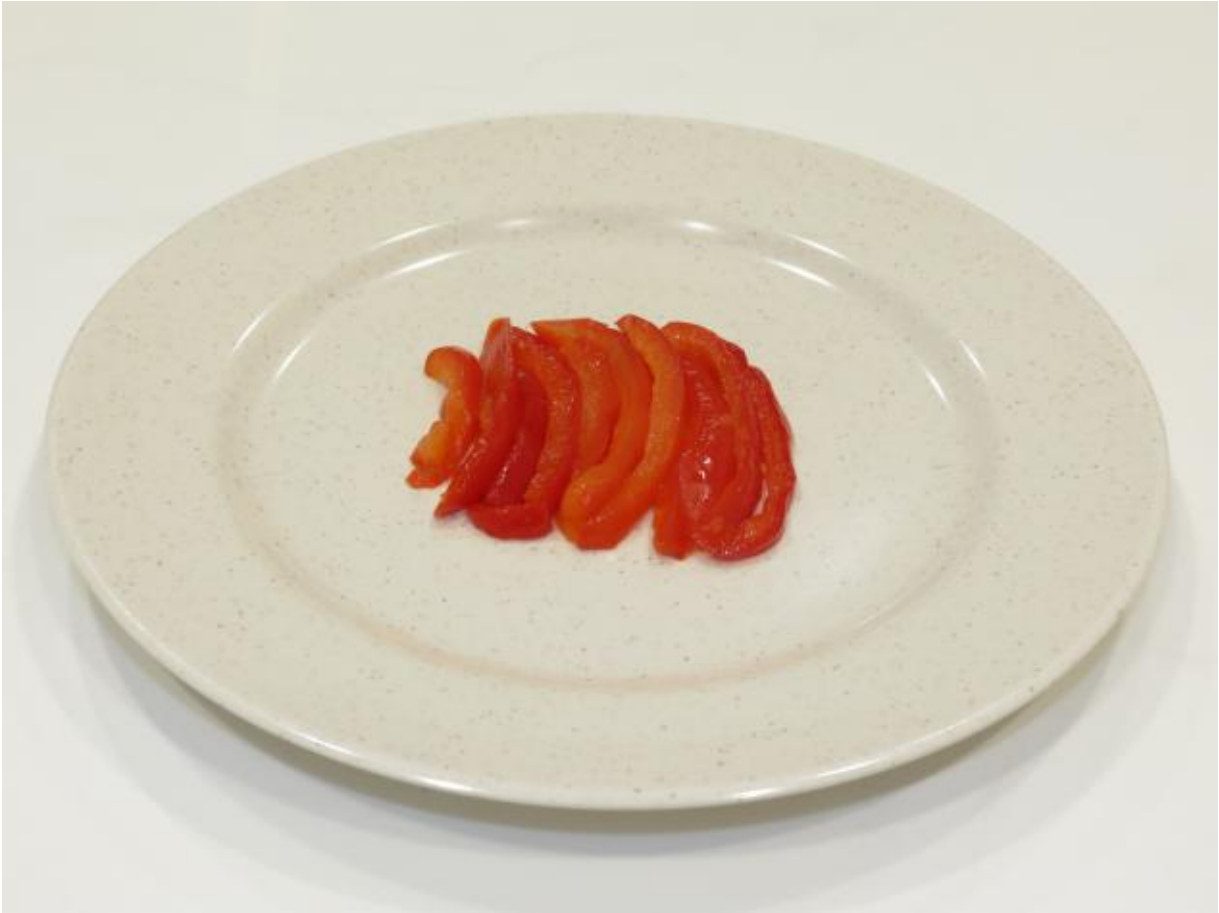
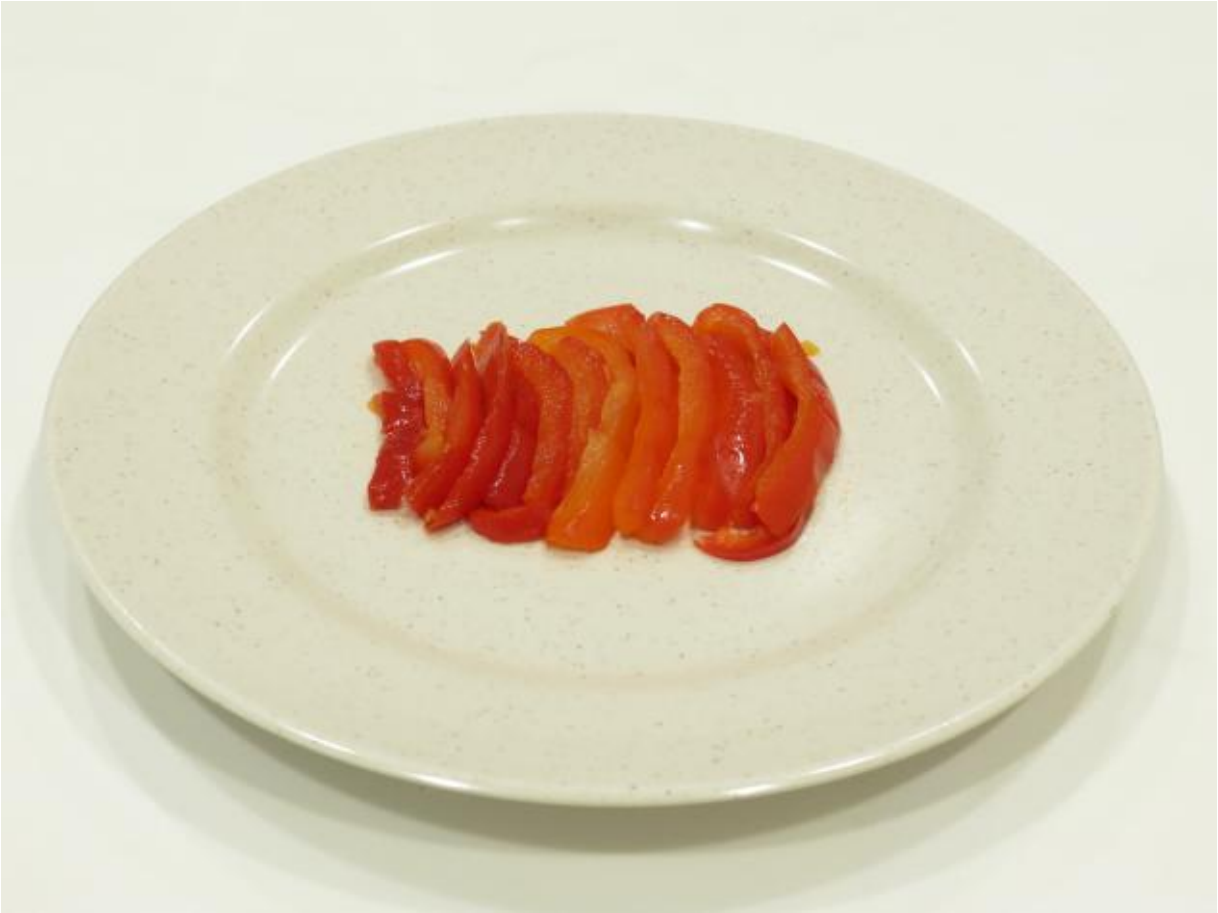

Beansprout (FA0098)

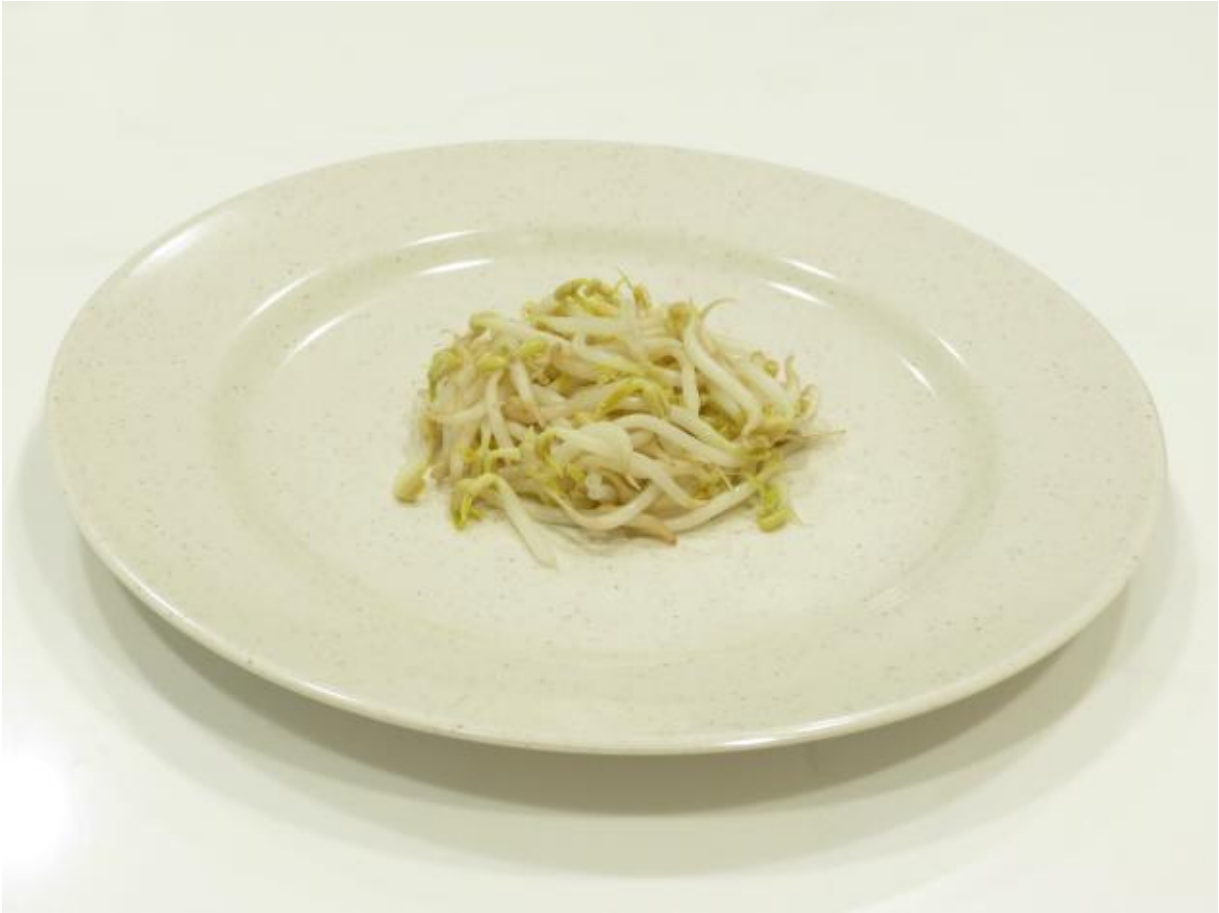
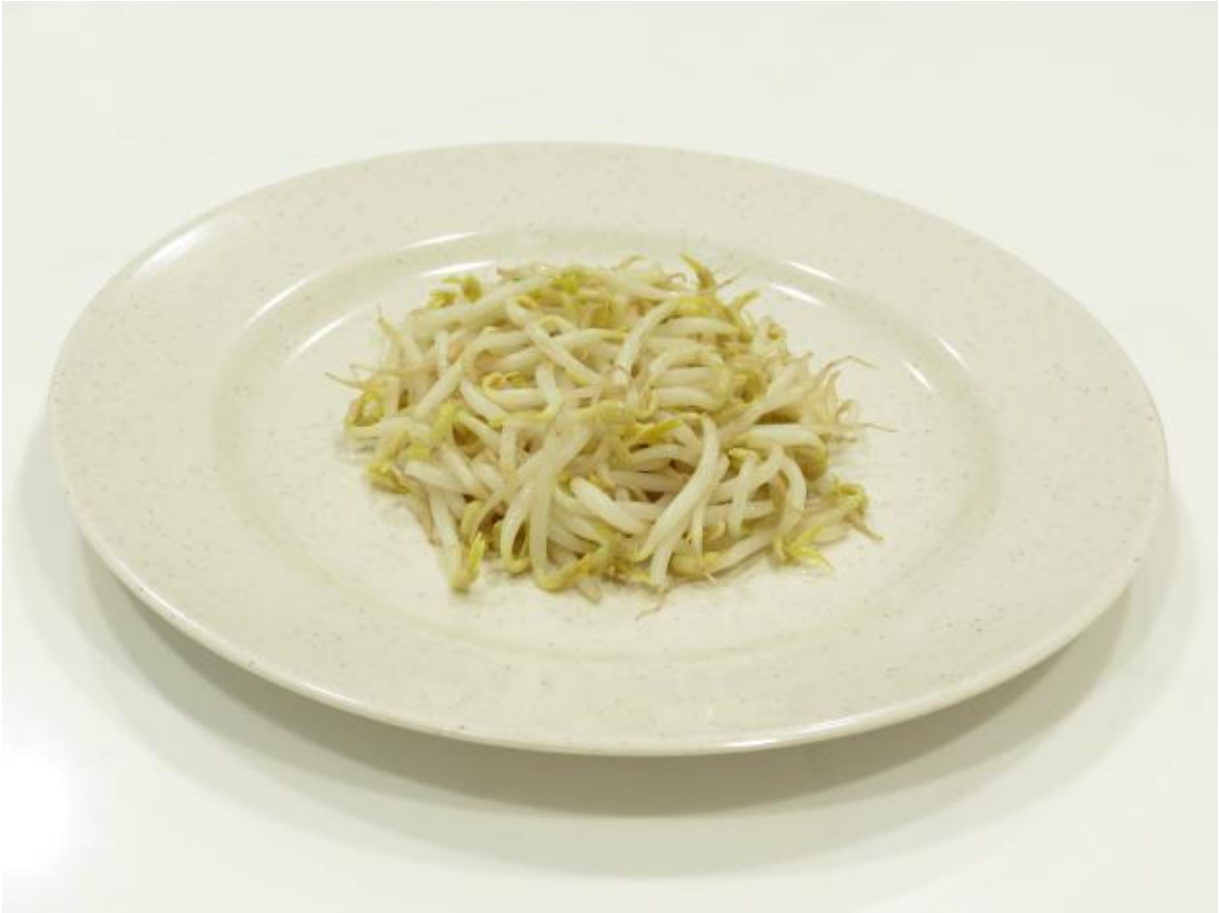
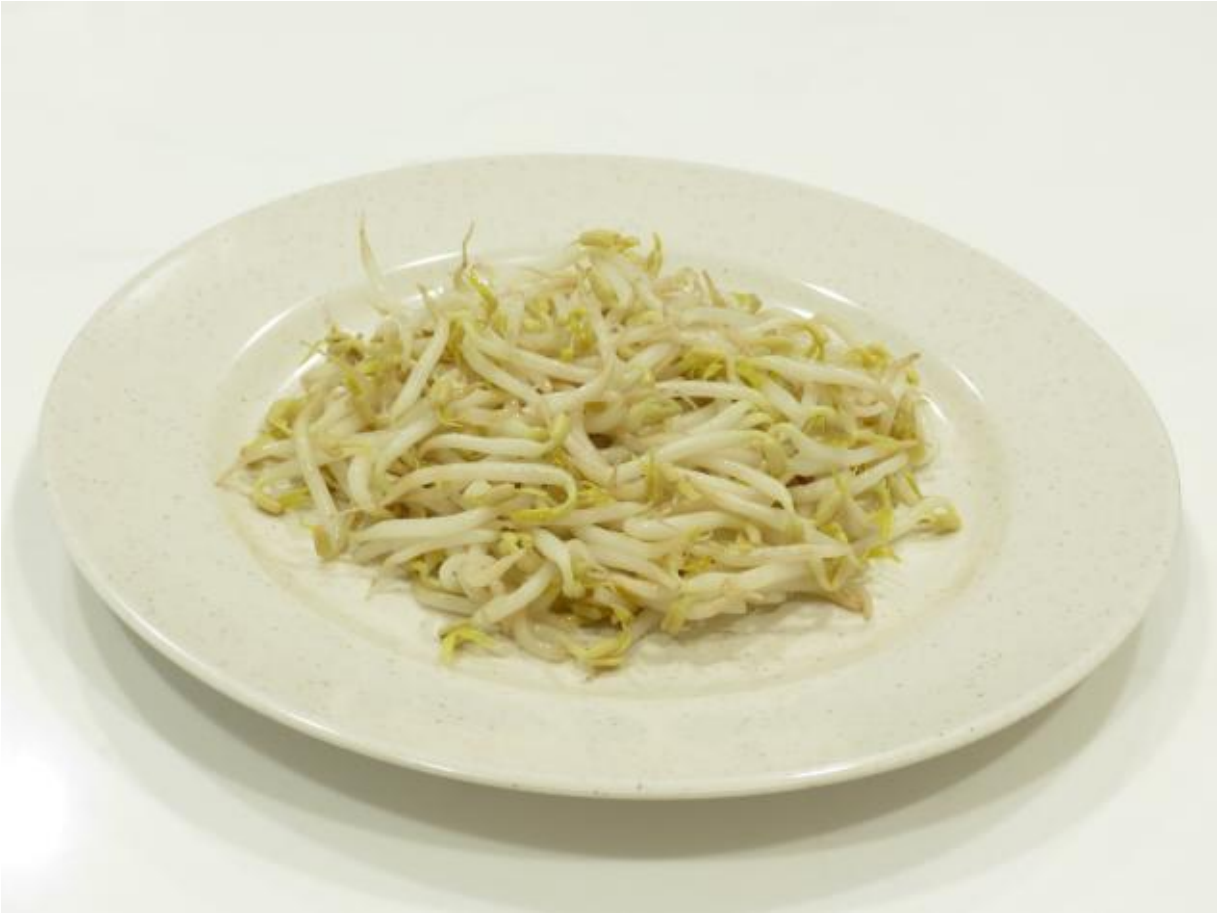
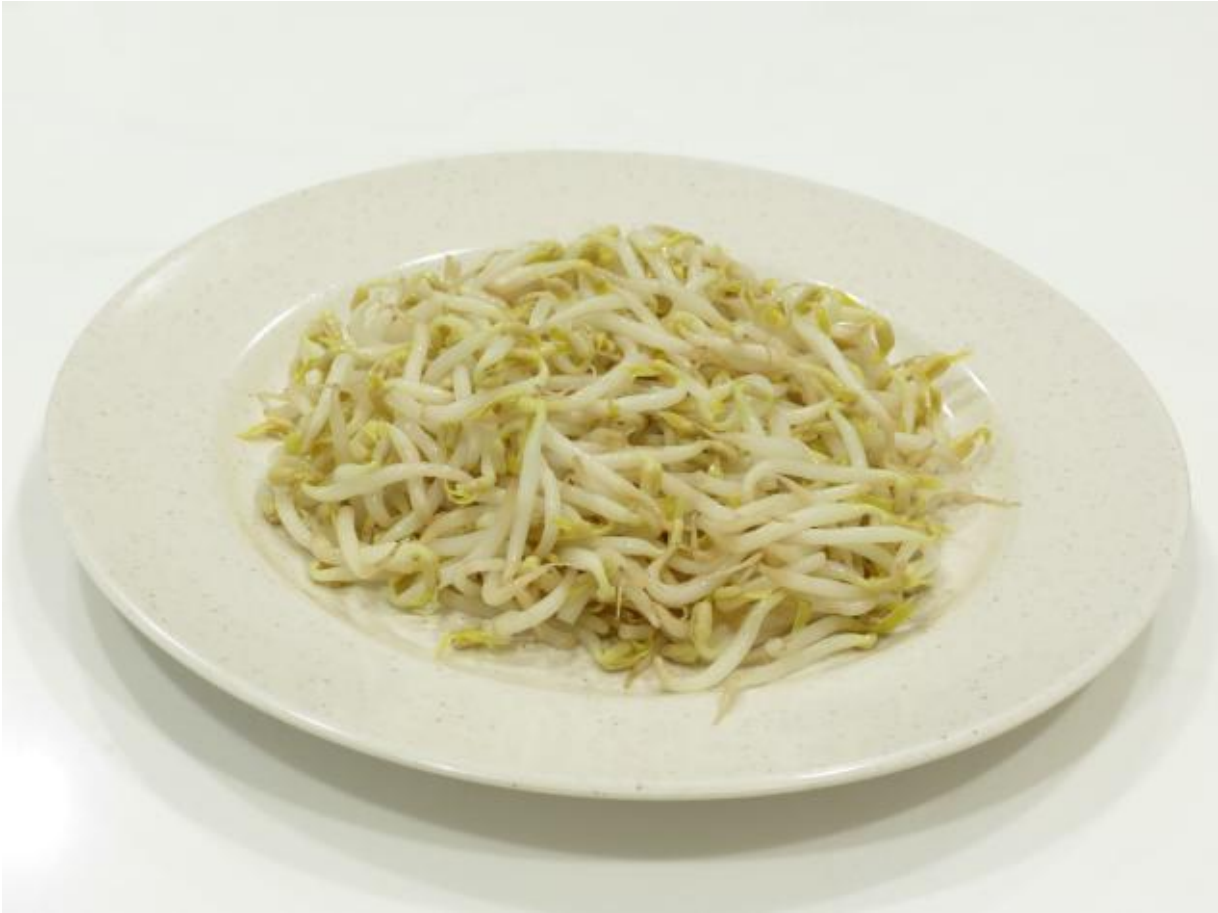

Chye sim (FA0128)

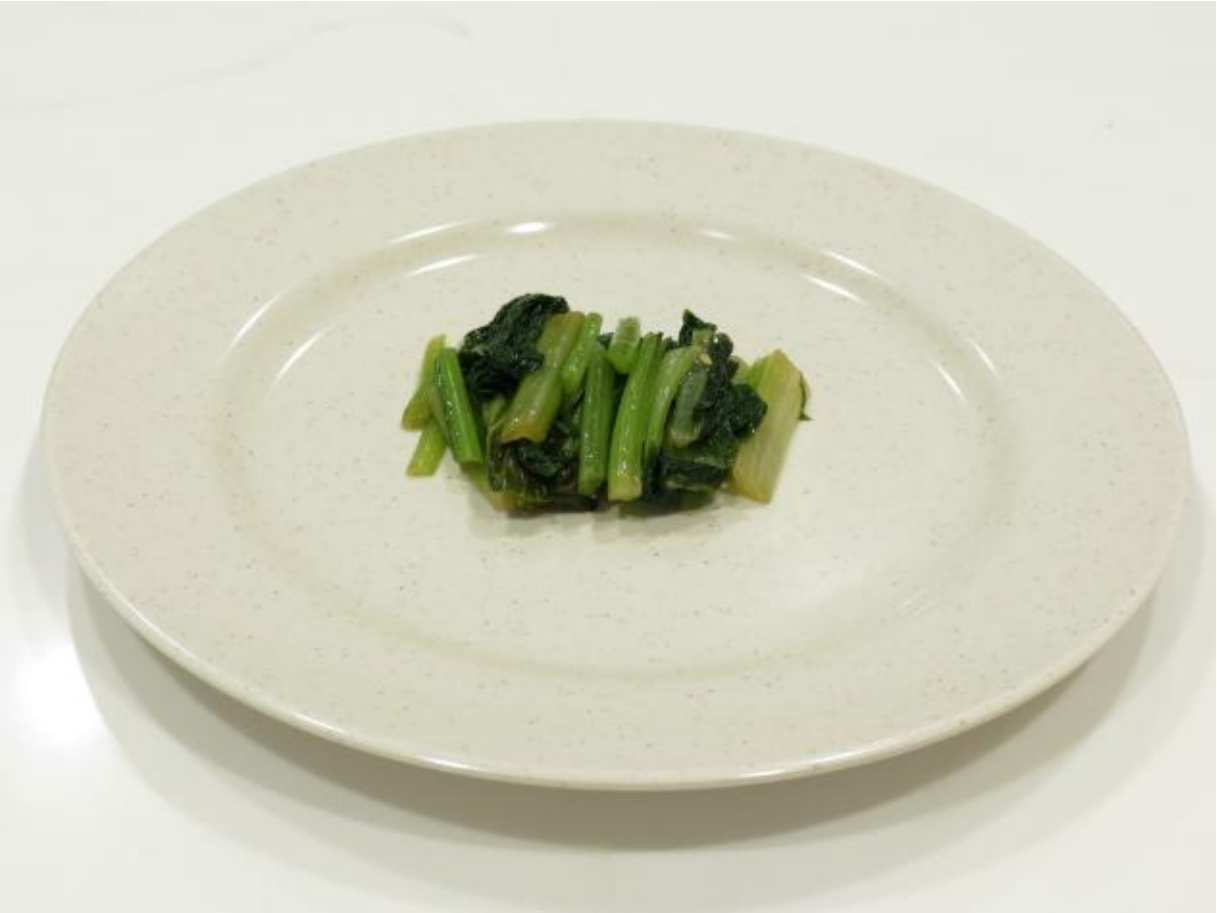

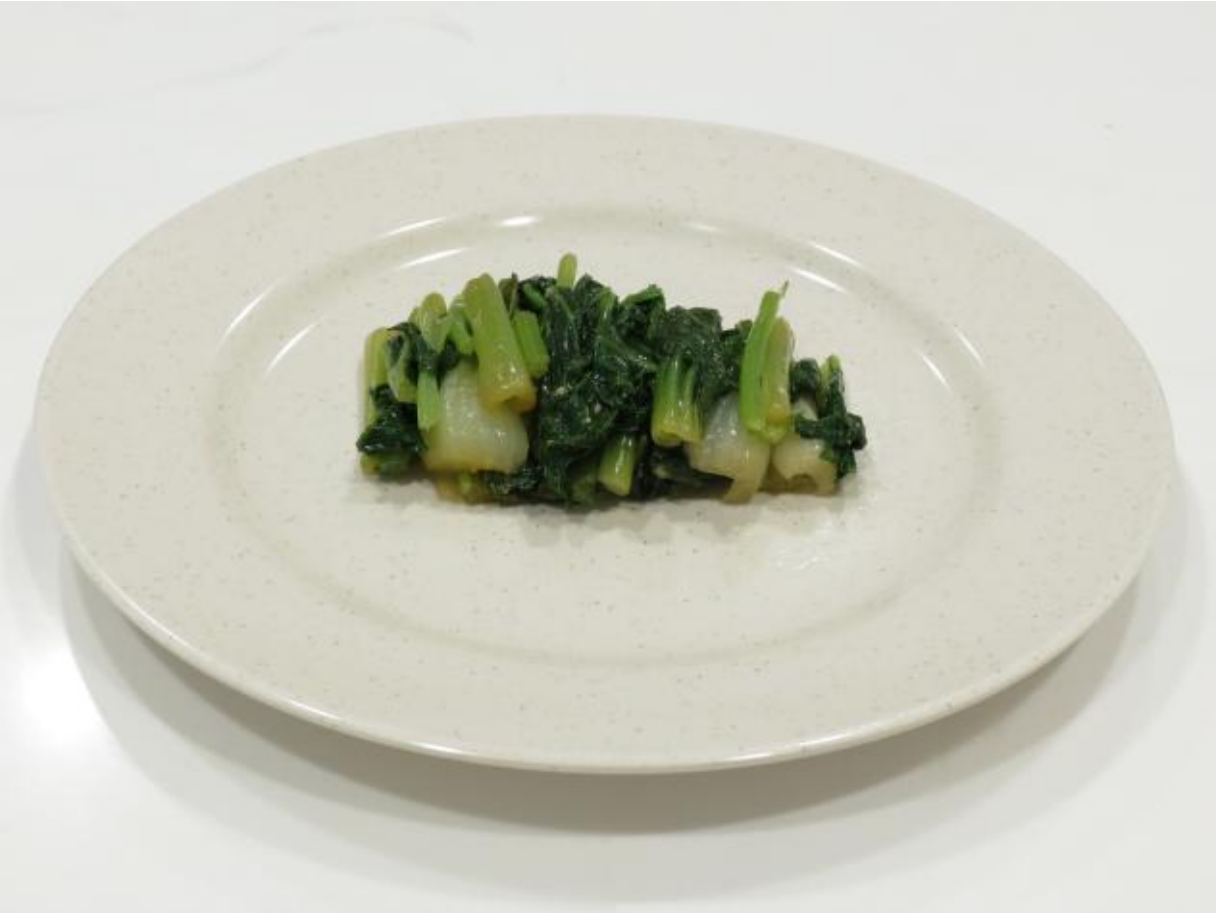

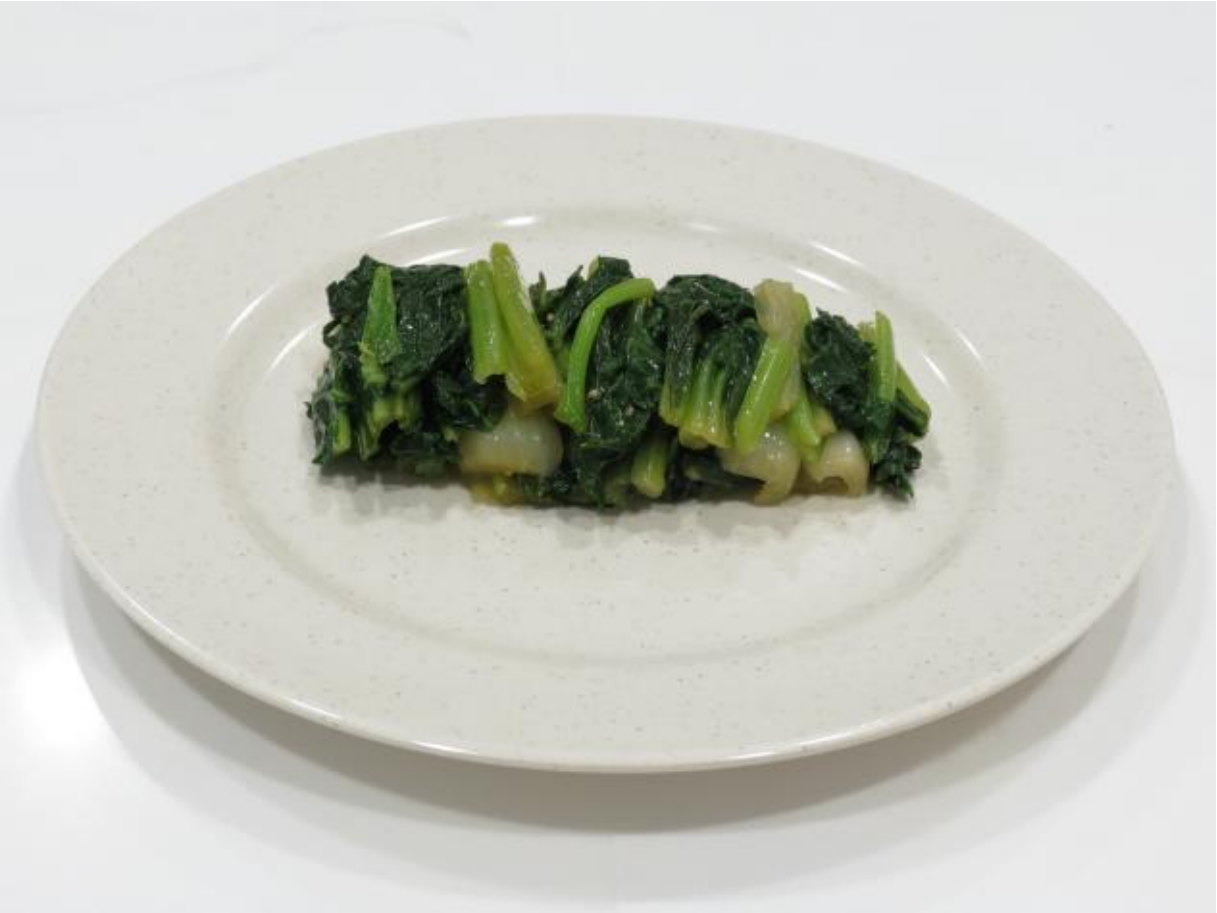

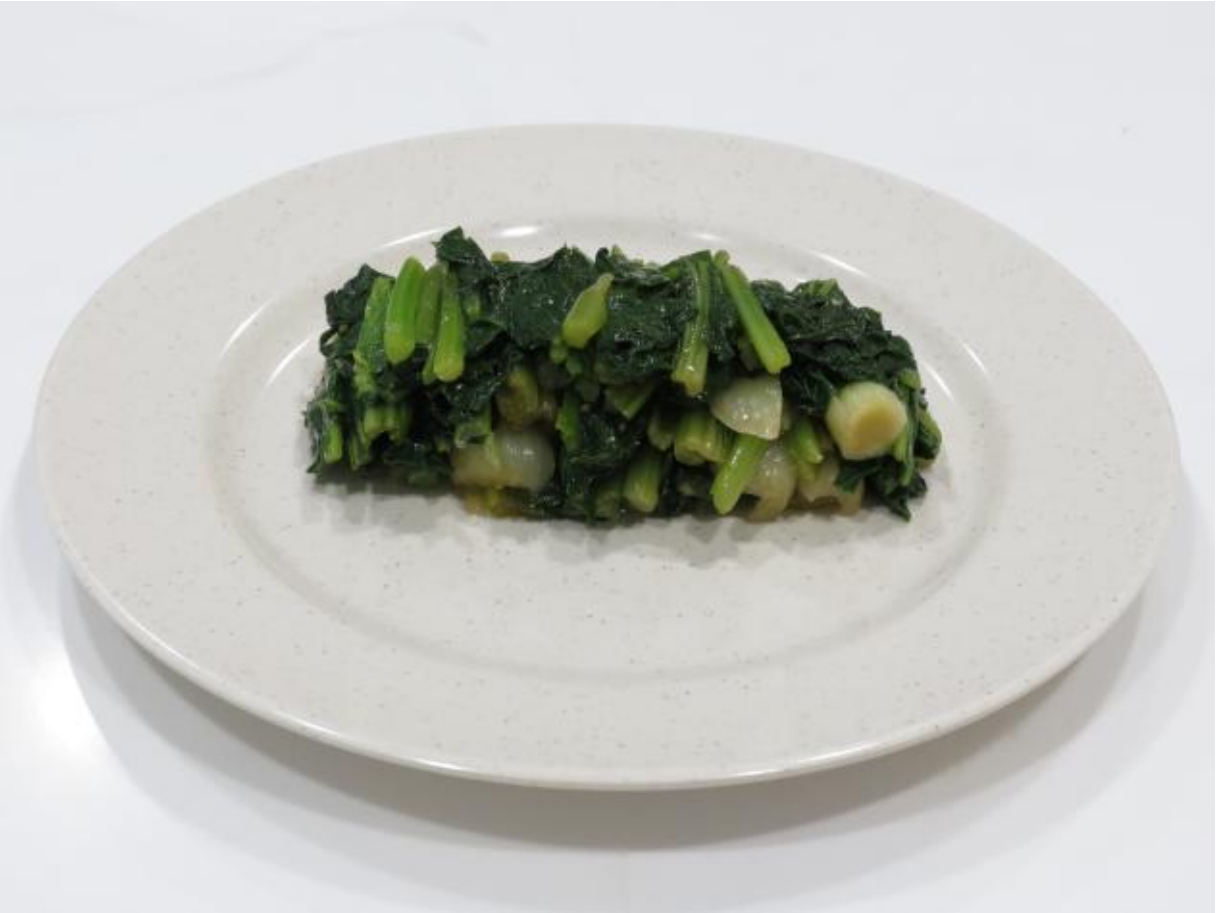

Kailan (FA0139)

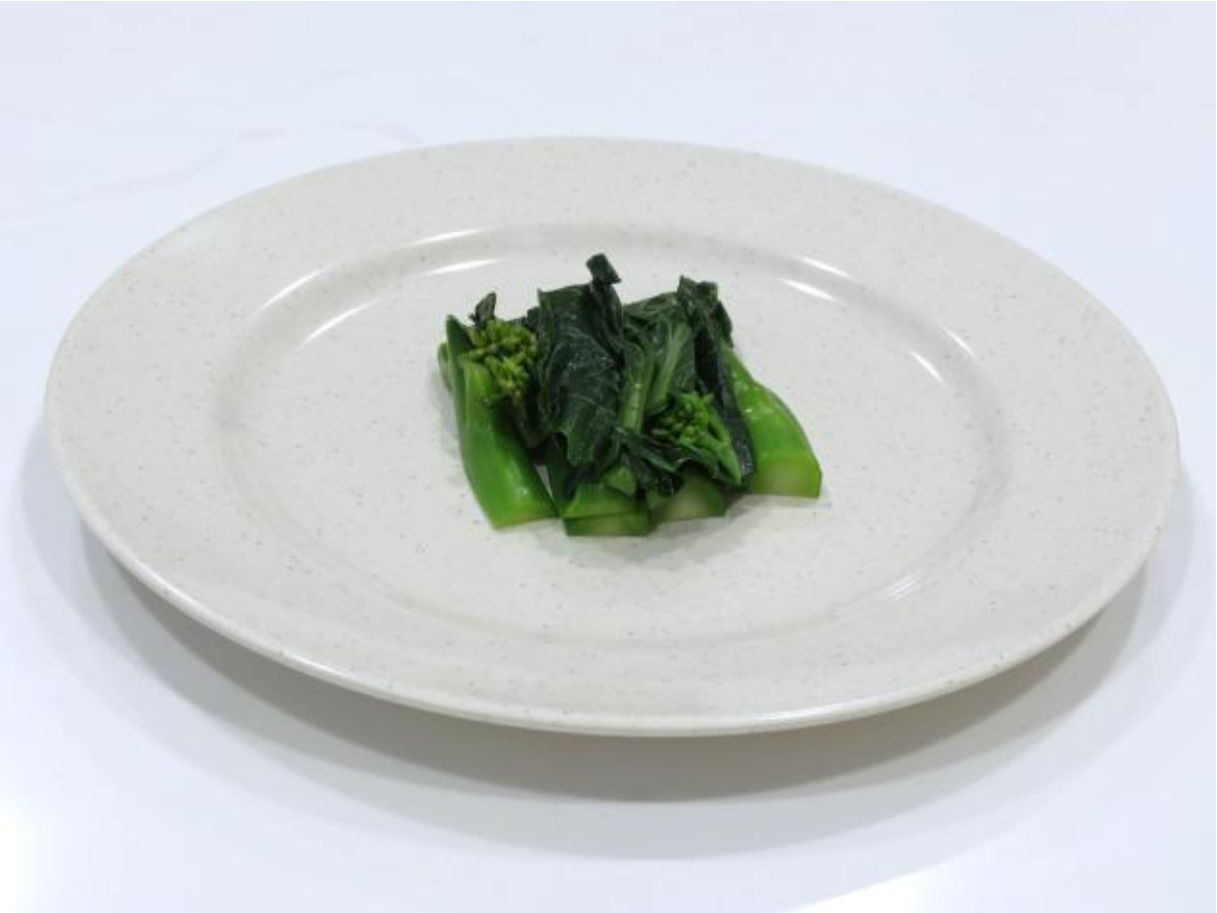
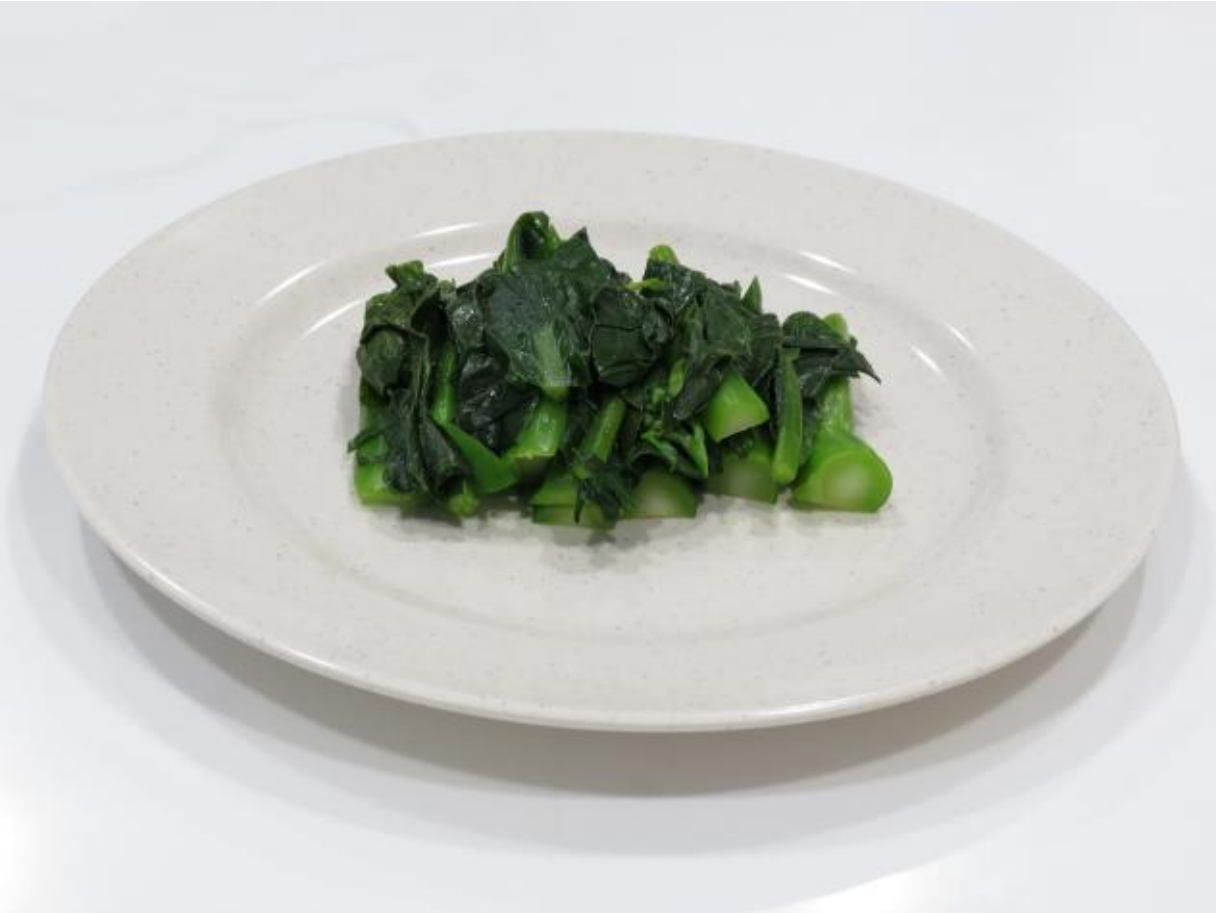
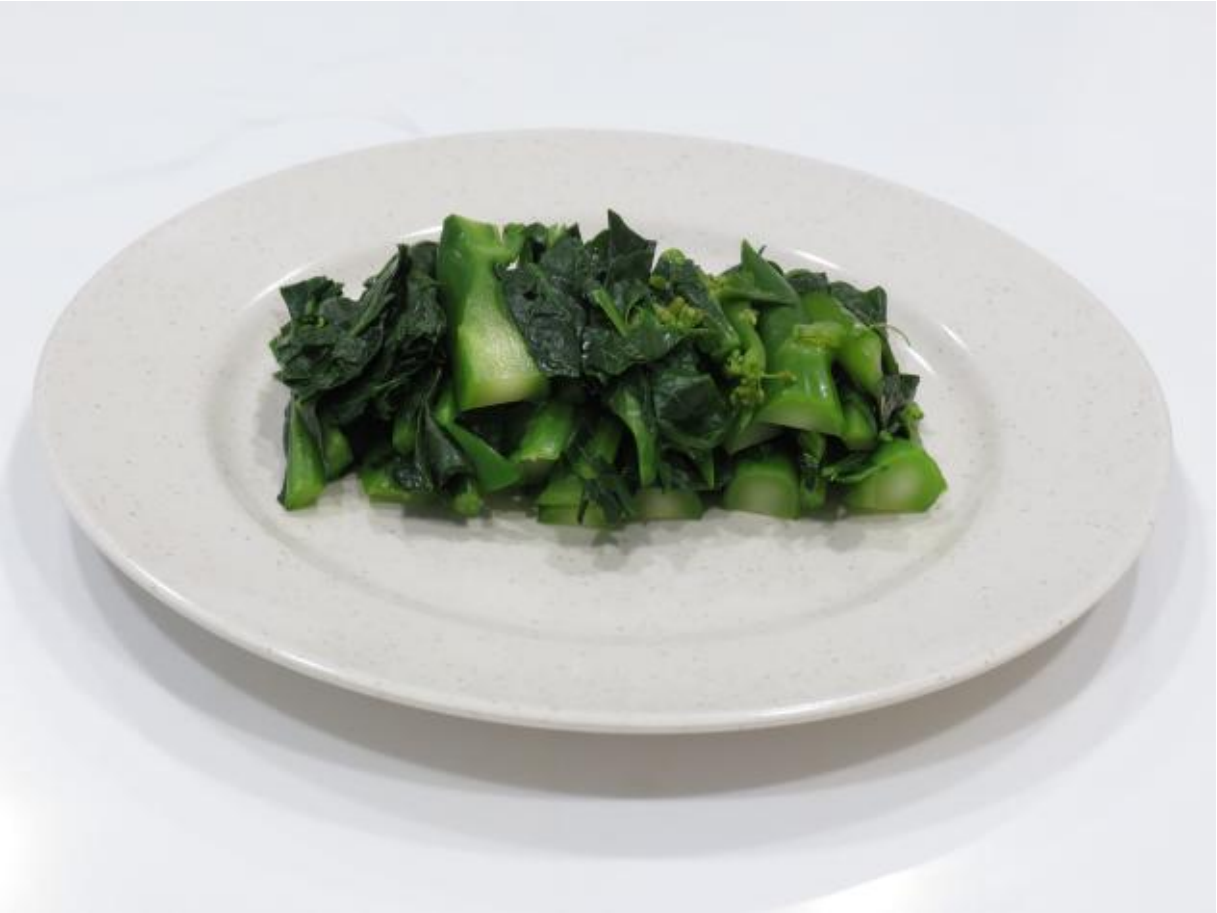
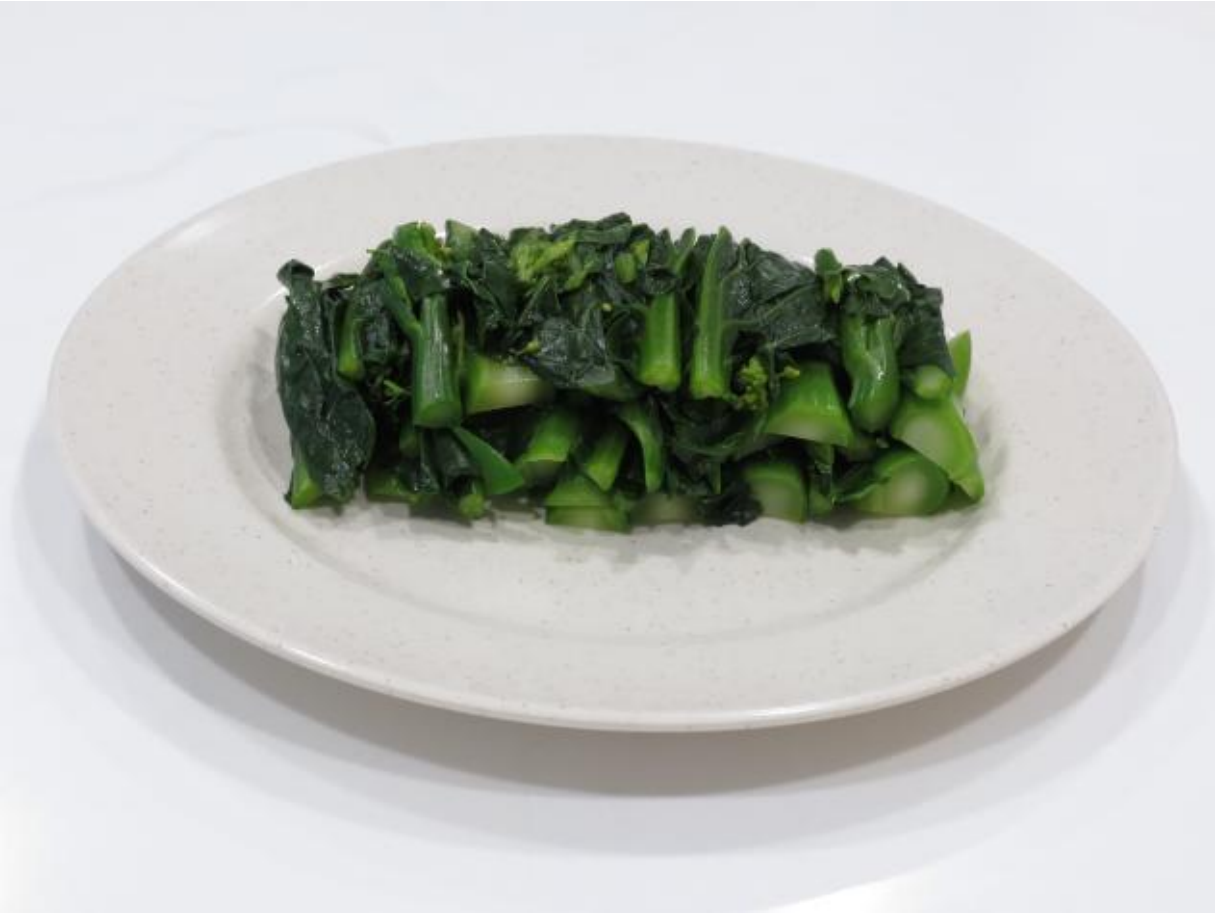

Preserved vegetable, salted (FA0078)

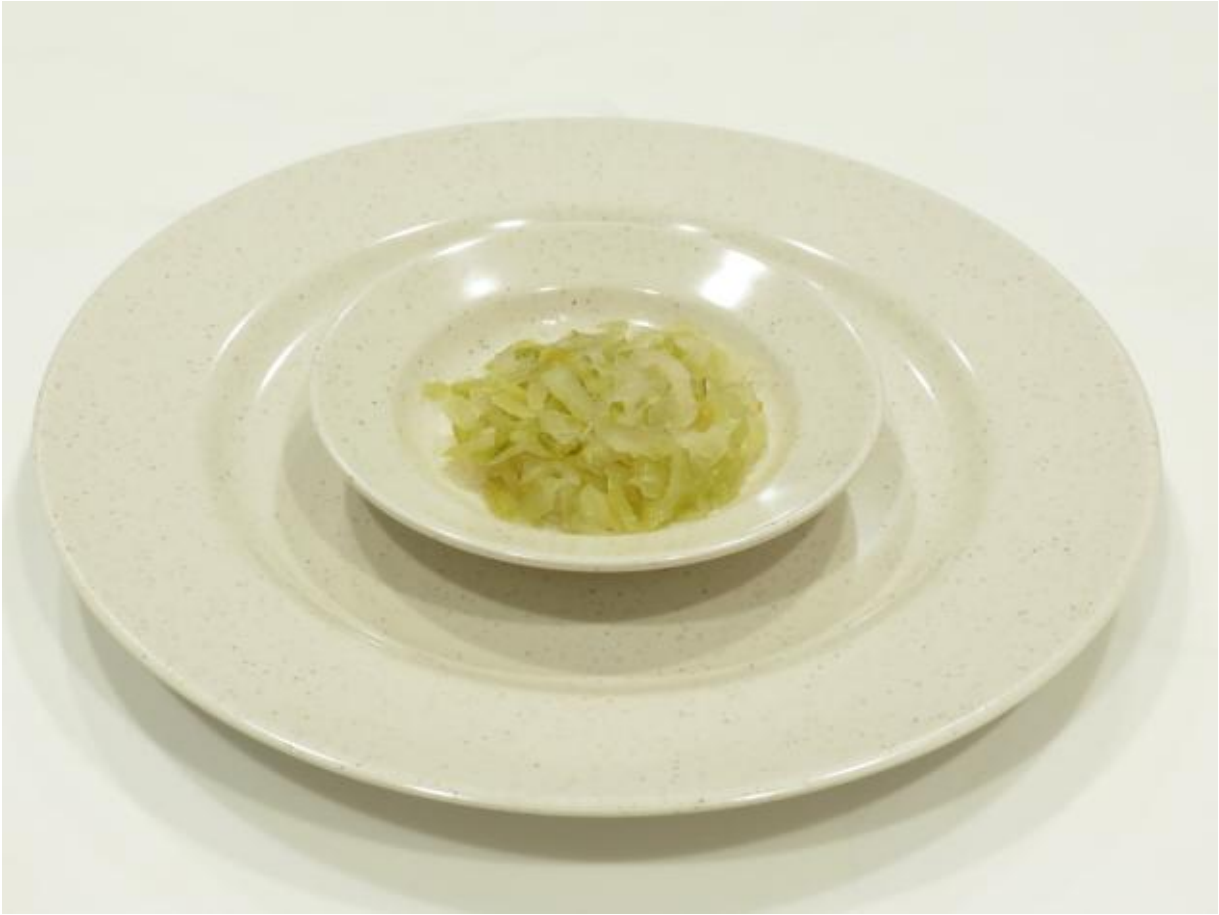
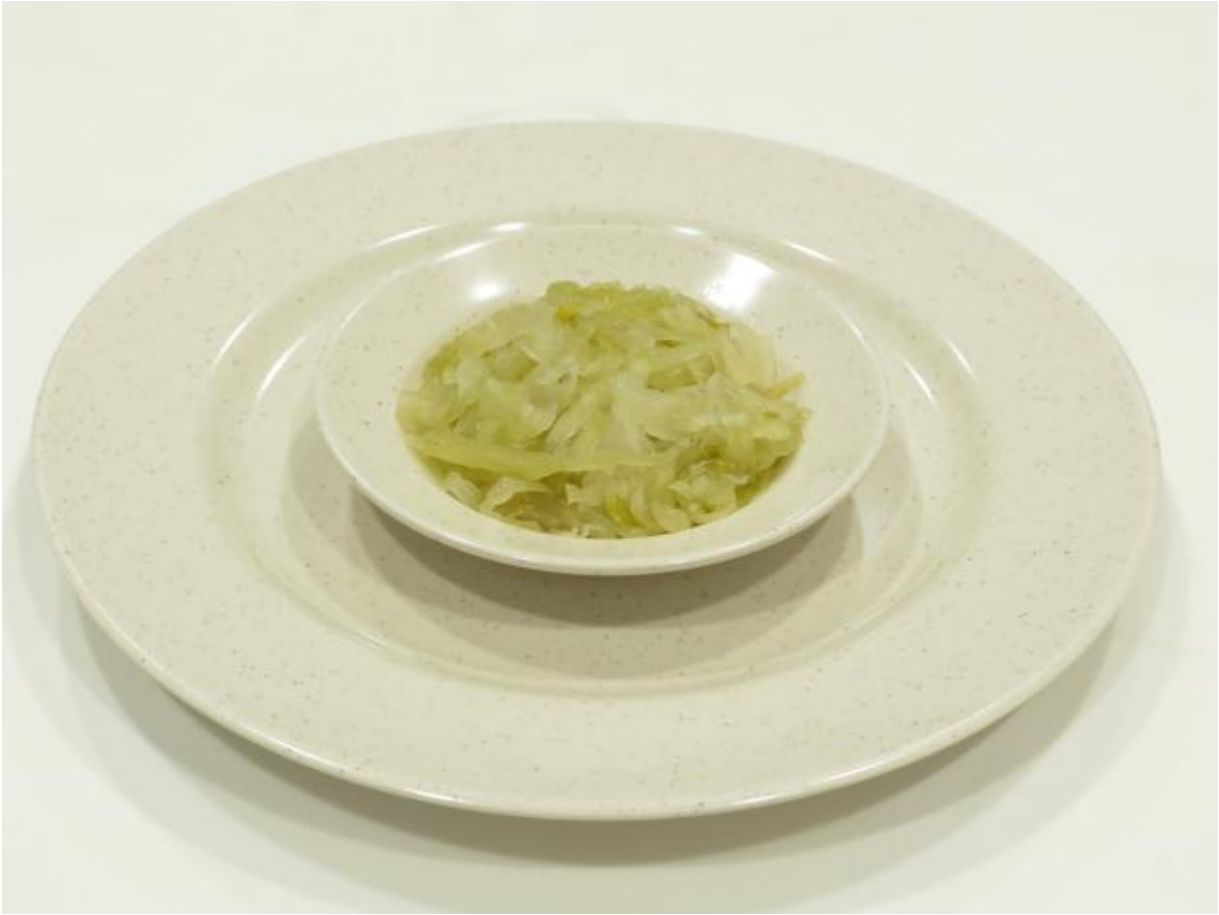
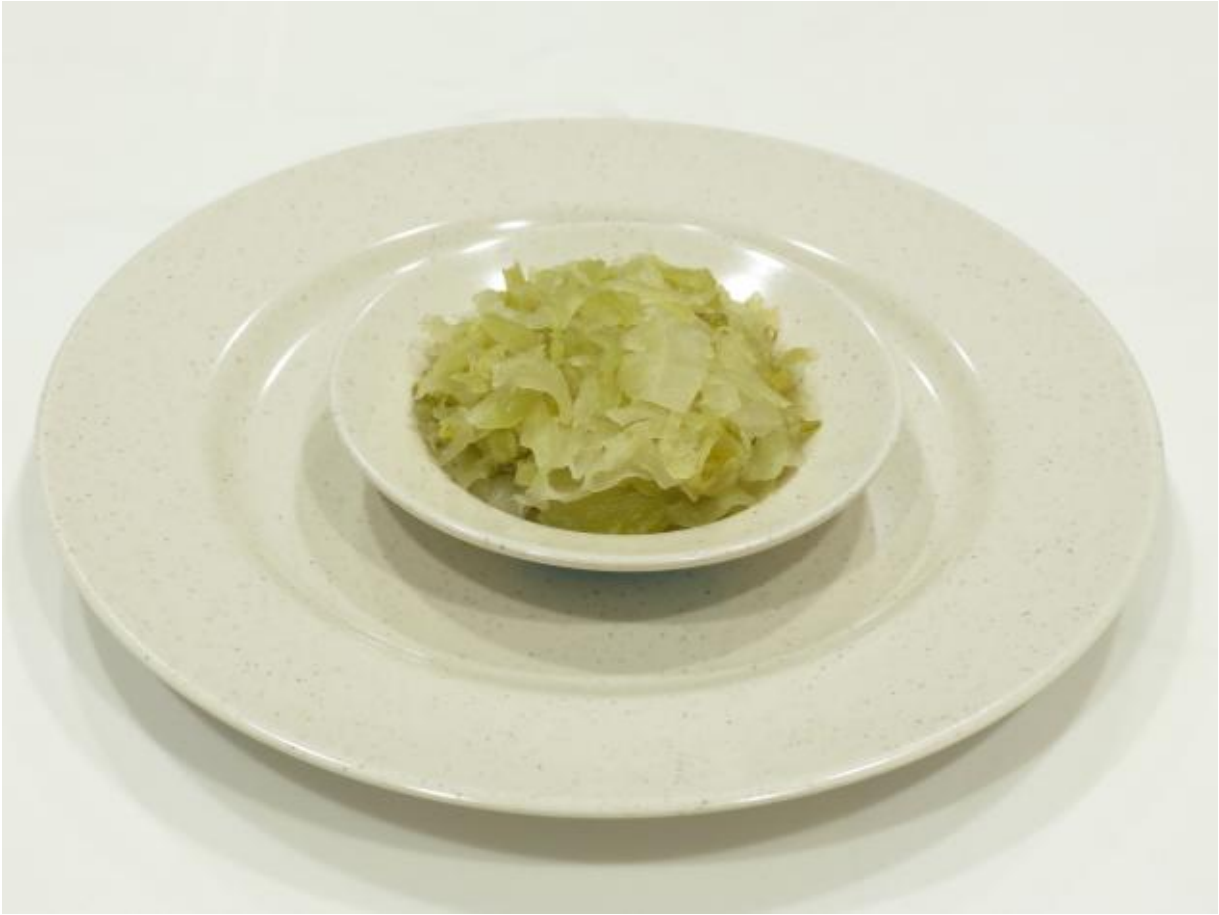
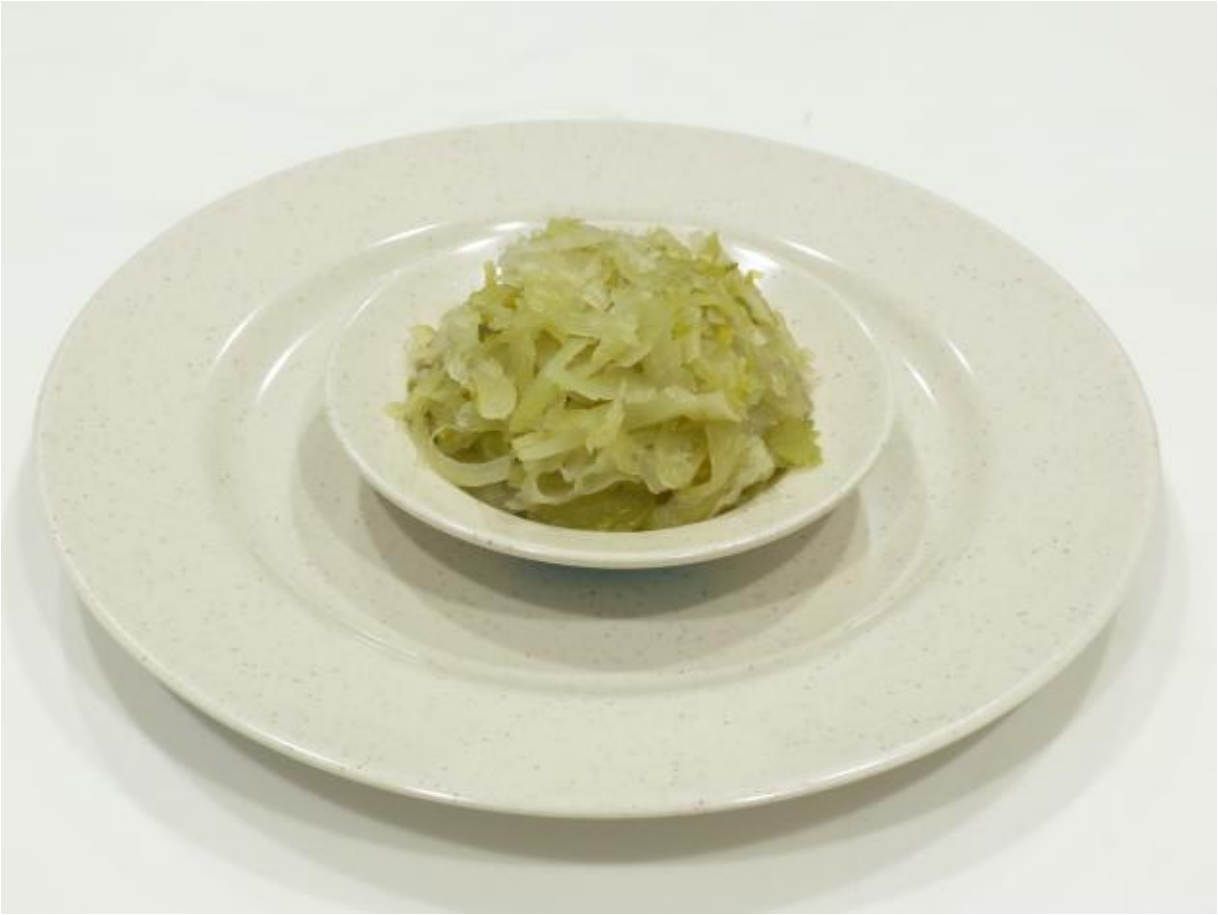

Spinach (FA0057)

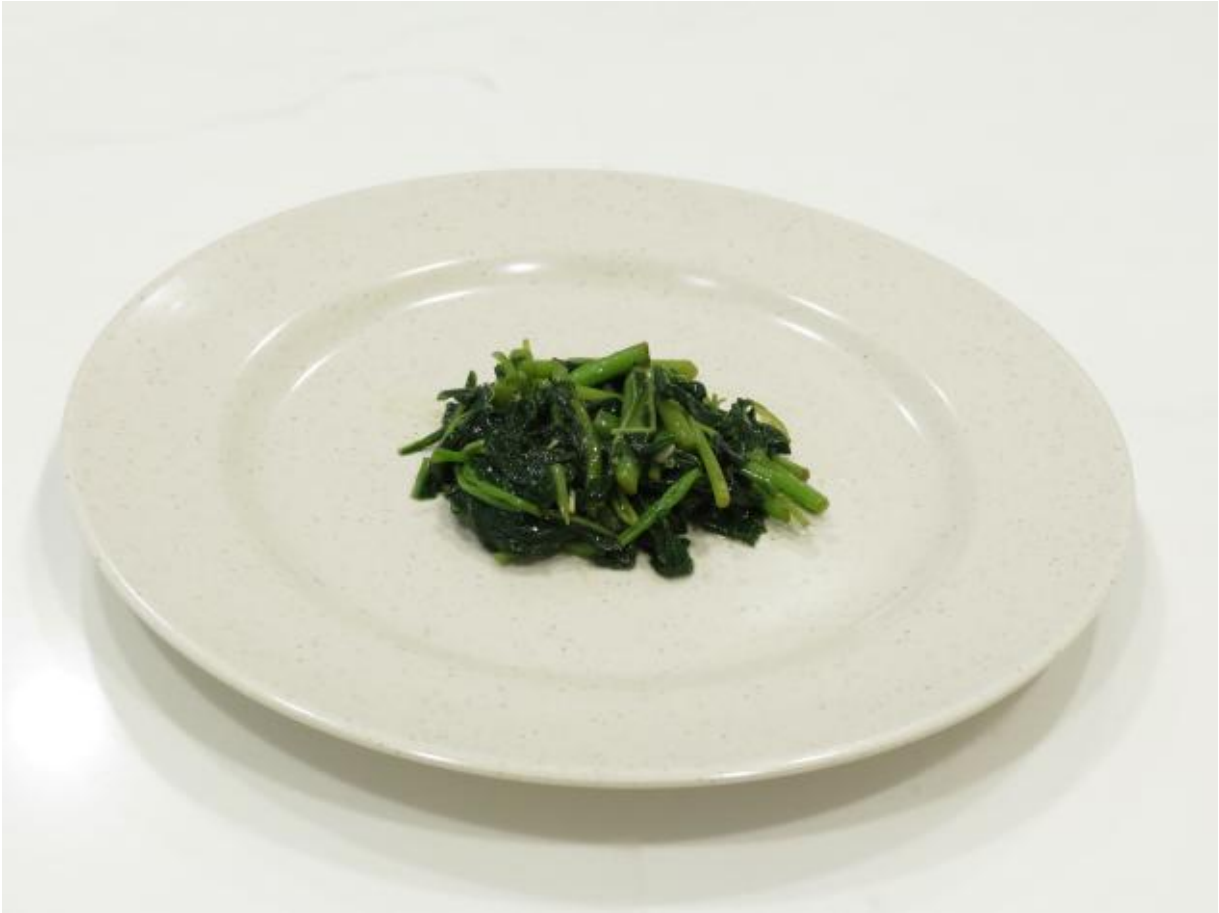

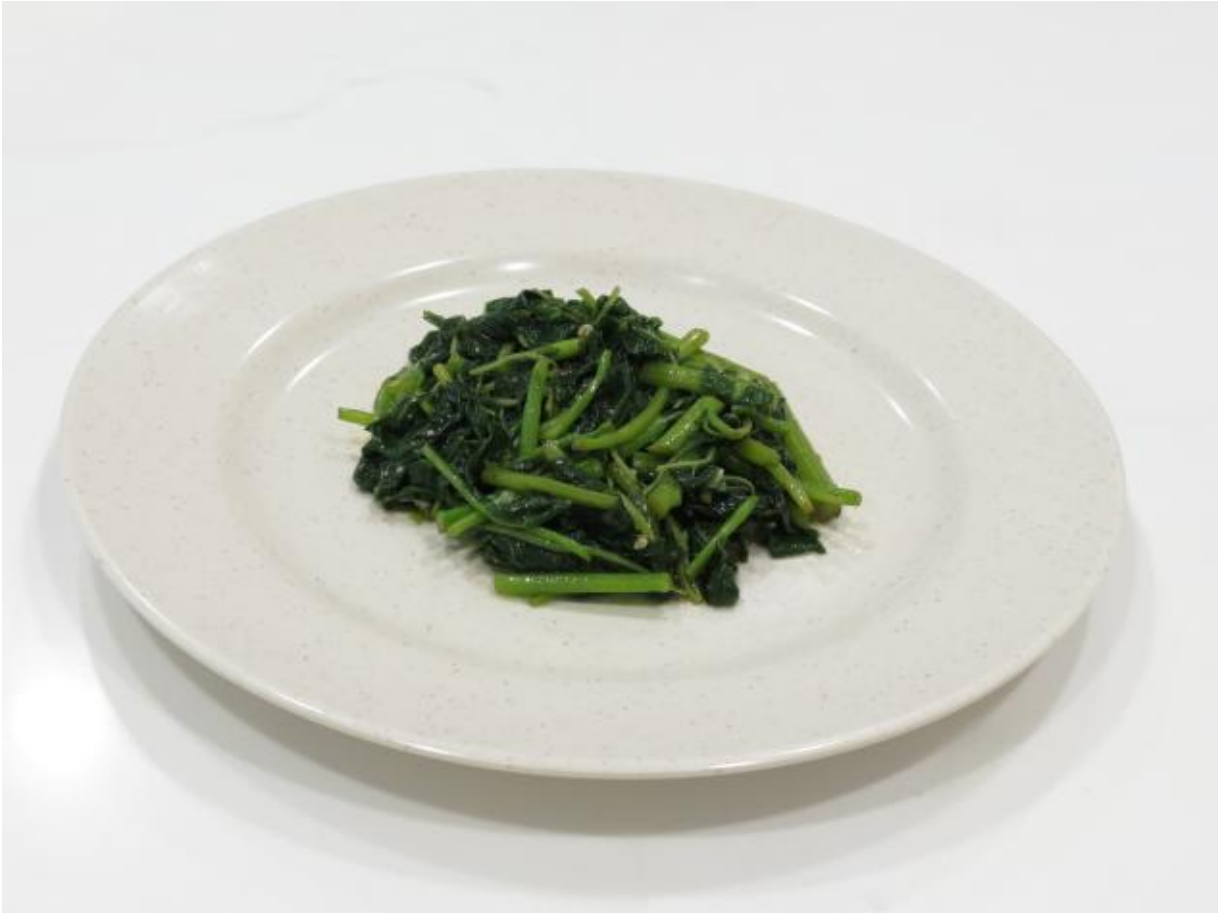

Beancurd, dessert, traditional (FA0026)

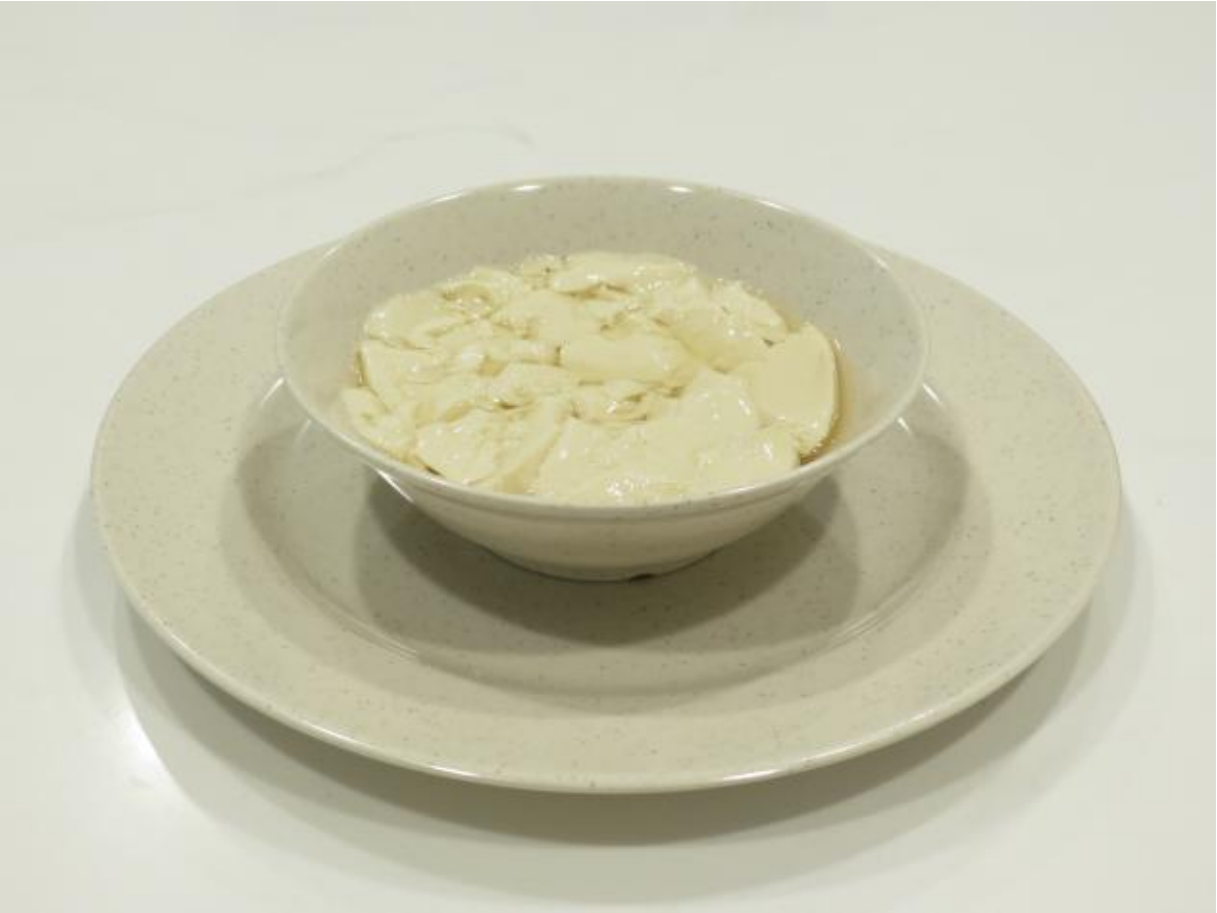

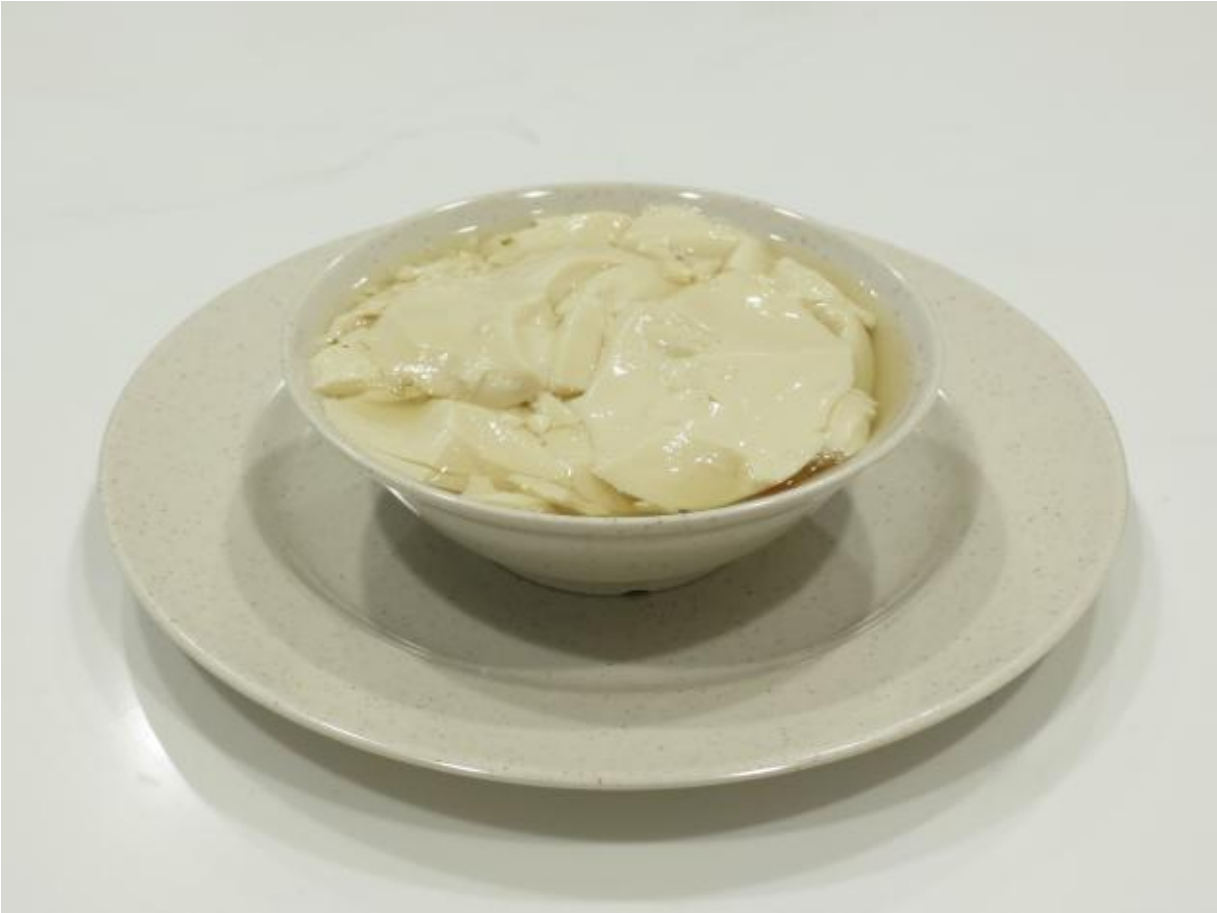

Egg tofu (FA0131)

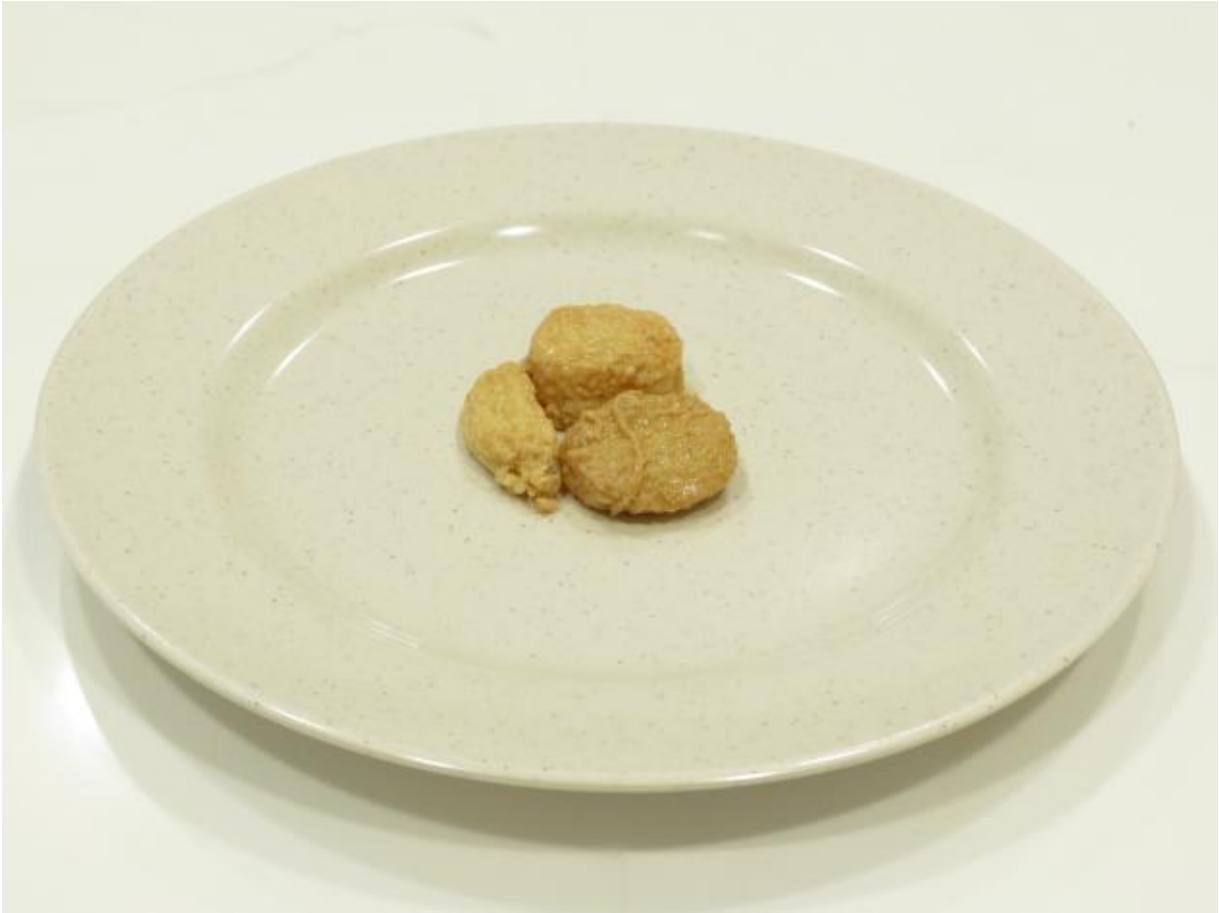

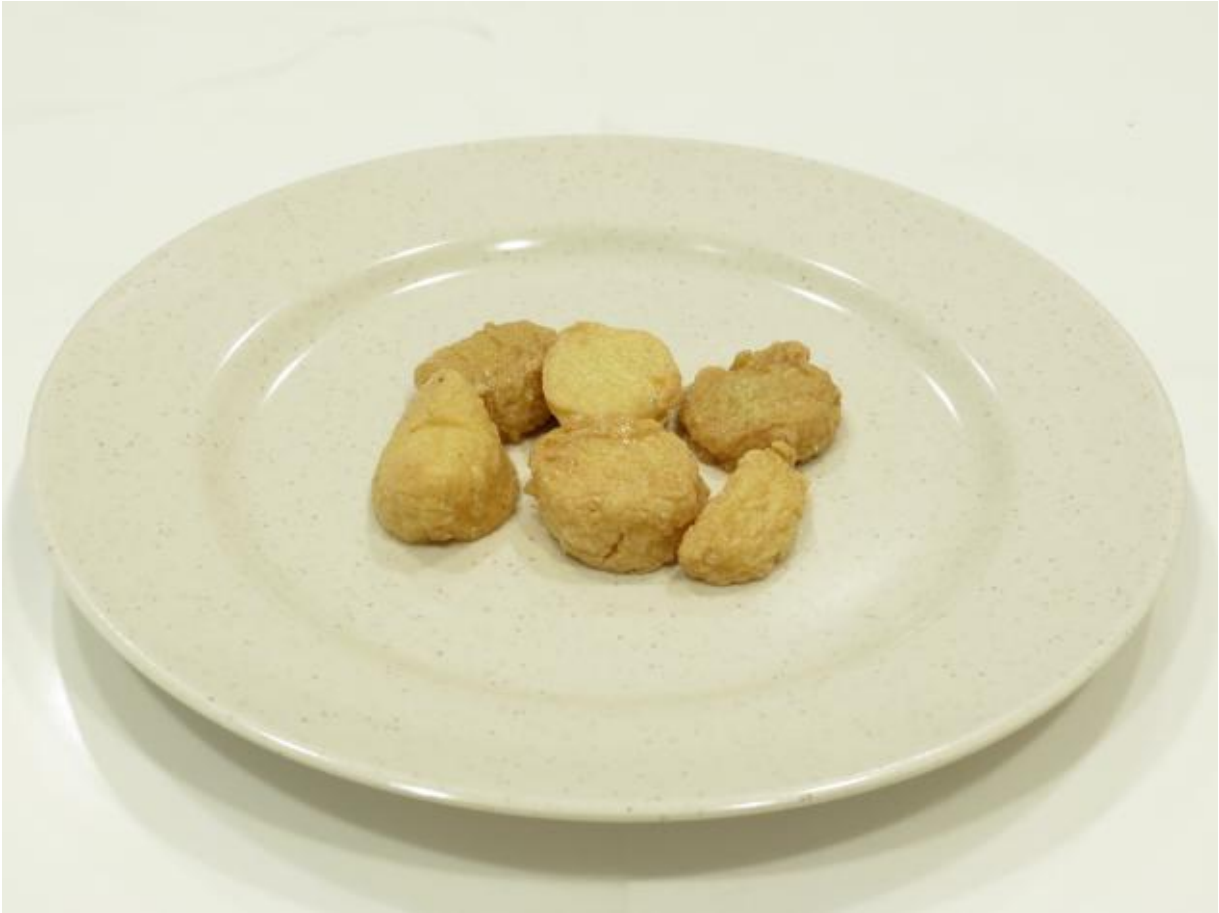

Long bean (FA0127)

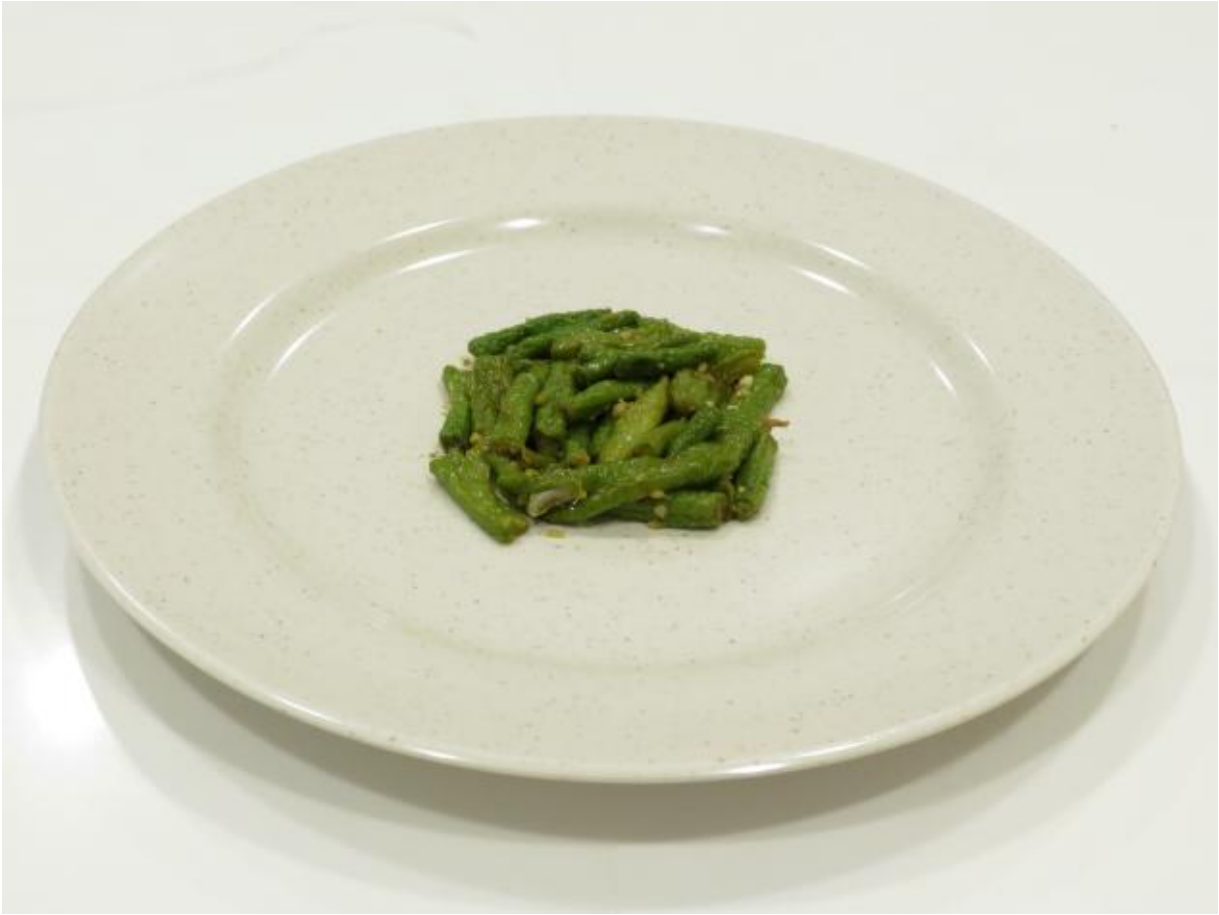

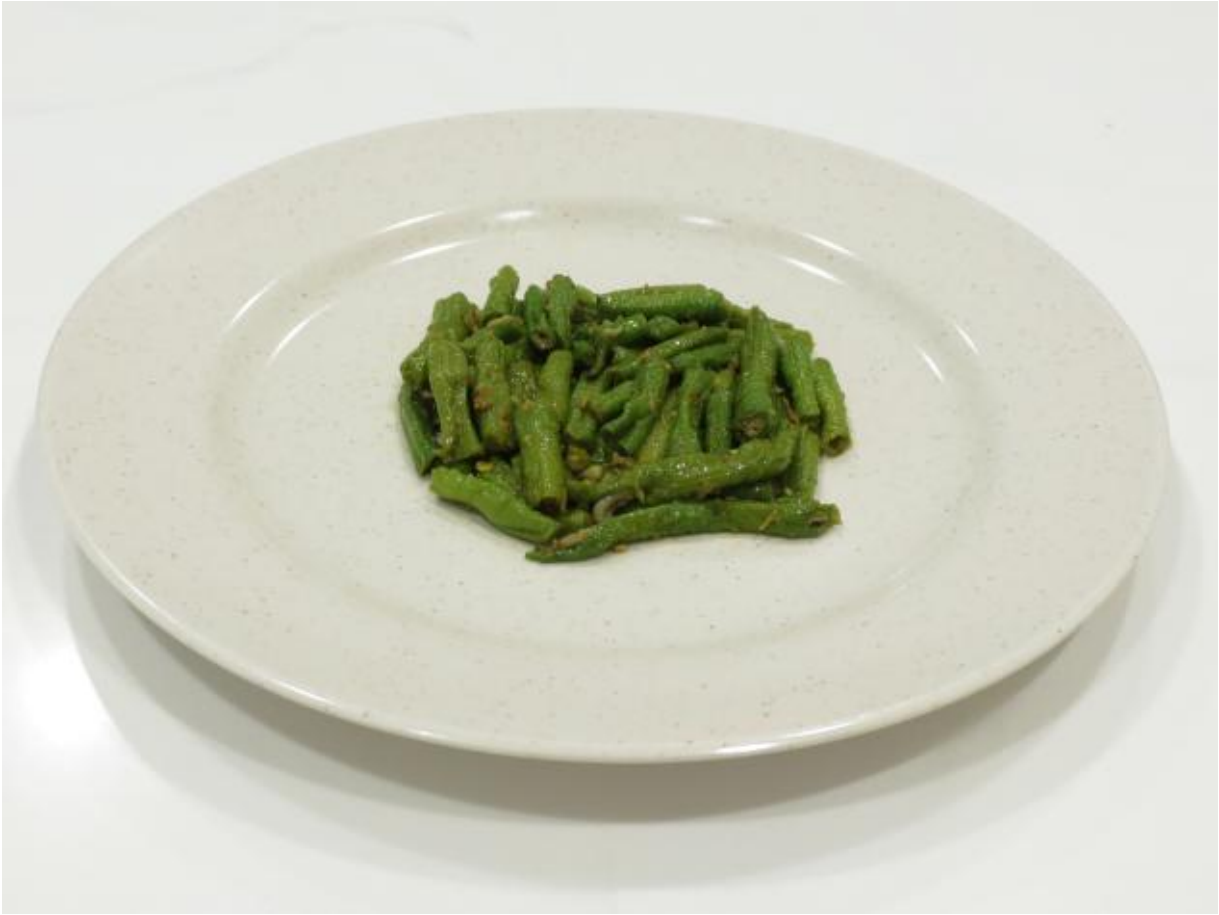

Tofu, firm (tau kwa) (FA0080)

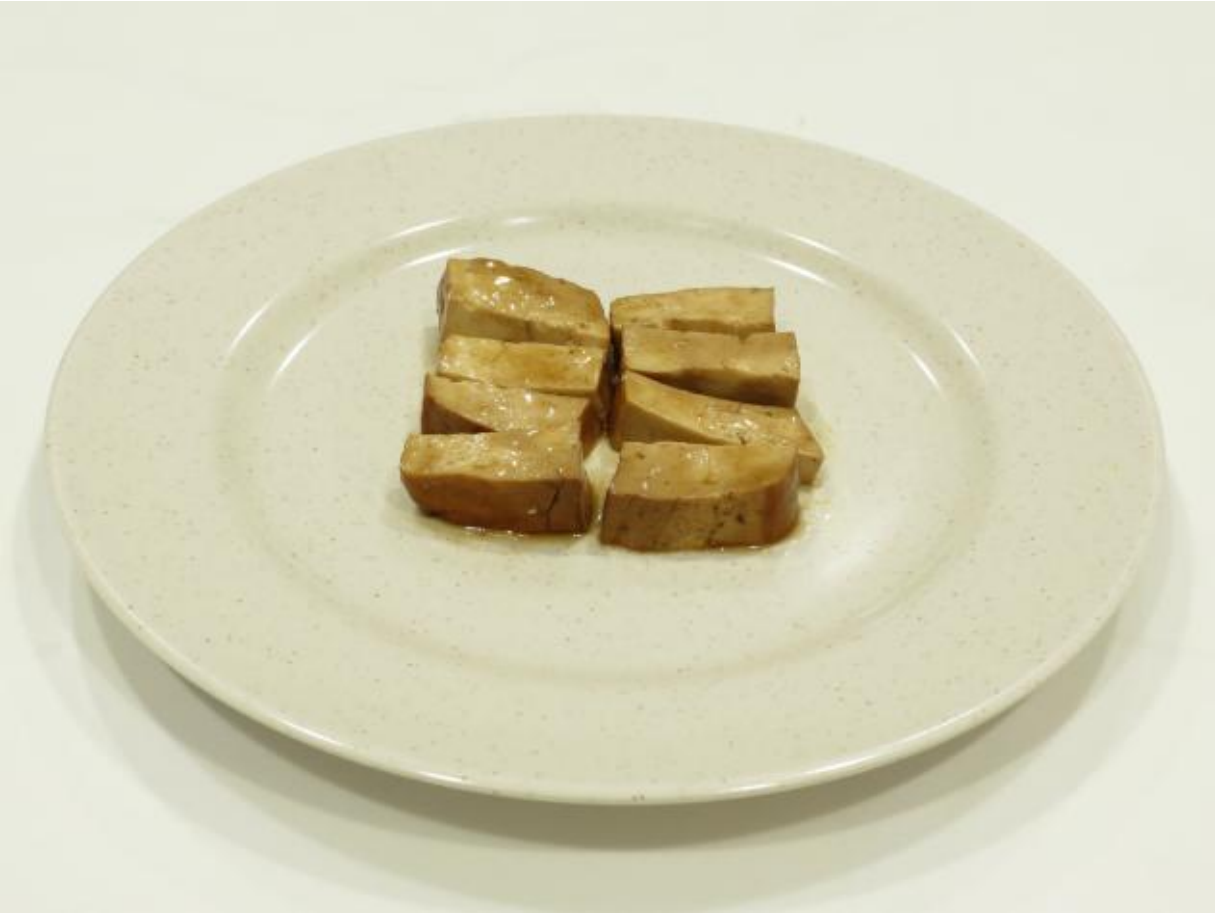

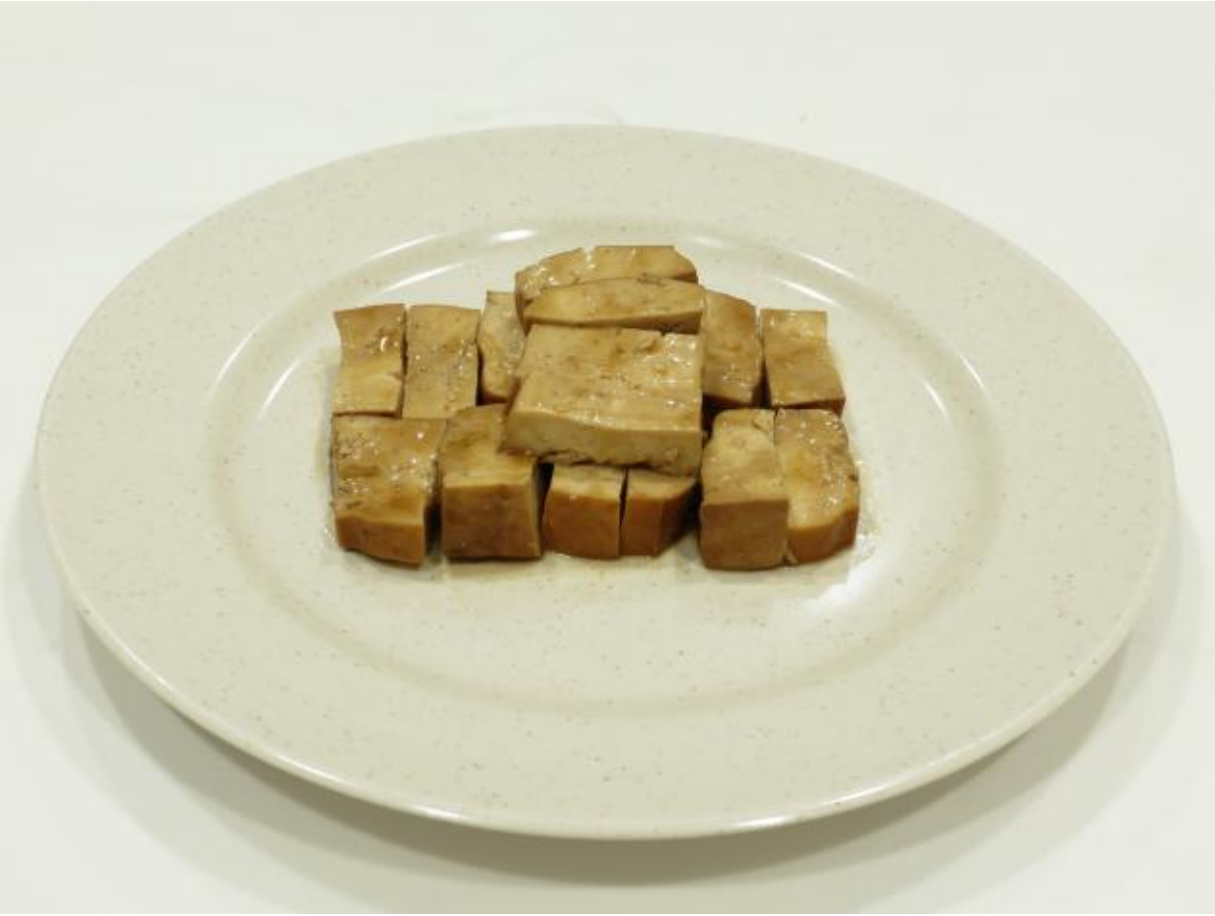

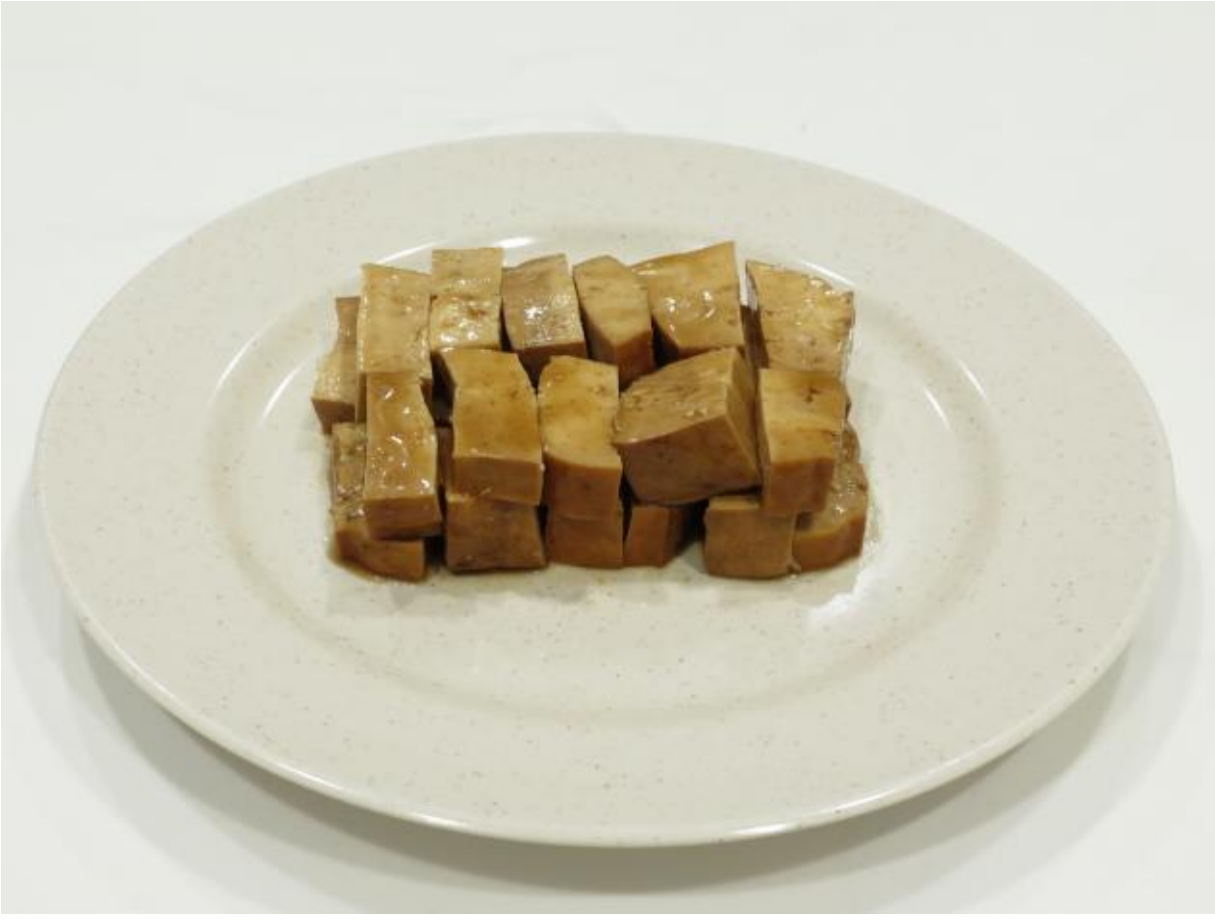

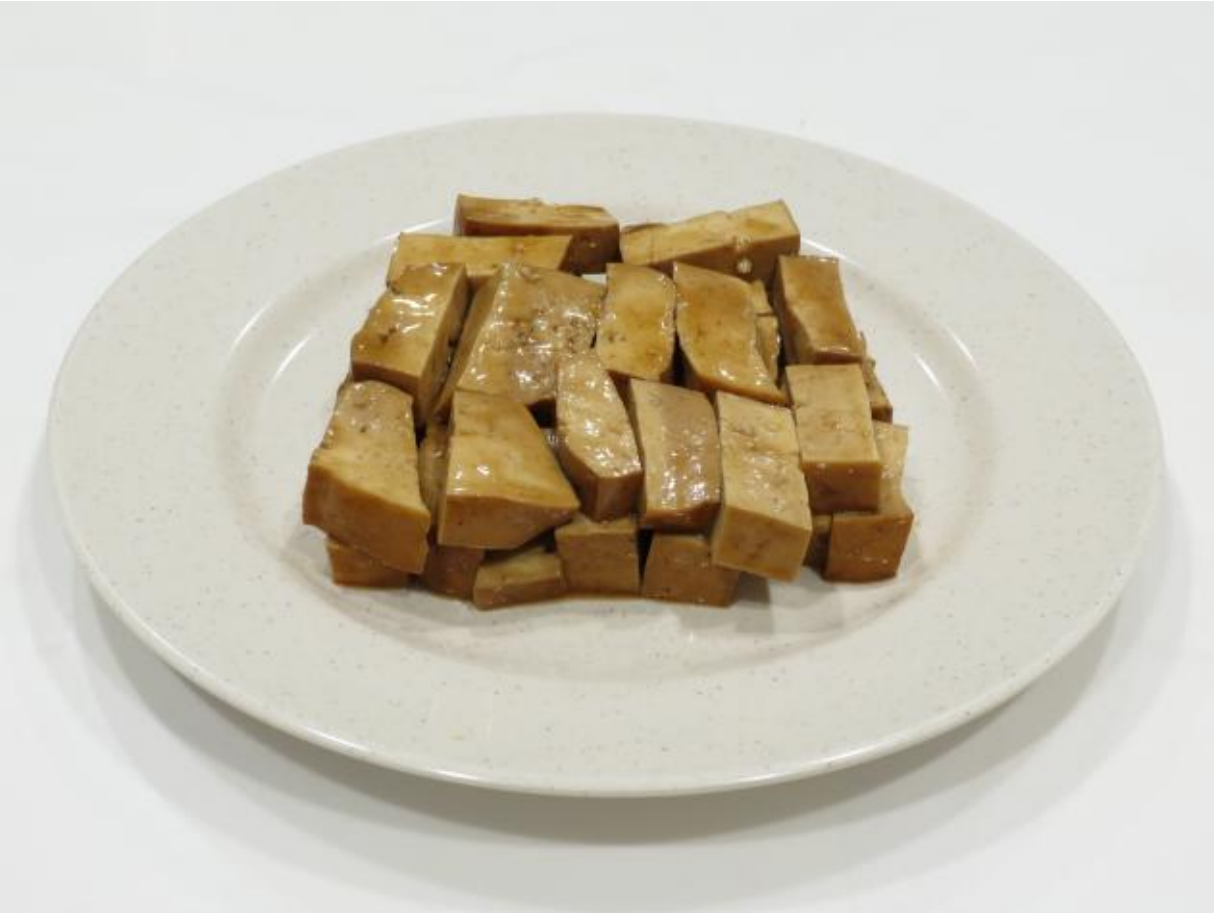

Tofu, silken / soft (FA0150)

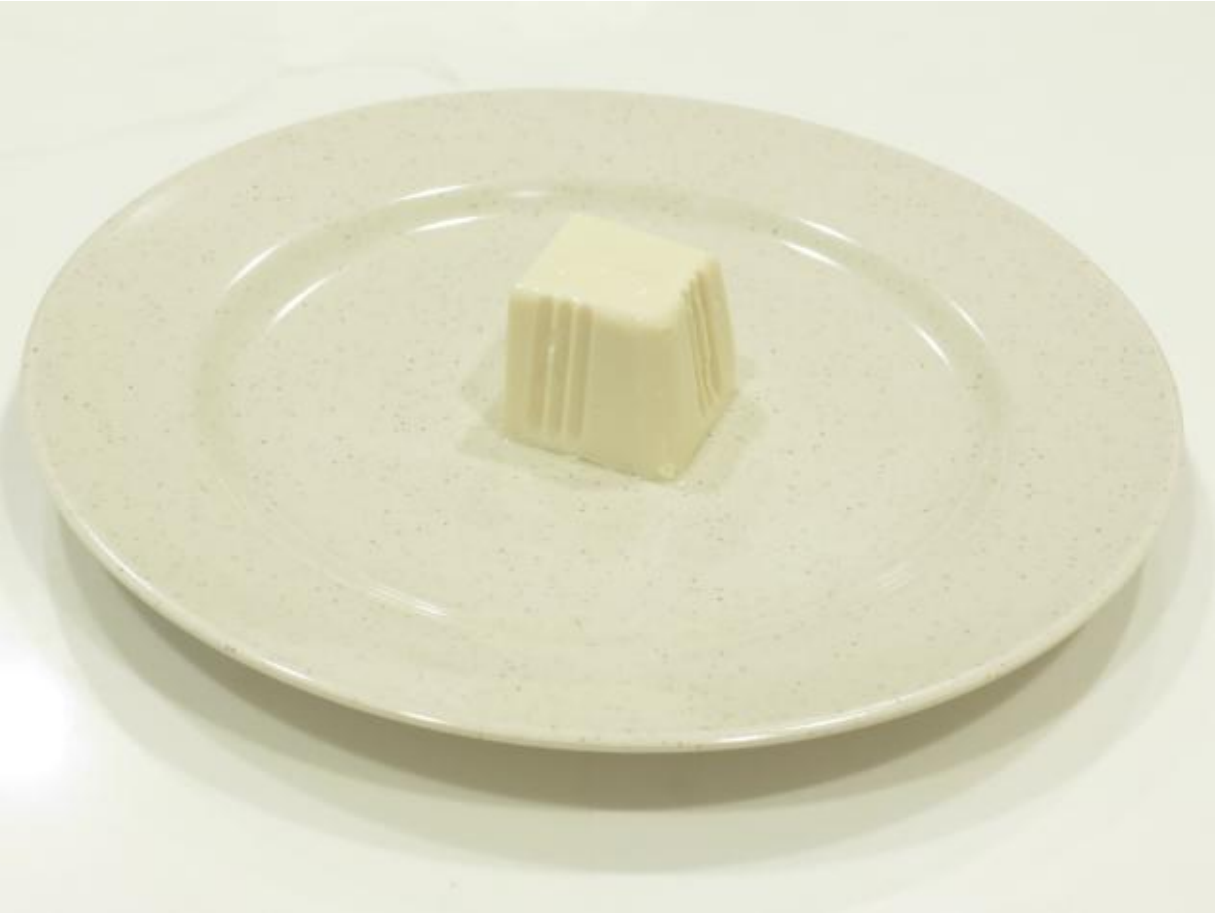

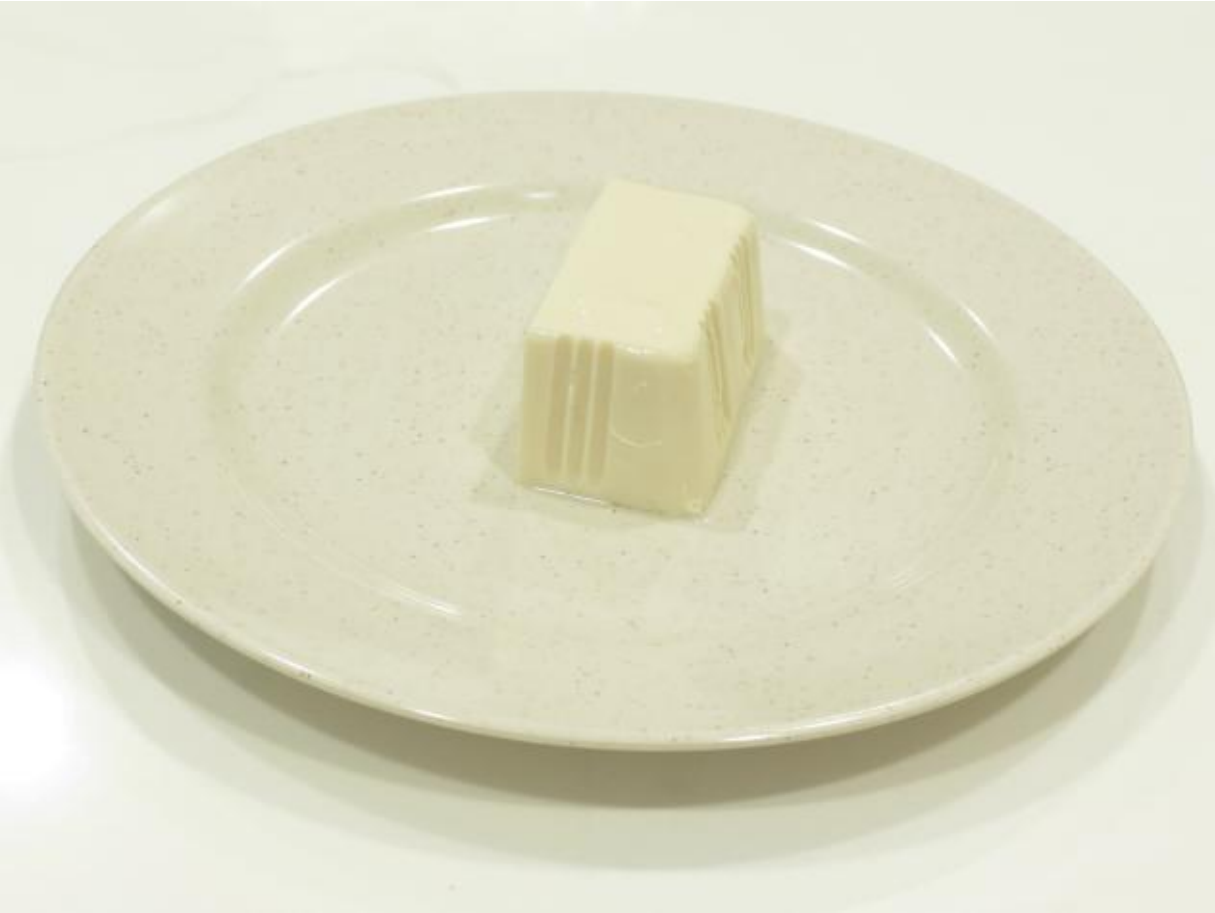

Carrot, shredded (FA0132)

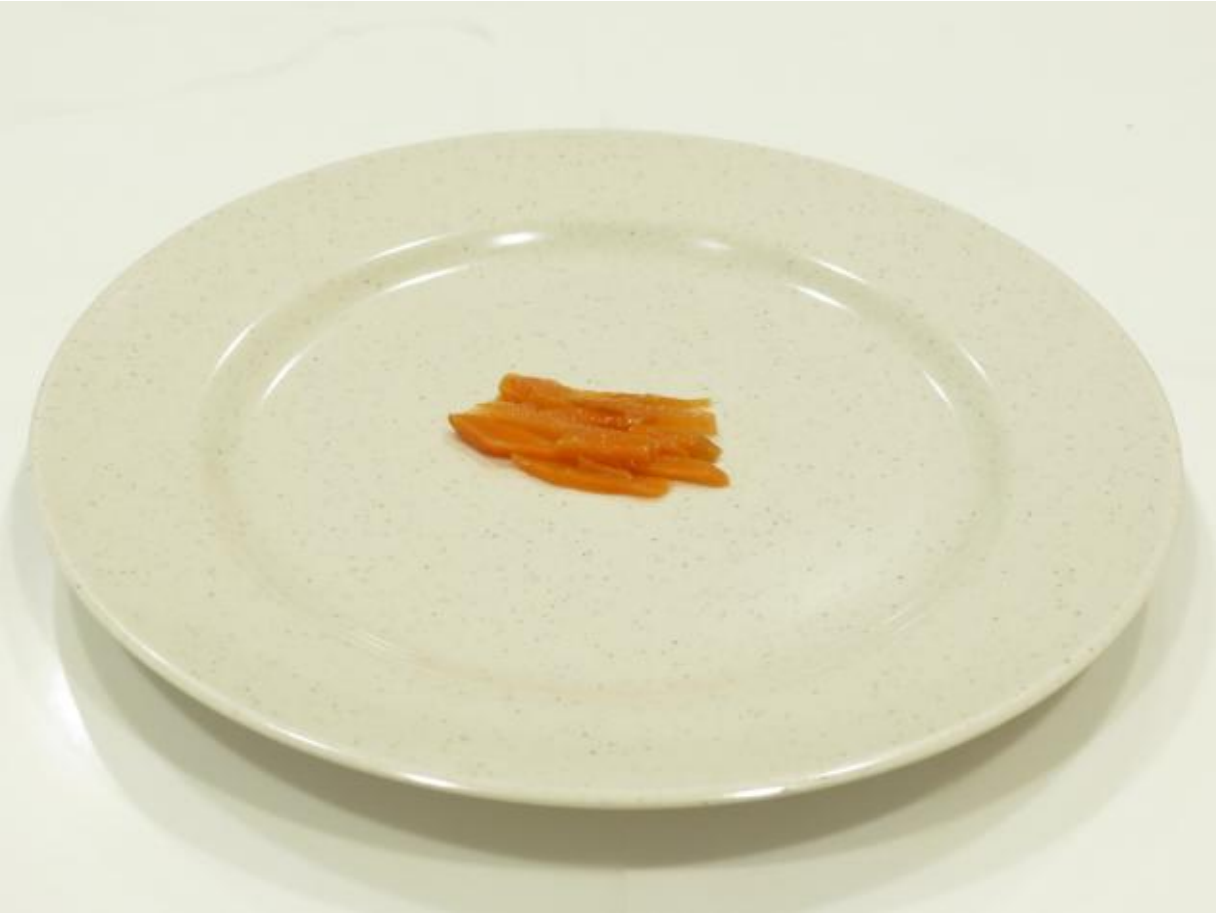

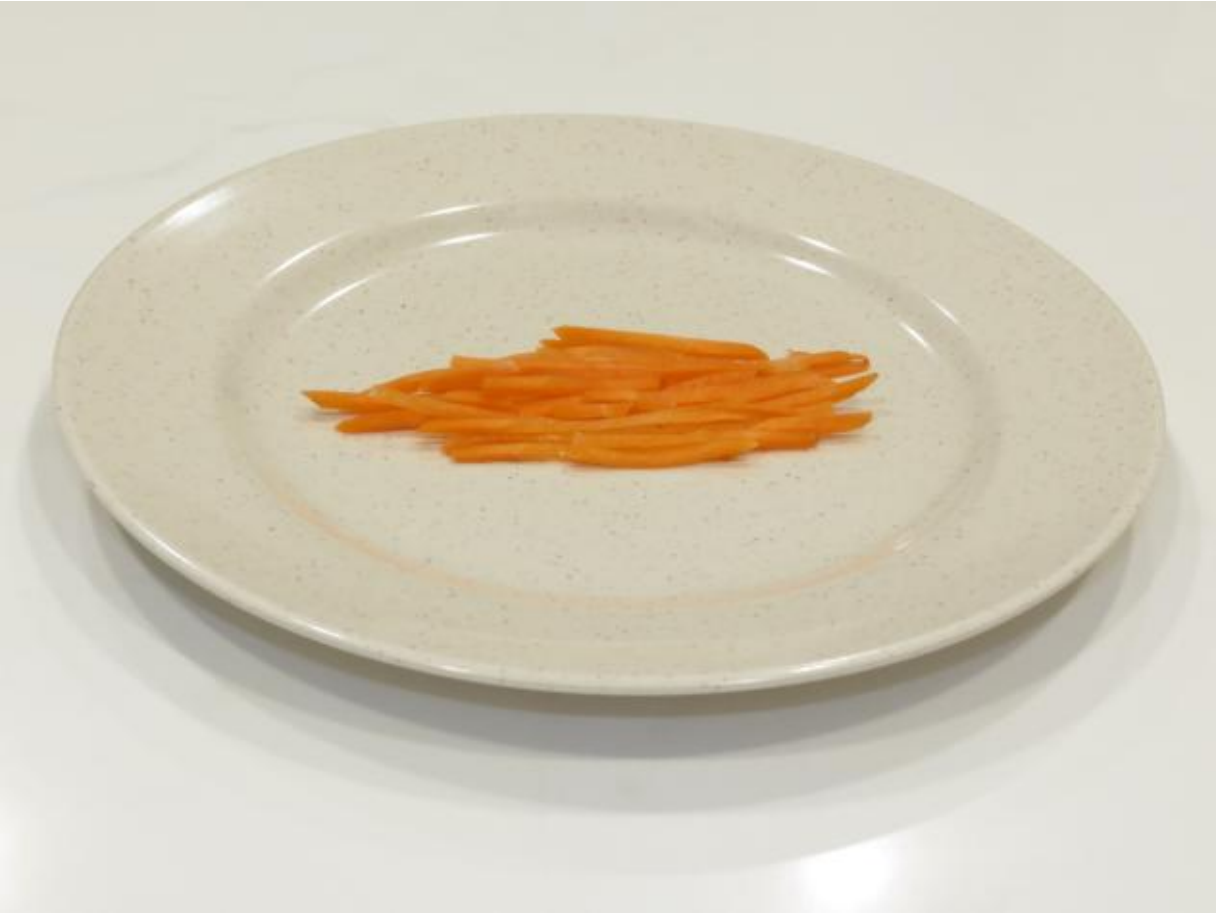

Fries, shoestring (FA0164)

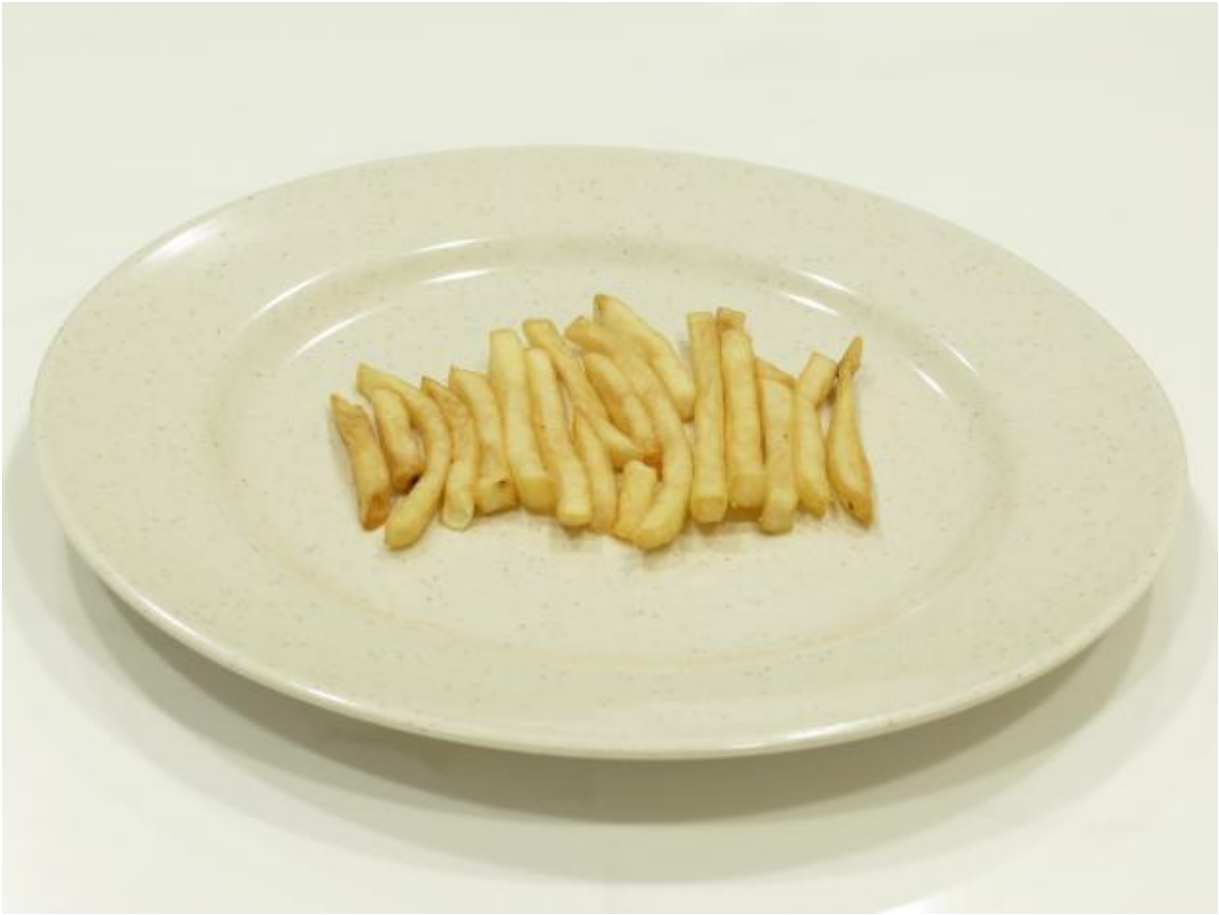
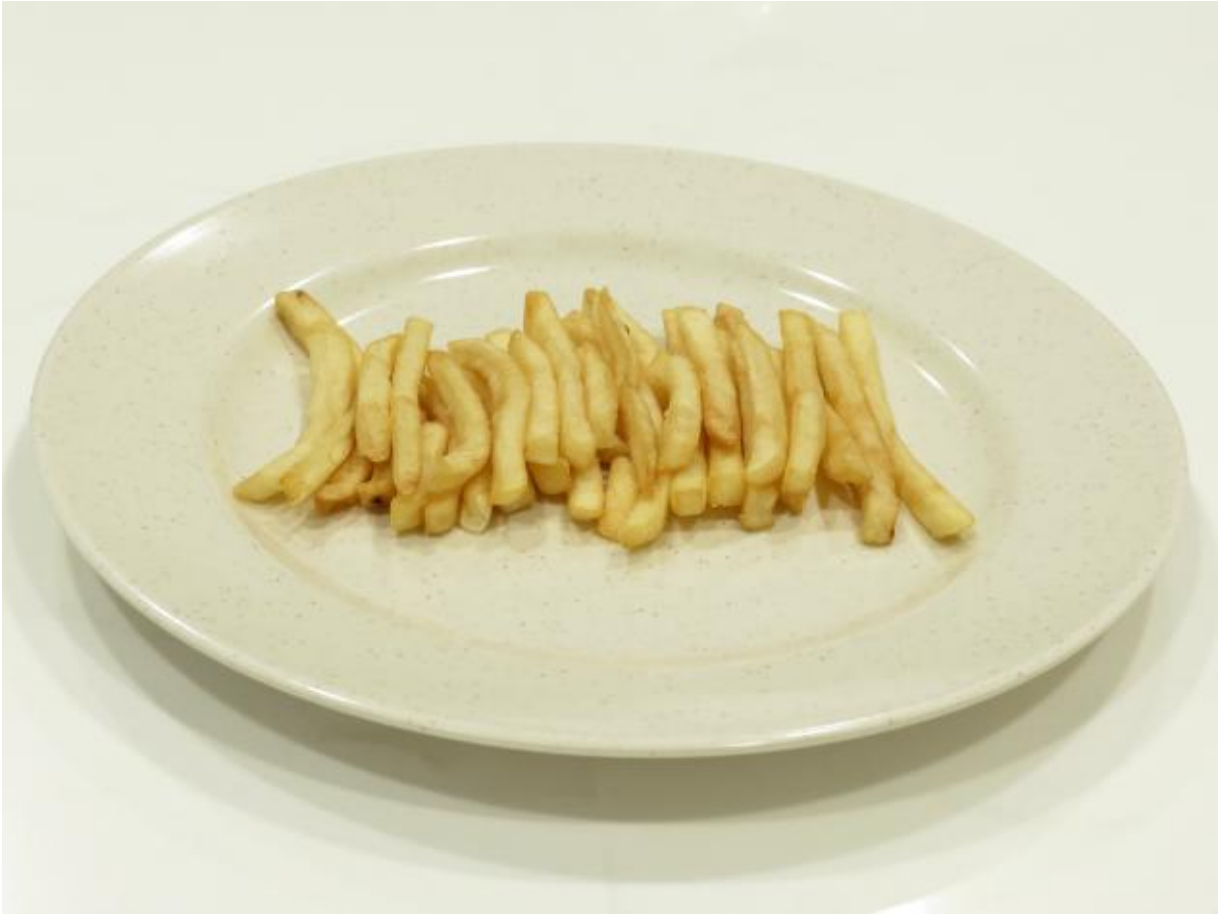
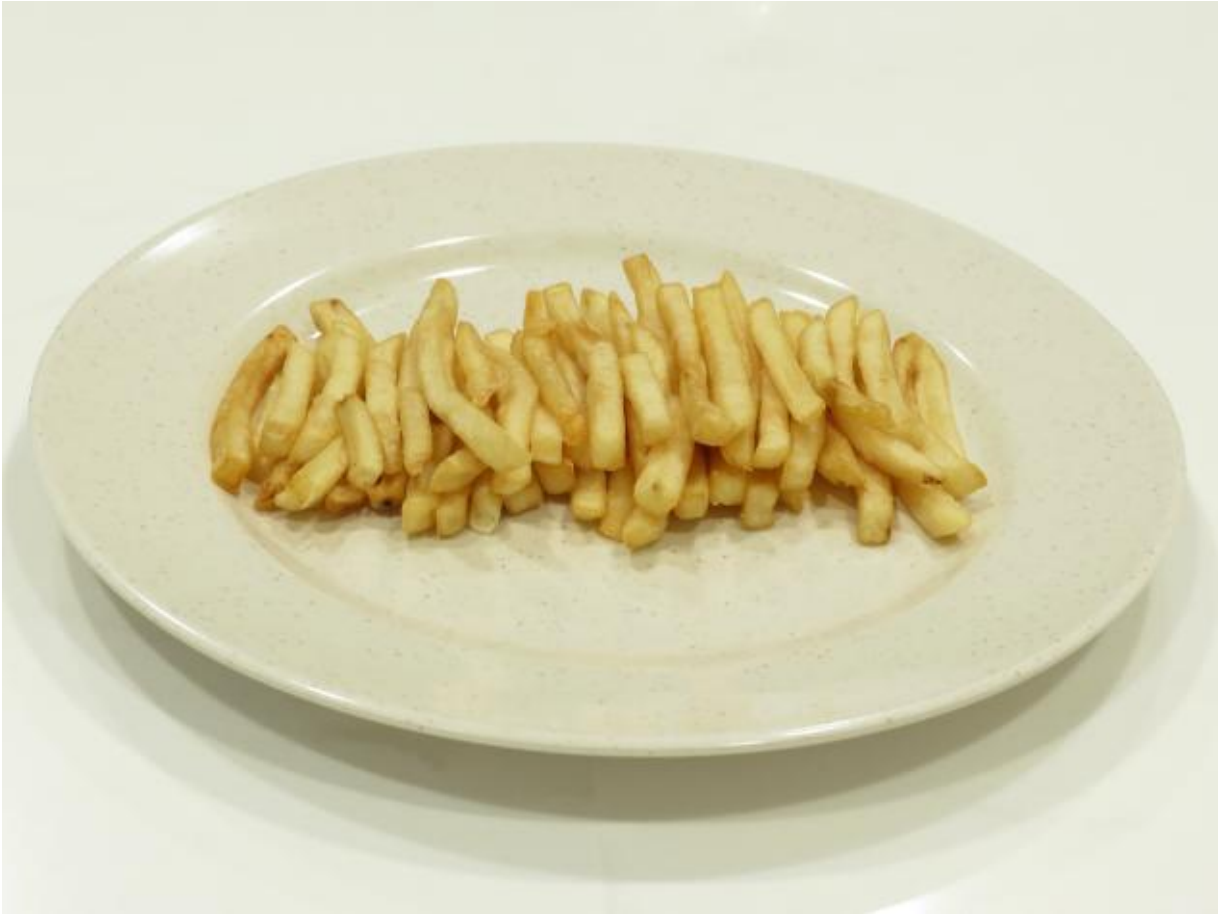
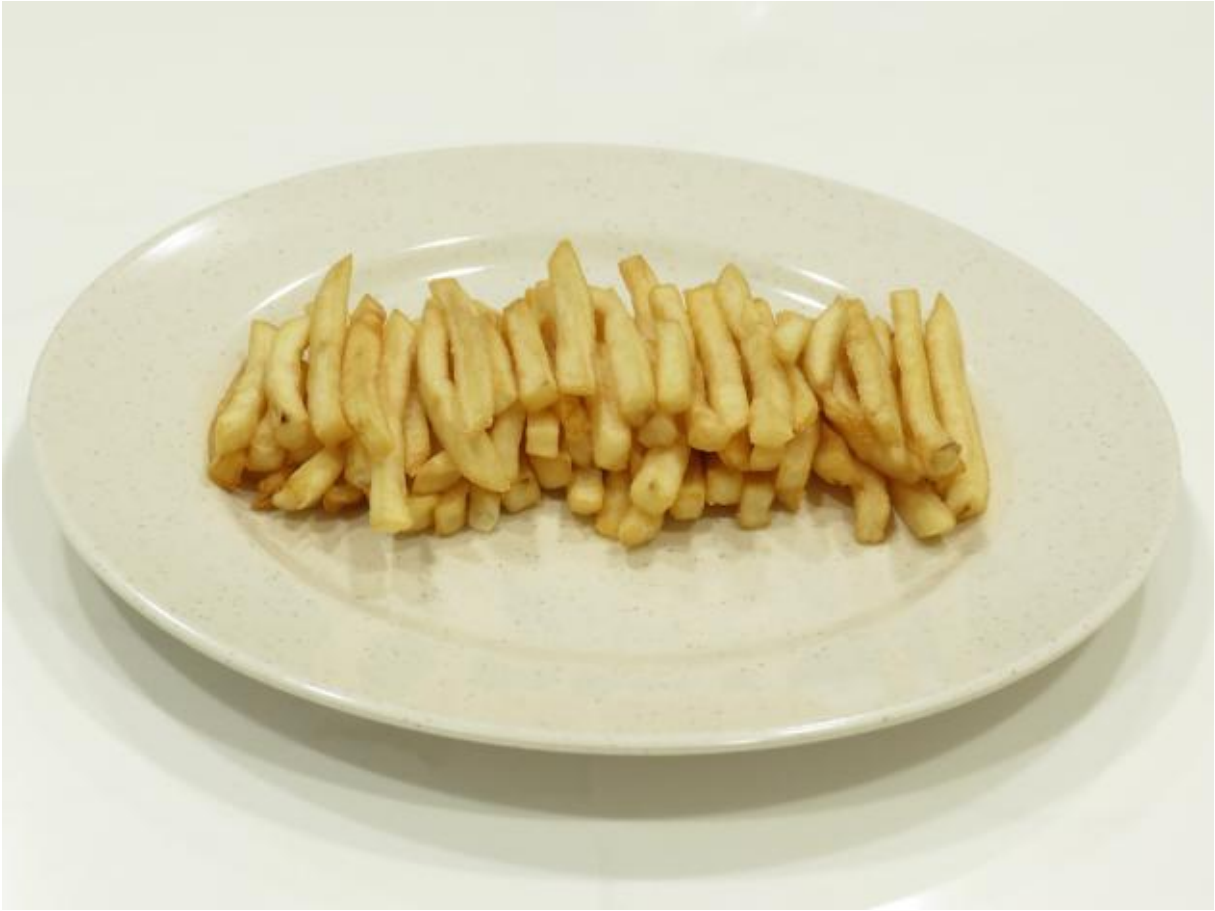

Fries, crinkle cut (FA0201)

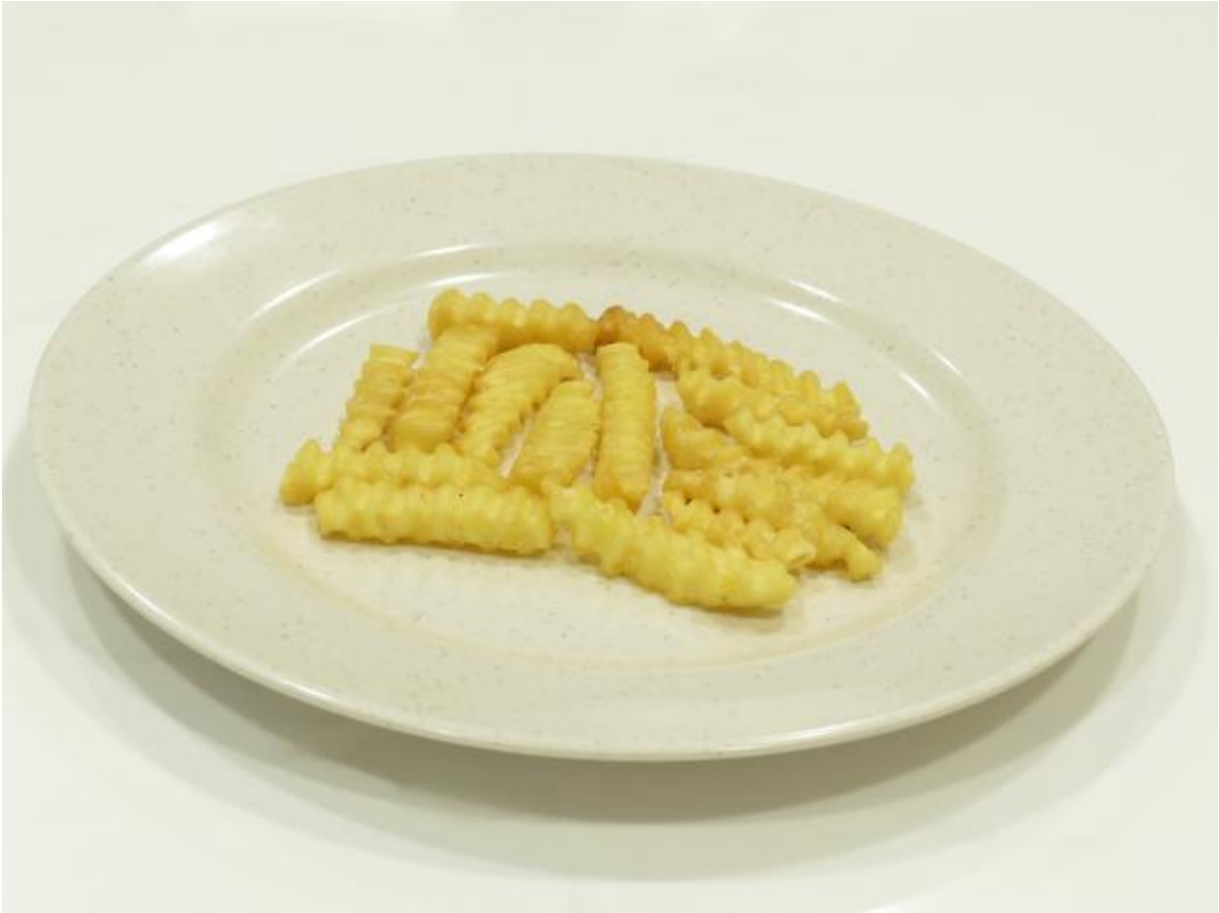
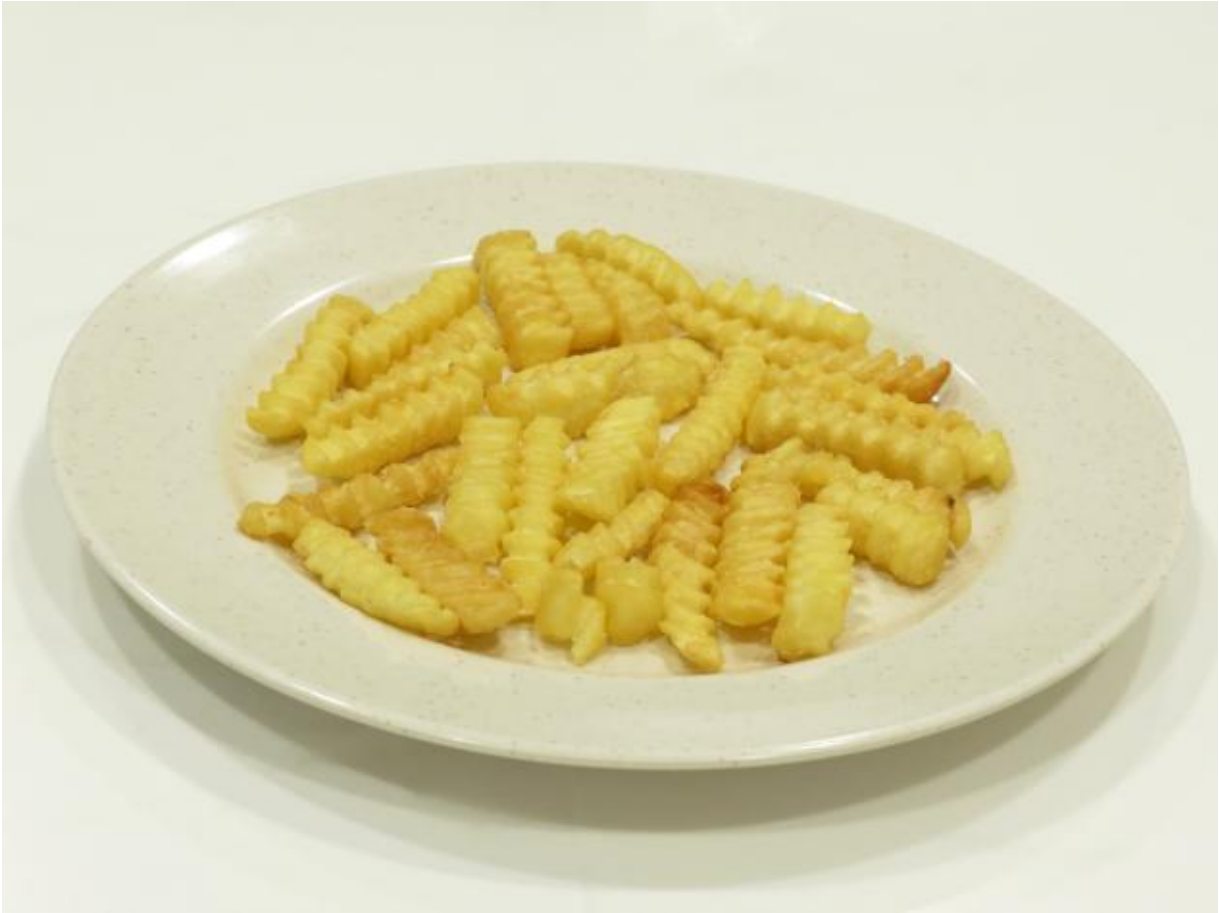
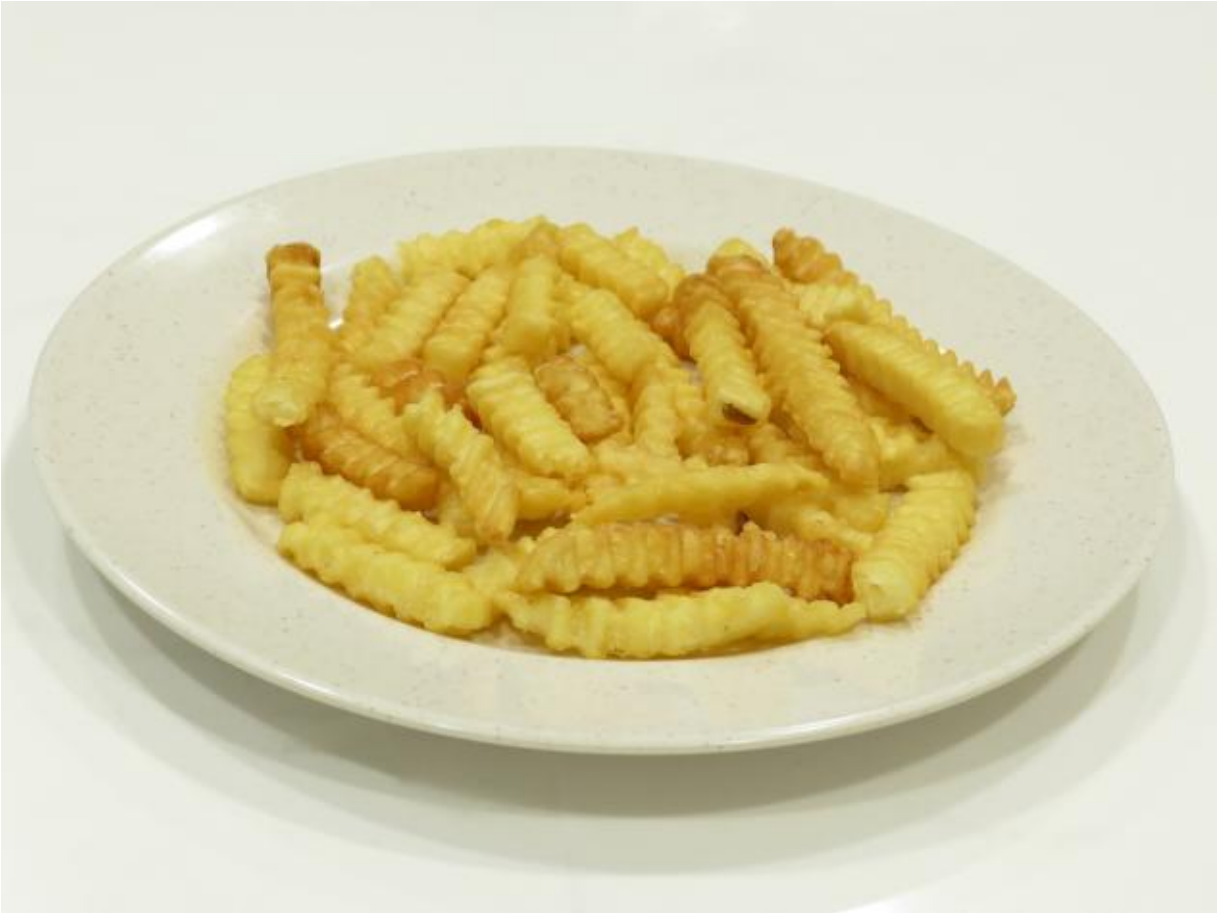
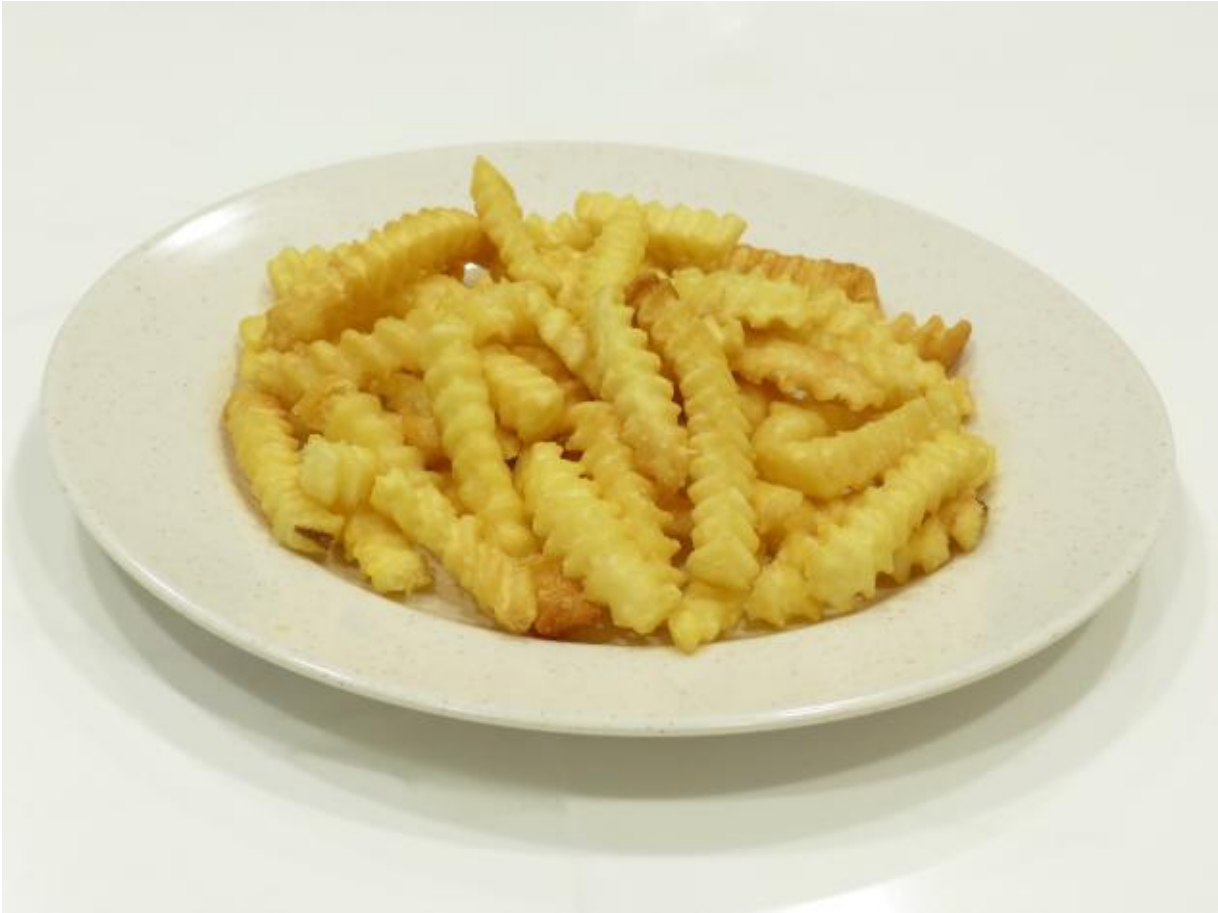

Fries, thick cut (FA0058)

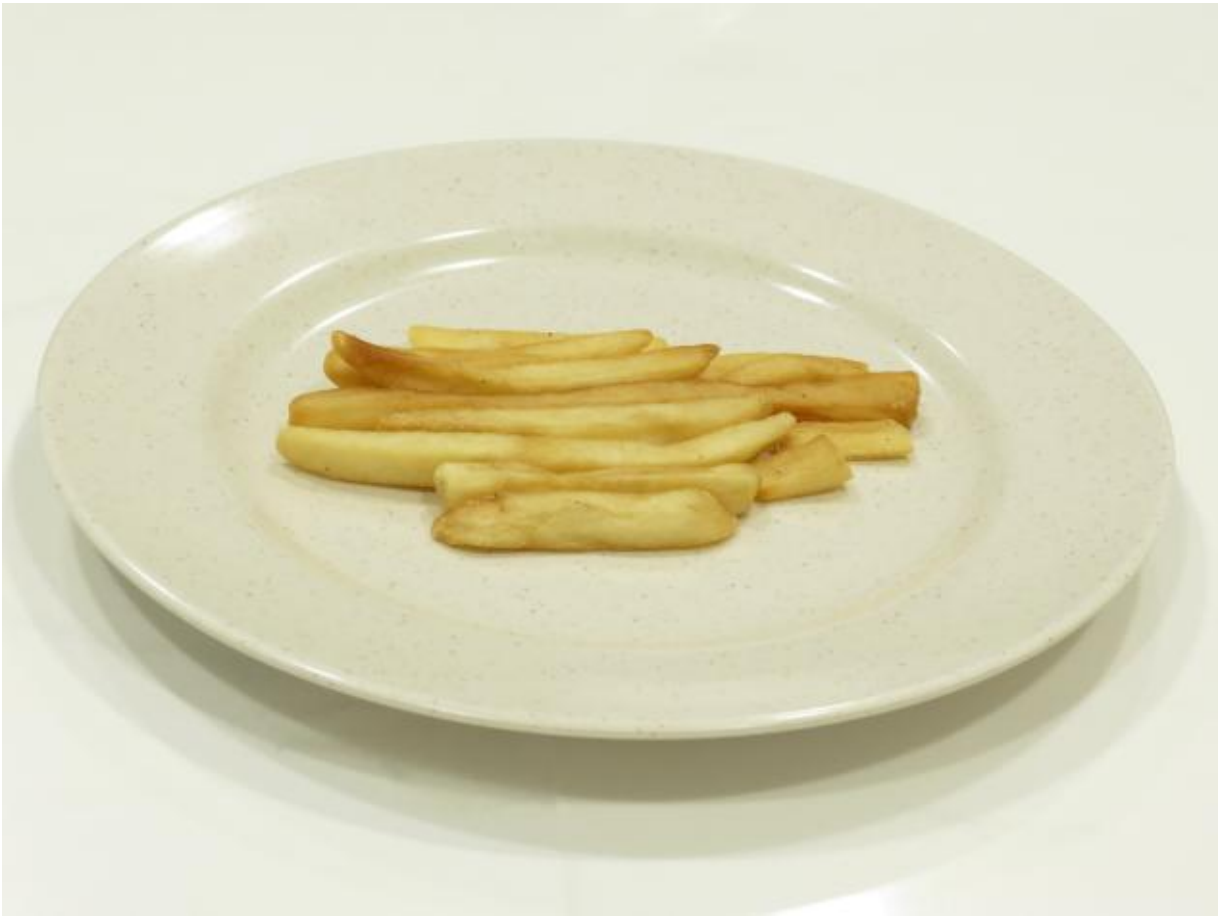

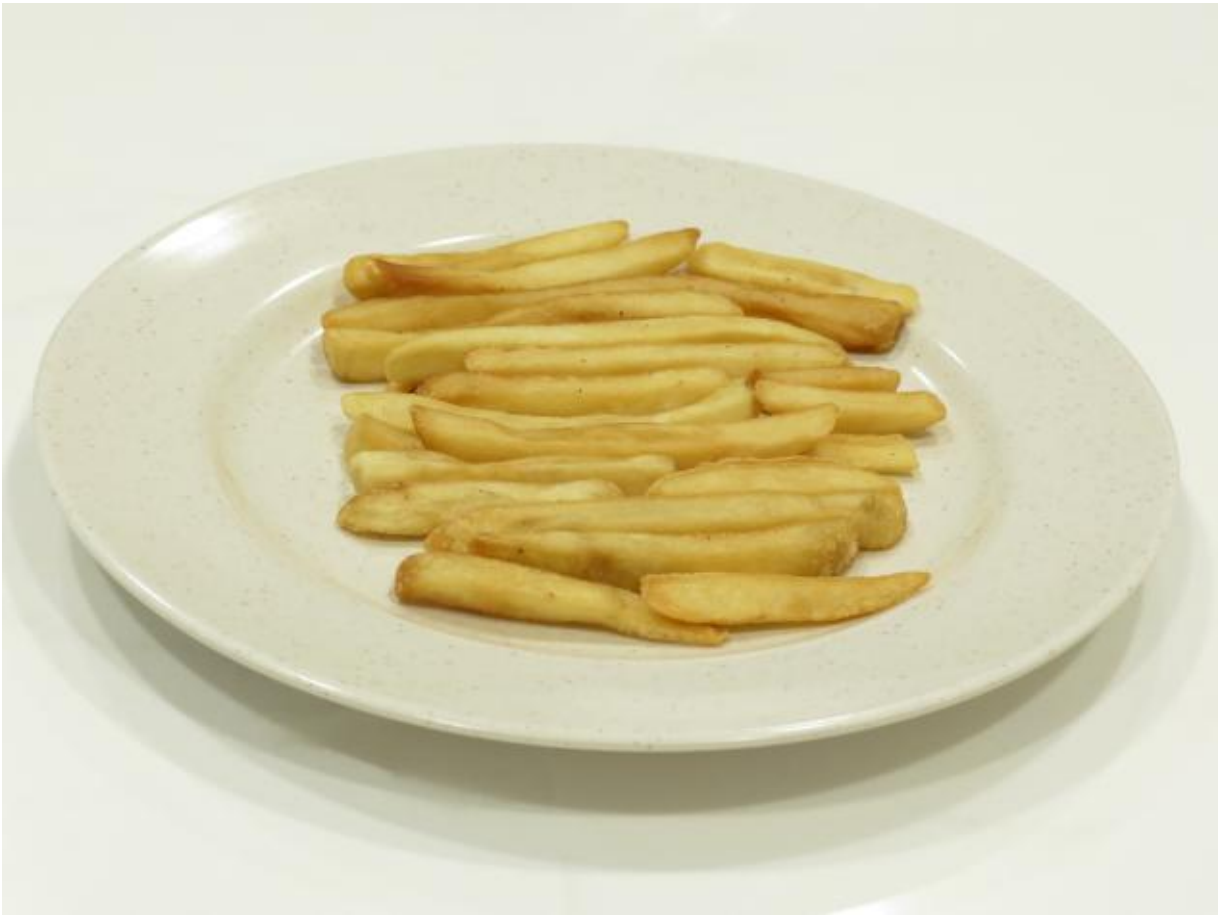

Potato, mashed (FA0170)

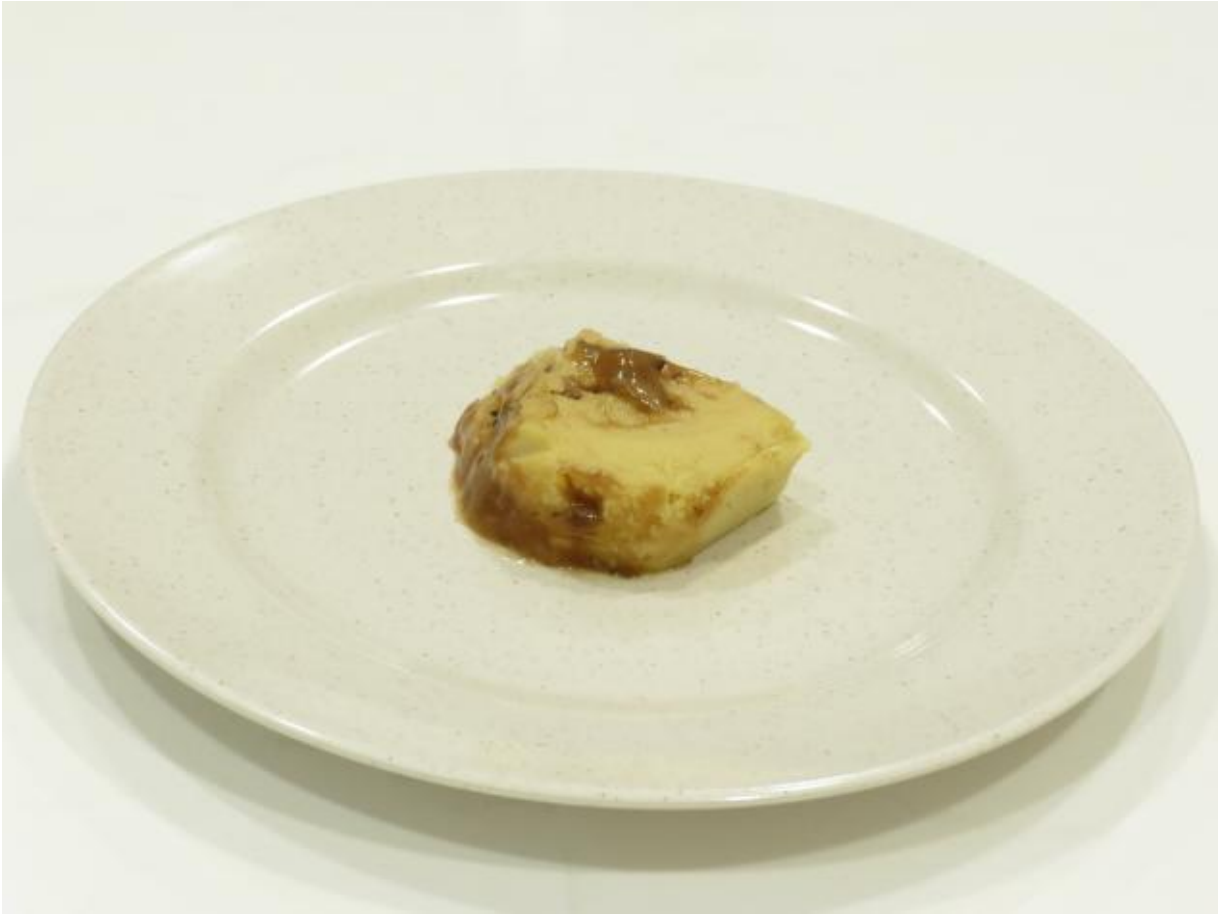

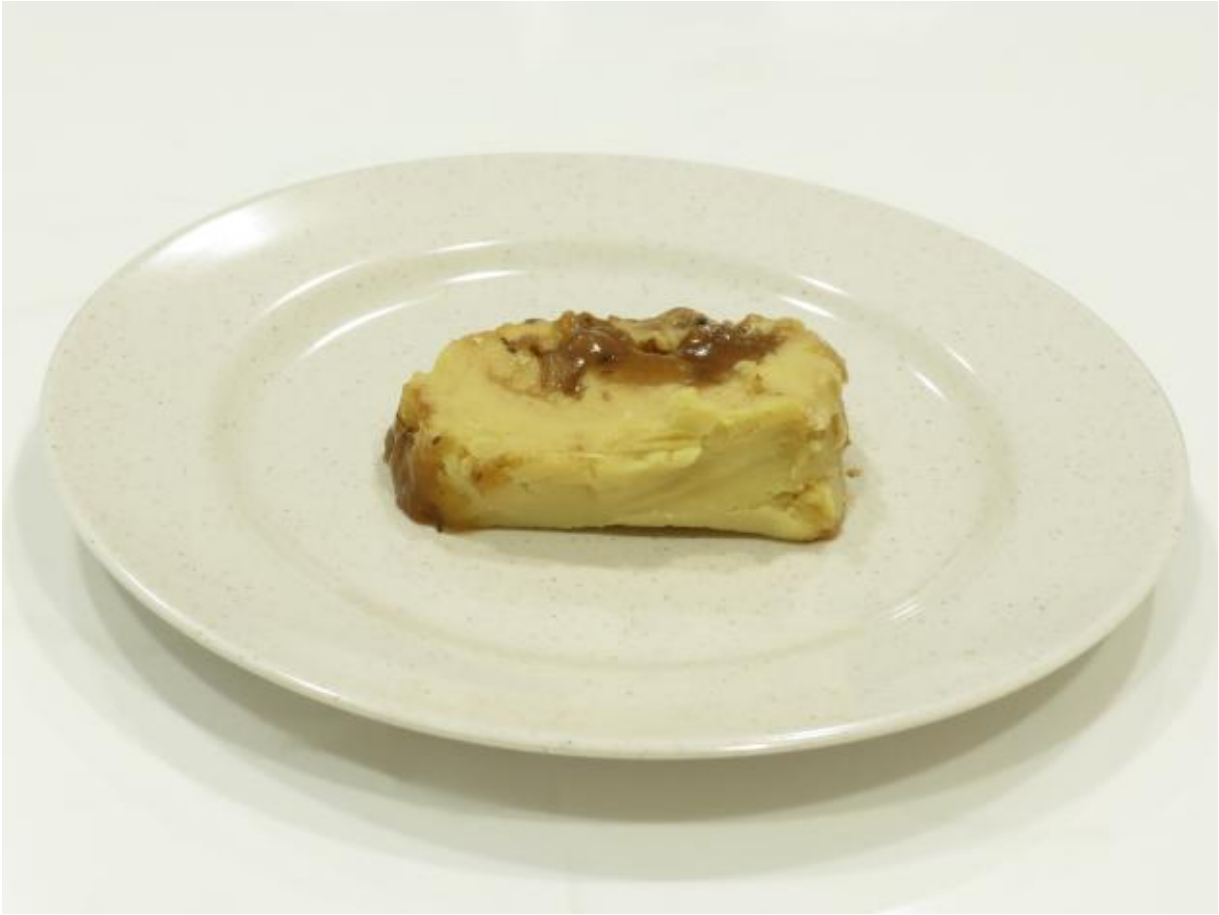

Potato, sliced (FA0126)

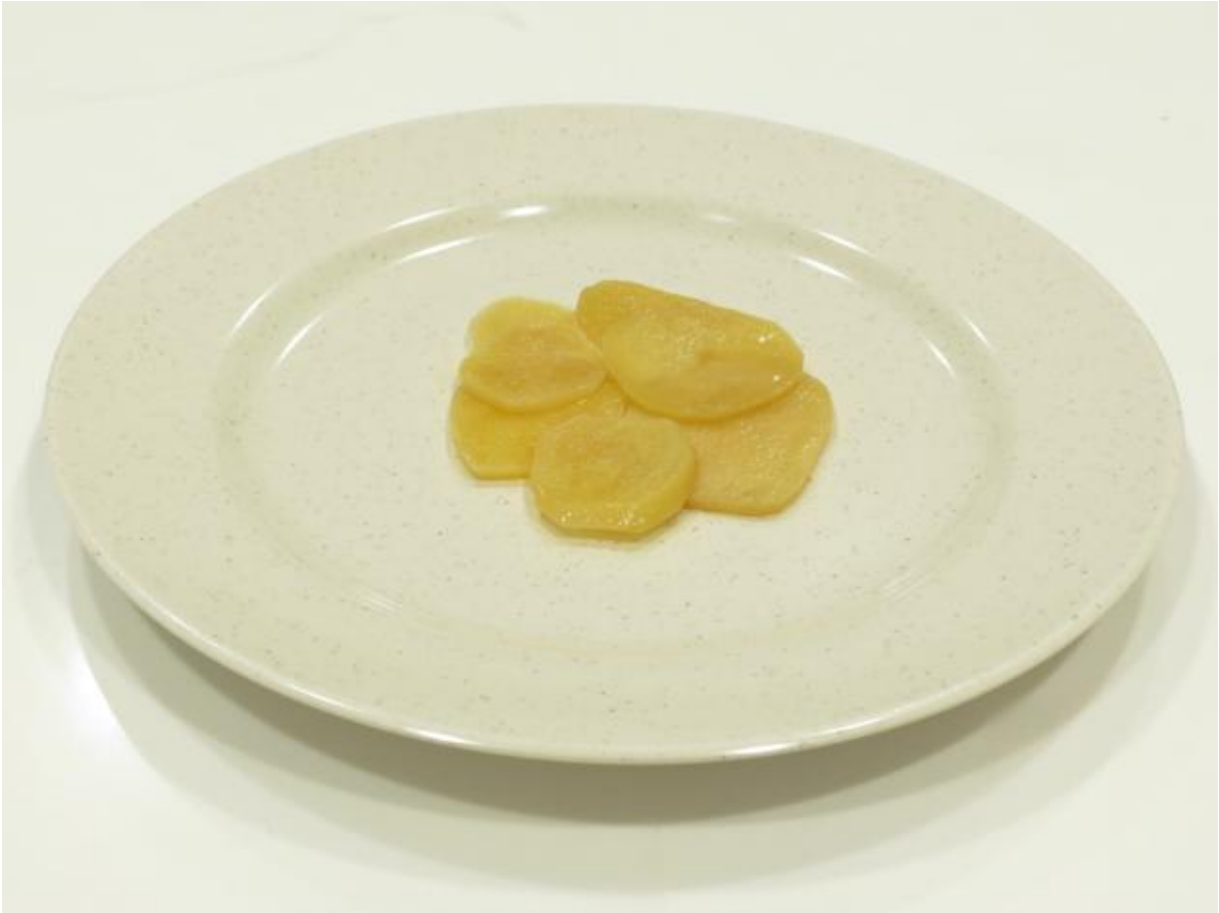
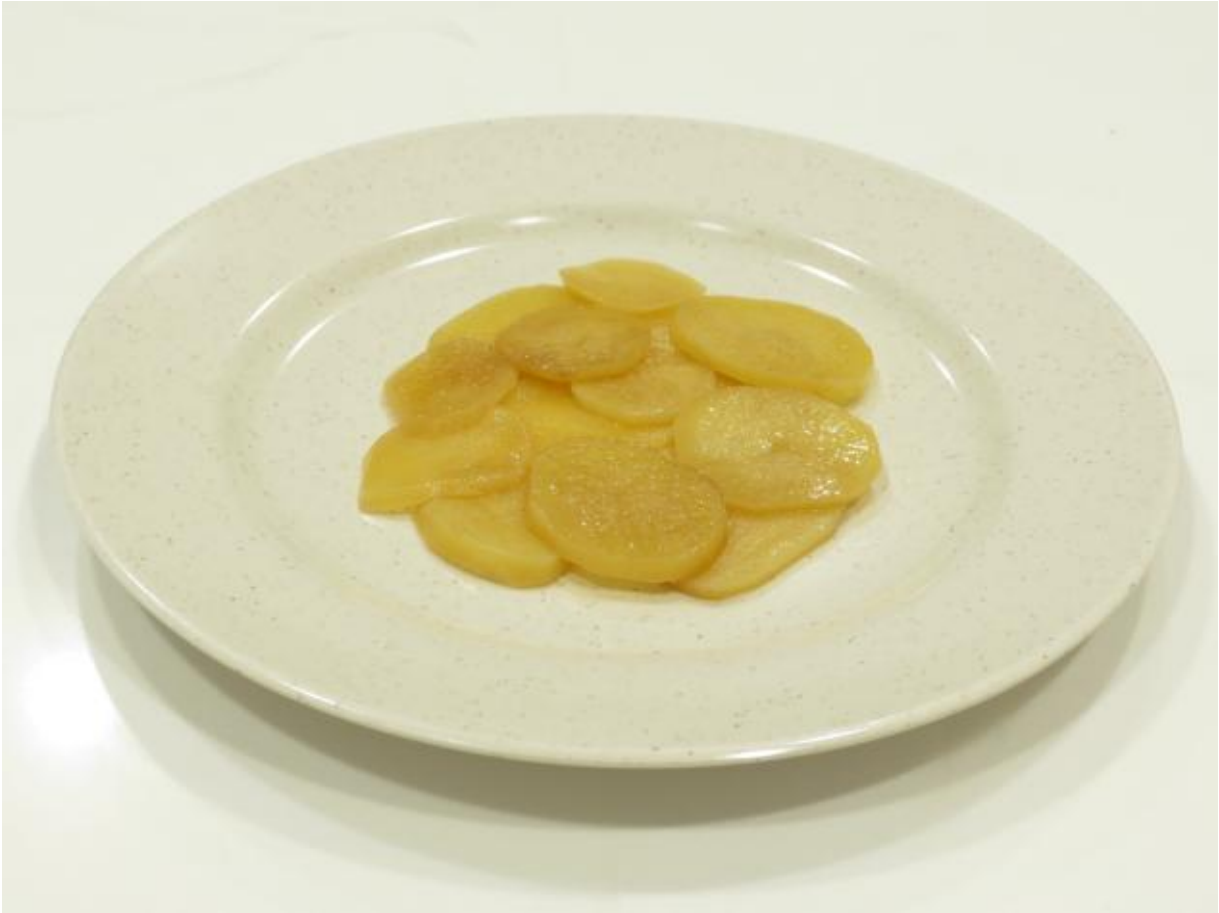
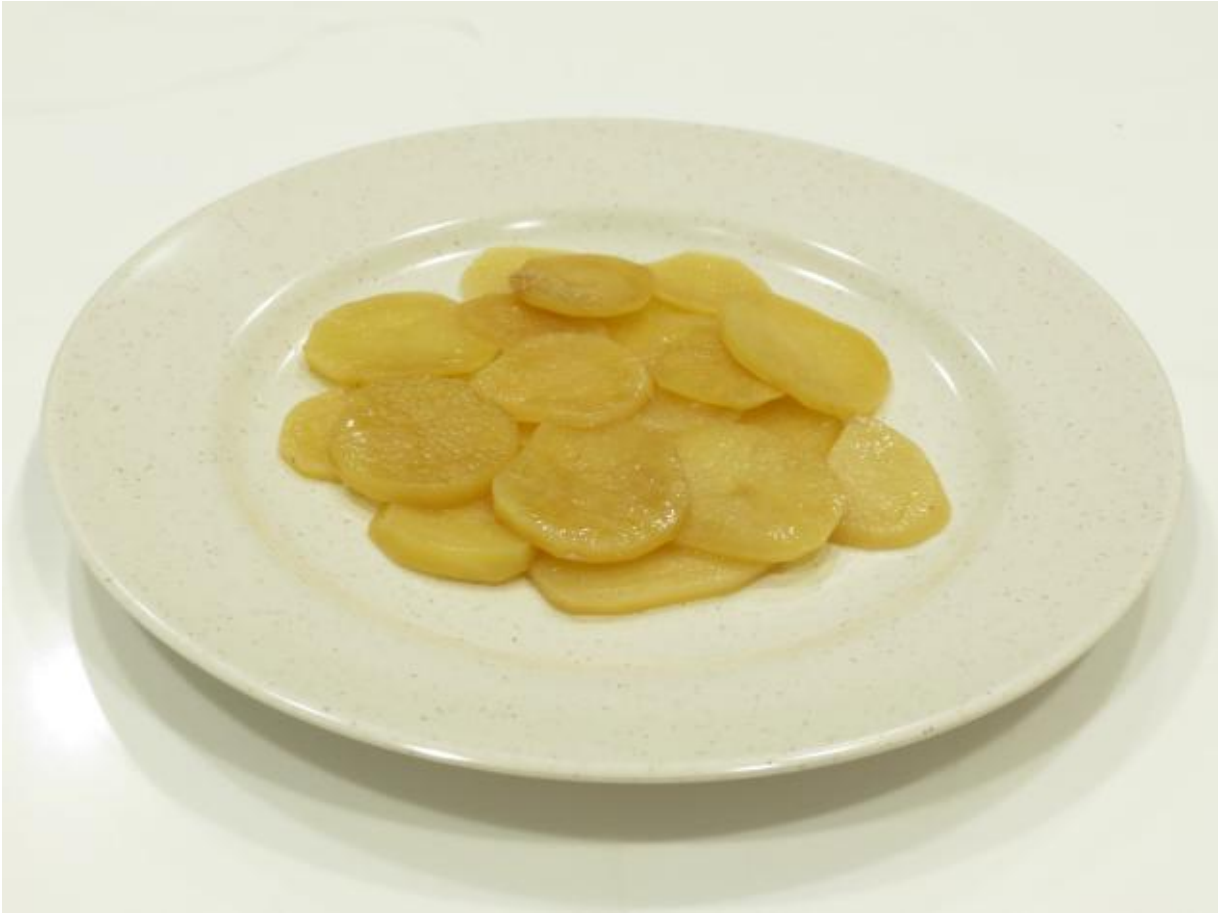
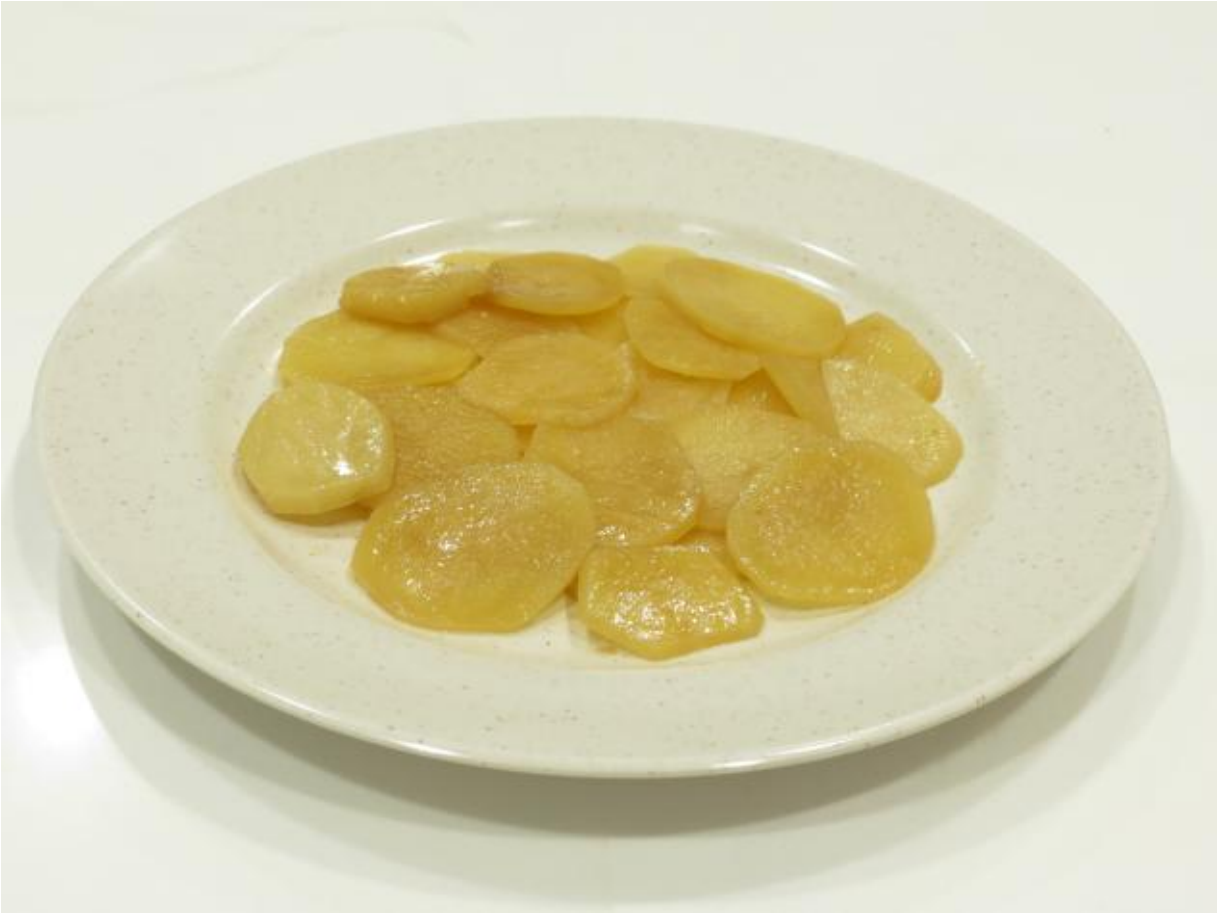

Onion, sliced (FA0138)

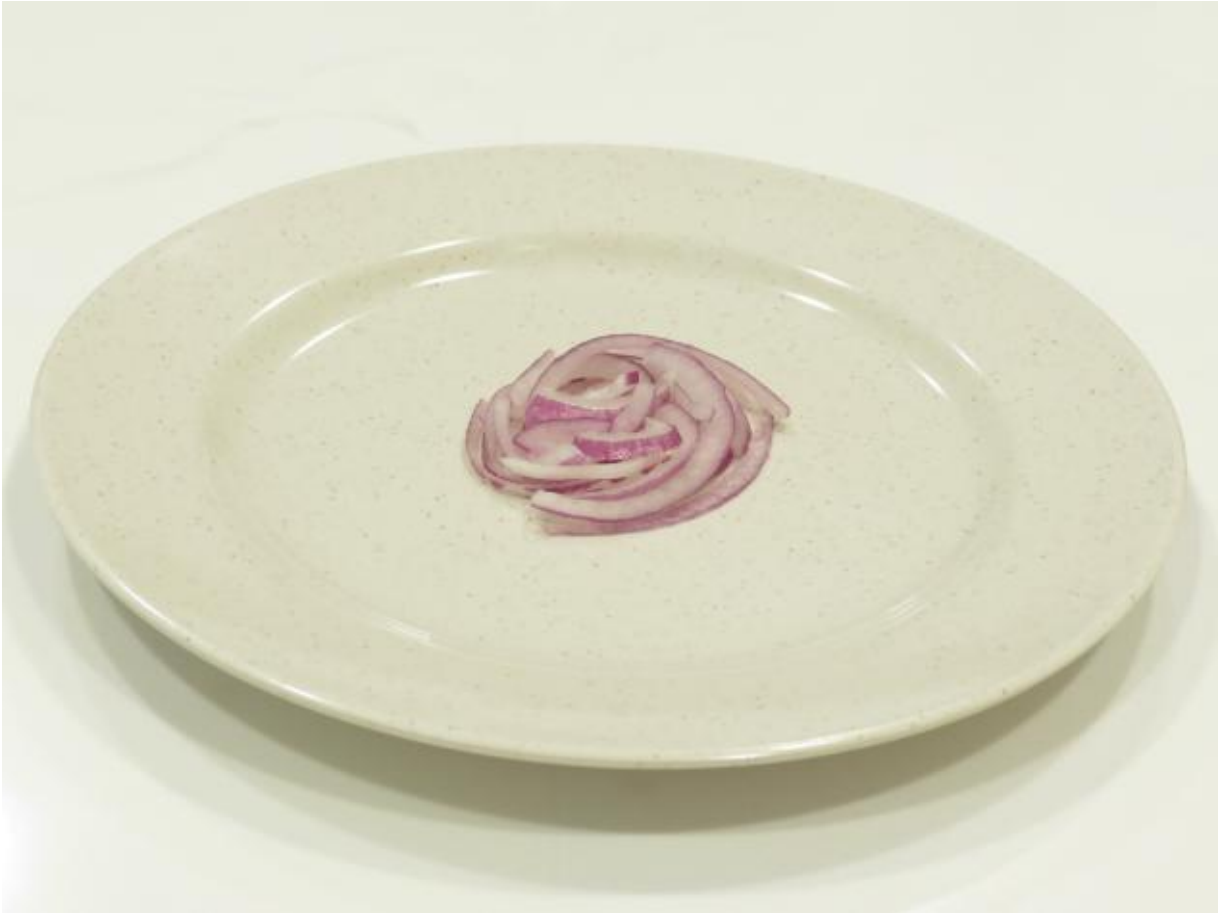

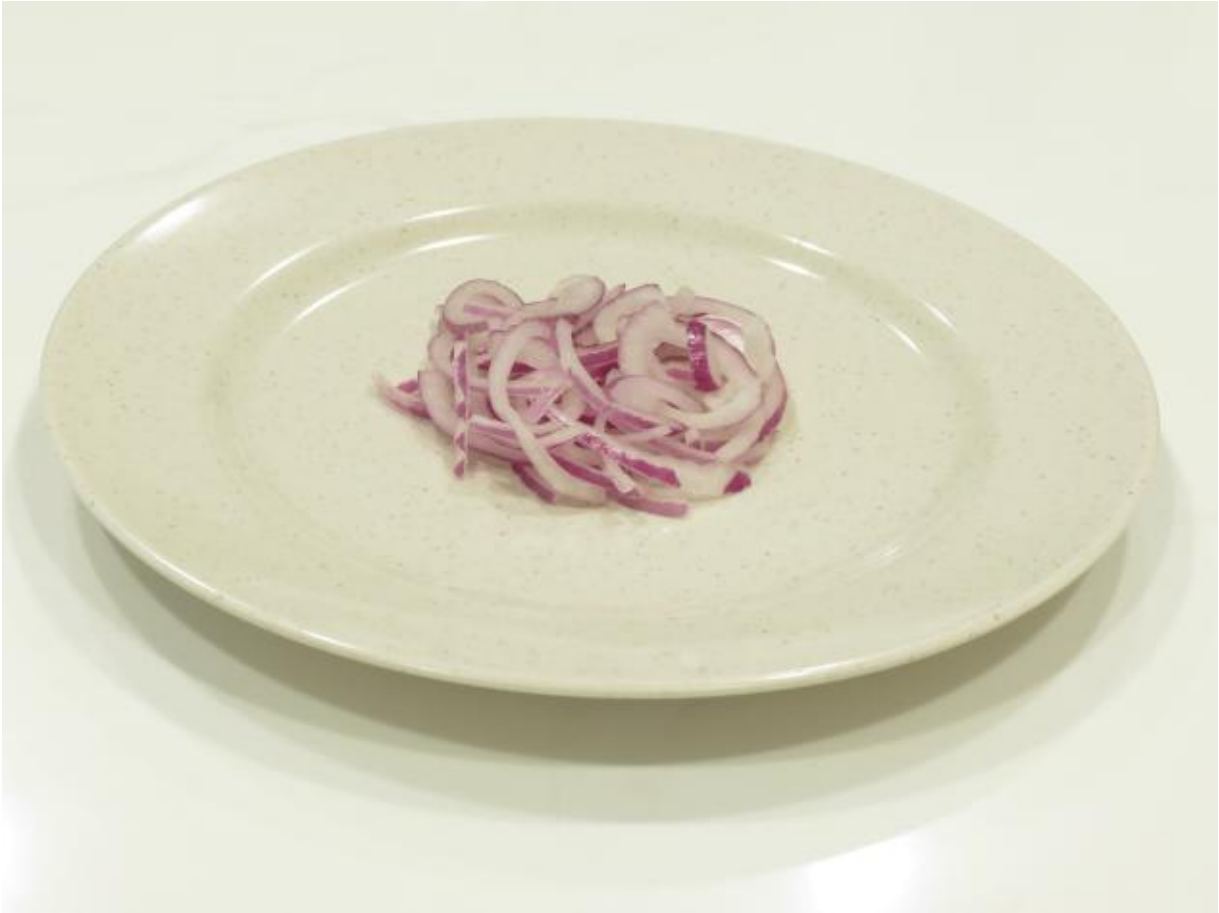

Baguette, sliced (FA0038)

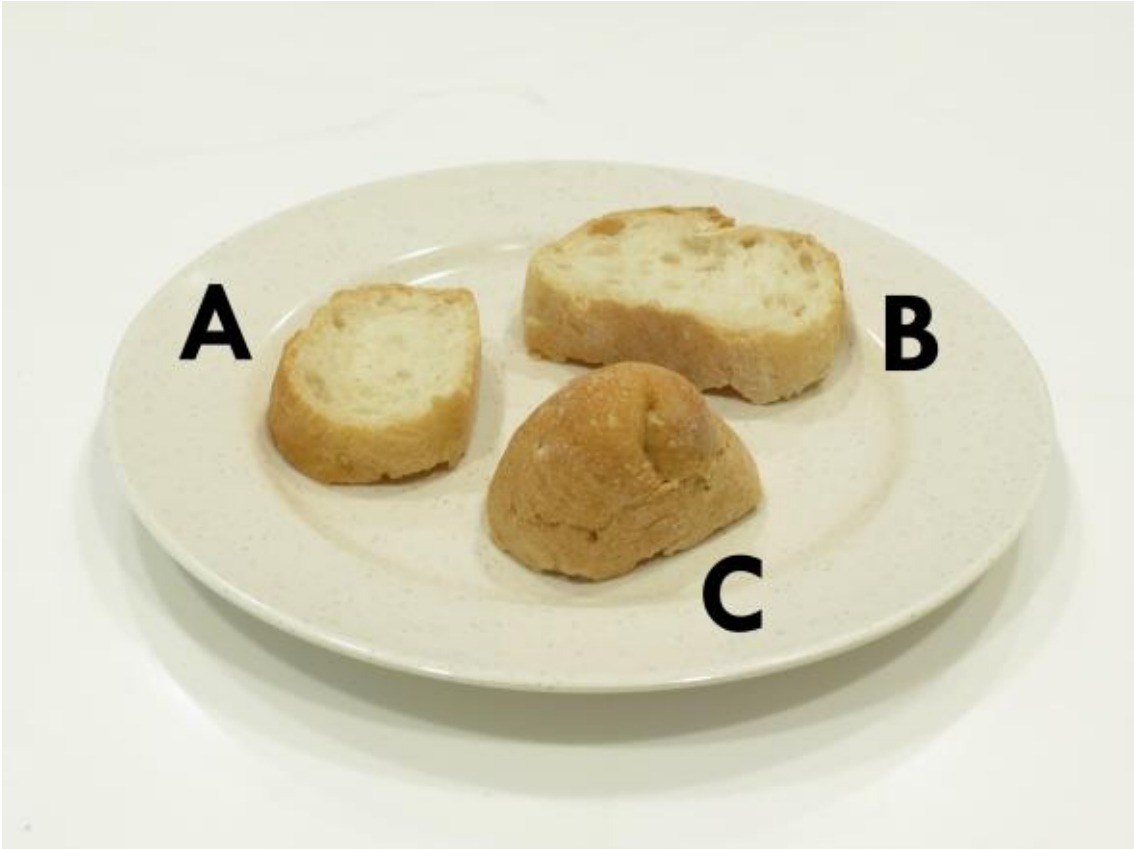

Bao, assorted (FA0039)

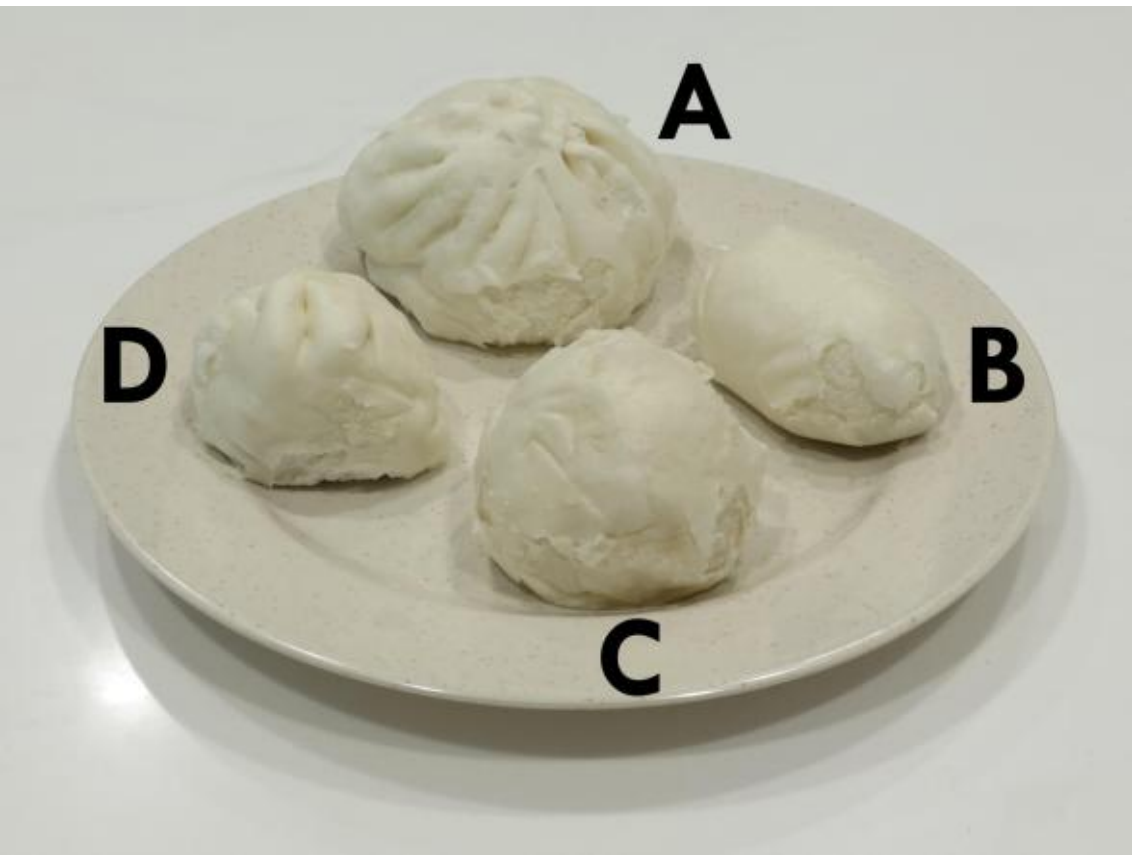

Biscuit, assorted (FA0200)

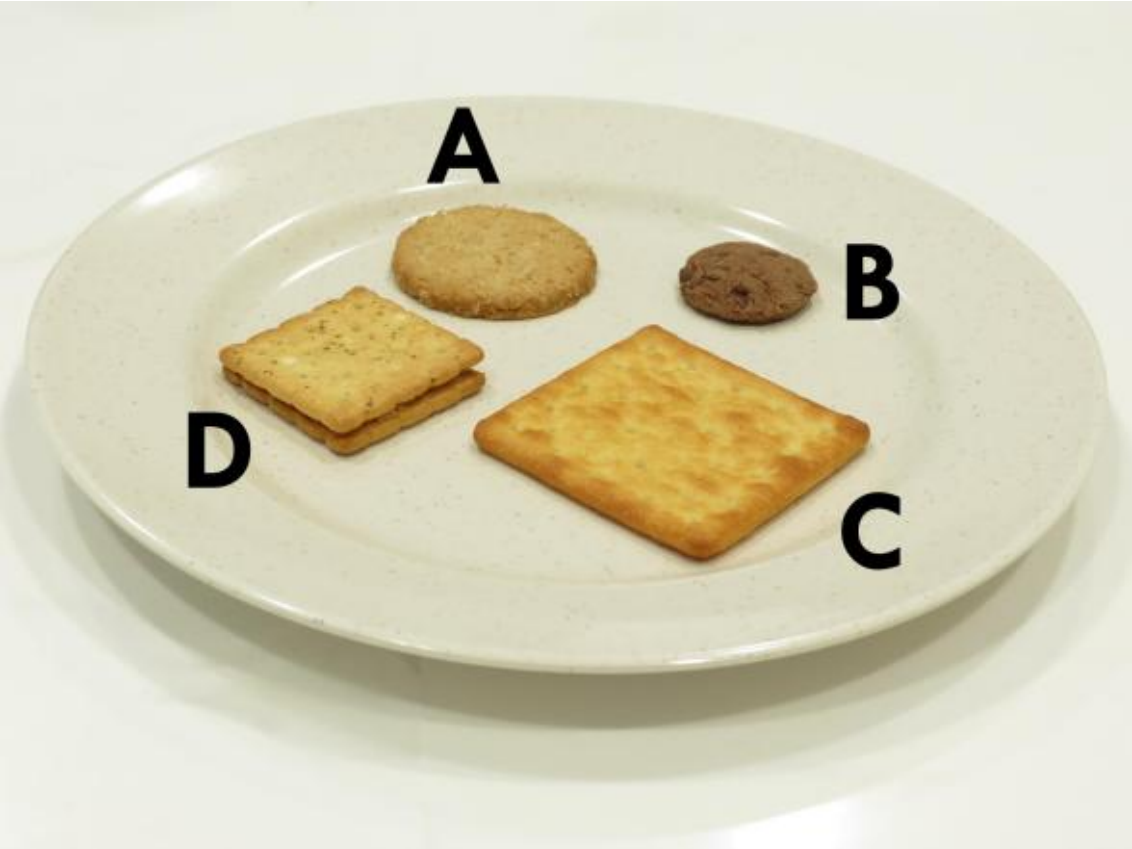

Biscuit, filled, small (FA0145)

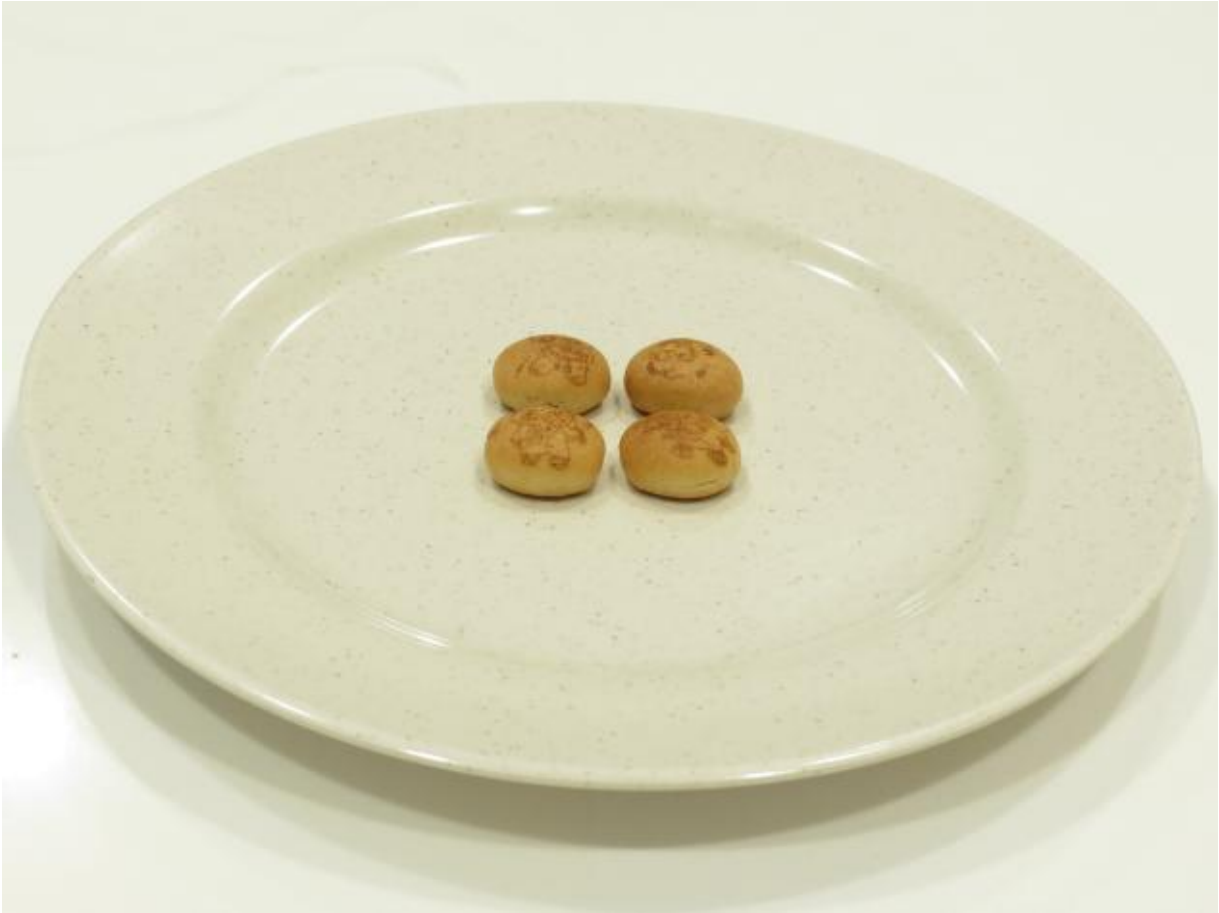

Biscuit, iced gem (FA0144)

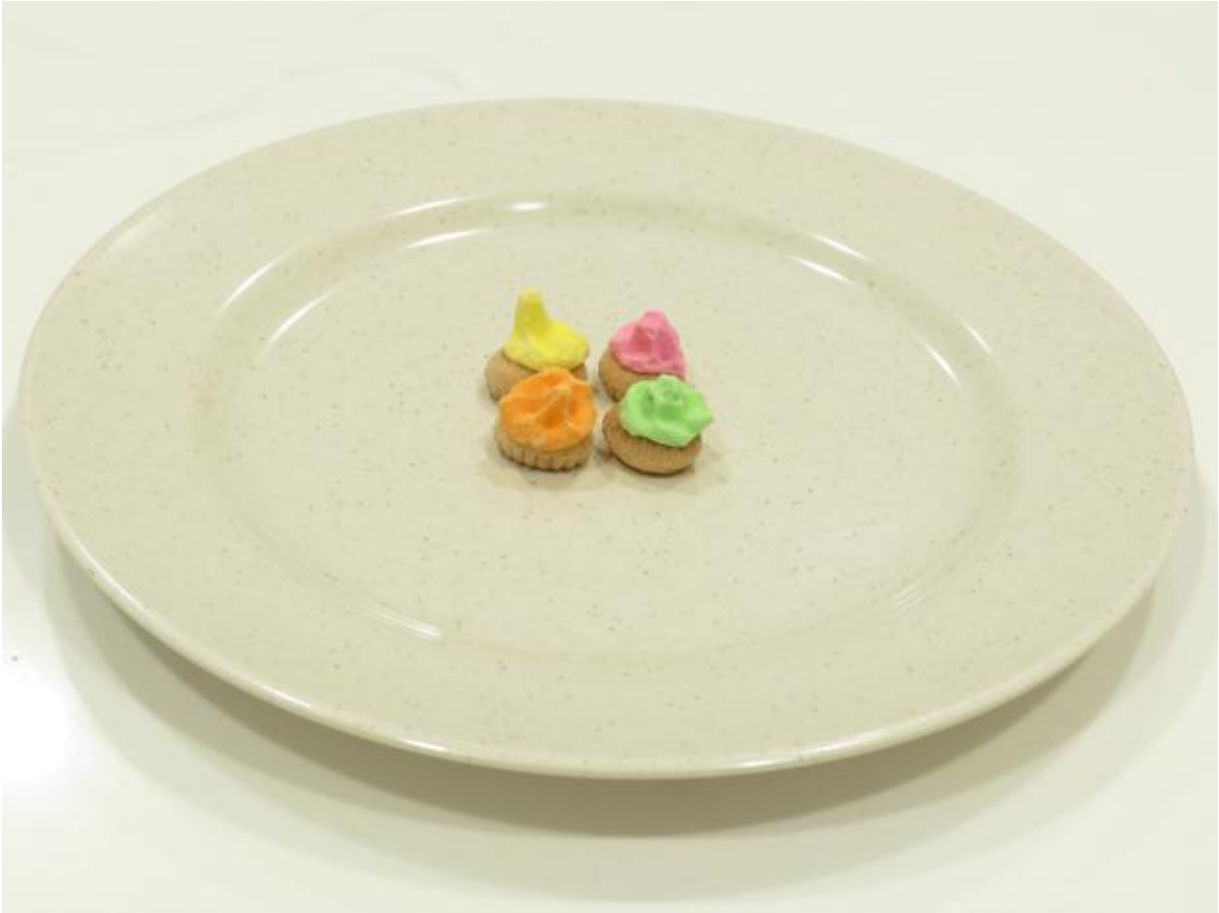

Biscuit, square (FA0172)

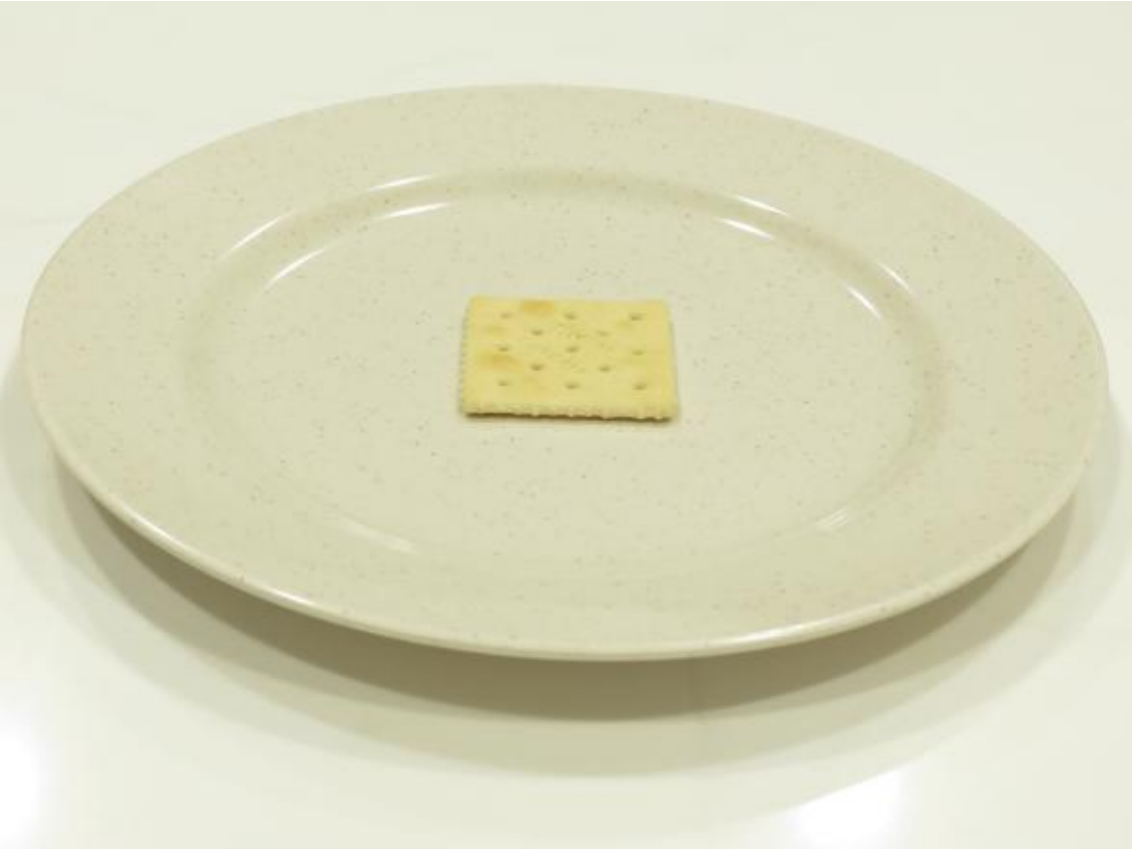

Biscuit, wafer (FA0143)

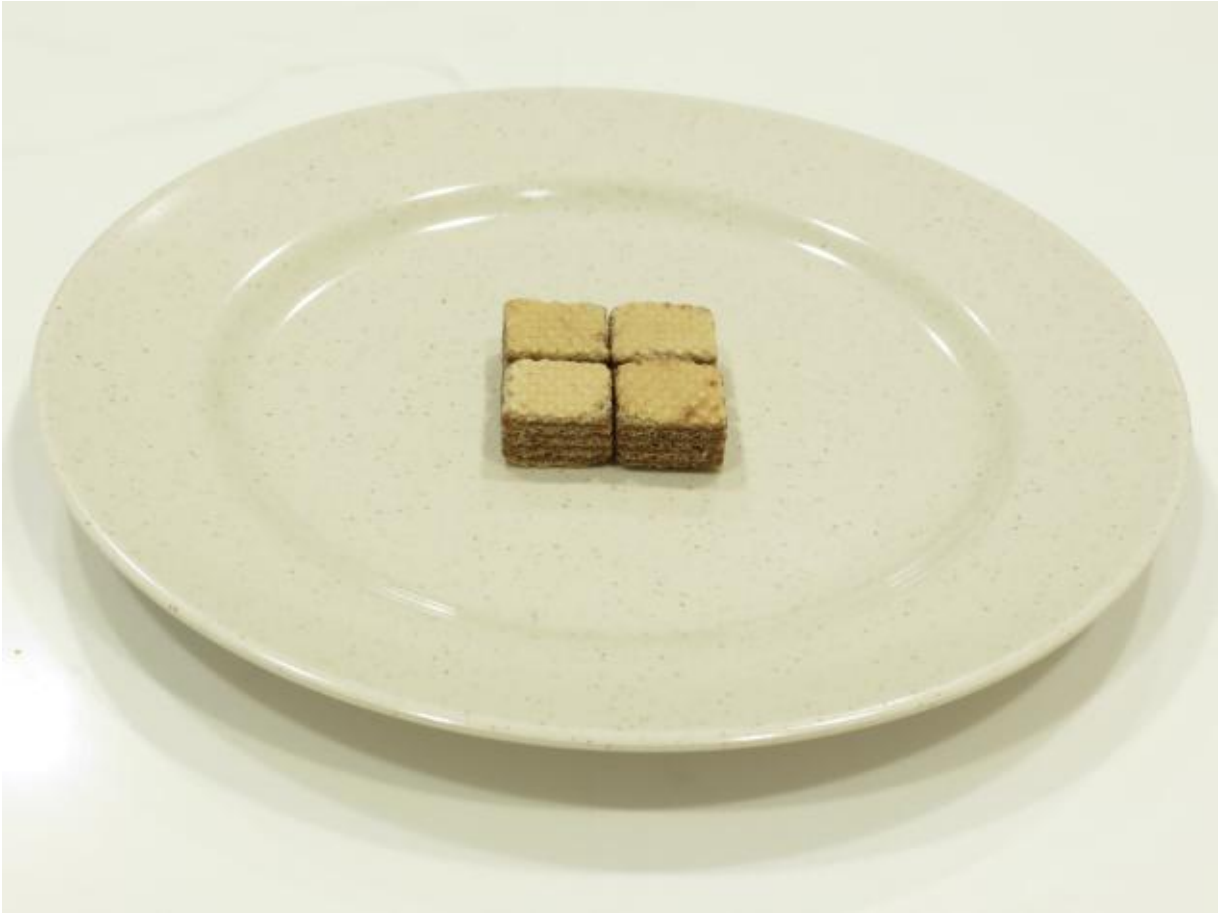

Bread, brioche (FA0085)

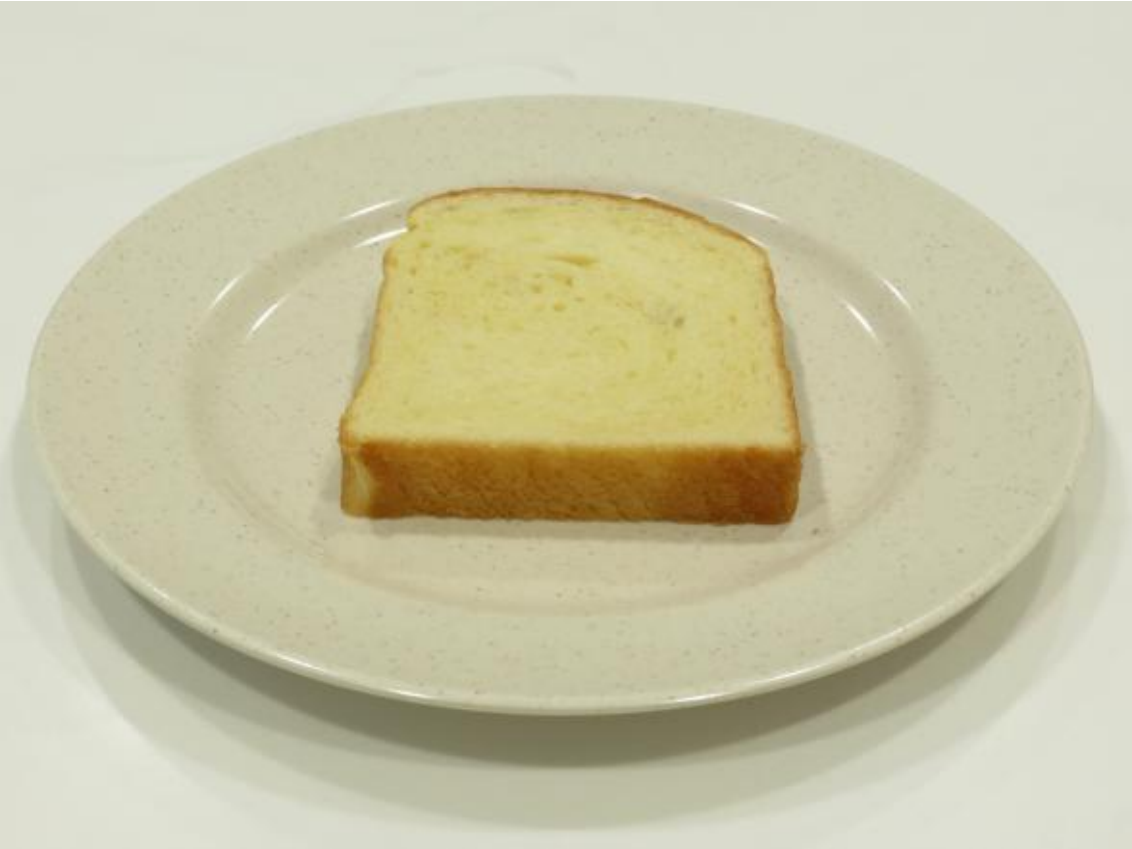

Bread, pita (FA0006)

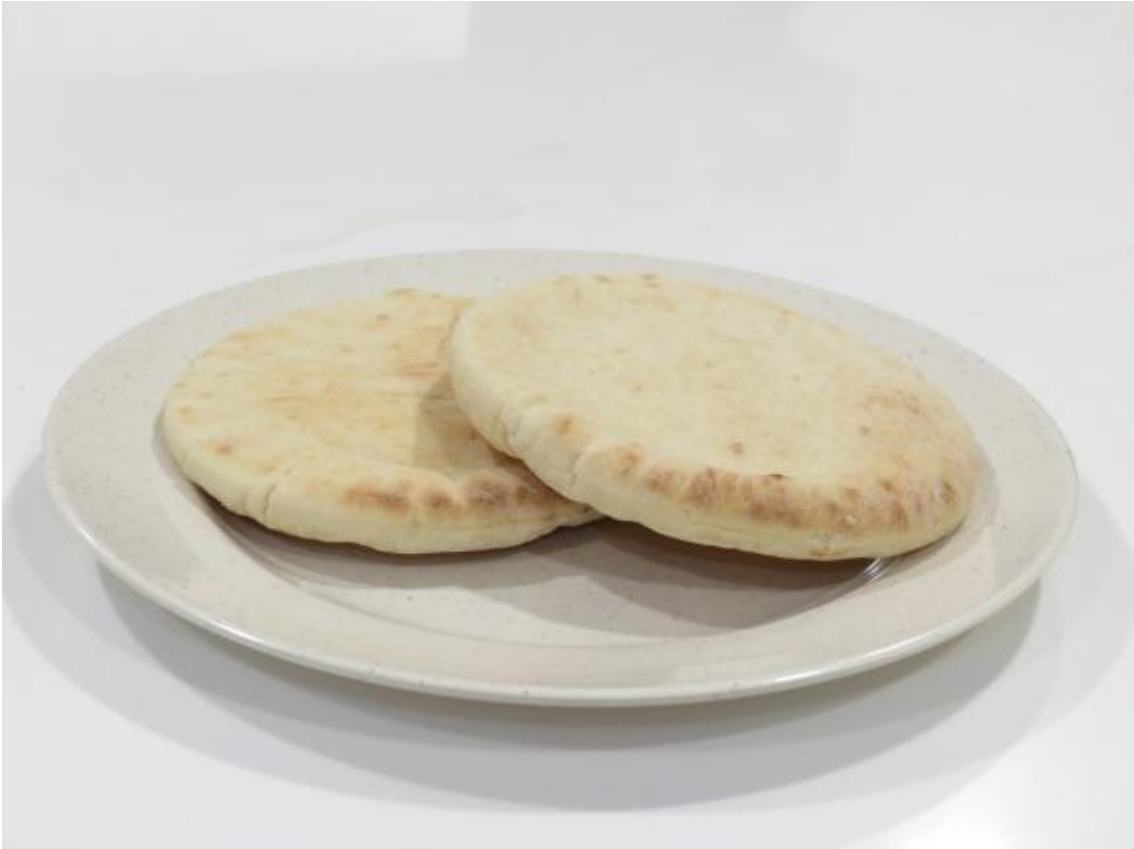

Bread, roll, long (FA0086)

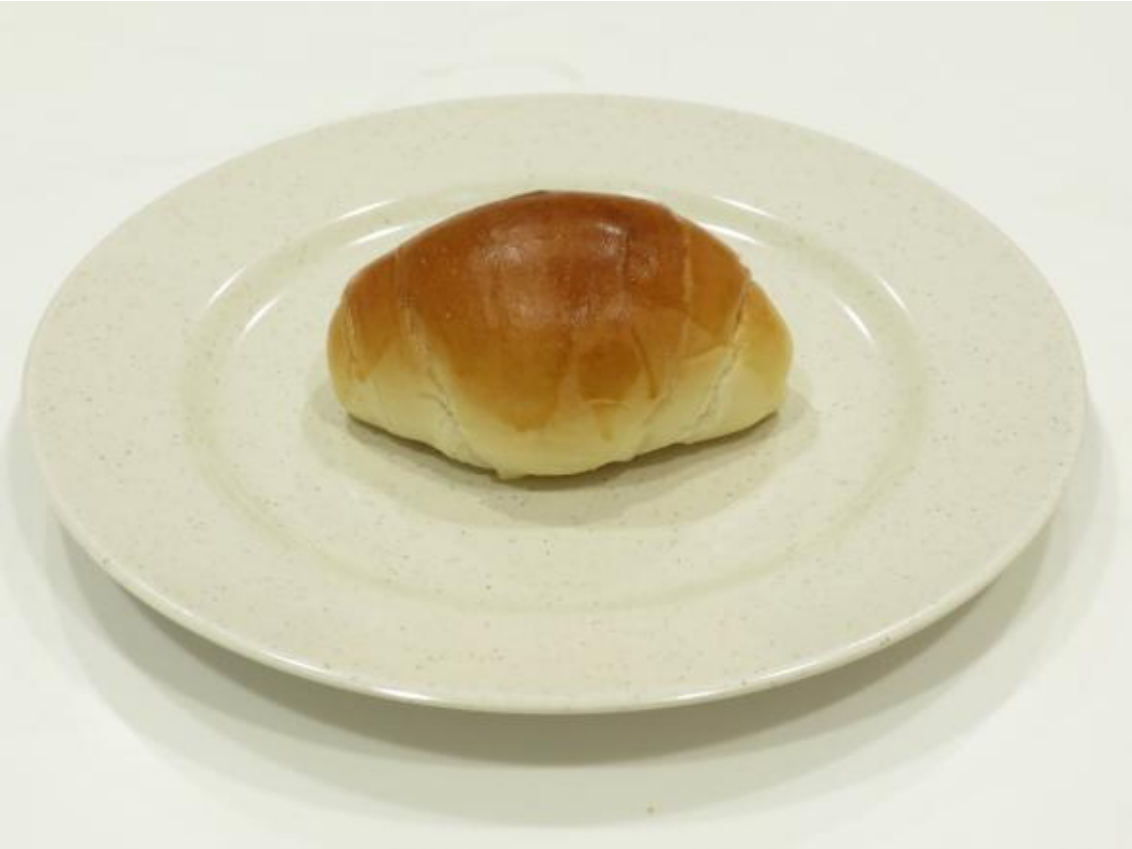

Bread, roll, long, multigrain (FA0136)

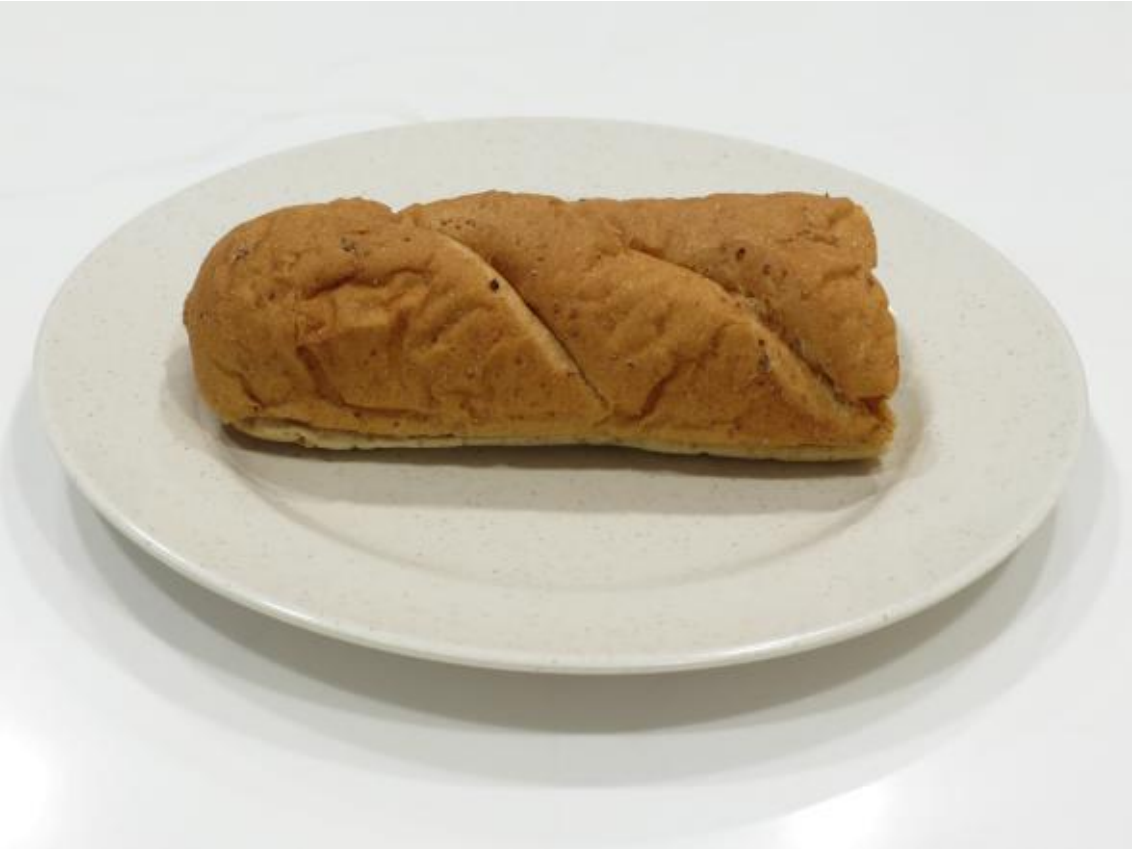

Bread, roll, round (FA0087)

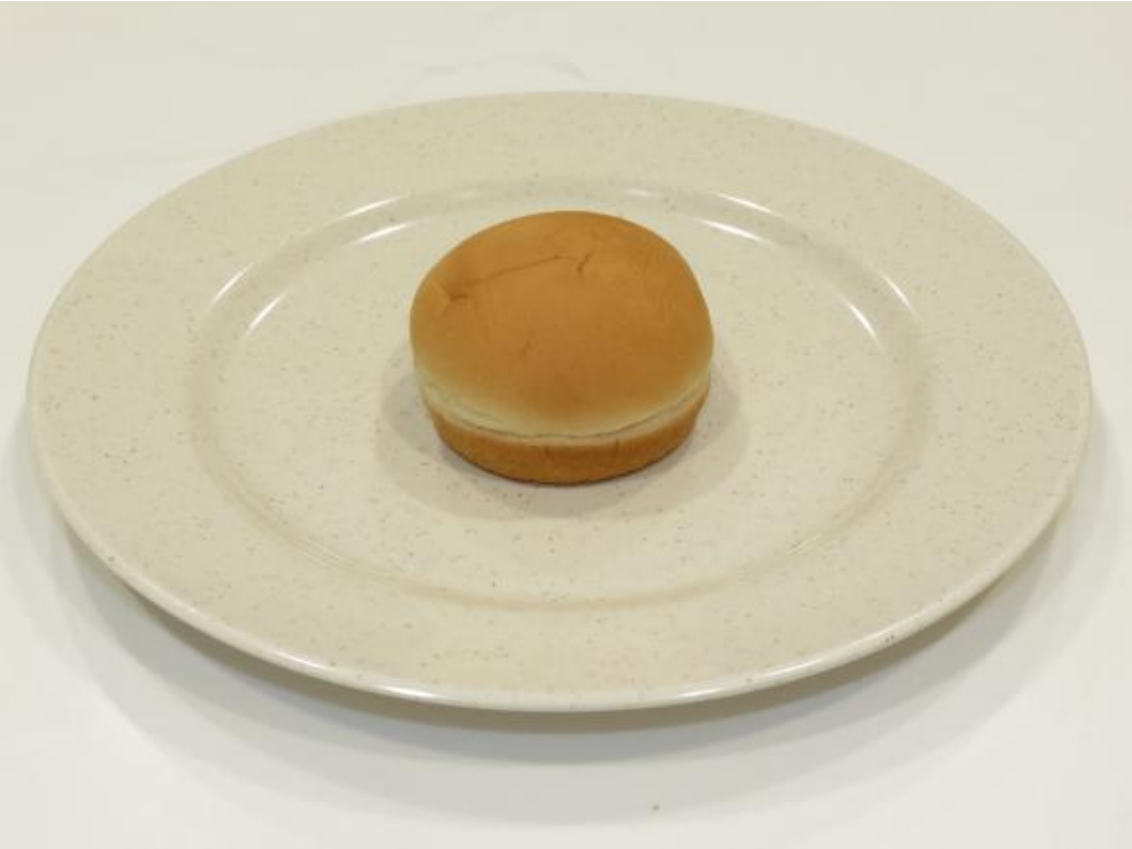

Cake, assorted (FA0035)

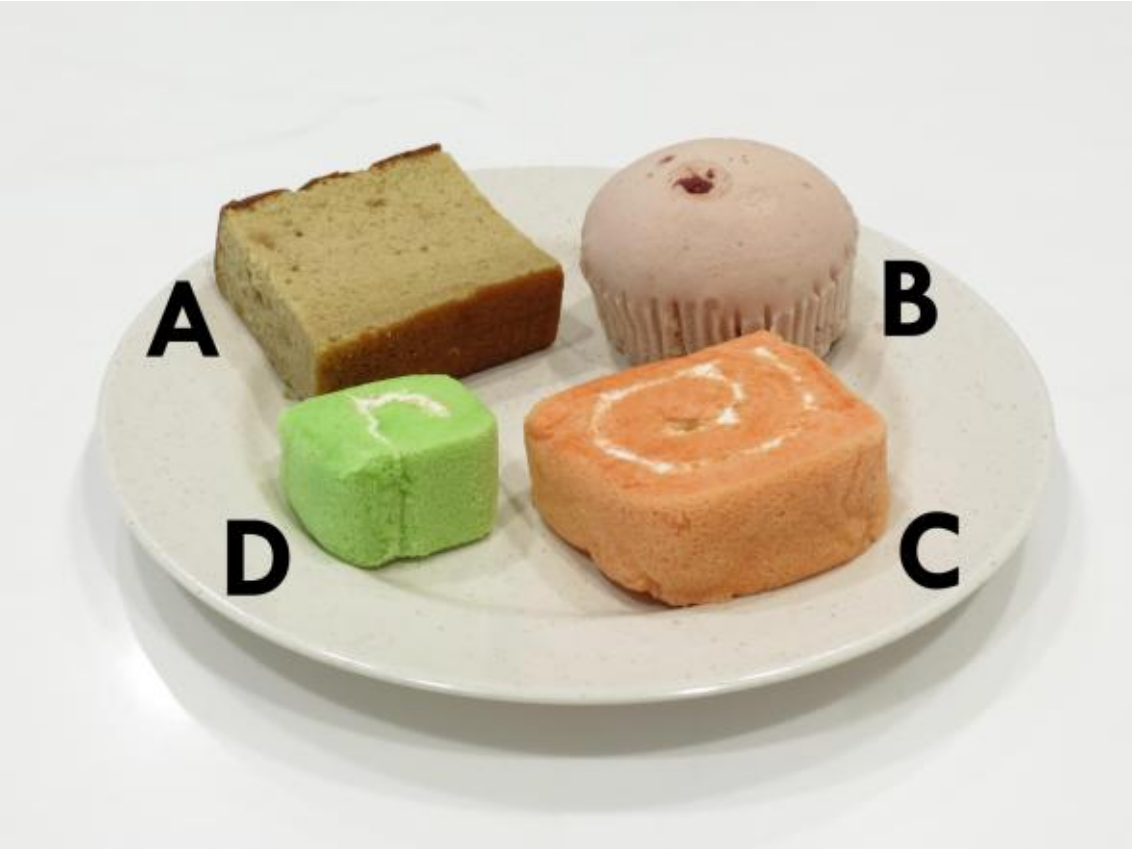

Cake, chiffon (FA0003)

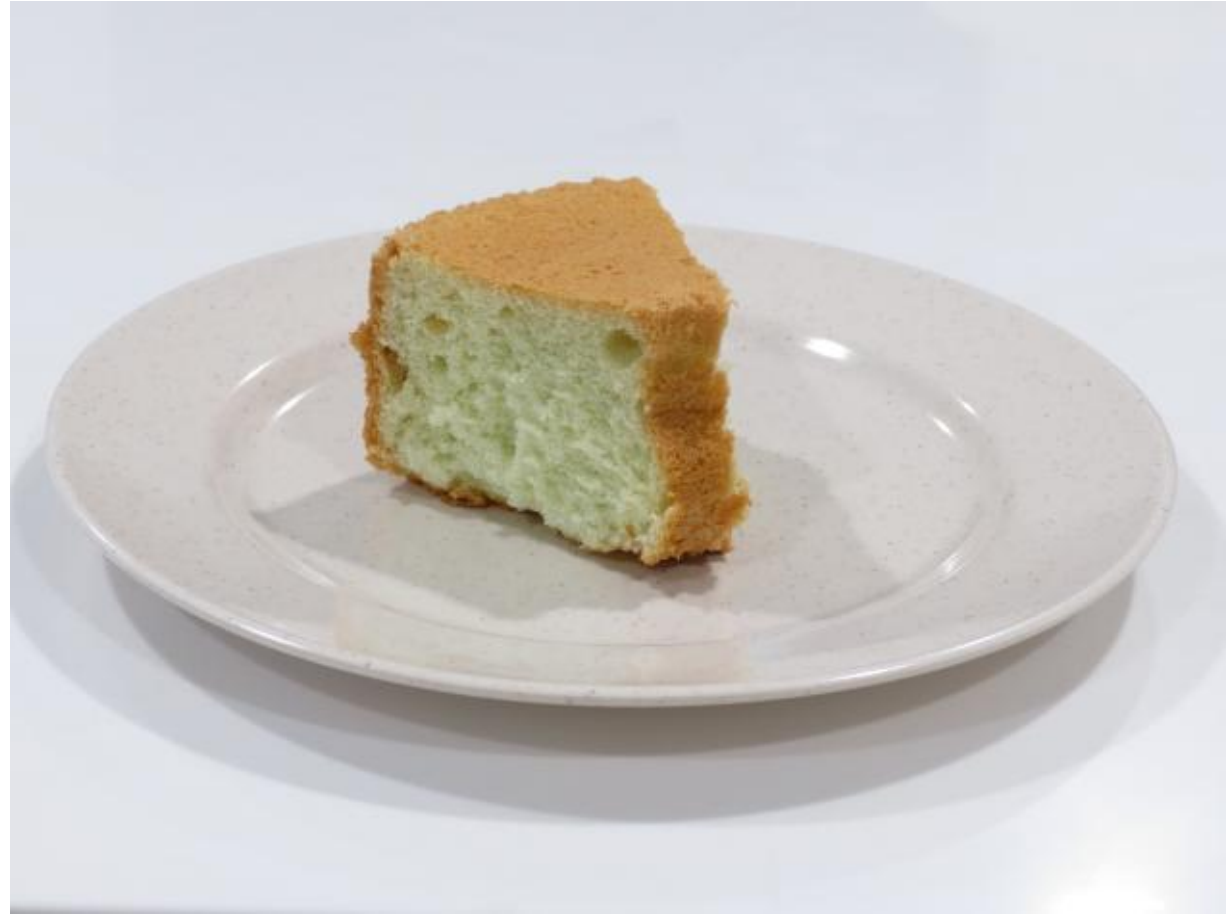

Cake, chocolate (FA0037)

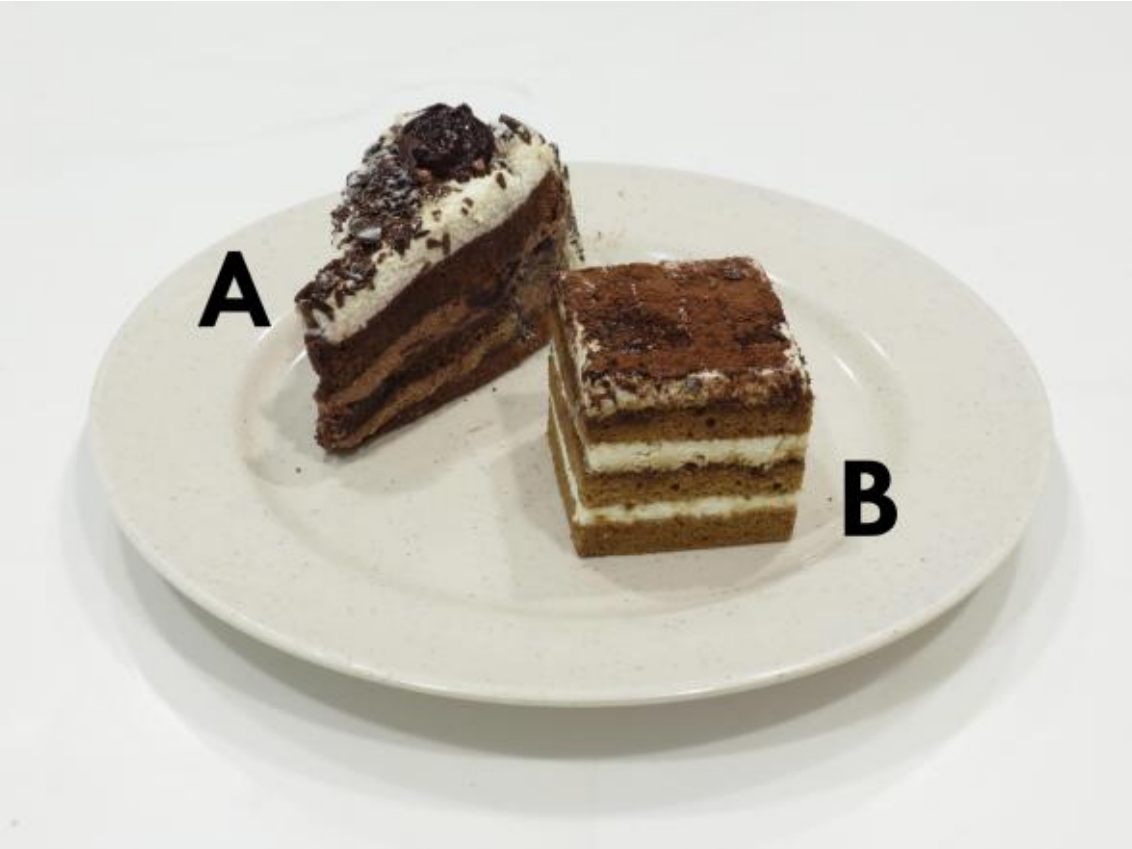

Cake, cream (FA0036)

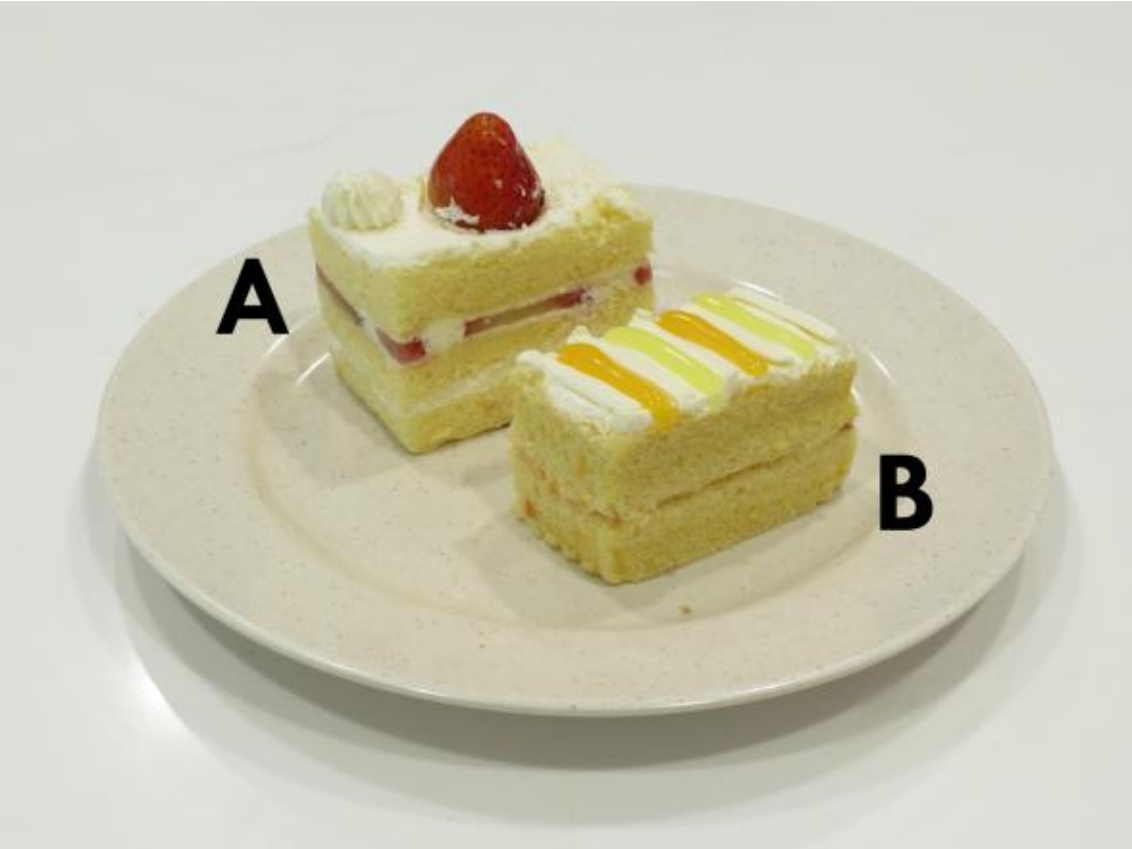

Chwee kueh (水粿) (FA0032)

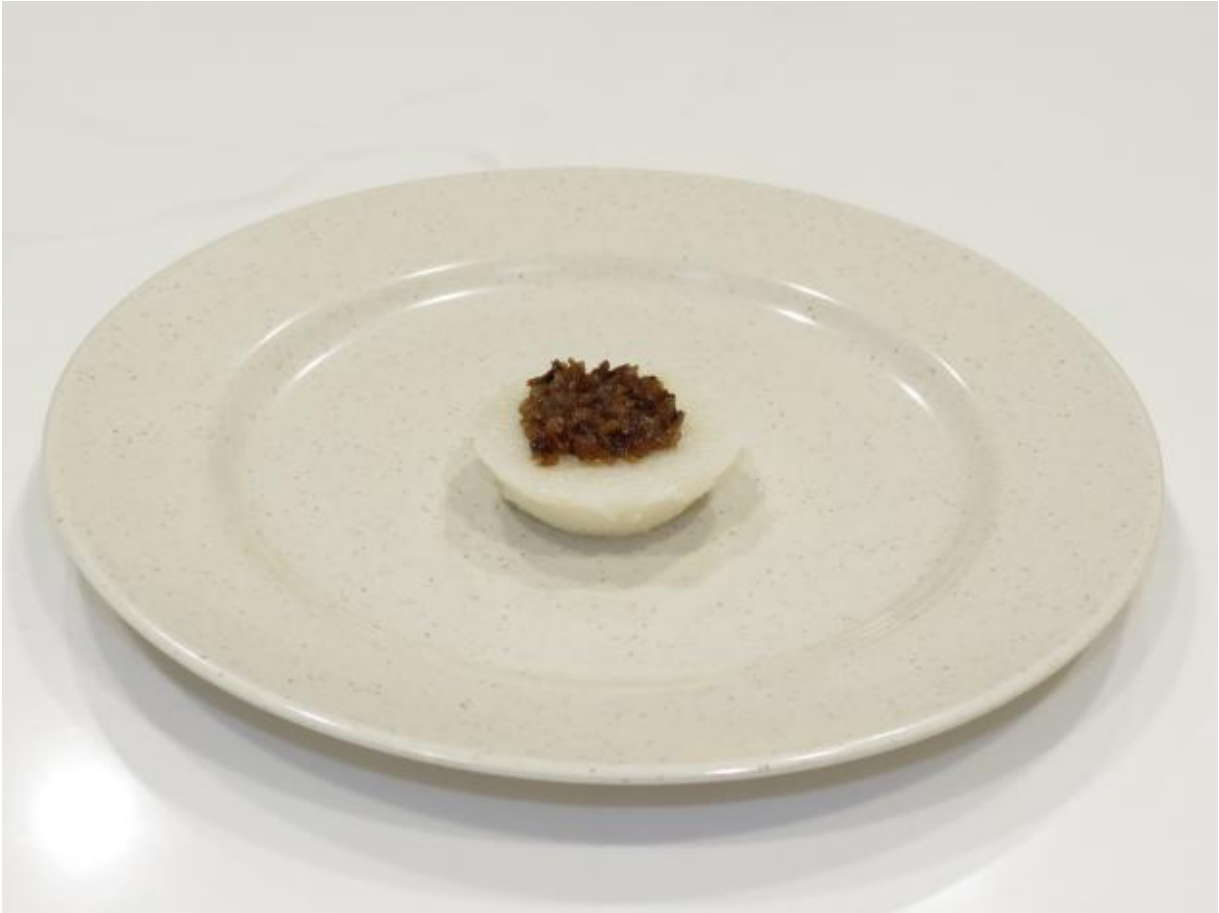

Egg tart (FA0027)

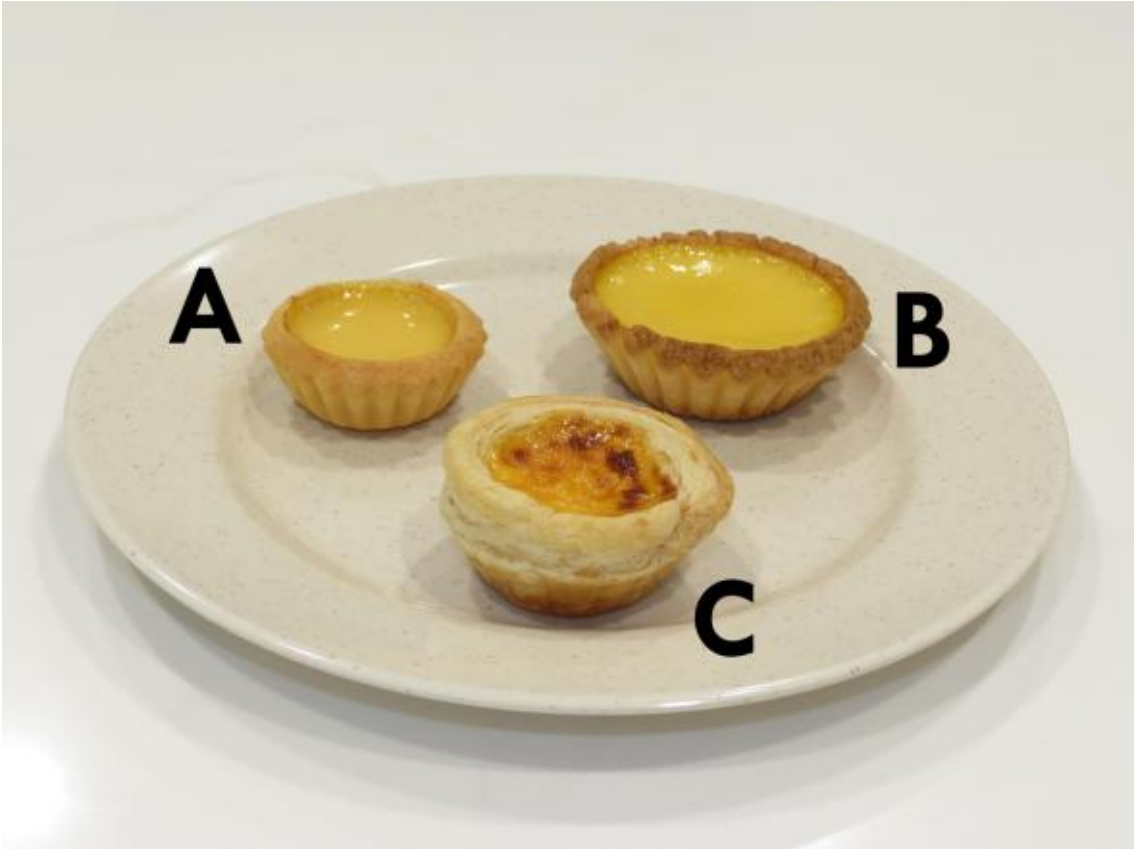

Kueh, assorted (FA0023)

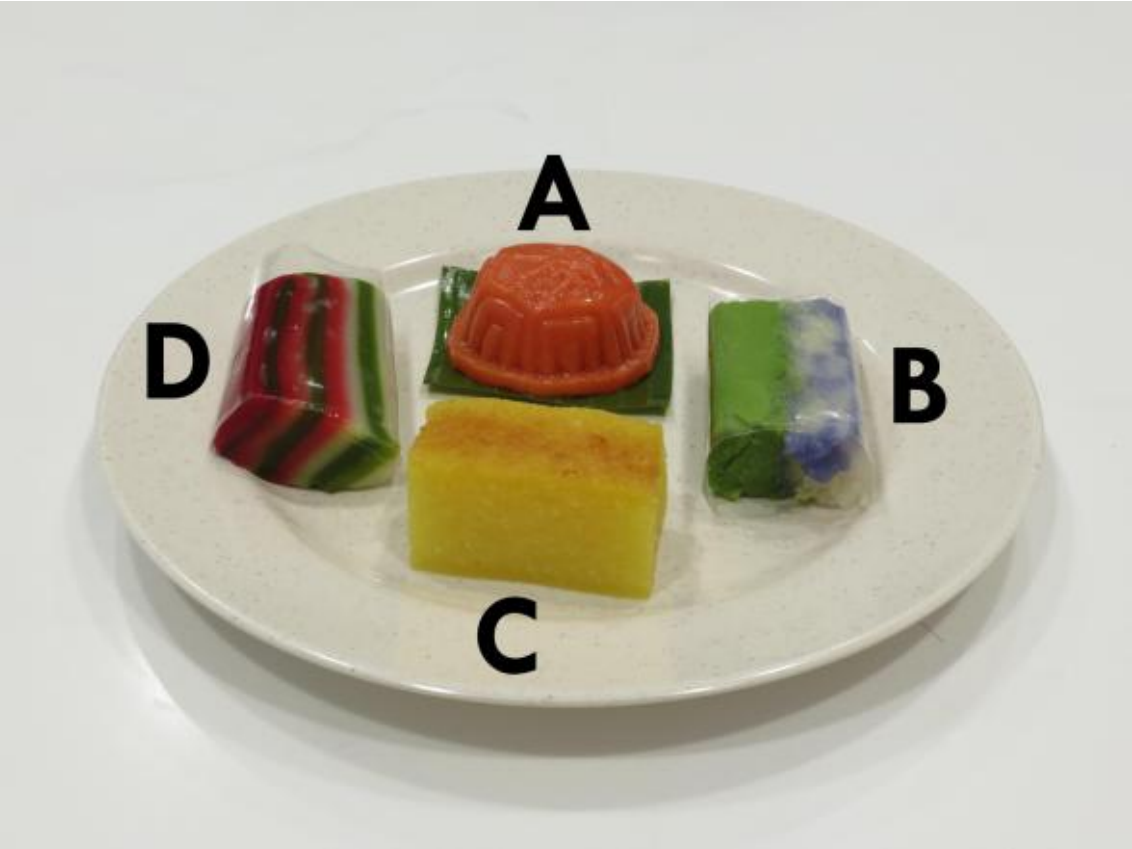

Kueh, kosui (FA0025)

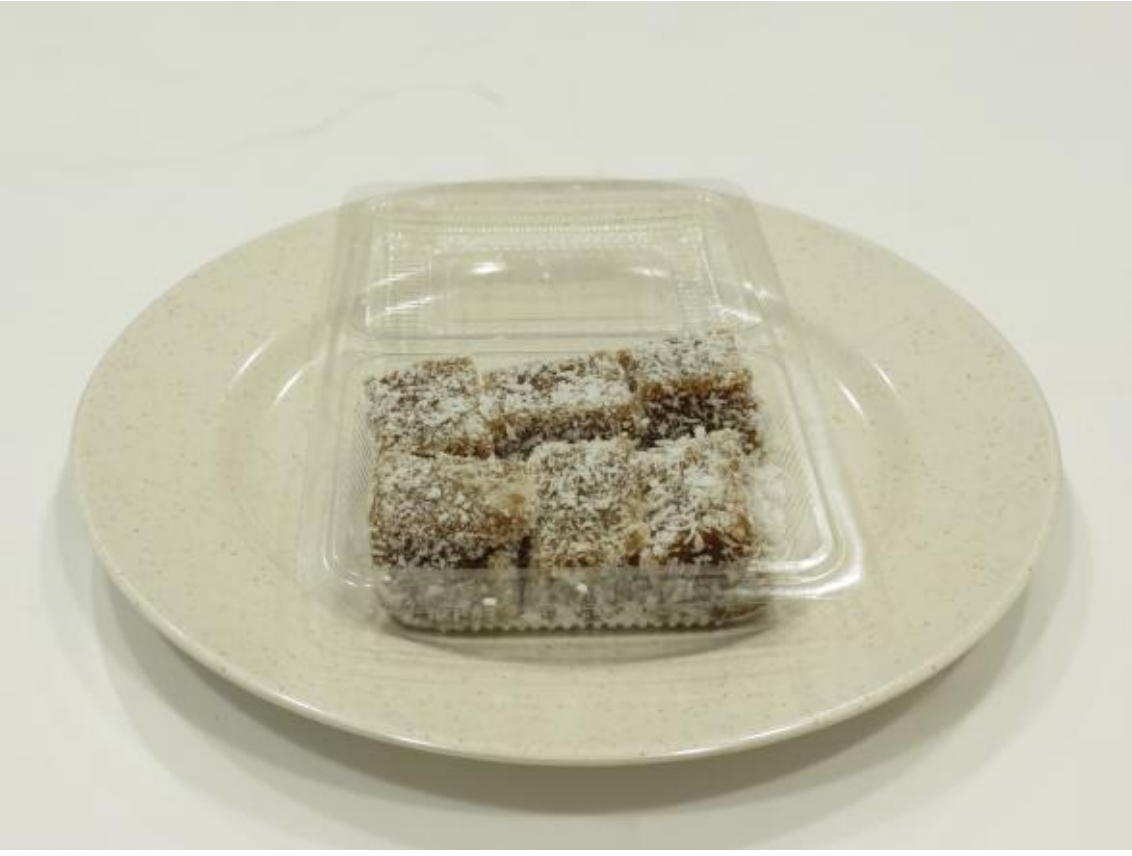

Kueh, onde onde (FA0024)

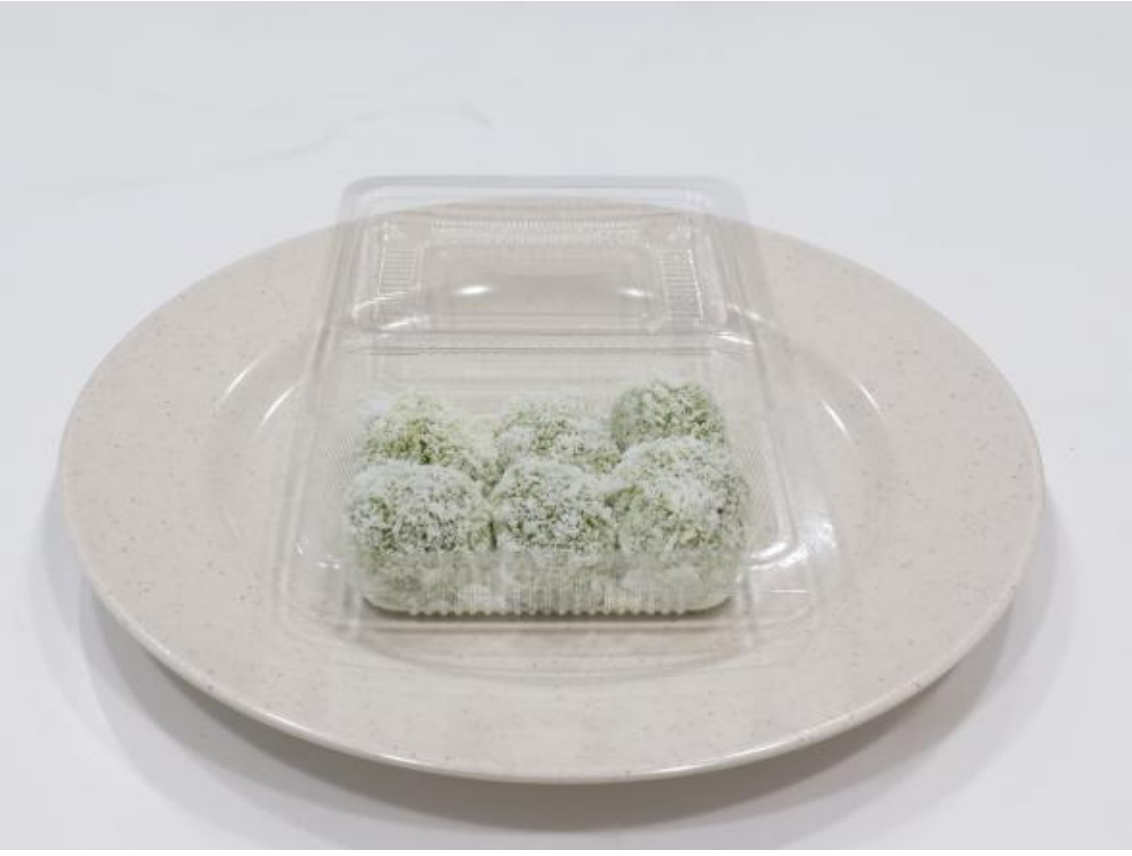

Mooncake, mini, no egg (FA0159)

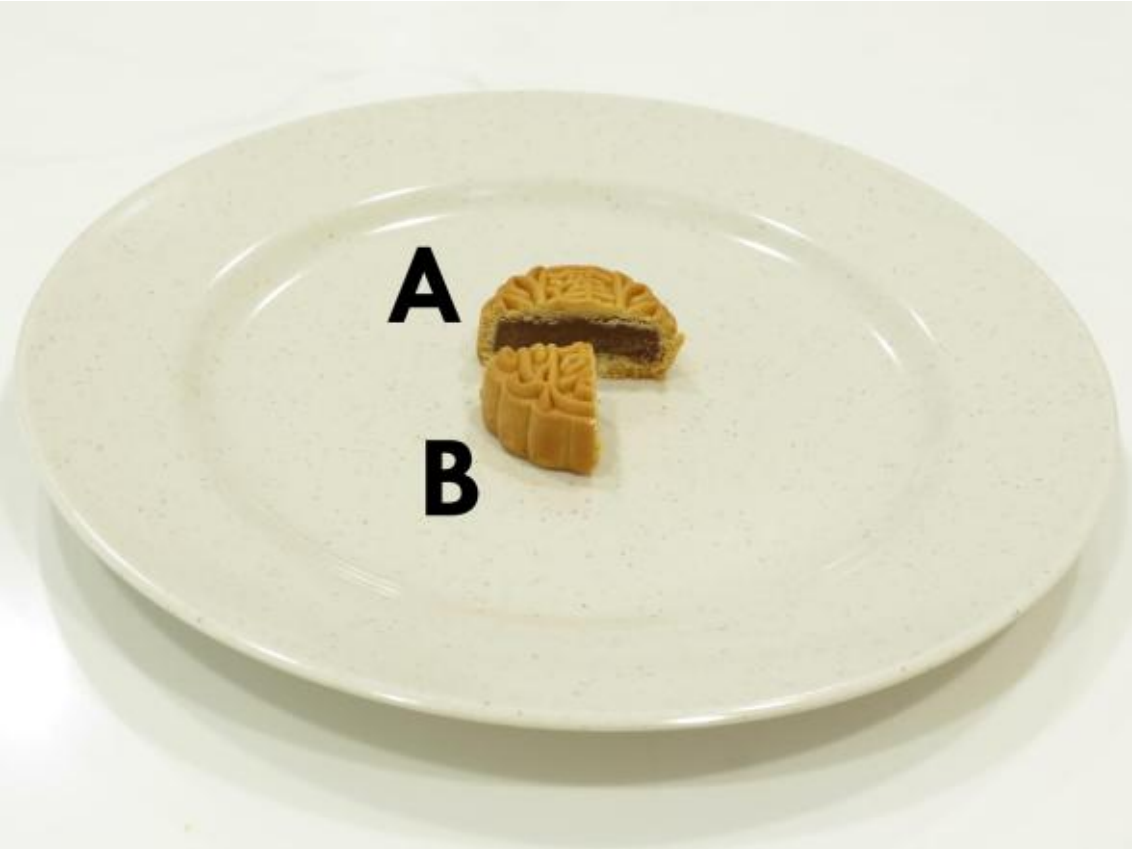

Mooncake, regular, no egg (FA0146)

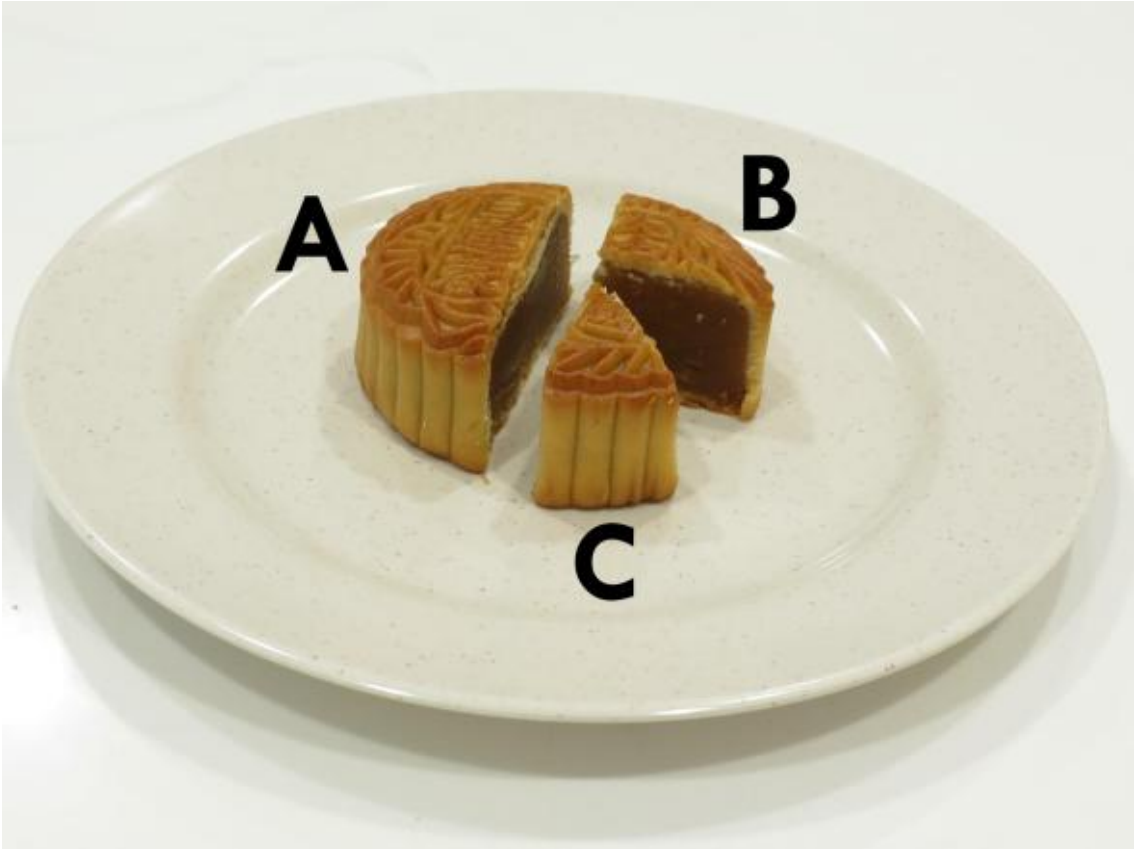

Mooncake, snow skin, no egg (FA0122)

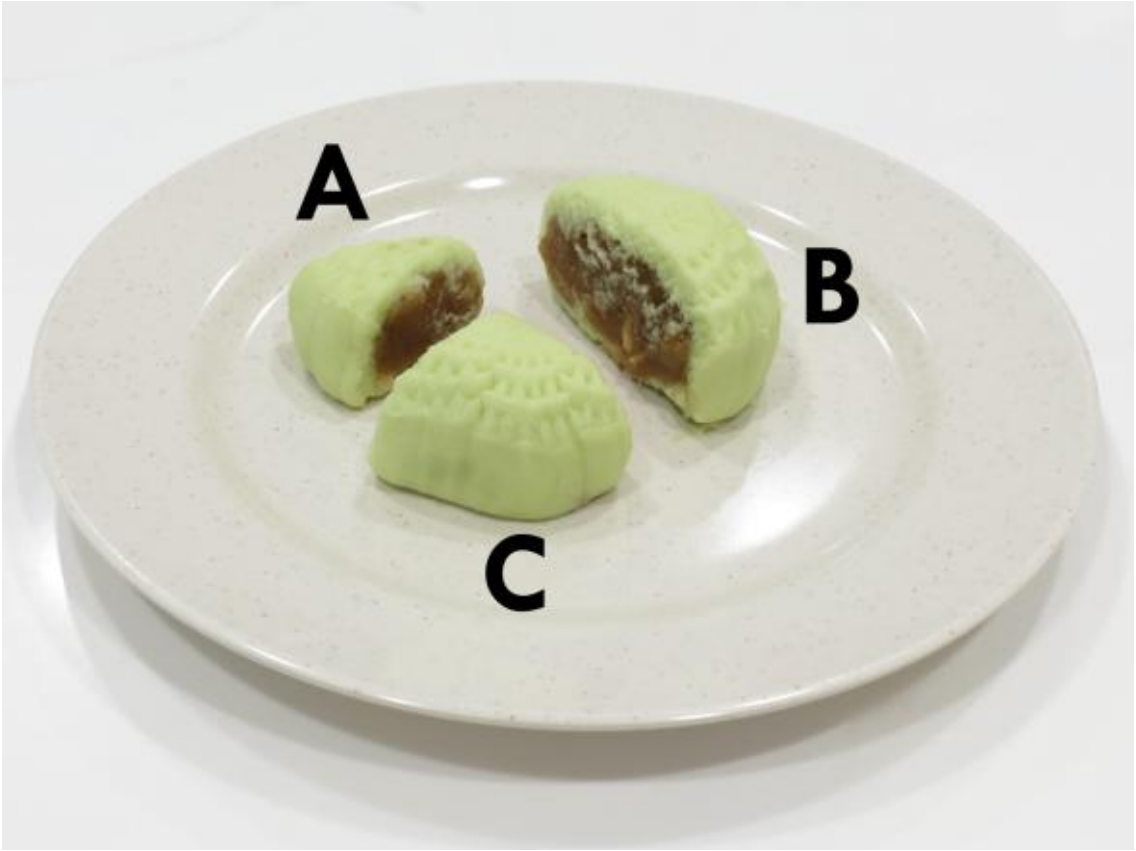

Pancake, regular (FA0043)

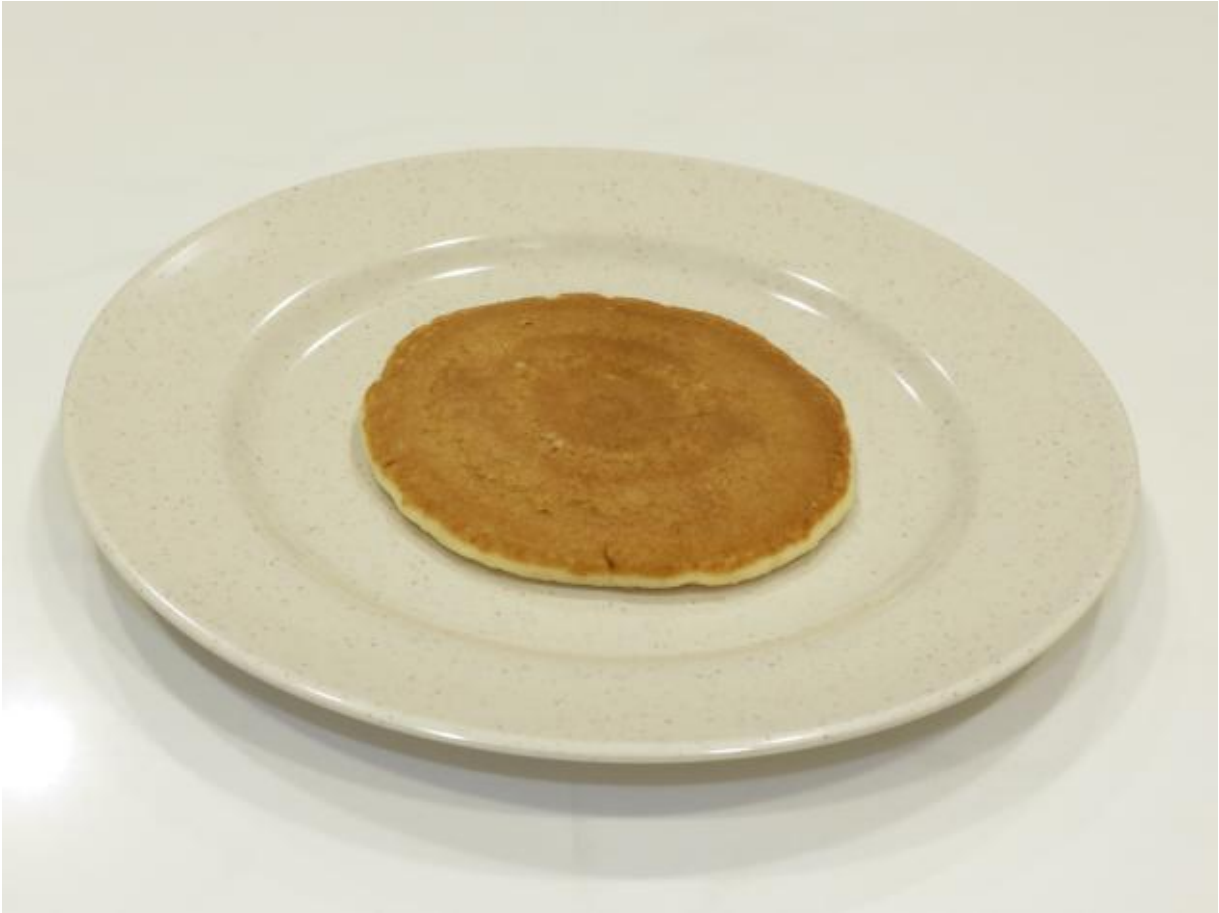

Pretzel, regular (FA0034)

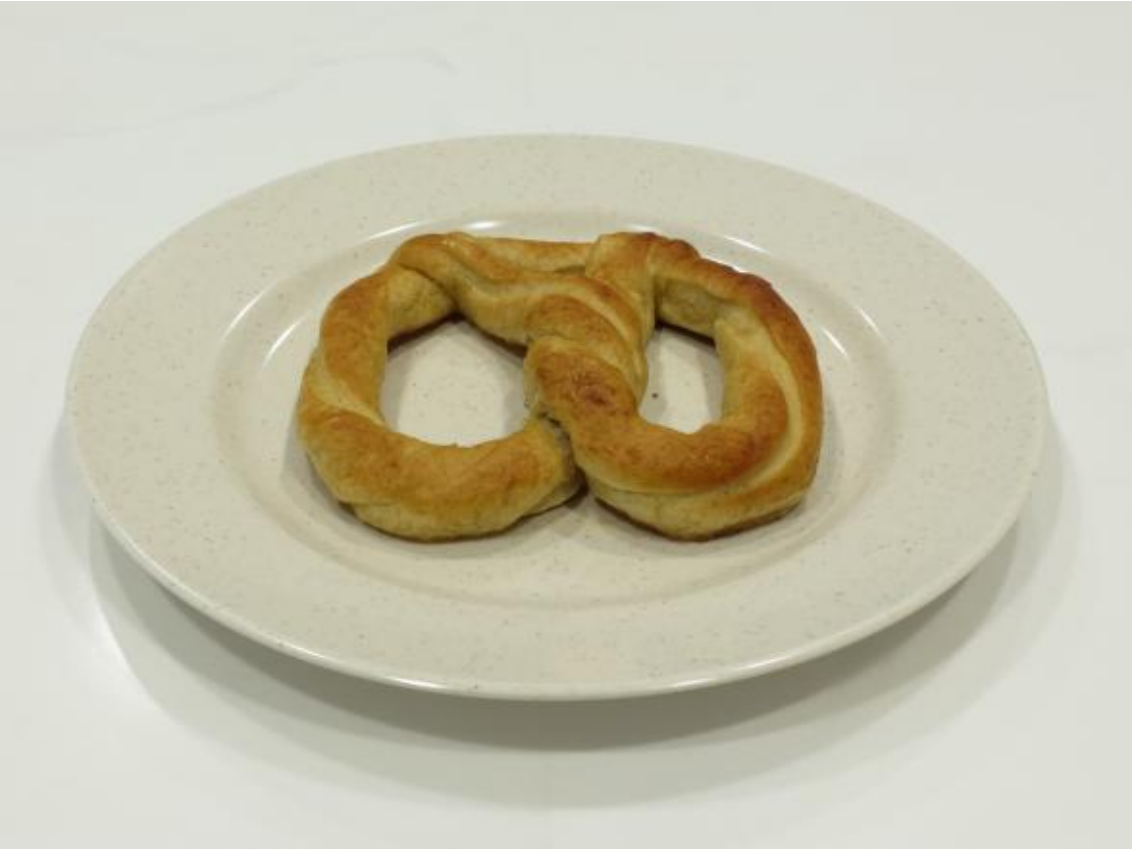

Pretzel, stick (FA0008)

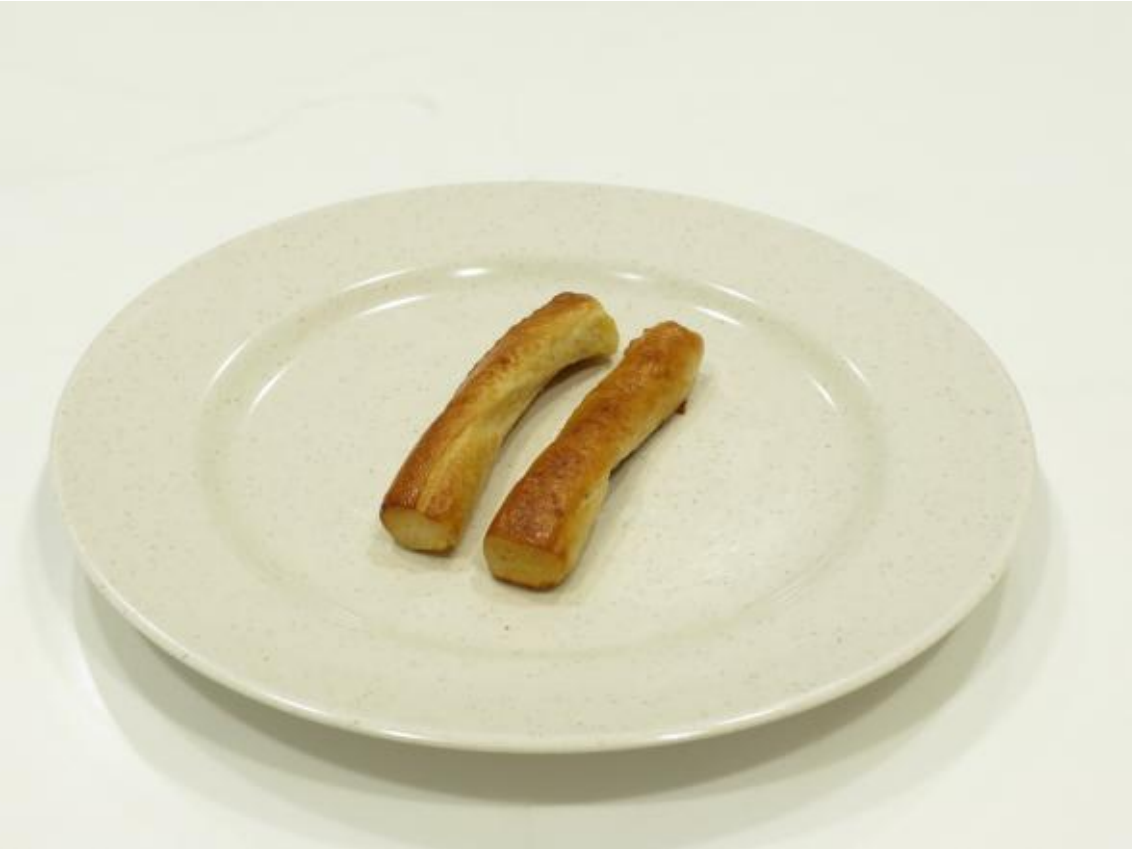

Sesame ball (FA0015)

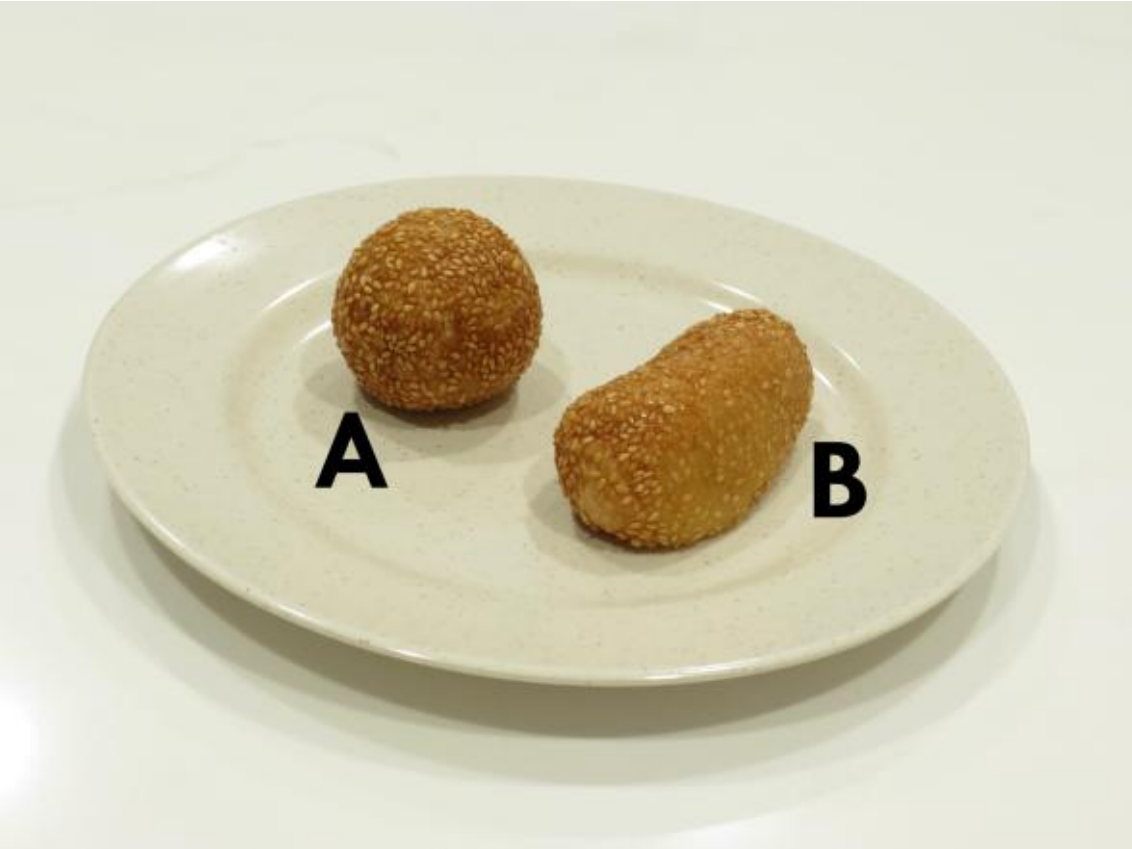

You tiao (油条) (FA0014)

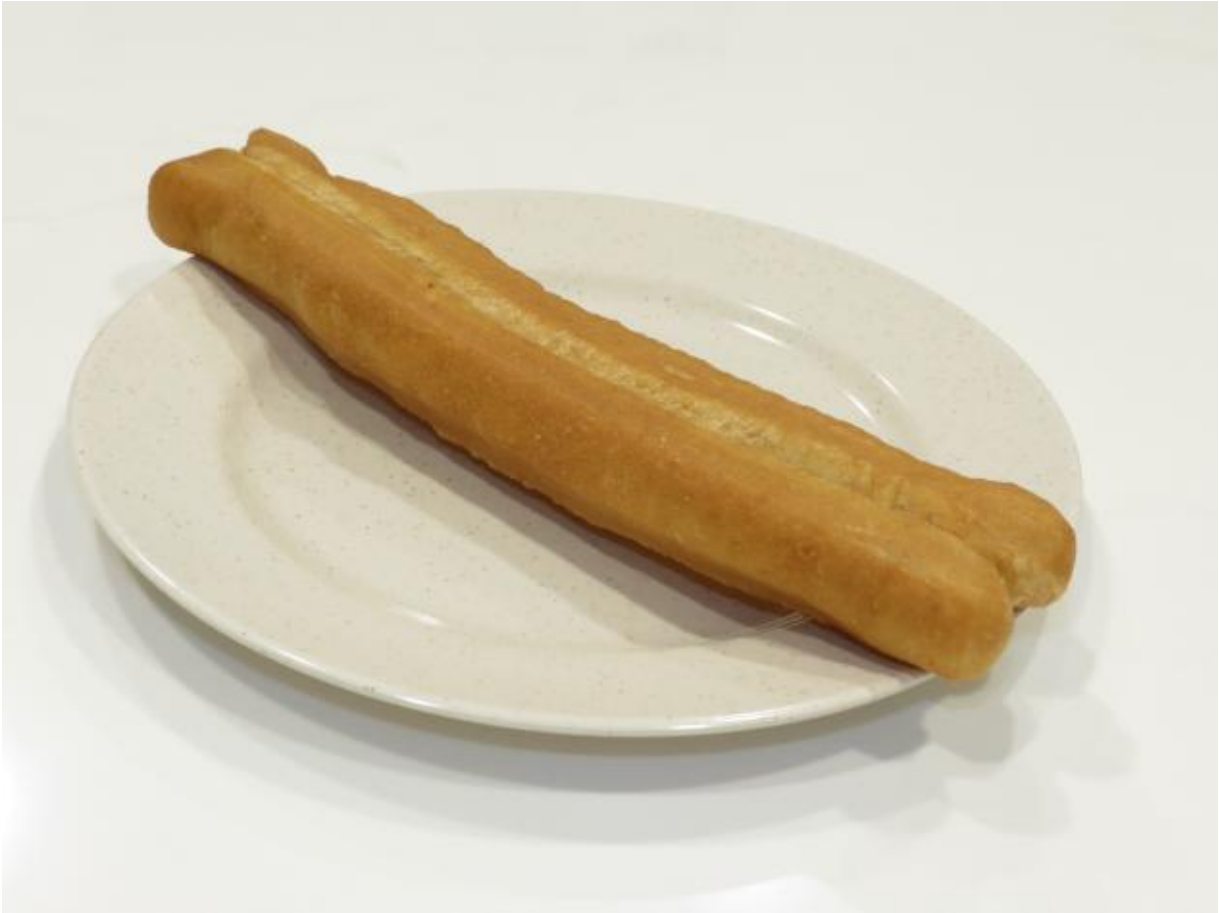

Barley drink (FA0099)

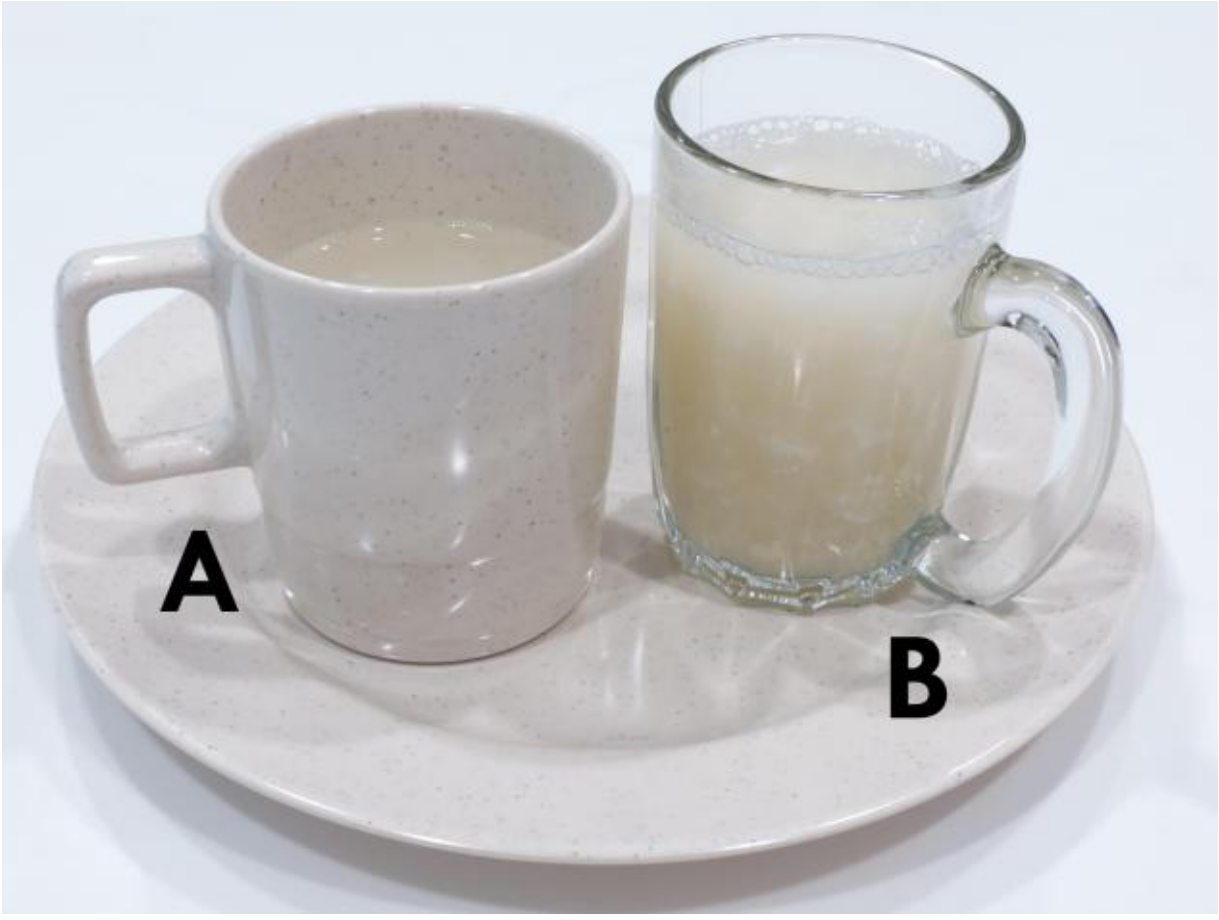

Blended shake (FA0097)

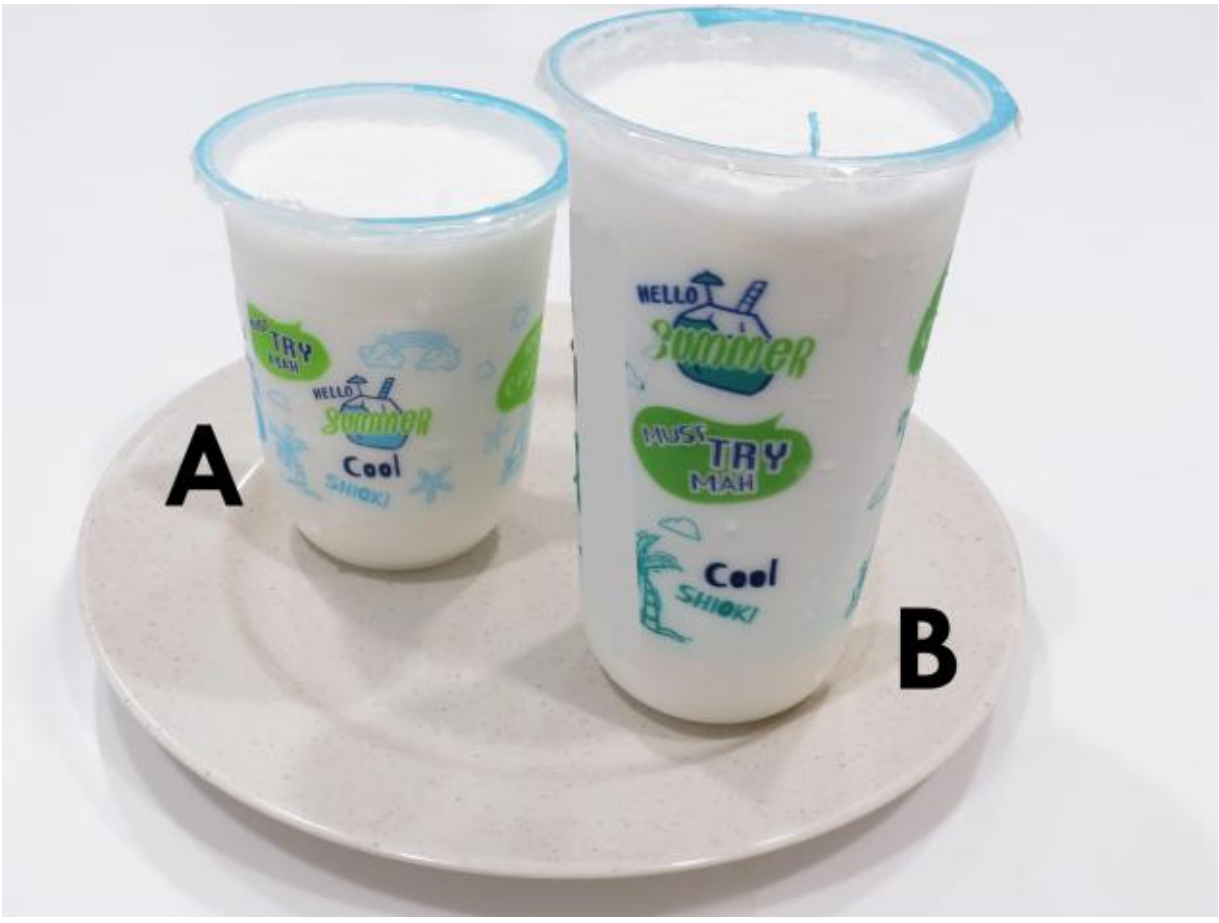

Coffee / Kopi (FA0040)

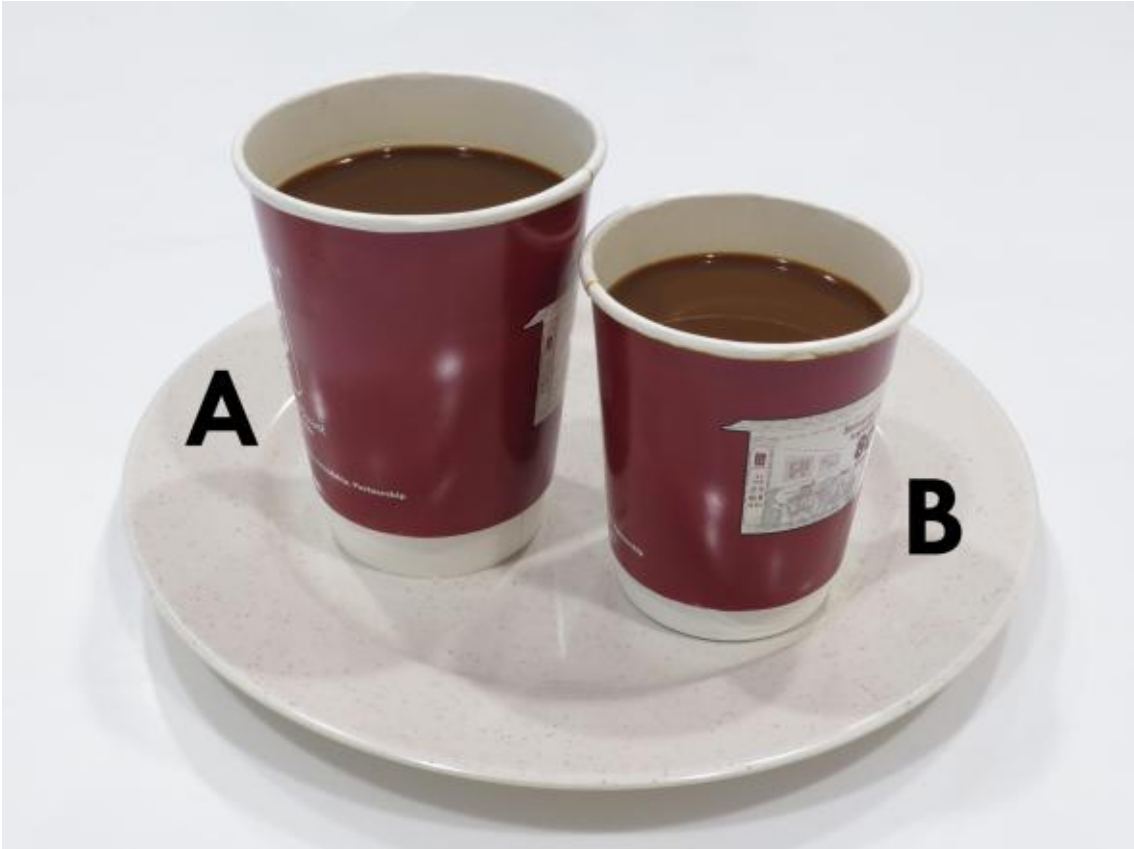

Dim sum, assorted (FA0028)

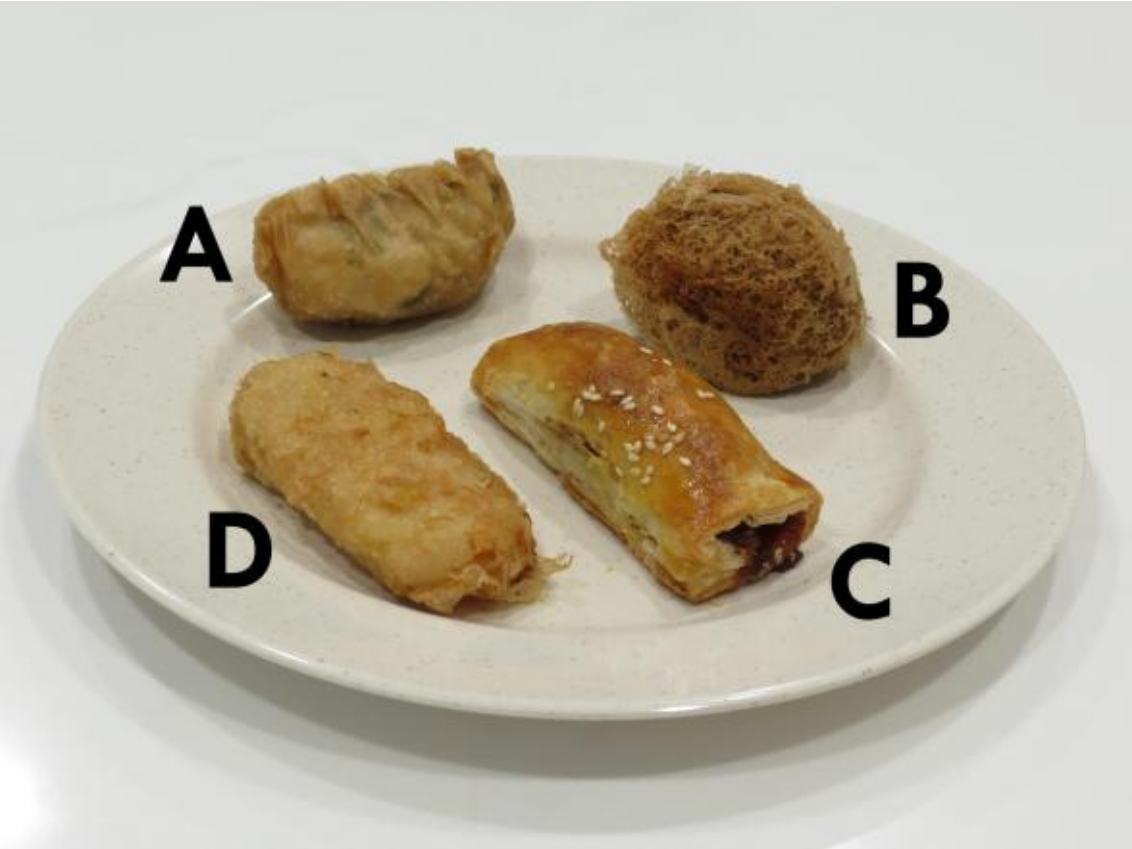

Dim sum, carrot cake (萝卜糕) (FA0030)

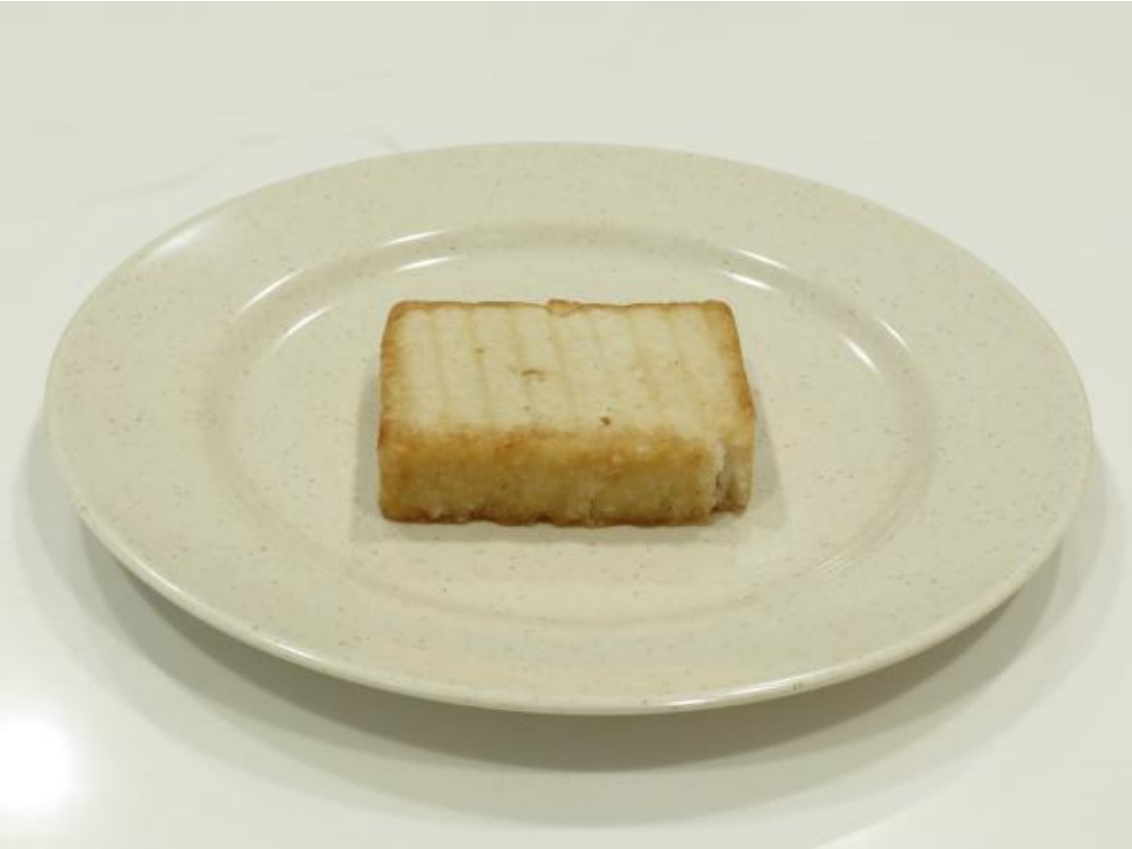

Dim sum, lor mai gai (糯米鸡) (FA0064)

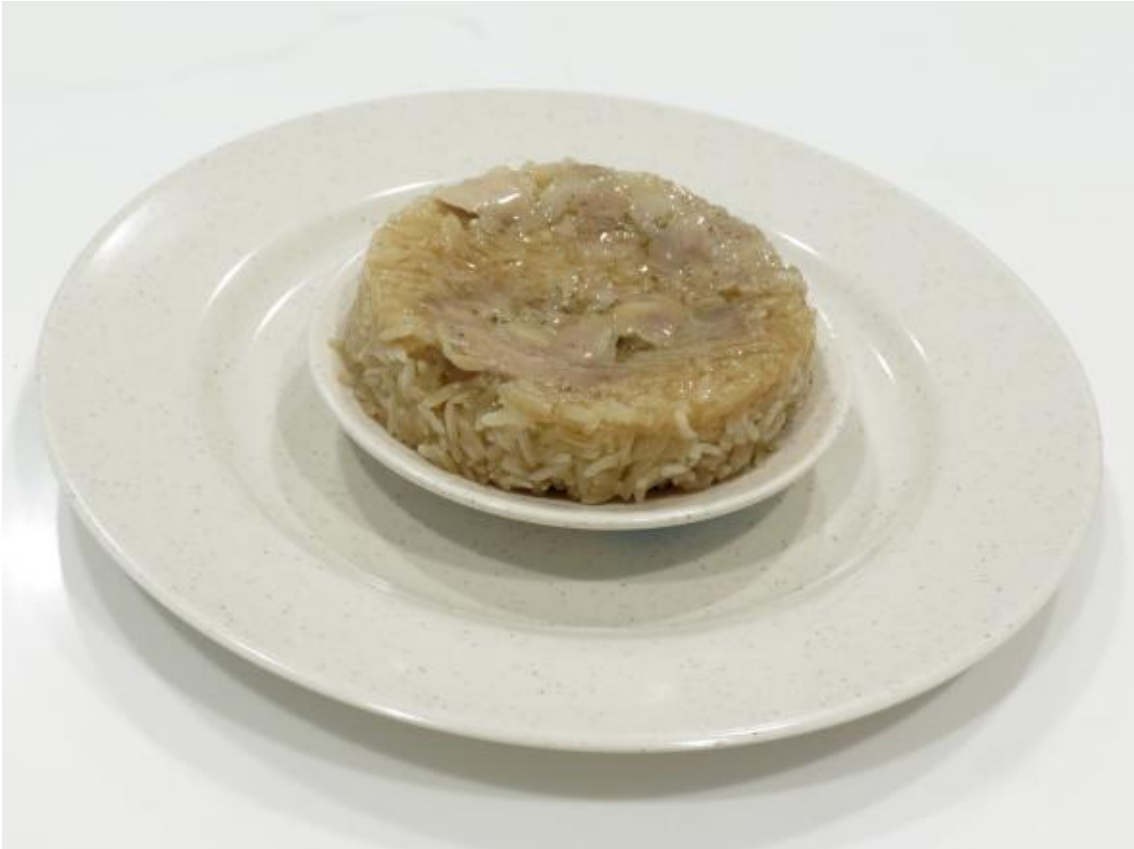

Dim sum, meat roll, ngoh hiang (五香卷) (FA0031)

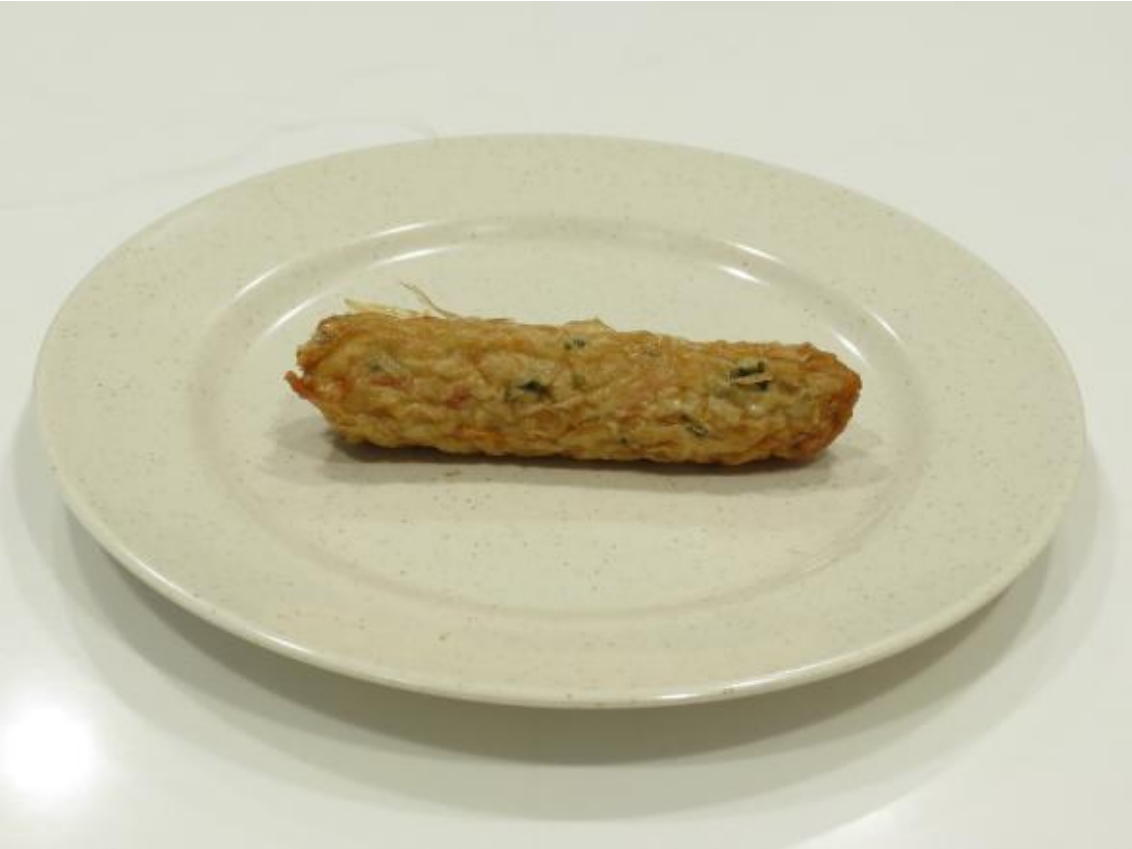

Dim sum, siew mai (烧卖) (FA0072)

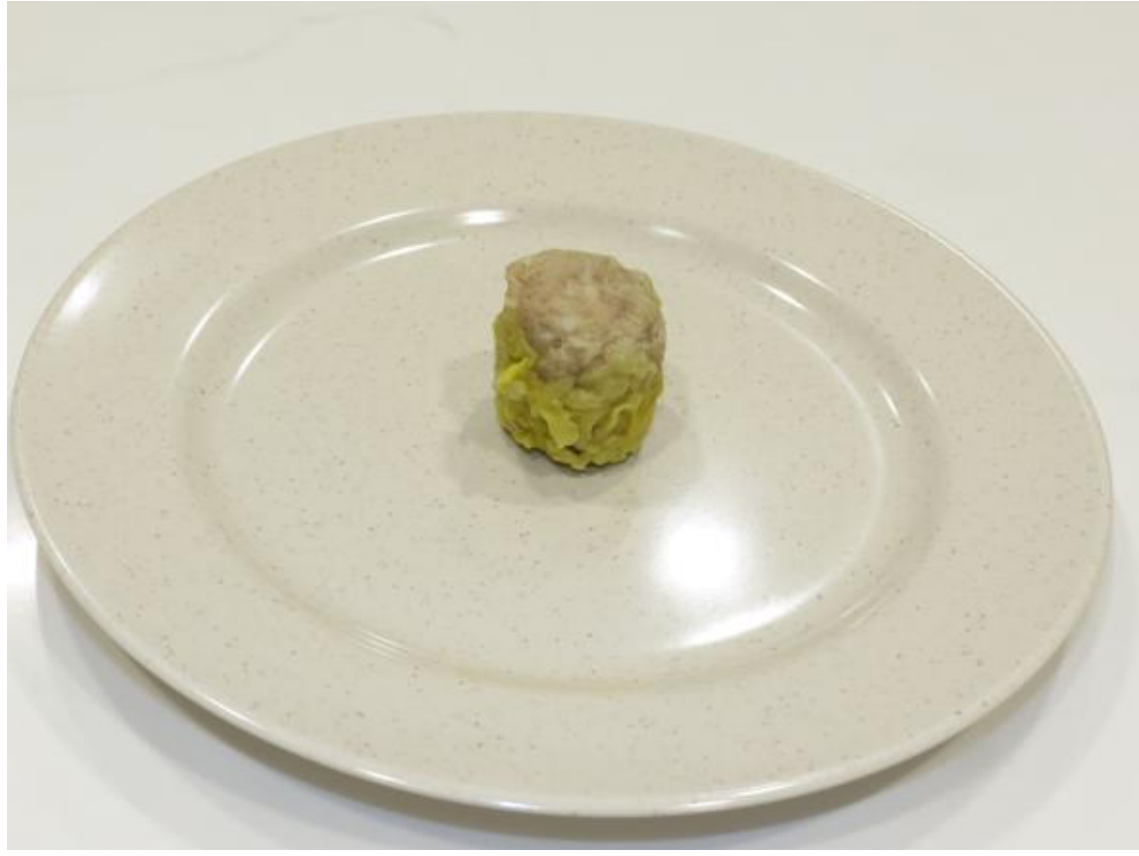

Gravy, braised, thick (FA0090)

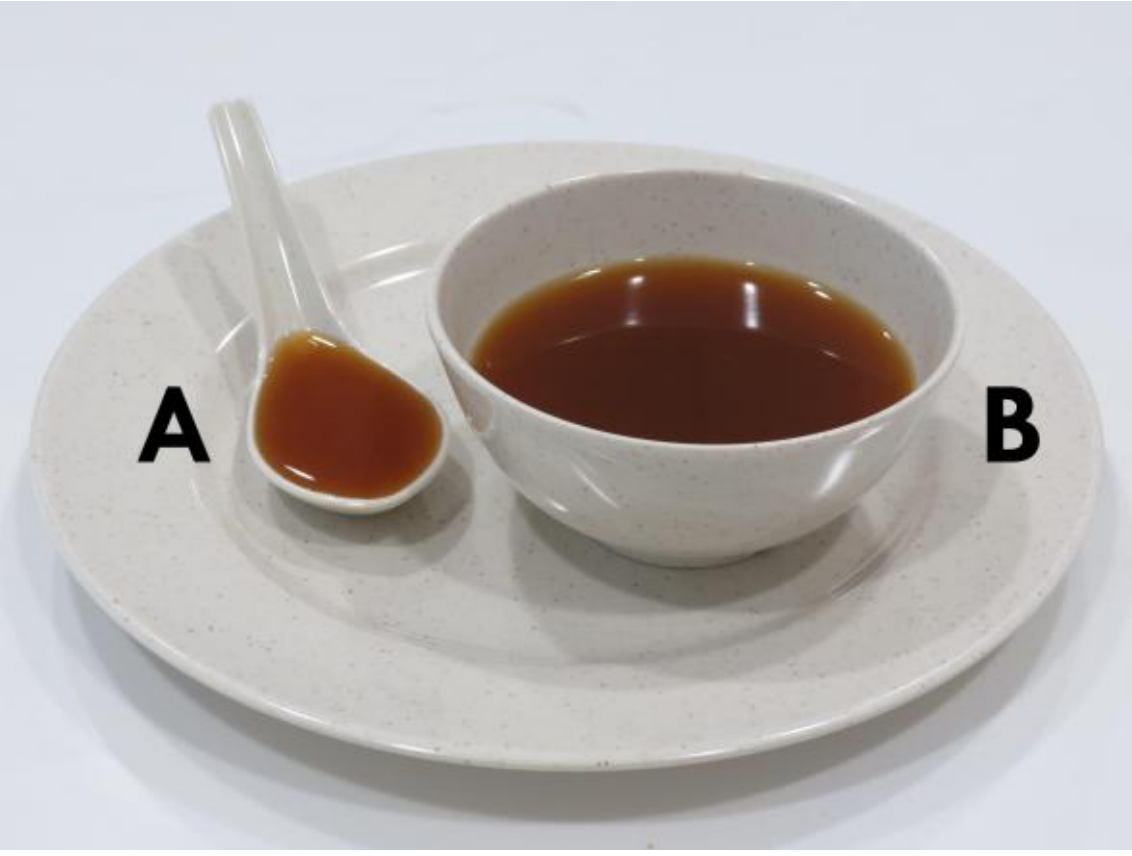

Gravy, curry, thick (FA0082)

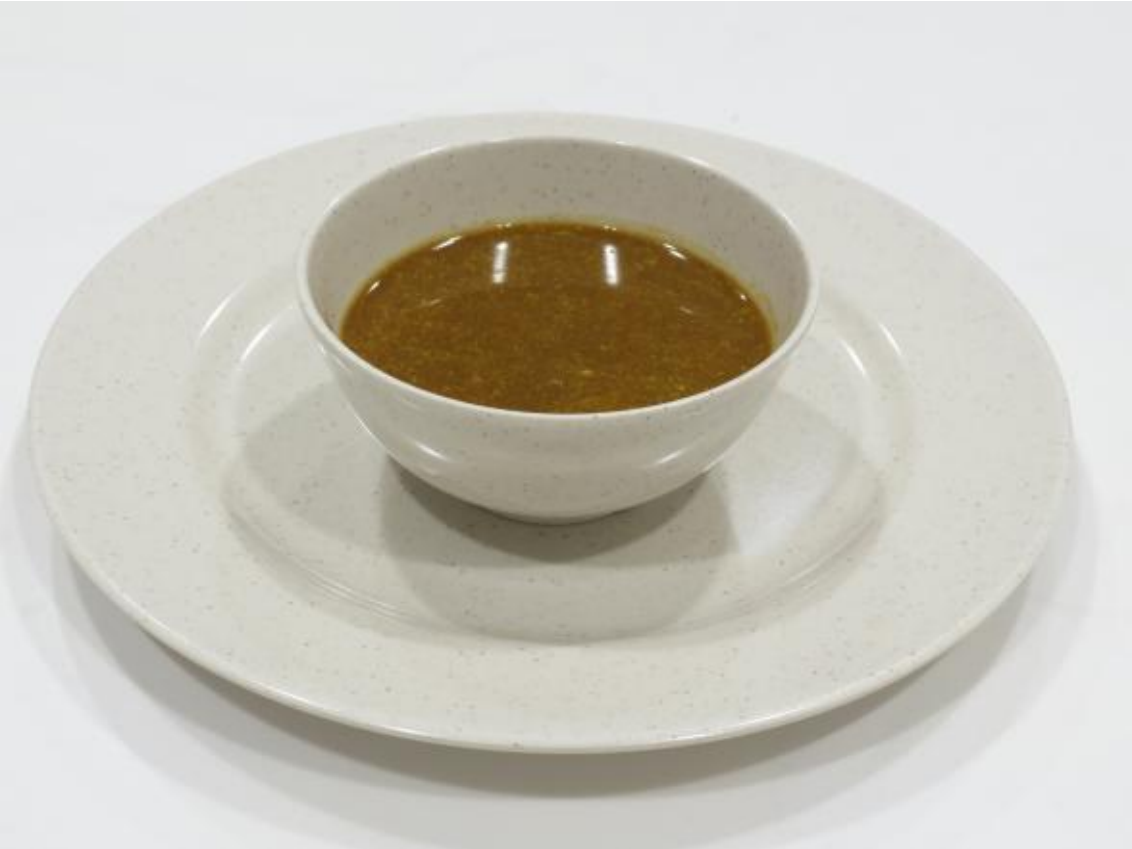

Kimchi (FA0013)

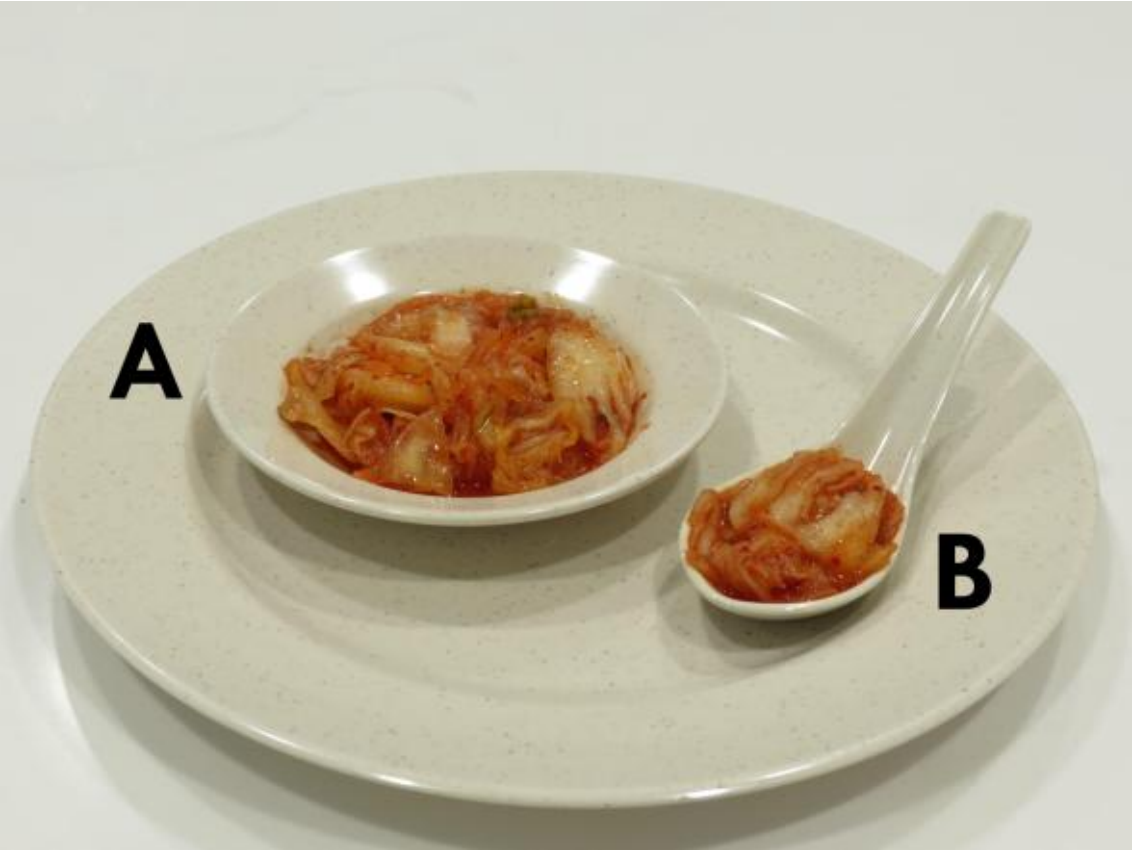

Meat gyoza (FA0161)

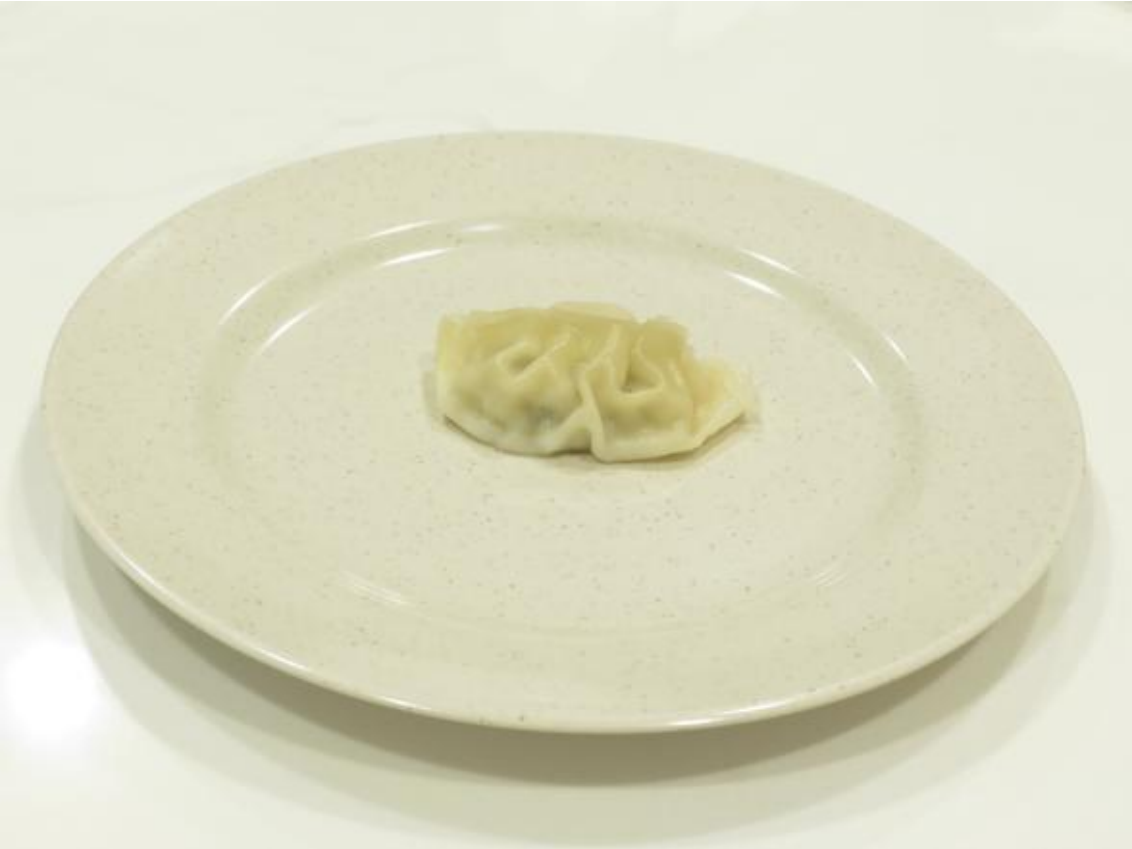

Pie, chicken (FA0029)

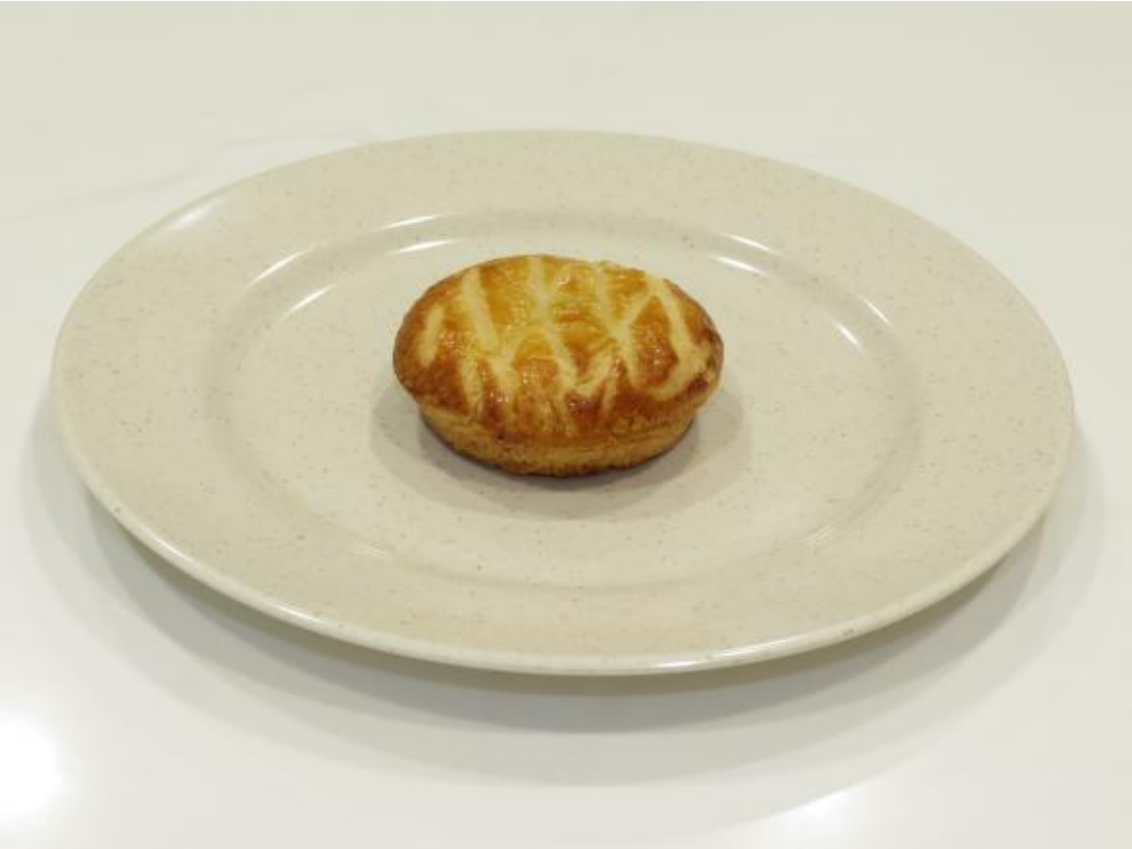

Sesame paste (FA0021)

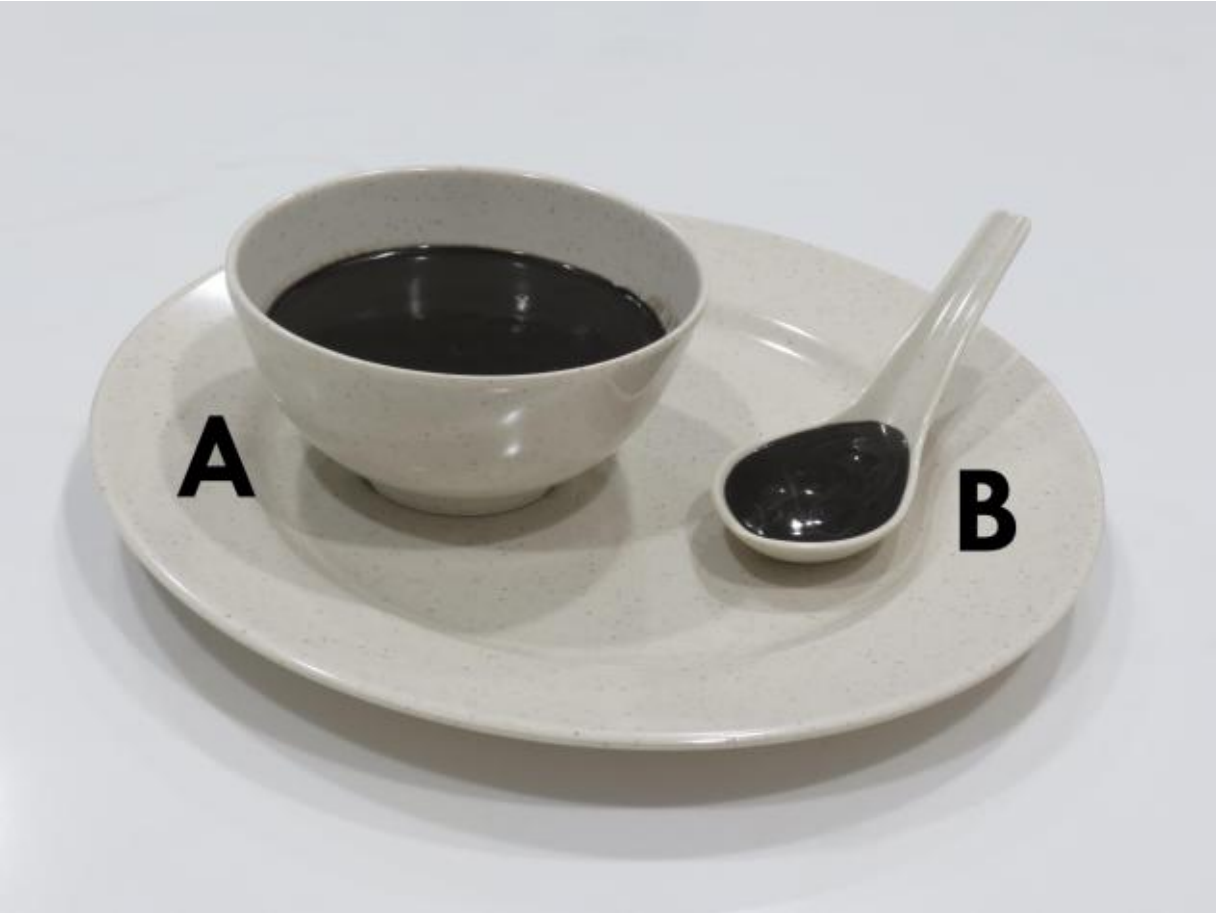

Spring roll, fried (FA0016)

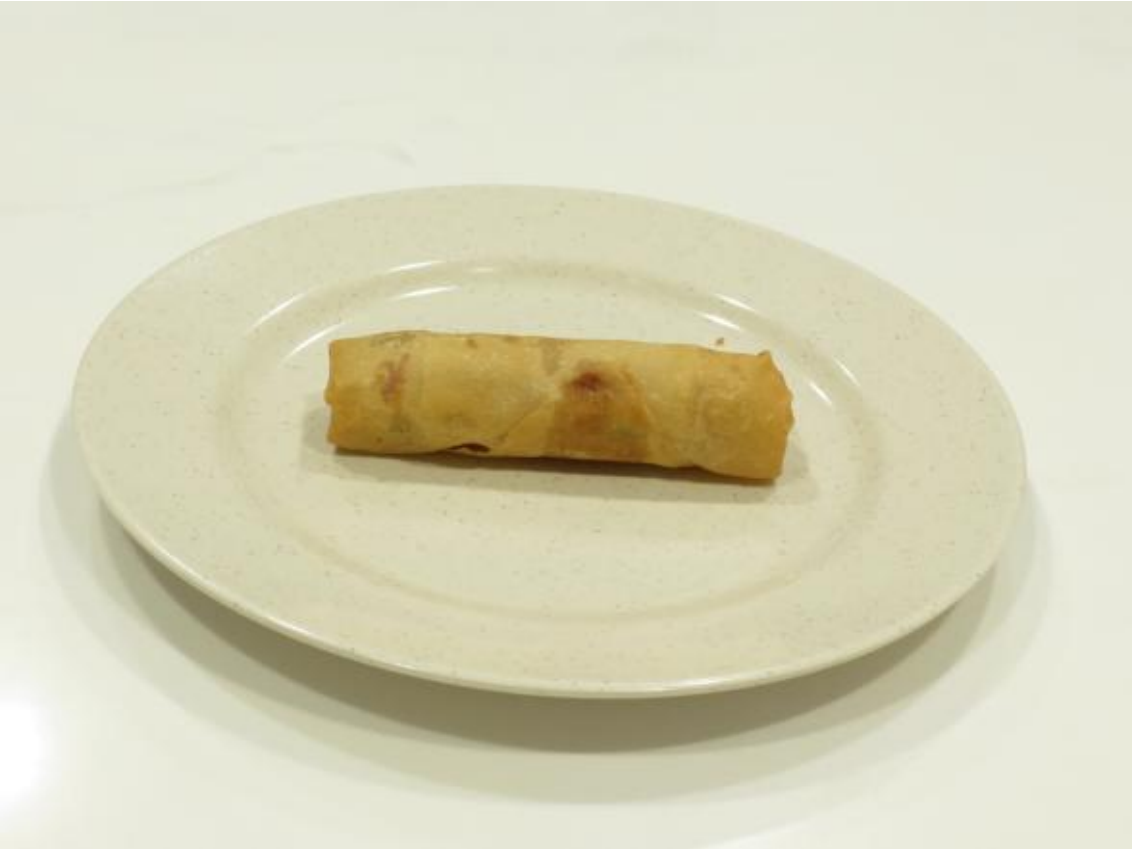

Sushi, salmon (FA0178)

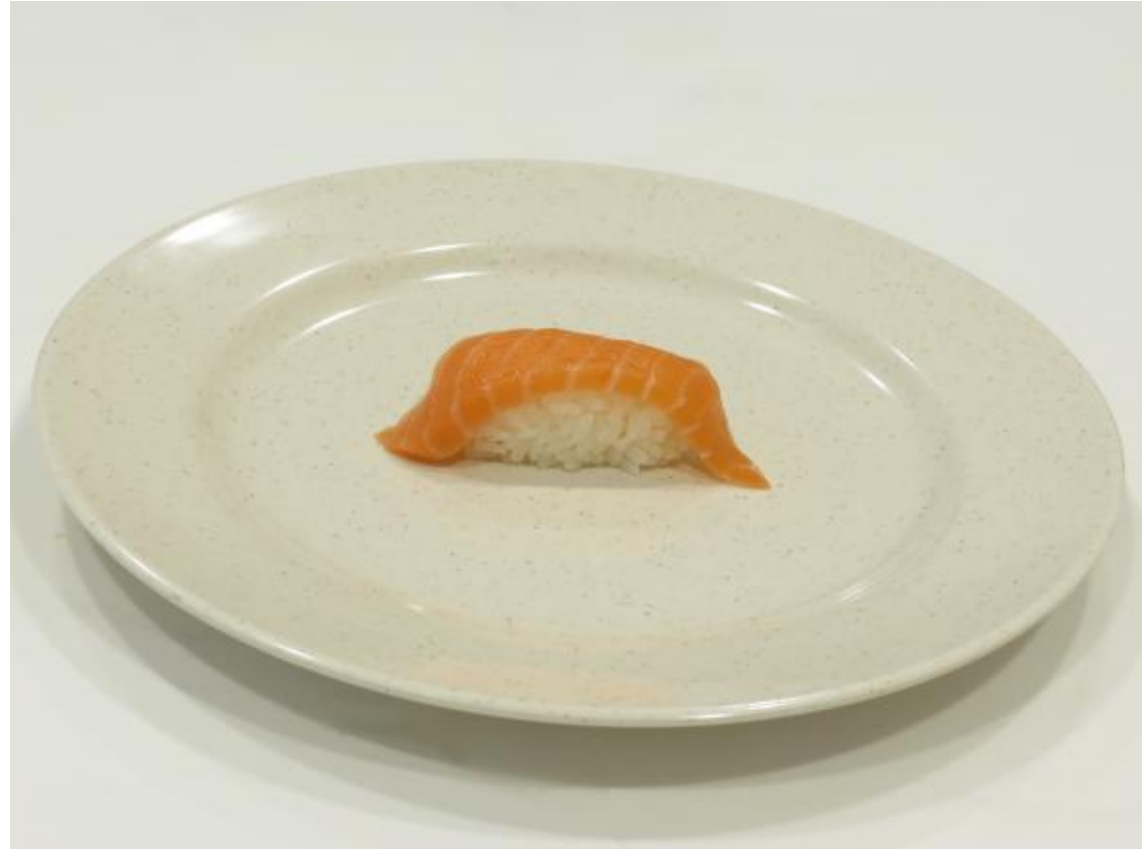

Yam paste (orh nee) (FA0020)

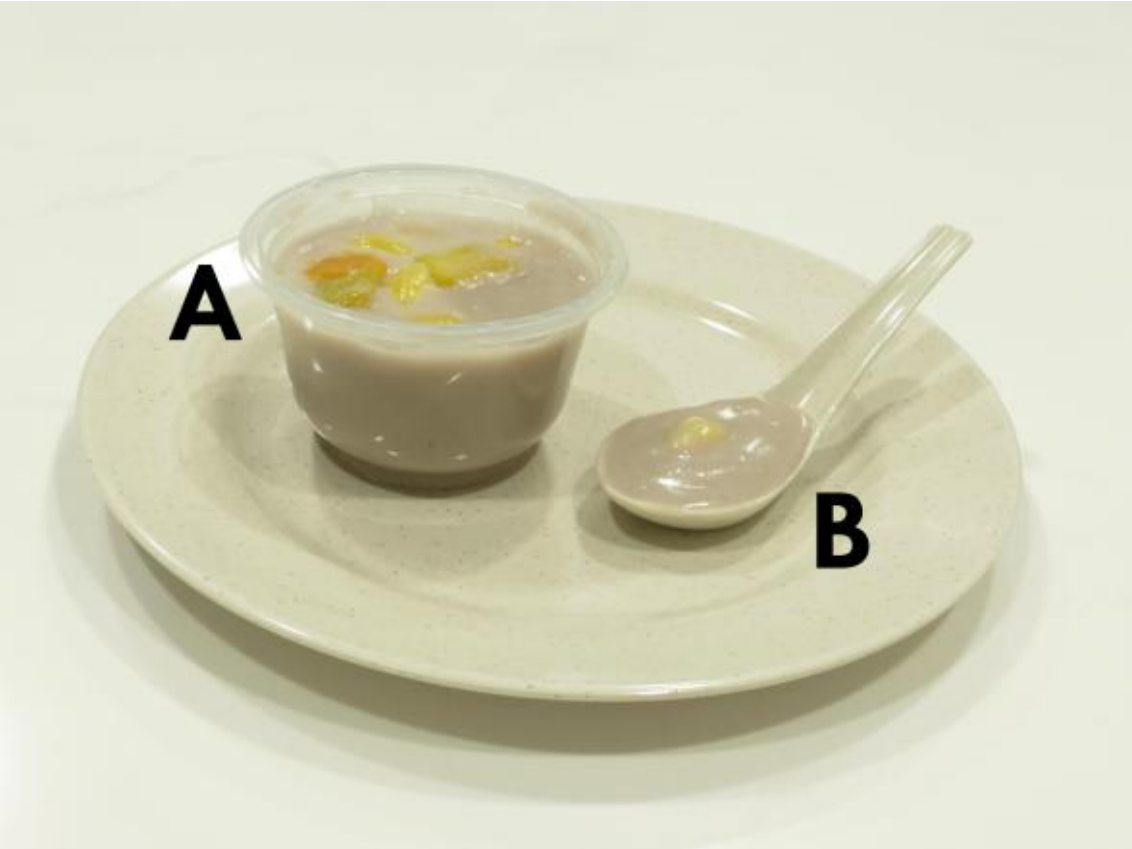

Chocolate, block (FA0060)

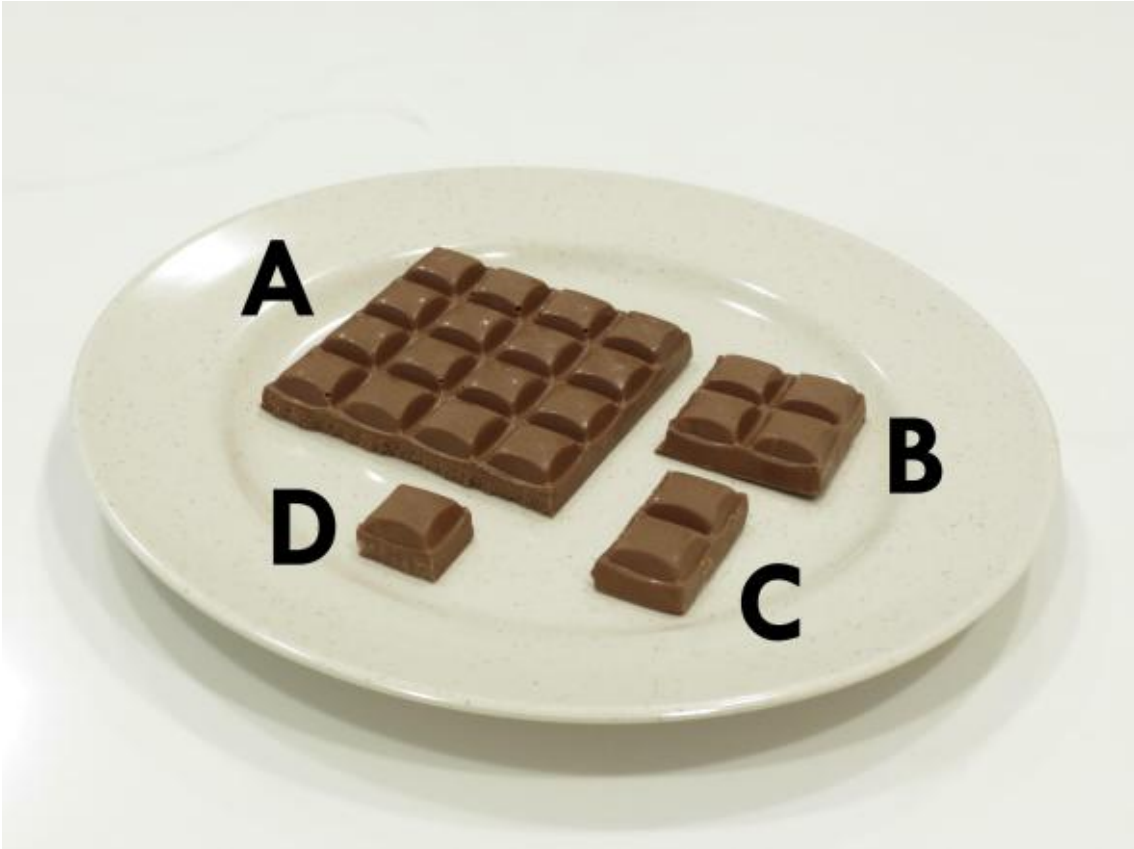

Palm sugar (gula melaka)(FA0188)

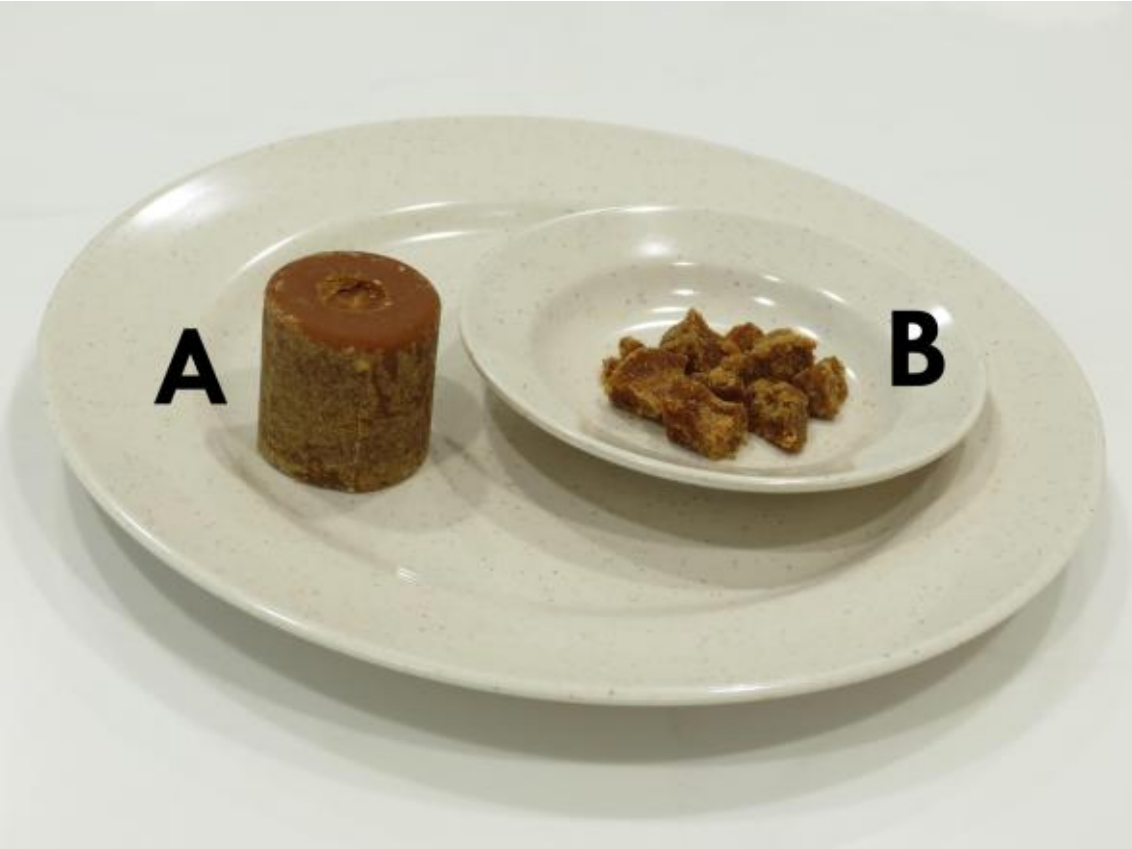

Pudding, mango (FA0019)

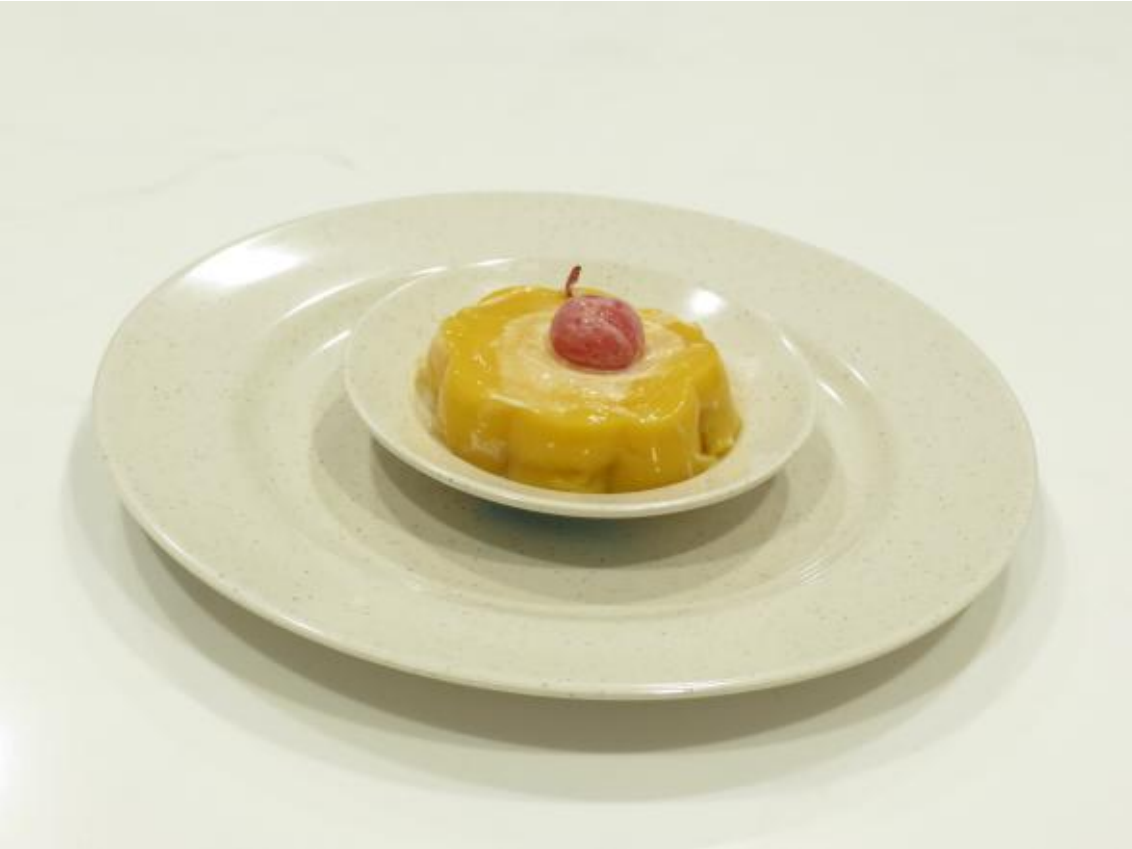

Egg, hard boiled (FA0069)

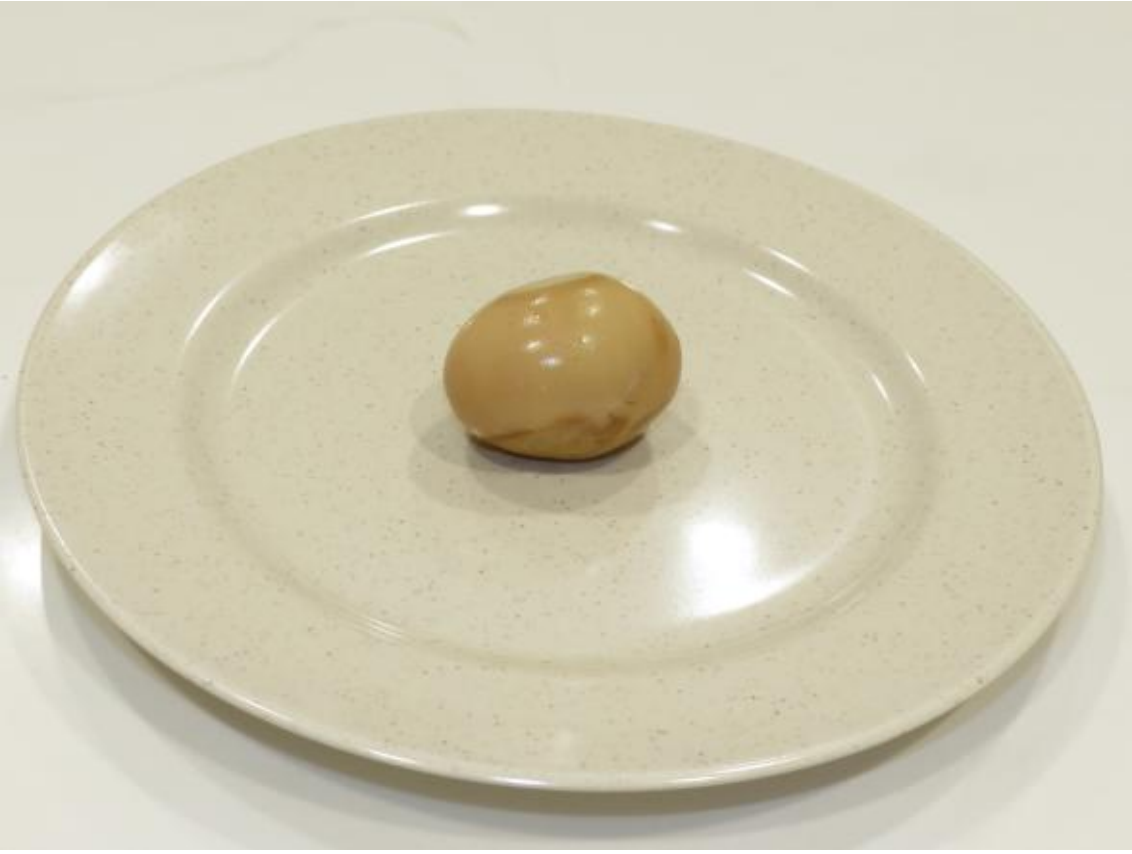

Abalone, baby (FA0088)

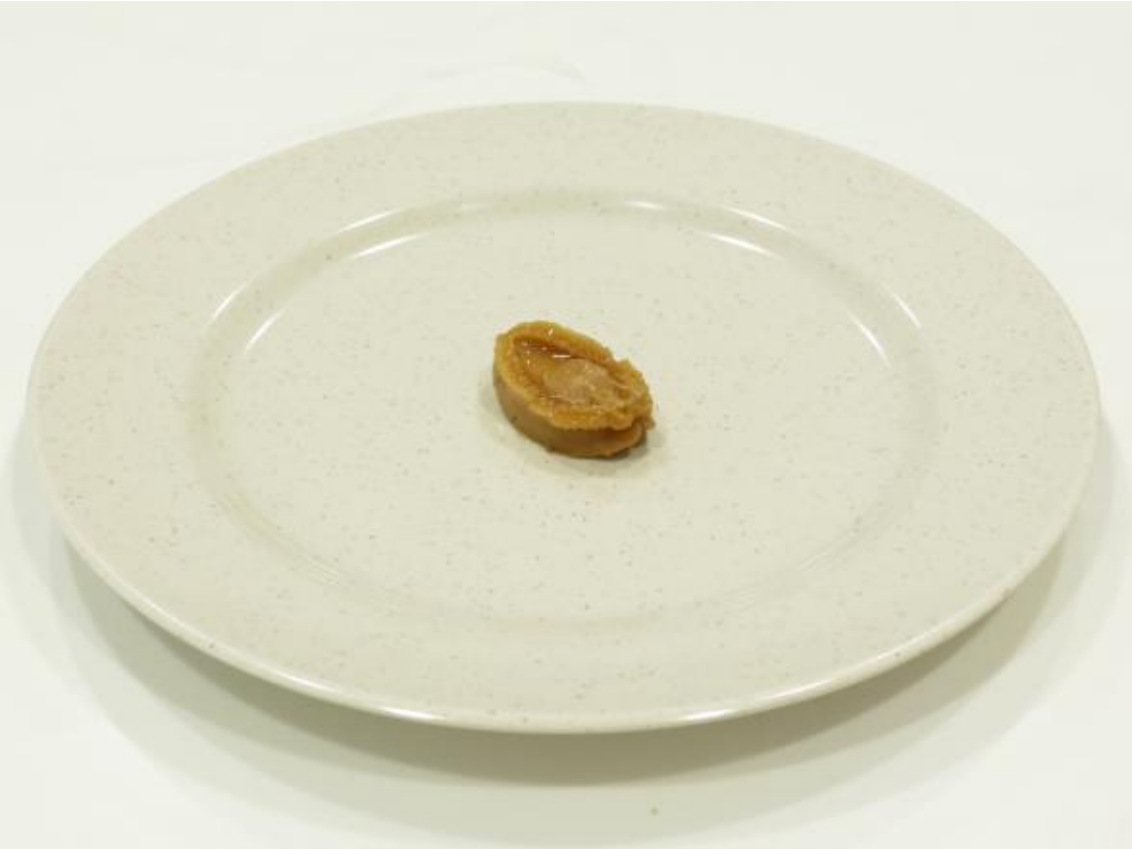

Abalone, sliced (FA0162)

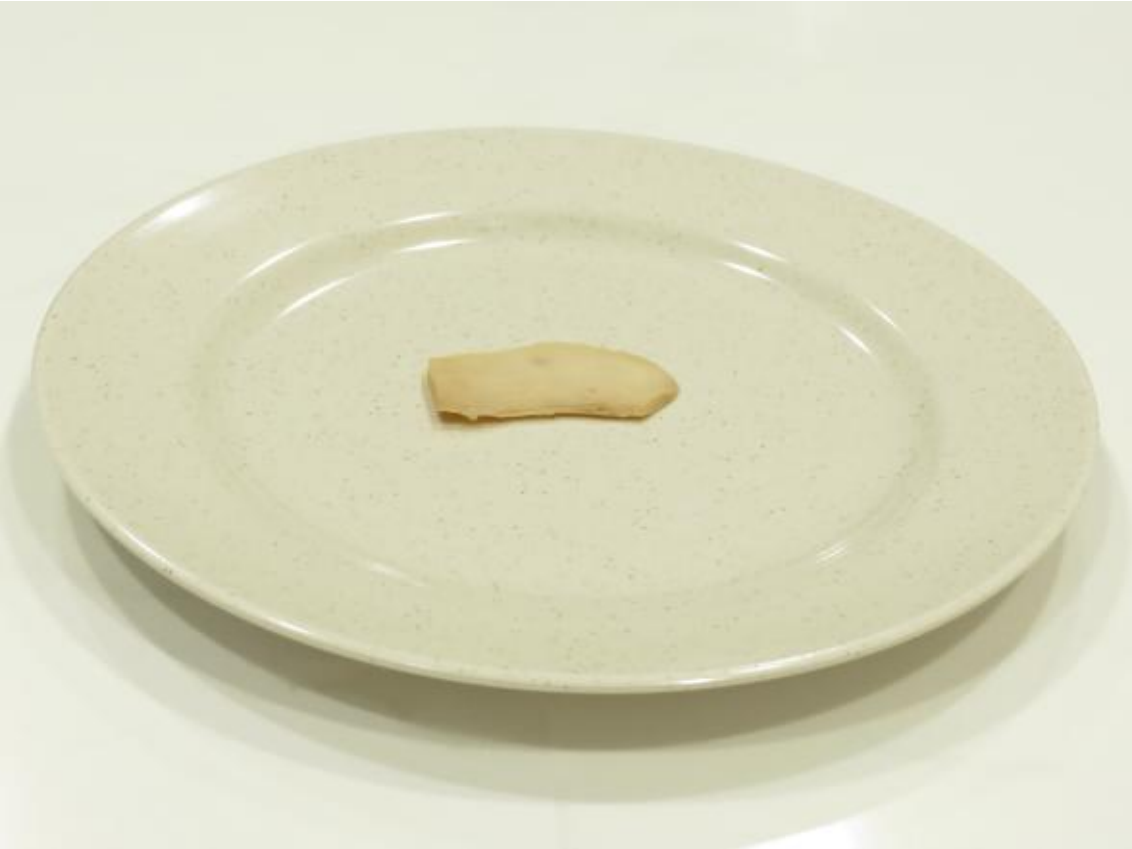

Crabstick, whole (FA0148)

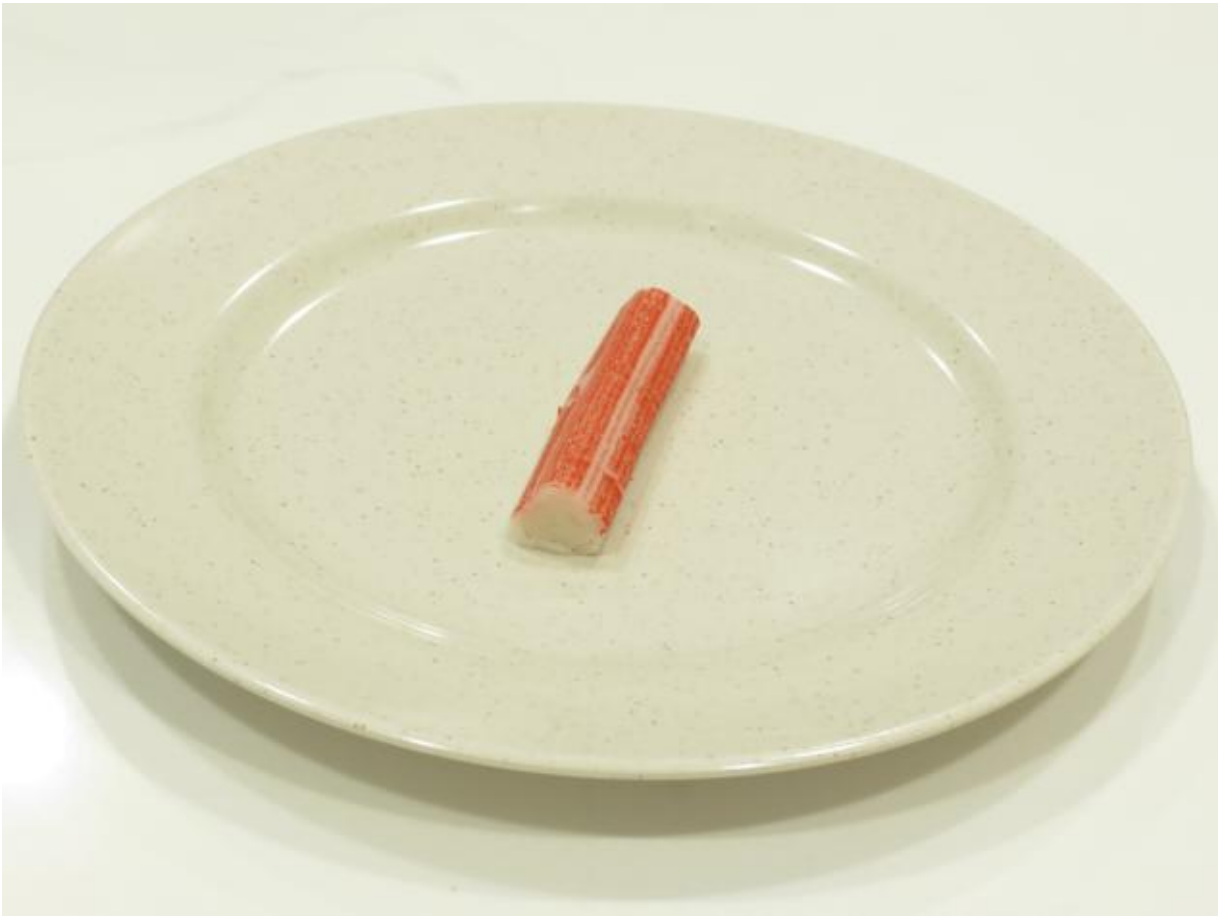

Fish cake, sliced (FA0068)

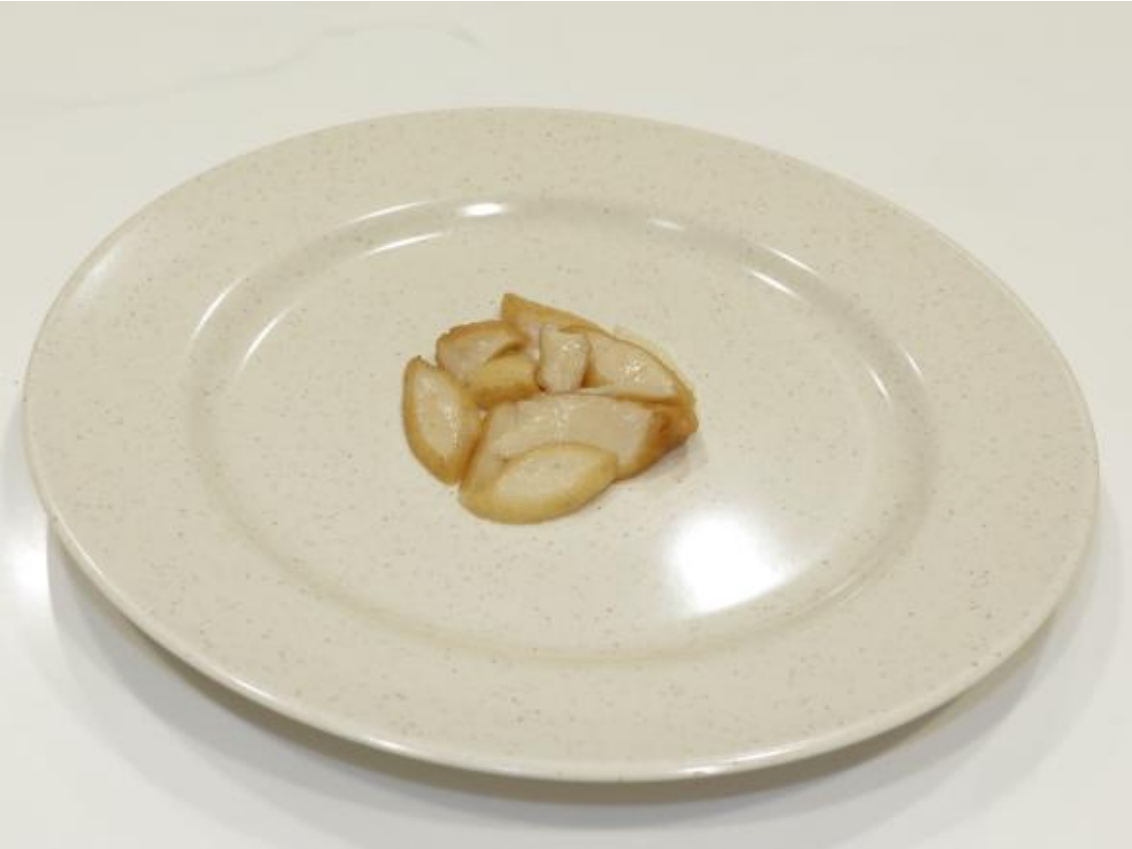

Fish, battered, boneless (FA0102)

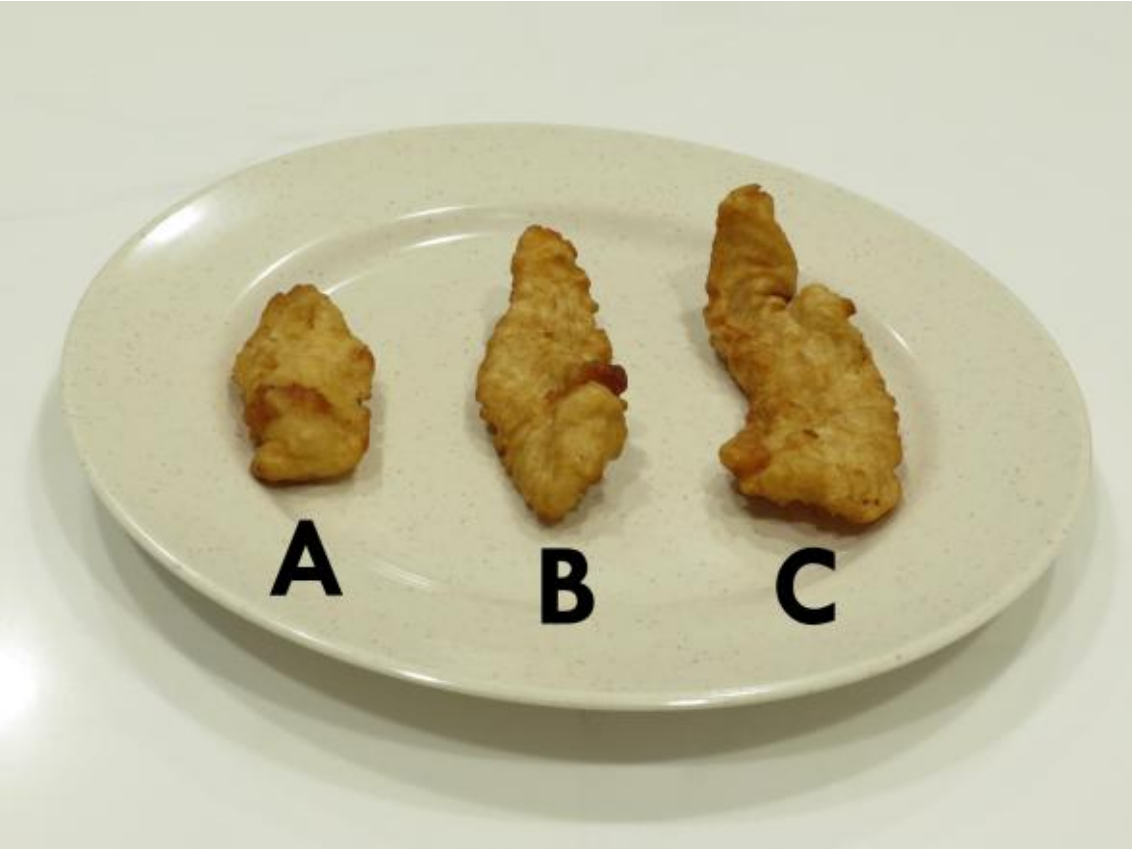

Fish, grilled, boneless (FA0168)

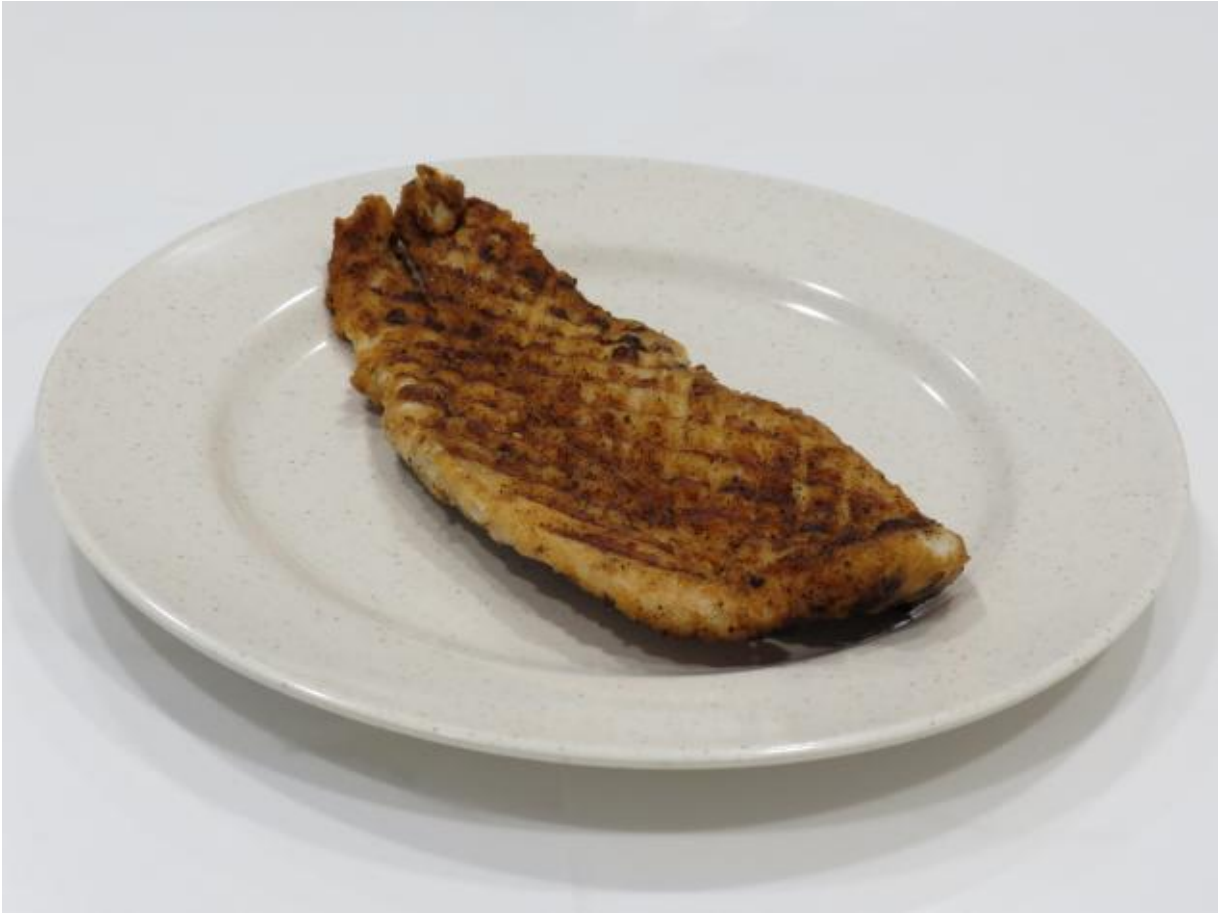

Fish, ikan selar (FA0152)

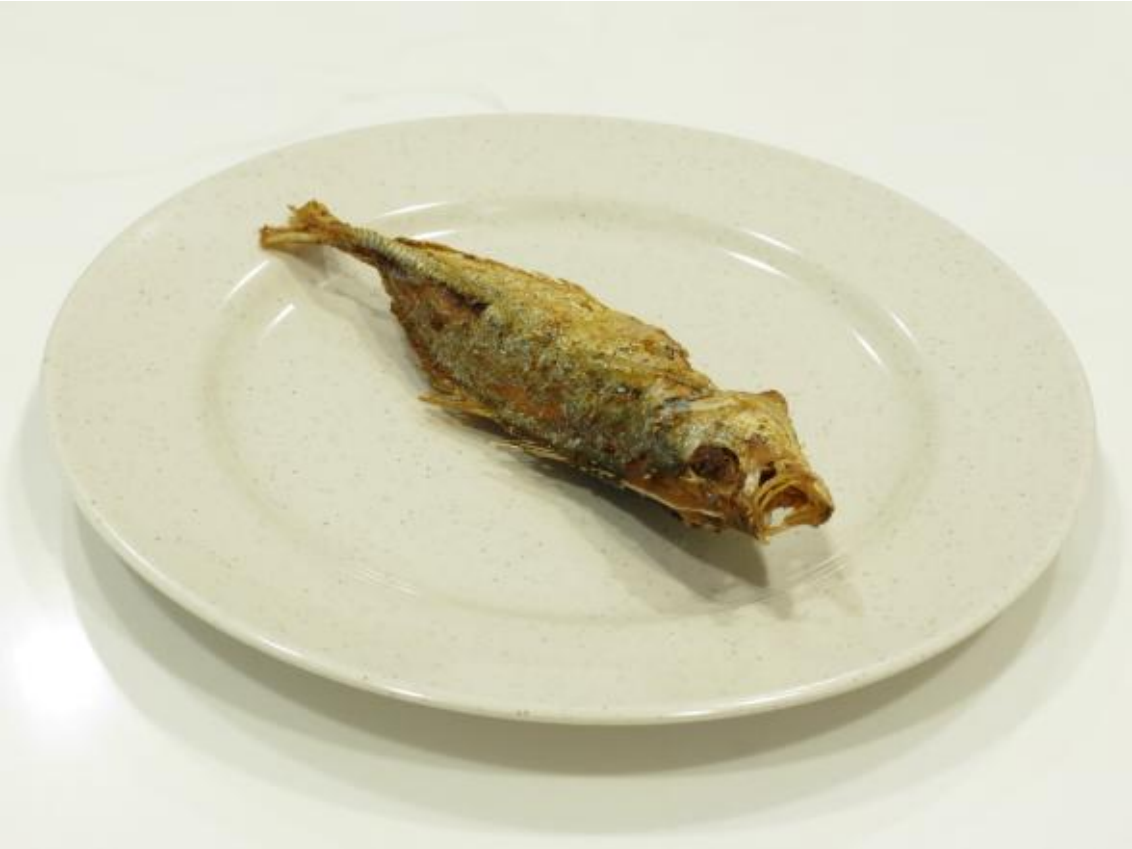

Fish, pomfret, fried (FA0154)

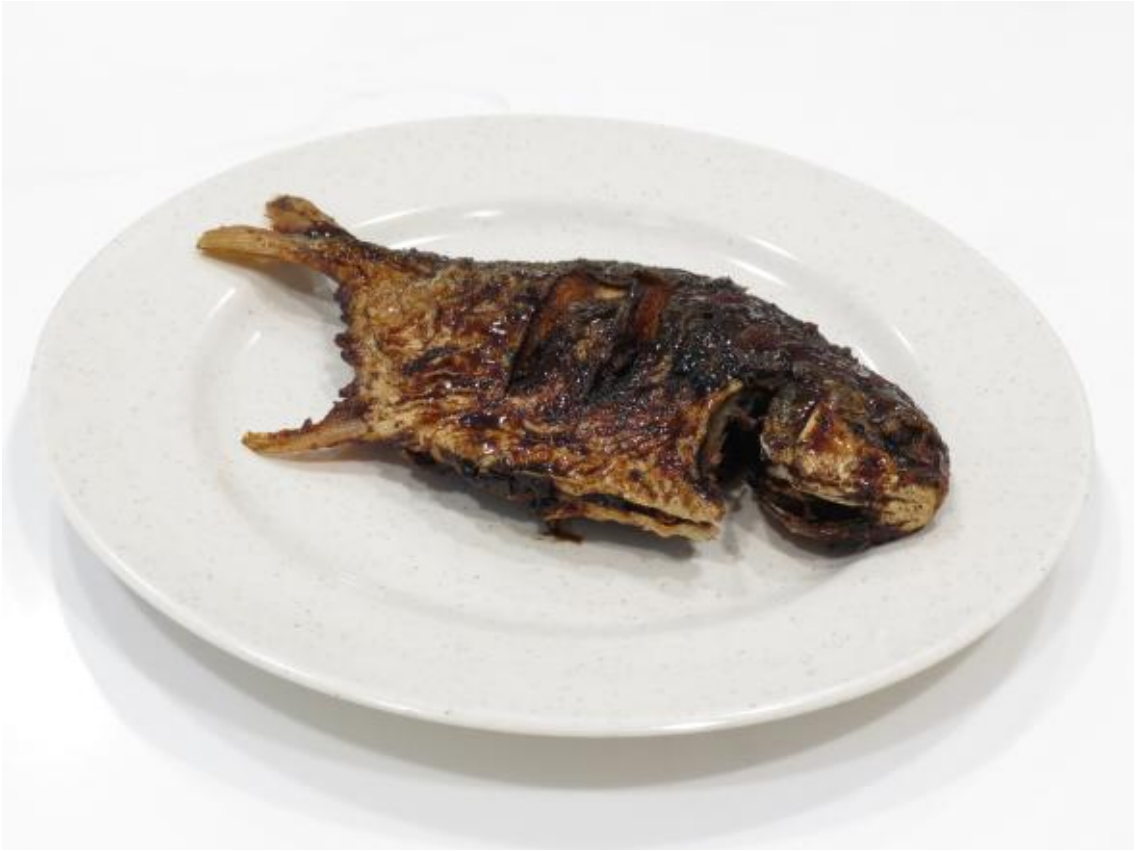

Fish, raw (sashimi) (FA0177)

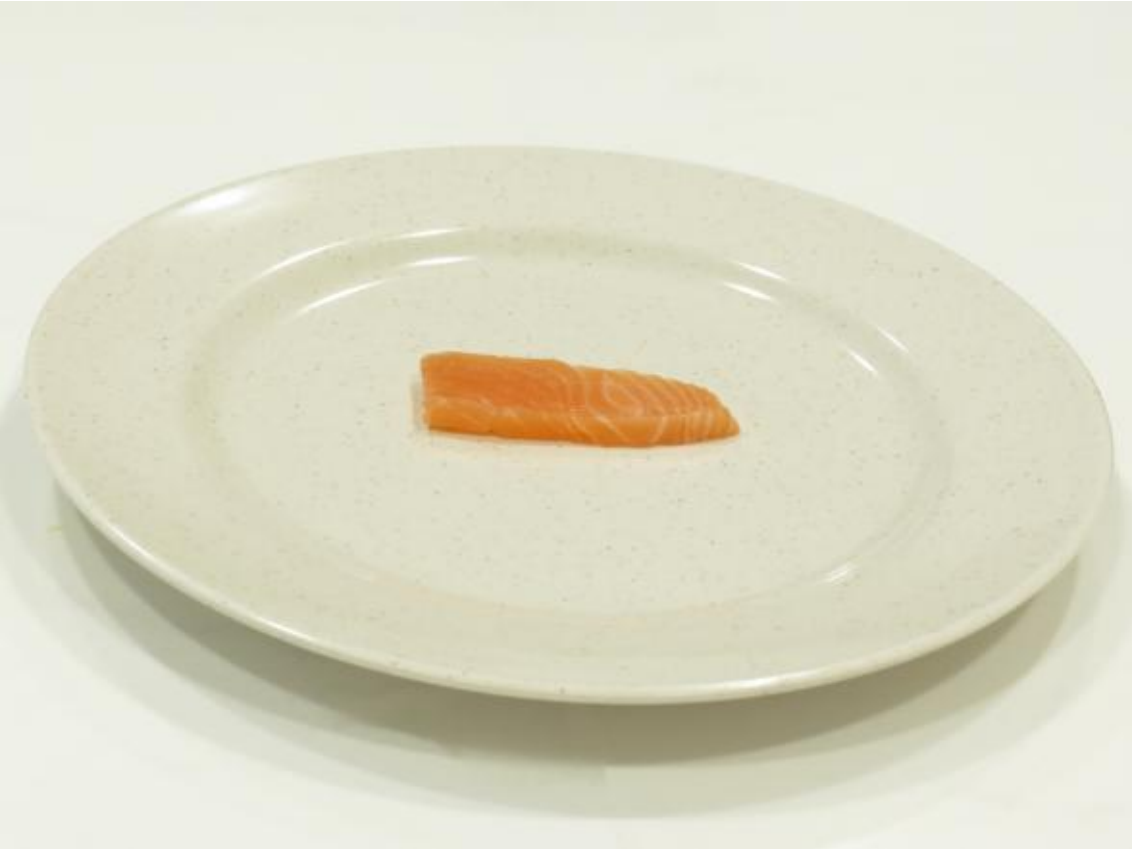

Fish, saba (FA0153)

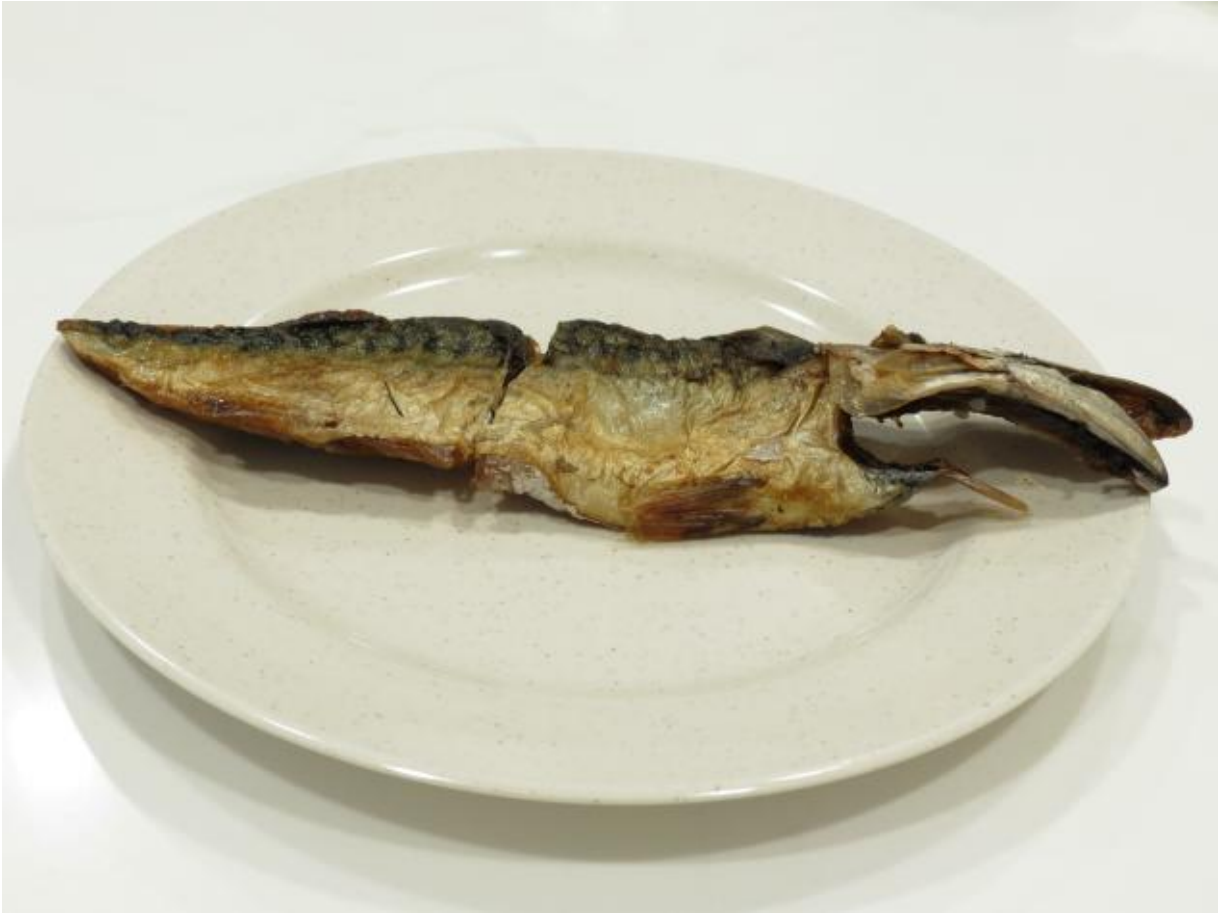

Fish, salmon, fillet (FA0151)

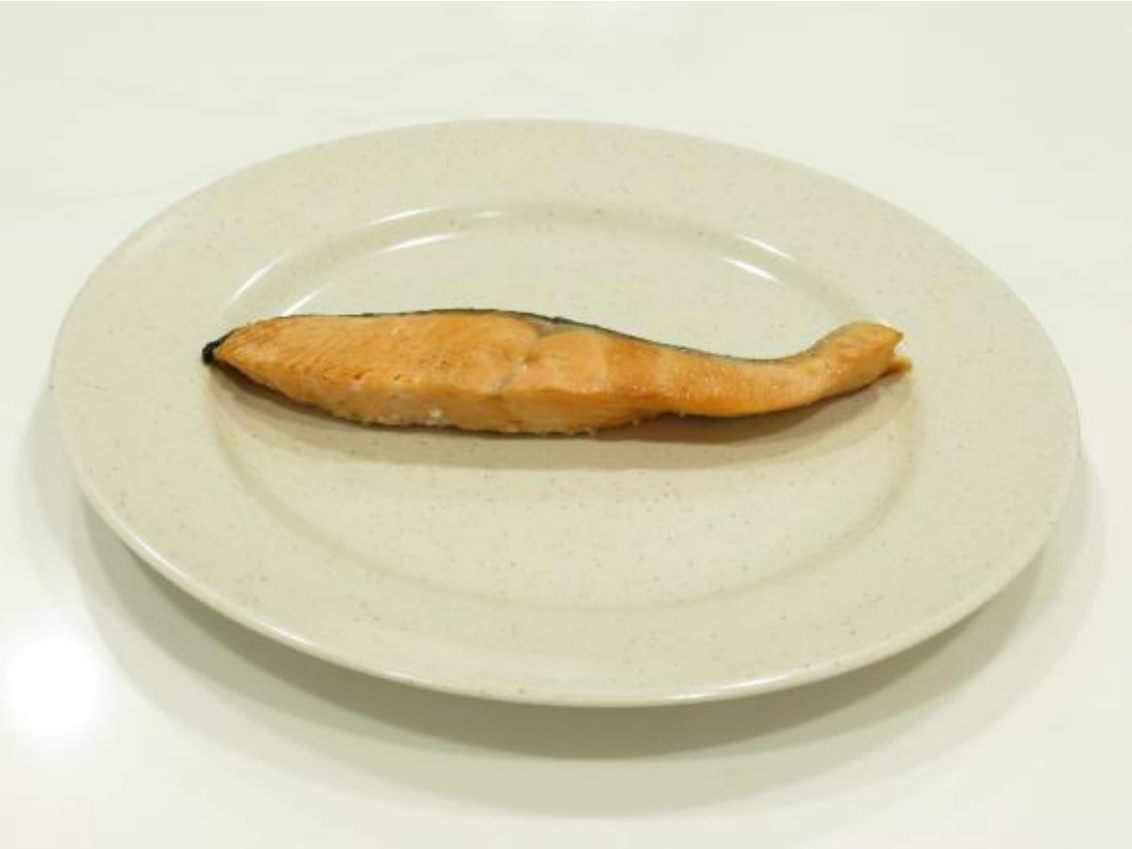

Fish, sardine (FA0111)

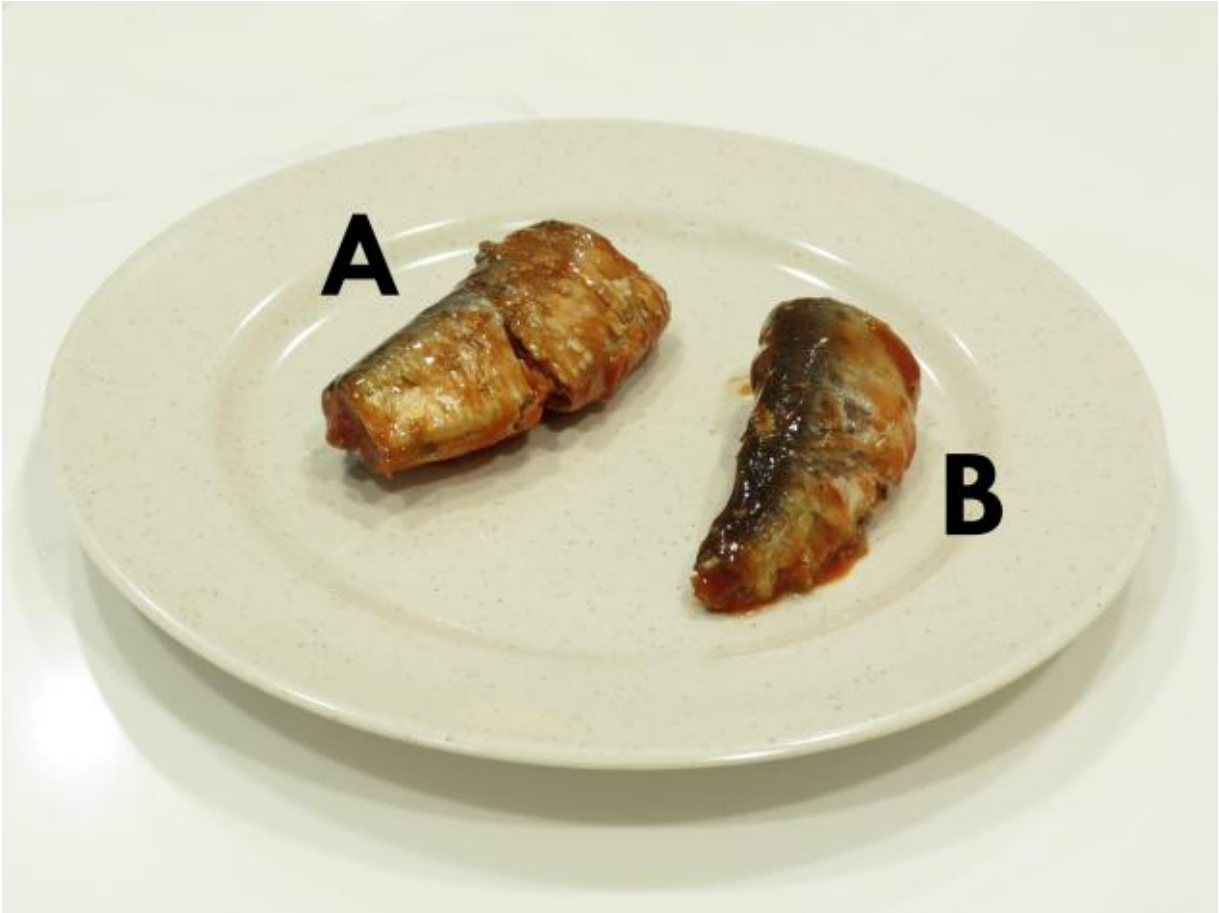

Fishball (FA0119)

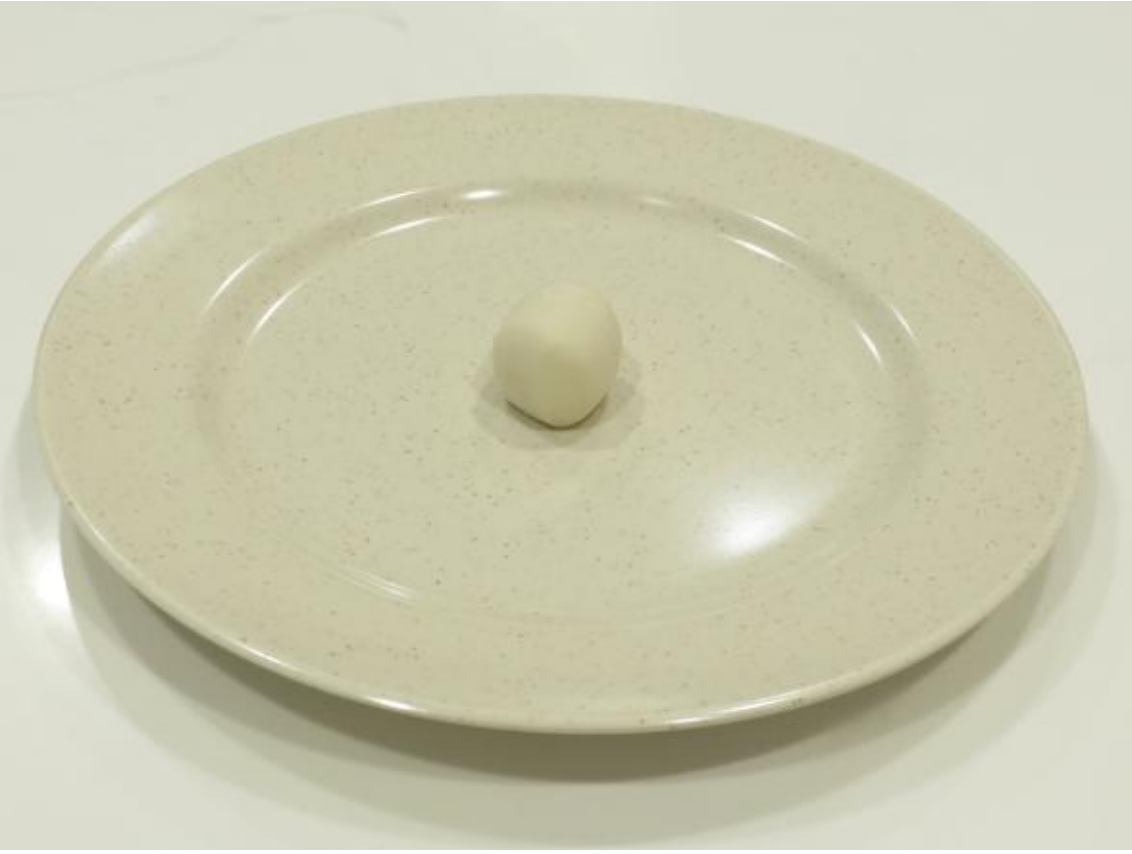

Mussel (FA0179)

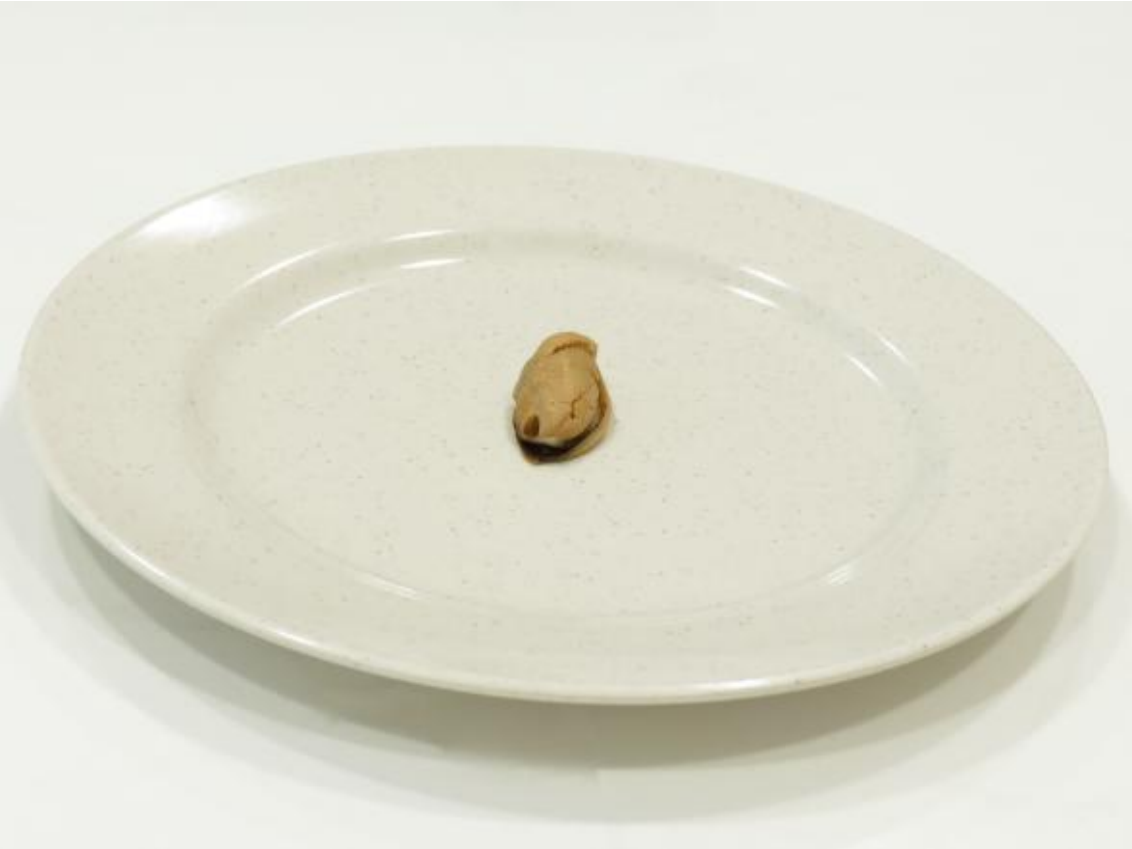

Otah, assorted (FA0063)

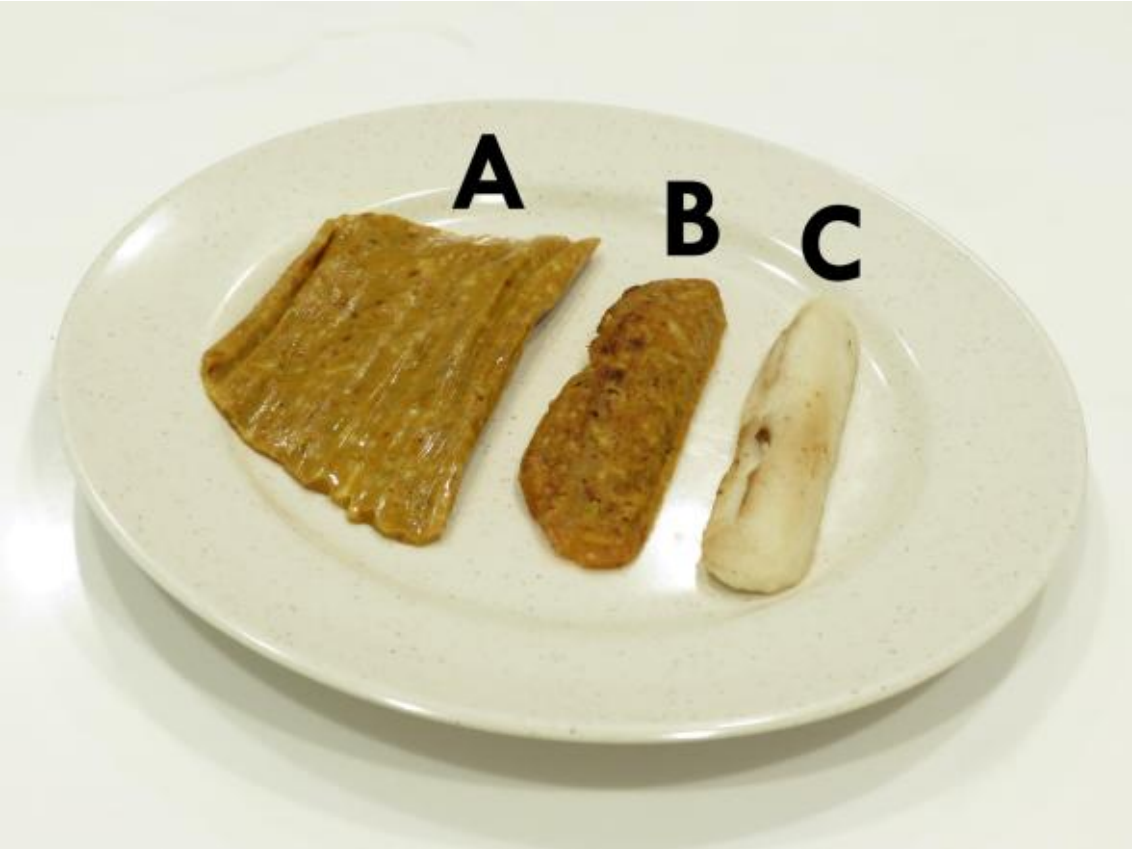

Prawn, regular, deshelled (FA0135)

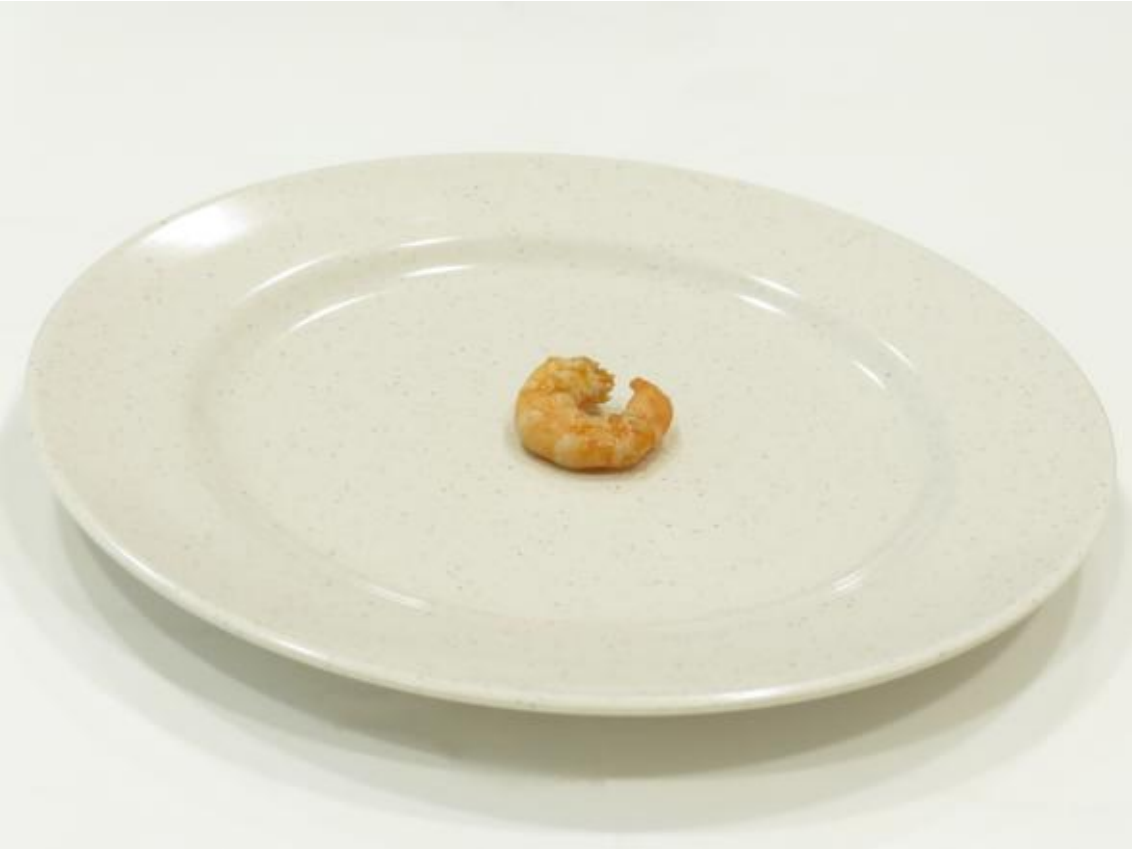

Prawn, king, deshelled (FA0182)

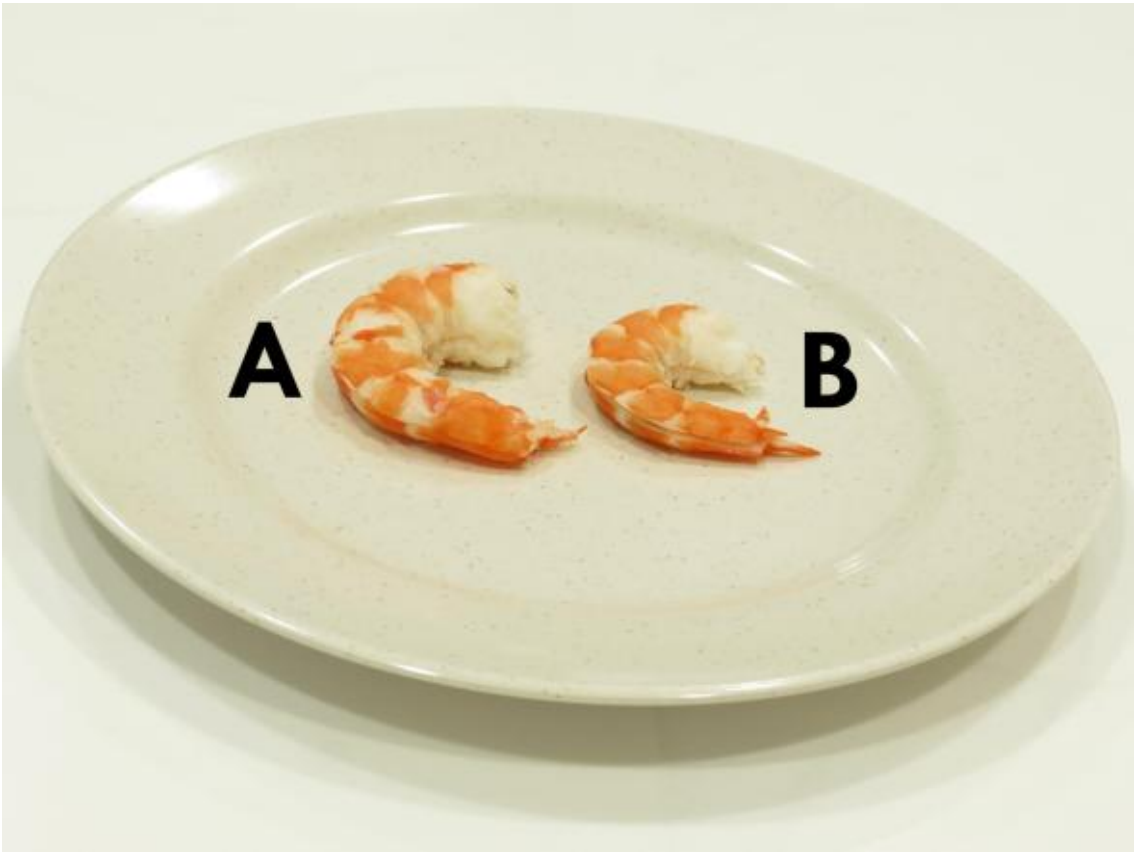

Scallop (FA0163)

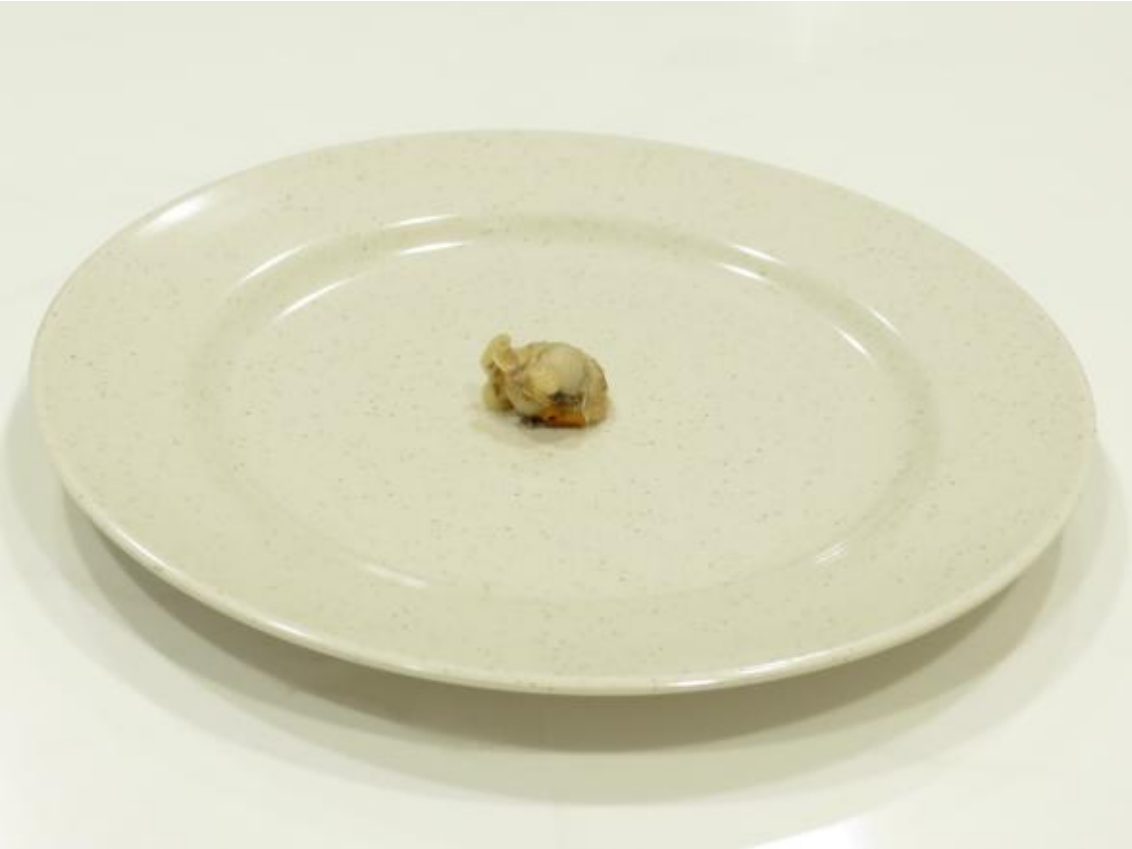

Apple, sliced (FA0046)

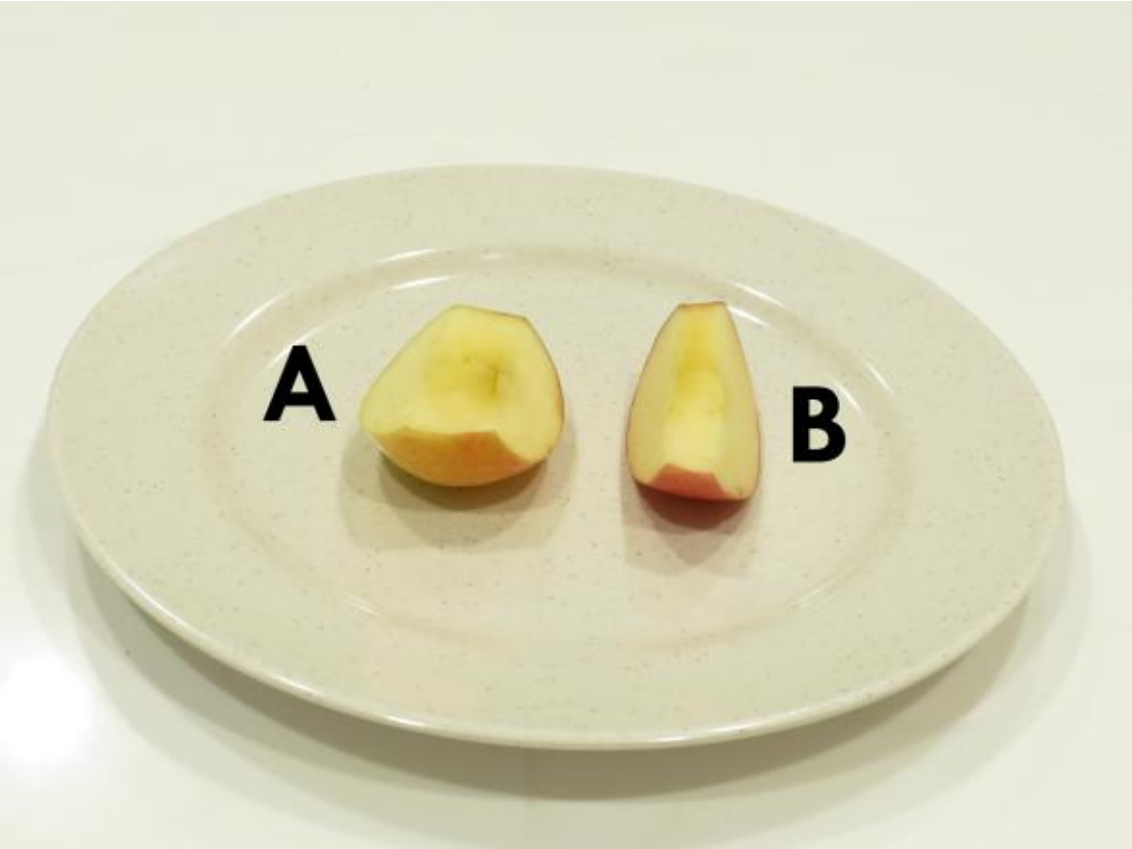

Banana, fried (FA0017)

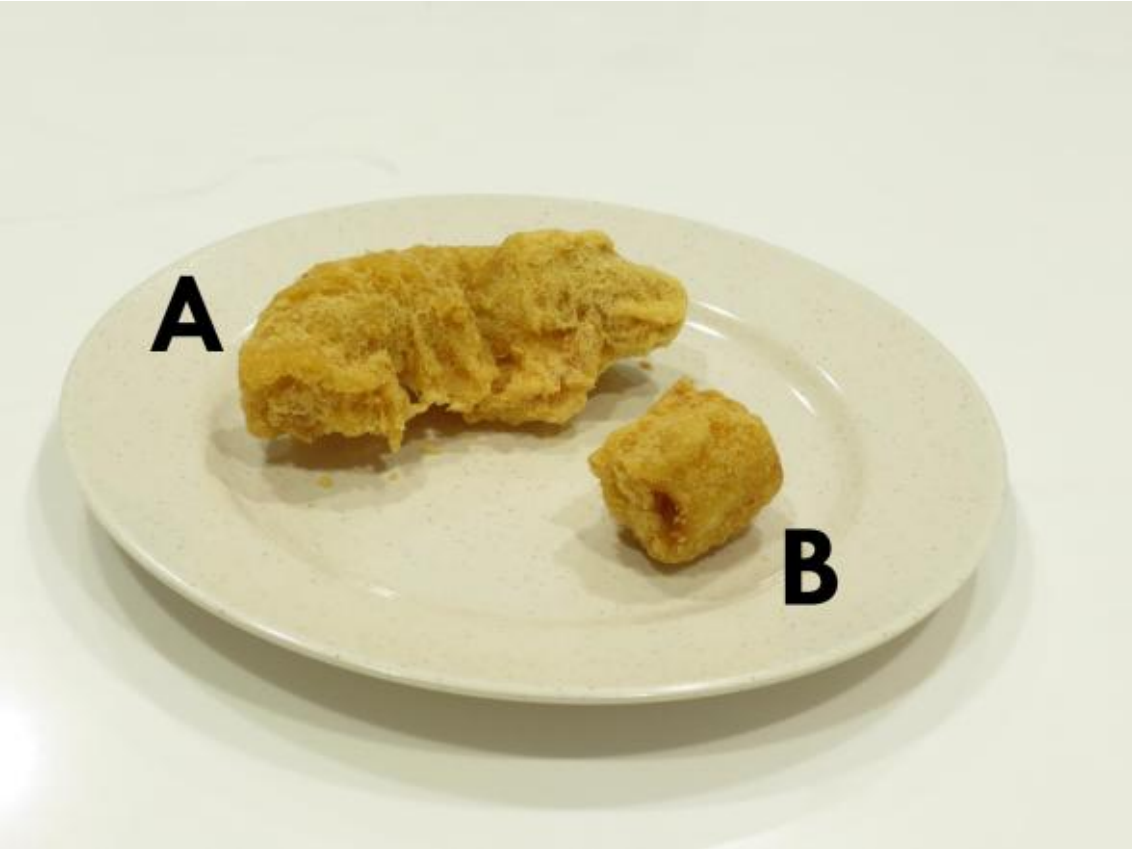

Banana, regular (FA0174)

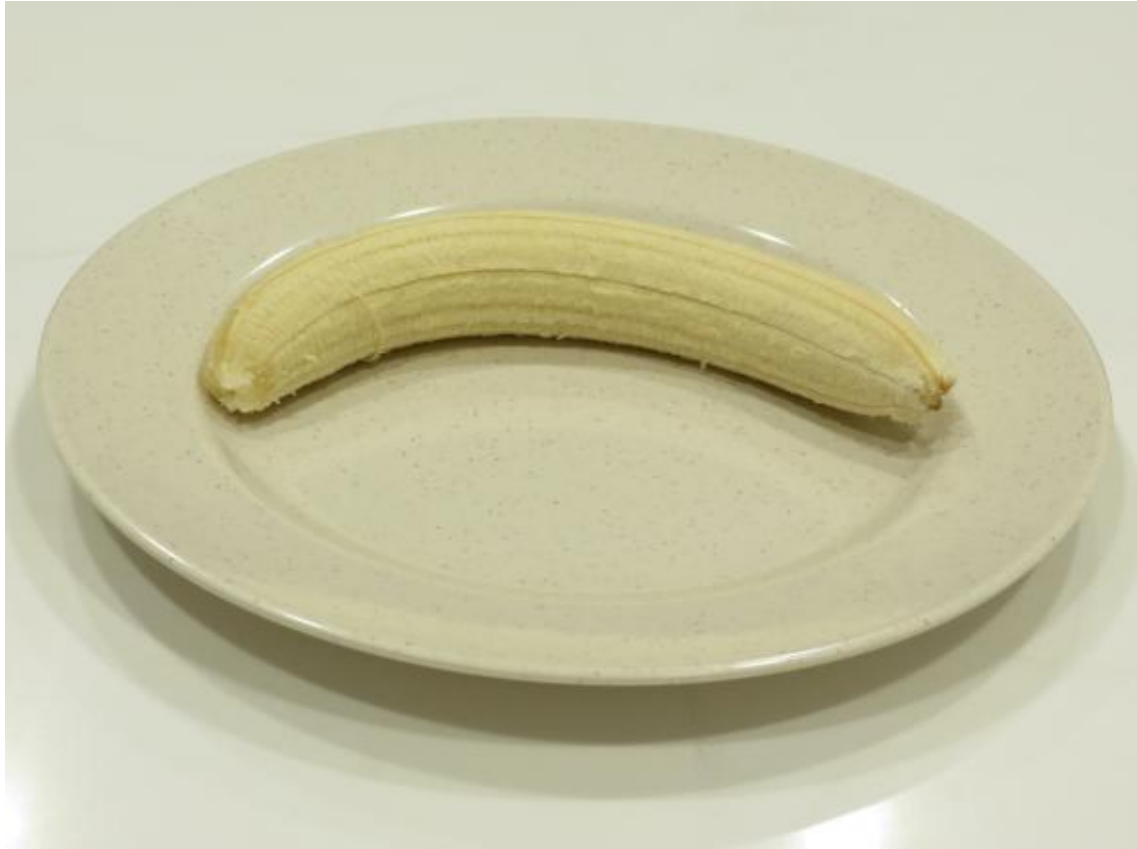

Banana, small (FA0204)

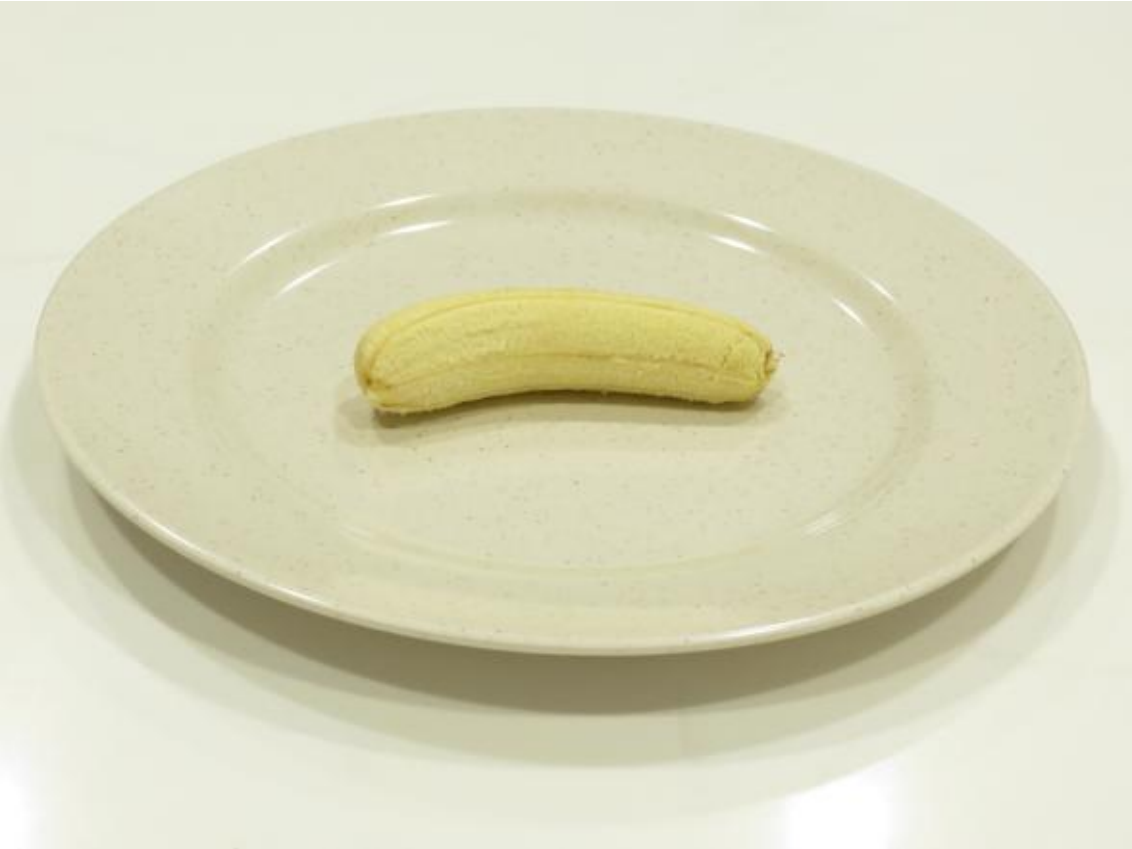

Dragon fruit, white flesh, wedge (FA0050)

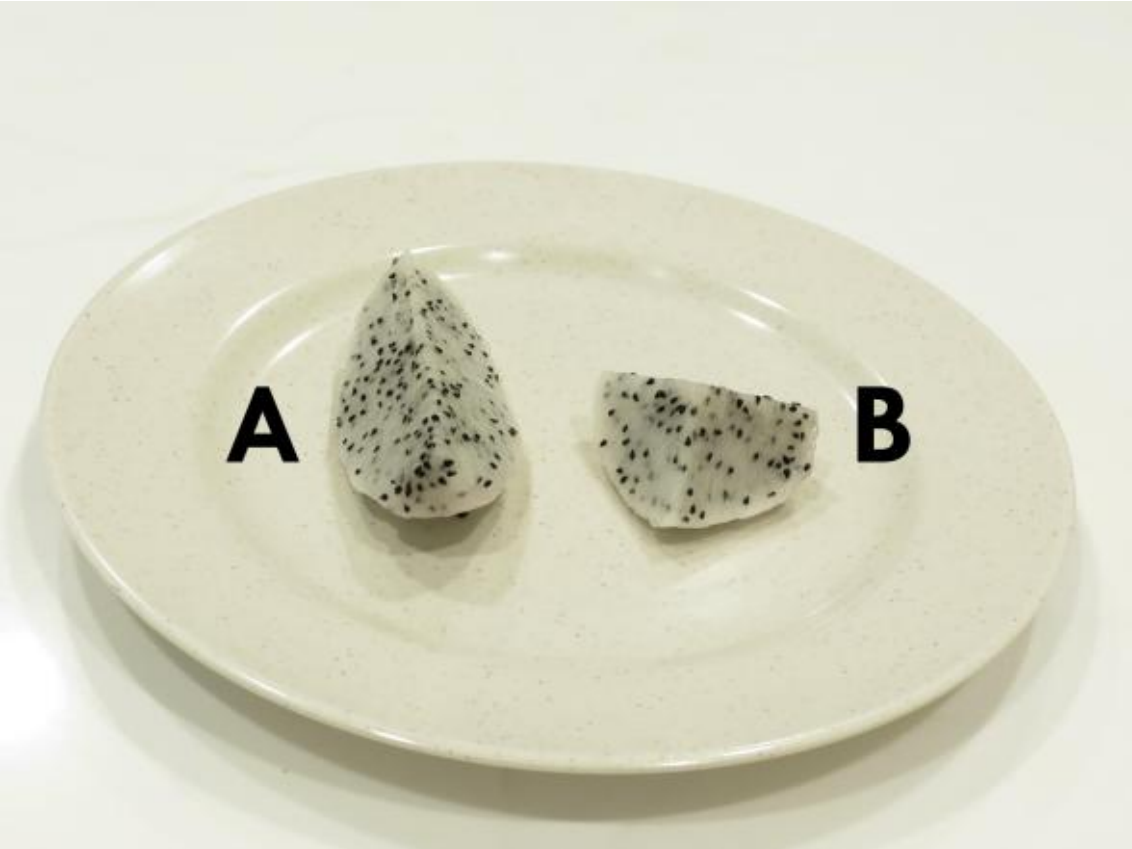

Grape (FA0005)

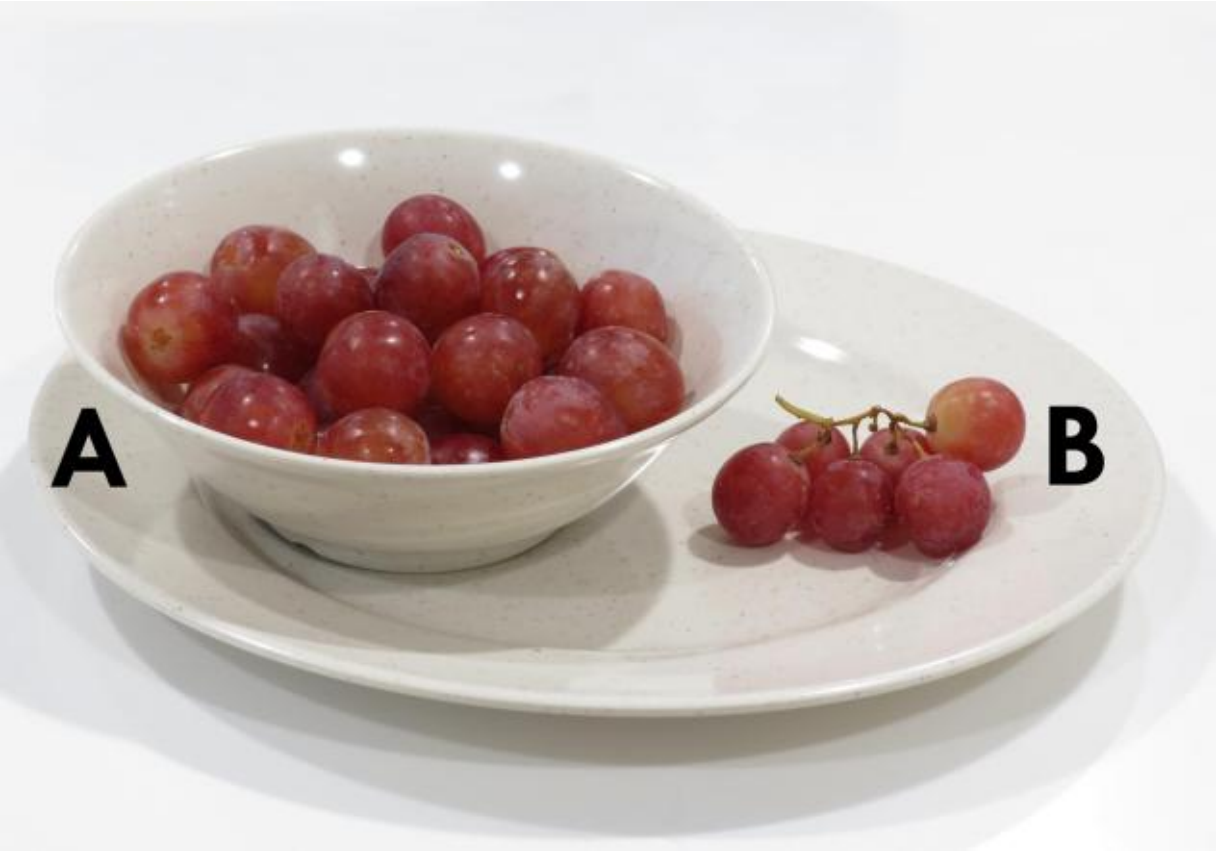

Kiwi, sliced (FA0049)

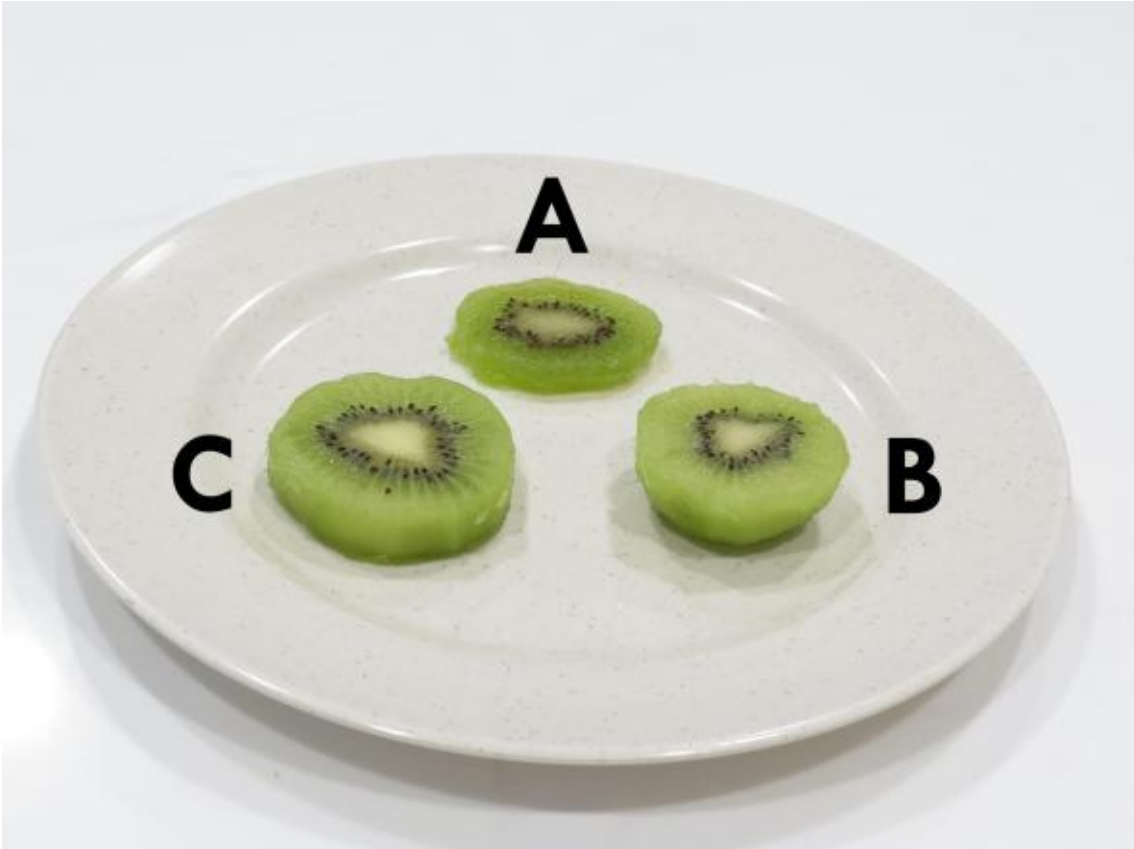

Mango, dried (FA0009)

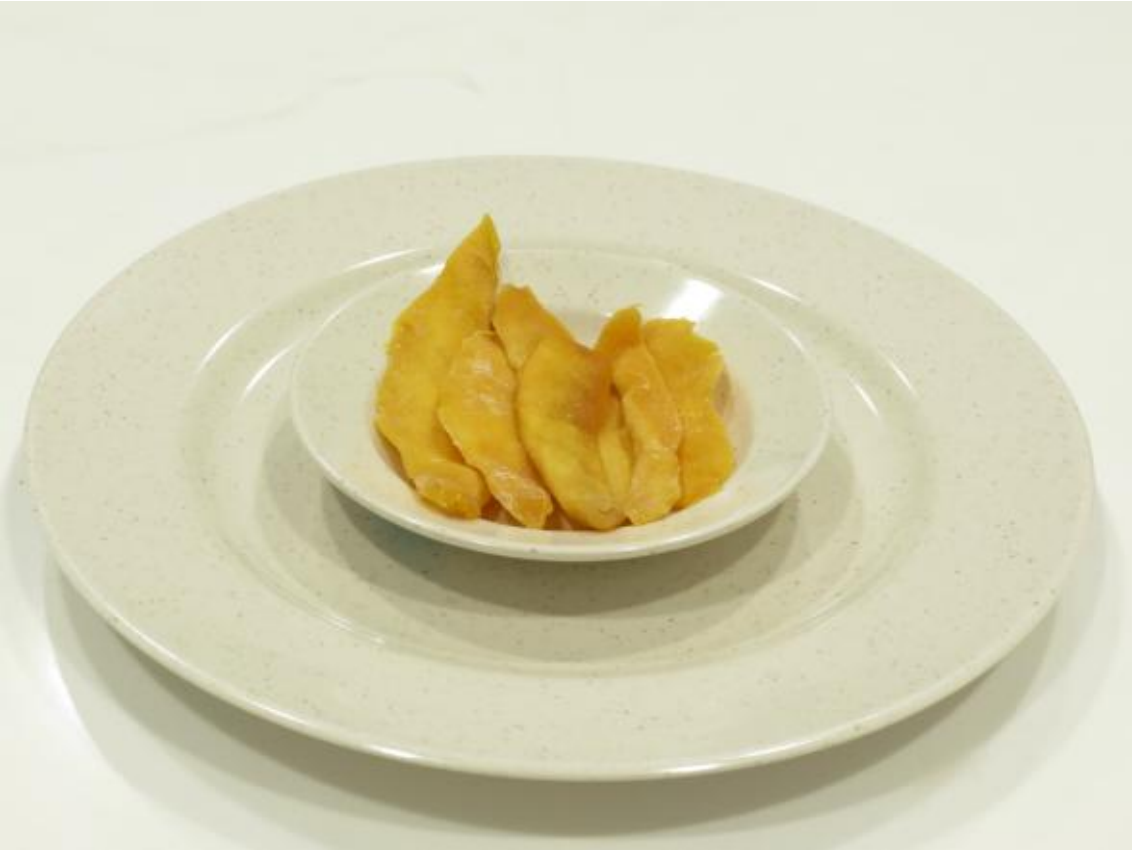

Mango, sliced (FA0052)

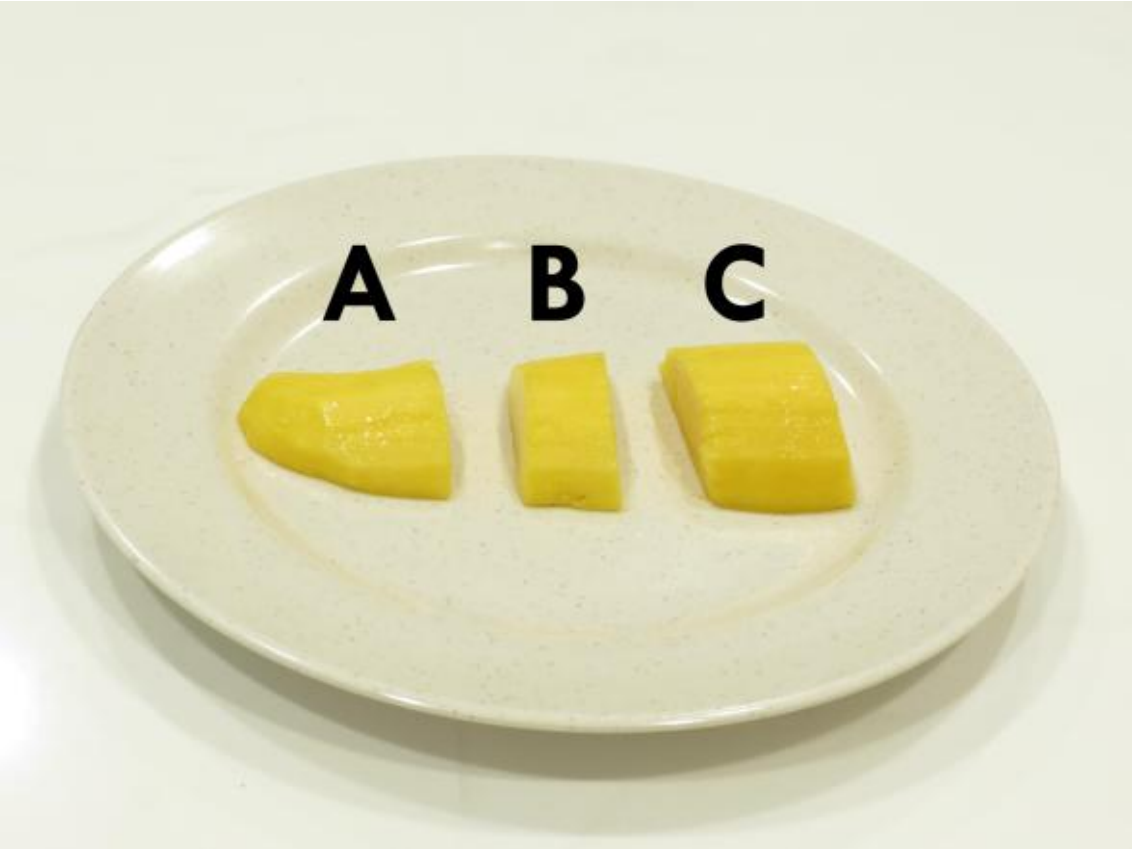

Orange, regular (FA0048)

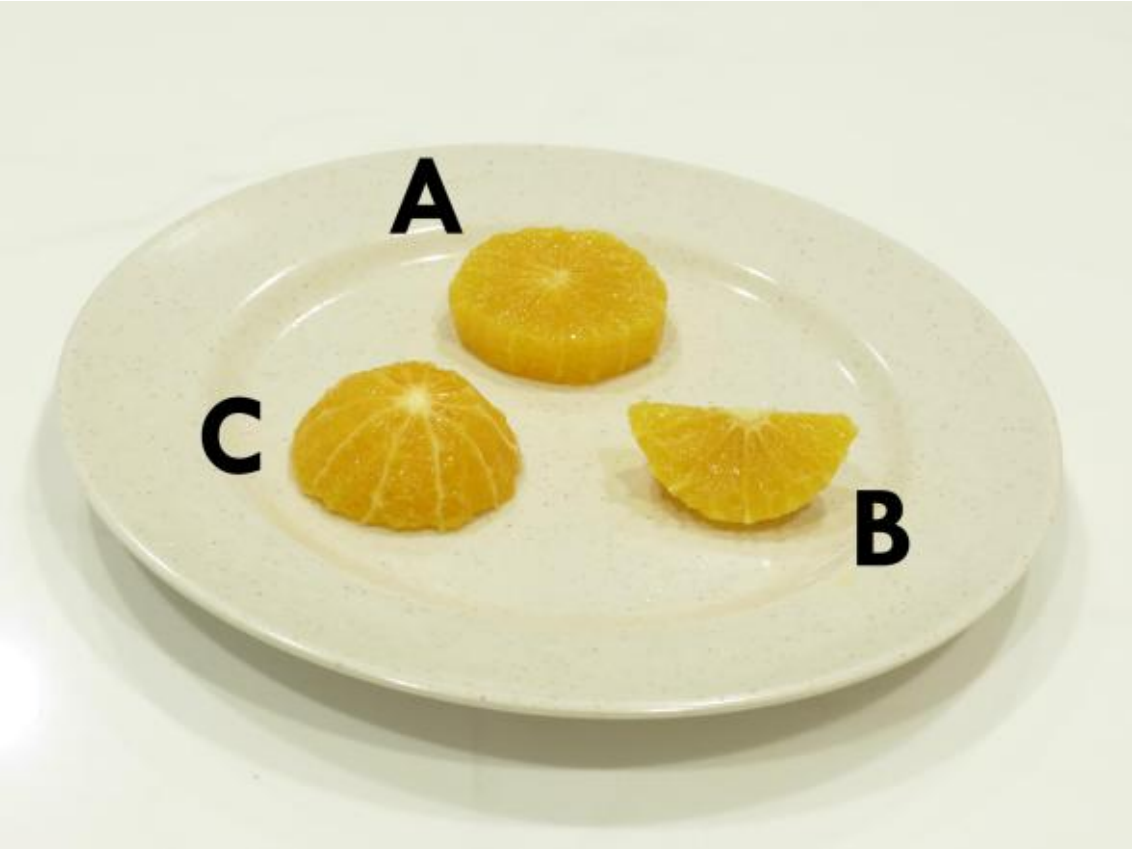

Orange, small (tangerine) (FA0176)

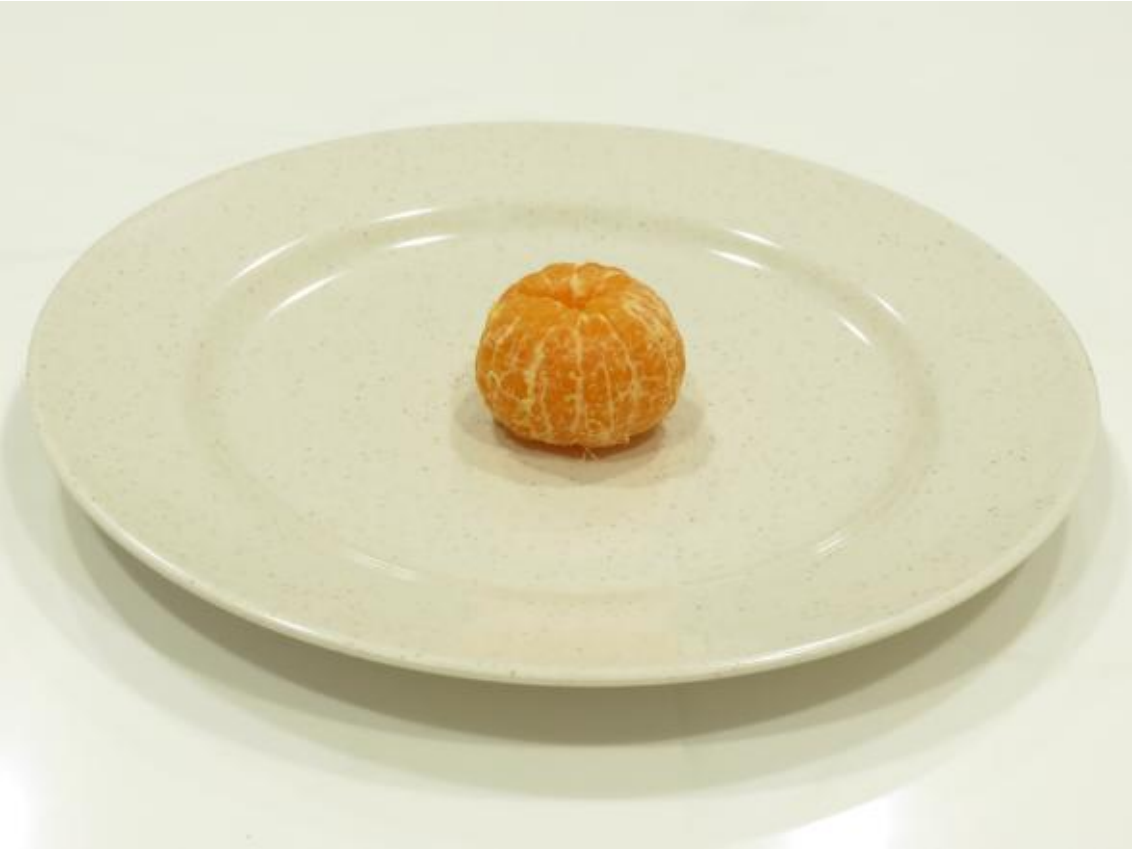

Pear, golden, wedge (FA0051)

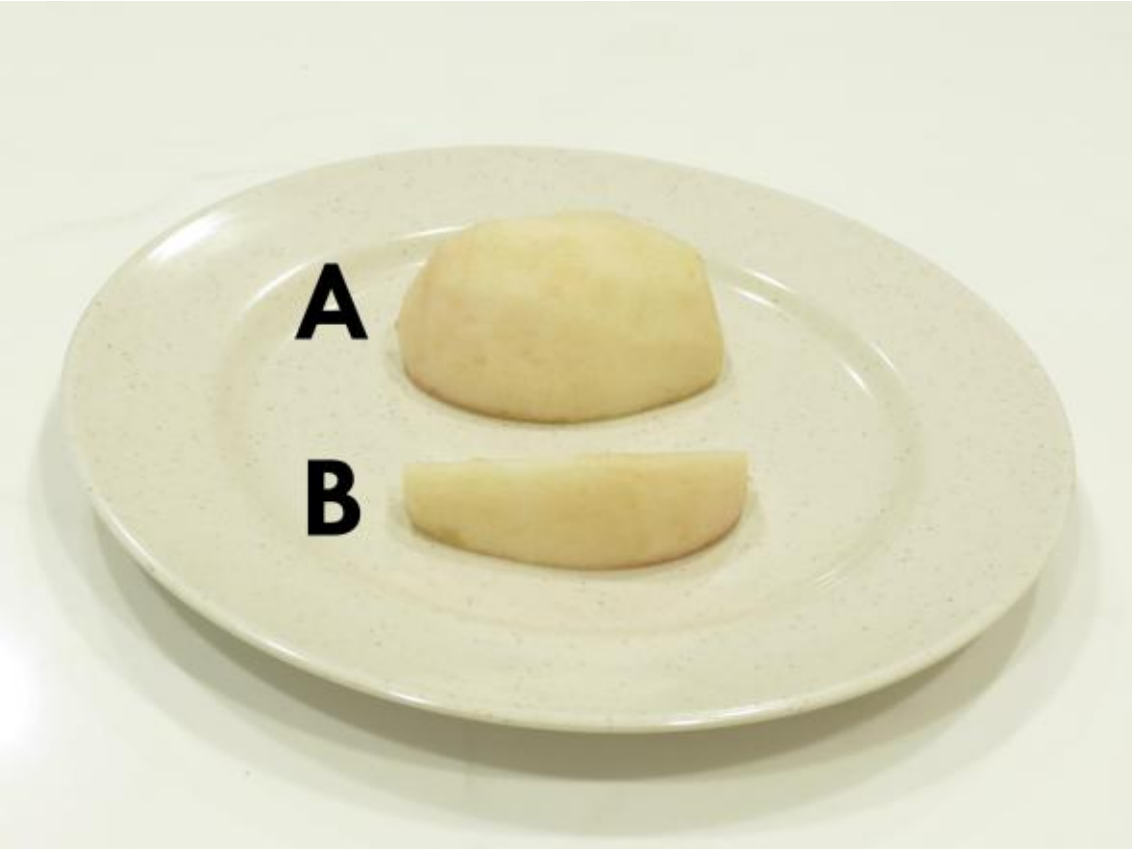

Pear, green, wedge (FA0175)

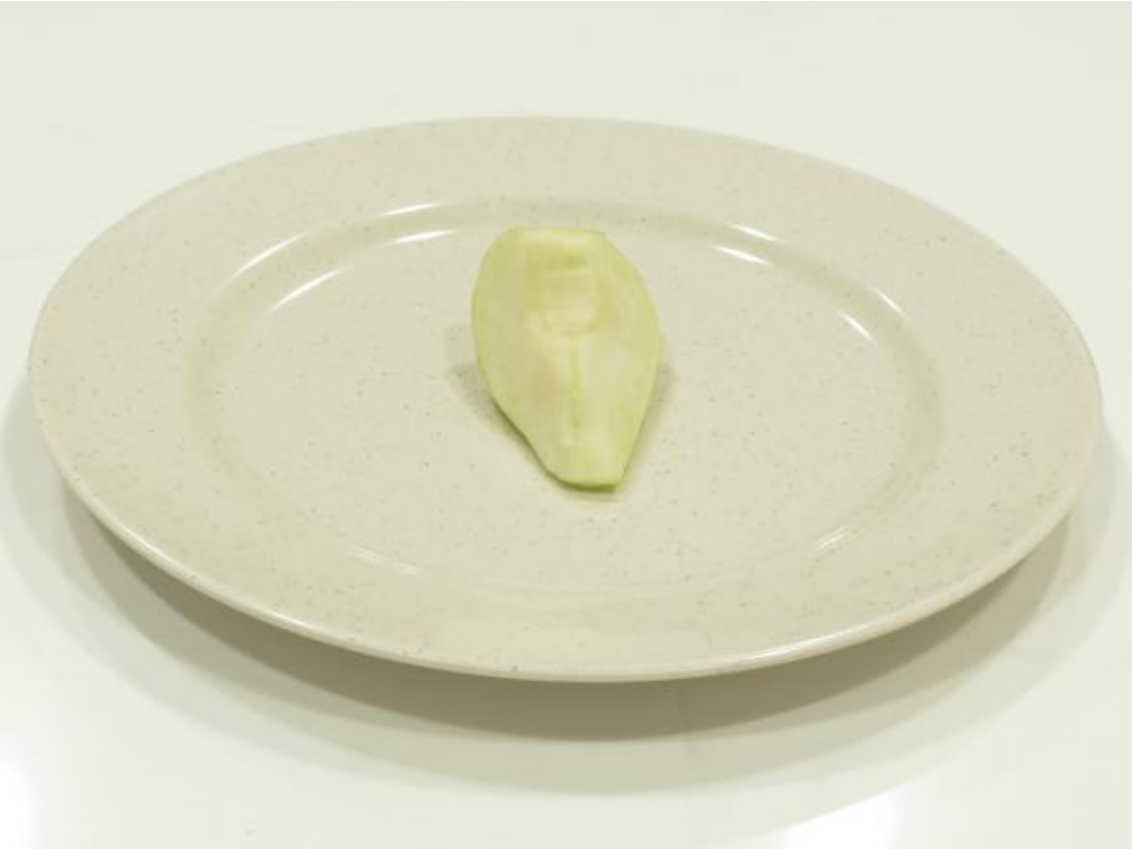

Pineapple (FA0047)

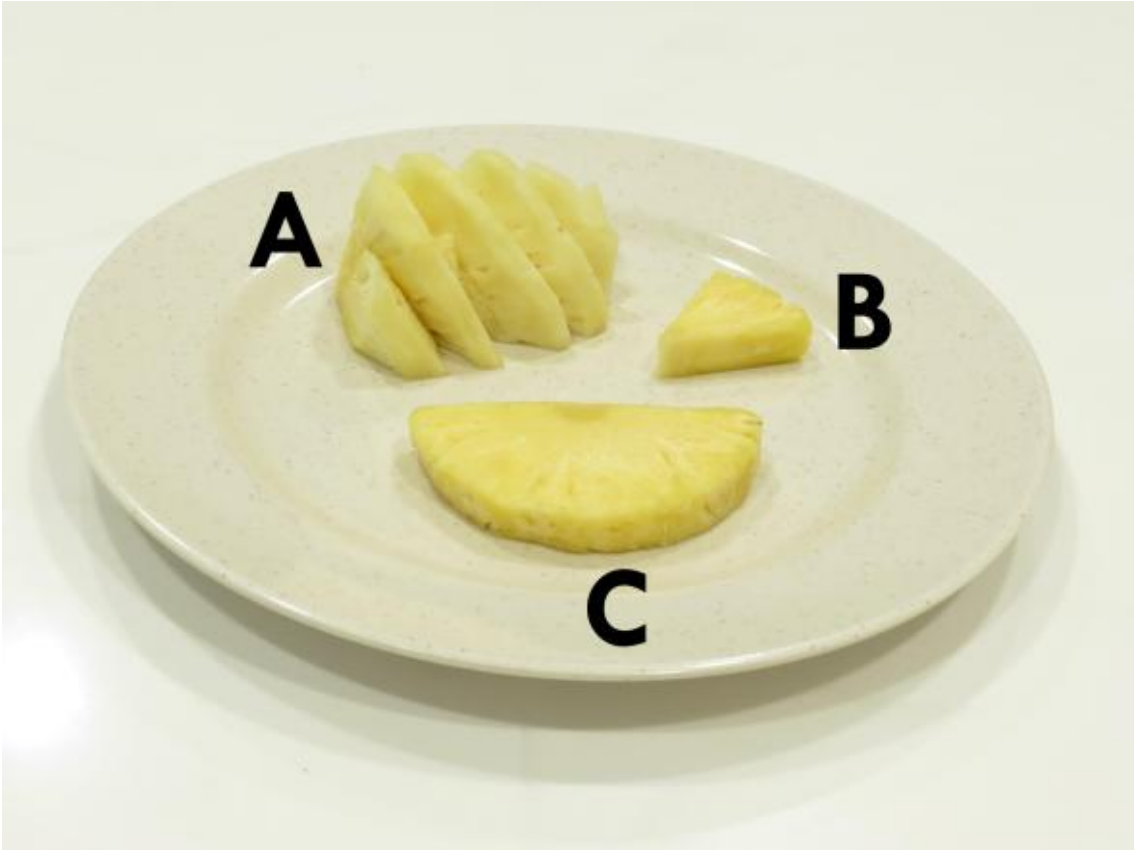

Pomelo (FA0053)

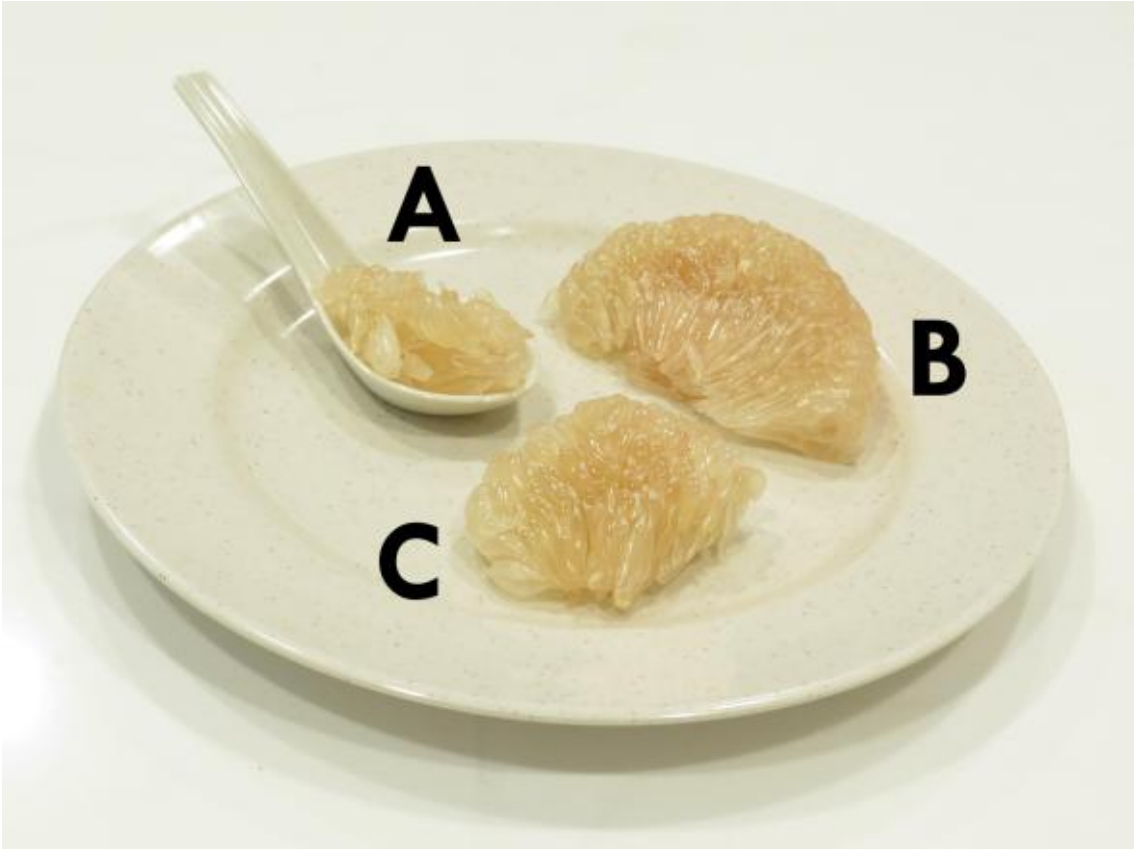

Watermelon, wedge (FA0186)

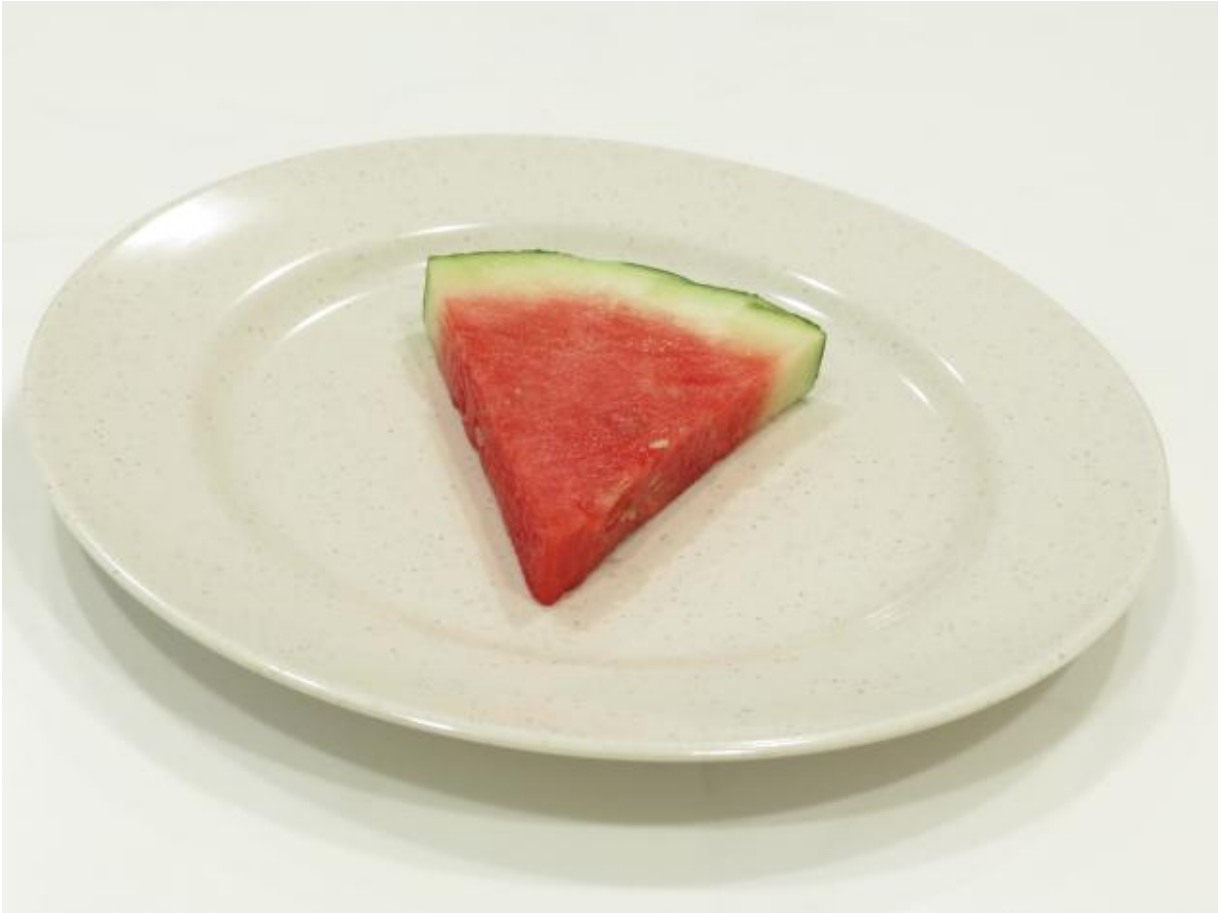

Fungus, black (FA0196)

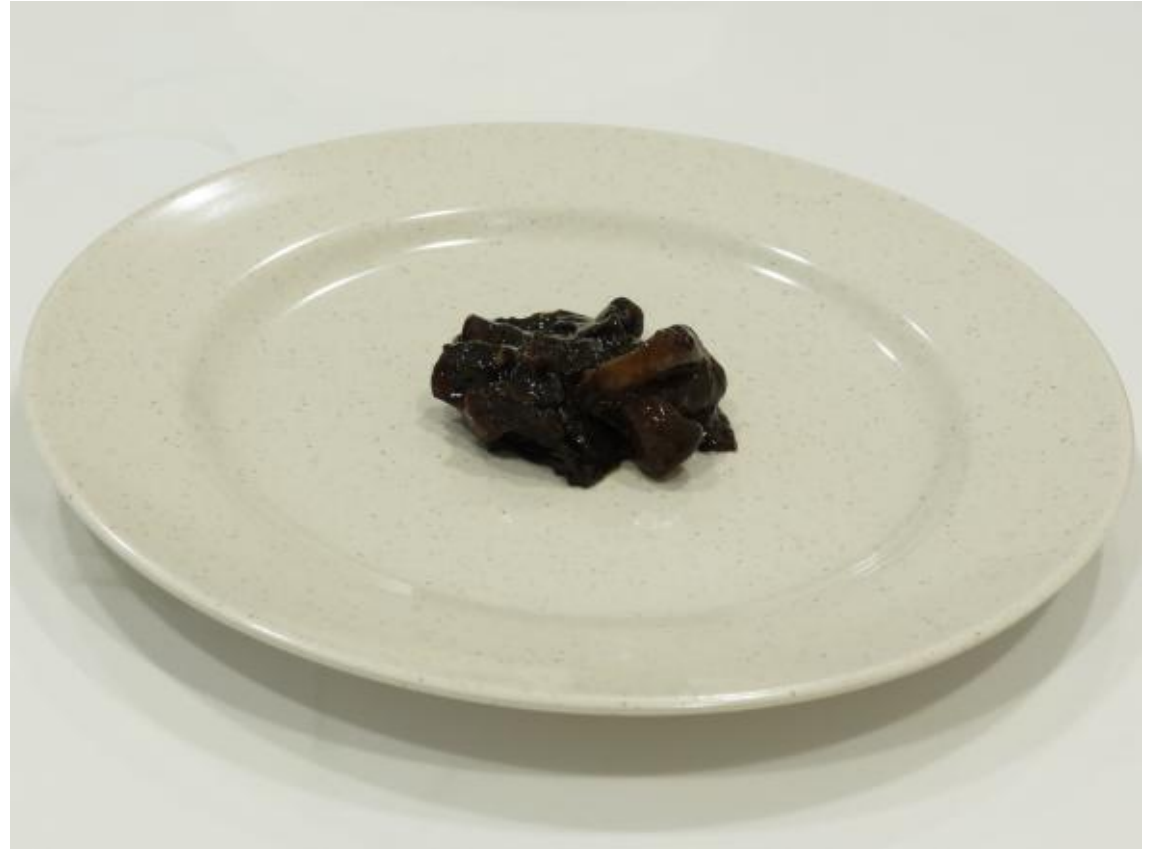

Mushroom, button, canned (FA0109)

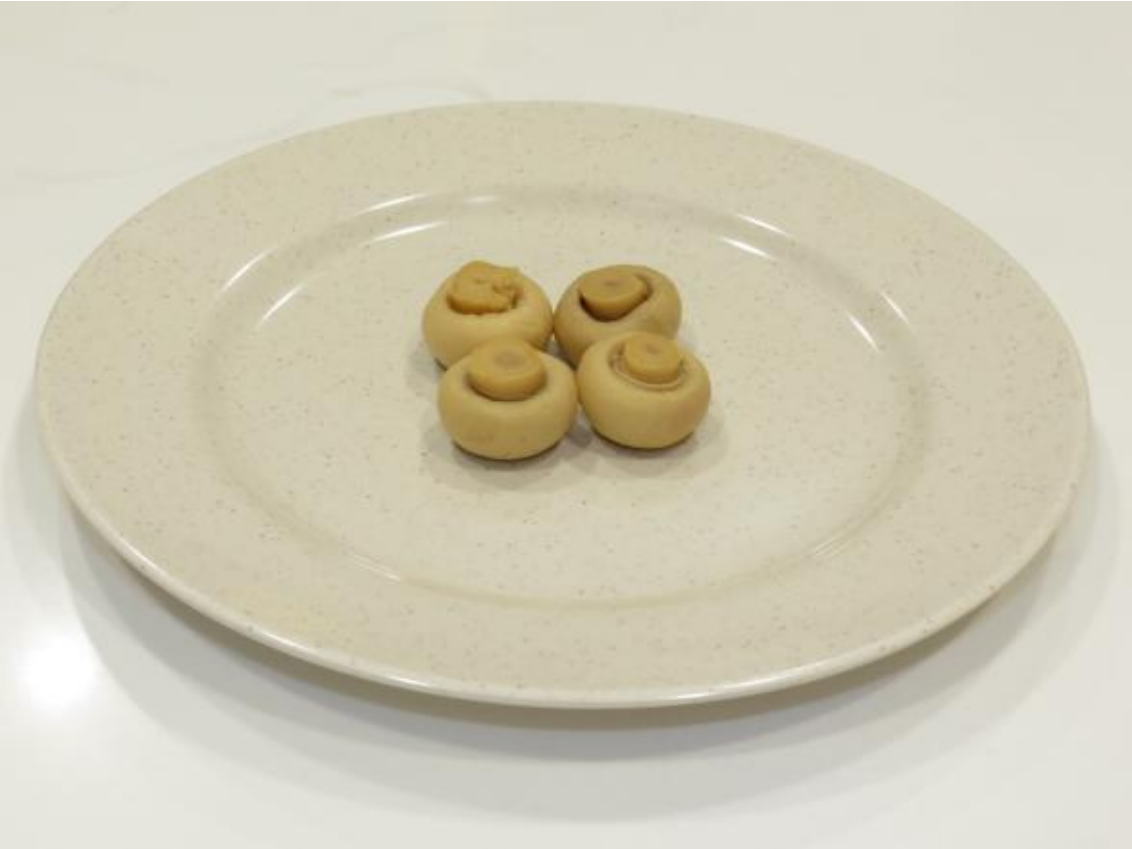

Mushroom, shiitake (FA0089)

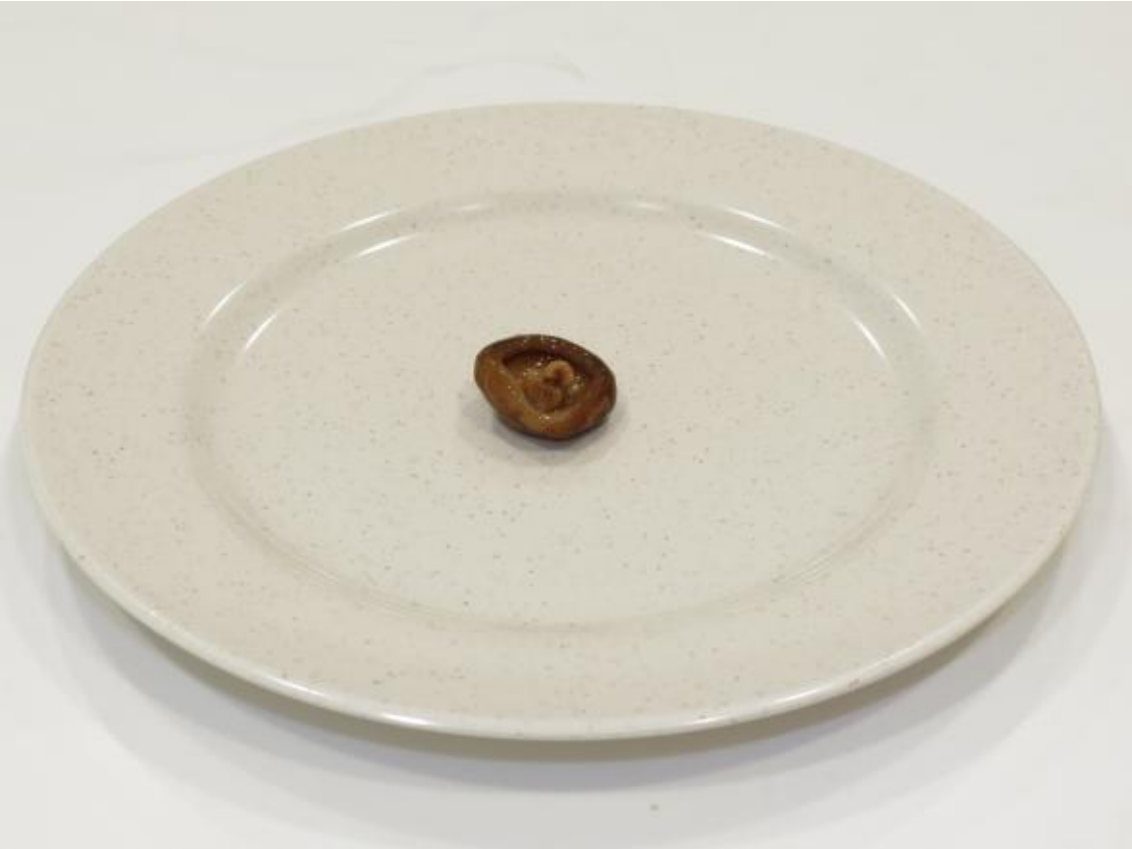

Rice dumpling, mini, nonya (FA0202)

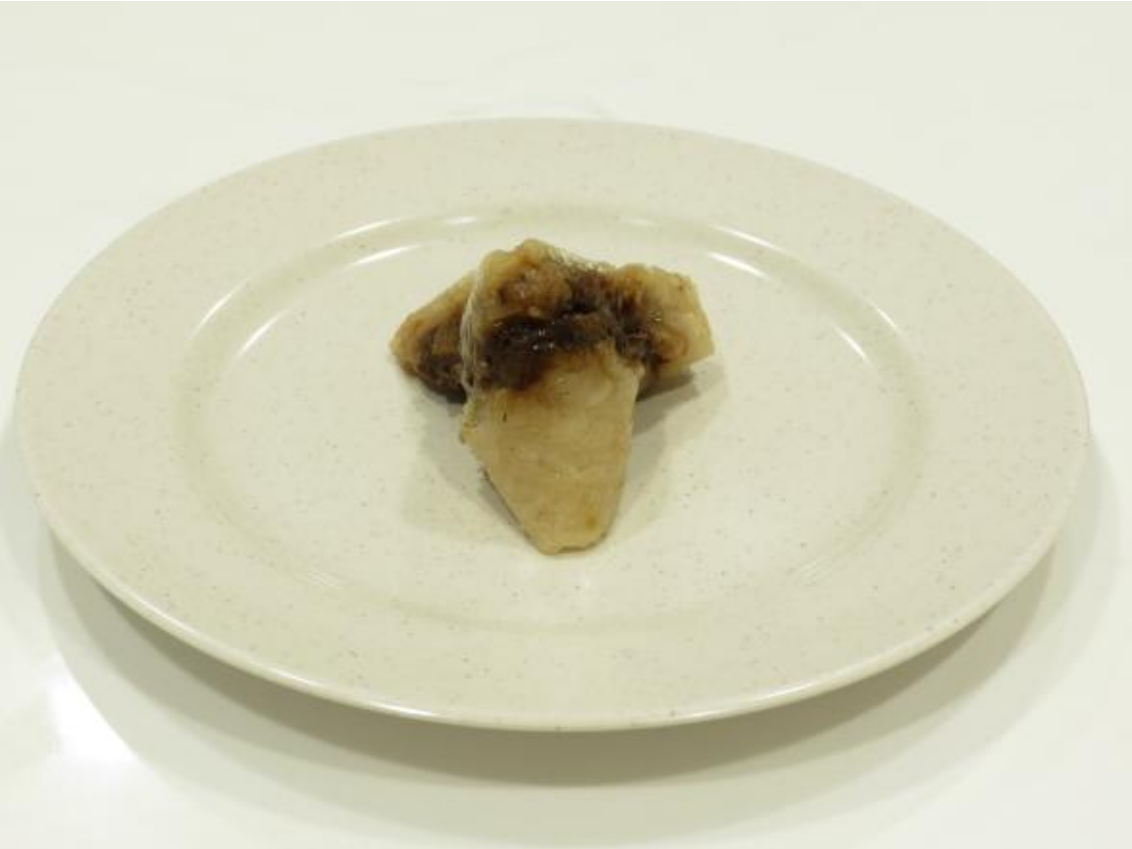

Rice dumpling, regular, nonya (FA0100)

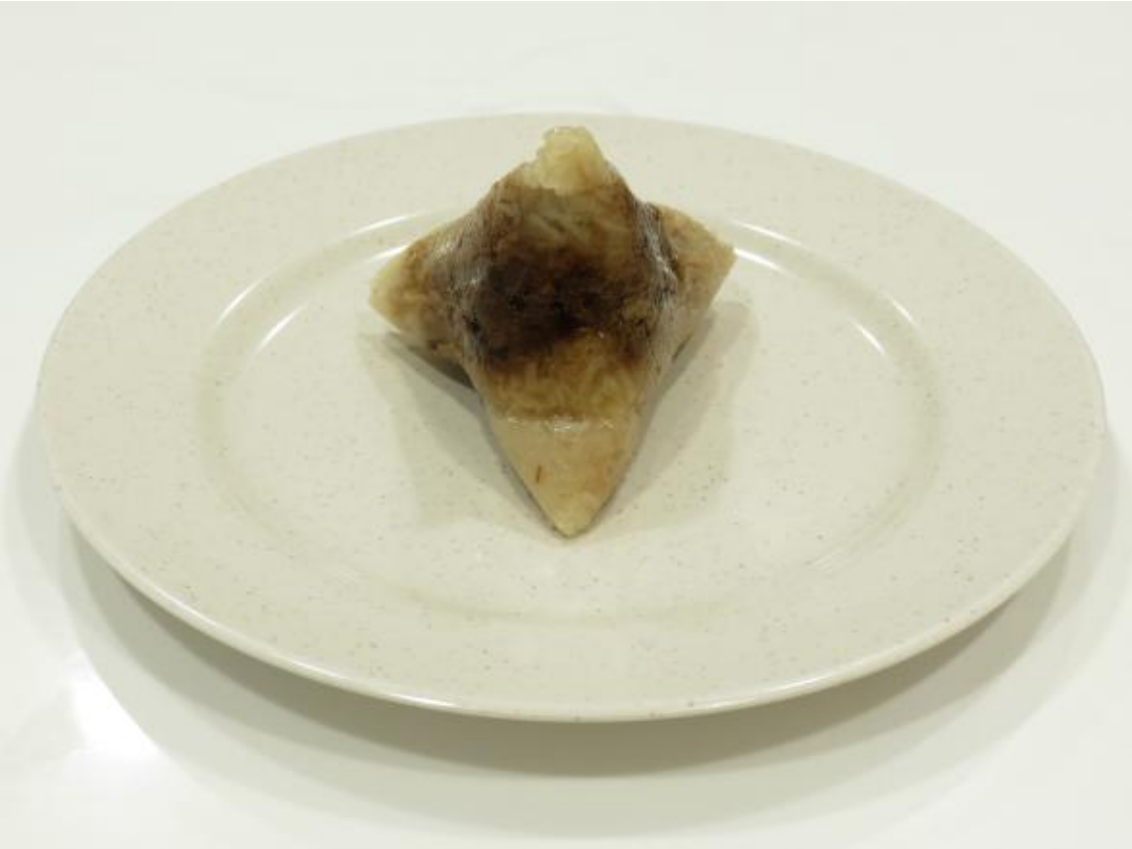

Rice dumpling, regular, hokkien (FA0203)

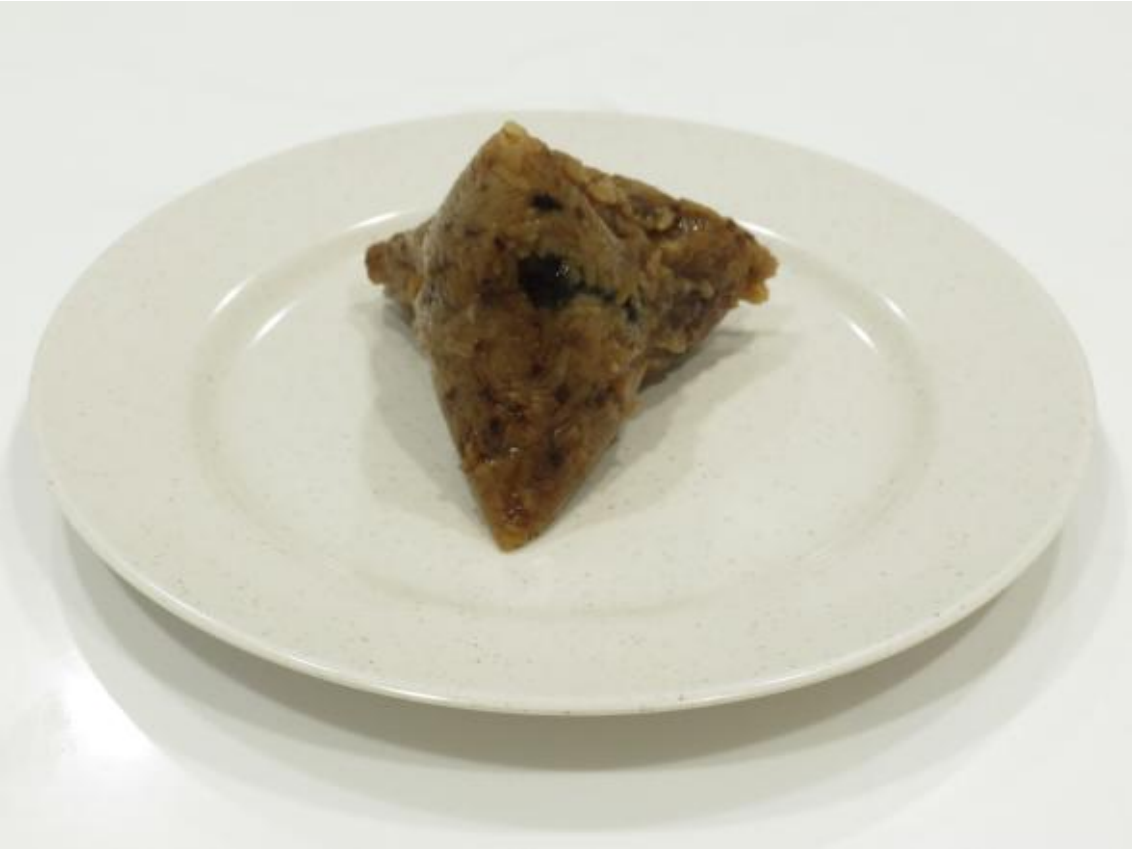

Rice noodle, bee hoon, thin, cooked (FA0185)

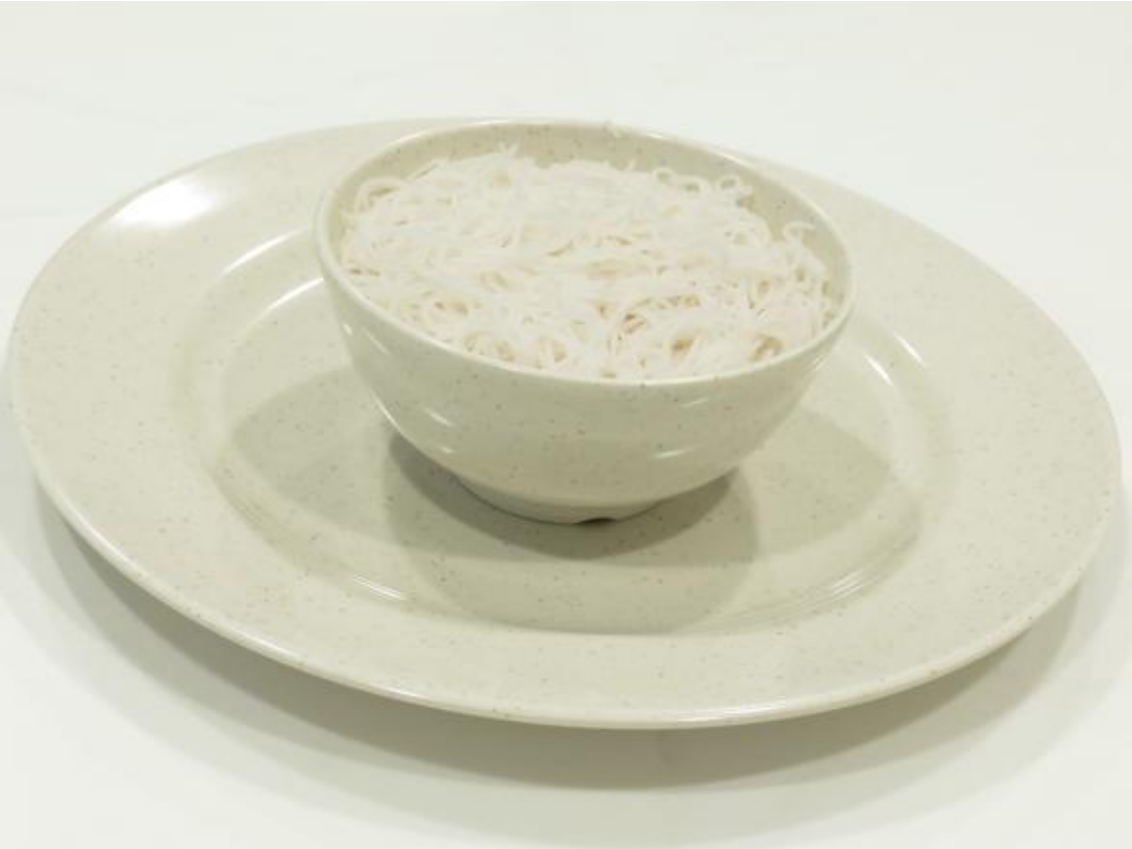

Rice noodle, kway teow, cooked (FA0117)

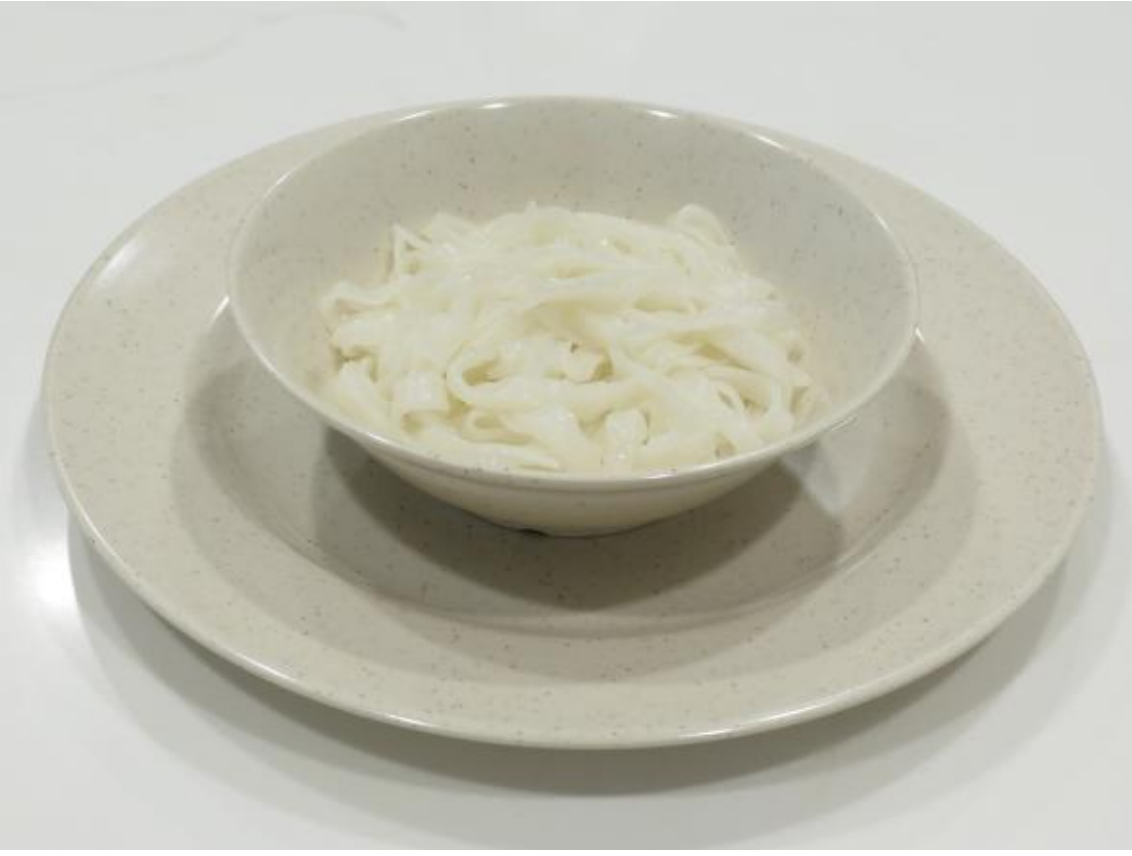

Bacon (FA0137)

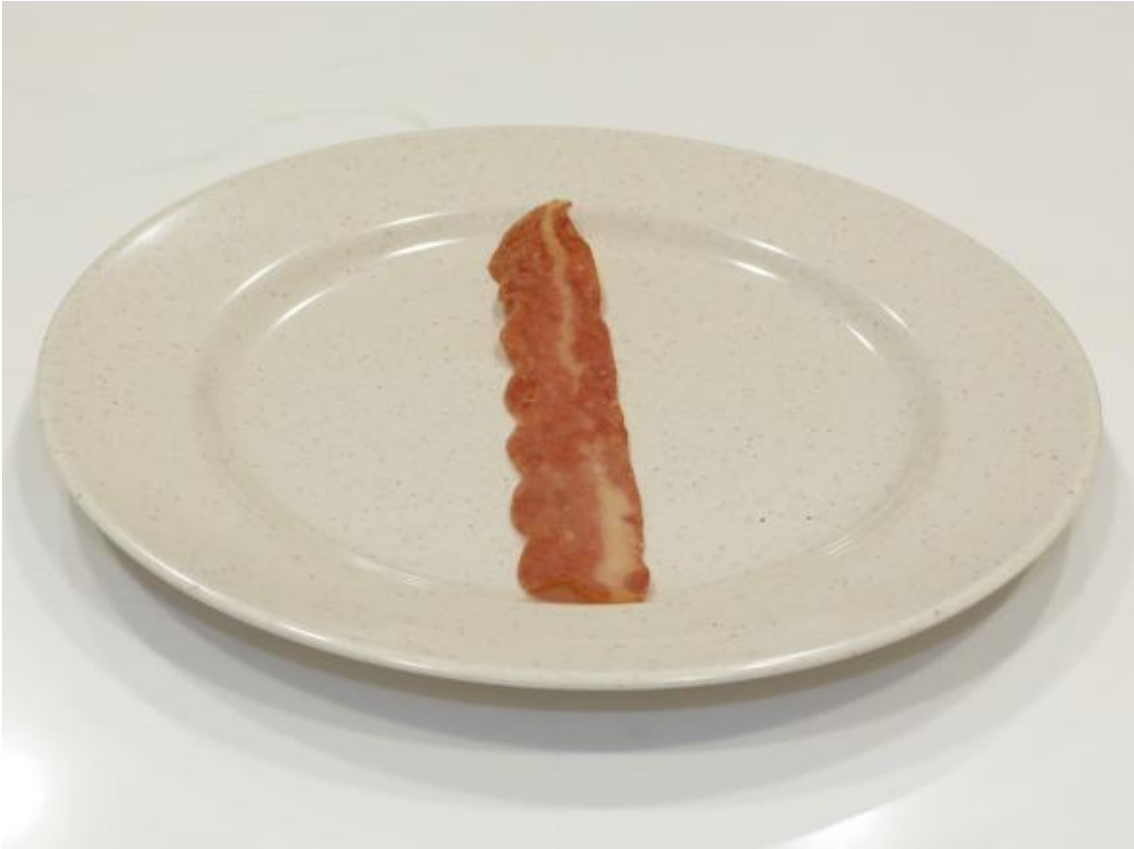

Chicken, breast, fillet, boneless (FA0134)

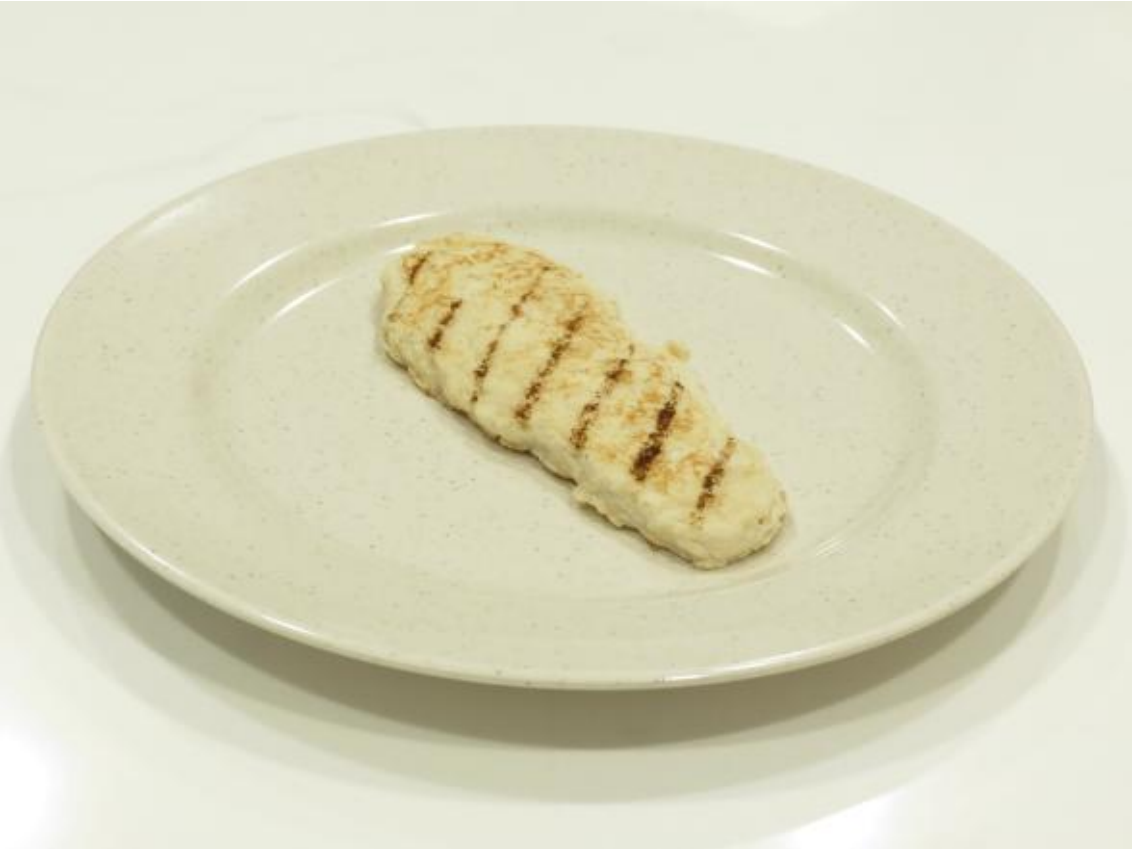

Chicken, breast, whole (FA0183)

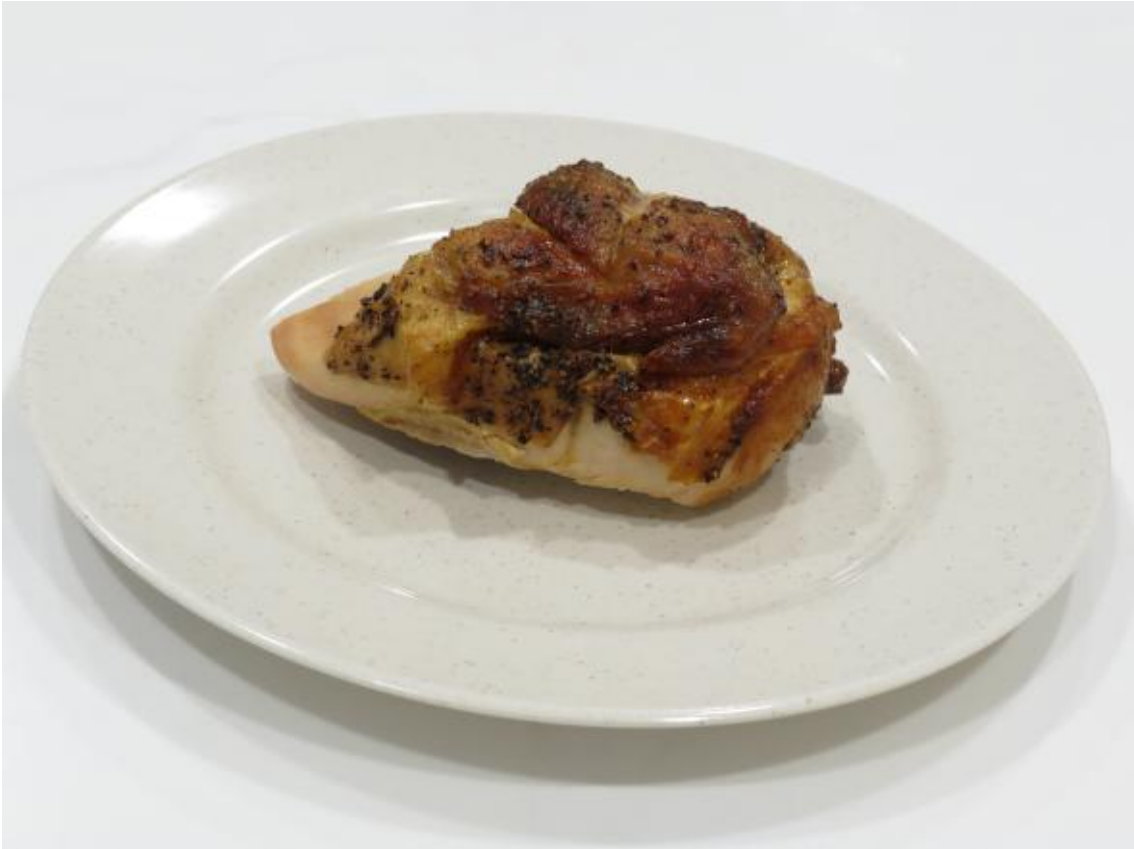

Chicken, chop, boneless (FA0165)

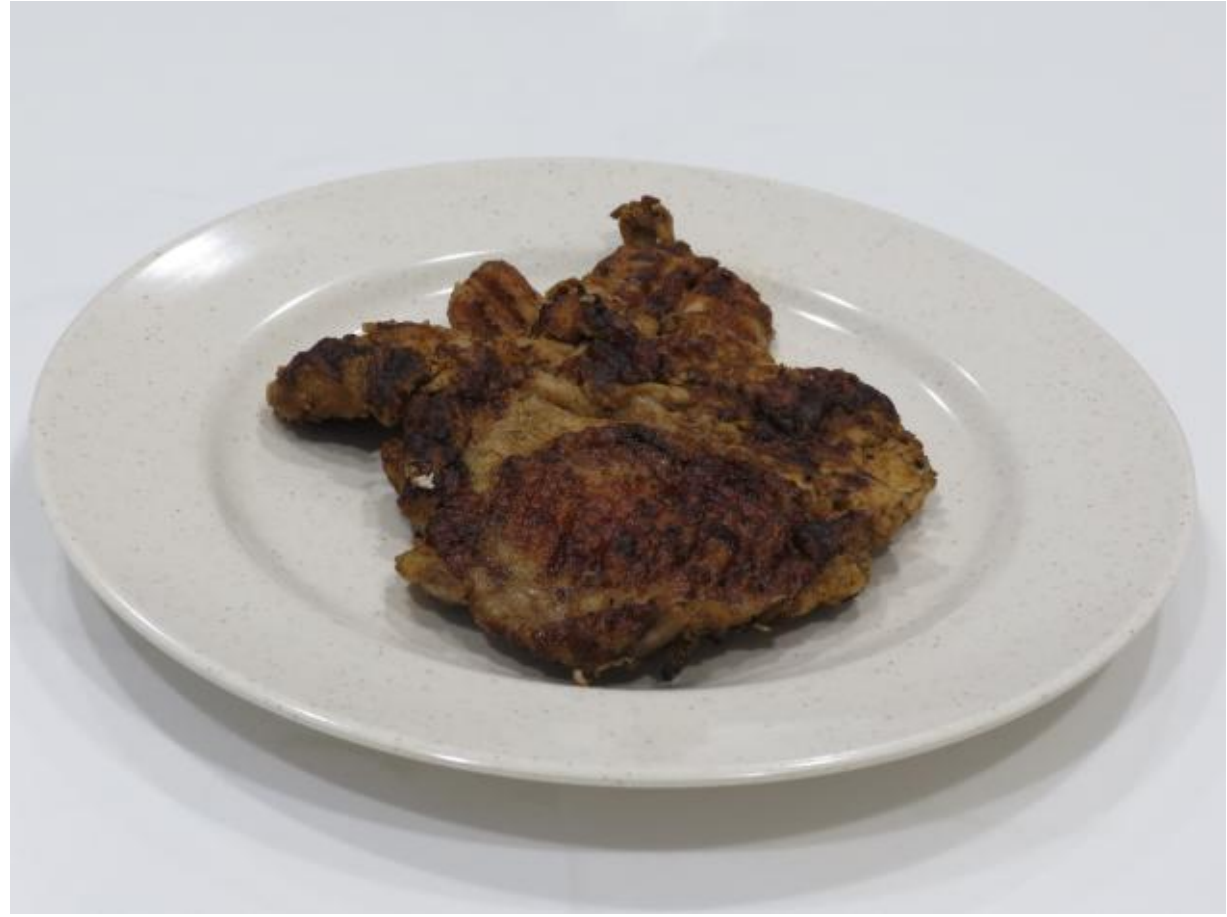

Chicken, drumstick, regular (FA0166)

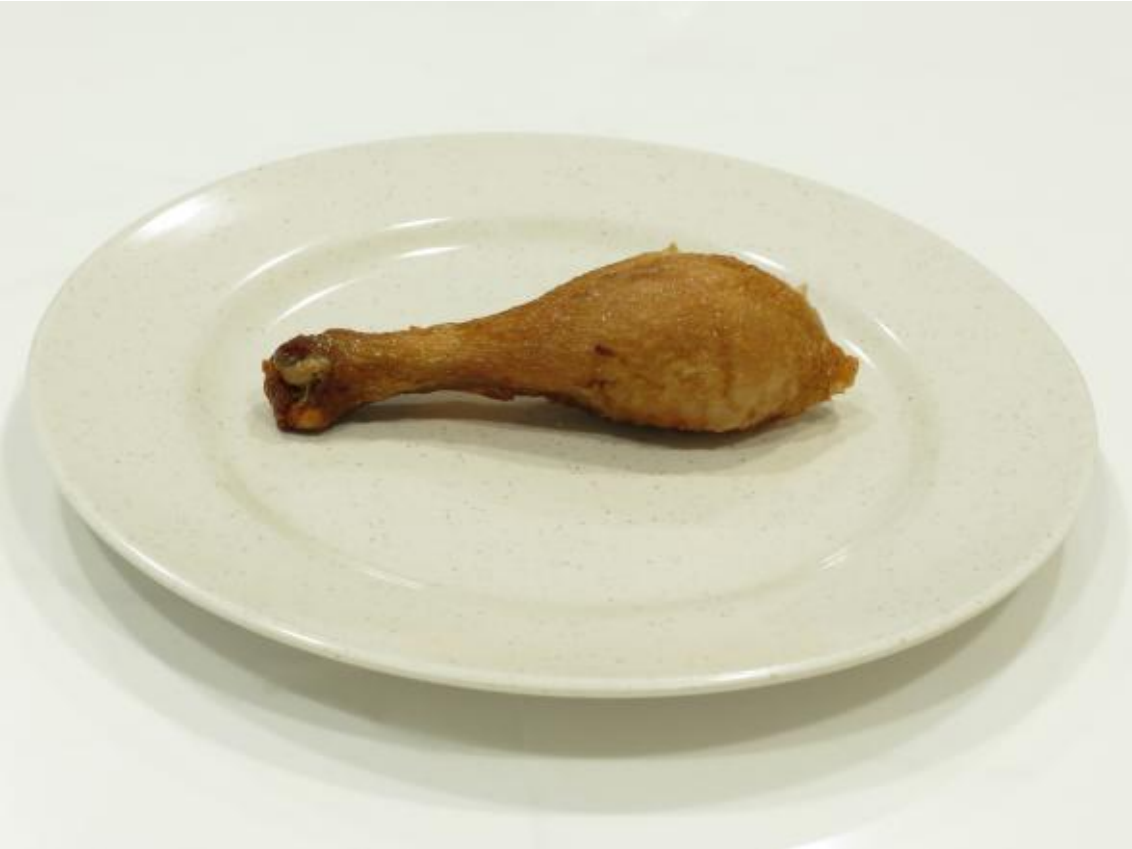

Chicken, drumstick, small (FA0158)

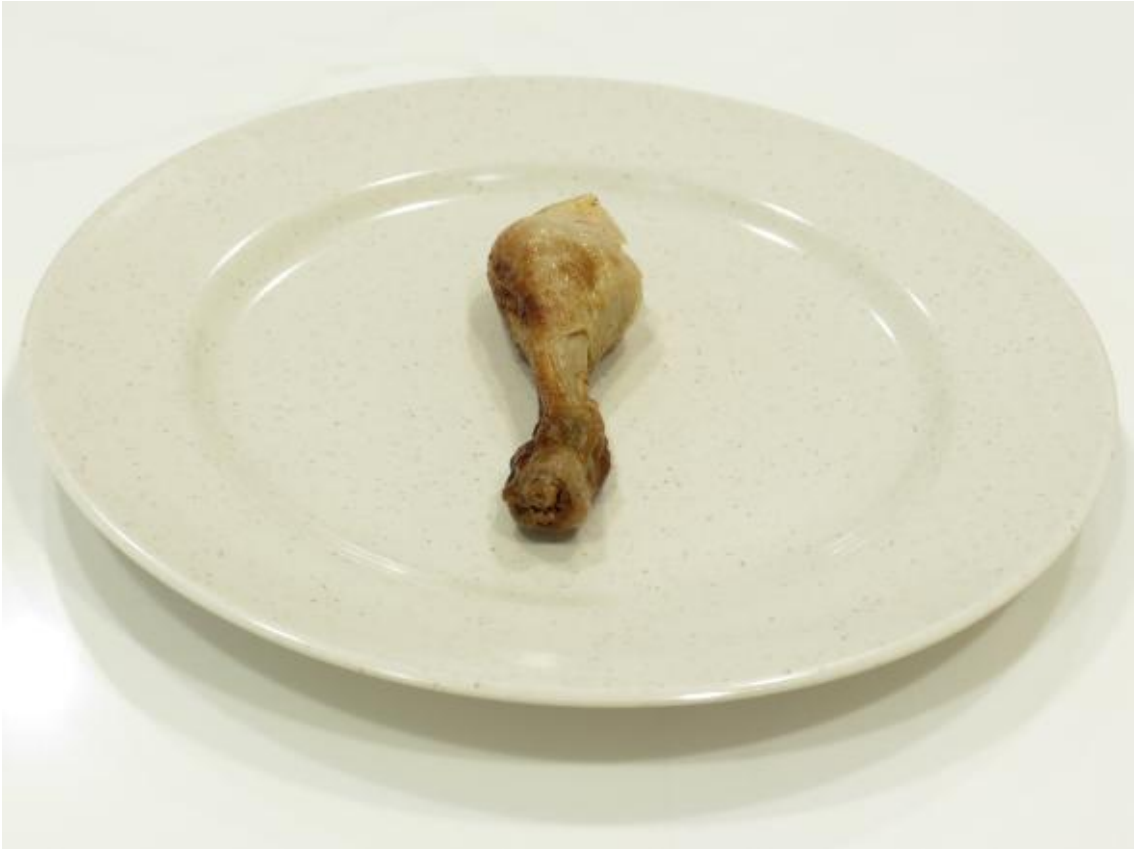

Chicken, leg, whole (FA0157)

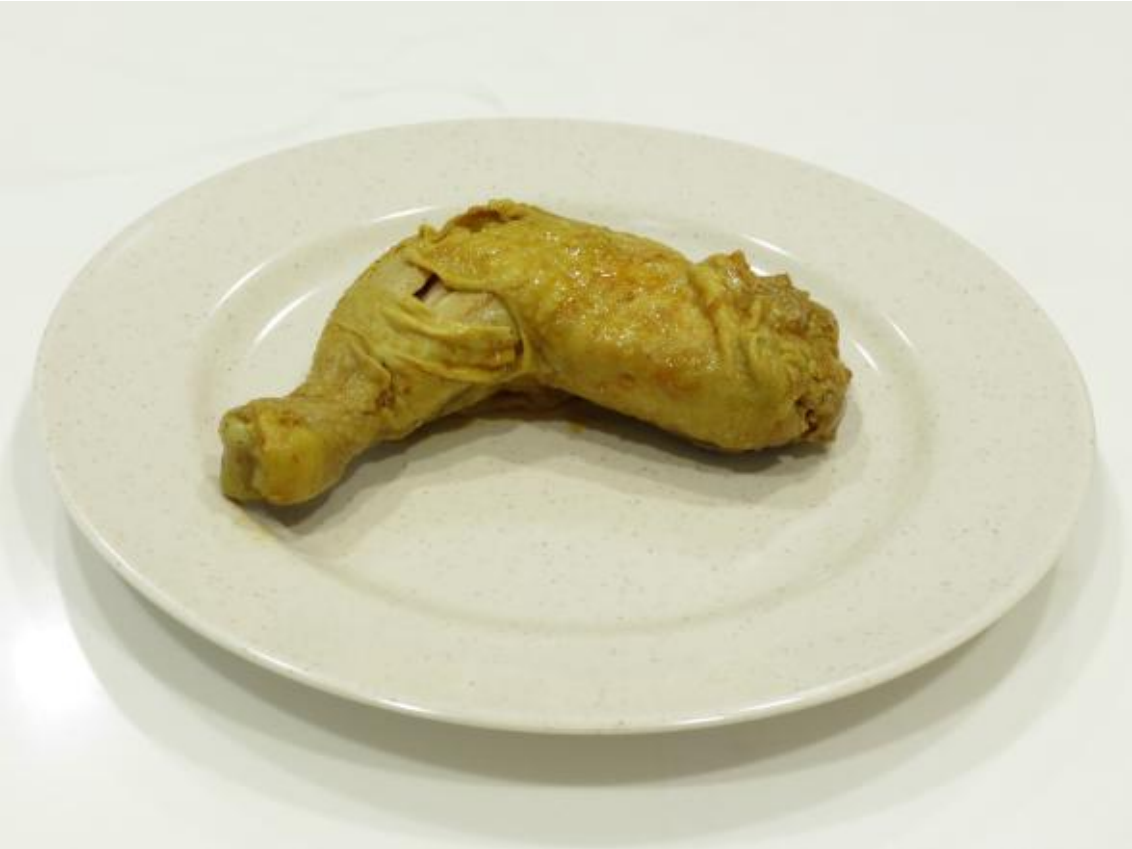

Chicken, thigh, fillet, boneless (FA0045)

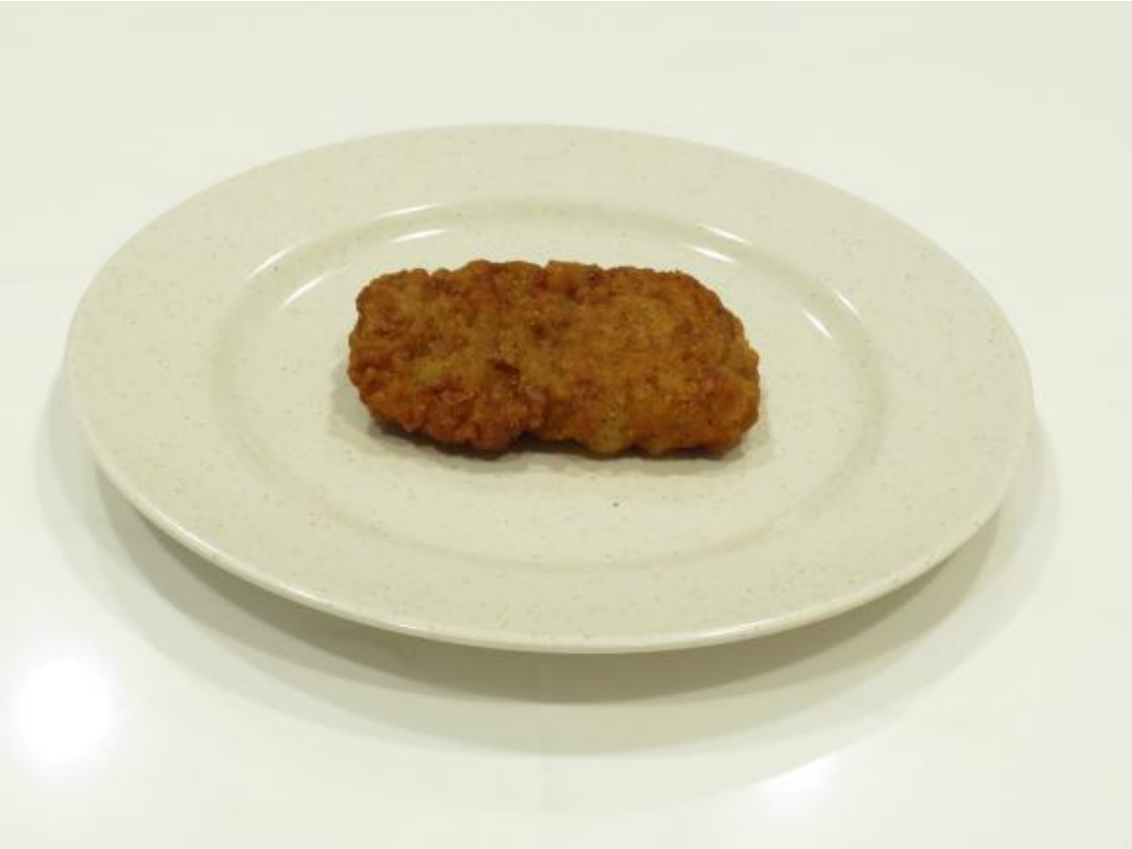

Chicken, mid-joint wing (FA0070)

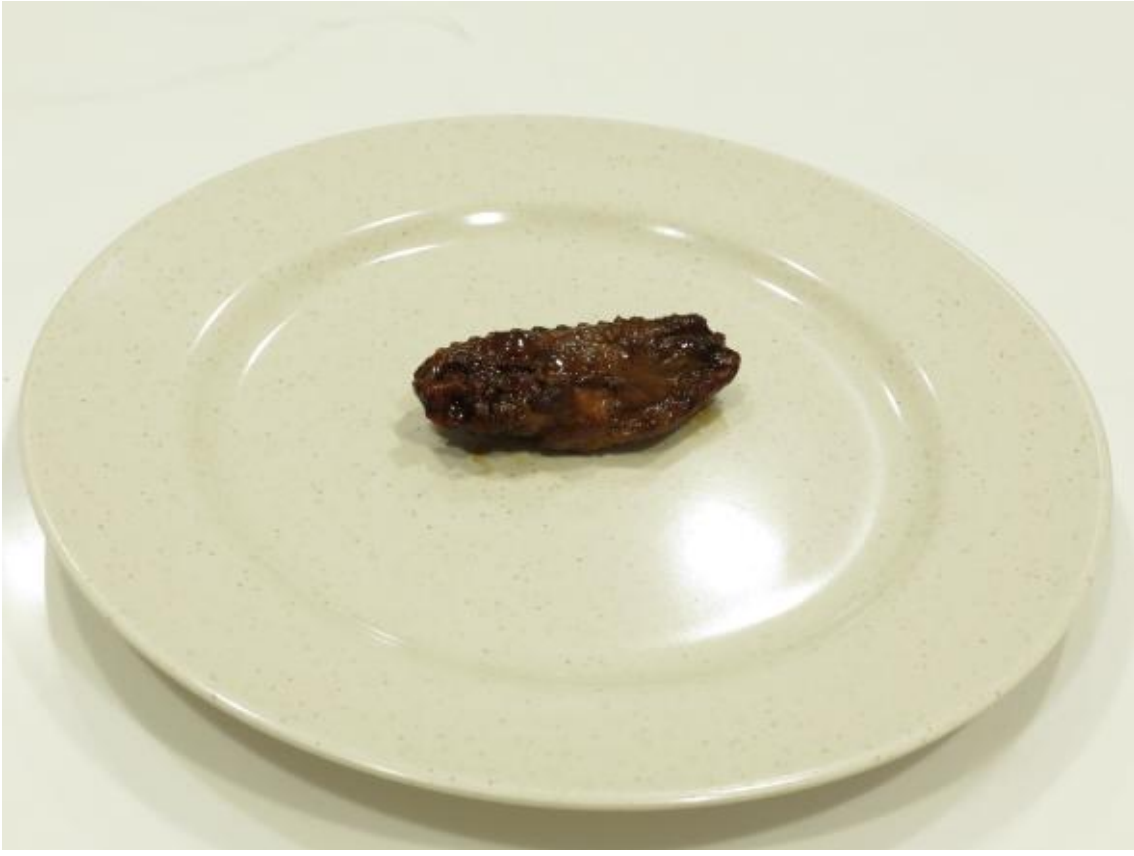

Chicken, wing (FA0167)

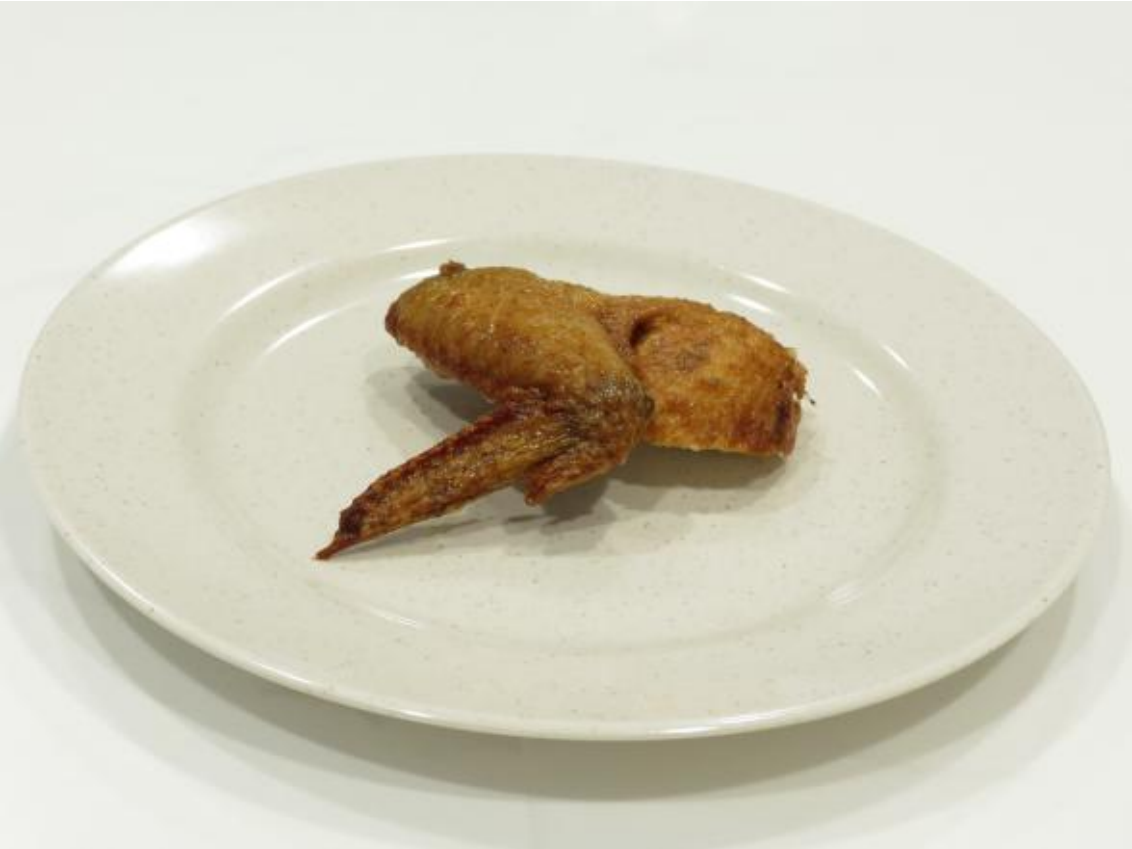

Chicken, spring (FA0156)

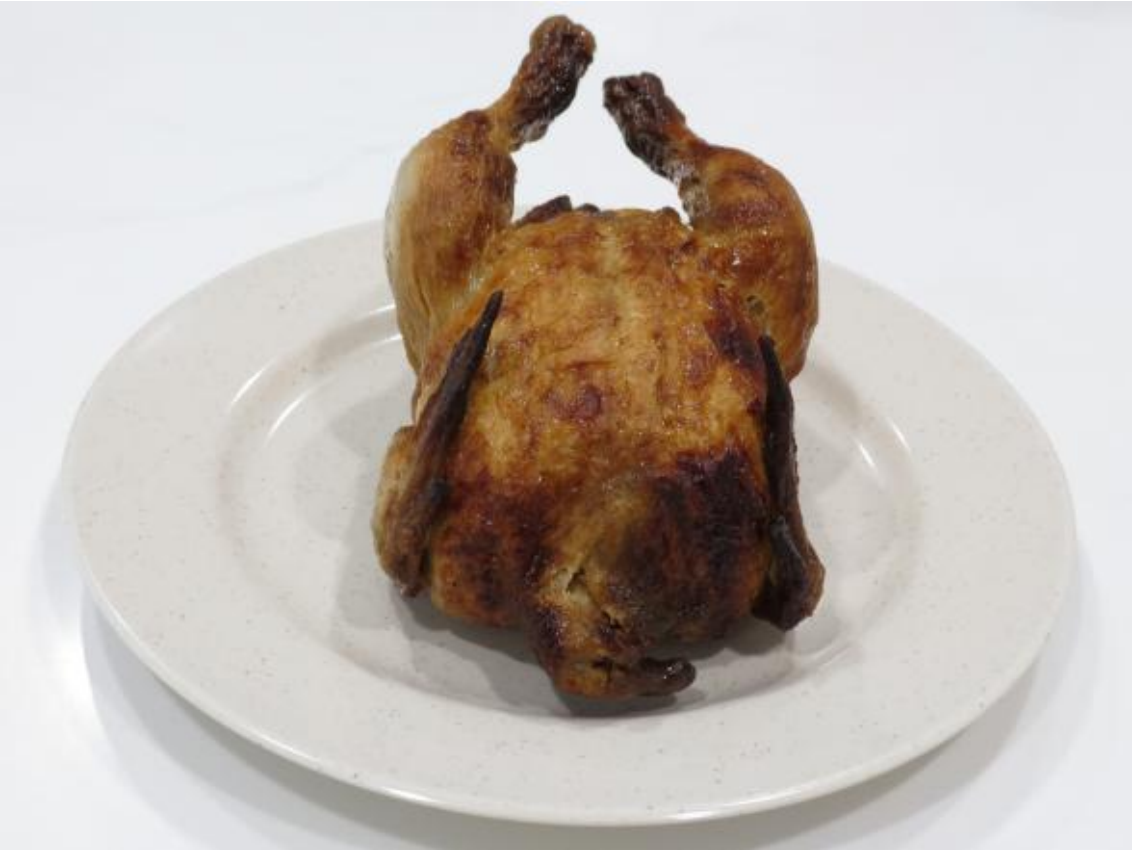

Luncheon meat (FA0055)

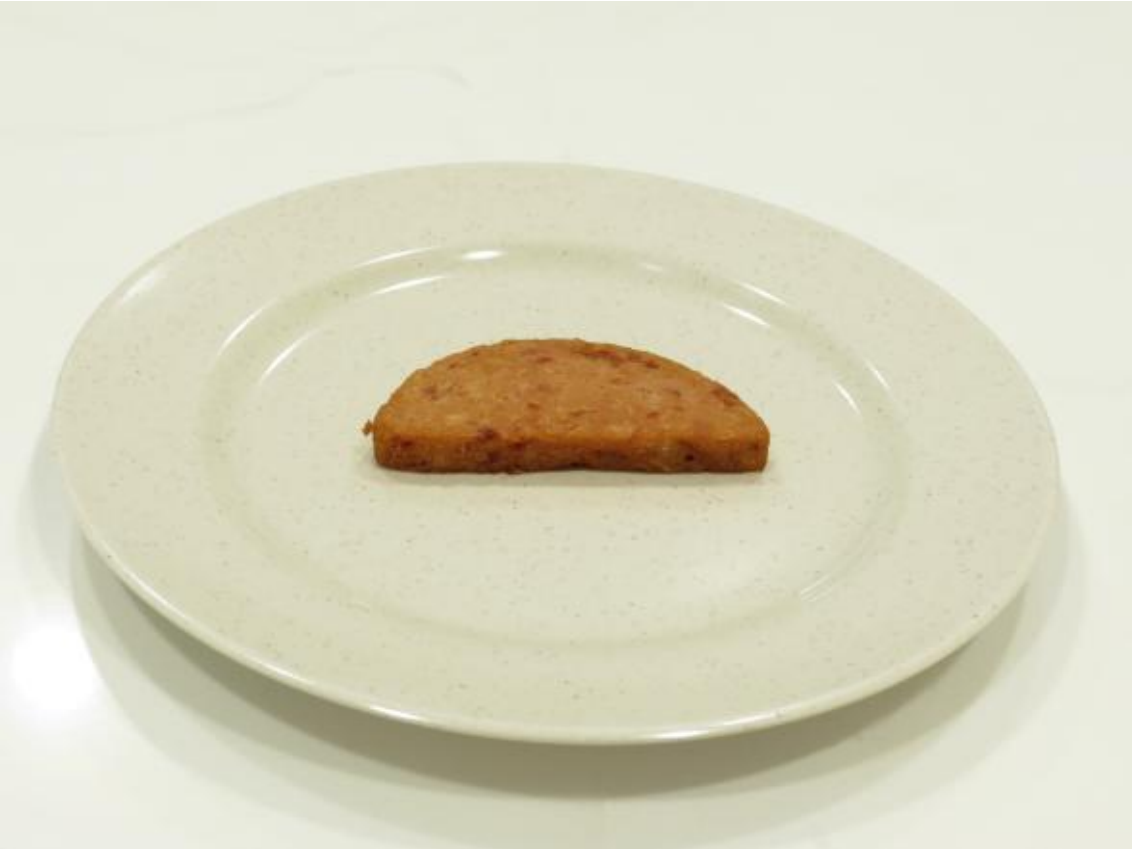

Meat floss (FA0018)

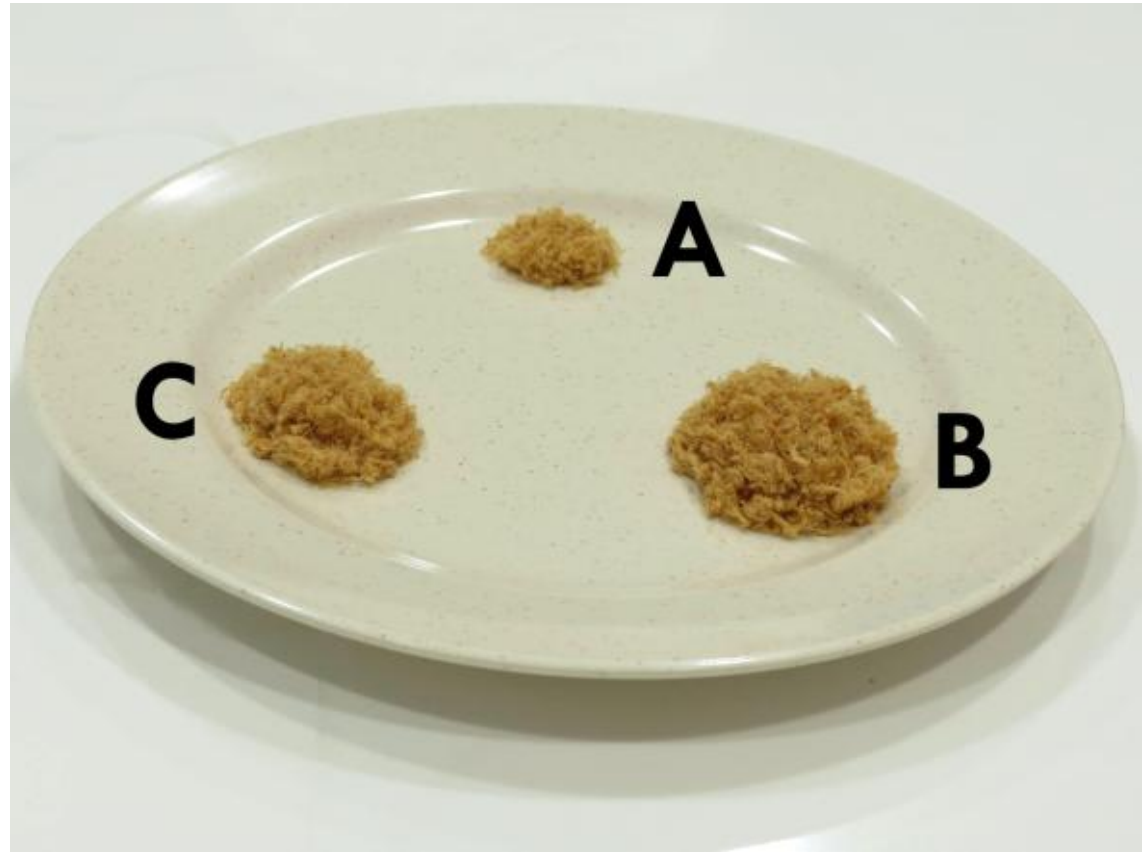

Meatball, handmade (FA0096)

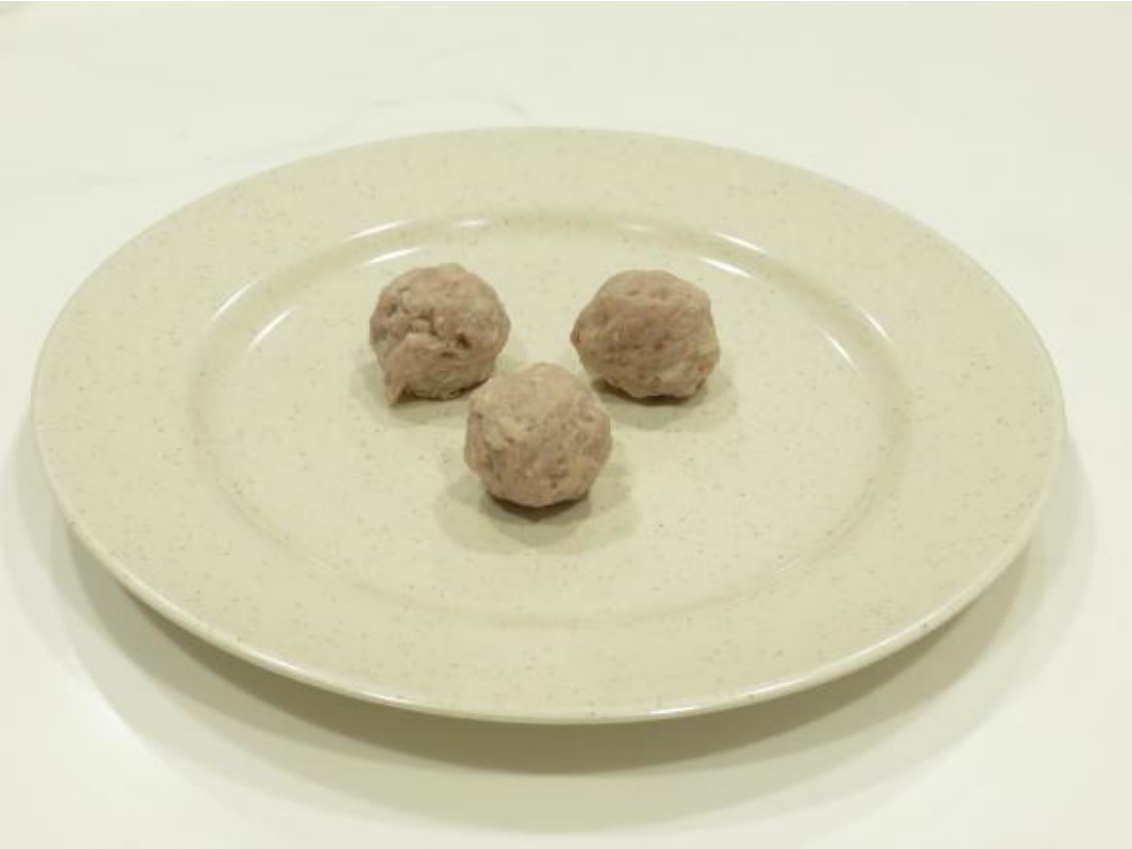

Meatball, processed (FA0118)

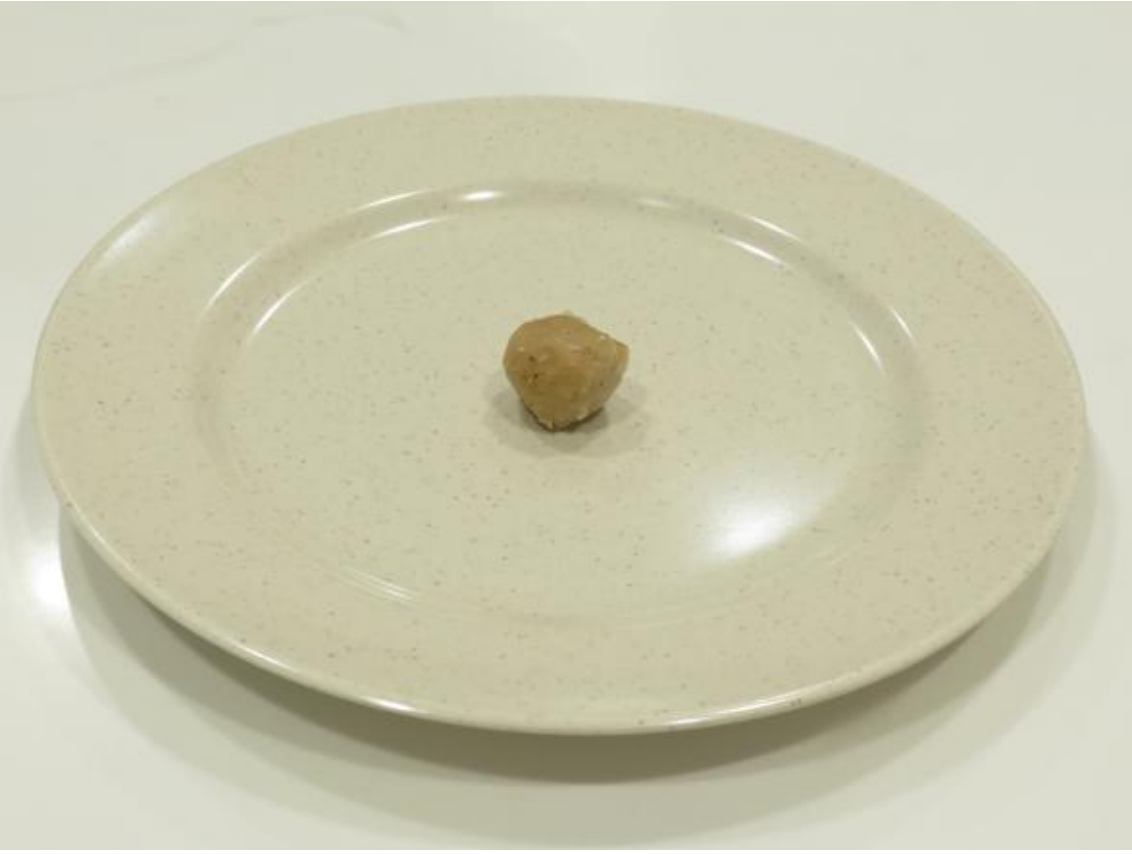

Pork belly (FA0125)

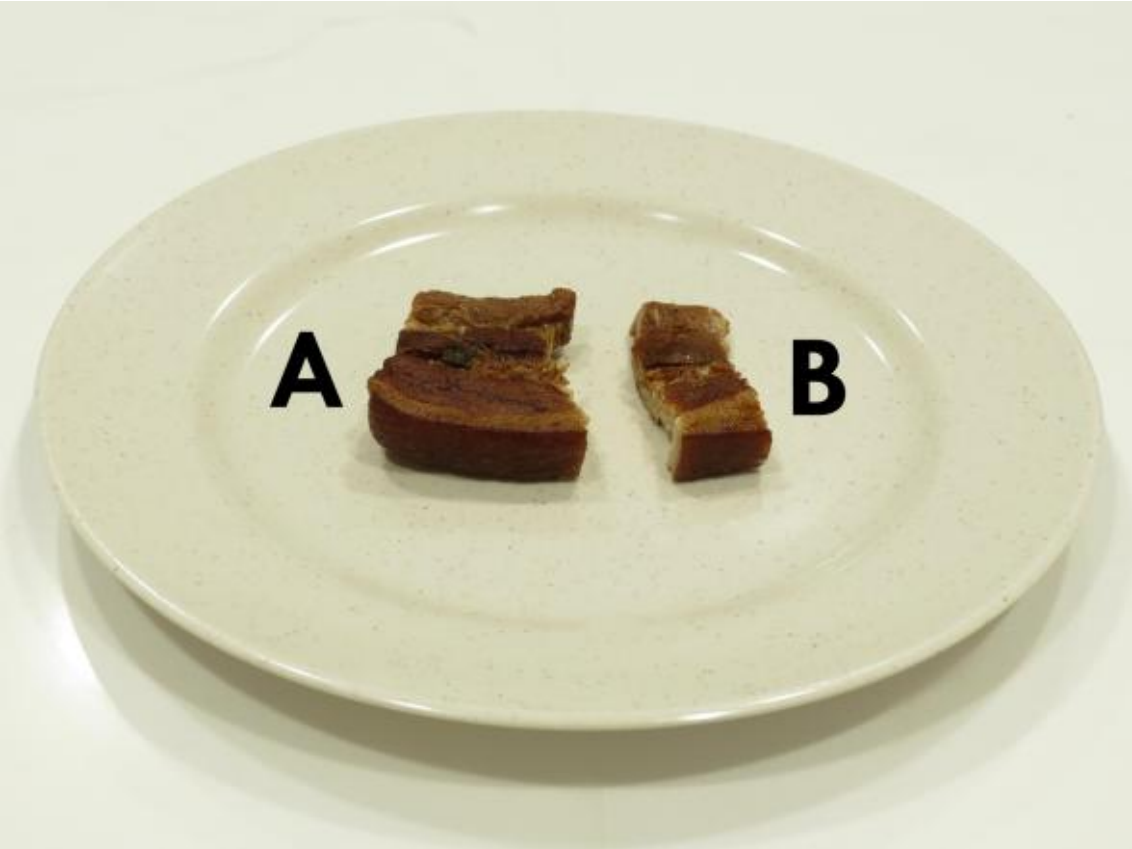

Pork rib, with bone (FA0141)

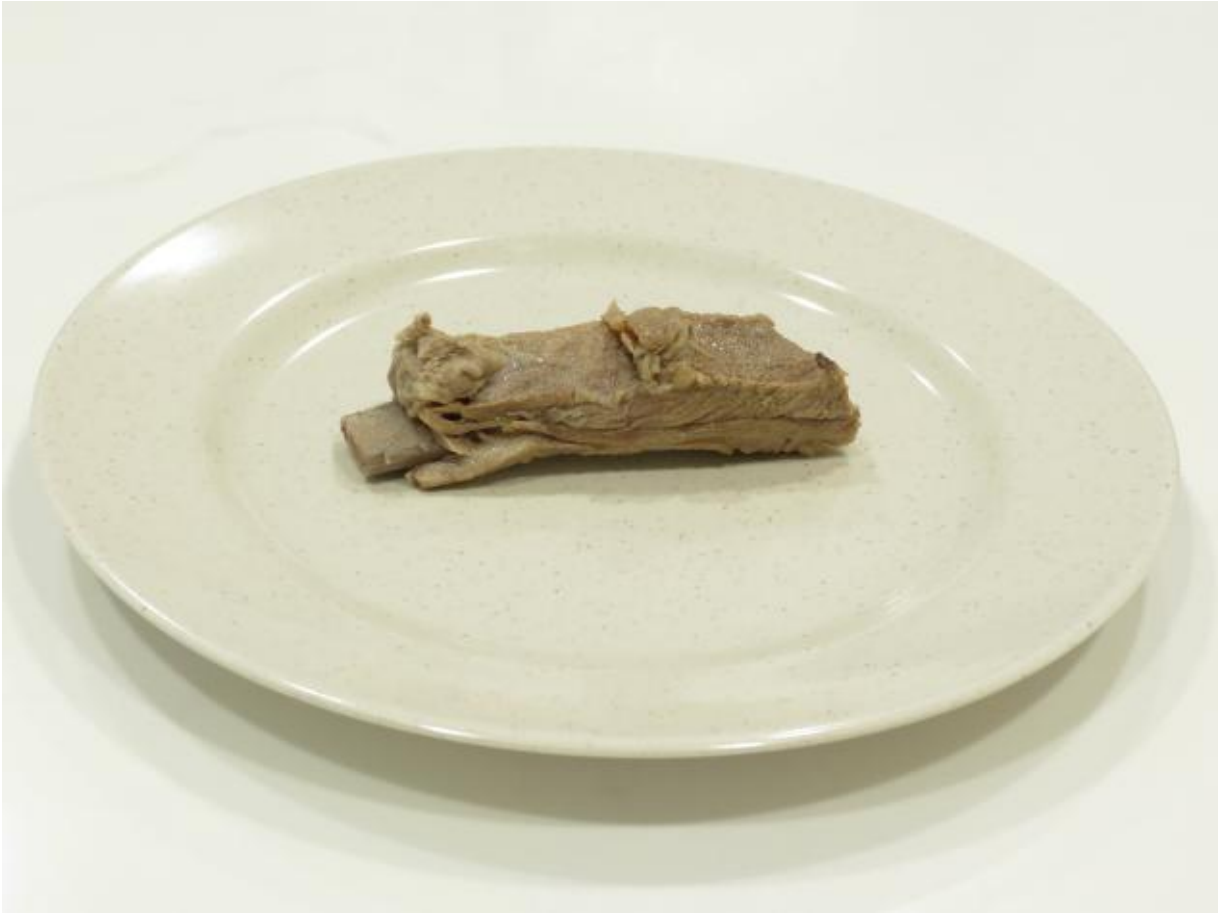

Pork rib, with bone, short (FA0184)

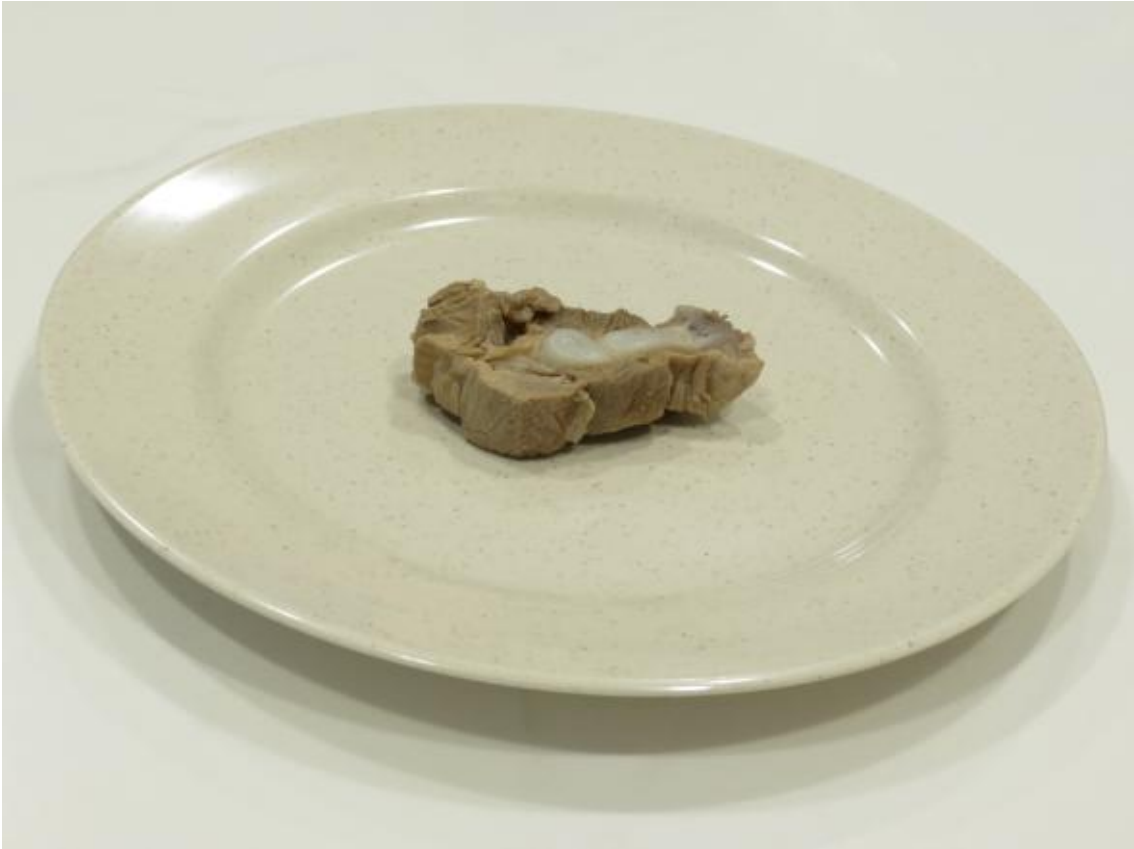

Sausage, assorted (FA0054)

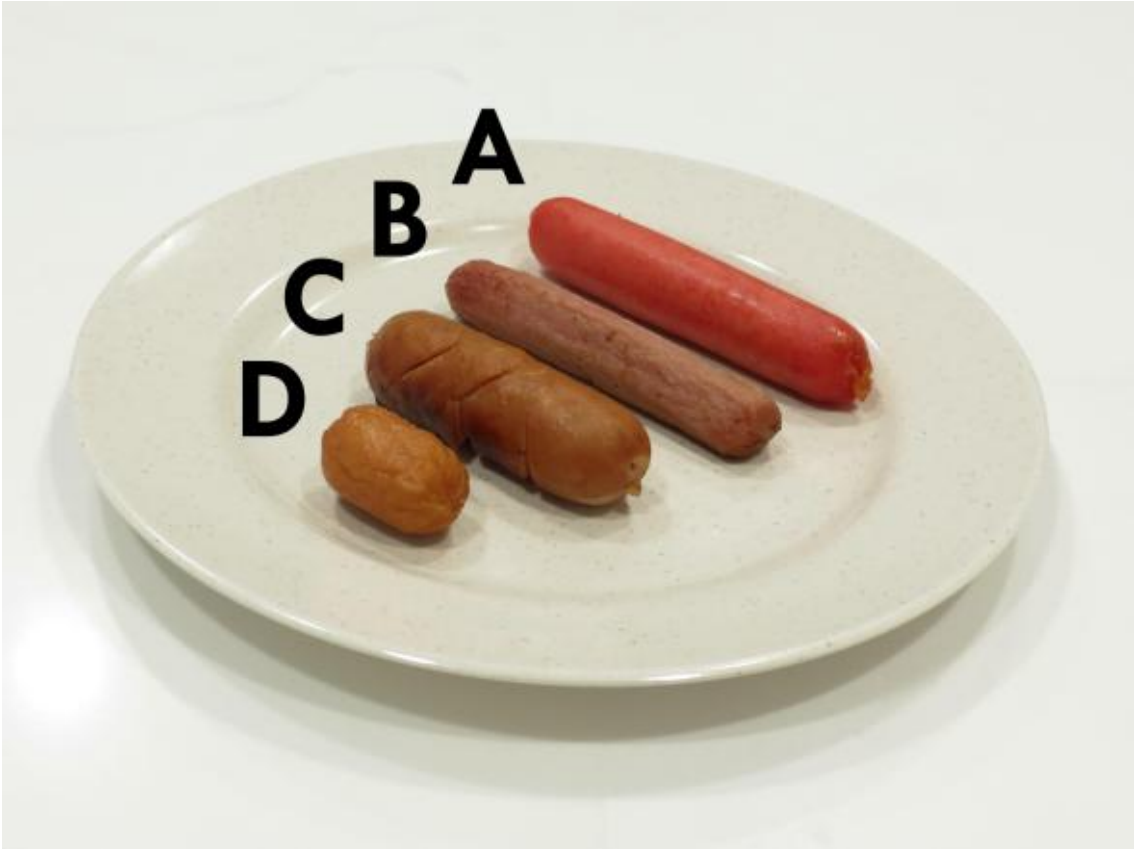

Butter, sliced (FA0149)

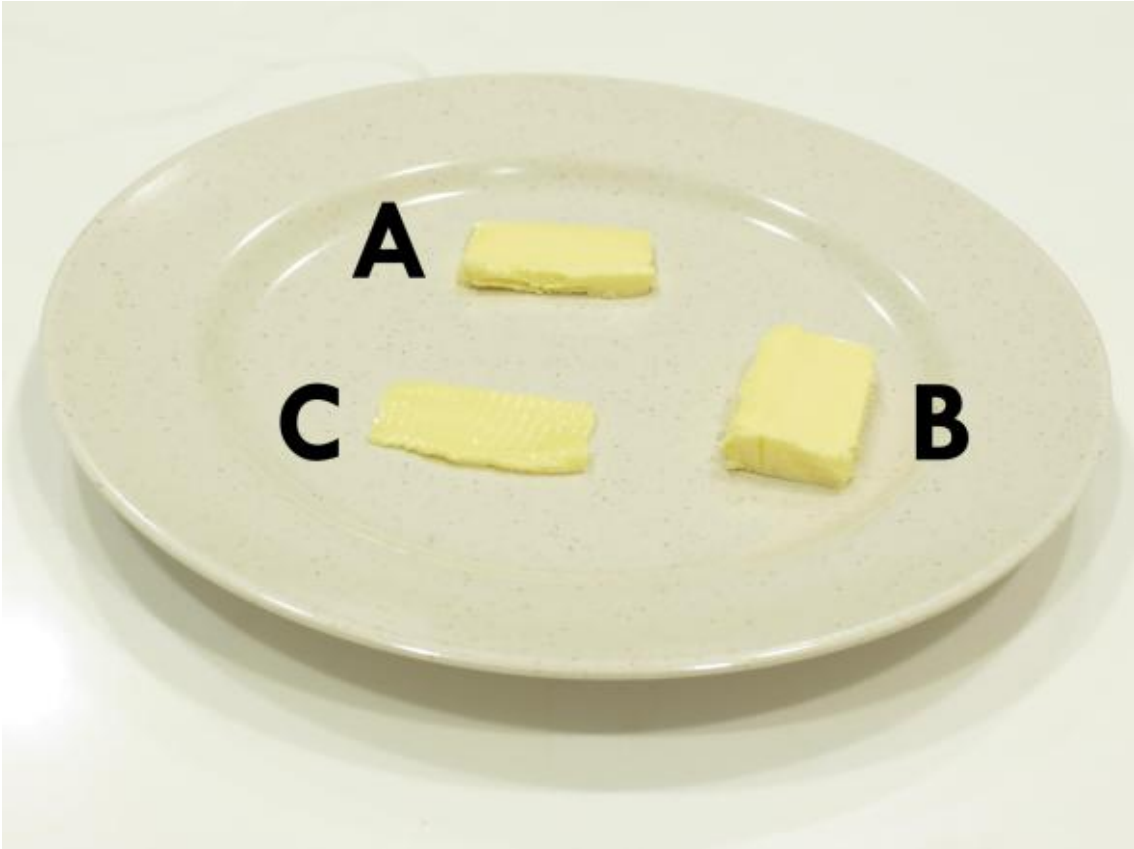

Cheese, cream, soft (FA0062)

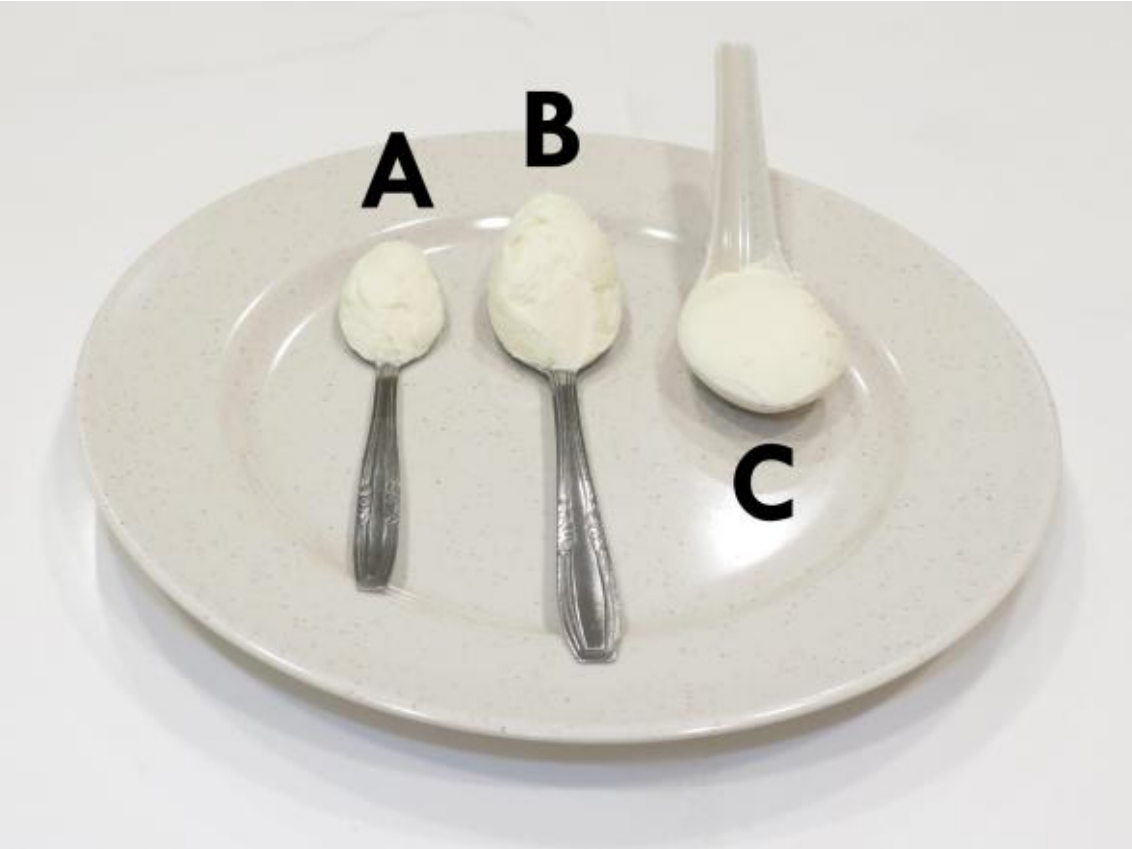

Cheese, mozzarella, shredded (FA0061)

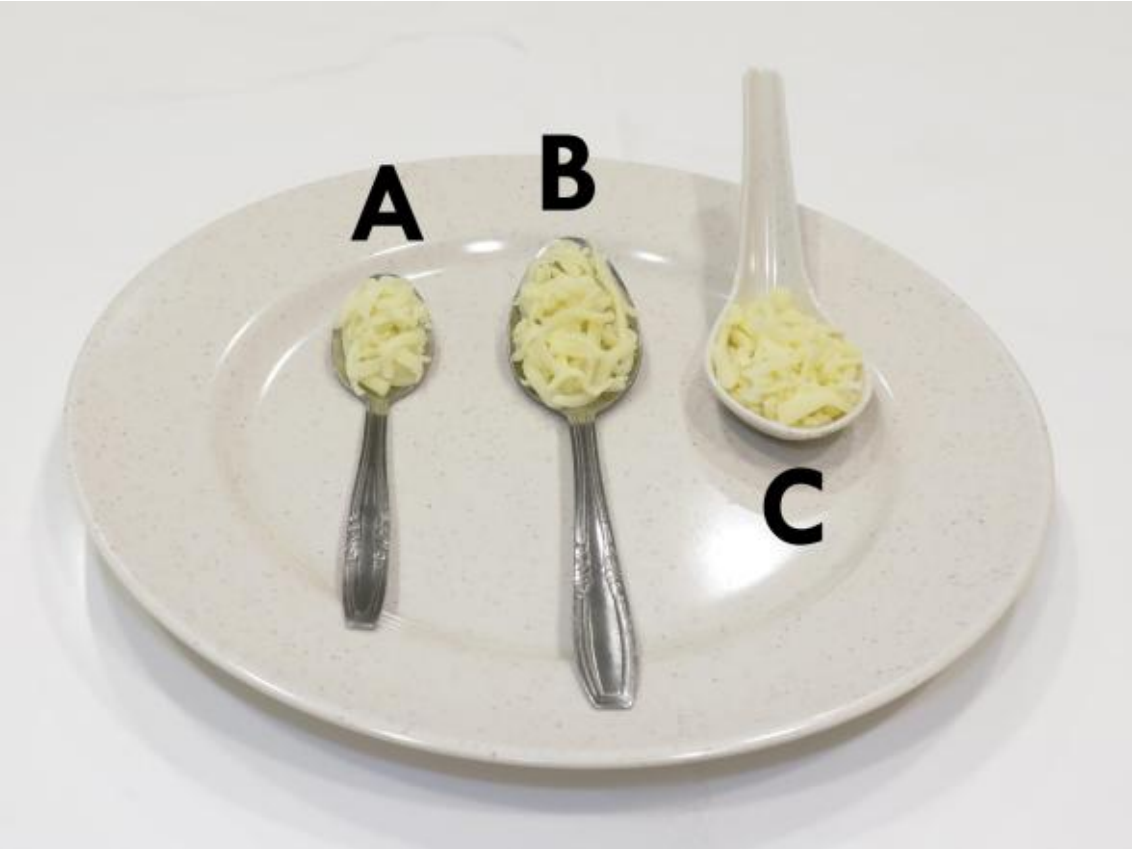

Milk, condensed (FA0108)

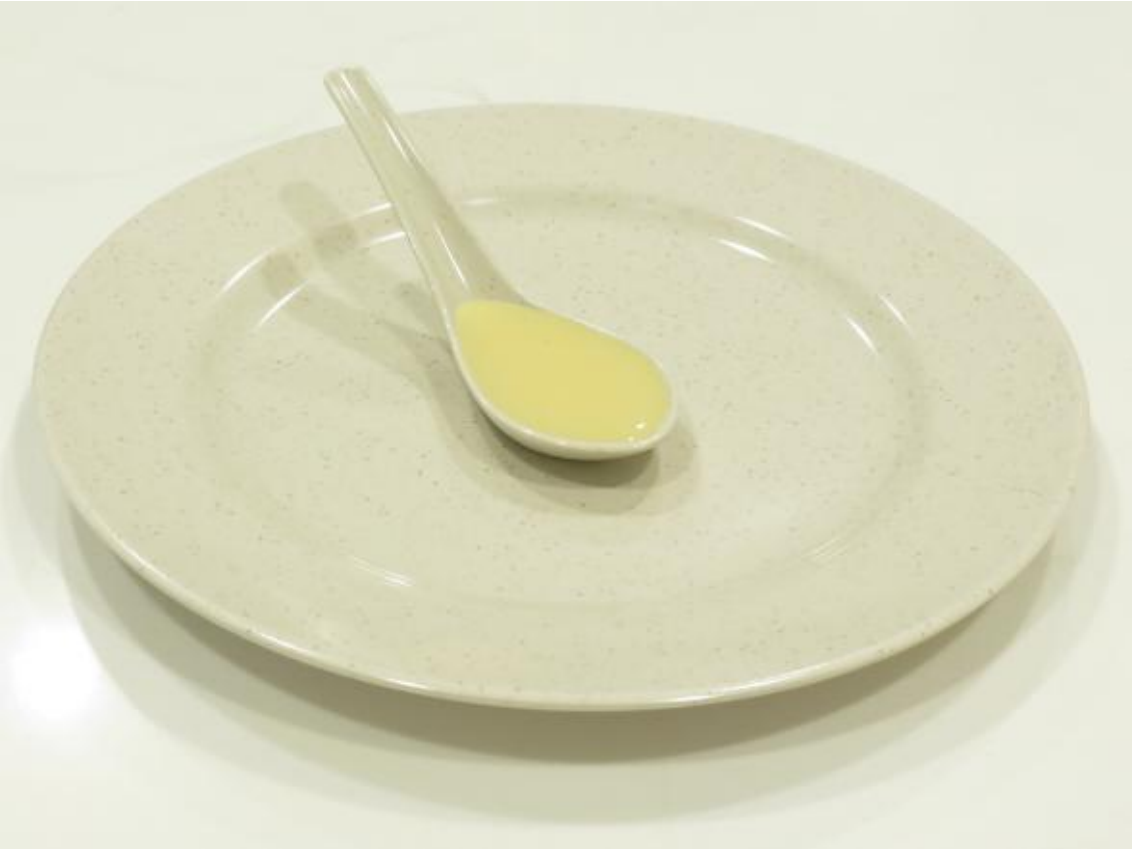

Yoghurt (FA0133)

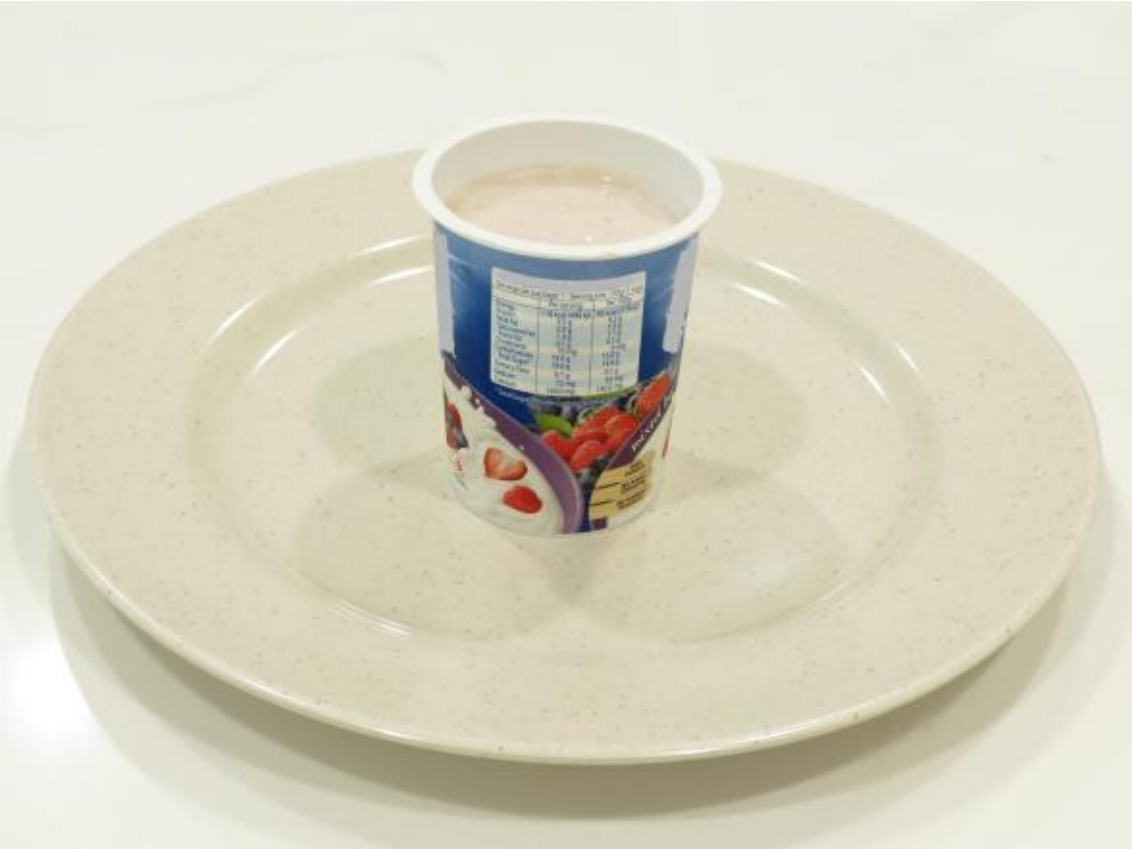

Cuttlefish snack, red, sugary (FA0011)

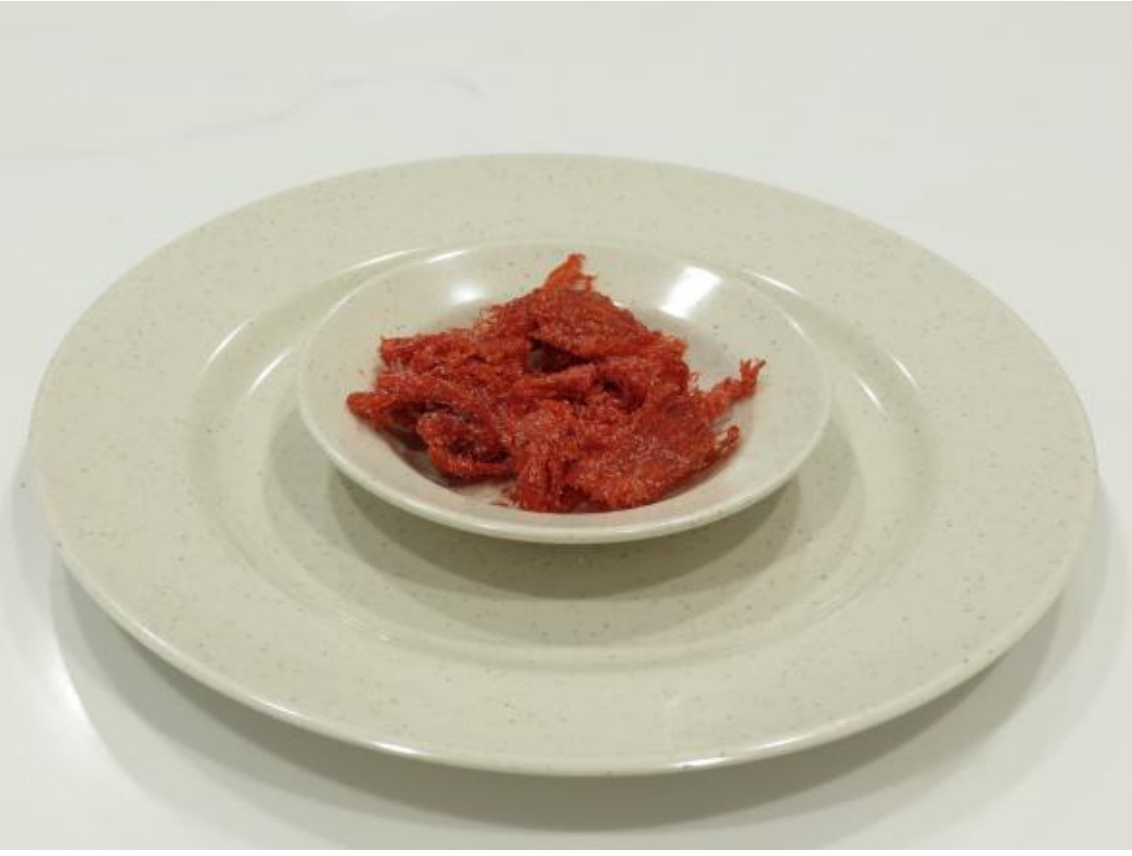

Cuttlefish snack, shredded (FA0012)

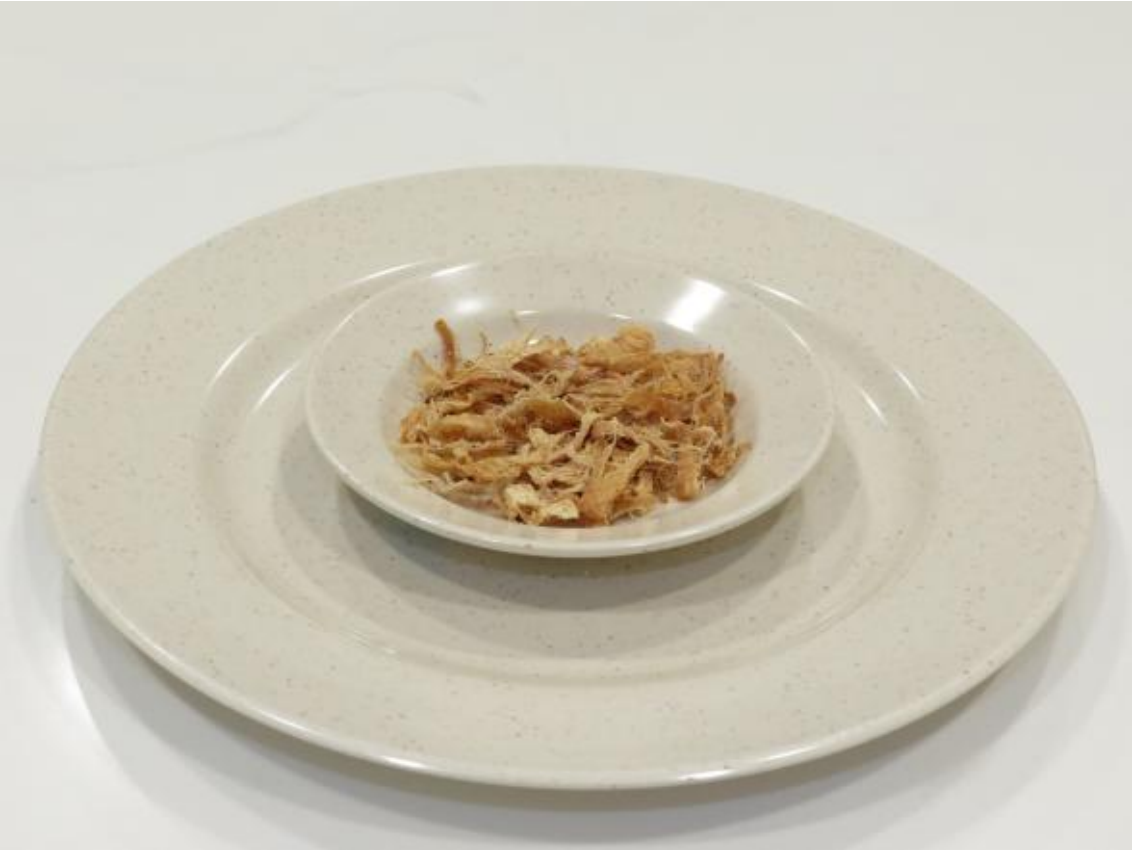

Black pepper, seed (FA0041)

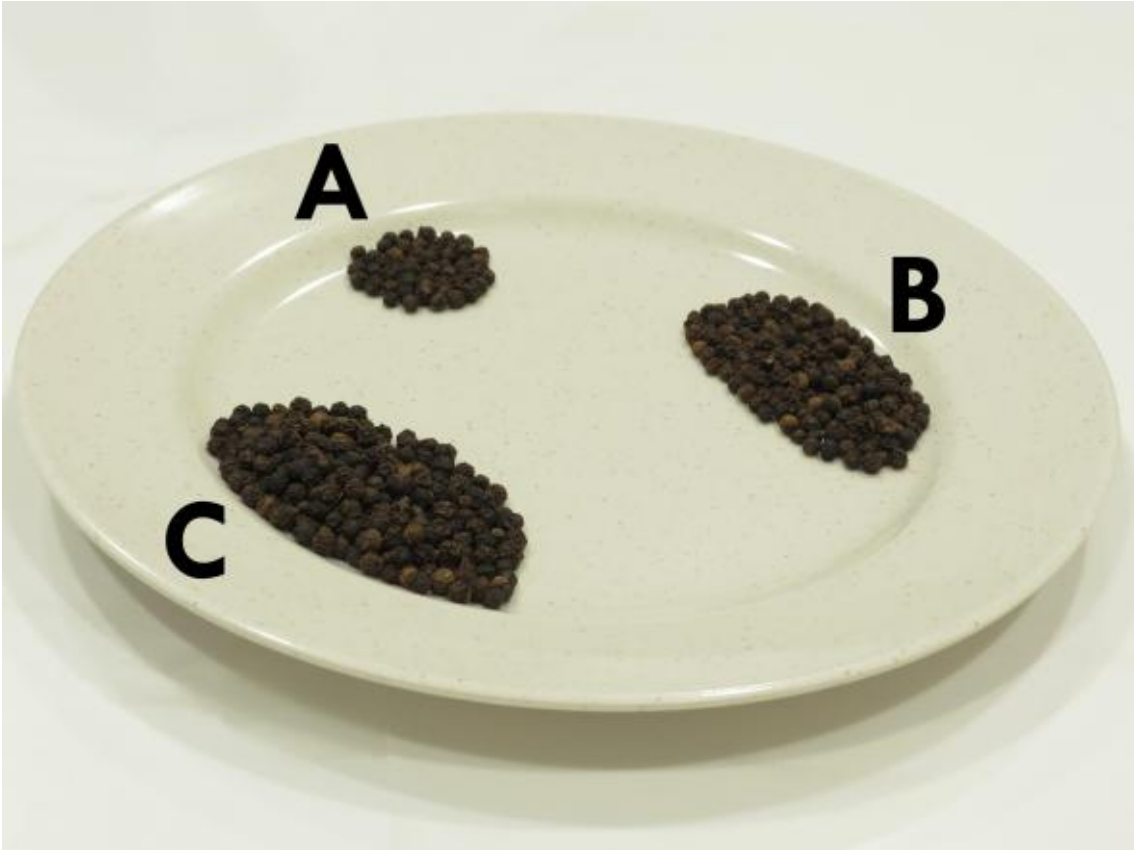

Chilli, powder (FA0083)

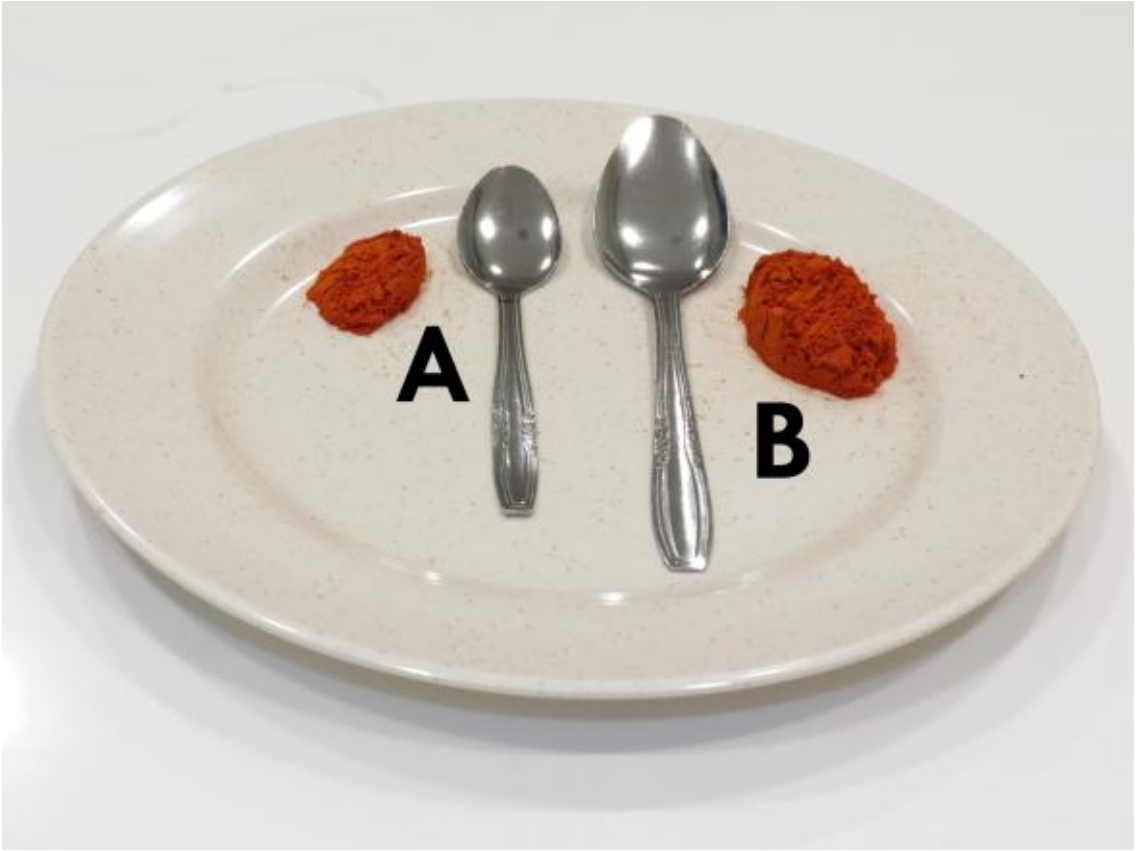

Chilli, sambal (FA0199)

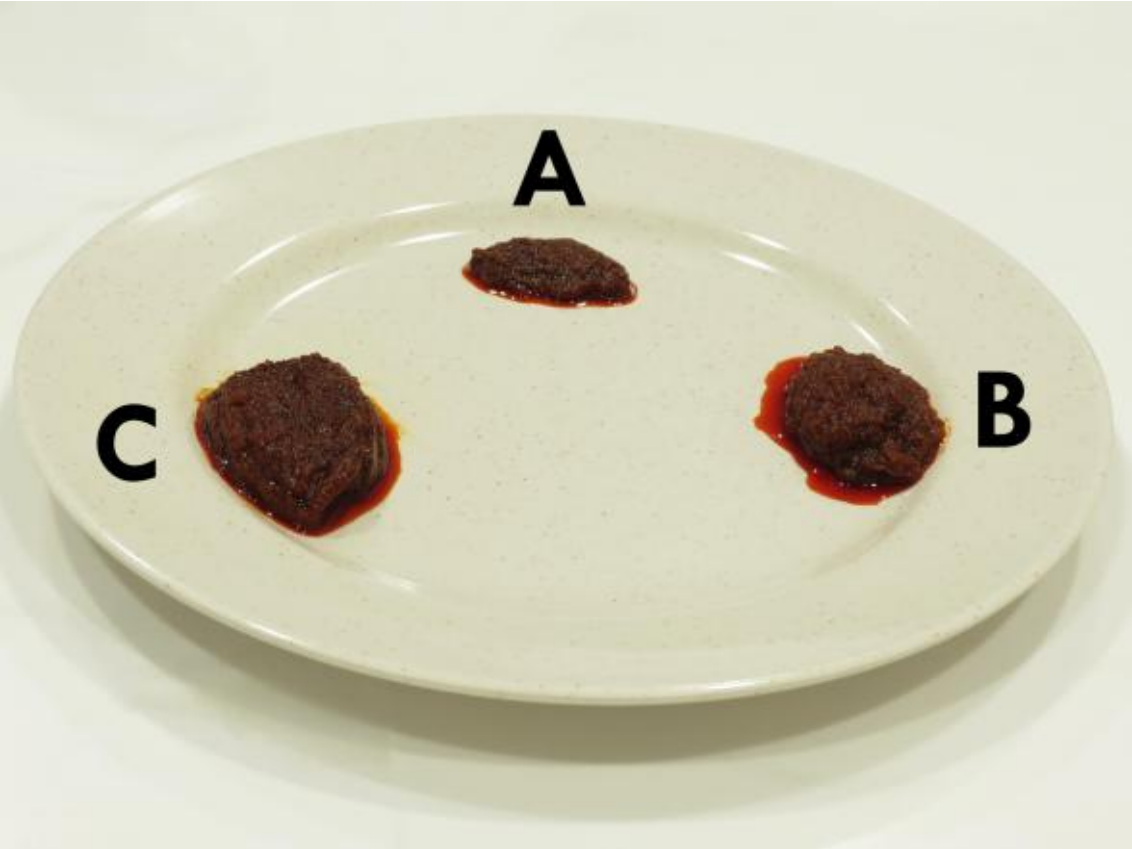

Salt (FA0106)

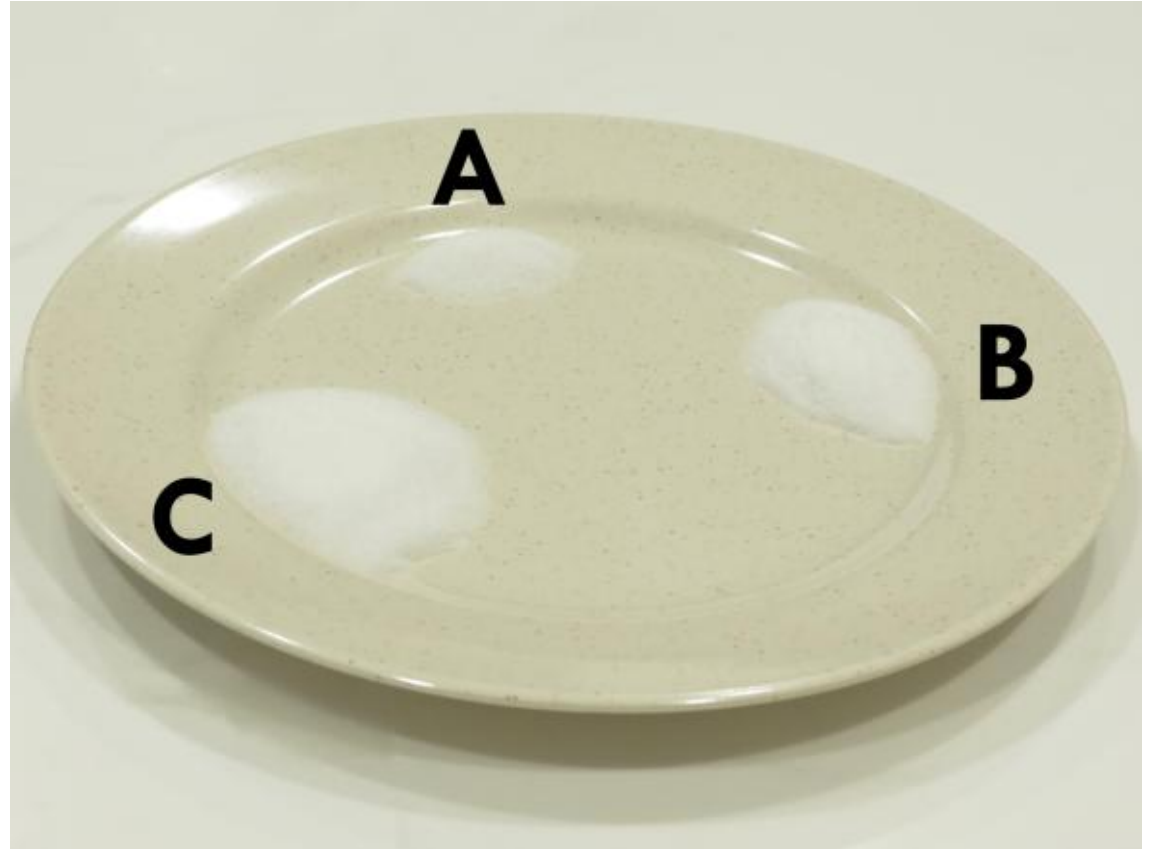

Sauce, black pepper (FA0171)

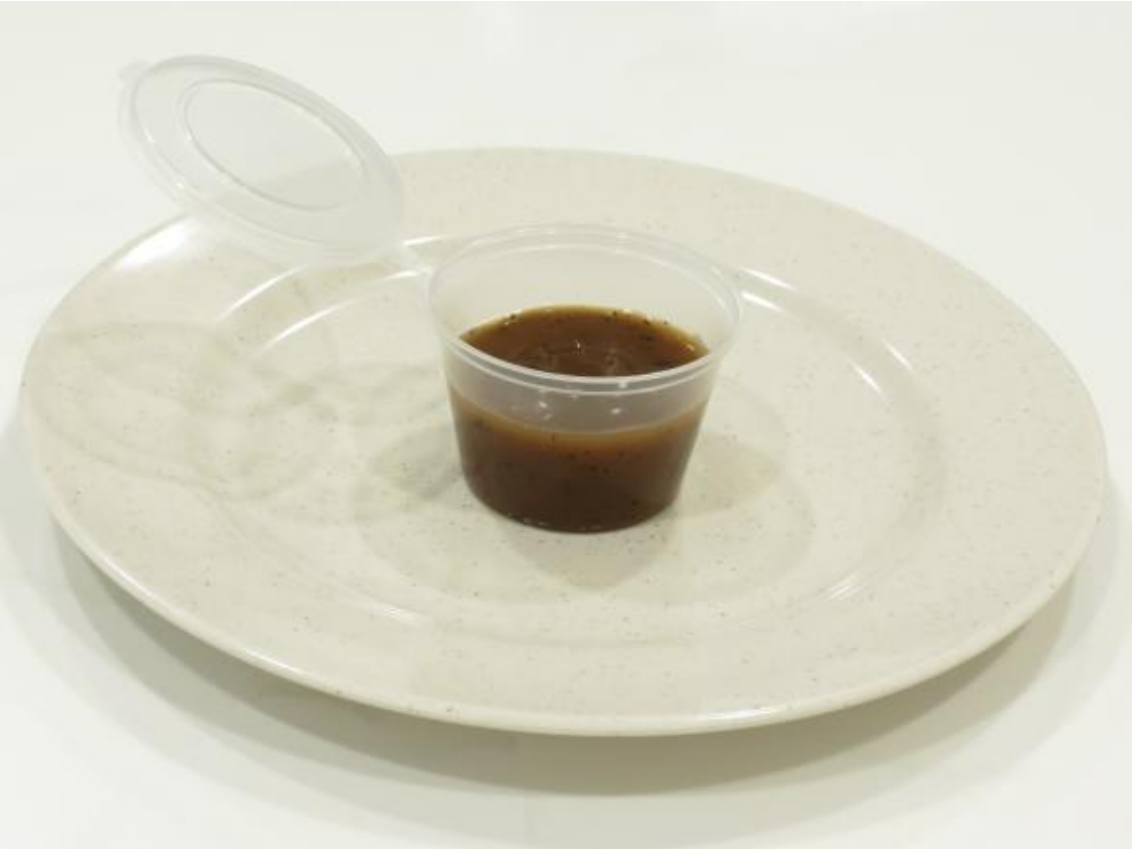

Sauce, chilli, sachet (FA0142)

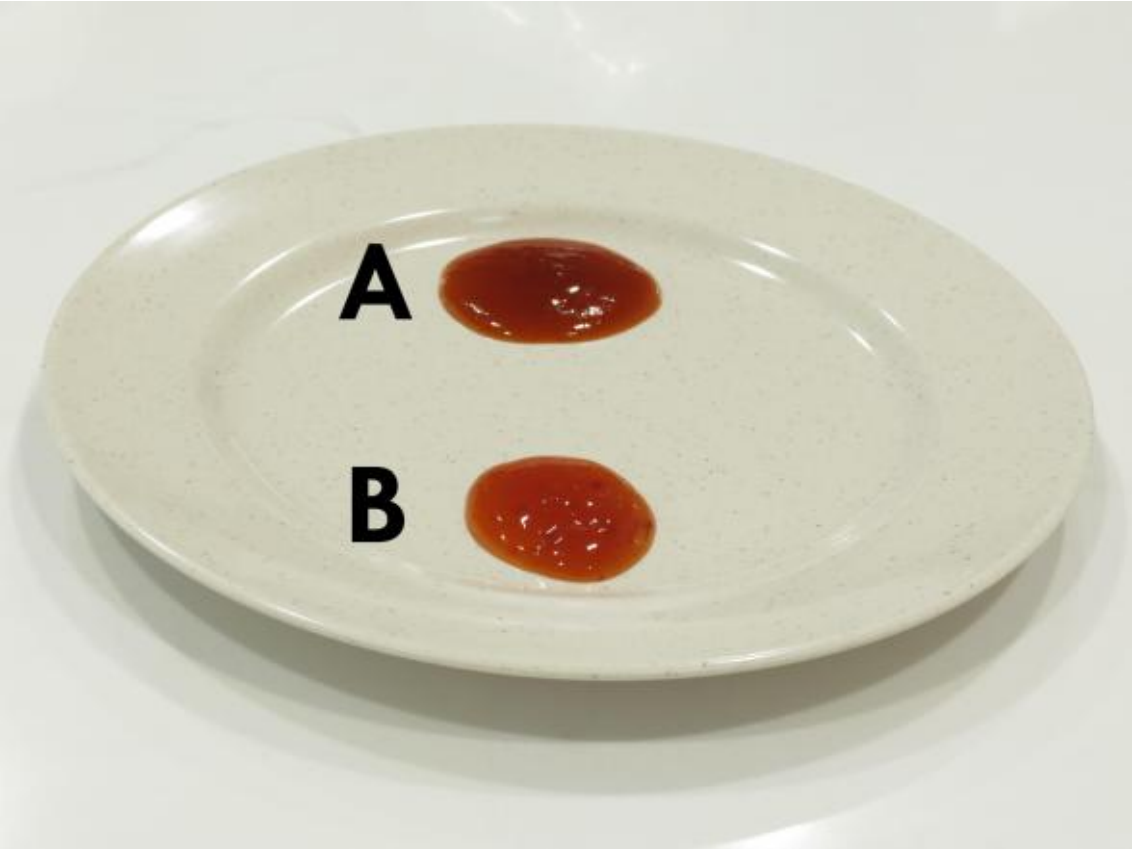

Sauce, cream-based (FA0107)

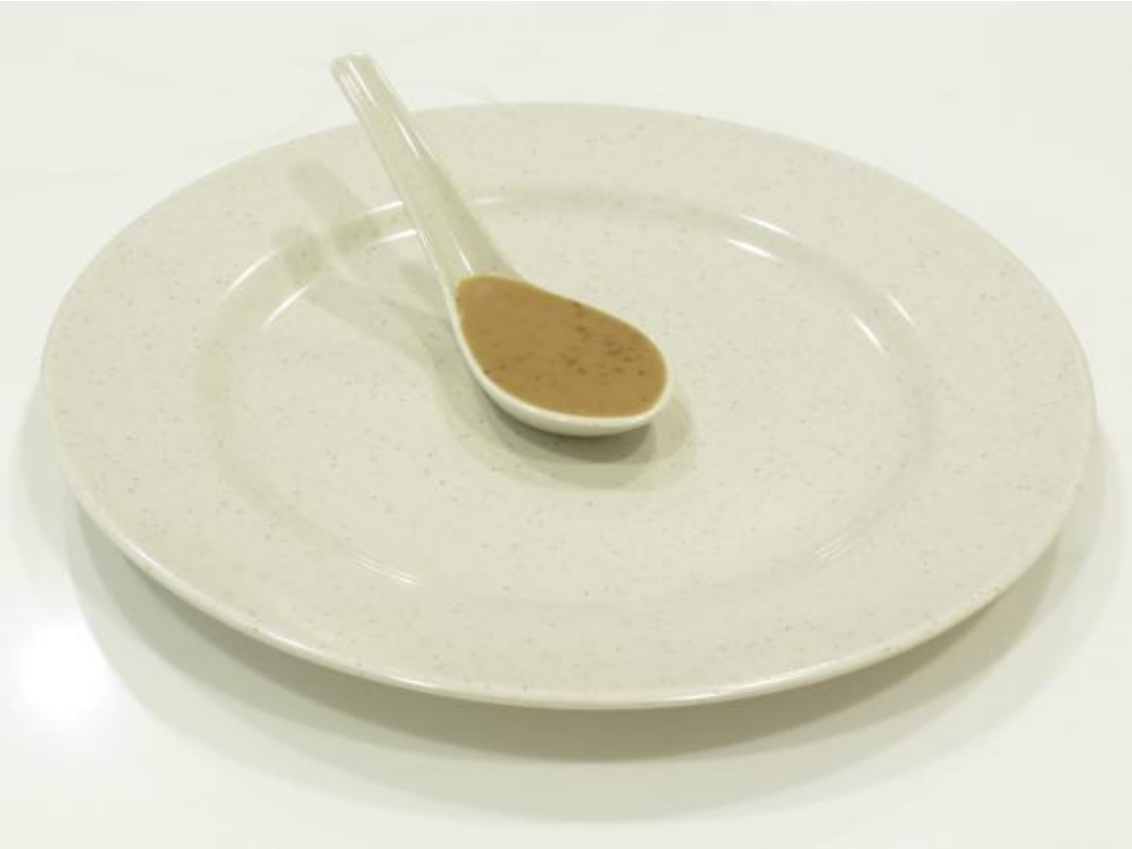

Soy sauce, dark (FA0104)

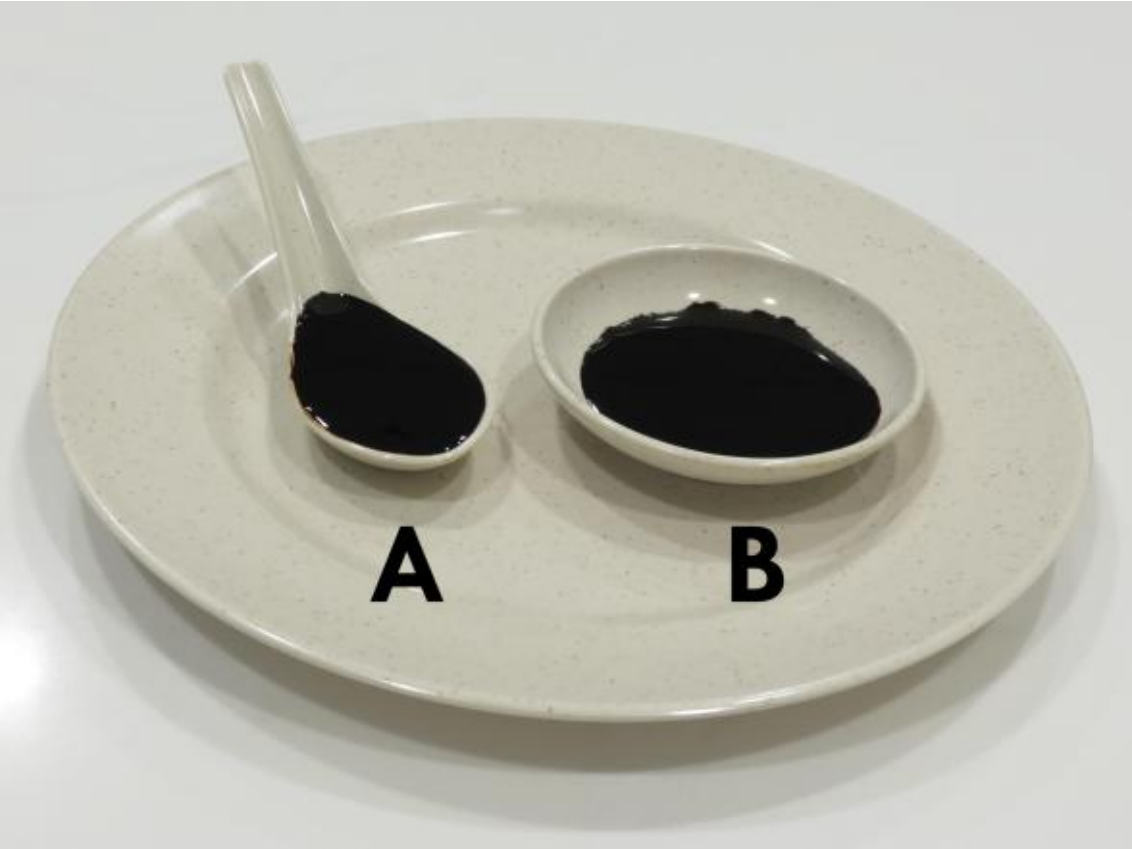

Soy sauce, light (FA0103)

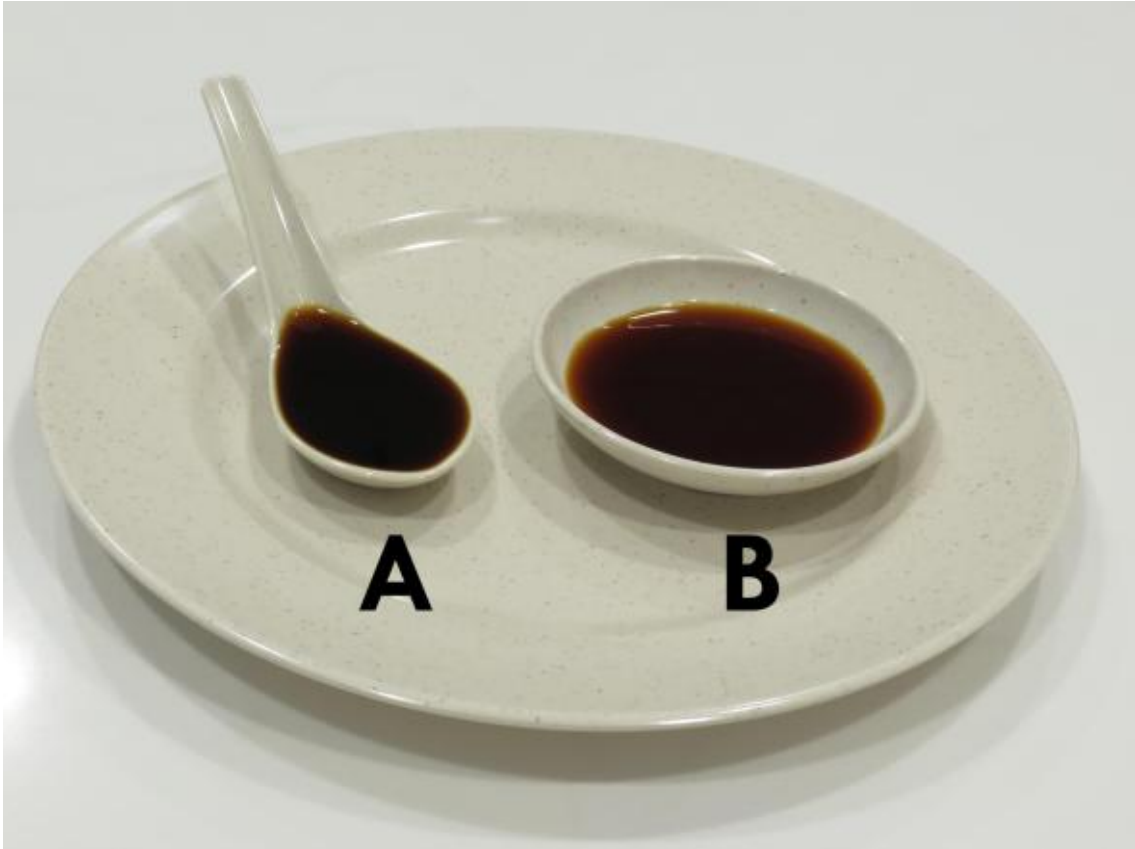

Cucumber (FA0116)

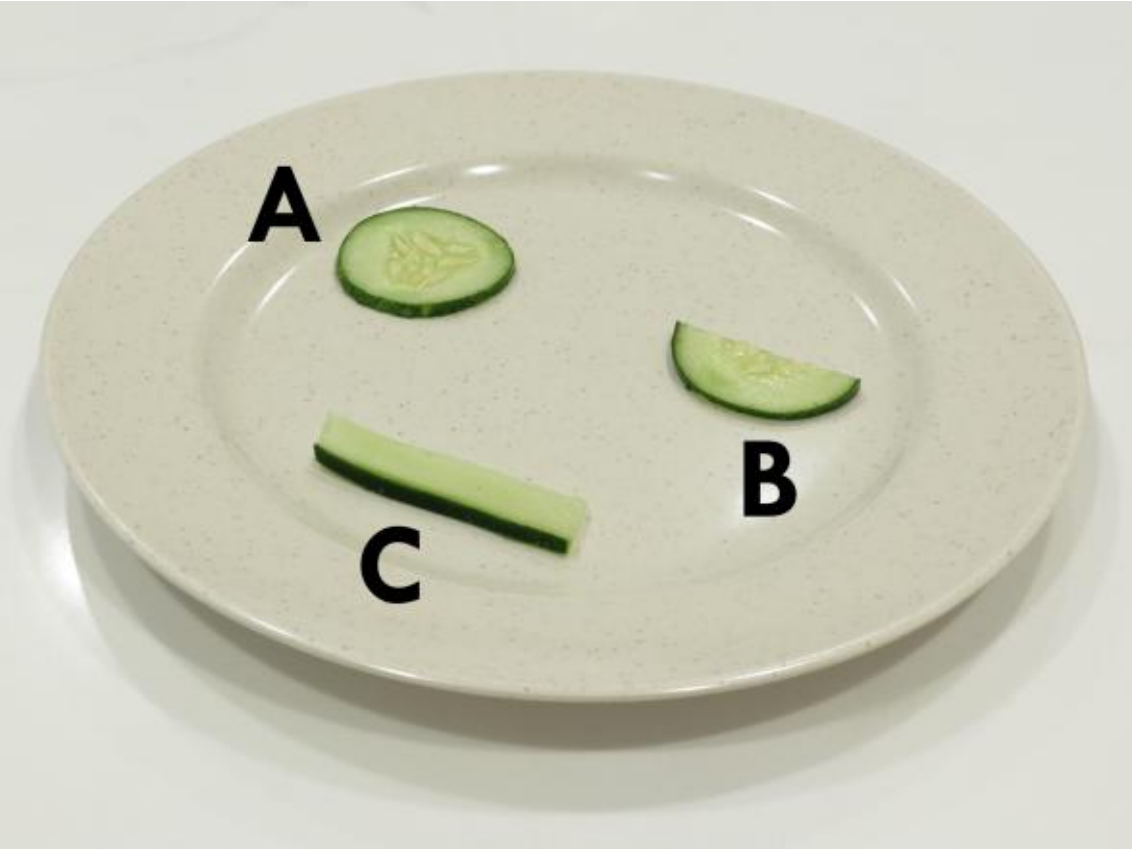

Tomato (FA0115)

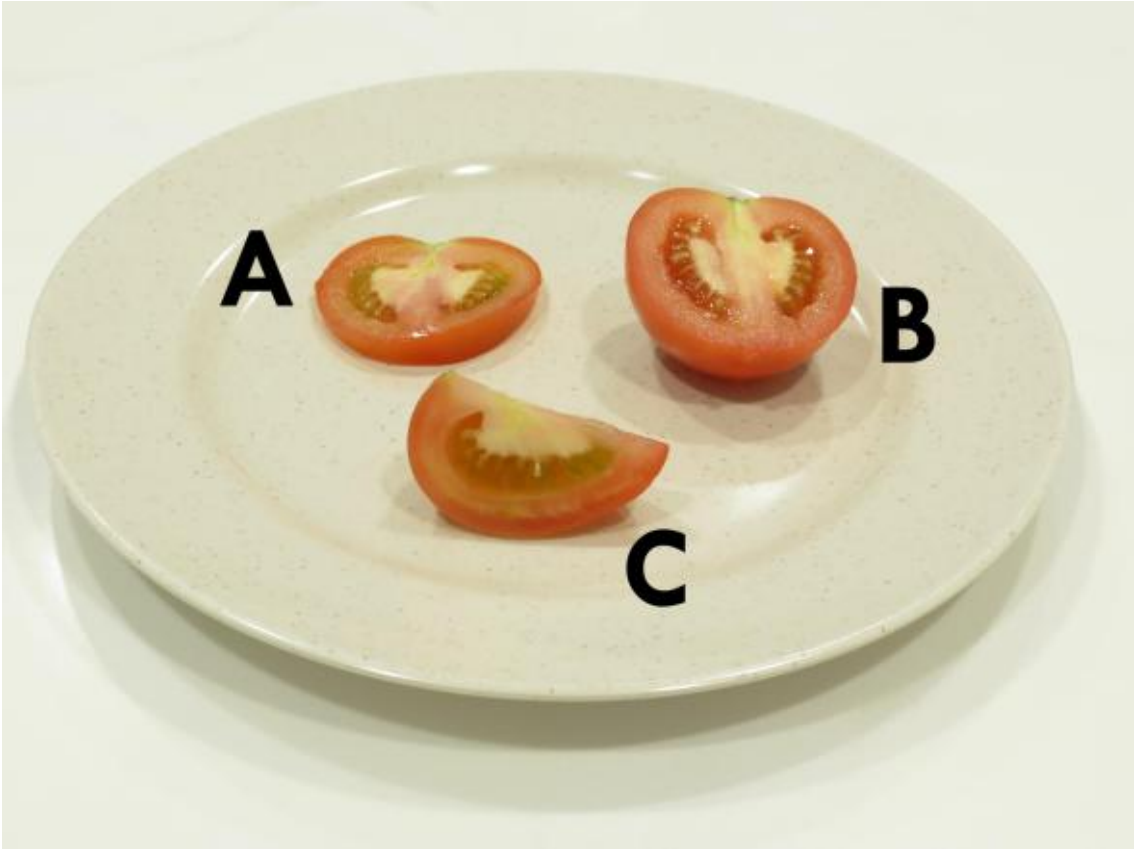

Tomato, cherry (FA0114)

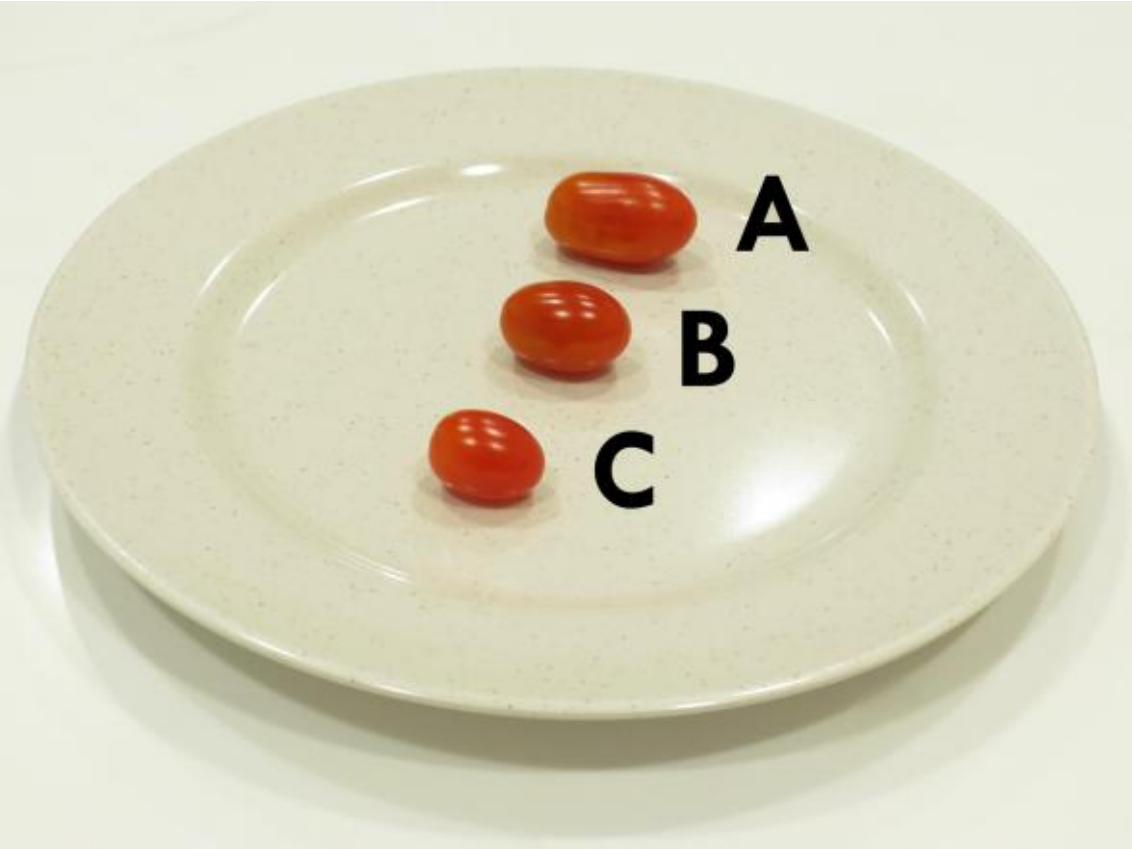

Basil, dried (FA0042)

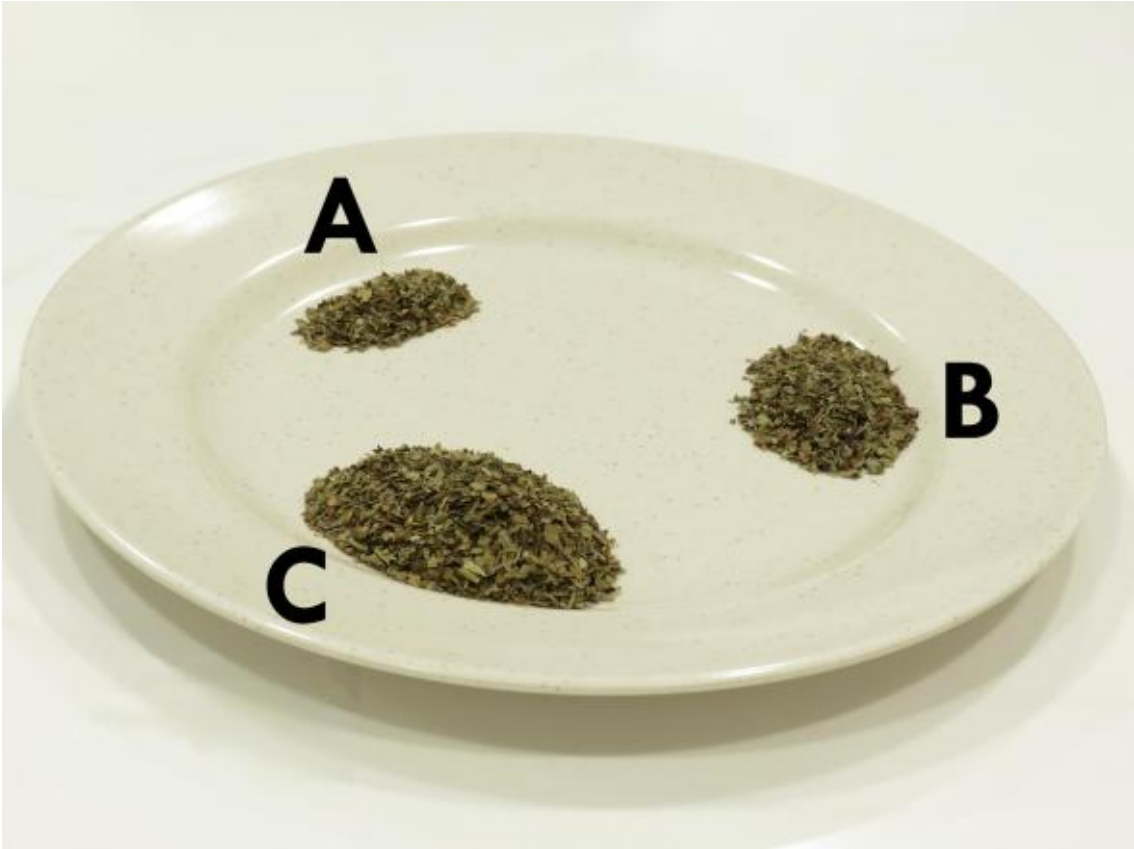

Lettuce, leaf (FA0147)

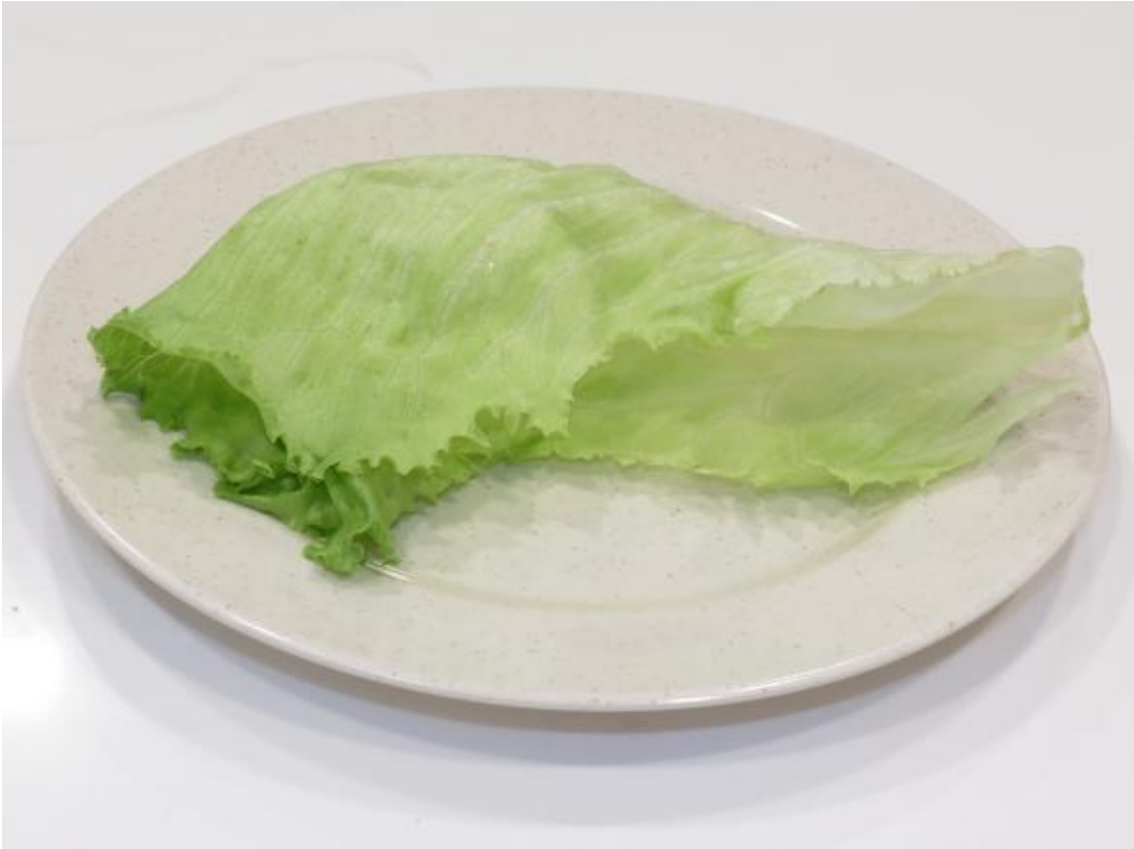

Beancurd puff (tau pok) (FA0073)

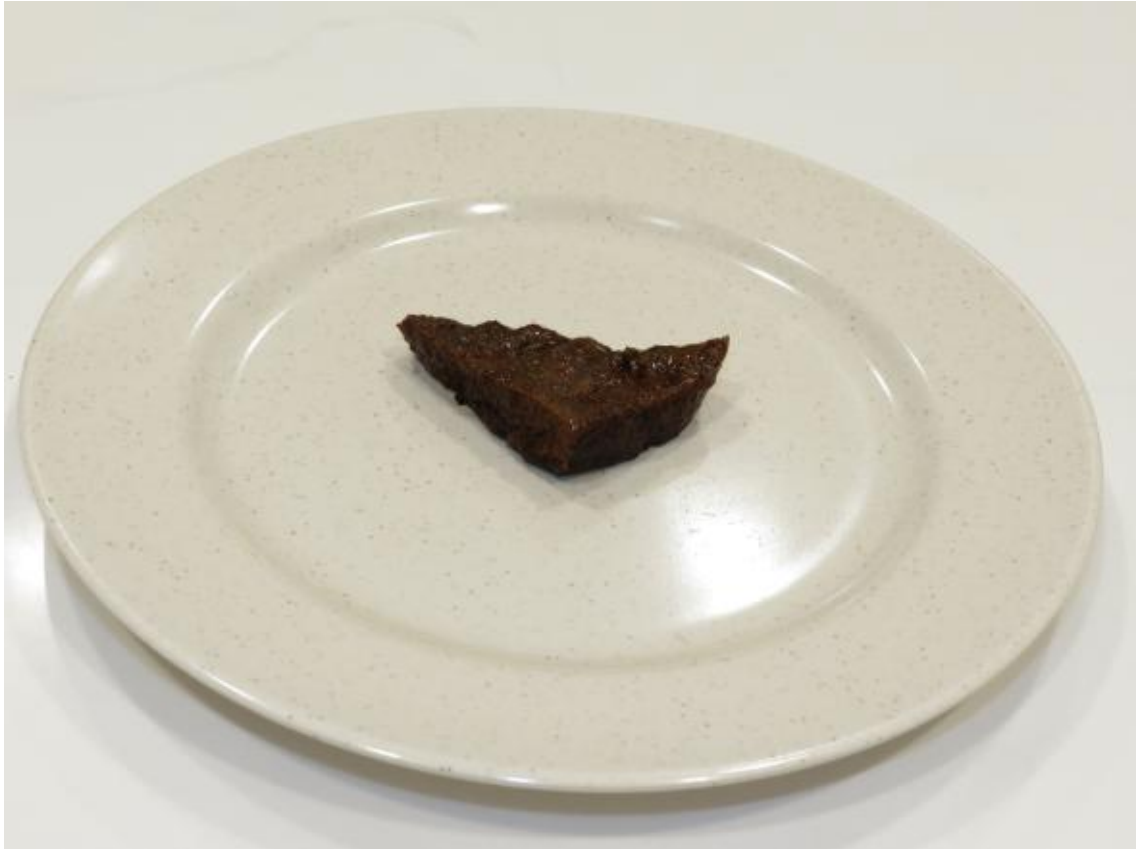

Beancurd, dessert (FA0022)

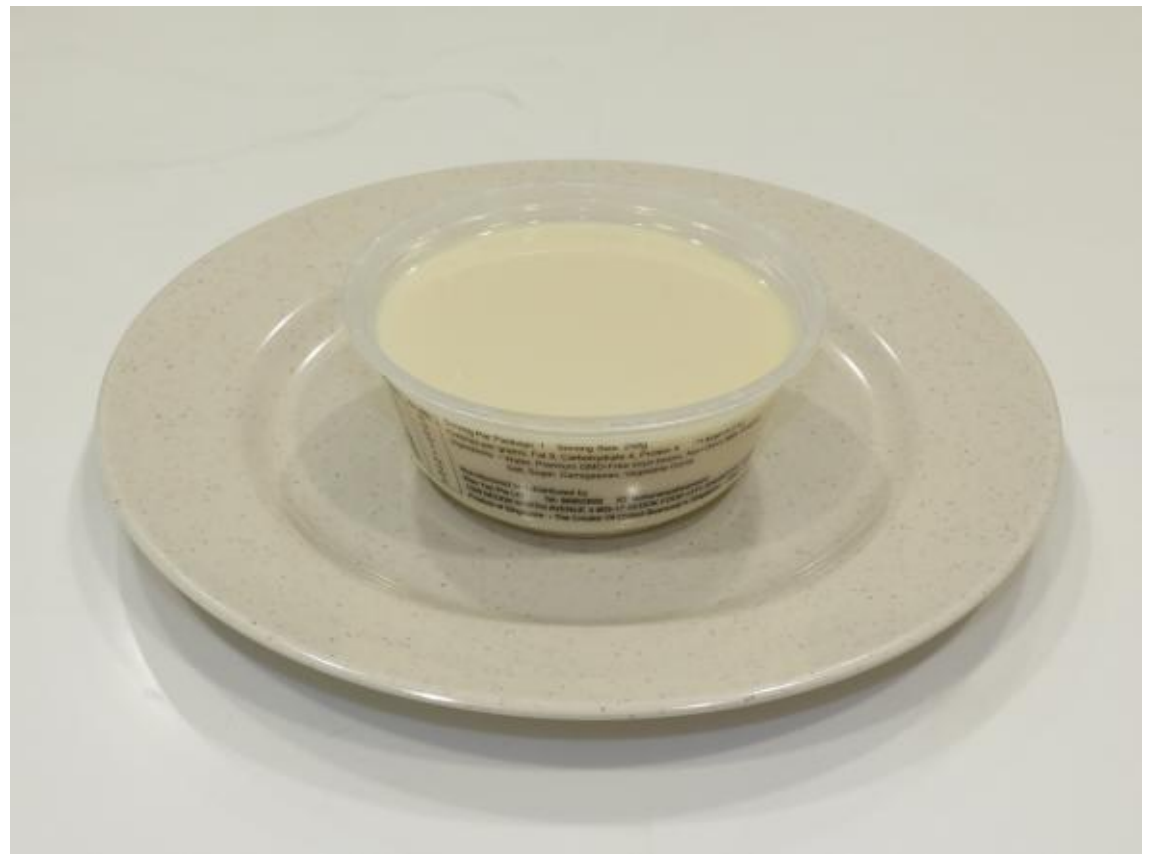

Hashbrown, assorted (FA0044)

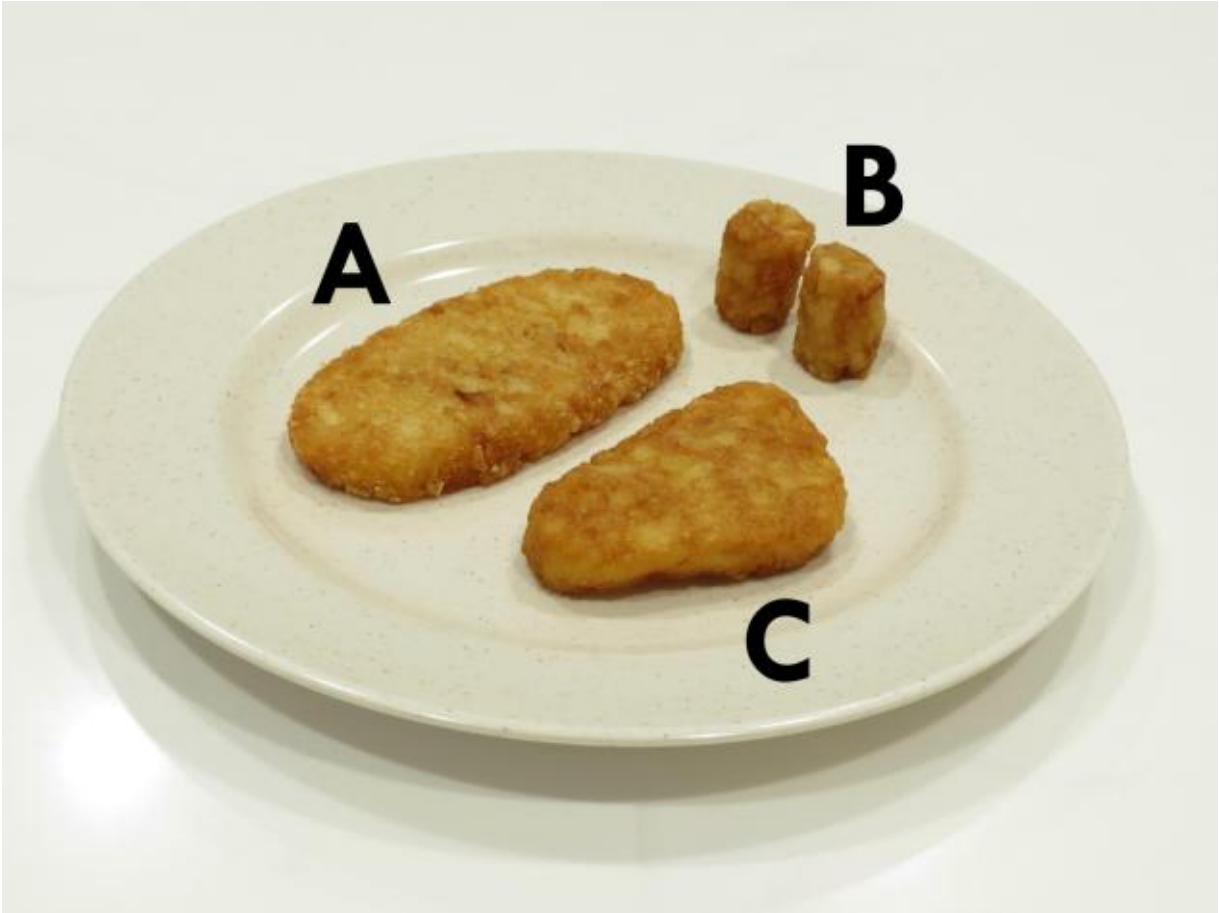

Potato, peeled (FA0093)

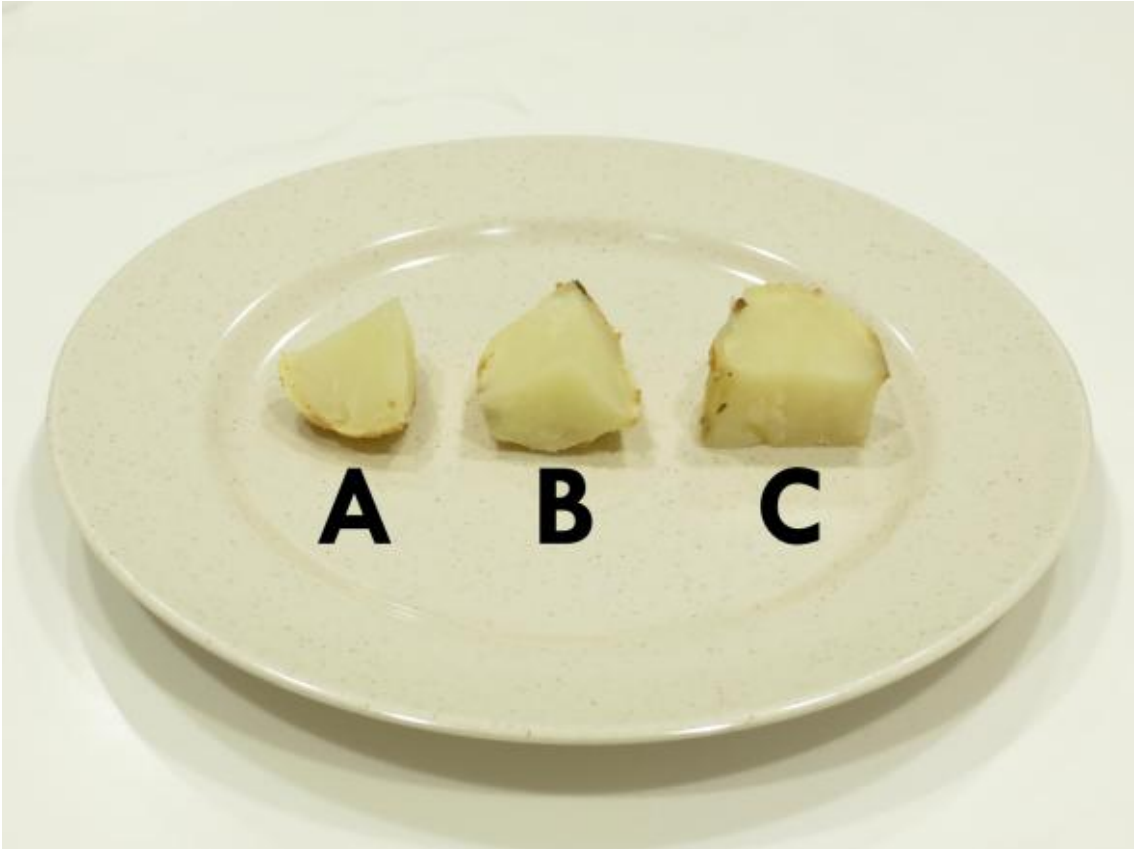

Ginger, sliced (FA0121)

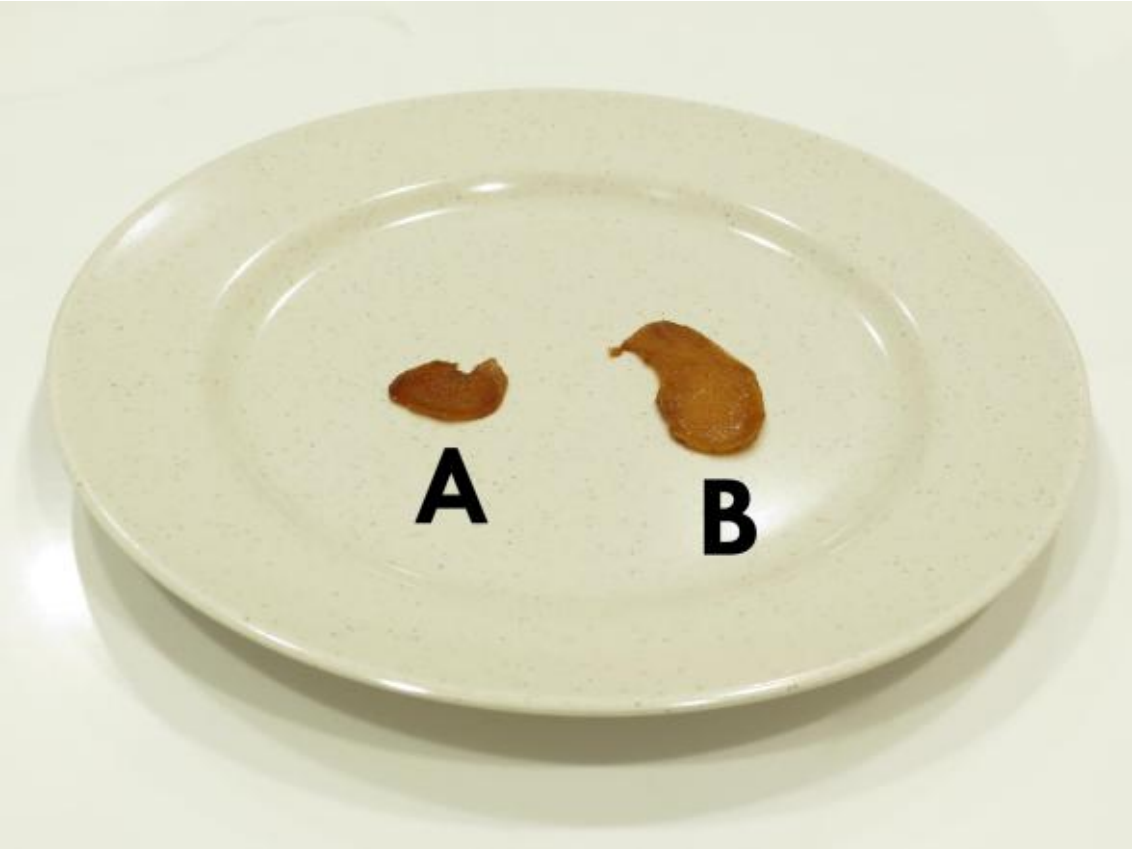

Bowl (R01)

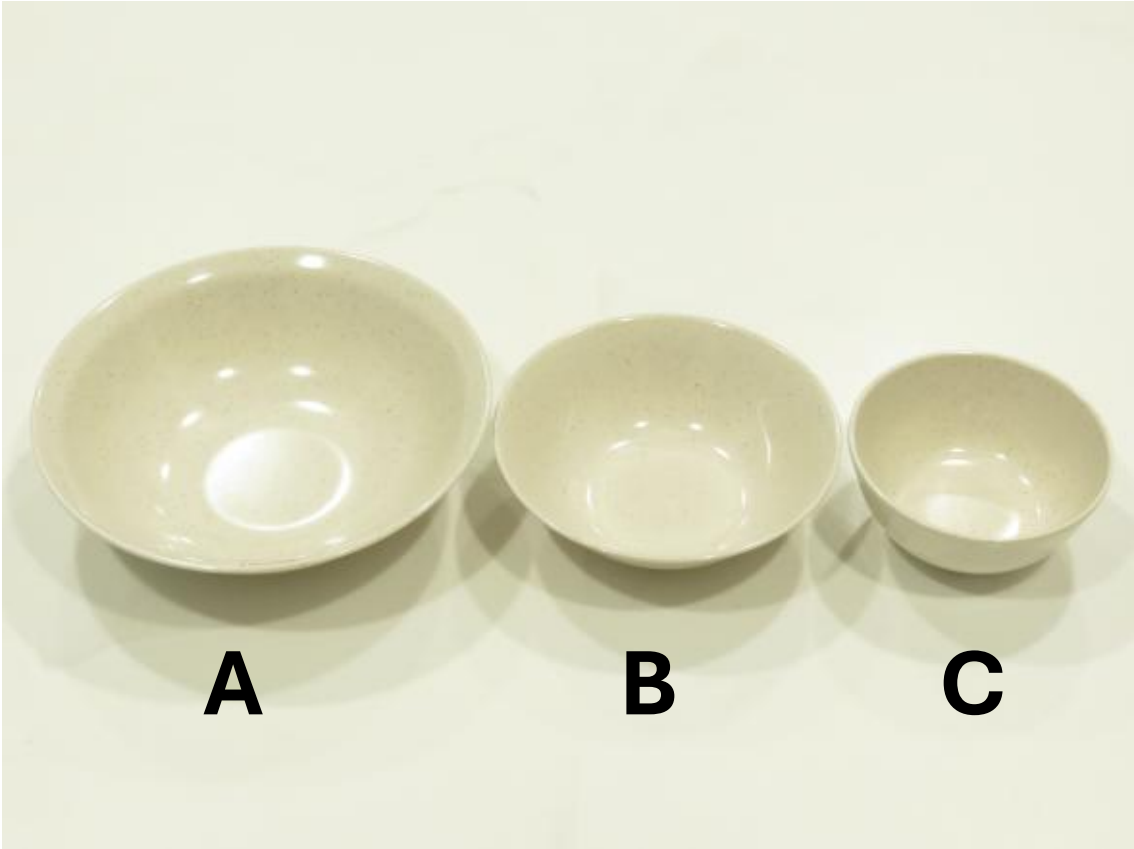

Cup (R02)

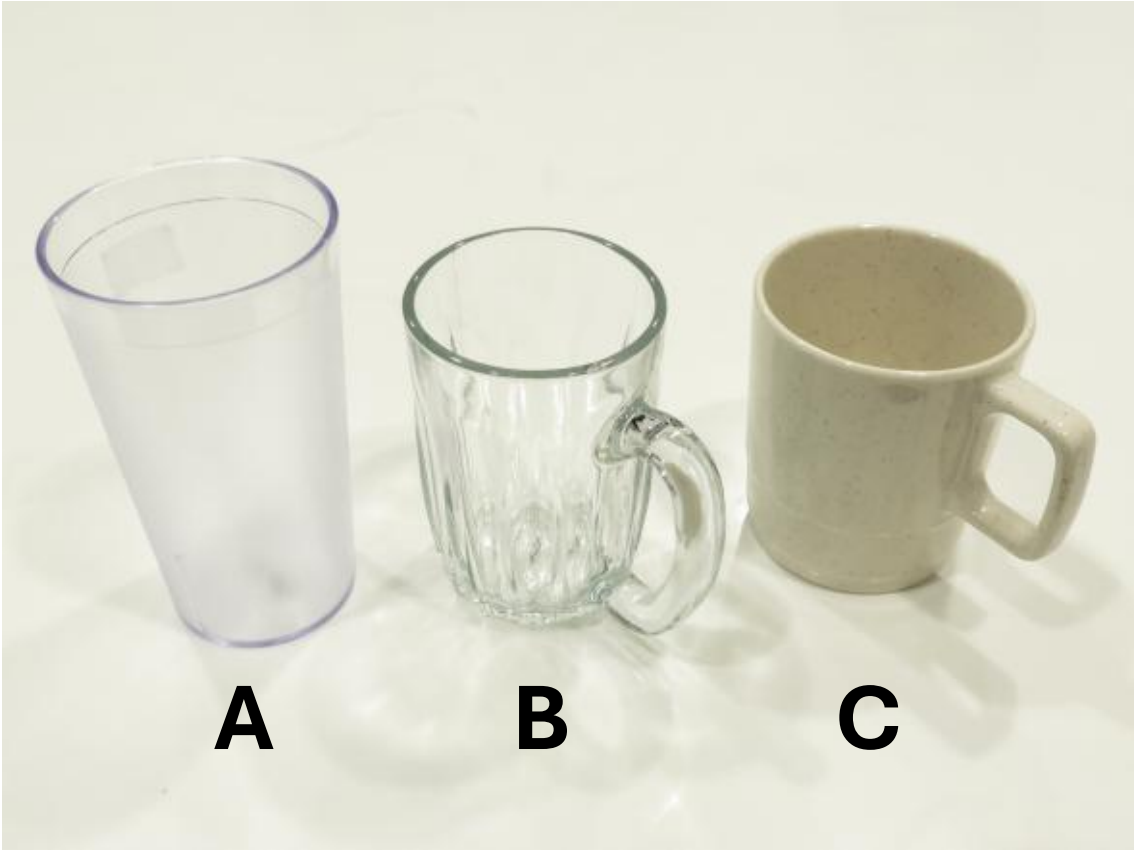

Plate/Saucer (R03)

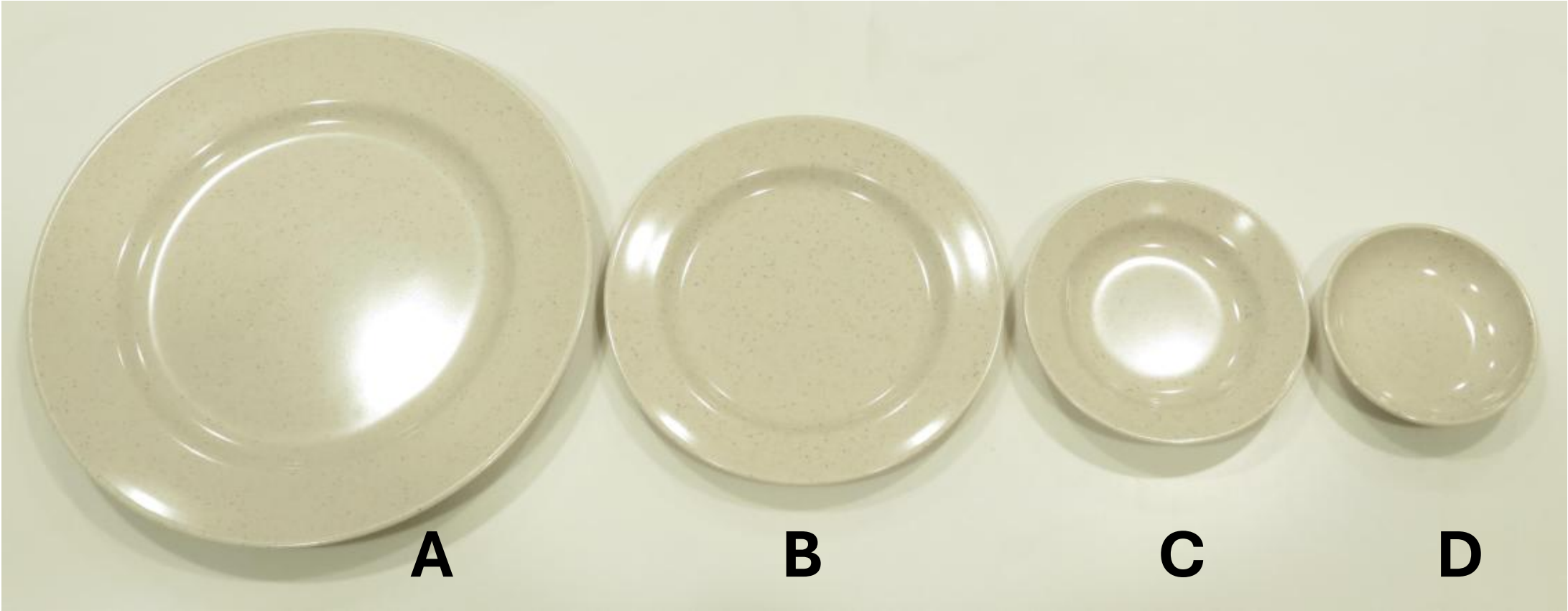

Spoon (R04)

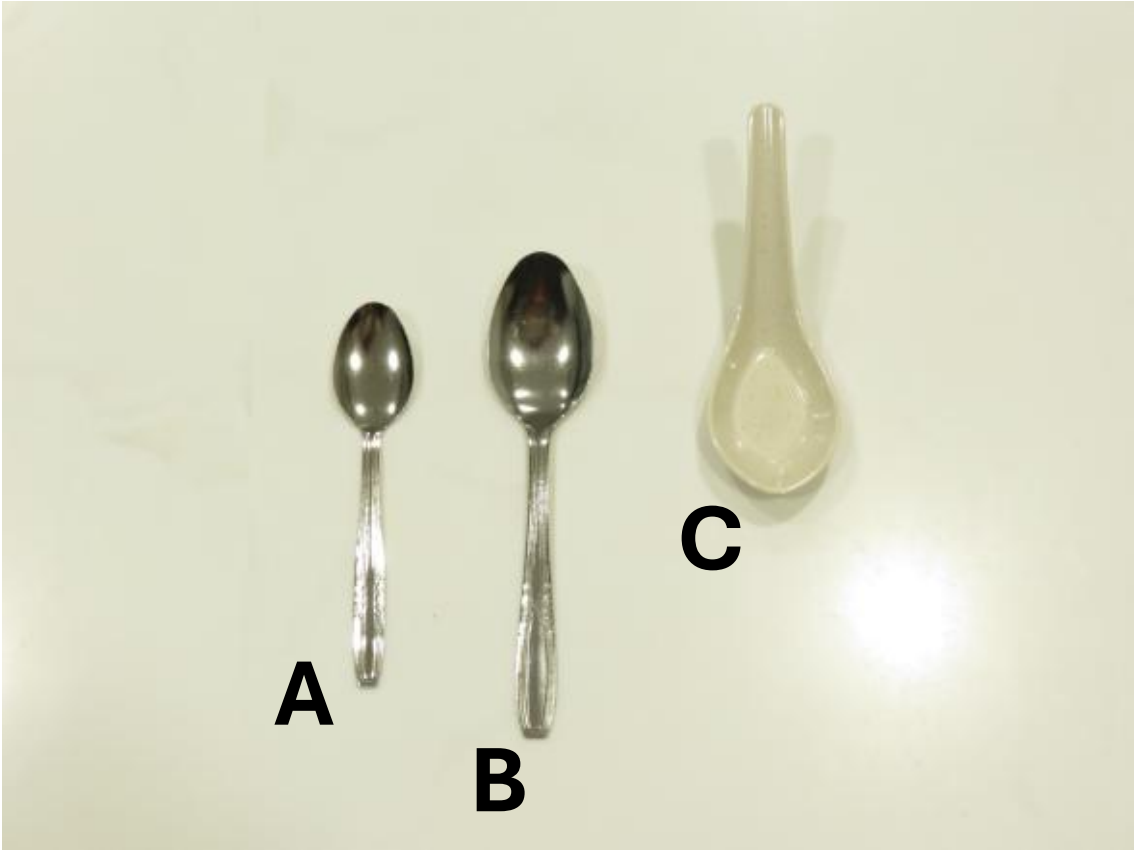

Standard plate with utensils (R05)

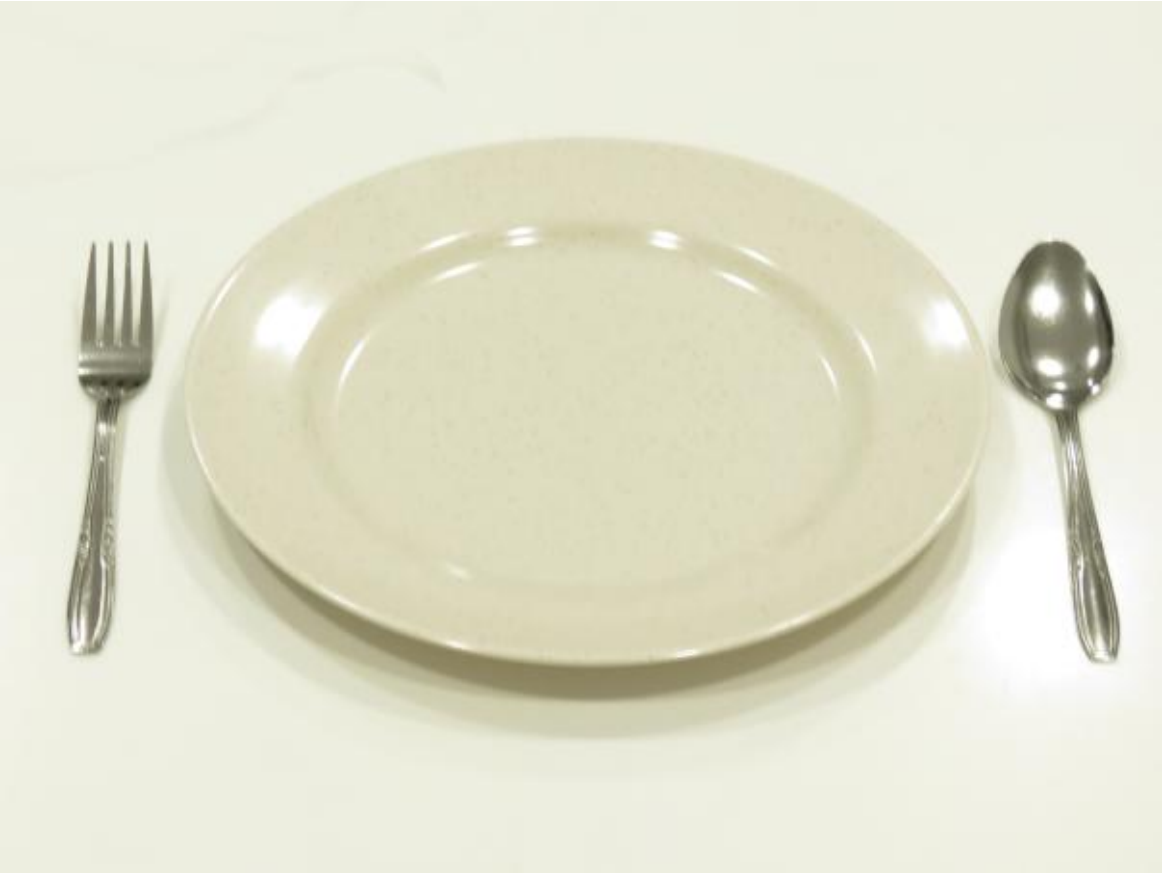

Supplement: Supplementary file 2 [file Data_Sheet_2.PDF]
