# Supplementary material for: Development and validation of a photographic food atlas for portion size estimation in Singapore
Source: Front Nutr. 2026 Sep 18;13:1930409. doi: 10.3389/fnut.2026.1930409 (PMC13630621; doi:10.3389/fnut.2026.1930409)
Supplement: Supplementary file 3 [file Data_Sheet_3.PDF]

Evaluation Form

Session No.: 1

Date: ________________

Time: ________________

Assigned Identifier: ________________

Section I – Participant Information

Please indicate your age group.

☐ 18 – 30 years

☐ 30 – 40 years

☐ Above 40 years

Please indicate your sex.

☐ Male

☐ Female

How familiar are you with accurate measurements in your daily activities?
(e.g. weighing of foods, volumetric measurements using pipettes)

☐ 1 – Not familiar

☐ 2 – Somewhat familiar

☐ 3 – Very familiar

Section II – Study Evaluation

For each of the foods listed below, please circle the code (#1, #2, #3, #4) which best represents the portion presented.

A. Rice, white	1	2	3	4
B. Mushroom, sliced	1	2	3	4
C. Peanuts	1	2	3	4
D. Spaghetti	1	2	3	4
E. Chicken	1	2	3	4
F. Egg, scrambled	1	2	3	4
G. Strawberry	1	2	3	4
H. Fish, sliced	1	2	3	4
I. Broccoli	1	2	3	4
J. Beansprouts	1	2	3	4
K. Blueberry	1	2	3	4
L. Kailan	1	2	3	4
M. Beef, sliced	1	2	3	4
N. Soup, cream	1	2	3	4

Evaluation Form

Session No.: 2 **Date: _______________**

Assigned Identifier: _______________ **Time: _______________**

For each of the foods listed below, please circle the code (#1, #2, #3, #4) which best represents the portion presented.

A. Rice, white	1	2	3	4
B. Mushroom, sliced	1	2	3	4
C. Peanuts	1	2	3	4
D. Spaghetti	1	2	3	4
E. Chicken	1	2	3	4
F. Egg, scrambled	1	2	3	4
G. Strawberry	1	2	3	4
H. Fish, sliced	1	2	3	4
I. Broccoli	1	2	3	4
J. Beansprouts	1	2	3	4
K. Blueberry	1	2	3	4
L. Kailan	1	2	3	4
M. Beef, sliced	1	2	3	4
N. Soup, cream	1	2	3	4

Feedback Form

On a scale of 1 to 4, how representative are the included foods of those you commonly eat?

☐ 1 – Not at all representative

☐ 2 – Somewhat representative

☐ 3 – Mostly representative

☐ 4 – Highly representative

1	Rice, white	8	Fish, sliced
2	Mushroom, sliced	9	Broccoli
3	Peanuts	10	Beansprouts
4	Spaghetti	11	Blueberry
5	Chicken	12	Kailan
6	Egg, scrambled	13	Beef, sliced
7	Strawberry	14	Soup, cream

Does the range of portion sizes in the food atlas cover your typical portion size?

☐ Yes, for 1 – 4 foods

☐ Yes, for 5 – 9 foods

☐ Yes, for 10 – 14 foods

☐ No

☐ Not sure

On a scale of 1 to 4, how confident are you in using the food atlas to estimate portion sizes in your daily life?

☐ 1 – Not at all confident

☐ 2 – Somewhat confident

☐ 3 – Mostly confident

☐ 4 – Highly confident

What suggestions do you have to improve the usability of the food atlas? Select all that apply.

☐ Include a wider range of portion sizes

☐ Improve the clarity of the food images

☐ Include a reference measurement tool in the images (e.g. tablespoon, ruler)

☐ Others (please specify): ______________________________

____

____
