# Supplementary material for: Development and validation of a photographic food atlas for portion size estimation in Singapore
Source: Front Nutr. 2026 Sep 18;13:1930409. doi: 10.3389/fnut.2026.1930409 (PMC13630621; doi:10.3389/fnut.2026.1930409)
Supplement: Supplementary file 4 [file Data_Sheet_4.PDF]

S3. Statistical Models

A. Mean Difference 95% CI

Code script

```
foods <- unique(Longdata$entry)

out <- data.frame(
  entry = character(),
  N = integer(),
  Mean_Difference = numeric(),
  Lower_95CI = numeric(),
  Upper_95CI = numeric(),
  stringsAsFactors = FALSE
)

for (f in foods) {
  sub <- Longdata[Longdata$entry == f, ]
  x <- sub$difference

  n <- sum(!is.na(x))
  m <- mean(x, na.rm = TRUE)
  s <- sd(x, na.rm = TRUE)
  se <- s / sqrt(n)
  tcrit <- qt(0.975, df = n - 1)

  out <- rbind(out, data.frame(
    entry = f,
    N = n,
    Mean_Difference = m,
    Lower_95CI = m - tcrit * se,
    Upper_95CI = m + tcrit * se,
    stringsAsFactors = FALSE
  ))
}
out
```

Data output

Entry	N	Mean_Difference	Lower_95CI	Upper_95CI
Soup, cream	82	-0.20731707	-0.30335860	-0.111275545
Egg, scrambled	82	-0.07317073	-0.14030188	-0.006039580
Fish, white, boneless, sliced	82	-0.42682927	-0.55615548	-0.297503055
Blueberry	82	-0.01219512	-0.06671940	0.042329154
Strawberry	82	-0.08536585	-0.15613401	-0.014597700
Mushroom, sliced	82	-0.23170732	-0.34250816	-0.120906471
Pasta, spaghetti	82	0.20731707	0.11769615	0.296937992
Rice, white, regular	82	0.04878049	-0.01942483	0.116985806
Beef, lean, sliced	82	-0.46341463	-0.58398574	-0.342843526
Chicken, boneless, pieces	82	-0.19512195	-0.29542088	-0.094823022
Peanut	82	-0.60975610	-0.71759817	-0.501914020
Broccoli, florets	82	0.18292683	0.09745731	0.268396353
Beansprout	82	-0.02439024	-0.05849290	0.009712415
Kailan	82	-0.06097561	-0.12414613	0.002194906

B. Quadratic Weighted Kappa 95% CI

Code script

```
library(vcd)


foods <- unique(Longdata$entry)


out <- data.frame(
  entry = character(),
  N = integer(),
  kappa = numeric(),
  lower_CI = numeric(),
  upper_CI = numeric(),
  stringsAsFactors = FALSE
)


for (f in foods) {
  sub <- Longdata[Longdata$entry == f, ]


  tab <- table(
    factor(sub$correct_photo, levels = 1:4),
    factor(sub$selected_photo, levels = 1:4)
  )
```

```
  k <- Kappa(tab, weights = "Fleiss-Cohen")

  out <- rbind(out, data.frame(
    entry = f,
    N = nrow(sub),
    kappa = k$Weighted["value"],
    lower_CI = k$Weighted["value"] - 1.96 * k$Weighted["ASE"],
    upper_CI = k$Weighted["value"] + 1.96 * k$Weighted["ASE"],
    stringsAsFactors = FALSE
  ))
}

out
```

Data output

Entry	N	Kappa	Lower_CI	Upper_CI
Soup, cream	82	0.6415094	0.5250552	0.7579637
Egg, scrambled	82	0.8048780	0.6778133	0.9319428
Fish, white, boneless, sliced	82	0.8512111	0.8126096	0.8898125
Blueberry	82	0.8837209	0.7891562	0.9782856
Strawberry	82	0.8163265	0.7198904	0.9127627
Mushroom, sliced	82	0.8251748	0.7639086	0.8864410
Pasta, spaghetti	82	0.9178744	0.8888959	0.9468529
Rice, white, regular	82	0.8431373	0.7578658	0.9284087
Beef, lean, sliced	82	0.4545455	0.3367333	0.5723576
Chicken, boneless, pieces	82	0.9392097	0.9140405	0.9643790
Peanut	82	0.2307692	0.1589013	0.3026371
Broccoli, florets	82	0.7887324	0.7277560	0.8497088
Beansprout	82	0.9512195	0.8845223	1.0179168
Kailan	82	0.9565217	0.9251601	0.9878833

C. Spearman Coefficient

Excel function

For each entry, apply RANK.AVG for correct_photo and selected_photo, then use CORREL to obtain Spearman coefficient.

D. Mixed-Effects Model

Formula

$Difference_{ij} = \beta_0 + \beta_1 (Entry_{ij}) + u_i + \epsilon_{ij}$

- $Difference_{ij}$ = the difference score for participant *i* for observation *j*
- β_0 = overall intercept
- $\beta_1 (Entry_{ij})$ = fixed effect of entry
- u_i = participant random error
- ϵ_{ij} = residual error

Code script

```
Longdata$entry <- relevel(factor(Longdata$entry), ref = "Rice, white")

model <- lmer(difference ~ entry + (1 | participant), data = Longdata)

summary(model)
```

Data output

Linear mixed model fit by REML ['lmerMod']

Formula: difference ~ entry + (1 | participant)

Data: Longdata

REML criterion at convergence: 1231.7
Number of obs: 1148, groups: participant, 41

**Scaled residuals**				
**Min**	**1Q**	**Median**	**3Q**	**Max**
-3.7449	-0.4267	0.1084	0.4784	3.6526

**Random effect**		
**Group**	**Variance**	**Std. Dev.**
Participant (Intercept)	0.005417	0.0736
Residual	0.160478	0.4006

**Fixed effect**			
**Entry**	**Estimate**	**Std. Error**	**t value**
Soup, cream	-0.25610	0.06256	-4.093
Egg, scrambled	-0.12195	0.06256	-1.949
Fish, white, boneless, sliced	-0.47561	0.06256	-7.602
Blueberry	-0.06098	0.06256	-0.975
Strawberry	-0.13415	0.06256	-2.144
Mushroom, sliced	-0.28049	0.06256	-4.483
Pasta, spaghetti	0.15854	0.06256	2.534
Rice, white, regular	0.04878	0.04571	1.067
Beef, lean, sliced	-0.51220	0.06256	-8.187
Chicken, boneless, pieces	-0.24390	0.06256	-3.899
Peanut	-0.65854	0.06256	-10.526
Broccoli, florets	0.13415	0.06256	2.144
Beansprout	-0.07317	0.06256	-1.170
Kailan	-0.10976	0.06256	-1.754
